# Design principles for enantiospecific *para*- and *ortho*-[3,3] rearrangements of chiral aryl–allyl ethers

**DOI:** 10.1039/d6qo00040a

**Published:** 2026-03-13

**Authors:** Johanna Breinsperger, Maximilian Kaiser, Peter Gärtner

**Affiliations:** a Institute of Applied Synthetic Chemistry, Technische Universität Wien Getreidemarkt 9/163 1060 Wien Austria maximilian.kaiser@tuwien.ac.at

## Abstract

We report a systematic study that elucidates the regio-determining features of the stereoretentive *para*-Claisen–Cope and *ortho*-Claisen rearrangements of enantioenriched aryl–allyl ethers under mild catalytic conditions. The role of the aromatic substitution pattern as well as the nature of the rearranging ether moiety were thoroughly investigated, revealing that both *para*- and *ortho*-alkylation proceeded enantiospecifically with near-perfect chirality transfer. These findings resulted in rational design principles for accessing synthetically versatile, enantioenriched phenols and gave insights into how steric and electronic influences direct the [3,3]-rearrangements.

## Abstract 2

Regioselective allyl–aryl rearrangements directed by substituents yield chiral *ortho*- and *para*-alkylated products in high enantiomeric purity.

## Graphical abstract

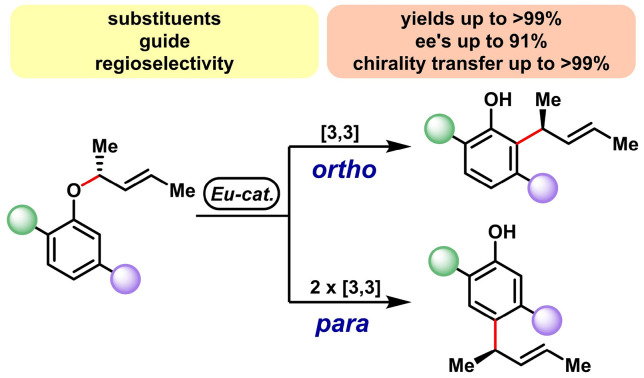

## Introduction

**Scheme 1:**
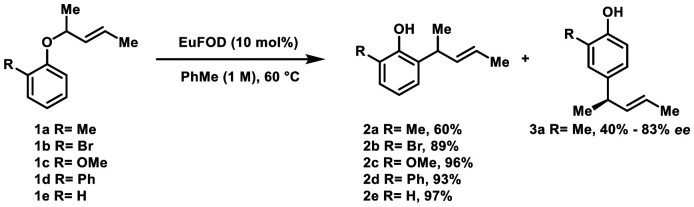
Rearrangement of mono-*ortho* substituted ethers.

We recently reported a stereoretentive *para*-Claisen–Cope rearrangement of enantioenriched 2,6-disubstituted aryl–allyl ethers into *para*-alkylated phenols with virtually perfect chirality transfer.^1^ The process proceeded under mild, air- and moisture-tolerant EuFOD-catalysis and provided access to products in excellent yields and enantiomeric excess (up to >98% *ee*). Mechanistic investigations revealed a consecutive [3,3]-sigmatropic rearrangement sequence resulting in overall retention of configuration. Nevertheless, substrates lacking the 2,6-di-substitution pattern suffered from poor selectivity, resulting in seemingly unpredictable product distributions that depended heavily on the specific reaction conditions. Further, reported examples are largely limited to simple, achiral allyl^2–5^ or prenyl^6–12^ ether chains. In our recent study, we found that *o*-cresol derived ether 1a (R = Me, 83% *ee*) rearranged into the expected *ortho*-alkylated product 2a in 60% yield (*ee* n.d.) and was accompanied by *para*-rearranged 3a in 40% yield, with excellent chirality transfer (83% *ee*). Interestingly, compounds 1b–1e only delivered *ortho*-rearrangement 2b–2e with merely trace amounts of *para*-alkylation (Scheme 1). These findings prompted a more comprehensive study to elucidate how electronic and steric effects of the aromatic substituents as well as the nature of the migrating ether chain influence the regioselectivity of this transformation. Despite growing interest and recent advances, the factors governing the *para*-to-*ortho* selectivity in aryl–allyl ether rearrangements are not well understood and require further investigation.^13–15^

**Fig. 1:**
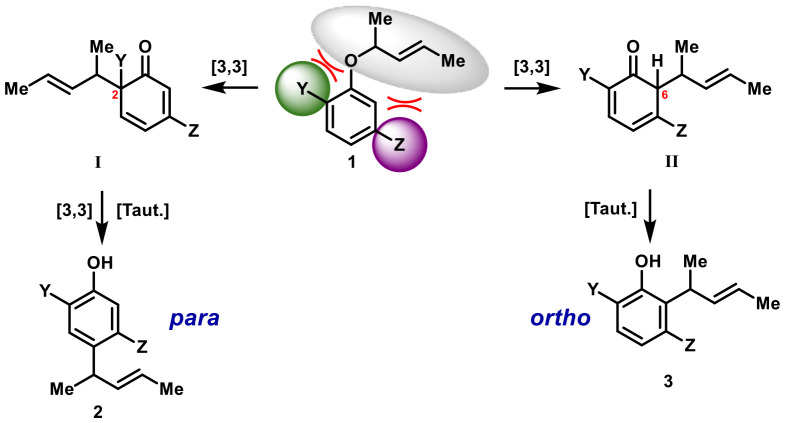
Mechanistic pathways leading to *para*- and *ortho*-alkylation.

In this work, our objective is the identification of the regio-determining features directing the (tandem-) [3,3]-sigmatropic rearrangement along the *para*-selective pathway. Therefore, our attention shifted to 2,5-disubstituted derivatives to gain a deeper mechanistic understanding of substituent interplay and its impact on the reaction outcome with the goal in mind to maximize *para*-selectivity. The products obtained *via* this method, represent versatile building blocks with multiple functional handles (Ar-**OH**, **Y**, **Z** and **olefine**-moiety, Fig. 1) otherwise difficult to access in high enantiomeric purity.^16–18^ In general, we assume that the nature of the substituents **Y** (C-2) and **Z** (C-5) in substrate 1 has the strongest influence on the regioselectivity primarily by destabilizing one of the competing reaction pathways either through steric interaction or electronic repulsion.^19^

Consequently, two plausible intermediates must be considered: I, arising from an initial [3,3] sigmatropic rearrangement onto C-2 forming a quaternary center. Subsequent second rearrangement then leads to the formation of *para*-product 2 after tautomerization. In analogy, alkylation of the unsubstituted *ortho*-position at C-6 gives rise to intermediate II, which then tautomerizes to the *ortho*-product 3.^20–22^ As depicted in Scheme 1, the exclusive formation of *ortho*-compound 2e strongly suggests that a rearrangement into *para*-position *via* an unsubstituted *ortho*-position can be ruled out as a viable reaction pathway under the applied reaction conditions. The central question now revolves around which factors dictate the preference for rearrangement onto the sterically encumbered C-2 position, rather than the comparatively “free” C-6 position. It remains unclear which properties of the adjacent C-5 substituent suppress the seemingly more accessible *ortho*-pathway, and which features of the C-2 substituent might actively promote or prevent migration onto C-2.

## Results and discussion

Compound 1f was selected as a model substrate, and the influence of solvent and temperature on product distribution as well as enantioselectivity was systematically evaluated. Applying conditions already established within our group, we subjected 1f to EuFOD-catalysis in toluene (1 M) at 40 °C to deliver 2f and 3f in a ratio of 1.7 : 1 favoring the desired *para*-alkylation (entry 1, Table 1). We were delighted to find that both regioisomers were obtained with perfect chirality transfer. Conducting the transformation in polar solvents such as EtOAc (entry 2), THF (entry 3), or CHCl_3_ (entry 4), did not lead to any product formation. When performed in hexane (entry 5), the products 2f and 3f were again obtained with excellent *ee*'s though with diminished *para*-selectivity. Interestingly, employing HFIP as the solvent resulted in exclusive formation of the *para*-product 2f (entry 6). However, major loss of stereochemical information was detected, suggesting that the highly polar medium may promote a competing ionic pathway for the transformation. Next, we investigated the temperature-effect on the *para*-to-*ortho* ratio.

**Table 1:** Optimization of reaction conditions

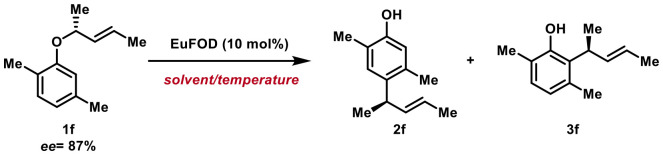				
Entry	Solvent (1 M)	Temp. [°C]	2f : 3f a	*ee* a (c.t.) in %
1	PhMe	40	1.7 : 1	87 (>99)
2	EtOAc	40	—	—
3	THF	40	—	—
4	CHCl_3_	40	—	—
5	Hexane	40	1.2 : 1	87 (>99)
6	HFIP	40	1 : 0	16 (>18)
7	Hexane	60	1.2 : 1	87 (>99)
8	Heptane	60	1.1 : 1	87 (>99)
9	PhMe	60	**1.85 : 1** b	87 (>99)
10	1,2-DCE	60	2 : 1	80 (>92)
11	1,2-DCB	60	2.3 : 1	77 (>89)
12	PhMe	80	1.6 : 1	87 (>99)
13	PhMe	100	1.5 : 1^c^	87 (>99)

a Determined by chiral HPLC of crude product or reaction mixture.

b Quantitative isolated yield.

c 91% isolated yield; 
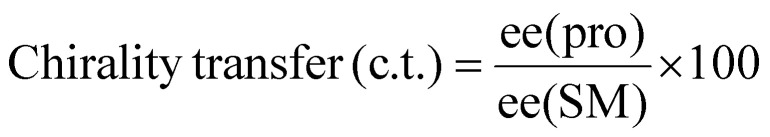
.

**Scheme 2:**
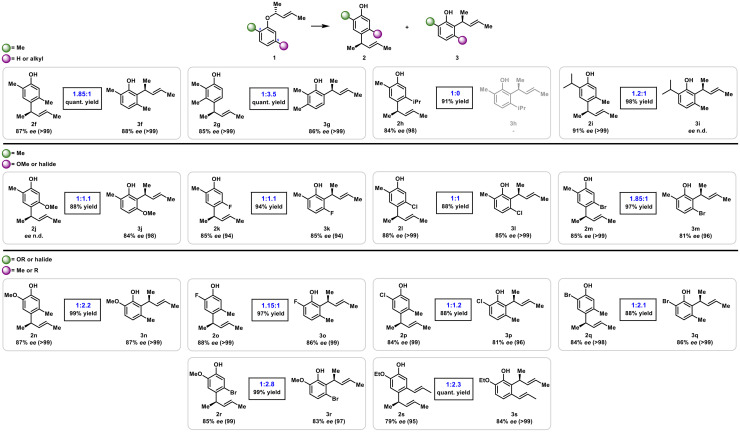
Scope and ratios of *para*- and *ortho*-alkylation.

At 60 °C, reactions in hexane (entry 7) and heptane (entry 8) gave comparable ratios of 2f : 3f while the proportion of *para*-product 2f increased to 1.85 : 1 in toluene (entry 9) with perfect chirality transfer. Switching to 1,2-DCE (entry 10) and 1,2-DCB (entry 11) favored the formation of 2f to an even greater extent but came with loss of some stereoinformation. Therefore, we proceeded with toluene as the solvent of choice and further investigated the role of reaction temperature. Going up to 80 °C (entry 12) or 100 °C (entry 13) progressively decreased the amount of *para*-product. Though the excellent transfer of stereochemistry stayed intact, the yield dropped slightly at higher temperatures (see entry 13). As 60 °C appeared to be the sweet spot for efficient *para*-alkylation, we then turned the attention towards the scope of the rearrangement to elucidate the influence of the substitution pattern on the *para*-to-*ortho* product distribution. The discussed rearrangements proceeded with high yields and excellent chirality transfer throughout, giving rise to *para*- and *ortho*-alkylated products with high enantioselectivities. As already depicted above (Table 1) the 2,5-dimethyl substitution pattern favors *para*-rearrangement over *ortho*-alkylation, furnishing a ratio of 2f : 3f of 1.85 : 1 in quantitative yield (Scheme 2). Seemingly, a C-5 substituent can significantly block the C-6 position, forcing the rearrangement to largely proceed *via* a type-I intermediate, giving rise to the *para*-rearrangement as major product.

In contrast, the 2,3-dimethyl substitution pattern favored *ortho*-rearranged 2g (1 : 3.5, quant. yield), over *para*-alkylation 3g indicating that a *meta*-substituent (at C-3 or C-5) can effectively prevent the formation of intermediates I/II, respectively, thereby influencing product distribution. Consistent with this interpretation, the sterically demanding 5-iPr enforced exclusive *para*-rearrangement (2h, 91% yield), whereas the corresponding *ortho*-product 3h was not detected.

This observation is consistent with literature precedents showing that a bulky *meta*-substituent exerts a shielding effect, impeding or at least strongly disfavouring rearrangement into the adjacent position on the aromatic ring.^23–25^ Inversion of this substitution pattern (2-iPr/5-Me) furnished 2i : 3i in a ratio of 1.2 : 1, reflecting increased steric congestion at C-2 compared to (2-Me/5-Me). At first, this result was surprising, as the 2-isopropyl substituent was expected to fully block the *ortho*-position, as seen for the 5-isopropyl derivative 2h. However, our recent work showed that 2,6-diisopropyl ethers readily undergo *para*-rearrangement, demonstrating that allyl groups can migrate to positions bearing a bulky isopropyl substituent.^1^

**Scheme 3:**
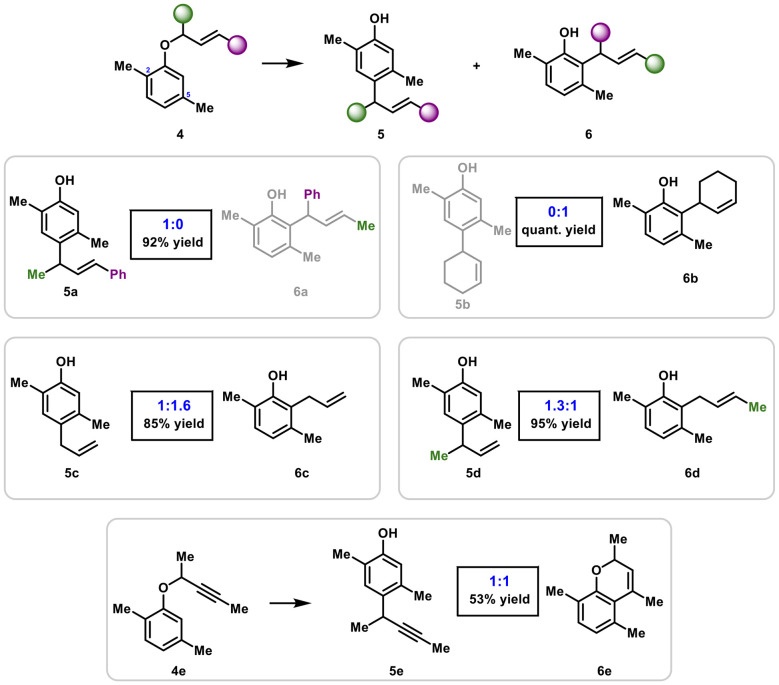
Influence of the ether moiety on *para*-to-*ortho* product distribution.

Next, we investigated various other C-5 substituents and their effects on product distribution while leaving the C-2 Me group unchanged. Introduction of 5-OMe led to the formation of 2j : 3j in excellent yield with slight preference for *ortho*-rearrangement (1 : 1.1). Since size difference between “OMe” and “Me” cannot explain this observation, it is assumed that the strong +M effect of the MeO-substituent favors a type-II intermediate, consequently favoring an *ortho*-rearrangement pathway. Incorporation of halides at C-5 furnished comparable ratios for 5-F (1 : 1.1 2k : 3k) and 5-Cl (1 : 1 2l : 3l), both in excellent combined yields. Introduction of a bromine into the 5-position again favors the *para*-pathway, affording a product distribution of 2m : 3m in a 1.85 : 1 ratio. This observation is likely a consequence of the greater steric bulk of Br relative to F and Cl, leading to effective shielding of the C-6 position. With these initial trends defined we broadened our focus from 2-alkyl substituents to explore diverse 2,5-substitution patterns. Introducing a strong electron donating OMe group into C-2 while having a C-5 Me group in place gave rise to *ortho*-alkylation as the major product (2n : 3n 1 : 2.2) in near quantitative yield. This result demonstrates the significance of electronic effects as the preference for *ortho*-selectivity cannot be reasoned by steric arguments alone when compared to 2f : 3f or 2i : 3i, respectively. Next, rearrangements of 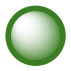 = halide, 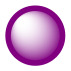 = Me substrates were investigated (see Fig. 1 for green and violet balls). For these substrates, we assumed that the product distribution might be determined by a combination of electronic and steric effects to a varying degree, depending on the halogen atom attached. We commenced with halide exhibiting the strongest +M-effect and the rearrangement of 2-F aryl-allyl ether furnished products 2o : 3o in a ratio of 1.15 : 1. In contrast to the strong electron donor 2-OMe (2n : 3n), *para*-alkylation was favored over *ortho*-rearrangement. Introduction of 2-Cl again prepared slightly more of the *ortho*-product (1 : 1.2 2p : 3p), whereas the more sterically demanding 2-Br significantly favored *ortho*-alkylation (1 : 2.1 2q : 3q). The substitution pattern of 2-OMe 5-Br gave rise to a 1 : 2.8 ratio of 2r : 3r favoring *ortho*-rearrangement. Similarly, 2-OEt 5-isoallyl furnished 2s : 3s in a ratio of 1 : 2.3. These results might be reasoned by electronic repulsion of the 2-alkoxy oxygen and the rearranging olefine moiety. In summary, product distribution can be rationalized primarily by steric effects for alkyl substituents, whereas electronic contributions might be an additional factor for halogen substituents. For OR-substituents, we suggest that, beyond steric demand and electronic repulsion, an additional factor contributes to the relatively high *ortho*-selectivity. We assume that the intermediate that consists of a conjugated enol ether moiety is favored, hence preferentially formed. Specifically, 2-OR groups are proposed to react preferentially *via* a type-II intermediate, which retains a conjugated enol ether motif. By contrast, a type-I pathway would disrupt this conjugation. This rationale accounts for the enhanced *ortho*-selectivity observed with 2-OR substituents and for the diminished *para* selectivity for 5-OR substrates relative to 2f/3f (2-Me, 5-Me), despite the potentially stronger steric and electronic influence onto a free *ortho*-position exerted by a 5-OR group. With the aromatic substitution pattern thoroughly investigated, we turned the focus towards the ether moiety. In case of 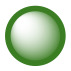 = Me, 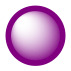 = Ph, the rearrangement delivered *para*-alkylated product 5a exclusively in 92% yield (Scheme 3). We reason that a type-II intermediate was unfavored due to steric interaction of the phenyl group of the migrating ether chain and the C-5 methyl group.

Further, we were delighted to find that cyclic ethers were also very well tolerated, though in this case, the reaction was completely *ortho*-selective forming compound 6b as sole product in quantitative yield. Here, we assume that the steric congestion at C-2 would be too severe, hence the rearrangement selectively takes place at C-6. Simple allyl-ethers gave rise to a 1 : 1.6 5c : 6c product mixture favoring *ortho*-alkylation. This observation fits well with previous observations as steric interactions between C-5 methyl and a “slim” ether moiety is most likely favored over quaternarization at C-2. In case of 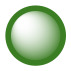 = Me, 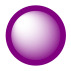 = H, the *para*-rearrangement pathway is slightly favored over *ortho* and compounds 5d and 6d are obtained in excellent combined yield in a 1.3 : 1 ratio. Finally, rearrangement of an alkyne ether (at 110 °C) gave rise to a 1 : 1 mixture of *para*-alkylated alkyne 5e and cyclic product 6e in moderate yield. The latter product is most likely formed *via* an intramolecular electrophilic hydroarylation.^26^ Lastly, strong electron-withdrawing substituents at the aromatic core represent a significant challenge and the desired rearrangements proved unfeasible (see SI for details).

## Conclusion

In this comparative study of enantioenriched 2,5-substituted aryl–allyl ethers, we were able to elucidate how different types of substituents dictate the reaction pathway between *ortho*- and *para*-rearrangement. Through systematic variation of the substituent pattern, it was possible to shed light onto the steric and electronic factors governing regioselectivity. Furthermore, we could demonstrate that the rearrangement into the *ortho*- and the *para*-position, respectively, proceed enantiospecifically and both regioisomers were obtained in high enantioselectivites with virtually perfect transfer of chirality. Insights from these model systems help clarify the interplay of substituent effects and reaction outcome, thereby enhancing the predictability and synthetic utility of this stereoretentive transformation.

## Author contributions

All authors have approved the final version of the manuscript. Formal analysis, writing – original draft: Johanna Breinsperger; methodology, investigation, data curation, writing – review & editing: Johanna Breinsperger, Maximilian Kaiser; conceptualization, project administration: Maximilian Kaiser; supervision and funding acquisition: Peter Gärtner.

## Conflicts of interest

There are no conflicts to declare.

## Supplementary Material

QO-013-D6QO00040A-s001

## Data Availability

The data supporting the findings of this study are available within the article and its Supplementary information (SI). Supplementary information is available. See DOI: https://doi.org/10.1039/d6qo00040a. Additional data underlying this work is available from the corresponding author upon reasonable request.
