# Supplementary material for: Design principles for enantiospecific para- and ortho-[3,3] rearrangements of chiral aryl–allyl ethers
Source: Org Chem Front. 2026 Mar 13;13(9):2805–9. doi: 10.1039/d6qo00040a (PMC12997403; doi:10.1039/d6qo00040a)

Supplementary Information (SI) for Organic Chemistry Frontiers.
This journal is © the Partner Organisations 2026

Supporting Information

Design Principles for Enantiospecific *para*- and *ortho*-[3,3] Rearrangements of Chiral Aryl–Allyl Ethers

Johanna Breinsperger, Maximilian Kaiser, Peter Gärtner*

Table of Contents

Table of Contents 2
General information 3
Synthetic procedures + compound characterization 4

General information

The reactions were performed as described in the general procedures. All reactions were stirred magnetically.

Phenols were purchased from commercial suppliers and used as received.

Dry toluene, dichloromethane and tetrahydrofuran were retrieved from an Innovative Technologies PureSolv system. Dichloromethane was degassed by two freeze-thaw cycles.

^{1}H and ^{13}C NMR spectra were recorded on a Bruker AC 400 at 400 and 101 MHz; AC 600 at 600 and 151 MHz using the solvent peak as reference. ^{13}C NMR spectra were run in proton-decoupled mode. Multiplicities of 1H signals were referred to as s (singlet), d (doublet), t (triplet), q (quartet) and more complex patterns or m (multiplet). TLC-analysis was done with precoated aluminum-backed plates (Silica gel 60 F254, Merck). Compounds were visualized by submerging in: an acidic phosphomolybdic acid / Cerium sulphate solution, $KMnO_4$, Vanillin or Anisaldehyde and dried with a heat gun. Column chromatography was carried out with silica gel Merck 60. Eluent systems refer to volumetric ratios, e.g., 4:1 =80%: 20%.

Chiral HPLC measurements were carried out on a DIONEX UPLC equipped with a photodiode array (PDA) plus detector (190–360 nm), using Diacel Chiralcel IB, OJ-3, OD and IA-3 columns (all 250 x 4.60 mm, 5 µm).

HRMS measurements were carried out in acetonitrile, methanol, water or a mixture on an Agilent 1100/1200 HPLC with binary pumps, a degassed and a column thermostat and an Agilent 6230 AJS ESI-TOF mass spectrometer.

Specific rotations were measured on an Anton Parr MCP 500 polarimeter at 20 °C and 589 nm.

Synthetic procedures + compound characterization

General procedure A – Synthesis of enantioenriched ethers 1

A flame dried Schlenk flask was charged with carbonate **Carb 1** (1.1 equiv.), $Pd_2dba_3{*}CHCl_3$ (2 mol%), *R,R*-DACH- ligand (6 mol%) and dissolved in dry degassed DCM (0.3 M). After 15 minutes, during which a color change from red to green was observed, the respective phenol (1 equiv.) was added in dry degassed DCM (0.3 M). The mixture was stirred under argon atmosphere for 19 hours. After TLC confirmed full consumption of starting material, the reaction was filtered over a plug of silica (petroleum ether/ethyl acetate 10:1) and concentrated *in vacuo*. The crude material was subjected to column chromatography.

*Racemic ethers **1*** for HPLC analysis were synthesized as follows:

A round bottom flask was charged with phenol (1 equiv.), carbonate **Carb 1**, **Carb 2** or **Carb 3** (1.1 equiv.), $Pd(PPh_3)_4$ (4 mol%) and dissolved in dry degassed DCM (0.1 M). The mixture was stirred until TLC confirmed full consumption of starting material. The reaction was filtered over a plug of silica (petroleum ether/ethyl acetate 10:1) and concentrated *in vacuo*. The crude material was purified by column chromatography.

General procedure B –Rearrangement

An 8 mL screw neck vial was charged with starting material (1 equiv.) and EuFOD (10 mol%) in dry toluene (1 M). The reaction was heated to 60 °C in a metal heating block until TLC showed full conversion (overnight). The reaction was directly purified by column chromatography.

General procedure C – Ozonolysis + reductive work-up

A Schlenk flask was charged with the starting material (1 equiv.) dissolved in DCM/MeOH (1:1, 0.05 M) and cooled to -80 °C. A stream of ozone was bubbled through the solution until it took on a deep blue color. After 7 minutes of further stirring, a stream of oxygen was bubbled through the solution until the blue color disappeared. Subsequently, sodium borohydride (4 equiv.) was added at -80 °C and the reaction was allowed to reach room temperature. After stirring at room temperature for 30 minutes, saturated NH_4Cl solution was added. The aqueous phase was extracted with DCM three times, the combined organic layer was dried over $MgSO_4$, filtered and concentrated *in vacuo*. The crude material was received either sufficiently pure or was subjected to column chromatography.

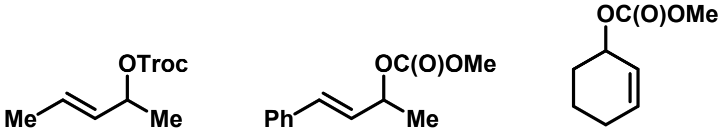

Carb 1 Carb 2 Carb 3

***(R,E)*-1,4-Dimethyl-2-(pent-3-en-2-yloxy)benzene (1f)**

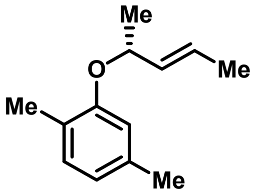

The title compound was synthesized from commercially available 2,5-dimethylphenol (302 mg, 2.47 mmol) following **general procedure A**. The crude material was purified by column chromatography (petroleum ether/ethyl acetate 40:1) to provide the desired product **1f** as colorless oil in 98% yield (462 mg, 2.43 mmol).

$[\alpha]^{20}$= + 30.09 (c 0.85, CH_2Cl_2).

^{1}H NMR (400 MHz, $CDCl_3$) δ 7.03 (dd, *J* = 7.4, 3.1 Hz, 1H), 6.68 (d, *J* = 8.3 Hz, 2H), 5.80 – 5.65 (m, 1H), 5.65 – 5.48 (m, 1H), 4.76 (h, *J* = 6.9 Hz, 1H), 2.32 (d, *J* = 2.9 Hz, 3H), 2.21 (d, *J* = 3.5 Hz, 3H), 1.79 – 1.66 (m, 3H), 1.45 – 1.39 (m, 3H).

^{13}C NMR (101 MHz, $CDCl_3$) δ 156.3, 136.3, 132.8, 130.5, 126.6, 124.7, 121.0, 114.8, 74.7, 21.7, 21.5, 17.9, 16.2.

87% *ee* (determined by chiral HPLC: Chiralpak® IB column, n-Heptane/iPrOH = 99.9:0.1, 0.3 mL/min, λ = 287.3 nm, 25 °C), minor enantiomer. t_r = 15.10 min, major enantiomer. t_r = 16.23 min.

^1H NMR (400 MHz, $CDCl_3$)

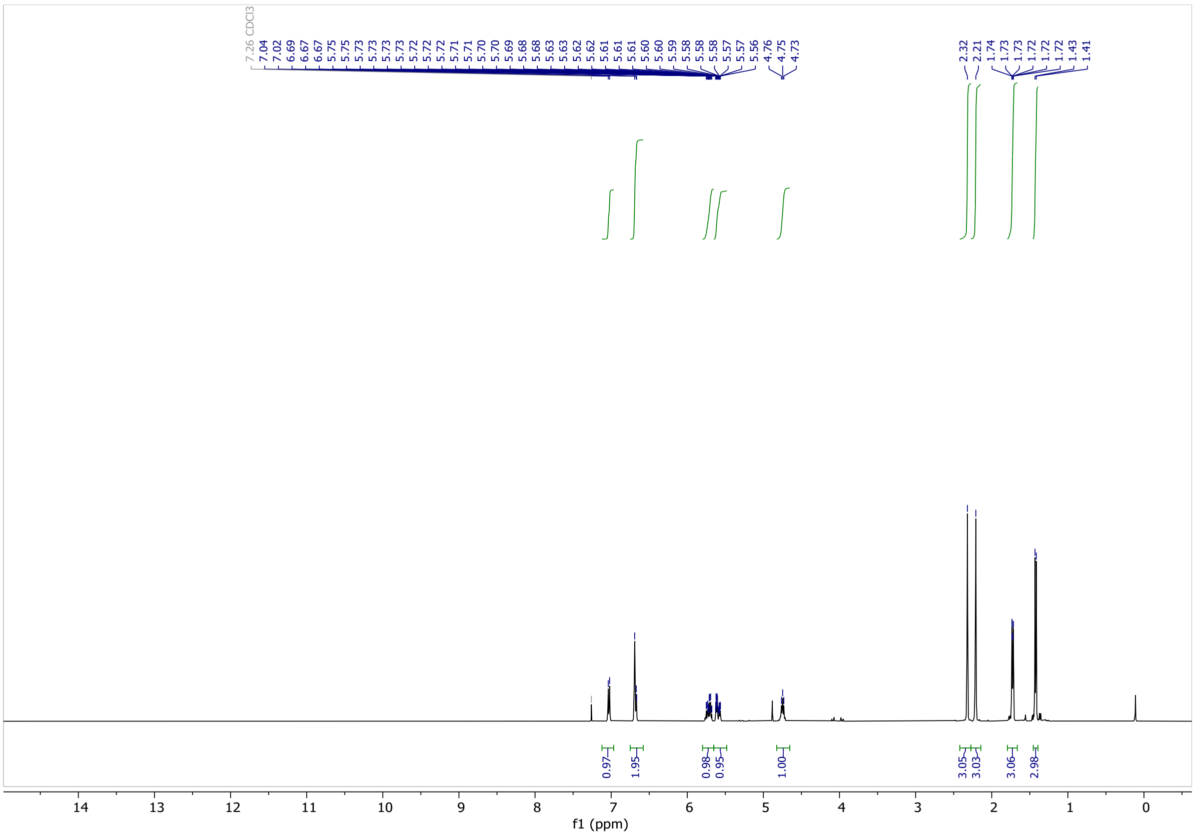

^{13}C NMR (101 MHz, $CDCl_3$)

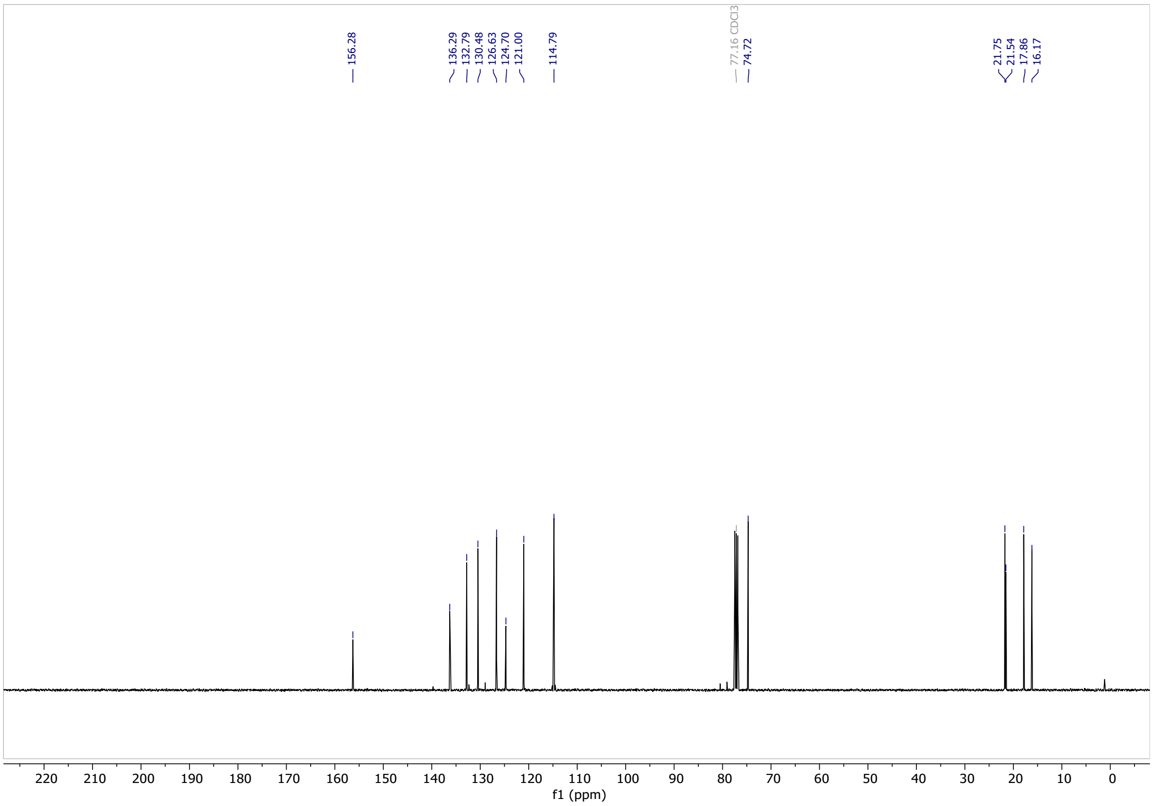

Chromatogram and Results

Instrument Method:	**Heptane_EtOH_99.9_0.1_0.3mlmin_25C_30min**	*B %:*	**0,0**
Column:	**IB**	*C %:*	**0,0**
Run Time (min):	**30,00**	*D %:*	**0,1**
Channel:	**UV_VIS_1**		
Wavelength:	**287,26**		

Chromatogram

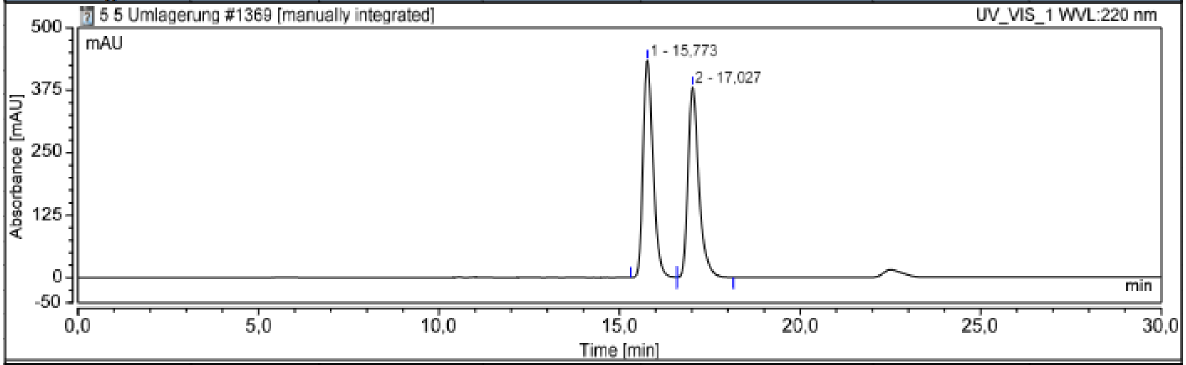

Integration Results

No.	Peak Name	Retention Time<br>min	Area<br>mAU*min	Height<br>mAU	Relative Area<br>%	Relative Height<br>%
1		15,773	138,157	433,376	51,00	53,26
2		17,027	132,731	380,323	49,00	46,74
**Total:**			**270,888**	**813,699**	**100,00**	**100,00**

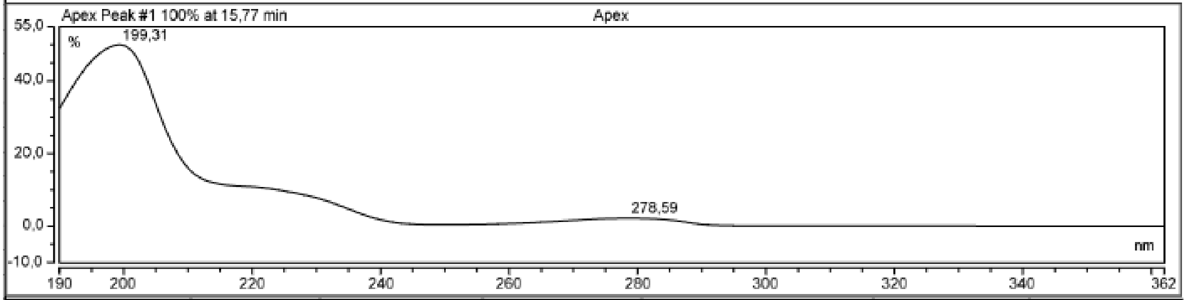

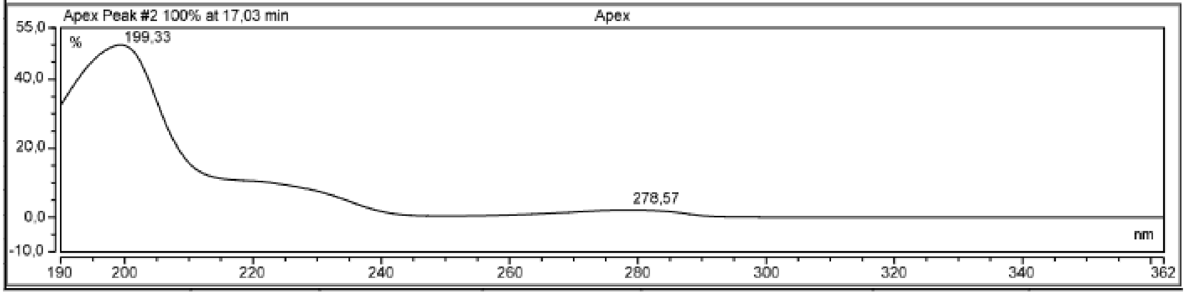

Chromatogram and Results

Instrument Method:	**Heptane_IPA_99.9_0.1_0.3mlmin_25C_30min**	*B %:*	**0,1**
Column:	**IB**	*C %:*	**0,0**
Run Time (min):	**30,00**	*D %:*	**0,0**
Channel:	**UV_VIS_1**		
Wavelength:	**287,26**		

Chromatogram

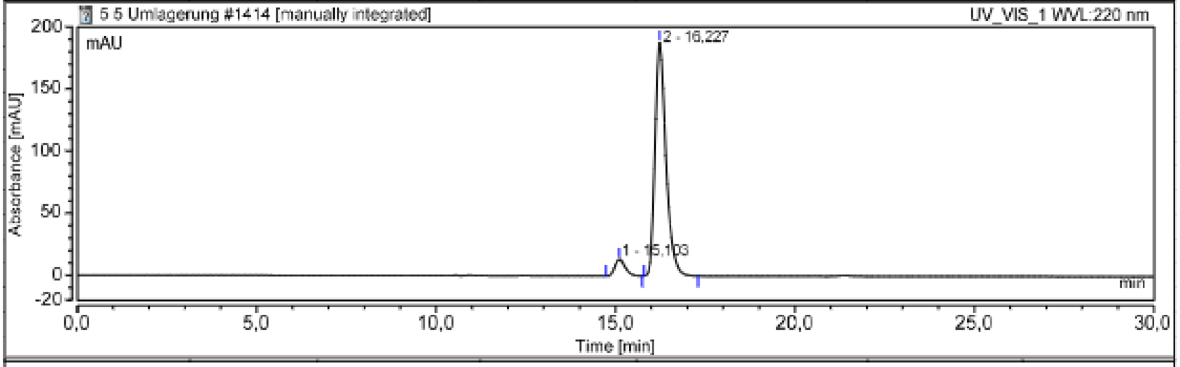

Integration Results

No.	Peak Name	Retention Time min	Area mAU*min	Height mAU	Relative Area %	Relative Height %
1		15,103	4,499	13,615	6,32	6,73
2		16,227	66,685	188,775	93,68	93,27
**Total:**			**71,183**	**202,390**	**100,00**	**100,00**

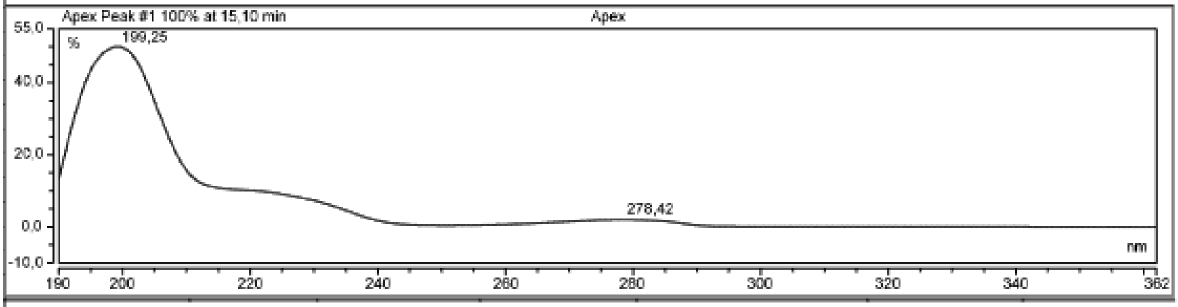

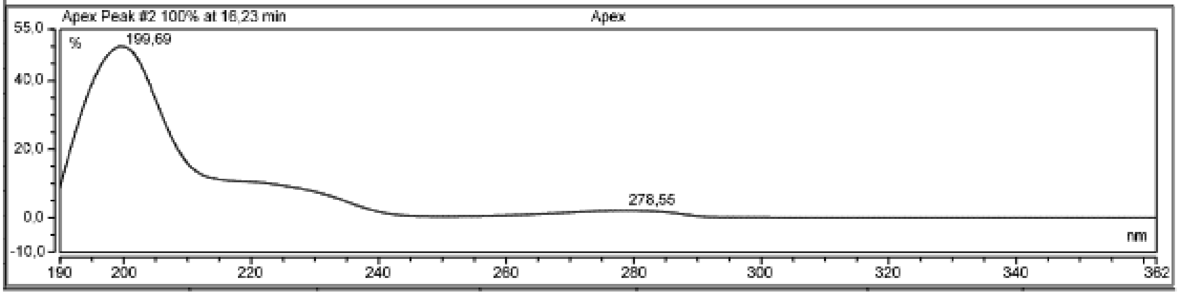

***(R,E)*-1,2-Dimethyl-3-(pent-3-en-2-yloxy)benzene (1g)**

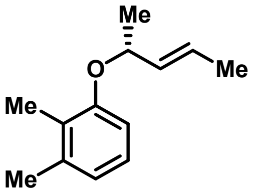

The title compound was synthesized from commercially available 2,3-dimethylphenol (158 mg, 1.29 mmol) following **general procedure A**. The crude material was purified by column chromatography (petroleum ether/ethyl acetate 40:1) to provide the desired product **1g** as colorless oil in 88% yield (217 mg, 1.29 mmol).

$[\alpha]^{20}$= + 0.75 (c 1.10, CH_2Cl_2).

^{1}H NMR (400 MHz, $CDCl_3$) δ 7.01 (t, *J* = 7.9 Hz, 1H), 6.75 (t, *J* = 8.3 Hz, 2H), 5.78 – 5.62 (m, 1H), 5.57 (ddt, *J* = 15.4, 6.3, 1.5 Hz, 1H), 4.71 (p, *J* = 6.5 Hz, 1H), 2.28 (d, *J* = 1.4 Hz, 3H), 2.17 (d, *J* = 1.7 Hz, 3H), 1.70 (dt, *J* = 6.2, 1.3 Hz, 3H), 1.41 (dd, *J* = 6.4, 1.5 Hz, 3H).

^{13}C NMR (101 MHz, $CDCl_3$) δ 156.2, 138.0, 132.9, 126.8, 126.4, 125.6, 122.3, 111.9, 75.1, 21.8, 20.3, 17.9, 12.0.

86% *ee* (determined by chiral HPLC: Chiralpak® IB column, n-Heptane/EtOH = 99.9:0.1, 0.5 mL/min, λ = 287.3 nm, 25 °C), minor enantiomer. t_r = 11.06 min, major enantiomer. t_r = 13.35 min.

^{1}H NMR (400 MHz, $CDCl_3$)

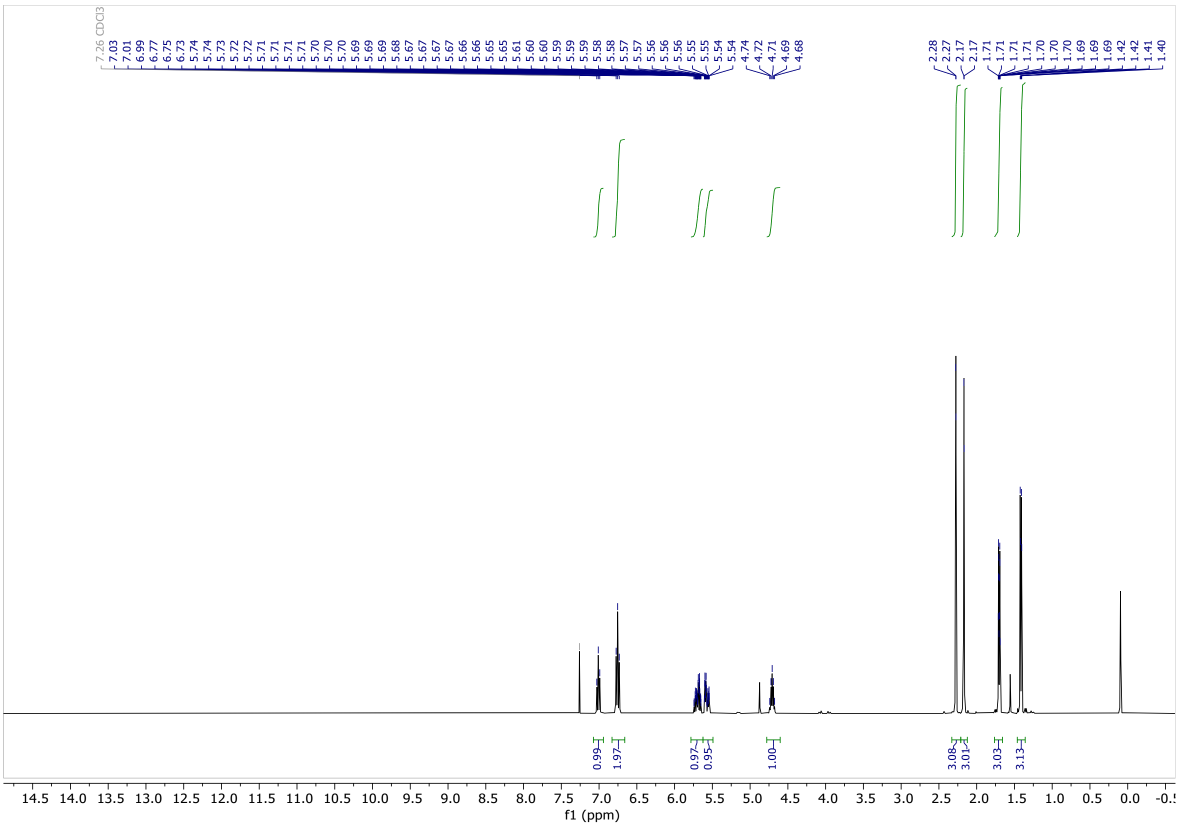

^{13}C NMR (101 MHz, $CDCl_3$)

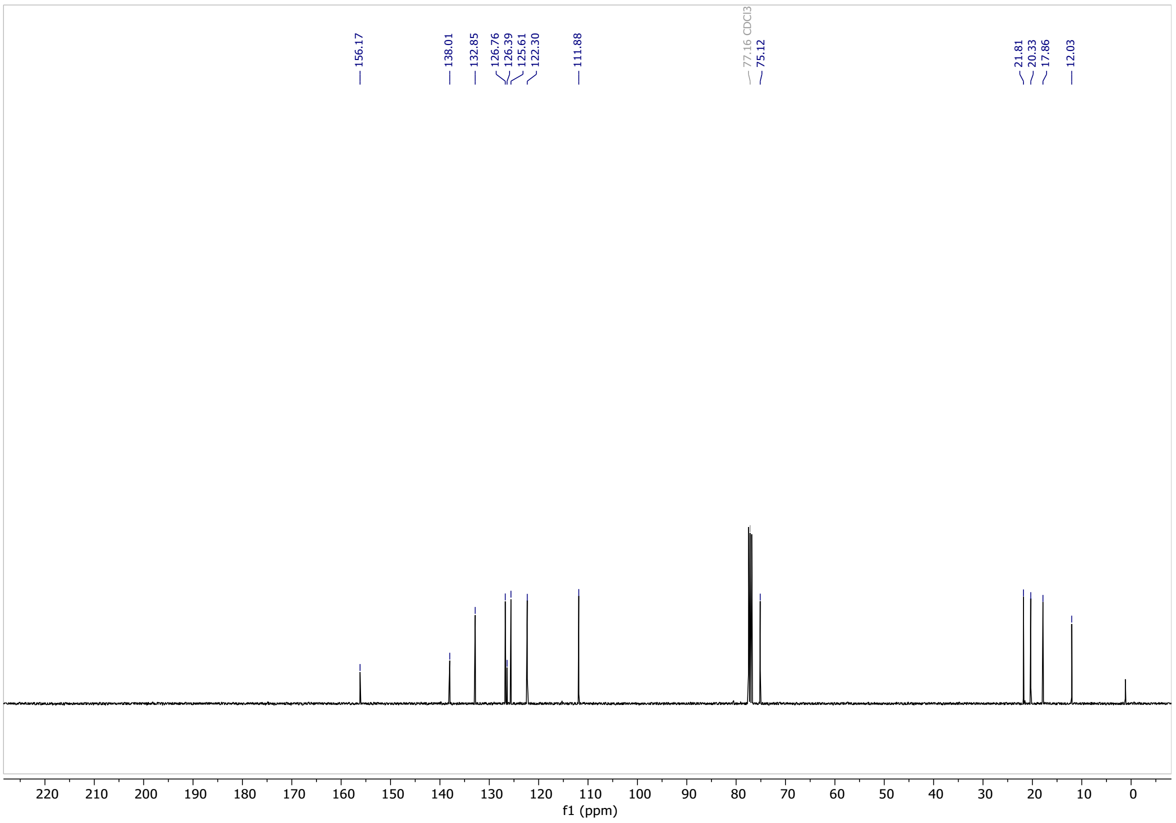

Chromatogram and Results

Instrument Method:	**Heptane_EtOH_99.9_0.1_0.5mlmin_25C_20min**	*B %:*	**0,0**
Column:	**IB**	*C %:*	**0,0**
Run Time (min):	**20,00**	*D %:*	**0,1**
Channel:	**UV_VIS_1**		
Wavelength:	**287,26**		

Chromatogram

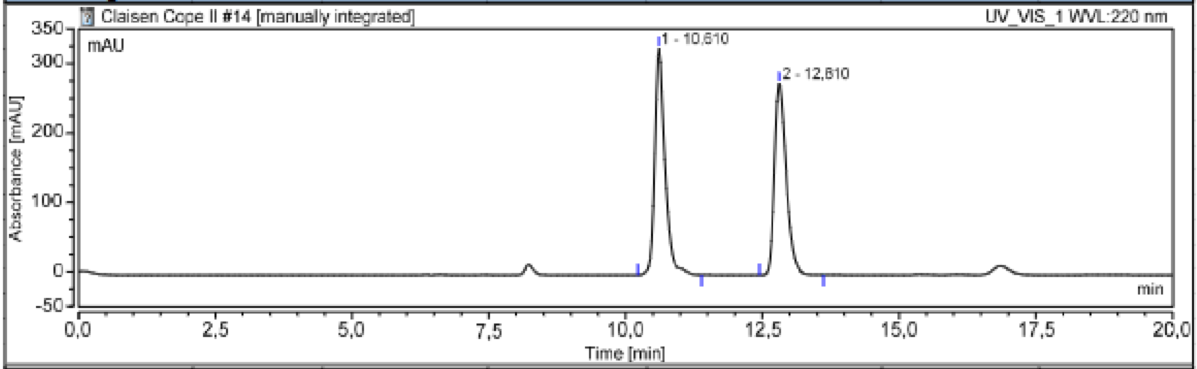

Integration Results

No.	Peak Name	Retention Time<br>min	Area<br>mAU*min	Height<br>mAU	Relative Area<br>%	Relative Height<br>%
1		10,610	71,630	327,143	51,05	54,22
2		12,810	68,692	276,187	48,95	45,78
**Total:**			**140,322**	**603,330**	**100,00**	**100,00**

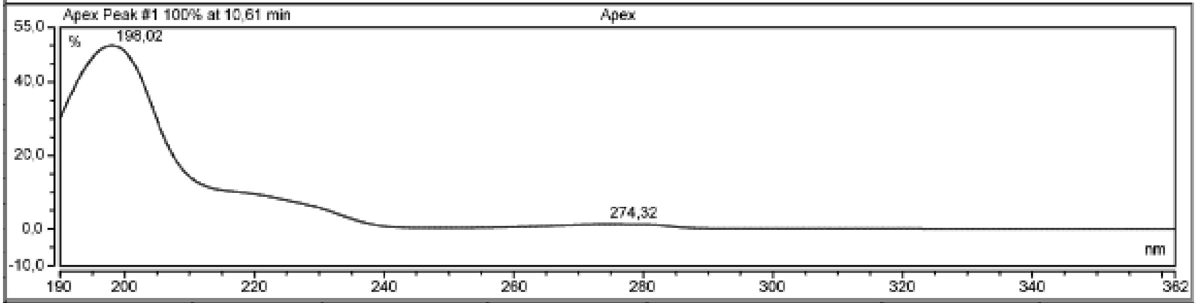

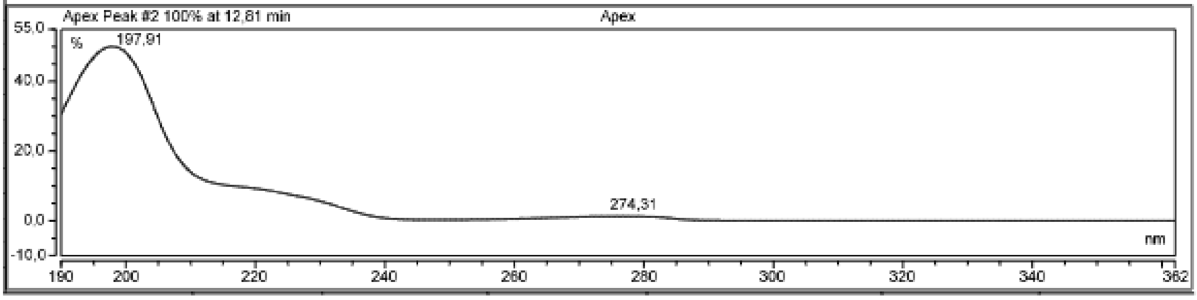

Chromatogram and Results

Instrument Method:	**Heptane_EtOH_99.9_0.1_0.5mlmin_25C_20min**	*B %:*	**0,0**
Column:	**IB**	*C %:*	**0,0**
Run Time (min):	**20,00**	*D %:*	**0,1**
Channel:	**UV_VIS_1**		
Wavelength:	**287,26**		

Chromatogram

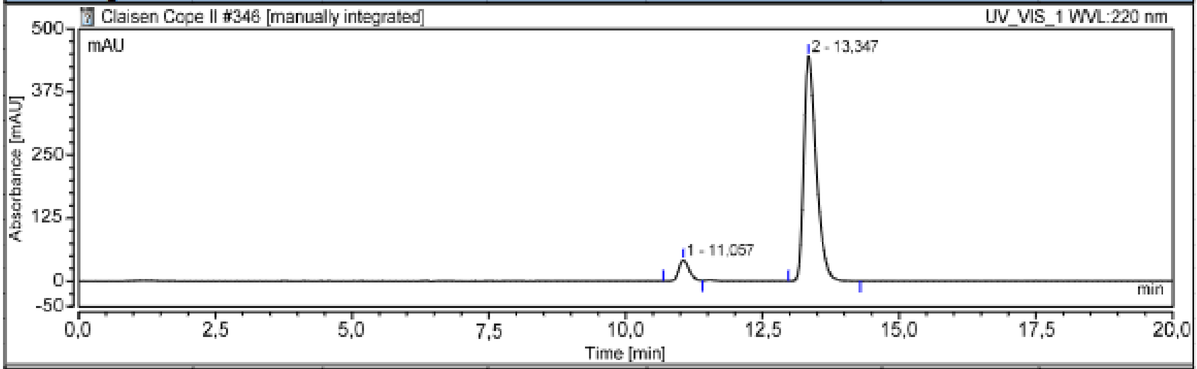

Integration Results

No.	Peak Name	Retention Time<br>min	Area<br>mAU*min	Height<br>mAU	Relative Area<br>%	Relative Height<br>%
1		11,057	9,198	41,667	7,25	8,53
2		13,347	117,598	446,781	92,75	91,47
**Total:**			**126,796**	**488,448**	**100,00**	**100,00**

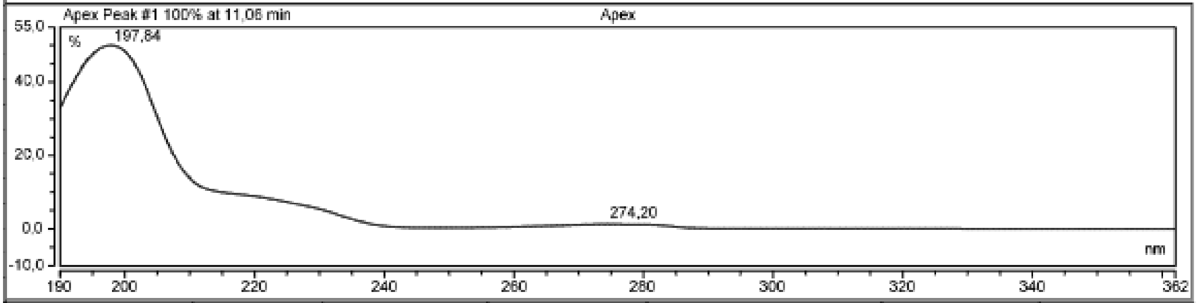

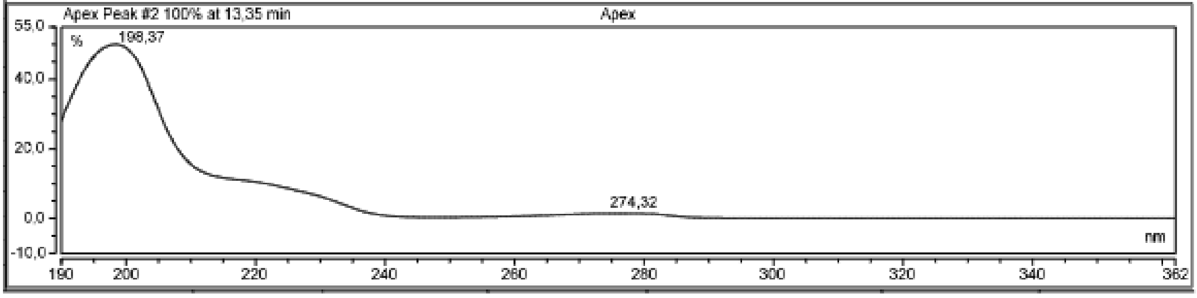

(R,E)-4-Isopropyl-1-methyl-2-(pent-3-en-2-yloxy)benzene (1h)

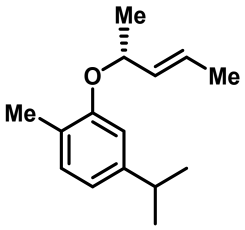

The title compound was synthesized from commercially available 5-isopropyl-2-methylphenol (161 mg, 1.02 mmol) following **general procedure A**. The crude material was purified by column chromatography (petroleum ether/ethyl acetate 40:1) to provide the desired product **1h** as colorless oil in quantitative yield (222 mg, 1.02 mmol).

$[\alpha]^{20}$= + 41.37 (c 0.90, CH_2Cl_2).

^{1}H NMR (400 MHz, $CDCl_3$) δ 7.04 (d, *J* = 7.5 Hz, 1H), 6.79 – 6.65 (m, 2H), 5.71 (dqt, *J* = 15.2, 6.3, 1.3 Hz, 1H), 5.65 – 5.46 (m, 1H), 4.74 (p, *J* = 6.5 Hz, 1H), 2.84 (ddd, *J* = 12.7, 7.5, 6.2 Hz, 1H), 2.19 (d, *J* = 1.4 Hz, 3H), 1.70 (dq, *J* = 6.4, 1.2 Hz, 3H), 1.41 (dd, *J* = 6.3, 1.4 Hz, 3H), 1.23 (dt, *J* = 6.9, 1.2 Hz, 6H).

^{13}C NMR (101 MHz, $CDCl_3$) δ 156.3, 147.6, 132.9, 130.5, 126.9, 125.2, 118.3, 112.4, 74.8, 34.1, 24.3, 24.2, 21.8, 17.9, 16.2.

86% *ee* (determined by chiral HPLC: Chiralcel® OJ3 column, n-Heptane/iPrOH = 99.9:0.1, 0.2 mL/min, λ = 287.3 nm, 25 °C), minor enantiomer. t_r = 19.17 min, major enantiomer. t_r = 20.41 min.

^{1}H NMR (400 MHz, $CDCl_3$)

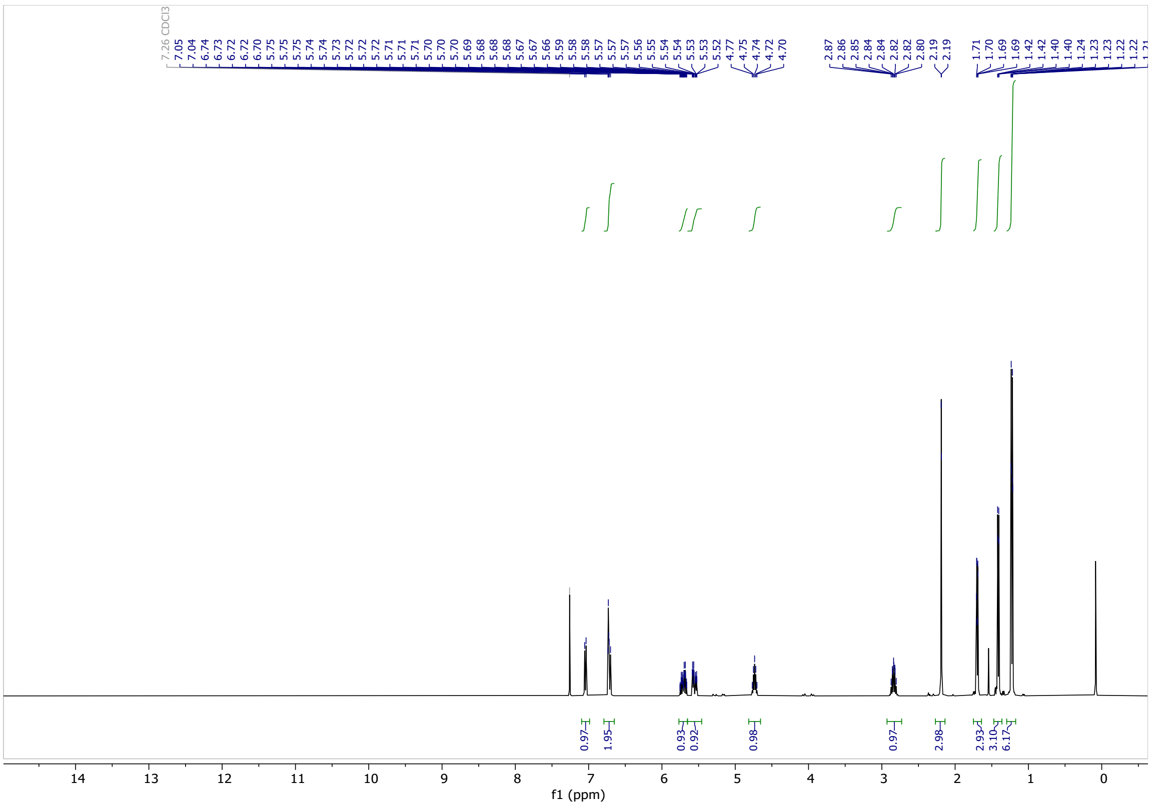

^{13}C NMR (101 MHz, $CDCl_3$)

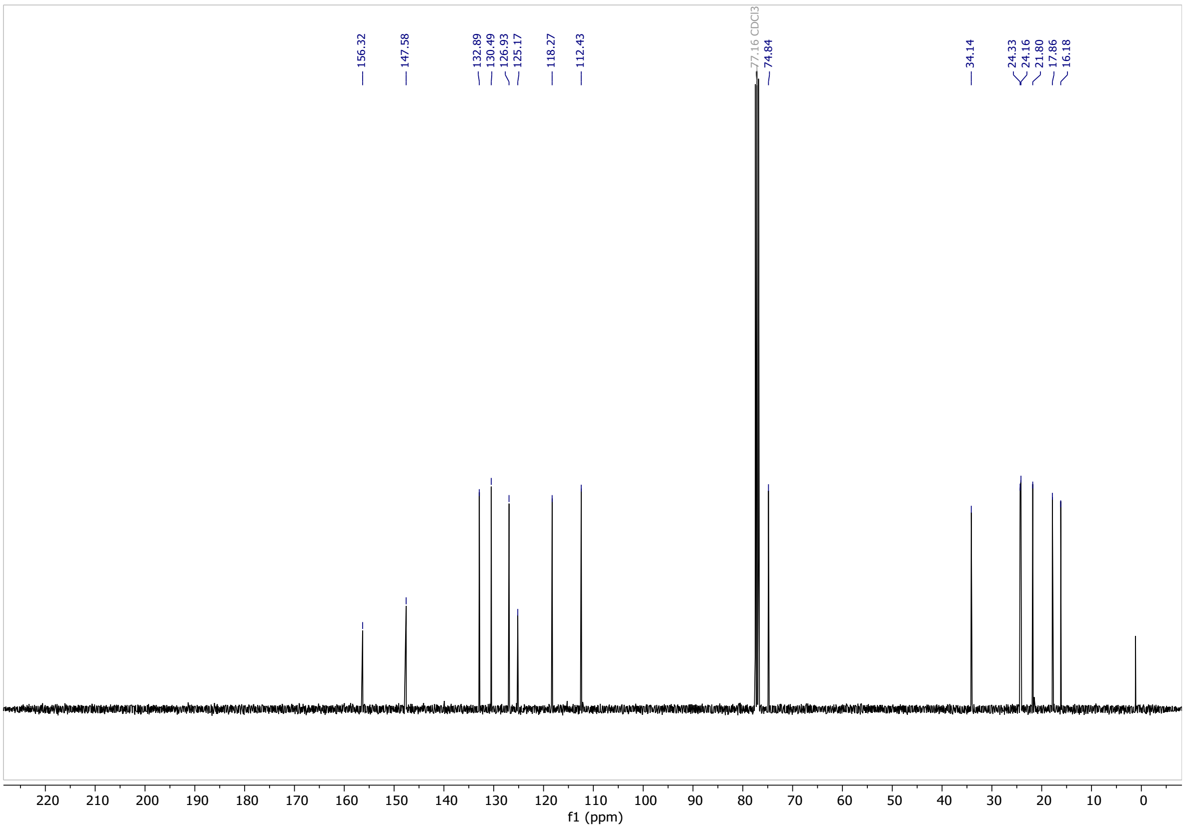

Chromatogram and Results

Instrument Method:	**Heptane_IPA_99.9_0.1_0.2mlmin_25C_30min-MK**	*B %:*	**0,1**
Column:	**OJ3**	*C %:*	**0,0**
Run Time (min):	**30,00**	*D %:*	**0,0**
Channel:	**UV_VIS_1**		
Wavelength:	**287,26**		

Chromatogram

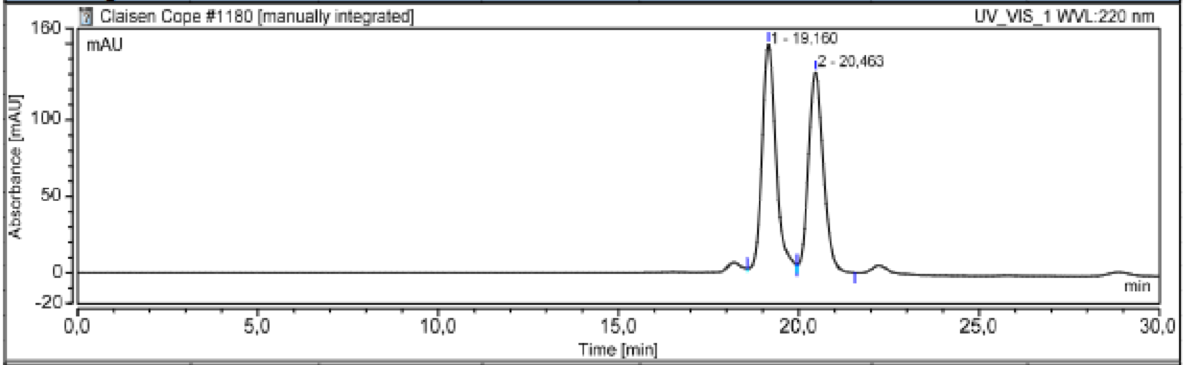

Integration Results

No.	Peak Name	Retention Time<br>min	Area<br>mAU*min	Height<br>mAU	Relative Area<br>%	Relative Height<br>%
1		19,160	67,991	149,967	51,78	53,26
2		20,463	63,312	131,622	48,22	46,74
**Total:**			**131,303**	**281,588**	**100,00**	**100,00**

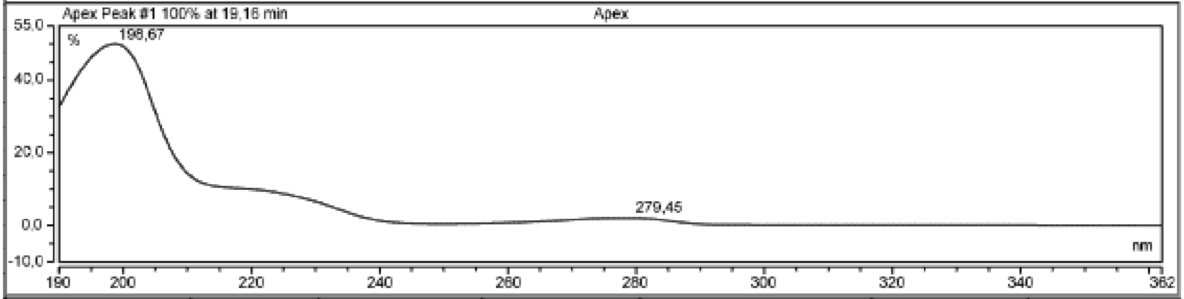

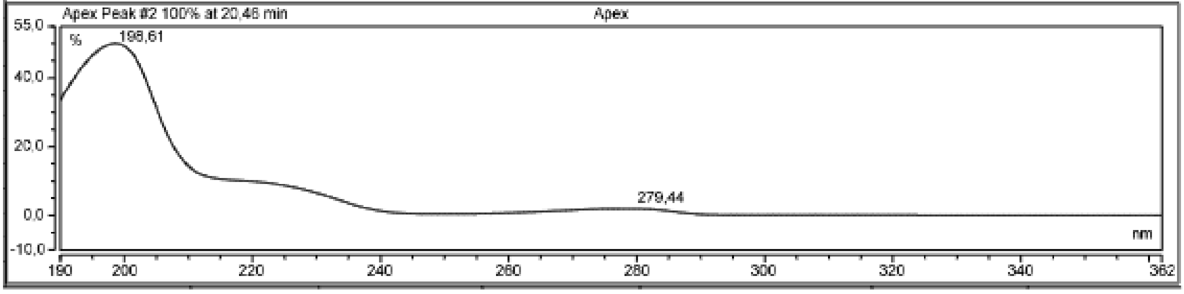

Chromatogram and Results

Instrument Method:	**Heptane_IPA_99.9_0.1_0.2mlmin_25C_30min-MK**	*B %:*	**0,1**
Column:	**OJ3**	*C %:*	**0,0**
Run Time (min):	**30,00**	*D %:*	**0,0**
Channel:	**UV_VIS_1**		
Wavelength:	**287,26**		

Chromatogram

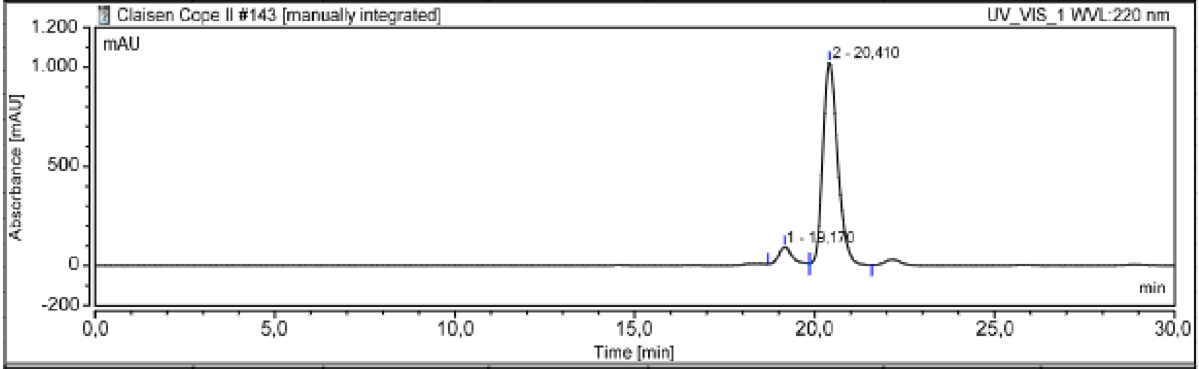

Integration Results

No.	Peak Name	Retention Time<br>min	Area<br>mAU*min	Height<br>mAU	Relative Area<br>%	Relative Height<br>%
1		19,170	37,627	87,771	7,20	7,93
2		20,410	484,759	1019,218	92,80	92,07
**Total:**			**522,386**	**1106,989**	**100,00**	**100,00**

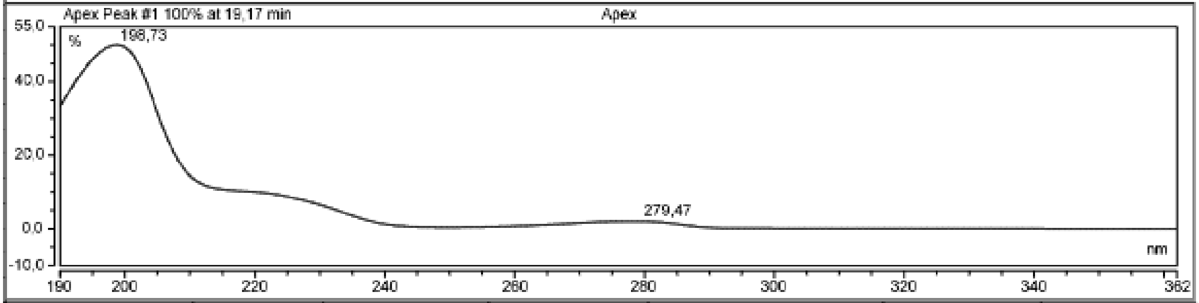

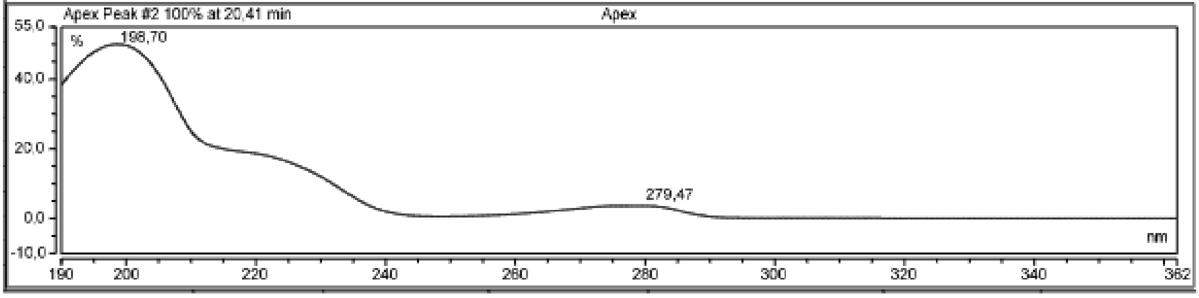

***(R,E)*-1-Isopropyl-4-methyl-2-(pent-3-en-2-yloxy)benzene (1i)**

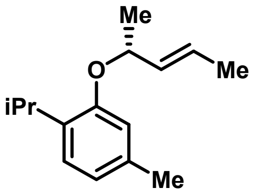

The title compound was synthesized from commercially available 2-isopropyl-5-methylphenol (153 mg, 1.02 mmol) following **general procedure A**. The crude material was purified by column chromatography (petroleum ether/ethyl acetate 40:1) to provide the desired product **1i** as colorless oil in quantitative yield (222 mg, 1.02 mmol).

$[\alpha]^{20}$= + 27.05 (c 1.25, CH_2Cl_2).

^1H NMR (400 MHz, $CDCl_3$) δ 7.12 (dd, *J* = 8.5, 2.6 Hz, 1H), 6.76 (dd, *J* = 7.7, 2.7 Hz, 1H), 6.72 (t, *J* = 2.3 Hz, 1H), 5.83 – 5.66 (m, 1H), 5.60 (dddt, *J* = 15.4, 6.0, 3.0, 1.5 Hz, 1H), 4.86 – 4.72 (m, 1H), 3.36 (qd, *J* = 6.8, 3.5 Hz, 1H), 2.33 (d, *J* = 4.0 Hz, 3H), 1.73 (ddq, *J* = 5.3, 2.8, 1.3 Hz, 3H), 1.47 – 1.39 (m, 3H), 1.24 (ddd, *J* = 7.1, 3.1, 1.6 Hz, 6H).

^{13}C NMR (101 MHz, $CDCl_3$) δ 155.2, 136.0, 135.0, 132.8, 126.6, 126.0, 121.2, 114.7, 74.5, 26.7, 23.0, 21.8, 21.5, 17.9.

90% *ee* (determined by chiral HPLC of derivative **1ia**).

^{1}H NMR (400 MHz, $CDCl_3$)

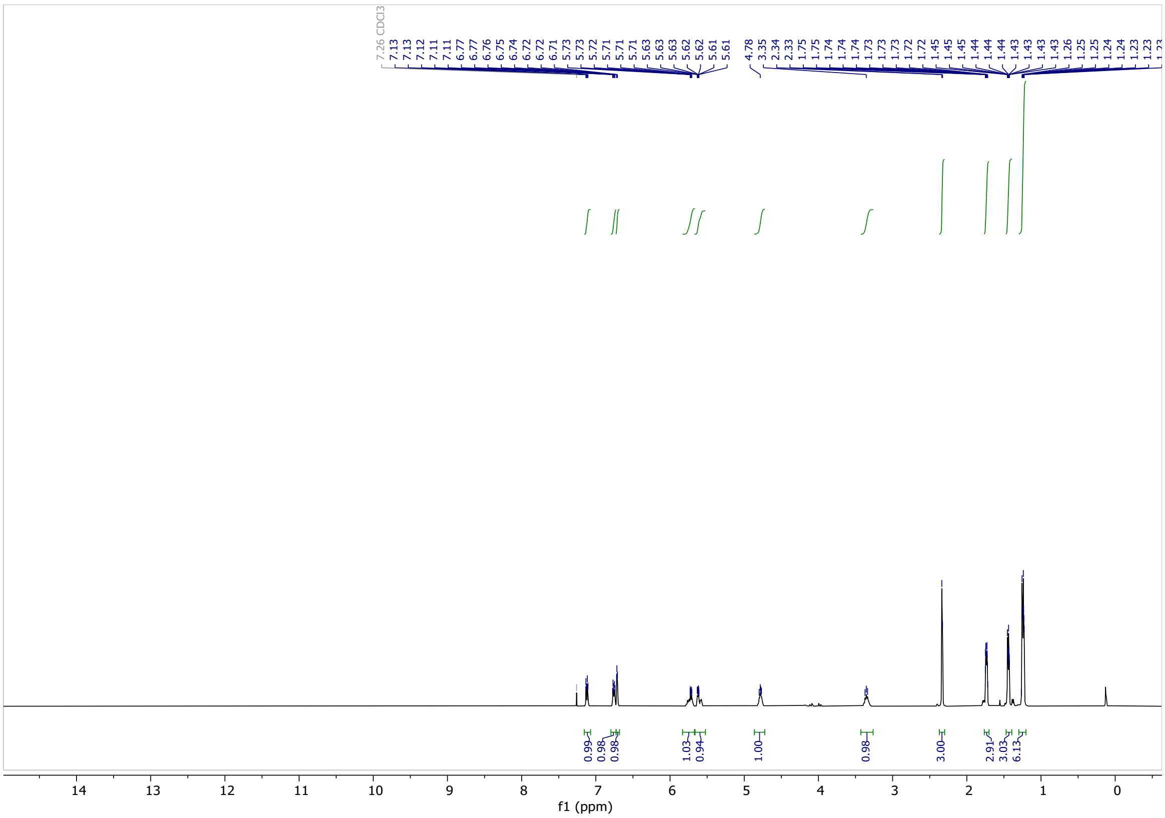

^{13}C NMR (101 MHz, $CDCl_3$)

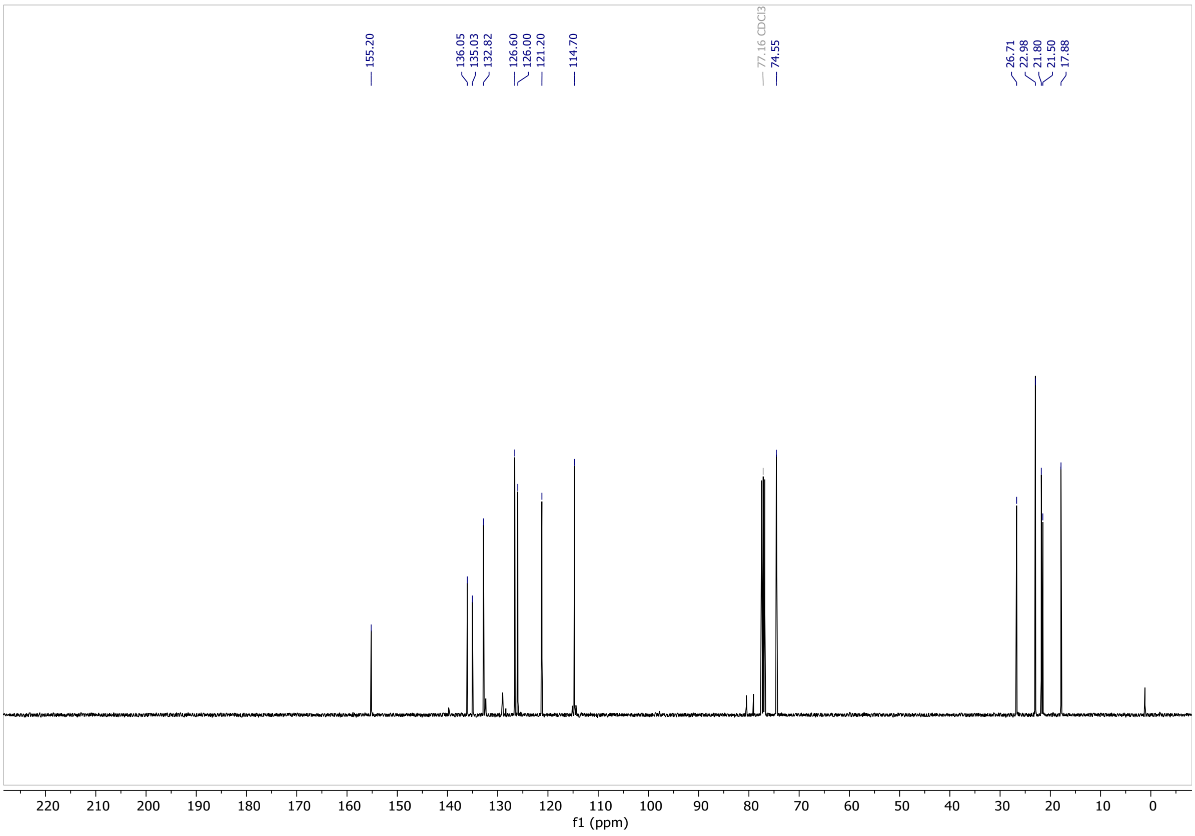

_(R)_-2-(2-Isopropyl-5-methylphenoxy)propan-1-ol (1ia)

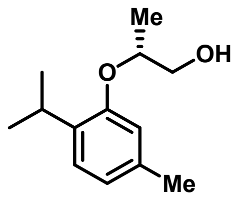

The title compound was synthesized from compound **1i** (33 mg, 0.15 mmol) following **general procedure C**. The desired product **1ia** was obtained in sufficient purity as colorless oil in 79% yield (25 mg, 0.12 mmol).

$[\alpha]^{20}$= - 29.71 (c 1.20, CH_2Cl_2).

^1H NMR (400 MHz, $CDCl_3$) δ 7.12 (d, *J* = 7.7 Hz, 1H), 6.80 – 6.75 (m, 1H), 6.74 (d, *J* = 1.6 Hz, 1H), 4.62 – 4.45 (m, 1H), 3.85 – 3.68 (m, 2H), 3.29 (hept, *J* = 6.9 Hz, 1H), 2.32 (s, 3H), 1.28 (d, *J* = 6.2 Hz, 4H), 1.21 (d, *J* = 7.0 Hz, 6H).

^{13}C NMR (101 MHz, $CDCl_3$) δ 154.5, 136.5, 135.2, 126.3, 121.9, 114.4, 74.8, 66.7, 26.6, 23.1, 23.1, 21.4, 16.1.

HRMS (ESI): exact mass calculated for $C_{13}H_{19}O_2^-$ [(M - H)$^-$], 207.1391; found 207.1380.

90% *ee* (determined by chiral HPLC: Chiralpak® IB column, n-Heptane/EtOH = 99.5:0.5, 0.7 mL/min, λ = 287.3 nm, 25 °C), major enantiomer. t_r = 16.65 min, minor enantiomer. t_r = 18.77 min.

^{1}H NMR (400 MHz, $CDCl_3$)

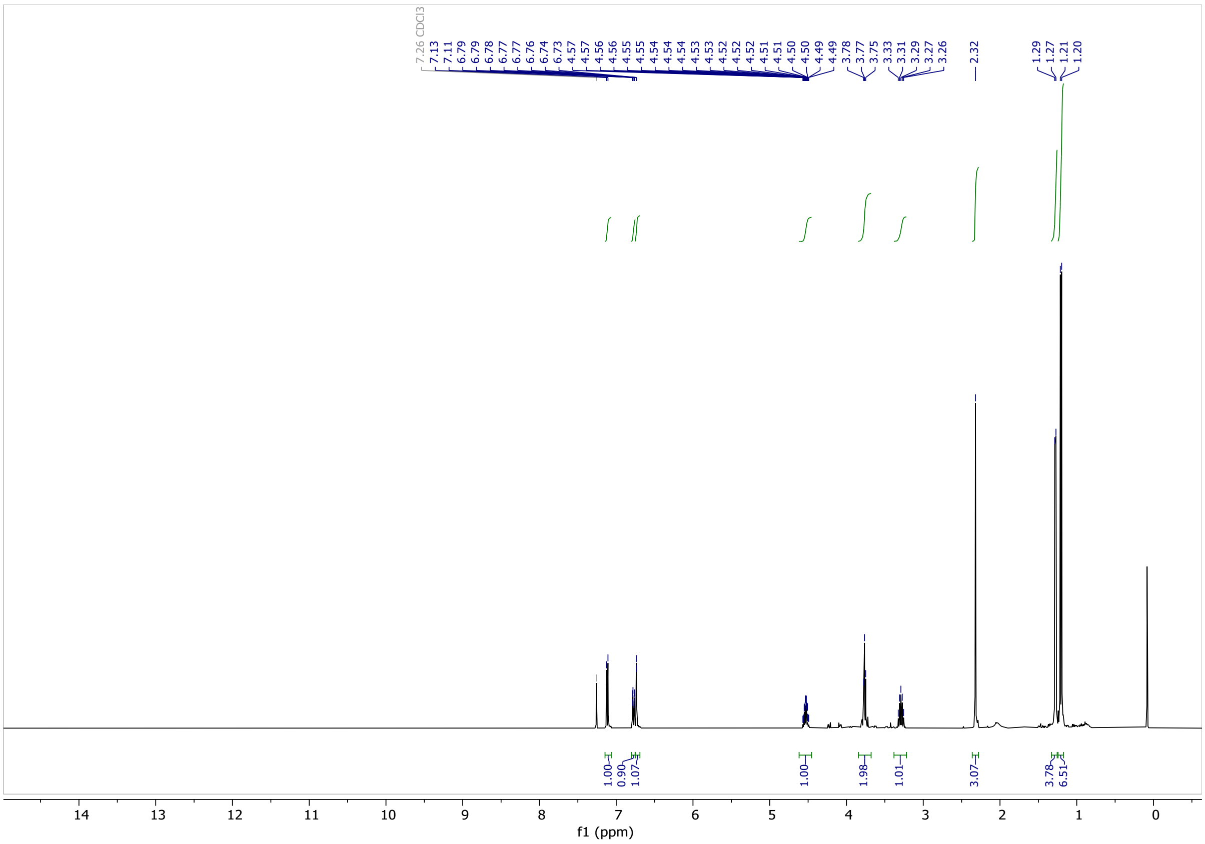

^{13}C NMR (101 MHz, $CDCl_3$)

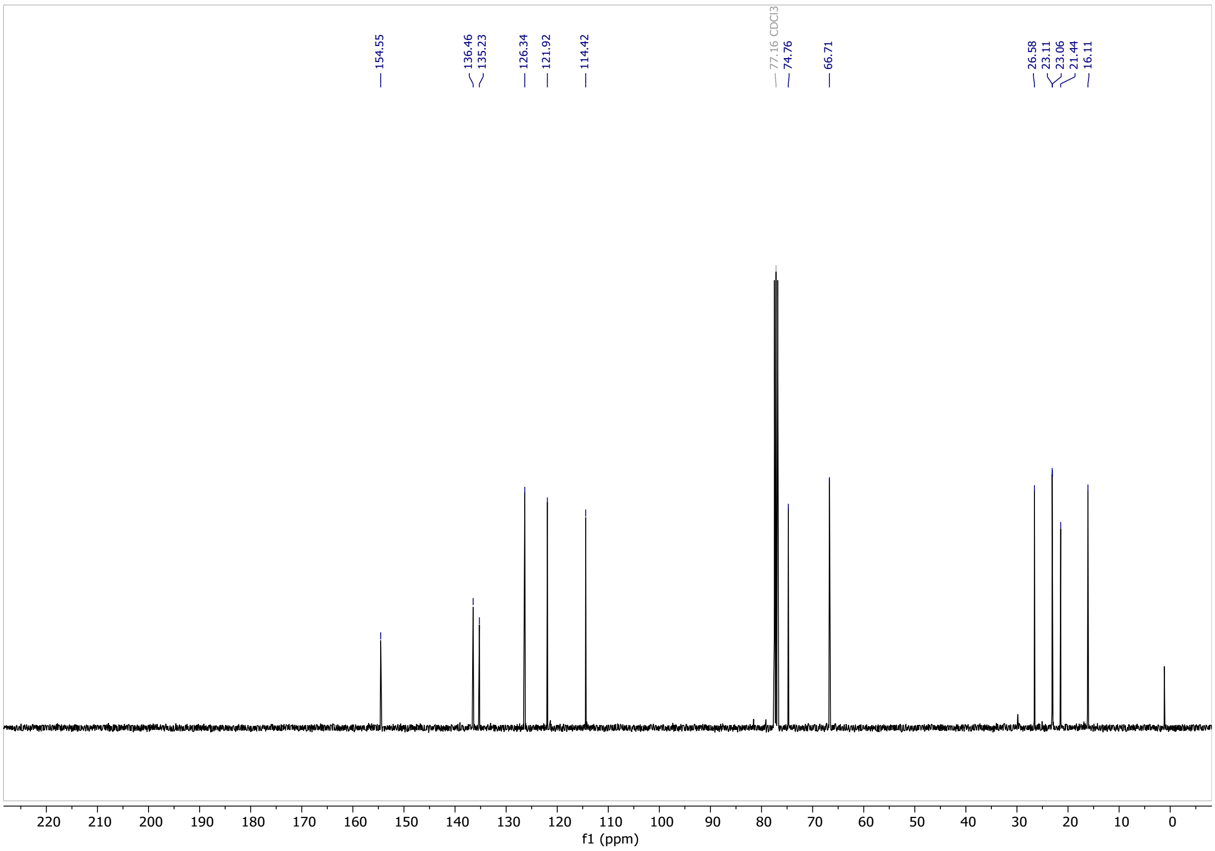

Chromatogram and Results

Instrument Method:	**Heptane_EtOH_99.5_0.5_0.7mlmin_25C_30min**	*B %:*	**0,0**
Column:	**IB**	*C %:*	**0,0**
Run Time (min):	**30,00**	*D %:*	**0,5**
Channel:	**UV_VIS_1**		
Wavelength:	**287,26**		

Chromatogram

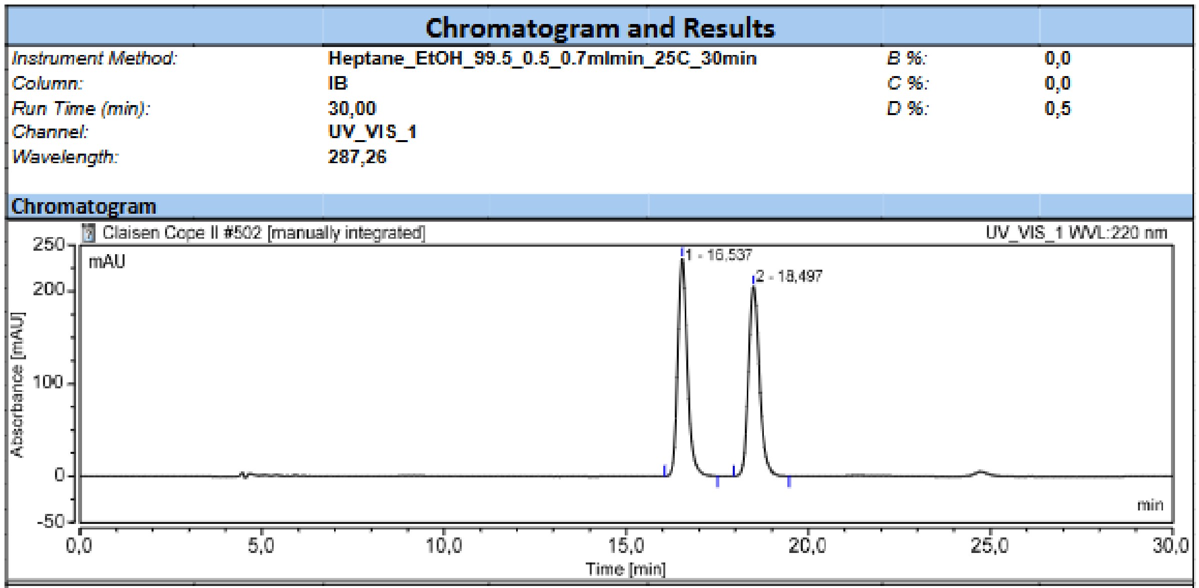

Integration Results

No.	Peak Name	Retention Time<br>min	Area<br>mAU*min	Height<br>mAU	Relative Area<br>%	Relative Height<br>%
1		16,537	72,274	235,296	50,32	53,42
2		18,497	71,348	205,209	49,68	46,58
**Total:**			**143,622**	**440,505**	**100,00**	**100,00**

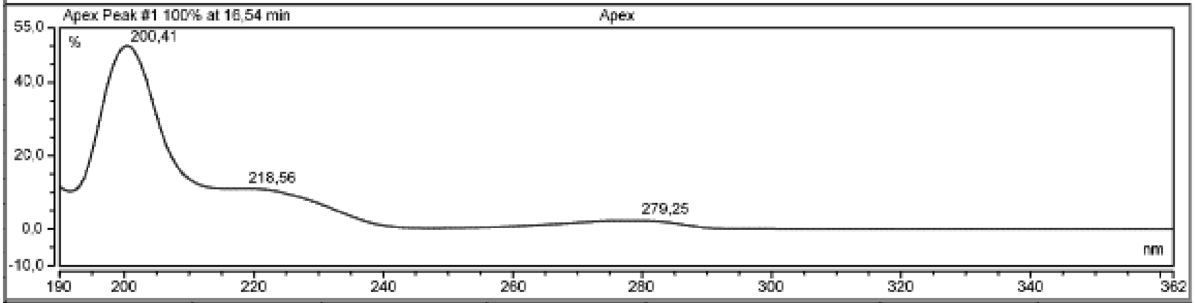

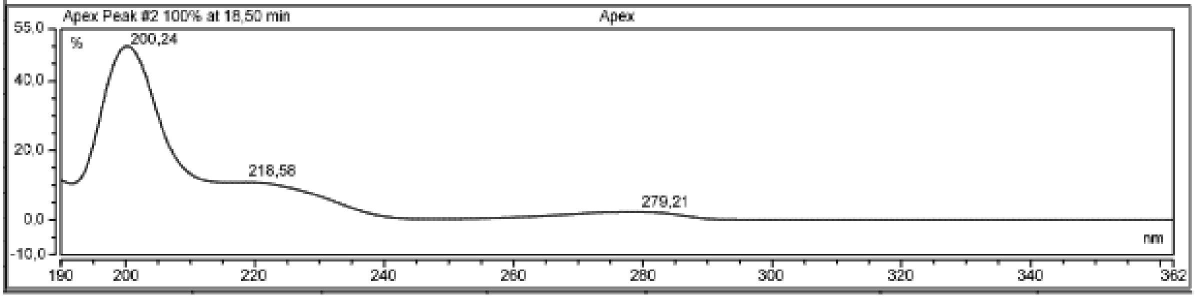

Chromatogram and Results

Instrument Method:	**Heptane_EtOH_99.5_0.5_0.7mlmin_25C_30min**	*B %:*	**0,0**
Column:	**IB**	*C %:*	**0,0**
Run Time (min):	**30,00**	*D %:*	**0,5**
Channel:	**UV_VIS_1**		
Wavelength:	**287,26**		

Chromatogram

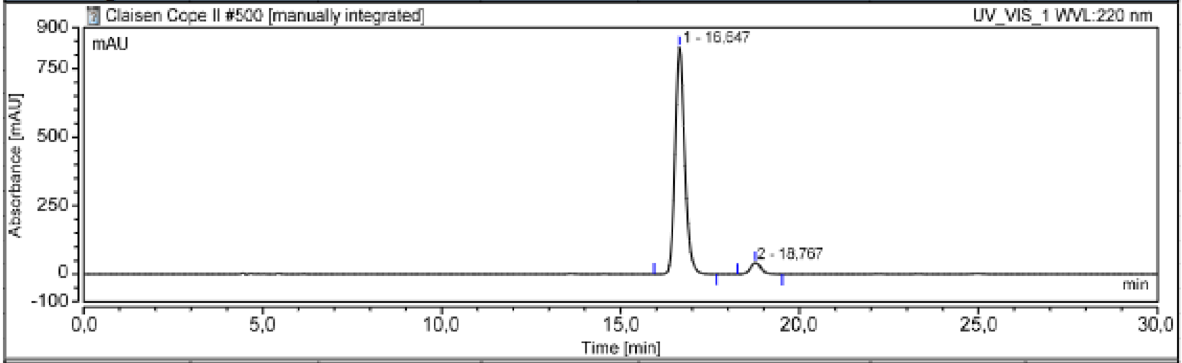

Integration Results

No.	Peak Name	Retention Time min	Area mAU*min	Height mAU	Relative Area %	Relative Height %
1		16,647	257,403	827,804	94,67	95,14
2		18,767	14,495	42,309	5,33	4,86
**Total:**			**271,898**	**870,112**	**100,00**	**100,00**

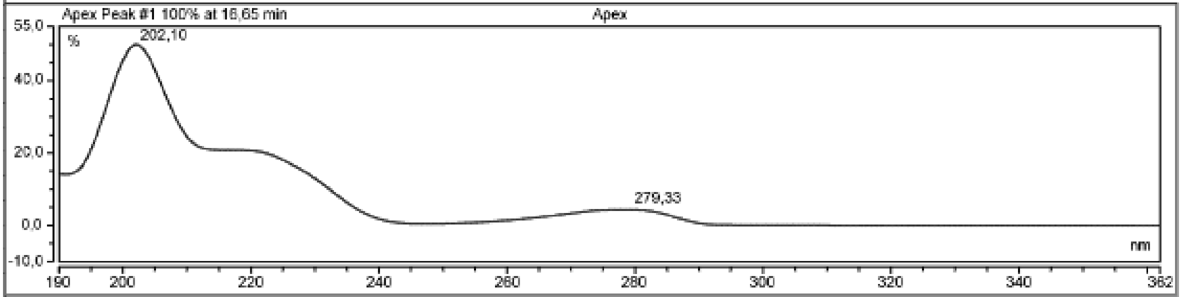

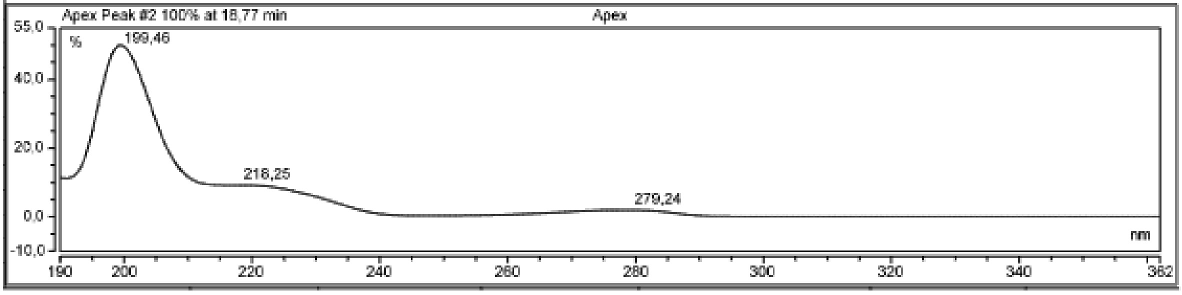

***(R,E)*-4-Methoxy-1-methyl-2-(pent-3-en-2-yloxy)benzene (1j)**

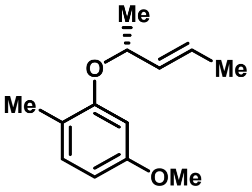

The title compound was synthesized from commercially available 5-methoxy-2-methylphenol (169 mg, 1.20 mmol) following **general procedure A**. The crude material was purified by column chromatography (petroleum ether/ethyl acetate 40:1) to provide the desired product **1j** as colorless oil in quantitative yield (247 mg, 1.20 mmol).

$[\alpha]^{20}$= + 39.63 (c 0.95, CH_2Cl_2).

^1H NMR (400 MHz, $CDCl_3$) δ 7.01 (dt, *J* = 8.2, 0.8 Hz, 1H), 6.46 (d, *J* = 2.5 Hz, 1H), 6.38 (dd, *J* = 8.2, 2.5 Hz, 1H), 5.80 – 5.61 (m, 1H), 5.61 – 5.48 (m, 1H), 4.77 – 4.63 (m, 1H), 3.76 (d, *J* = 0.6 Hz, 3H), 2.15 (d, *J* = 0.9 Hz, 3H), 1.69 (ddt, *J* = 6.4, 1.5, 0.8 Hz, 3H), 1.40 (dd, *J* = 6.3, 0.7 Hz, 3H).

^{13}C NMR (101 MHz, $CDCl_3$) δ 158.7, 157.1, 132.6, 130.6, 126.9, 120.1, 104.3, 101.5, 74.9, 55.5, 21.8, 17.9, 15.8.

86% *ee* (determined by chiral HPLC: Chiralpak® IB column, n-Heptane/iPrOH = 99.9:0.1, 0.7 mL/min, λ = 287.3 nm, 25 °C), minor enantiomer. t_r = 11.15 min, major enantiomer. t_r = 11.66 min.

^{1}H NMR (400 MHz, $CDCl_3$)

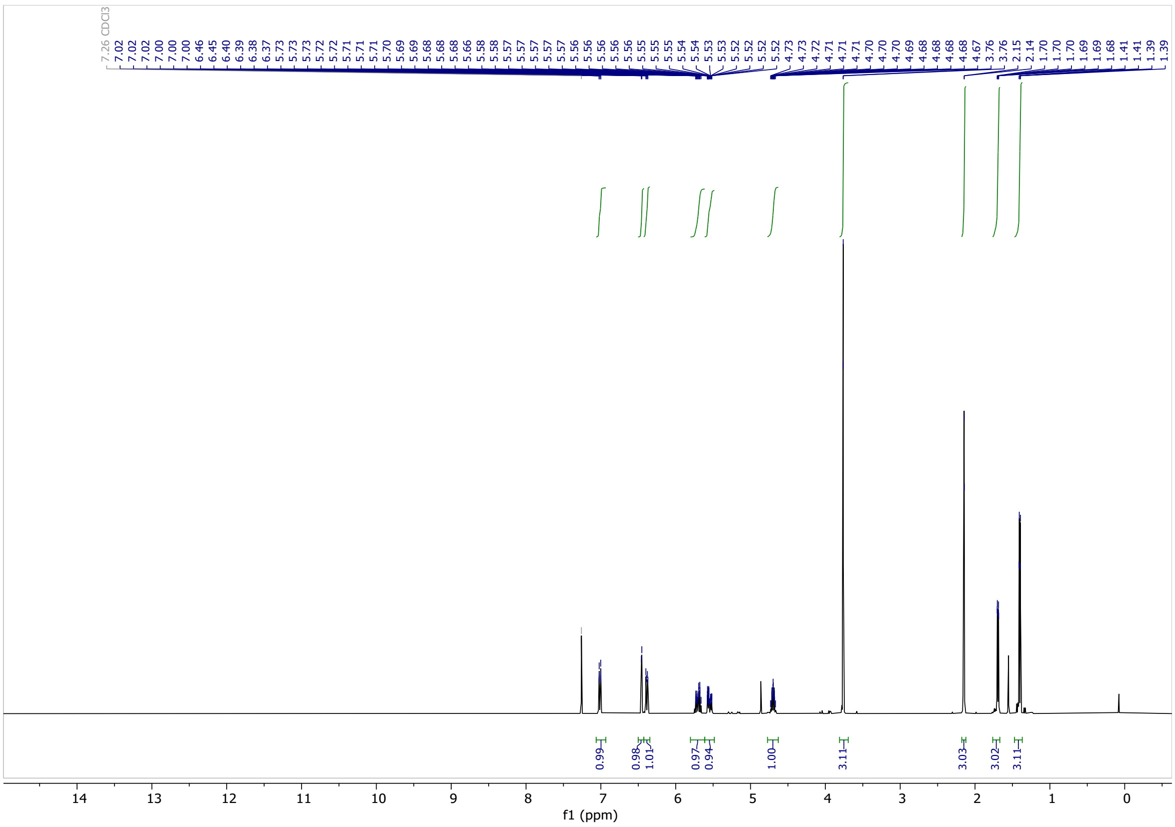

^{13}C NMR (101 MHz, $CDCl_3$)

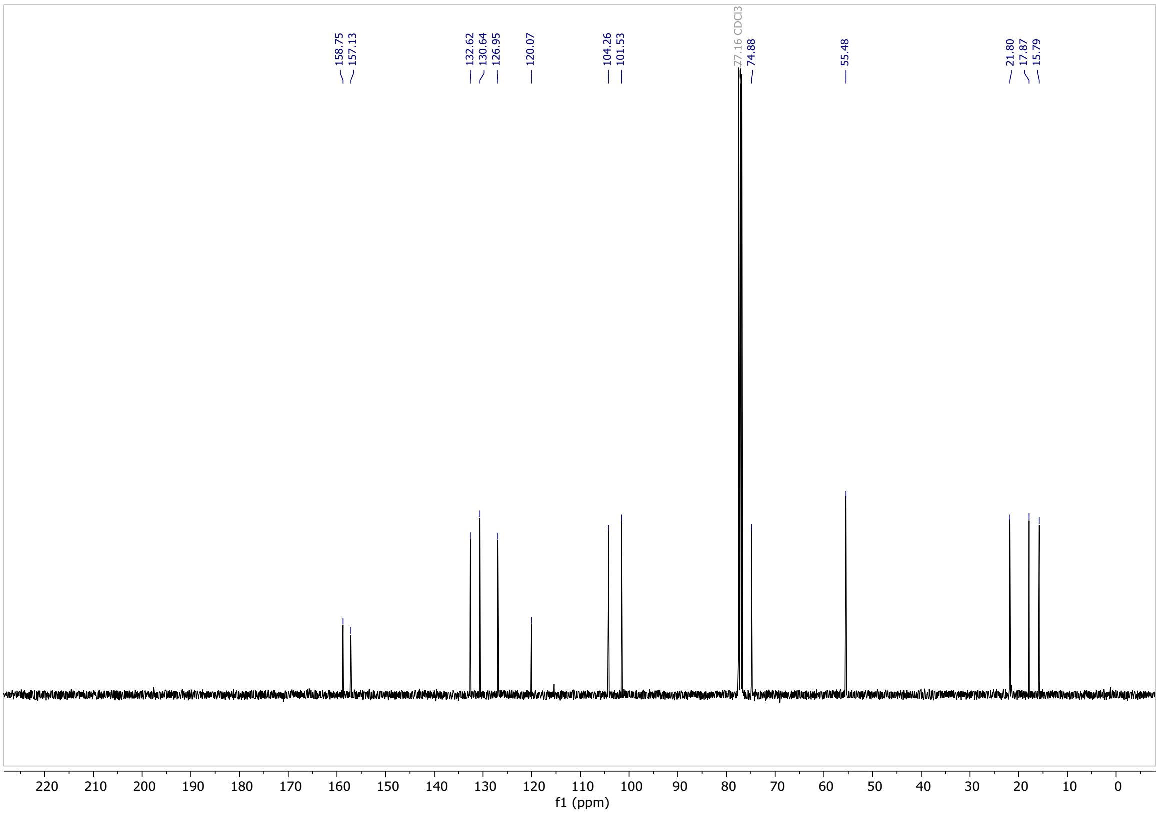

Chromatogram and Results

Instrument Method:	**Heptane_IPA_99.9_0.1_0.7mlmin_25C_45min-MK**	*B %:*	**0,1**
Column:	**IB**	*C %:*	**0,0**
Run Time (min):	**45,00**	*D %:*	**0,0**
Channel:	**UV_VIS_1**		
Wavelength:	**287,26**		

Chromatogram

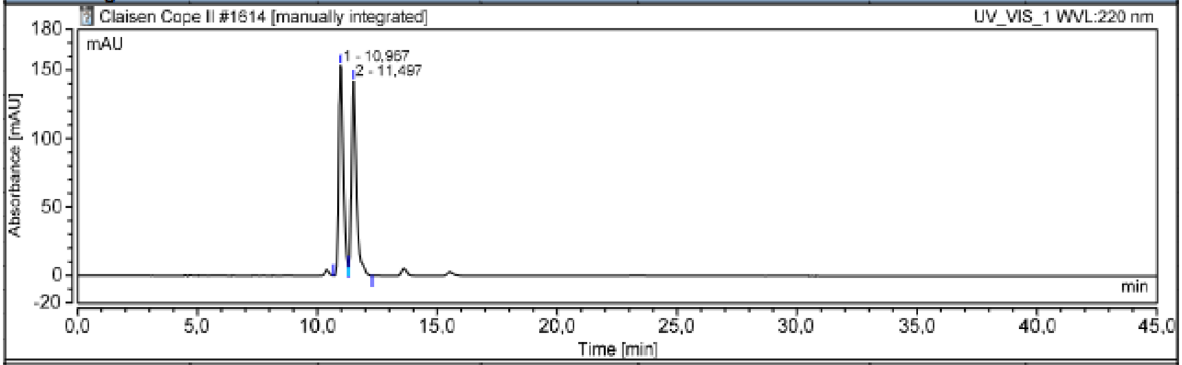

Integration Results

No.	Peak Name	Retention Time min	Area mAU*min	Height mAU	Relative Area %	Relative Height %
1		10,967	33,212	153,262	49,22	51,96
2		11,497	34,268	141,722	50,78	48,04
**Total:**			**67,480**	**294,984**	**100,00**	**100,00**

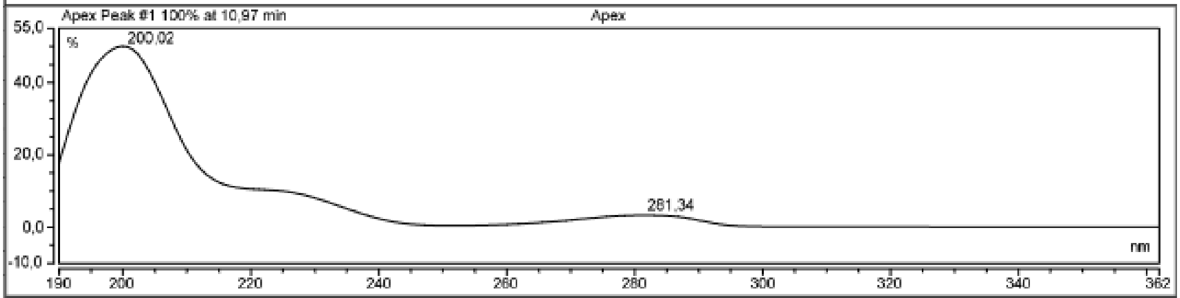

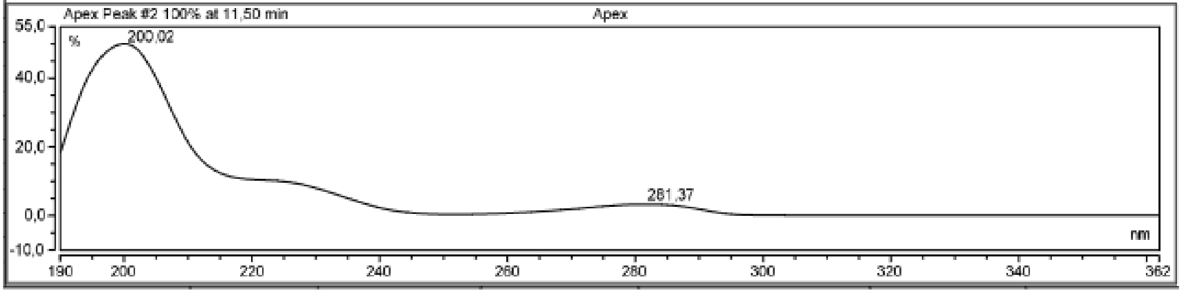

Chromatogram and Results

Instrument Method:	**Heptane_IPA_99.9_0.1_0.7mlmin_25C_45min-MK**	*B %:*	**0,1**
Column:	**IB**	*C %:*	**0,0**
Run Time (min):	**45,00**	*D %:*	**0,0**
Channel:	**UV_VIS_1**		
Wavelength:	**287,26**		

Chromatogram

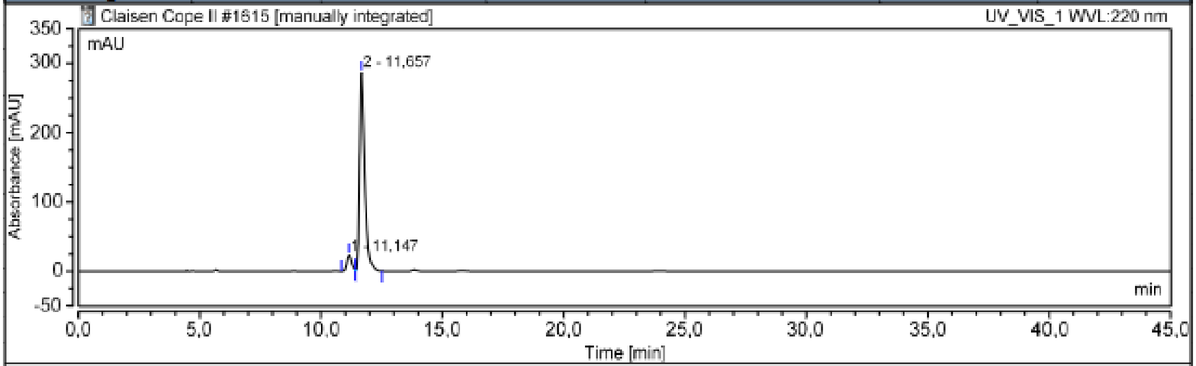

Integration Results

No.	Peak Name	Retention Time<br>min	Area<br>mAU*min	Height<br>mAU	Relative Area<br>%	Relative Height<br>%
1		11,147	5,216	23,572	6,87	7,59
2		11,657	70,722	286,993	93,13	92,41
**Total:**			**75,938**	**310,565**	**100,00**	**100,00**

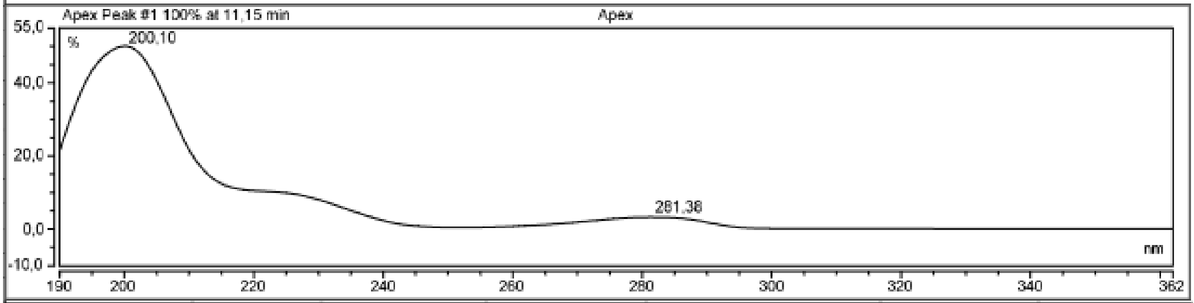

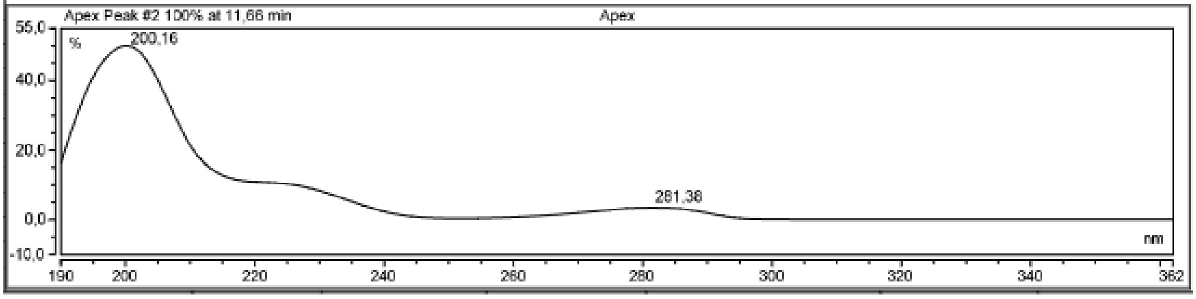

***(R,E)*-4-Fluoro-1-methyl-2-(pent-3-en-2-yloxy)benzene (1k)**

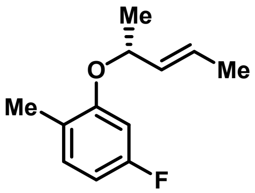

The title compound was synthesized from commercially available 5-fluoro-2-methylphenol (152 mg, 1.20 mmol) following **general procedure A**. The crude material was purified by column chromatography (petroleum ether/ethyl acetate 40:1) to provide the desired product **1k** as colorless oil in 91% yield (213 mg, 1.10 mmol).

$[\alpha]^{20}$= + 23.15 (c 1.30, CH_2Cl_2).

^{1}H NMR (400 MHz, $CDCl_3$) δ 7.03 (ddd, *J* = 8.0, 6.9, 0.9 Hz, 1H), 6.61 – 6.48 (m, 2H), 5.71 (dqd, *J* = 15.5, 6.4, 1.0 Hz, 1H), 5.52 (ddq, *J* = 15.5, 6.5, 1.6 Hz, 1H), 4.69 (h, *J* = 6.7 Hz, 1H), 2.16 (t, *J* = 1.0 Hz, 3H), 1.70 (ddd, *J* = 6.5, 1.6, 0.8 Hz, 3H), 1.41 (d, *J* = 6.4 Hz, 3H).

^{13}C NMR (101 MHz, $CDCl_3$) δ 161.8 (d, *J* = 241.6 Hz), 157.1 (d, *J* = 9.8 Hz), 132.1, 130.7 (d, *J* = 9.7 Hz), 127.4, 123.1 (d, *J* = 3.2 Hz), 106.3 (d, *J* = 20.9 Hz), 101.5 (d, *J* = 25.1 Hz), 75.1, 21.8, 17.9, 16.0.

^{19}F NMR (377 MHz, $CDCl_3$) δ -115.67 (d, *J* = 2.7 Hz).

90% *ee* (determined by chiral HPLC: Chiralcel® OJ3 column, n-Hexane/iPrOH = 99.9:0.1, 0.5 mL/min, λ = 287.3 nm, 25 °C), minor enantiomer. t_r = 8.05 min, major enantiomer. t_r = 8.29 min.

^{1}H NMR (400 MHz, $CDCl_3$)

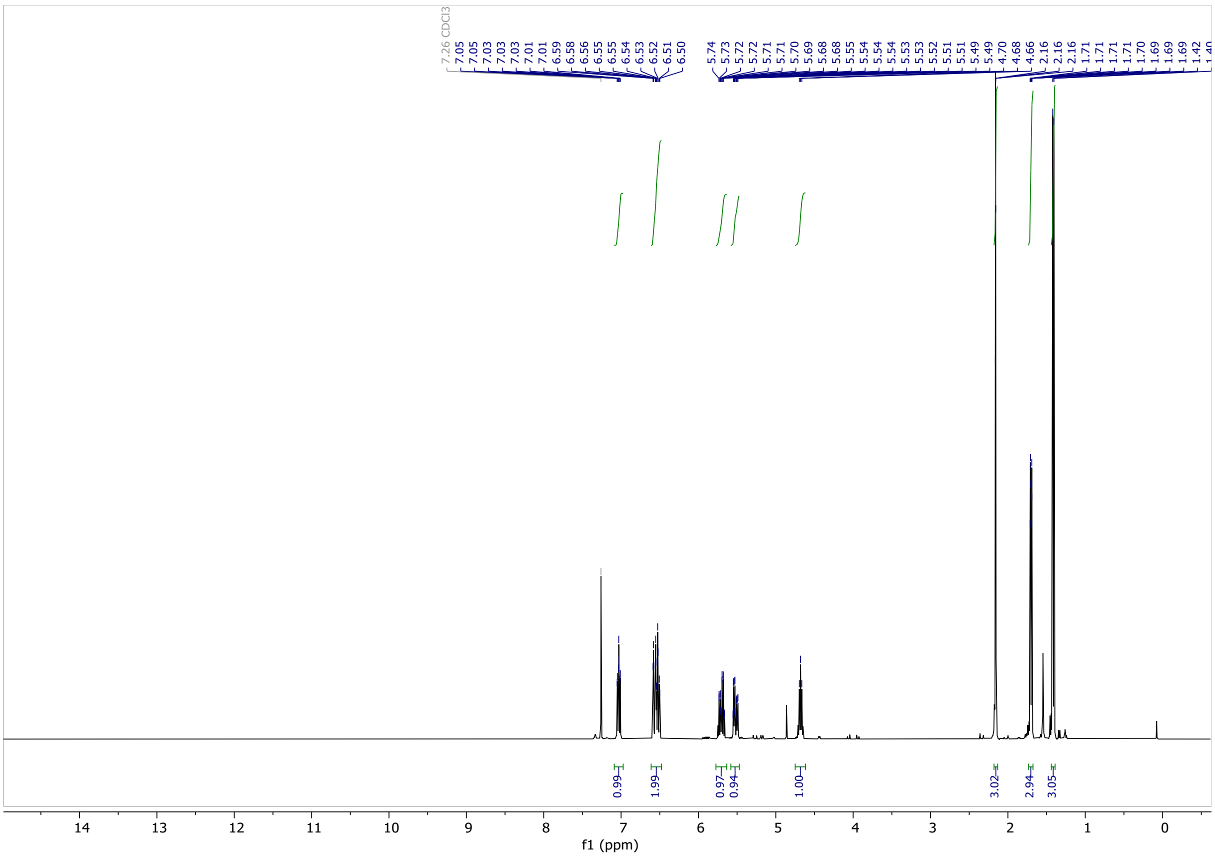

^{13}C NMR (101 MHz, $CDCl_3$)

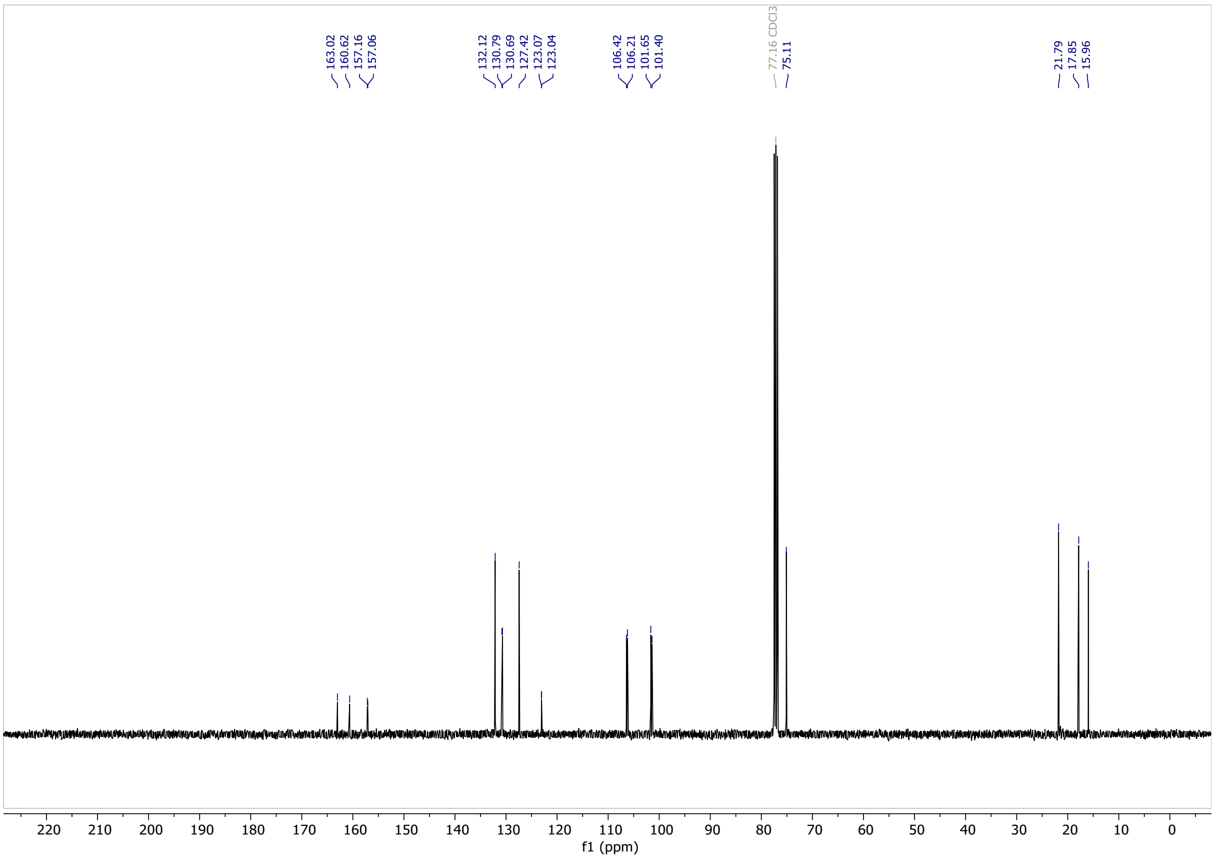

^{19}F NMR (377 MHz, $CDCl_3$)

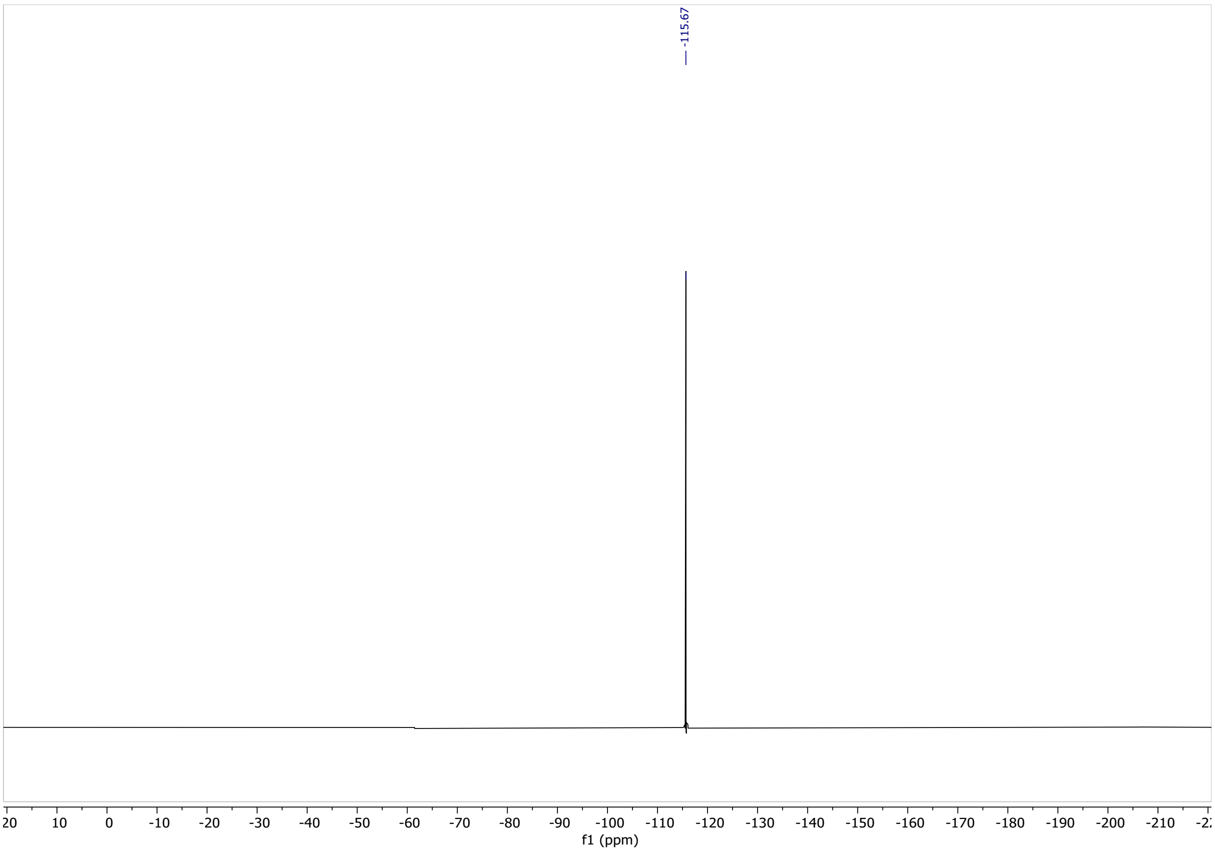

Chromatogram and Results

Instrument Method:	**Hexane_IPA_99.9_0.1 _0.5mLmin_25C_20min-MK**	*B %:*	**0,1**
Column:	**OJ3**	*C %:*	**99,9**
Run Time (min):	**20,00**	*D %:*	**0,0**
Channel:	**UV_VIS_1**		
Wavelength:	**287,26**		

Chromatogram

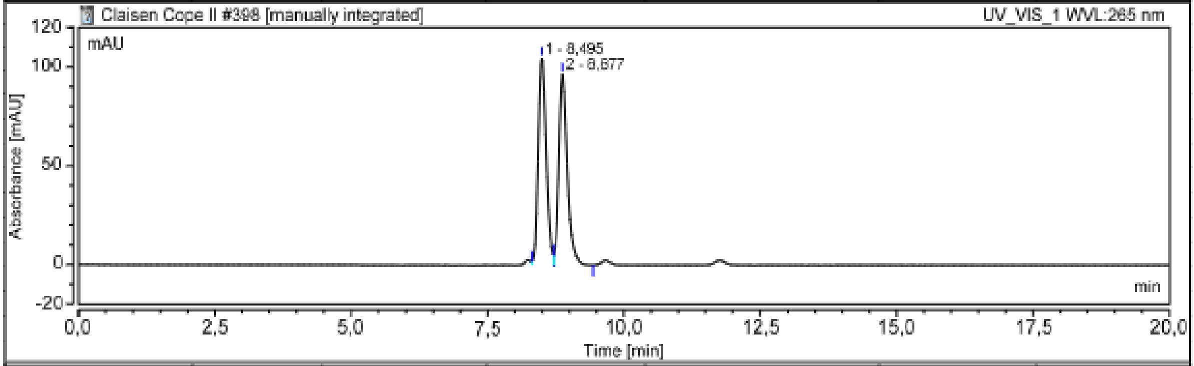

Integration Results

No.	Peak Name	Retention Time<br>min	Area<br>mAU*min	Height<br>mAU	Relative Area<br>%	Relative Height<br>%
1		8,495	17,033	104,846	49,60	52,04
2		8,877	17,305	96,614	50,40	47,96
**Total:**			**34,339**	**201,460**	**100,00**	**100,00**

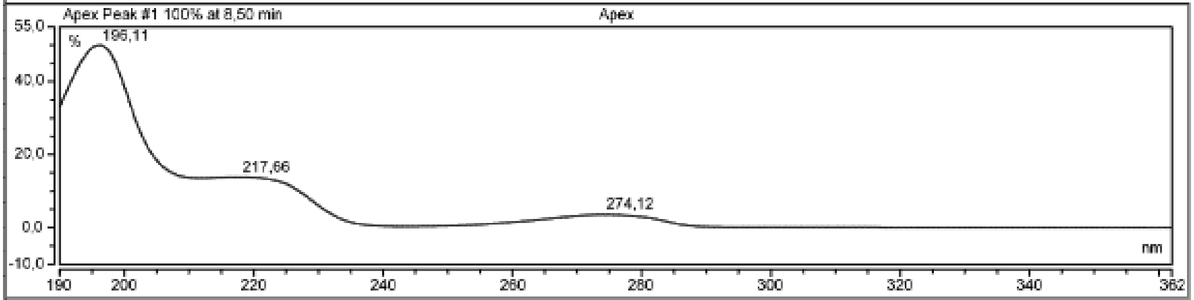

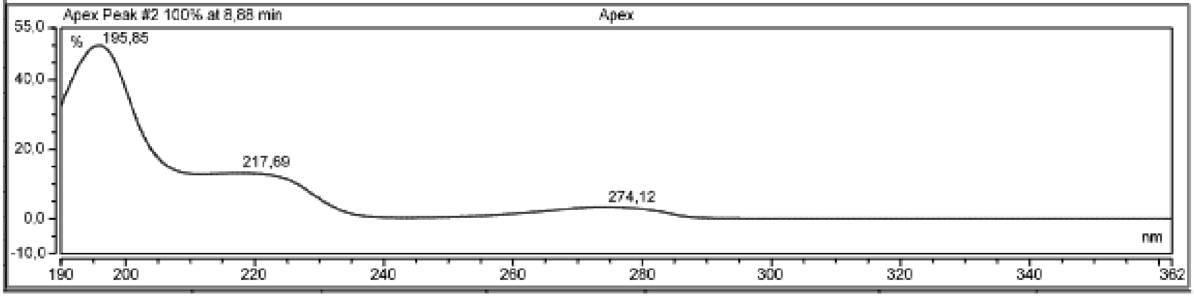

Chromatogram and Results

Instrument Method:	**Hexane_IPA_99.9_0.1 _0.5mLmin_25C_20min-MK**	*B %:*	**0,1**
Column:	**OJ3**	*C %:*	**99,9**
Run Time (min):	**20,00**	*D %:*	**0,0**
Channel:	**UV_VIS_1**		
Wavelength:	**287,26**		

Chromatogram

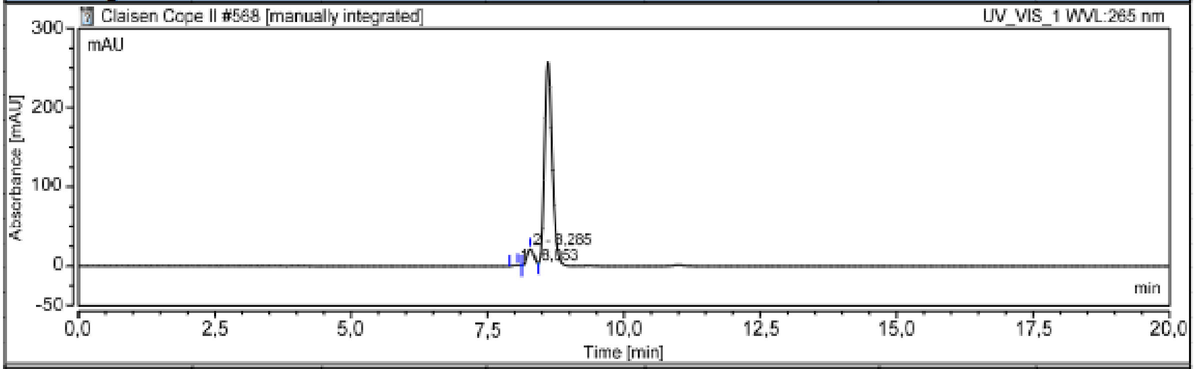

Integration Results

No.	Peak Name	Retention Time min	Area mAU*min	Height mAU	Relative Area %	Relative Height %
1		8,053	0,176	1,372	5,18	6,00
2		8,285	3,214	21,494	94,82	94,00
**Total:**			**3,390**	**22,866**	**100,00**	**100,00**

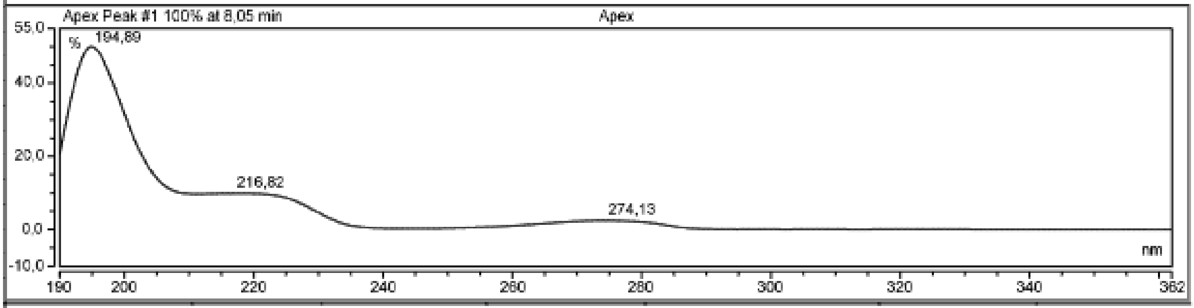

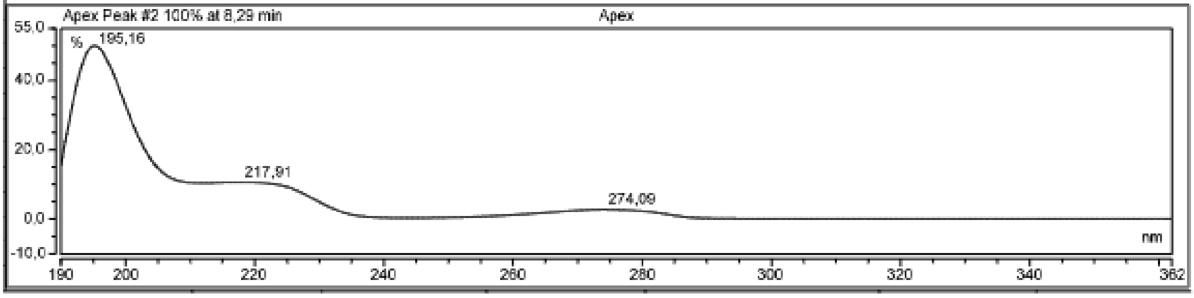

***(R,E)*-4-Chloro-1-methyl-2-(pent-3-en-2-yloxy)benzene (1l)**

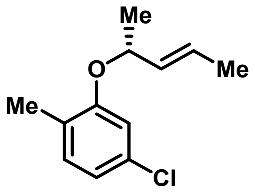

The title compound was synthesized from commercially available 5-chloro-2-methylphenol (155 mg, 1.05 mmol) following **general procedure A**. The crude material was purified by column chromatography (petroleum ether/ethyl acetate 40:1) to provide the desired product **1l** as colorless oil in quantitative yield (222 mg, 1.05 mmol).

$[\alpha]^{20}$= + 51.35 (c 1.10, CH_2Cl_2).

^{1}H NMR (400 MHz, $CDCl_3$) δ 7.09 – 6.89 (m, 1H), 6.80 (d, *J* = 6.9 Hz, 2H), 5.79 – 5.62 (m, 1H), 5.62 – 5.44 (m, 1H), 4.71 (h, *J* = 6.6 Hz, 1H), 2.17 (s, 3H), 1.70 (ddd, *J* = 6.4, 1.6, 0.8 Hz, 3H), 1.41 (dd, *J* = 6.3, 0.7 Hz, 3H).

^{13}C NMR (101 MHz, $CDCl_3$) δ 156.9, 132.0, 131.6, 131.3, 127.4, 126.3, 120.2, 114.1, 75.2, 21.7, 17.9, 16.2.

85% *ee* (determined by chiral HPLC: Chiralcel® OJ3 column, n-Heptane/EtOH = 99.9:0.1, 0.5 mL/min, λ = 287.3 nm, 25 °C), minor enantiomer. t_r = 8.15 min, major enantiomer. t_r = 8.89 min.

^{1}H NMR (400 MHz, $CDCl_3$)

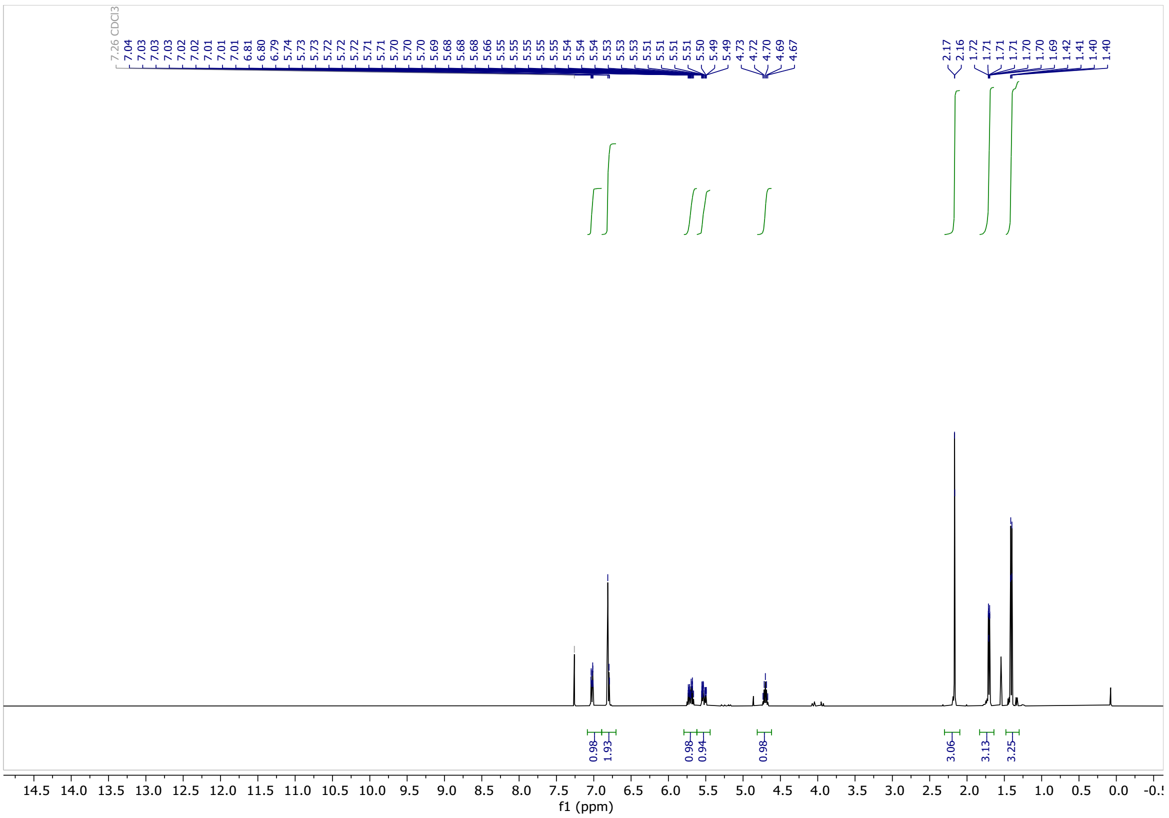

^{13}C NMR (101 MHz, $CDCl_3$)

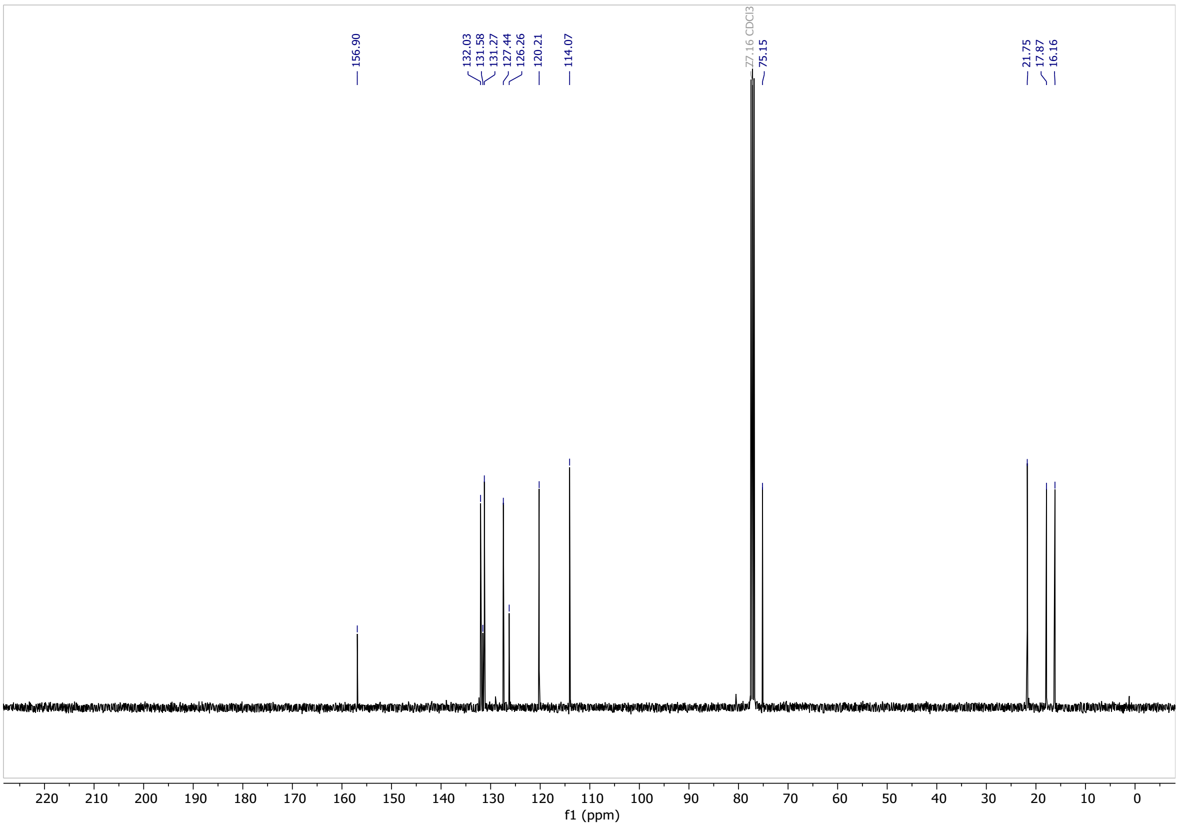

Chromatogram and Results

Instrument Method:	**Heptane_EtOH_99.9_0.1_0.5mlmin_25C_20min**	*B %:*	**0,0**
Column:	**OJ-3**	*C %:*	**0,0**
Run Time (min):	**20,00**	*D %:*	**0,1**
Channel:	**UV_VIS_1**		
Wavelength:	**287,26**		

Chromatogram

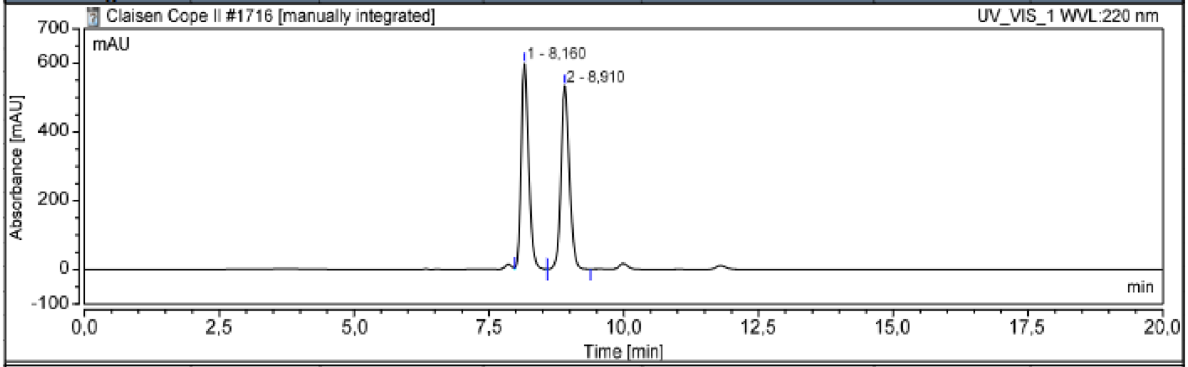

Integration Results

No.	Peak Name	Retention Time min	Area mAU*min	Height mAU	Relative Area %	Relative Height %
1		8,160	95,635	598,469	49,76	52,84
2		8,910	96,542	534,105	50,24	47,16
**Total:**			**192,177**	**1132,574**	**100,00**	**100,00**

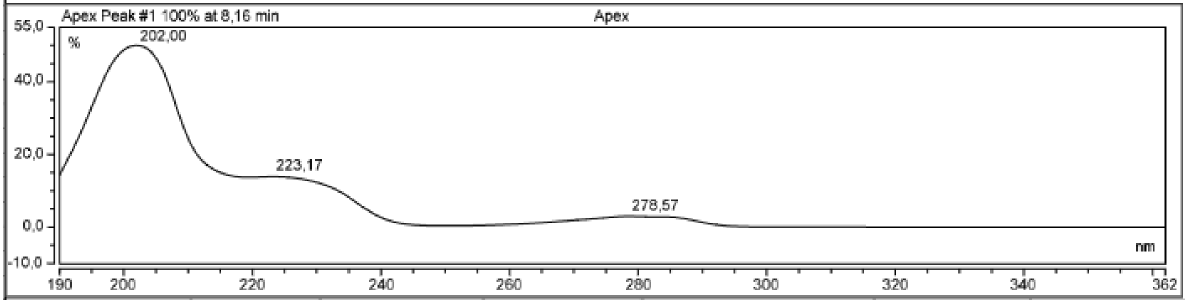

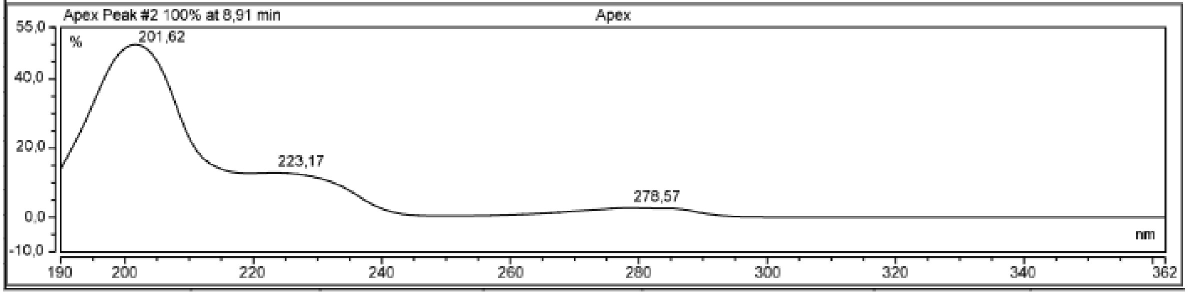

Chromatogram and Results

Instrument Method:	**Heptane_EtOH_99.9_0.1_0.5mlmin_25C_20min**	*B %:*	**0,0**
Column:	**OJ-3**	*C %:*	**0,0**
Run Time (min):	**20,00**	*D %:*	**0,1**
Channel:	**UV_VIS_1**		
Wavelength:	**287,26**		

Chromatogram

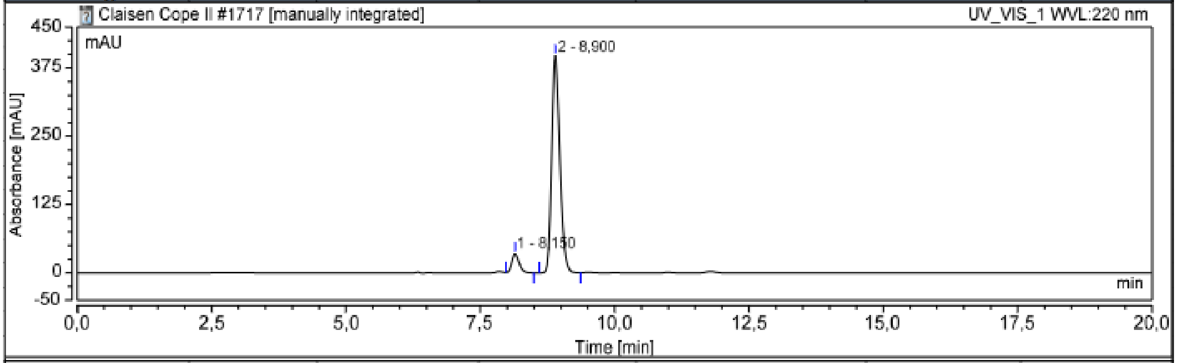

Integration Results

No.	Peak Name	Retention Time min	Area mAU*min	Height mAU	Relative Area %	Relative Height %
1		8,150	5,559	35,157	7,38	8,13
2		8,900	69,758	397,514	92,62	91,87
**Total:**			**75,317**	**432,671**	**100,00**	**100,00**

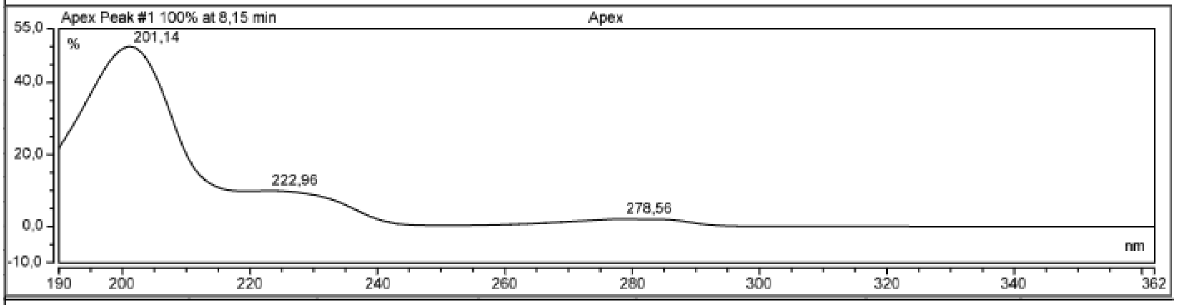

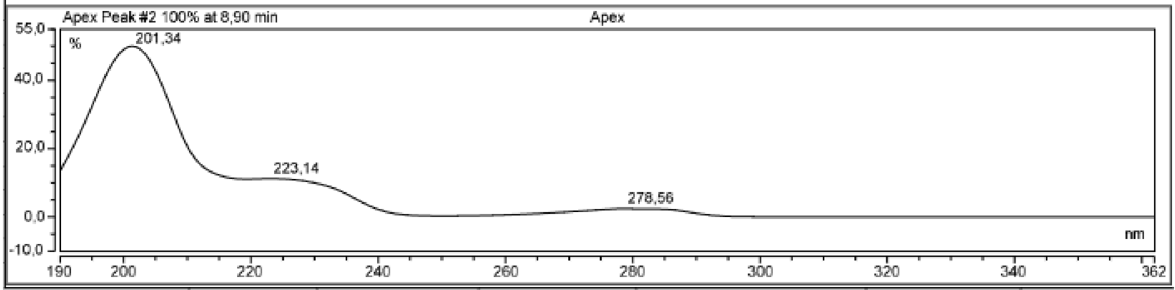

(R,E)-4-Bromo-1-methyl-2-(pent-3-en-2-yloxy)benzene (1m)

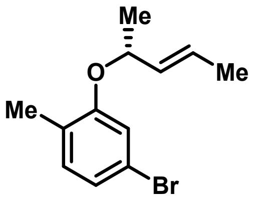

The title compound was synthesized from commercially available 5-bromo-2-methylphenol (151 mg, 0.798 mmol) following **general procedure A**. The crude material was purified by column chromatography (petroleum ether/ethyl acetate 40:1) to provide the desired product **1m** as colorless oil in 91% yield (186 mg, 0.73 mmol).

$[\alpha]^{20}$= + 58.48 (c 1.00, CH_2Cl_2).

^{1}H NMR (400 MHz, $CDCl_3$) δ 7.02 – 6.93 (m, 3H), 5.80 – 5.61 (m, 1H), 5.61 – 5.44 (m, 1H), 4.71 (p, *J* = 6.4 Hz, 1H), 2.16 (d, *J* = 0.9 Hz, 3H), 1.71 (ddt, *J* = 6.5, 1.8, 0.9 Hz, 3H), 1.41 (dd, *J* = 6.3, 0.9 Hz, 3H).

^{13}C NMR (101 MHz, $CDCl_3$) δ 157.1, 132.0, 131.7, 127.5, 126.8, 123.2, 119.3, 116.9, 75.2, 21.7, 17.9, 16.2.

84% *ee* (determined by chiral HPLC: Chiralcel® OJ3 column, n-Heptane/EtOH = 99.9:0.1, 0.3 mL/min, λ = 287.3 nm, 25 °C), minor enantiomer. t_r = 15.09 min, major enantiomer. t_r = 17.35 min.

^{1}H NMR (400 MHz, $CDCl_3$)

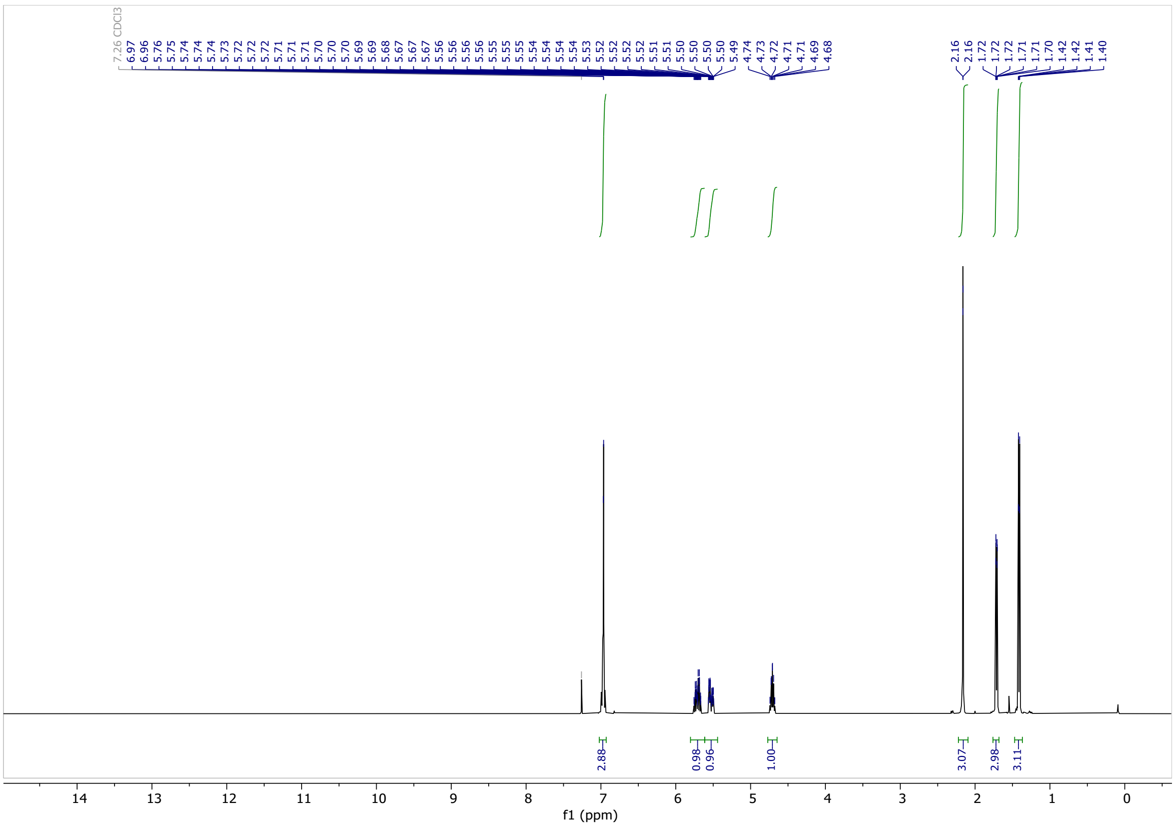

^{13}C NMR (101 MHz, $CDCl_3$)

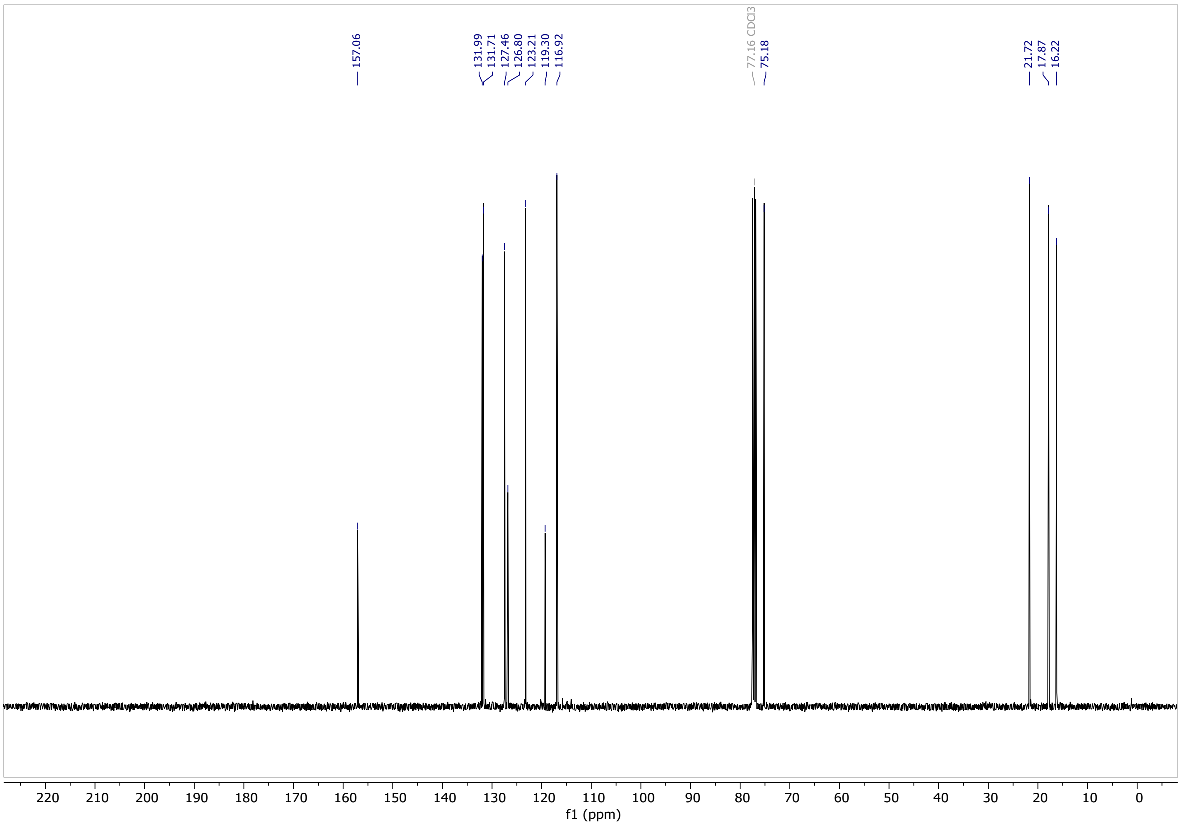

Chromatogram and Results

Instrument Method:	**Heptane_EtOH_99.9_0.1_0.3mlmin_25C_30min**	*B %:*	**0,0**
Column:	**OJ3**	*C %:*	**0,0**
Run Time (min):	**30,00**	*D %:*	**0,1**
Channel:	**UV_VIS_1**		
Wavelength:	**287,26**		

Chromatogram

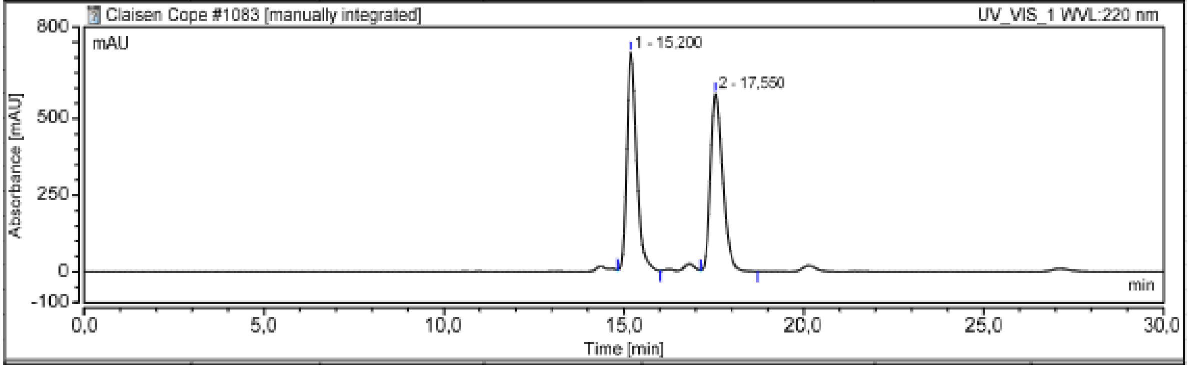

Integration Results

No.	Peak Name	Retention Time<br>min	Area<br>mAU*min	Height<br>mAU	Relative Area<br>%	Relative Height<br>%
1		15,200	233,935	715,680	50,63	55,11
2		17,550	228,077	582,875	49,37	44,89
**Total:**			**462,012**	**1298,555**	**100,00**	**100,00**

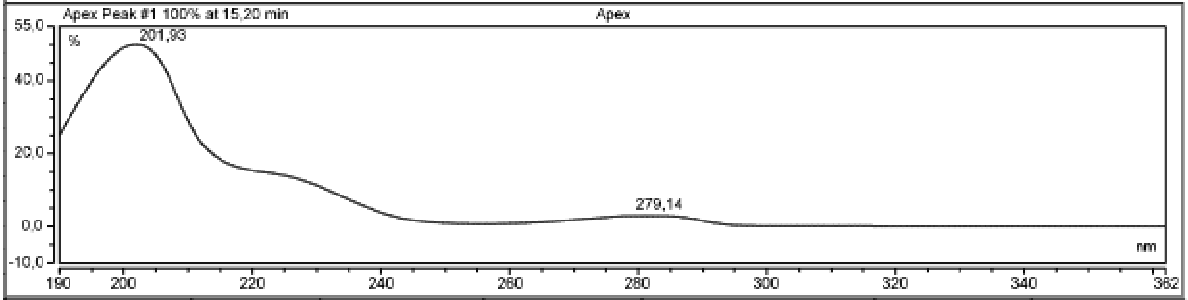

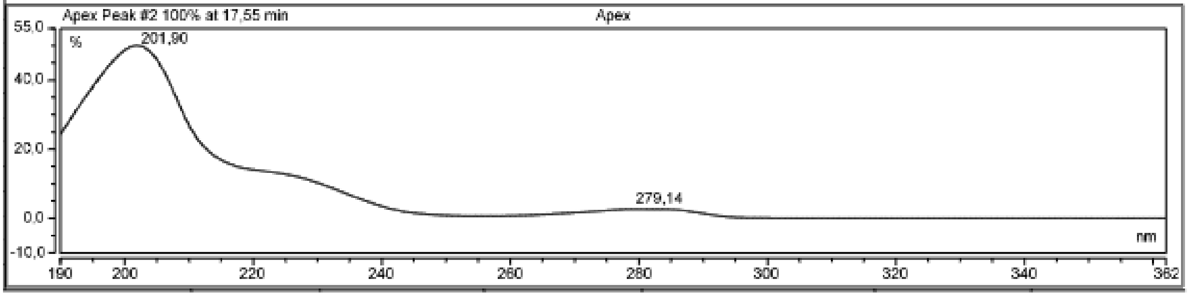

Chromatogram and Results

Instrument Method:	**Heptane_EtOH_99.9_0.1_0.3mlmin_25C_30min**	*B %:*	**0,0**
Column:	**OJ3**	*C %:*	**0,0**
Run Time (min):	**30,00**	*D %:*	**0,1**
Channel:	**UV_VIS_1**		
Wavelength:	**287,26**		

Chromatogram

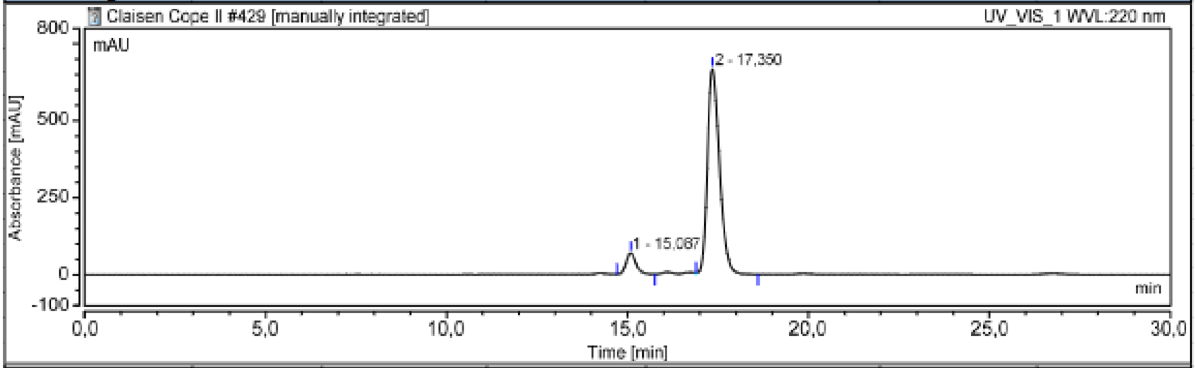

Integration Results

No.	Peak Name	Retention Time<br>min	Area<br>mAU*min	Height<br>mAU	Relative Area<br>%	Relative Height<br>%
1		15,087	22,805	70,950	8,09	9,58
2		17,350	258,973	669,363	91,91	90,42
**Total:**			**281,778**	**740,313**	**100,00**	**100,00**

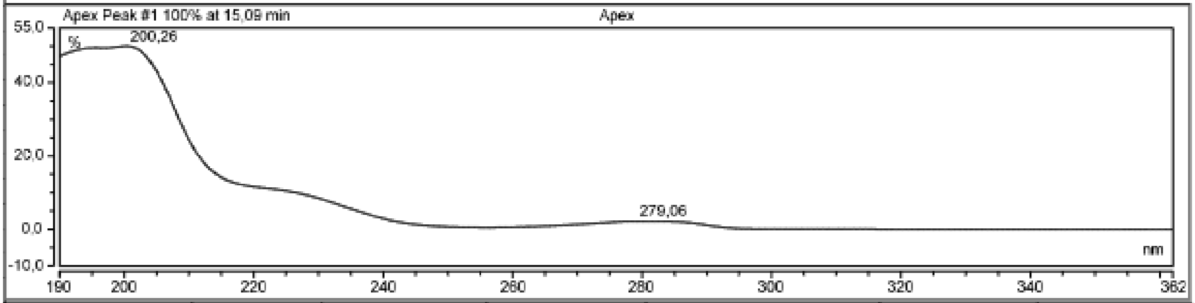

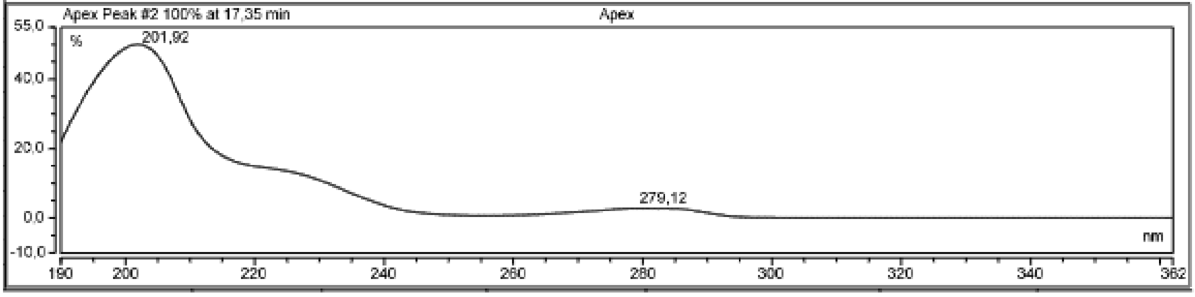

***(R,E)*-1-Methoxy-4-methyl-2-(pent-3-en-2-yloxy)benzene (1n)**

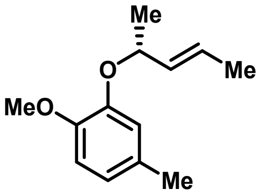

The title compound was synthesized from commercially available 2-methoxy-5-methylphenol (150 mg, 1.06 mmol) following **general procedure A**. The crude material was purified by column chromatography (petroleum ether/ethyl acetate 40:1) to provide the desired product **1n** as colorless oil in 64% yield (140 mg, 0.68 mmol).

$[\alpha]^{20}$= + 36.70 (c 0.30, CH_2Cl_2).

^1H NMR (400 MHz, $CDCl_3$) δ 6.83 – 6.64 (m, 3H), 5.77 – 5.51 (m, 2H), 4.73 (p, *J* = 6.4 Hz, 1H), 3.83 (s, 3H), 2.27 (s, 3H), 1.73 – 1.62 (m, 3H), 1.44 (dd, *J* = 6.3, 1.0 Hz, 3H).

^{13}C NMR (101 MHz, $CDCl_3$) δ 148.3, 147.3, 132.5, 130.3, 127.3, 121.5, 117.7, 112.1, 75.9, 56.2, 21.5, 21.0, 17.8.

HRMS (ESI): exact mass calculated for $C_{13}H_{18}NaO_2^+$ [$(M + Na)^+$], 229.1199; found 229.1200.

87% *ee* (determined by chiral HPLC: Chiralcel® IA-3 column, n-Heptane/EtOH = 99.9:0.1, 0.5 mL/min, λ = 287.3 nm, 25 °C), minor enantiomer. t_r = 13.17 min, major enantiomer. t_r = 14.18 min.

^{1}H NMR (400 MHz, $CDCl_3$)

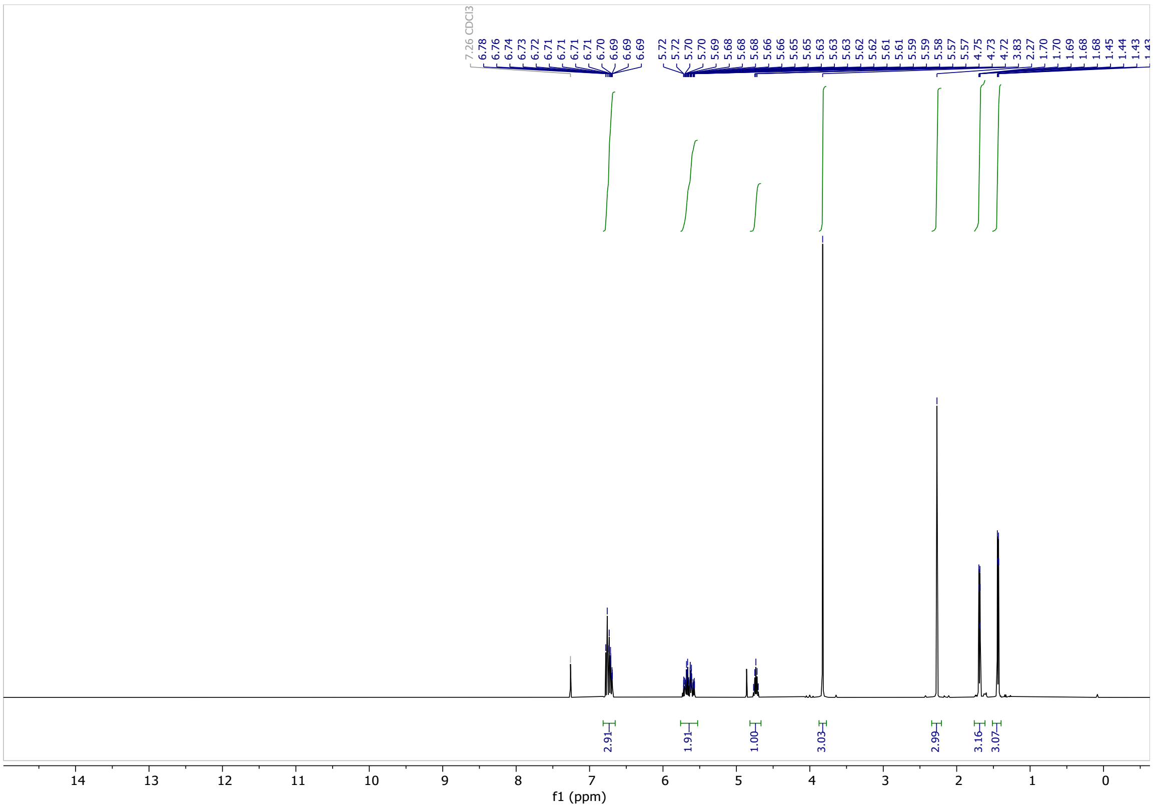

^{13}C NMR (101 MHz, $CDCl_3$)

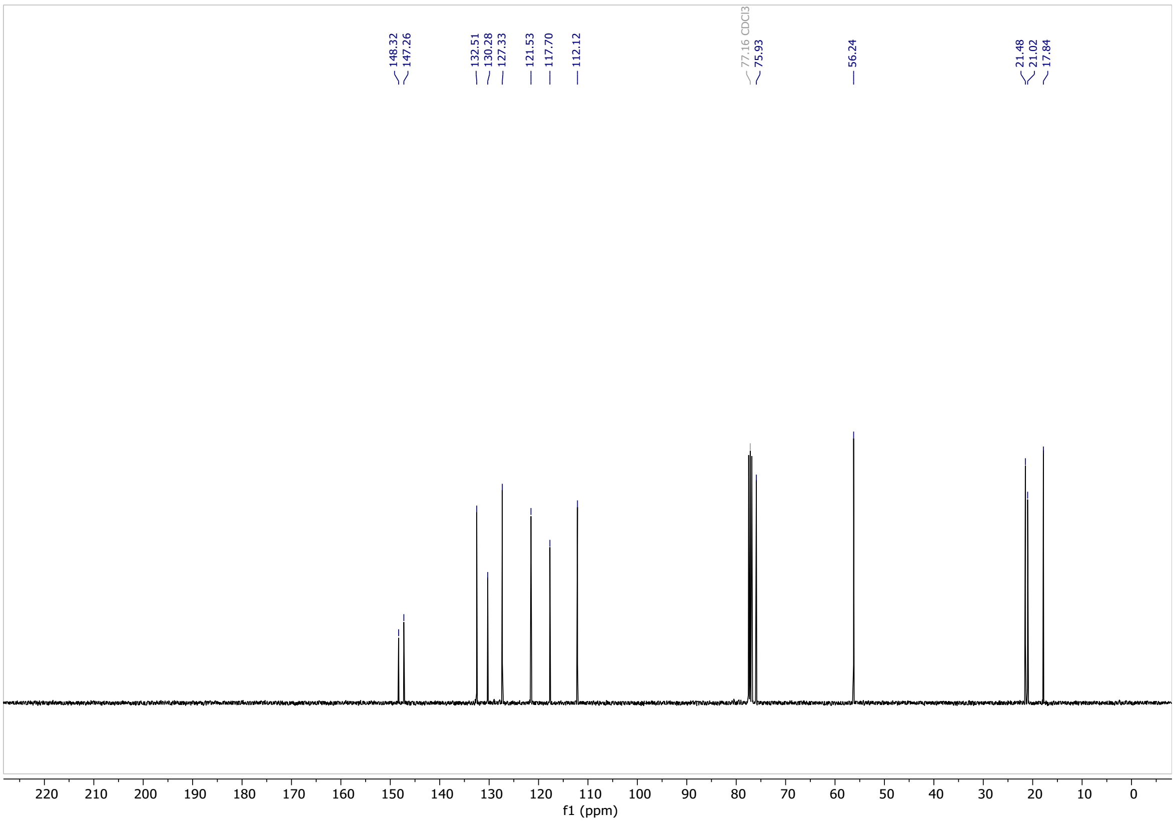

Chromatogram and Results

Instrument Method:	**Heptane_EtOH_99.9_0.1_0.5mlmin_25C_45min**	*B %:*	**0,0**
Column:	**IA-3**	*C %:*	**0,0**
Run Time (min):	**45,00**	*D %:*	**0,1**
Channel:	**UV_VIS_1**		
Wavelength:	**287,26**		

Chromatogram

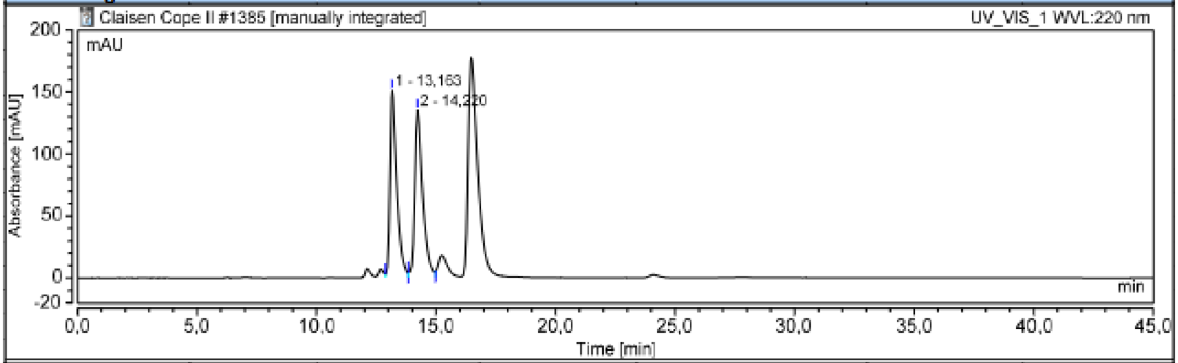

Integration Results

No.	Peak Name	Retention Time min	Area mAU*min	Height mAU	Relative Area %	Relative Height %
1		13,163	50,369	150,899	49,06	52,81
2		14,220	52,291	134,825	50,94	47,19
**Total:**			**102,660**	**285,724**	**100,00**	**100,00**

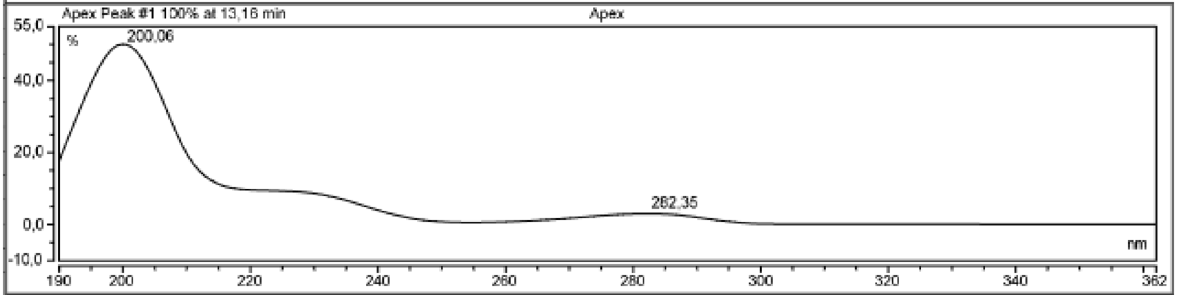

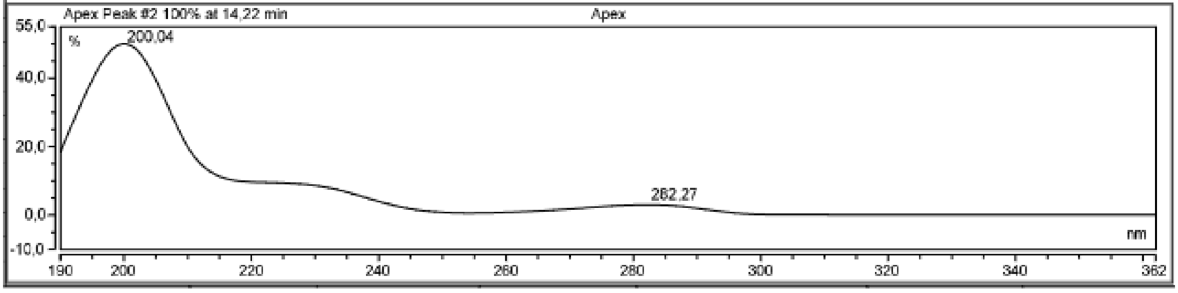

Chromatogram and Results

Instrument Method:	**Heptane_EtOH_99.9_0.1_0.5mlmin_25C_45min**	*B %:*	**0,0**
Column:	**IA-3**	*C %:*	**0,0**
Run Time (min):	**45,00**	*D %:*	**0,1**
Channel:	**UV_VIS_1**		
Wavelength:	**287,26**		

Chromatogram

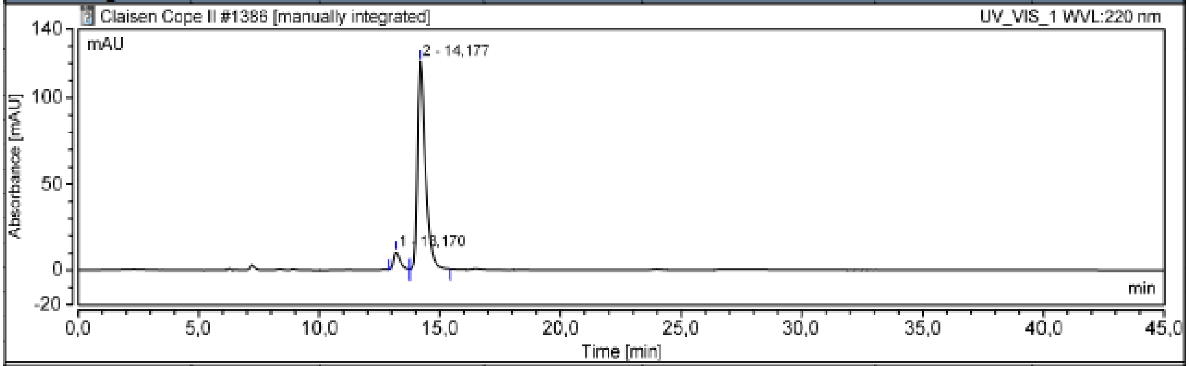

Integration Results

No.	Peak Name	Retention Time min	Area mAU*min	Height mAU	Relative Area %	Relative Height %
1		13,170	3,248	10,350	6,70	7,89
2		14,177	45,252	120,853	93,30	92,11
**Total:**			**48,500**	**131,203**	**100,00**	**100,00**

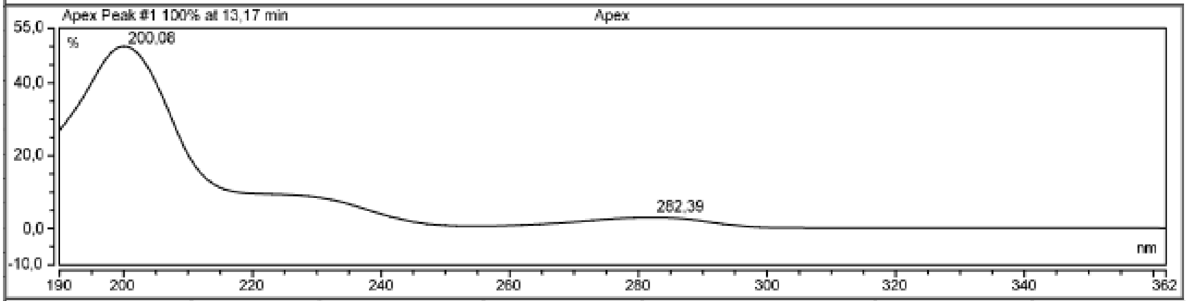

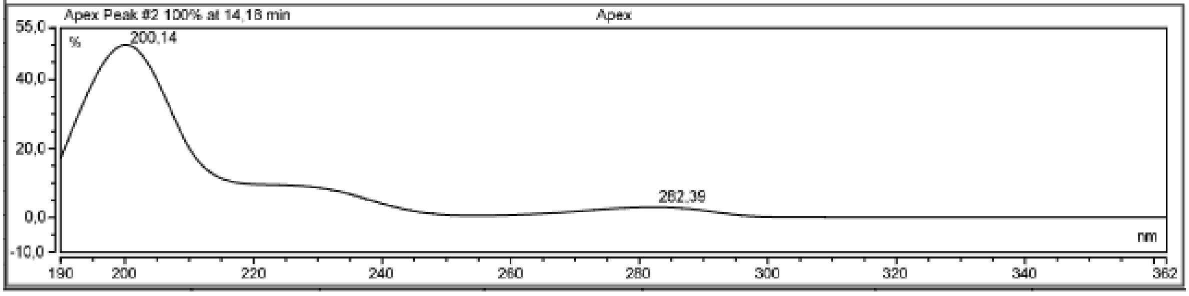

***(R,E)*-1-Fluoro-4-methyl-2-(pent-3-en-2-yloxy)benzene (1o)**

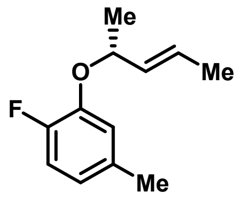

The title compound was synthesized from commercially available 2-fluoro-5-methylphenol (154 mg, 1.22 mmol) following **general procedure A**. The crude material was purified by column chromatography (petroleum ether/ethyl acetate 40:1) to provide the desired product **1o** as colorless oil in 94% yield (223 mg, 1.15 mmol).

$[\alpha]^{20}$= + 60.33 (c 1.00, CH_2Cl_2).

^{1}H NMR (400 MHz, $CDCl_3$) δ 6.97 – 6.89 (m, 1H), 6.78 (dd, *J* = 8.0, 2.1 Hz, 1H), 6.67 (dddd, *J* = 8.2, 4.3, 2.1, 0.8 Hz, 1H), 5.69 (dqd, *J* = 15.4, 6.3, 0.8 Hz, 1H), 5.56 (ddq, *J* = 15.5, 6.8, 1.5 Hz, 1H), 4.72 (p, *J* = 6.4 Hz, 1H), 2.28 (d, *J* = 1.1 Hz, 3H), 1.69 (ddd, *J* = 6.4, 1.5, 0.7 Hz, 3H), 1.43 (d, *J* = 6.3 Hz, 3H).

^{13}C NMR (101 MHz, $CDCl_3$) δ 152.0 (d, *J* = 242.4 Hz), 145.5 (d, *J* = 10.6 Hz), 133.8 (d, *J* = 3.9 Hz), 132.0, 128.0, 121.9 (d, *J* = 6.8 Hz), 119.3 (d, *J* = 1.8 Hz), 115.9 (d, *J* = 18.7 Hz), 76.9, 21.5, 21.2, 17.8.

^{19}F NMR (376 MHz, $CDCl_3$) δ -137.93.

HRMS (ESI): exact mass calculated for $C_{12}H_{15}FNaO^+$ [$(M + Na)^+$], 217.0999; found 217.1004.

87% *ee* (determined by chiral HPLC: Chiralcel® OD column, n-Heptane/iPrOH = 99.9:0.1, 0.5 mL/min λ = 287.3 nm, 25 °C), minor enantiomer. t_r = 10.62 min, major enantiomer. t_r = 11.56 min.

^{1}H NMR (400 MHz, $CDCl_3$)

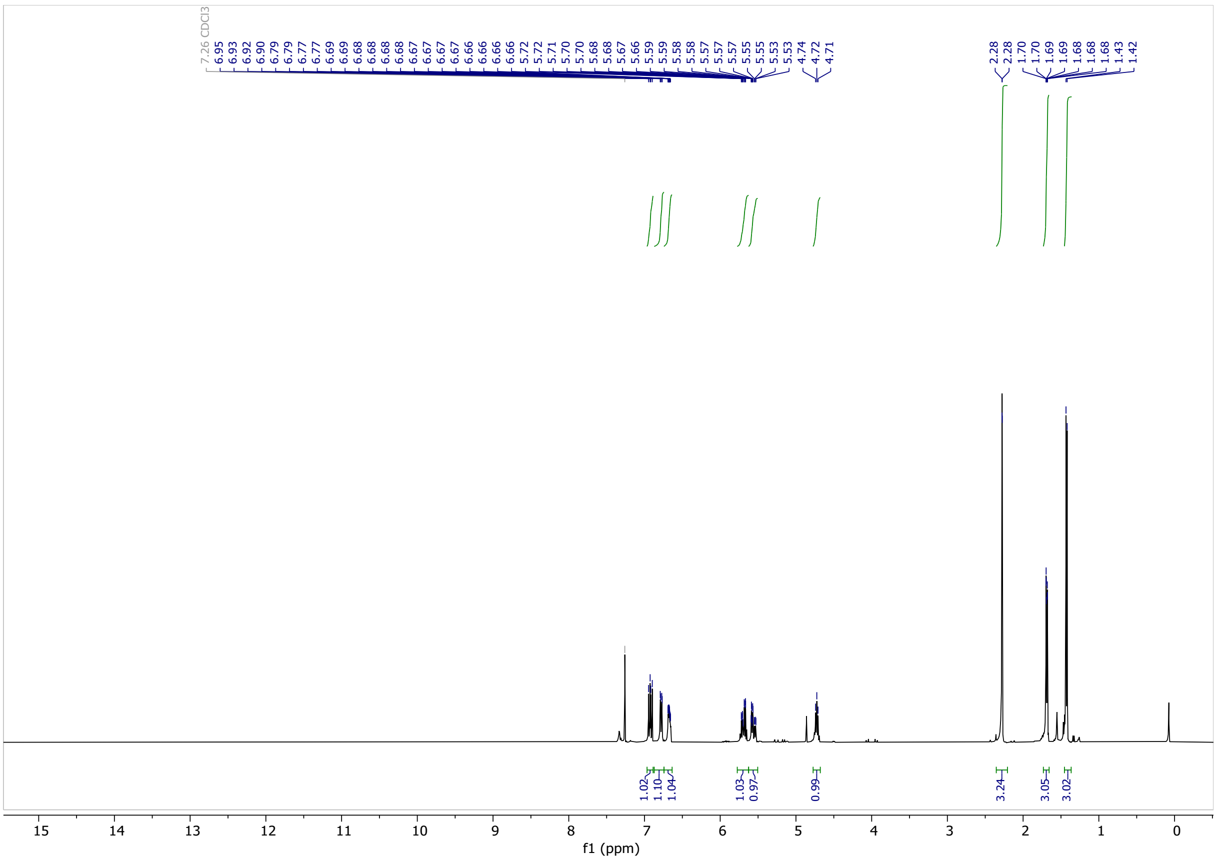

^{13}C NMR (101 MHz, $CDCl_3$)

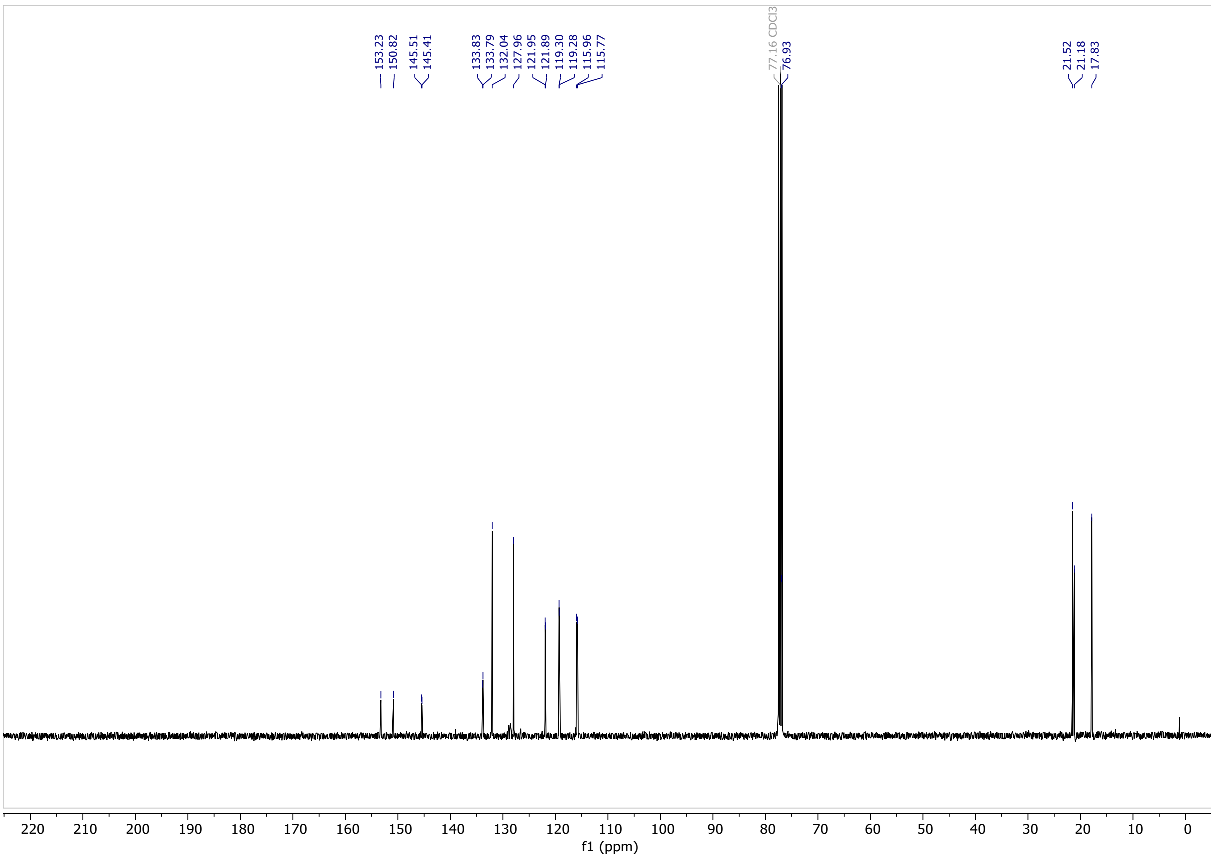

^{19}F NMR (376 MHz, $CDCl_3$)

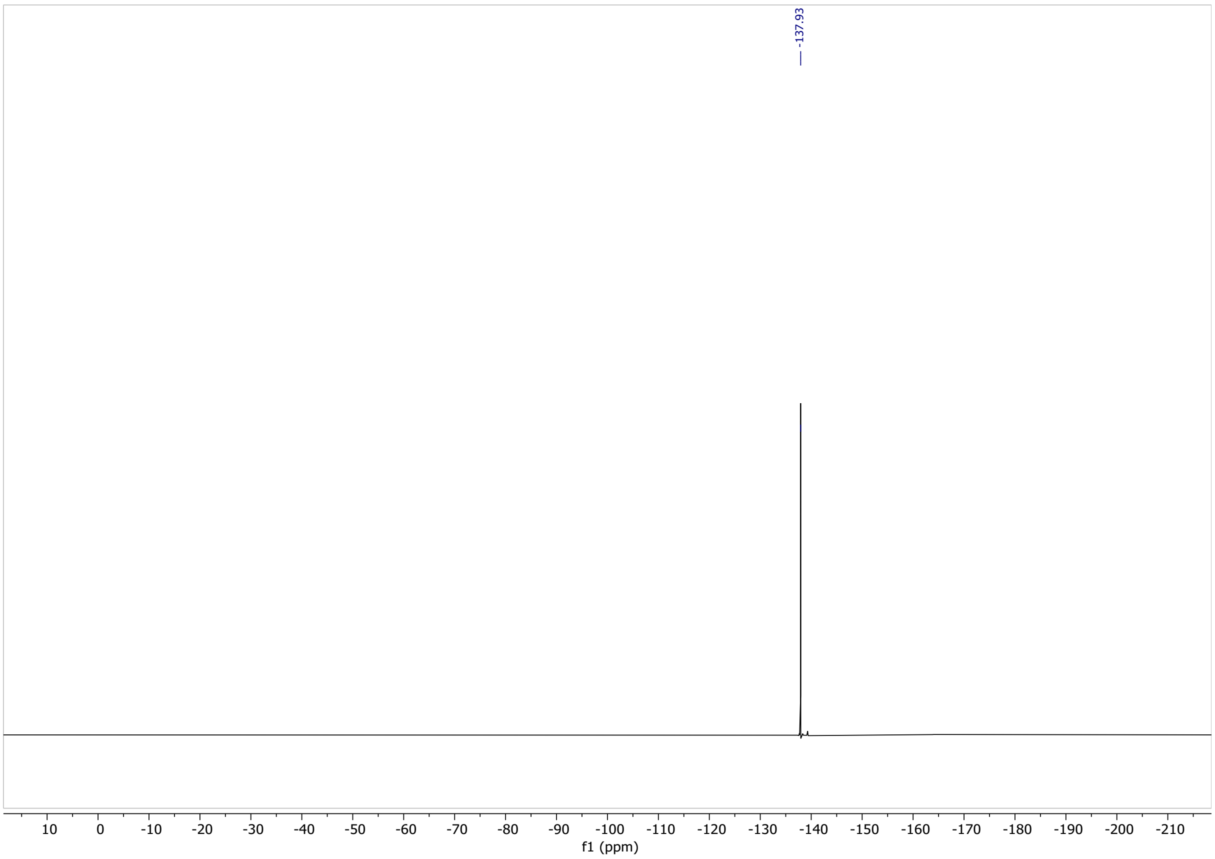

Chromatogram and Results

Instrument Method:	**Heptane_IPA_99.9_0.1_0.5mlmin_25C_20min**	*B %:*	**0,1**
Column:	**OD**	*C %:*	**0,0**
Run Time (min):	**20,00**	*D %:*	**0,0**
Channel:	**UV_VIS_1**		
Wavelength:	**287,26**		

Chromatogram

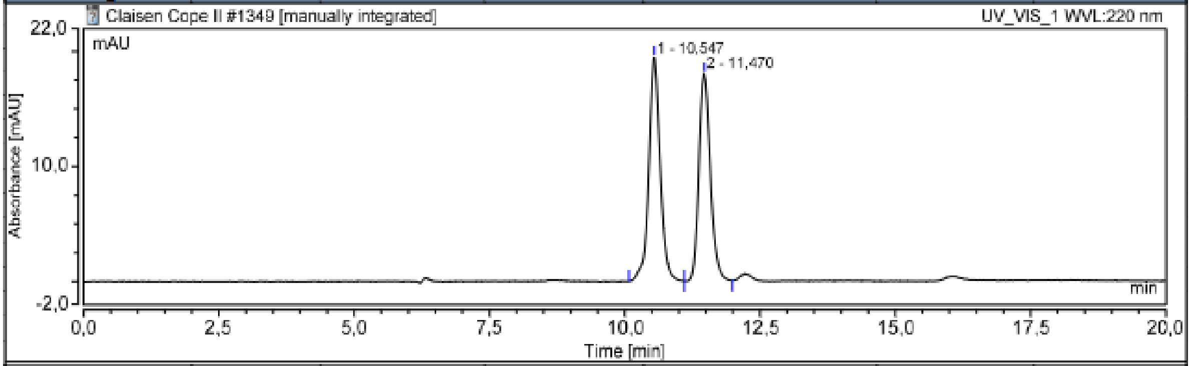

Integration Results

No.	Peak Name	Retention Time<br>min	Area<br>mAU*min	Height<br>mAU	Relative Area<br>%	Relative Height<br>%
1		10,547	4,722	19,463	51,78	51,90
2		11,470	4,396	18,036	48,22	48,10
**Total:**			**9,118**	**37,499**	**100,00**	**100,00**

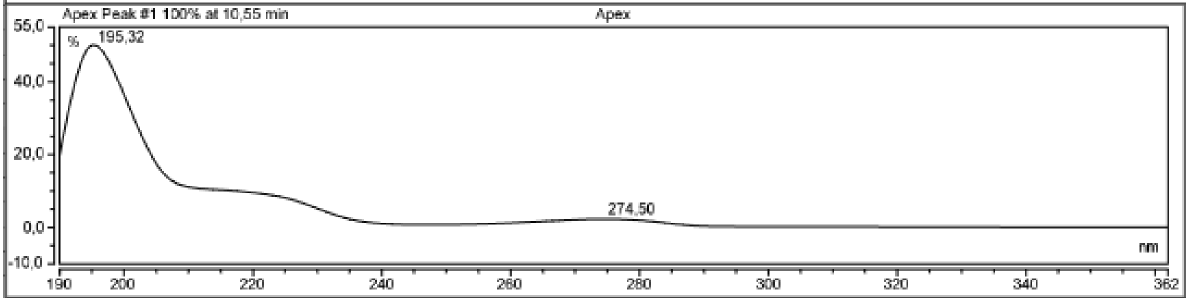

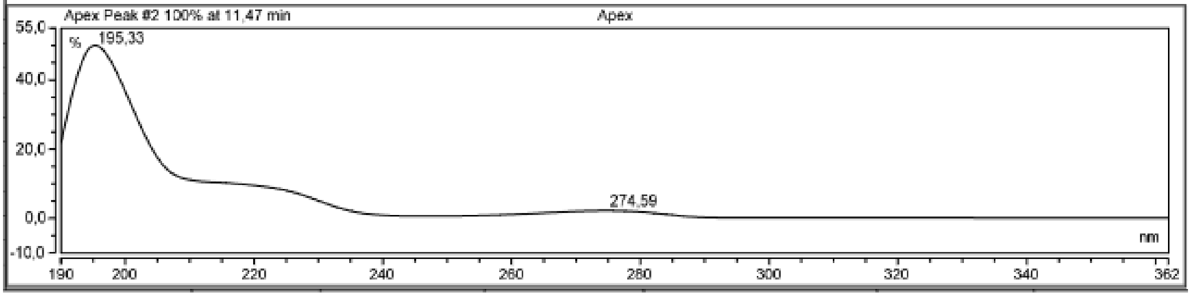

Chromatogram and Results

Instrument Method:	**Heptane_IPA_99.9_0.1_0.5mlmin_25C_20min**	*B %:*	**0,1**
Column:	**OD**	*C %:*	**0,0**
Run Time (min):	**20,00**	*D %:*	**0,0**
Channel:	**UV_VIS_1**		
Wavelength:	**287,26**		

Chromatogram

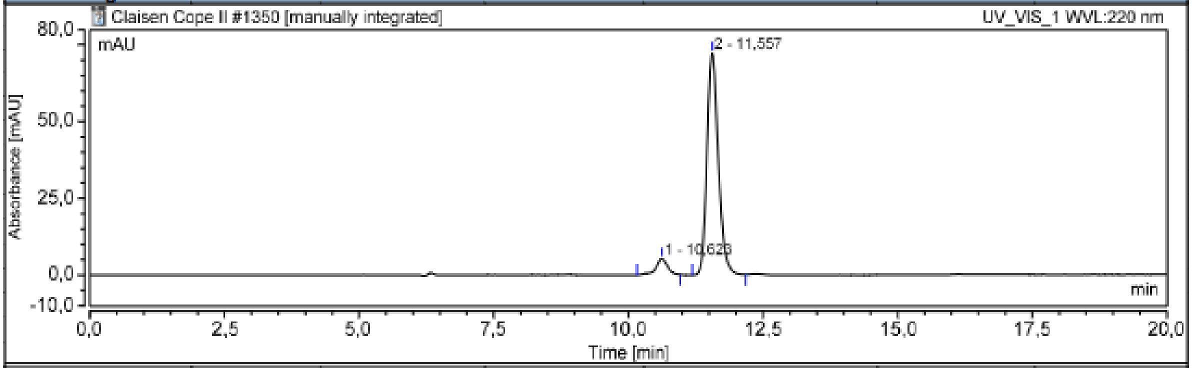

Integration Results

No.	Peak Name	Retention Time min	Area mAU*min	Height mAU	Relative Area %	Relative Height %
1		10,623	1,275	5,251	6,71	6,77
2		11,557	17,734	72,346	93,29	93,23
**Total:**			**19,009**	**77,597**	**100,00**	**100,00**

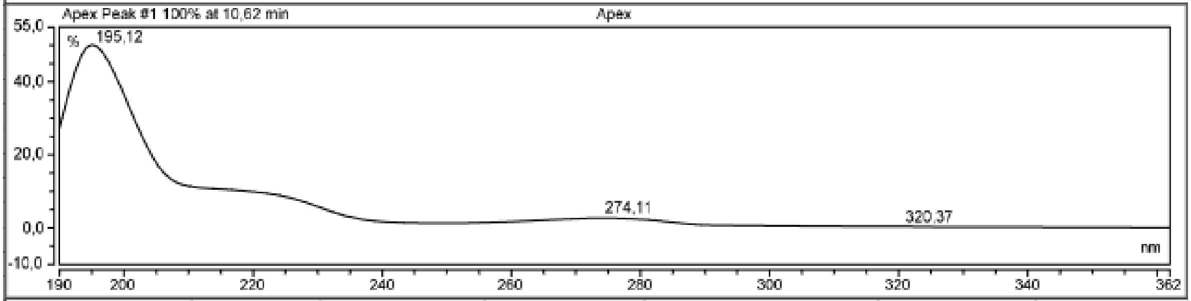

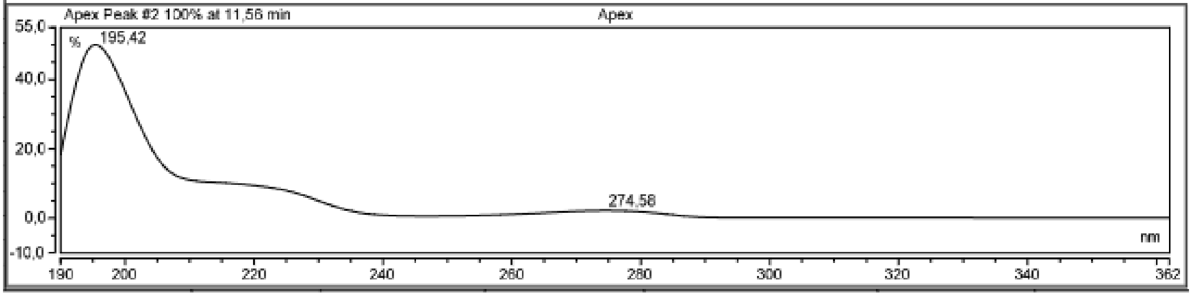

***(R,E)*-1-Chloro-4-methyl-2-(pent-3-en-2-yloxy)benzene (1p)**

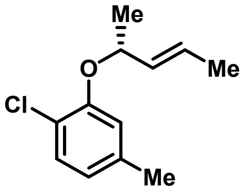

The title compound was synthesized from commercially available 2-chloro-5-methylphenol (149 mg, 1.03 mmol) following **general procedure A**. The crude material was purified by column chromatography (petroleum ether/ethyl acetate 40:1) to provide the desired product **1p** as colorless oil in quantitative yield (217 mg, 1.03 mmol).

$[\alpha]^{20}$= + 22.71 (c 0.95, CH_2Cl_2).

^{1}H NMR (400 MHz, $CDCl_3$) δ 7.21 (d, *J* = 8.0 Hz, 1H), 6.82 – 6.73 (m, 1H), 6.69 (ddd, *J* = 8.0, 2.0, 0.8 Hz, 1H), 5.71 (dqd, *J* = 15.5, 6.3, 0.9 Hz, 1H), 5.58 (ddq, *J* = 15.4, 6.6, 1.5 Hz, 1H), 4.81 – 4.69 (m, 1H), 2.29 (d, *J* = 0.8 Hz, 3H), 1.70 (ddd, *J* = 6.4, 1.5, 0.8 Hz, 3H), 1.45 (d, *J* = 6.3 Hz, 3H).

^{13}C NMR (101 MHz, $CDCl_3$) δ 153.5, 137.6, 132.0, 129.9, 127.7, 122.4, 117.7, 76.5, 21.6, 21.5, 17.9.

85% *ee* (determined by chiral HPLC: Chiralpak® IB column, n-Heptane/EtOH = 99.9:0.1, 0.5 mL/min, λ = 287.3 nm, 25 °C), minor enantiomer. t_r = 11.12 min, major enantiomer. t_r = 12.00 min.

^1H NMR (400 MHz, $CDCl_3$)

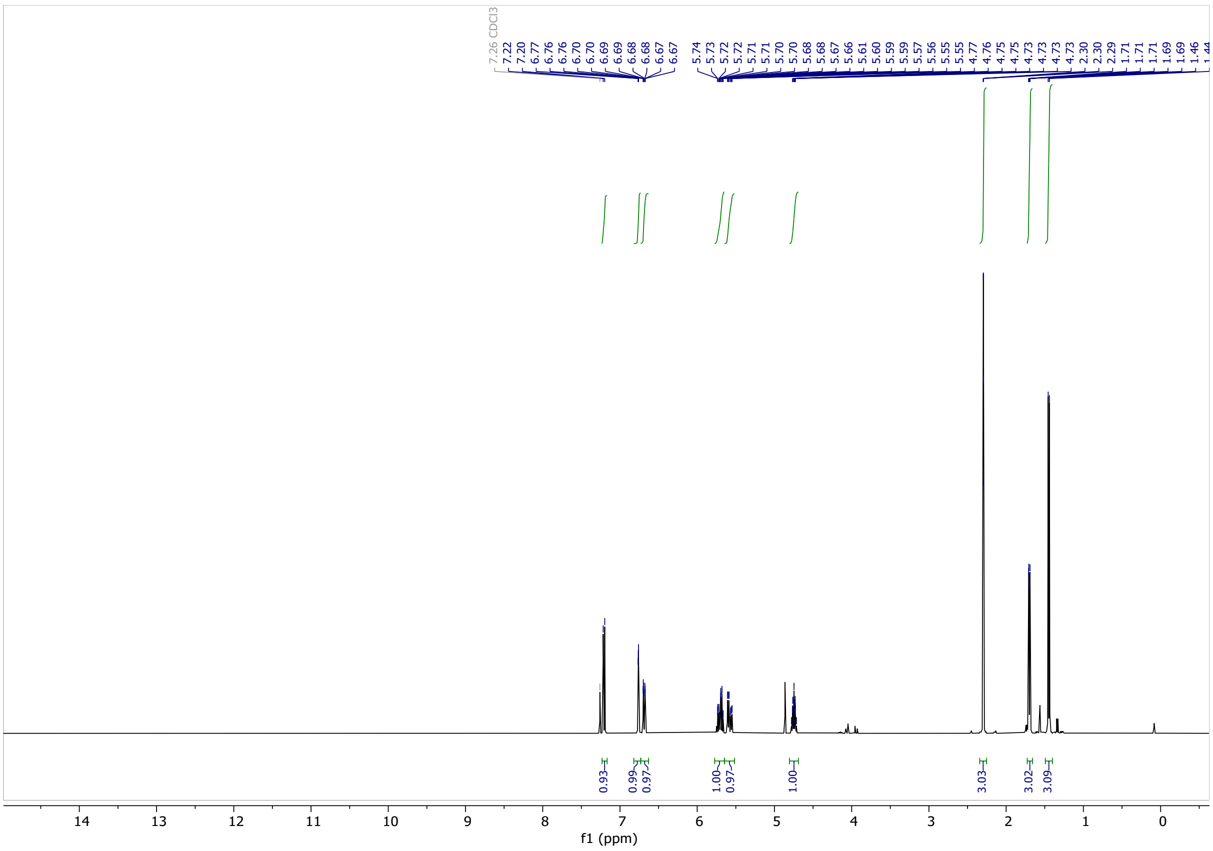

^{13}C NMR (101 MHz, $CDCl_3$)

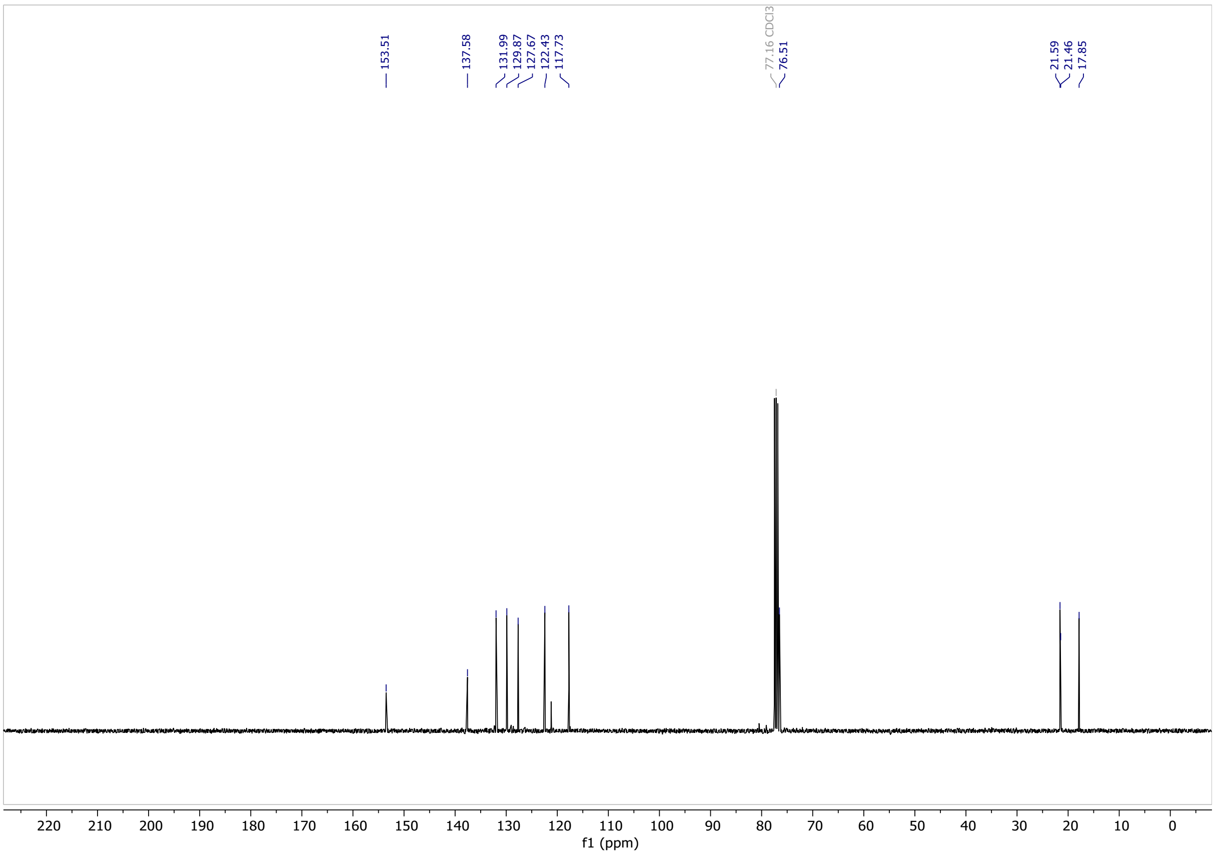

Chromatogram and Results

Instrument Method:	**Heptane_EtOH_99.9_0.1_0.5mlmin_25C_20min**	*B %:*	**0,0**
Column:	**IB**	*C %:*	**0,0**
Run Time (min):	**20,00**	*D %:*	**0,1**
Channel:	**UV_VIS_1**		
Wavelength:	**287,26**		

Chromatogram

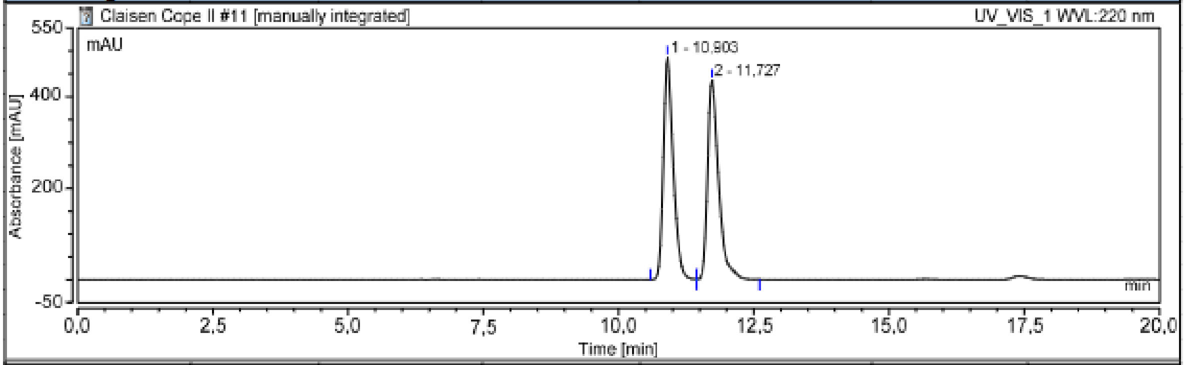

Integration Results

No.	Peak Name	Retention Time<br>min	Area<br>mAU*min	Height<br>mAU	Relative Area<br>%	Relative Height<br>%
1		10,903	103,740	487,244	49,95	52,76
2		11,727	103,963	436,317	50,05	47,24
**Total:**			**207,703**	**923,561**	**100,00**	**100,00**

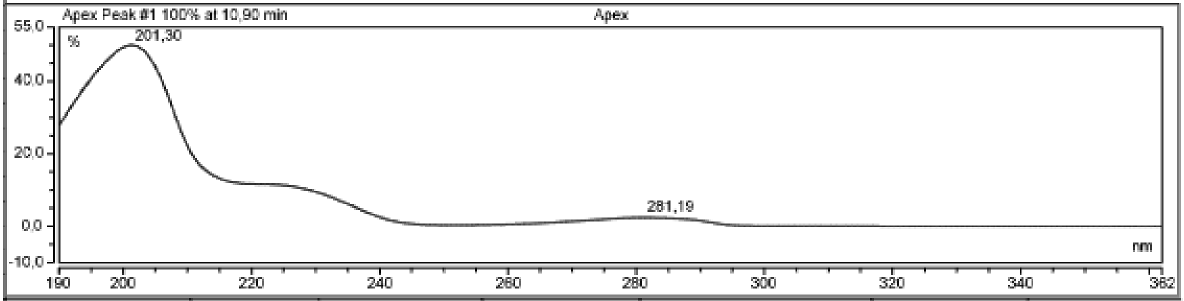

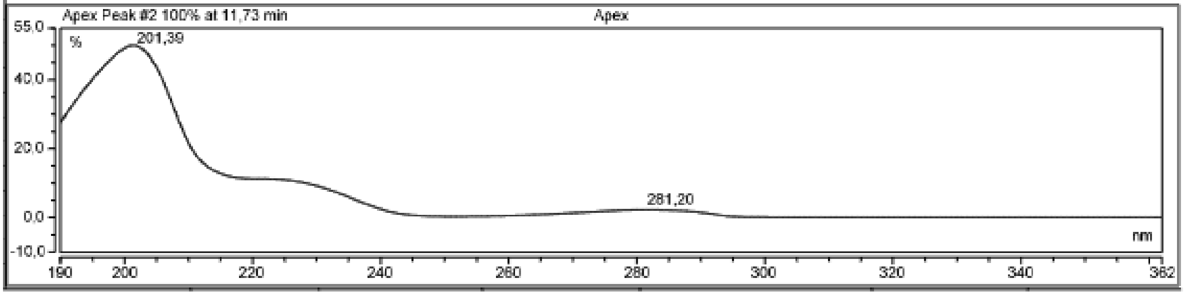

Chromatogram and Results

Instrument Method:	**Heptane_EtOH_99.9_0.1_0.5mlmin_25C_20min**	*B %:*	**0,0**
Column:	**IB**	*C %:*	**0,0**
Run Time (min):	**20,00**	*D %:*	**0,1**
Channel:	**UV_VIS_1**		
Wavelength:	**287,26**		

Chromatogram

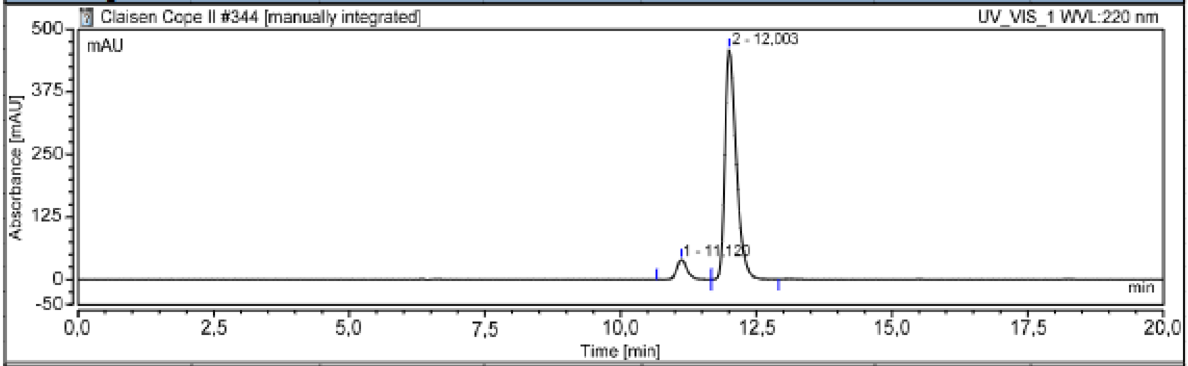

Integration Results

No.	Peak Name	Retention Time min	Area mAU*min	Height mAU	Relative Area %	Relative Height %
1		11,120	9,015	40,149	7,47	8,03
2		12,003	111,729	459,781	92,53	91,97
**Total:**			**120,745**	**499,930**	**100,00**	**100,00**

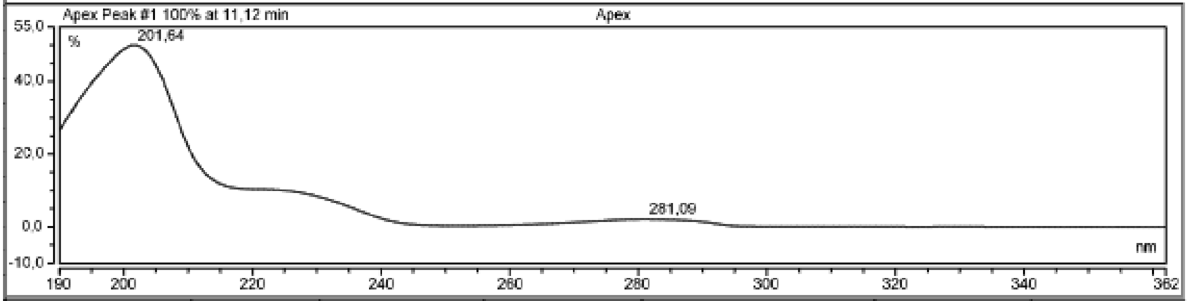

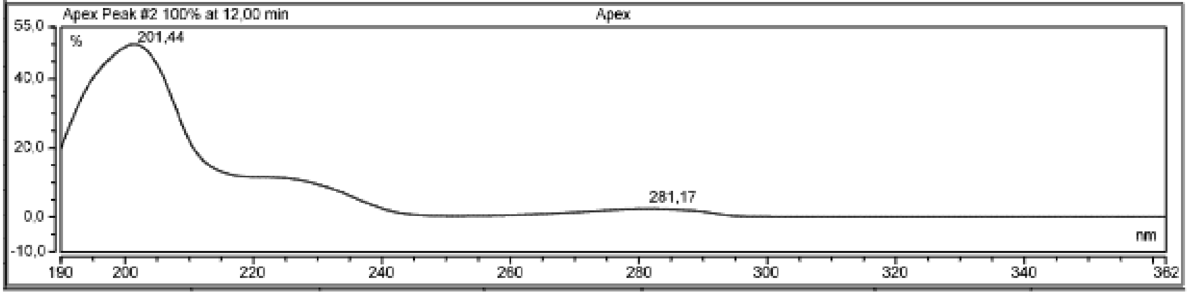

(R,E)-1-Bromo-4-methyl-2-(pent-3-en-2-yloxy)benzene (1q)

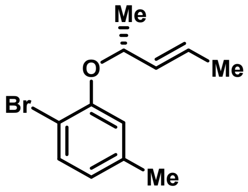

The title compound was synthesized from commercially available 2-bromo-5-methylphenol (152 mg, 0.80 mmol) following **general procedure A**. The crude material was purified by column chromatography (petroleum ether/ethyl acetate 40:1) to provide the desired product **1q** as colorless oil in 98% yield (200 mg, 0.78 mmol).

$[\alpha]^{20}$= + 9.46 (c 0.80, CH_2Cl_2).

^{1}H NMR (400 MHz, $CDCl_3$) δ 7.37 (d, *J* = 8.0 Hz, 1H), 6.73 (d, *J* = 1.9 Hz, 1H), 6.63 (ddd, *J* = 8.1, 2.1, 0.8 Hz, 1H), 5.71 (dqd, *J* = 15.4, 6.4, 0.9 Hz, 1H), 5.57 (ddq, *J* = 15.4, 6.5, 1.5 Hz, 1H), 4.75 (tt, *J* = 7.4, 5.7 Hz, 1H), 2.28 (d, *J* = 0.7 Hz, 3H), 1.70 (ddd, *J* = 6.4, 1.5, 0.8 Hz, 3H), 1.45 (d, *J* = 6.3 Hz, 3H).

^{13}C NMR (101 MHz, $CDCl_3$) δ 154.5, 138.4, 132.9, 132.0, 127.6, 123.0, 117.5, 110.4, 76.5, 21.6, 21.5, 17.9.

86% *ee* (determined by chiral HPLC: Chiralcel® OD column, n-Heptane/iPrOH = 99.9:0.1, 0.5 mL/min, λ = 287.3 nm, 25 °C), minor enantiomer. t_r = 12.76 min, major enantiomer. t_r = 14.41 min.

^{1}H NMR (400 MHz, $CDCl_3$)

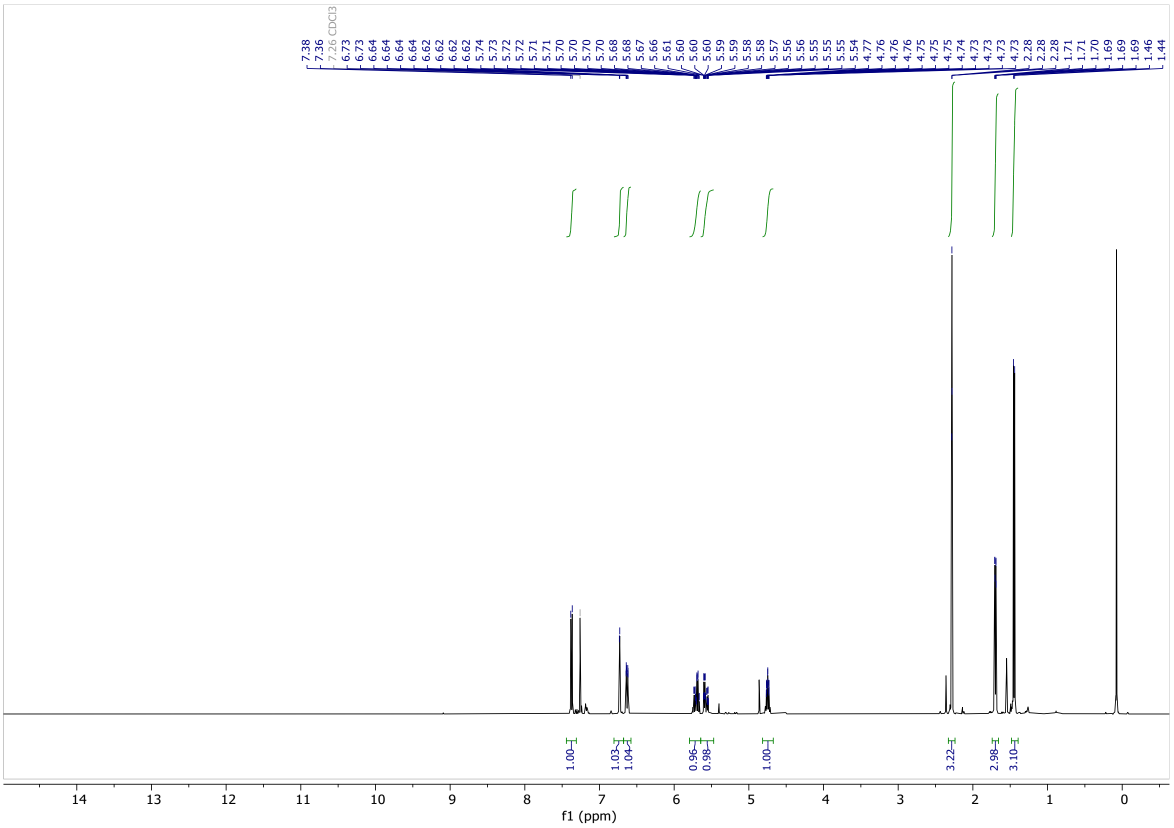

^{13}C NMR (101 MHz, $CDCl_3$)

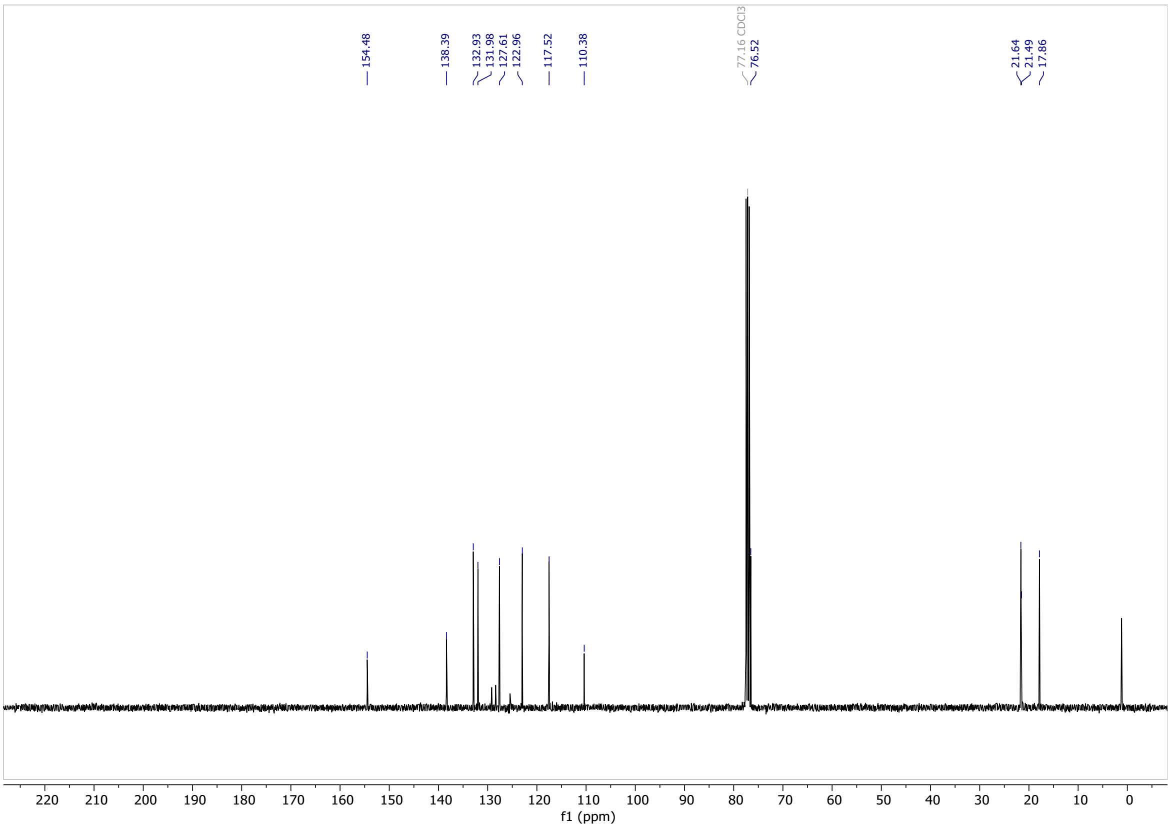

Chromatogram and Results

Instrument Method:	**Heptane_IPA_99.9_0.1_0.5mlmin_25C_20min**	*B %:*	**0,1**
Column:	**OD**	*C %:*	**0,0**
Run Time (min):	**20,00**	*D %:*	**0,0**
Channel:	**UV_VIS_1**		
Wavelength:	**287,26**		

Chromatogram

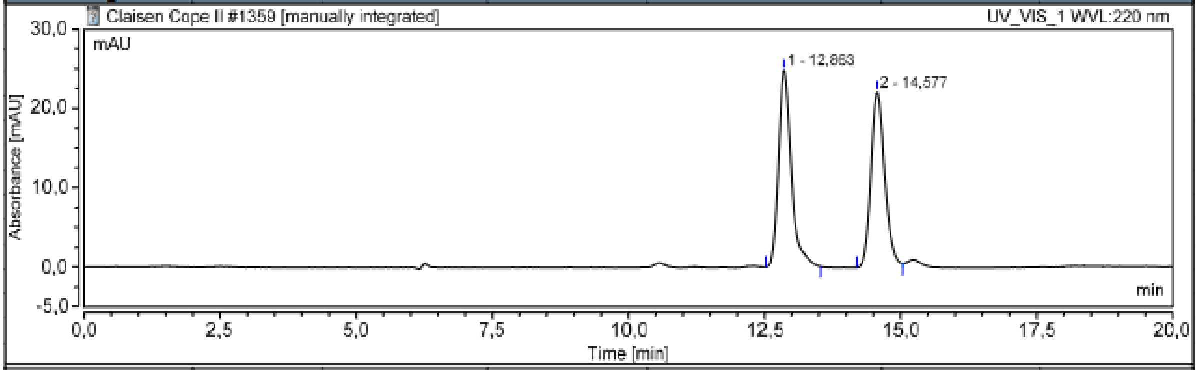

Integration Results

No.	Peak Name	Retention Time min	Area mAU*min	Height mAU	Relative Area %	Relative Height %
1		12,863	6,758	24,804	50,96	52,86
2		14,577	6,503	22,124	49,04	47,14
**Total:**			**13,261**	**46,929**	**100,00**	**100,00**

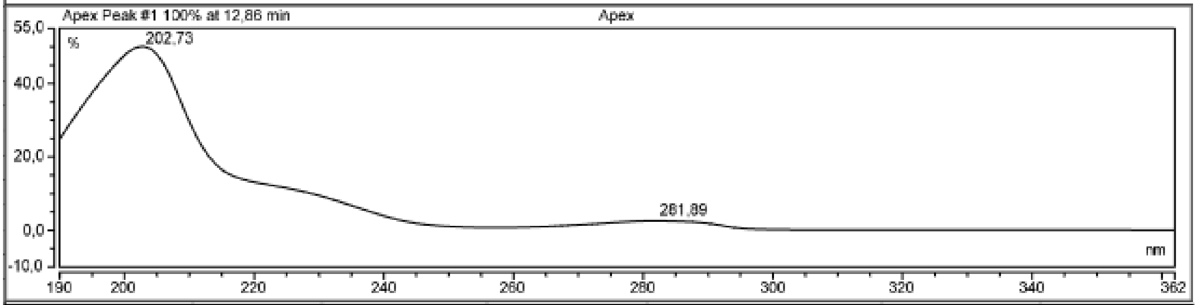

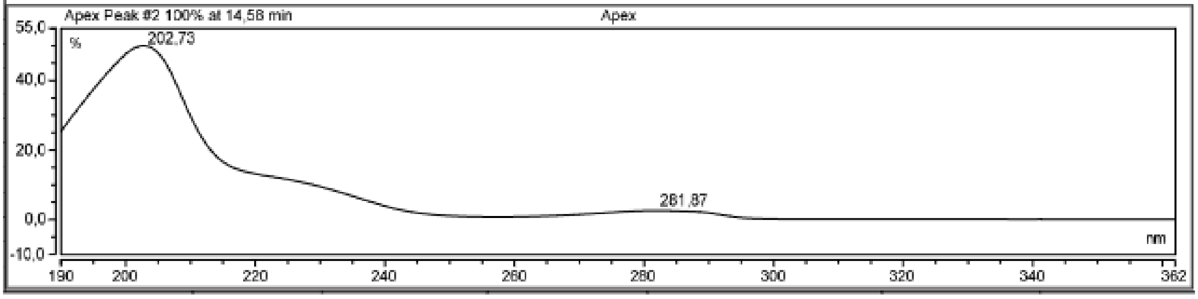

Chromatogram and Results

Instrument Method:	**Heptane_IPA_99.9_0.1_0.5mlmin_25C_20min**	*B %:*	**0,1**
Column:	**OD**	*C %:*	**0,0**
Run Time (min):	**20,00**	*D %:*	**0,0**
Channel:	**UV_VIS_1**		
Wavelength:	**287,26**		

Chromatogram

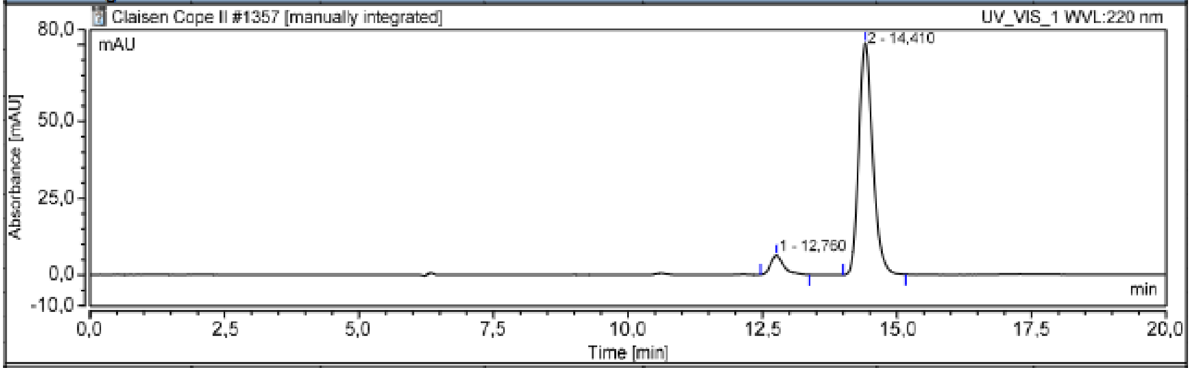

Integration Results

No.	Peak Name	Retention Time min	Area mAU*min	Height mAU	Relative Area %	Relative Height %
1		12,760	1,758	6,279	7,21	7,67
2		14,410	22,632	75,591	92,79	92,33
**Total:**			**24,390**	**81,870**	**100,00**	**100,00**

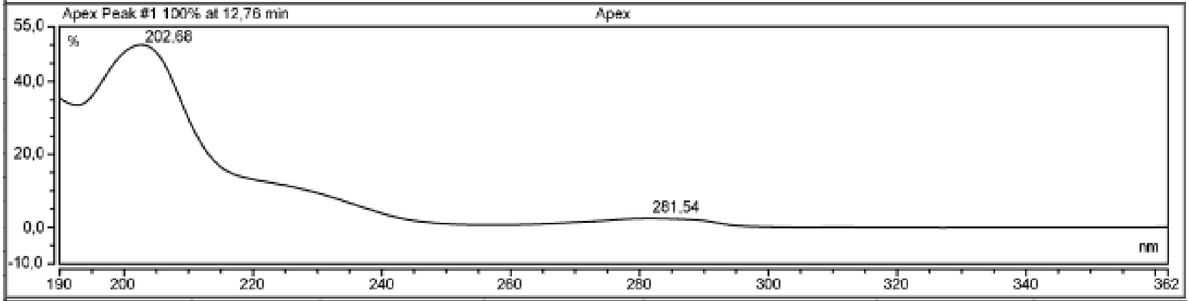

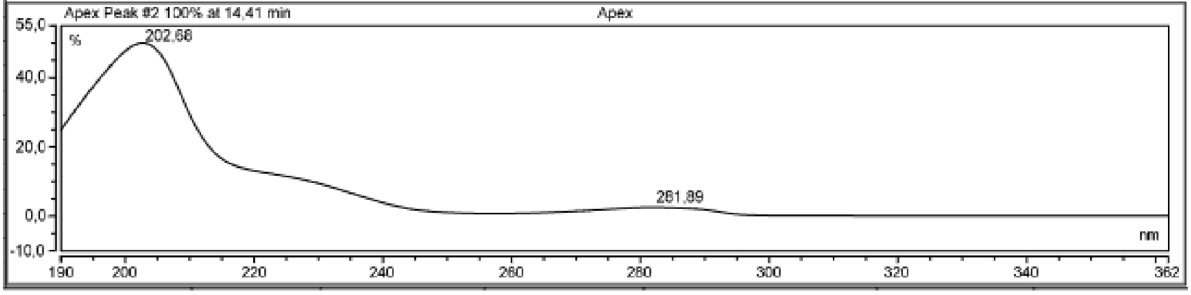

***(R,E)*-4-Bromo-1-methoxy-2-(pent-3-en-2-yloxy)benzene (1r)**

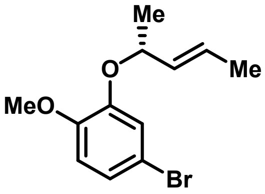

The title compound was synthesized from commercially available 5-bromo-2-methoxyphenol (153 mg, 0.75 mmol) following **general procedure A**. The crude material was purified by column chromatography (petroleum ether/ethyl acetate 40:1) to provide the desired product **1r** as colorless oil in 89% yield (181 mg, 0.67 mmol).

$[\alpha]^{20}$= + 58.21 (c 0.85, CH_2Cl_2).

^{1}H NMR (400 MHz, $CDCl_3$) δ 7.05 – 6.95 (m, 2H), 6.77 – 6.64 (m, 1H), 5.69 (dqd, *J* = 15.4, 6.4, 0.9 Hz, 1H), 5.55 (ddq, *J* = 15.5, 6.8, 1.5 Hz, 1H), 4.71 (p, *J* = 6.5 Hz, 1H), 3.81 (s, 3H), 1.73 – 1.64 (m, 3H), 1.44 (d, *J* = 6.3 Hz, 3H).

^{13}C NMR (101 MHz, $CDCl_3$) δ 149.6, 148.3, 131.7, 128.1, 123.8, 119.5, 113.2, 112.5, 76.3, 56.2, 21.4, 17.8.

86% *ee* (determined by chiral HPLC: Chiralcel® OD column, n-Heptane/iPrOH = 99.5:0.5, 0.7 mL/min, λ = 287.3 nm, 25 °C), minor enantiomer. t_r = 20.75 min, major enantiomer. t_r = 23.35 min.

^{1}H NMR (400 MHz, $CDCl_3$)

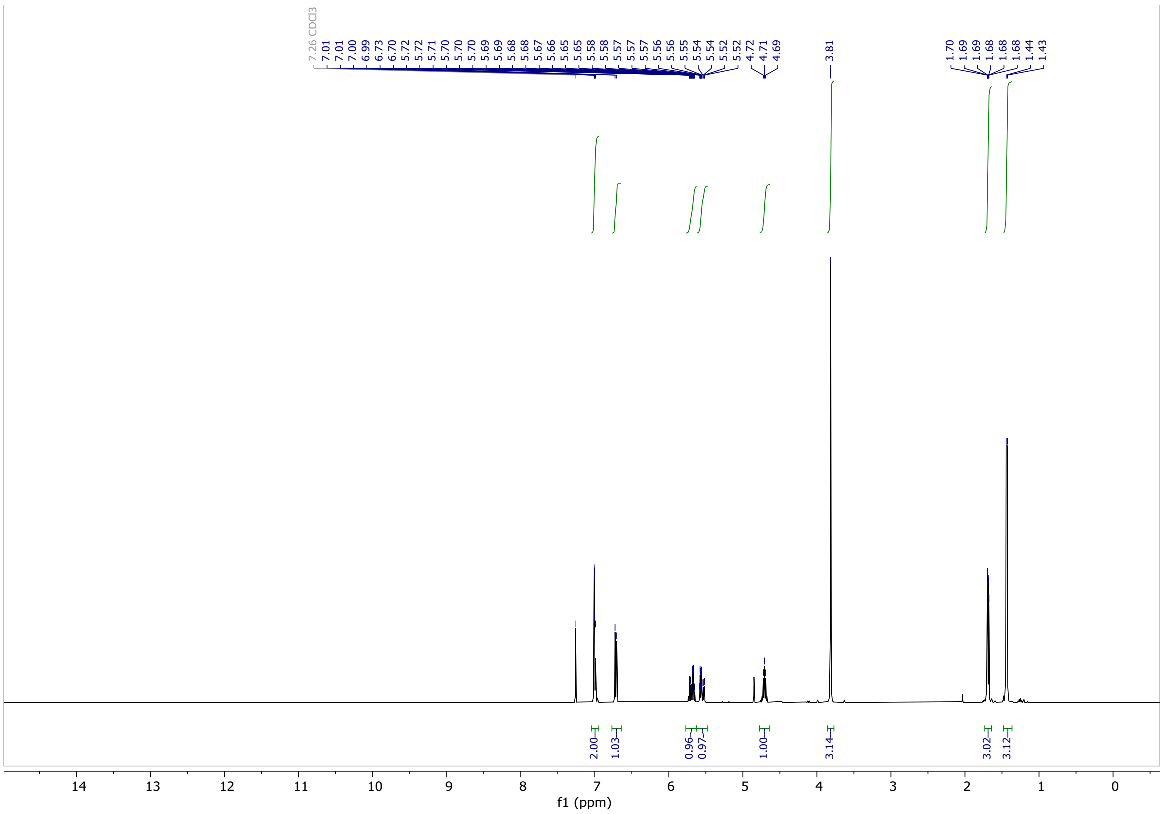

^{13}C NMR (101 MHz, $CDCl_3$)

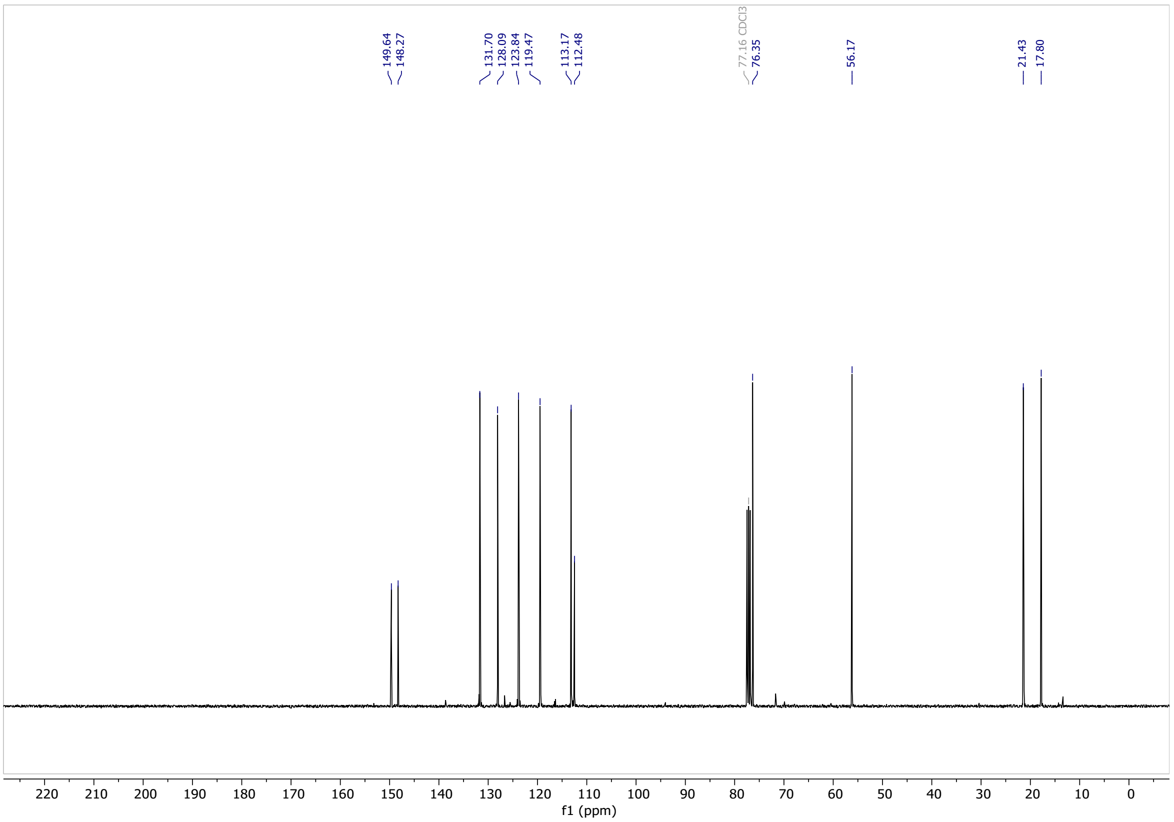

Chromatogram and Results

Instrument Method:	**Heptane_IPA_99.5_0.5_0.7mlmin_25C_30min**	*B %:*	**0,1**
Column:	**OD**	*C %:*	**0,0**
Run Time (min):	**30,00**	*D %:*	**0,0**
Channel:	**UV_VIS_1**		
Wavelength:	**287,26**		

Chromatogram

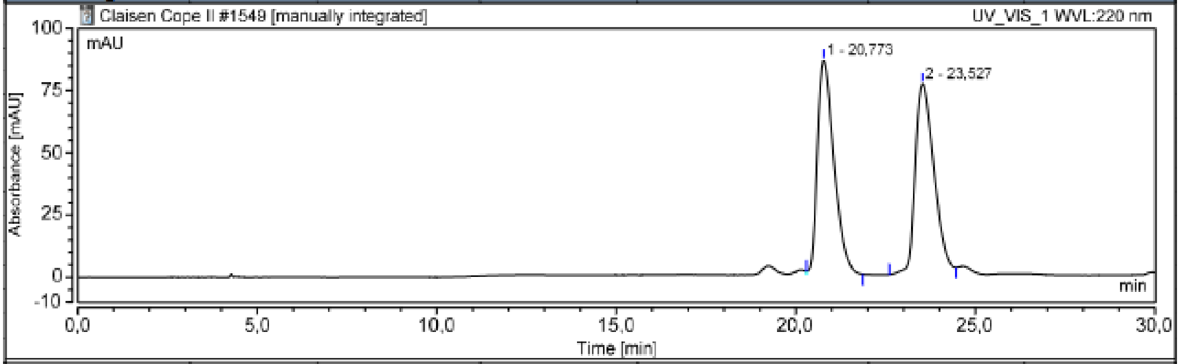

Integration Results

No.	Peak Name	Retention Time<br>min	Area<br>mAU*min	Height<br>mAU	Relative Area<br>%	Relative Height<br>%
1		20,773	46,004	86,052	51,01	53,34
2		23,527	44,179	75,283	48,99	46,66
**Total:**			**90,184**	**161,334**	**100,00**	**100,00**

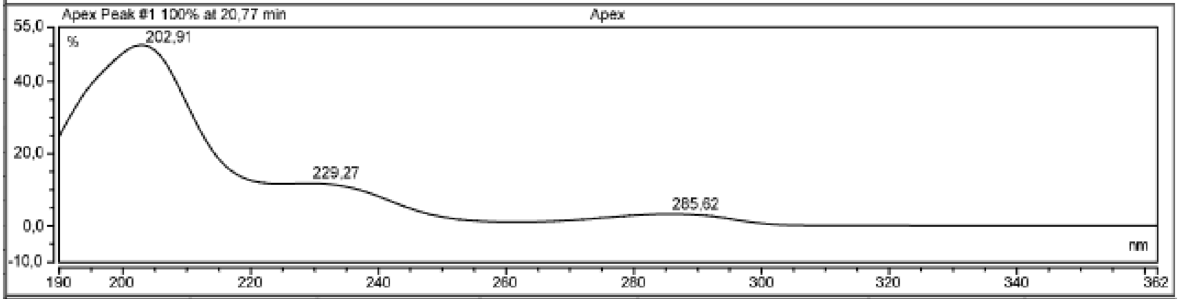

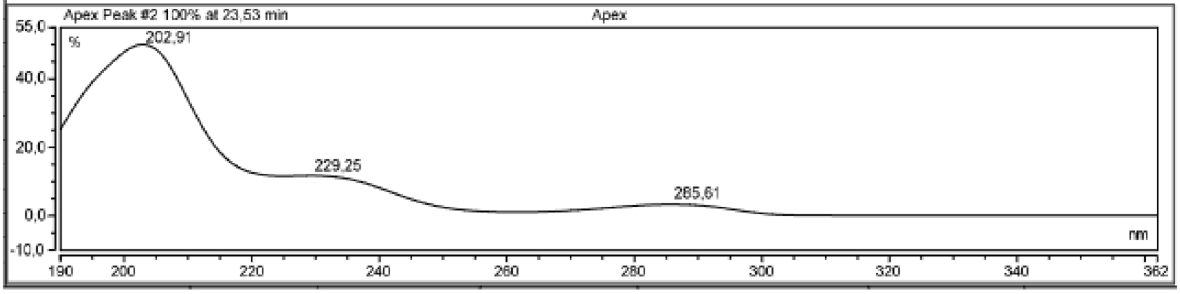

Chromatogram and Results

Instrument Method:	**Heptane_IPA_99.5_0.5_0.7mlmin_25C_30min**	*B %:*	**0,1**
Column:	**OD**	*C %:*	**0,0**
Run Time (min):	**30,00**	*D %:*	**0,0**
Channel:	**UV_VIS_1**		
Wavelength:	**287,26**		

Chromatogram

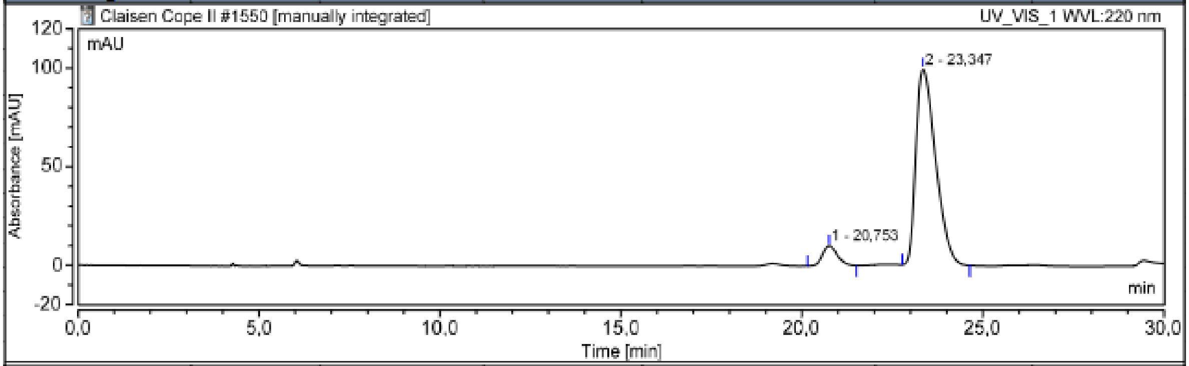

Integration Results

No.	Peak Name	Retention Time min	Area mAU*min	Height mAU	Relative Area %	Relative Height %
1		20,753	4,778	10,127	7,09	9,25
2		23,347	62,566	99,381	92,91	90,75
**Total:**			**67,344**	**109,508**	**100,00**	**100,00**

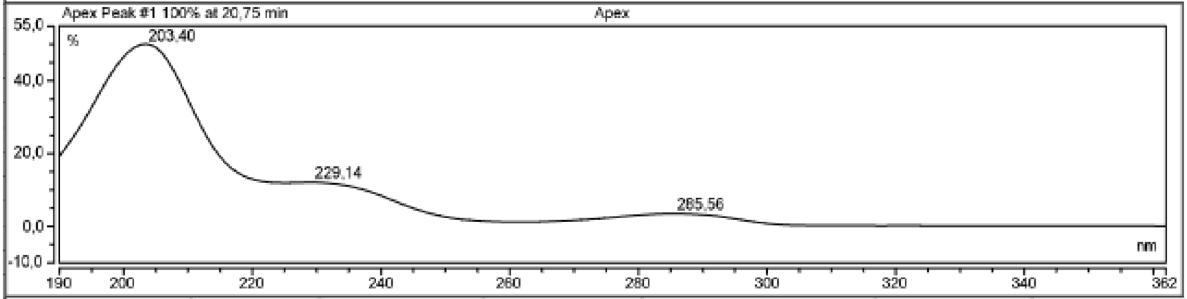

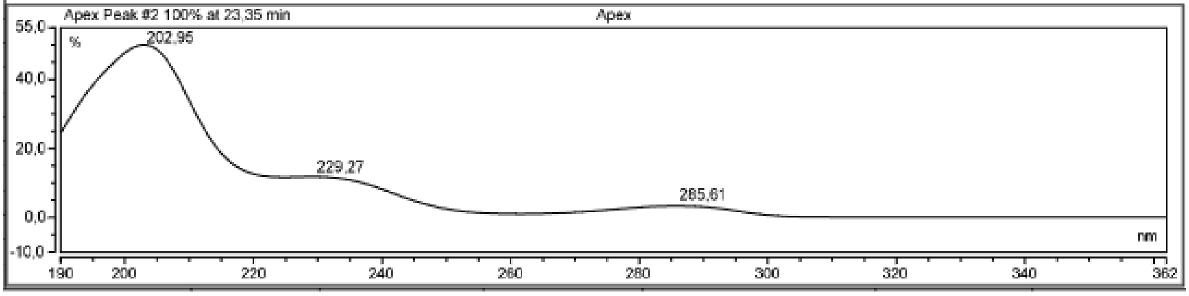

1-Ethoxy-2-(((*R,E*)-pent-3-en-2-yl)oxy)-4-((*E*)-prop-1-en-1-yl)benzene (1s)

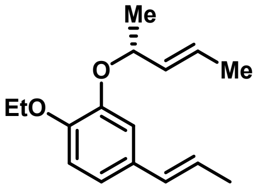

The title compound was synthesized from commercially available (*E*)-2-ethoxy-5-(prop-1-en-1-yl)phenol (152 mg, 0.84 mmol) following **general procedure A**. The crude material was purified by column chromatography (petroleum ether/ethyl acetate 40:1) to provide the desired product **1s** as colorless oil in 75% yield (156 mg, 0.63 mmol).

$[\alpha]^{20}$= + 50.25 (c 0.95, CH_2Cl_2).

^{1}H NMR (400 MHz, $CDCl_3$) δ 6.95 (d, *J* = 2.1 Hz, 1H), 6.86 (dd, *J* = 8.3, 2.1 Hz, 1H), 6.80 (d, *J* = 8.3 Hz, 1H), 6.30 (dq, *J* = 15.7, 1.7 Hz, 1H), 6.06 (dq, *J* = 15.7, 6.6 Hz, 1H), 5.78 – 5.53 (m, 2H), 4.72 (p, *J* = 6.1 Hz, 1H), 4.06 (q, *J* = 7.0 Hz, 2H), 1.86 (dd, *J* = 6.6, 1.7 Hz, 3H), 1.74 – 1.63 (m, 3H), 1.48 – 1.37 (m, 6H).

^{13}C NMR (101 MHz, $CDCl_3$) δ 149.4, 147.9, 132.7, 131.2, 130.7, 127.3, 123.6, 119.7, 115.8, 114.1, 76.8, 64.7, 21.5, 18.5, 17.8, 15.0.

HRMS (ESI): exact mass calculated for $C_{16}H_{23}O_2^+$ [$(M + H)^+$], 247.1693; found 247.1702.

83% *ee* (determined by chiral HPLC: Chiralpak® IB column, n-Heptane/EtOH = 99.7:0.3, 0.5 mL/min, λ = 287.3 nm, 25 °C), major enantiomer. t_r = 12.65 min, minor enantiomer. t_r = 13.87 min.

^{1}H NMR (400 MHz, $CDCl_3$)

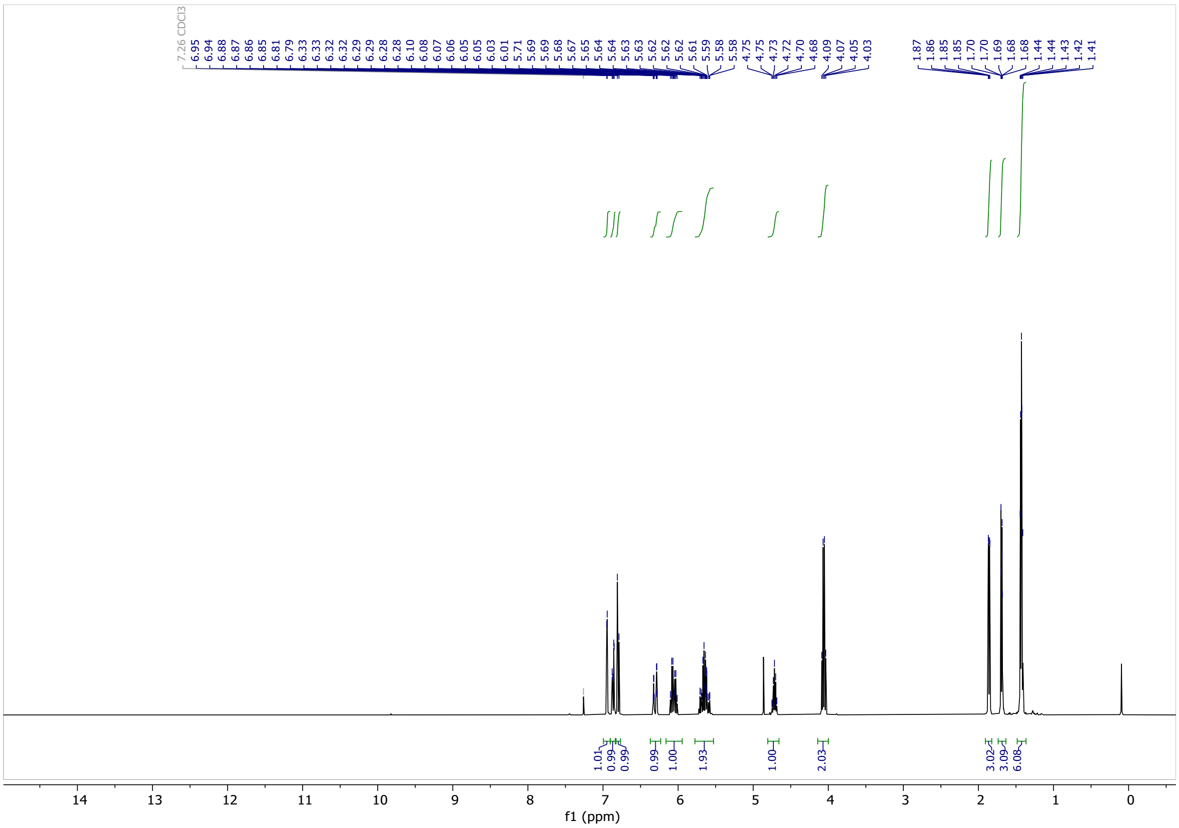

^{13}C NMR (101 MHz, $CDCl_3$)

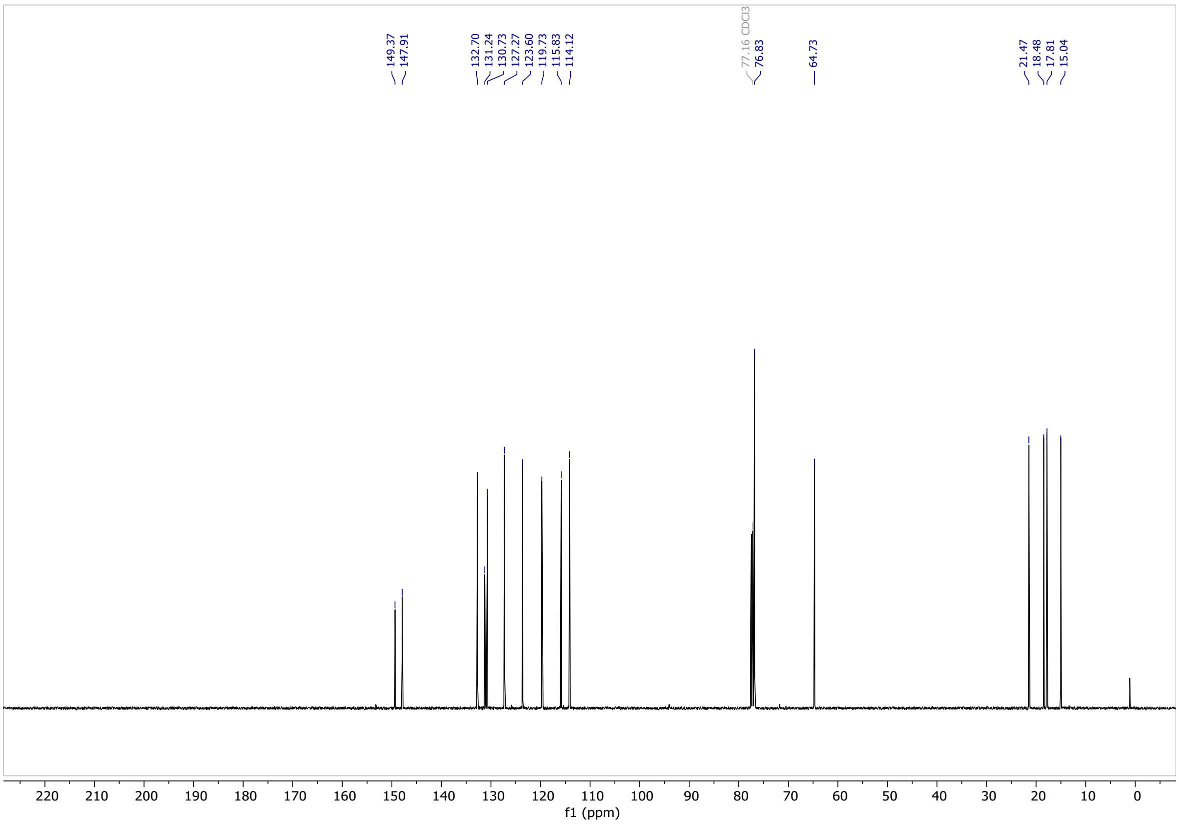

Chromatogram and Results

Instrument Method:	**Heptane_EtOH_99.7_0.3_0.5mlmin_25C_20min**	*B %:*	**0,0**
Column:	**IB**	*C %:*	**0,0**
Run Time (min):	**20,00**	*D %:*	**0,3**
Channel:	**UV_VIS_1**		
Wavelength:	**287,26**		

Chromatogram

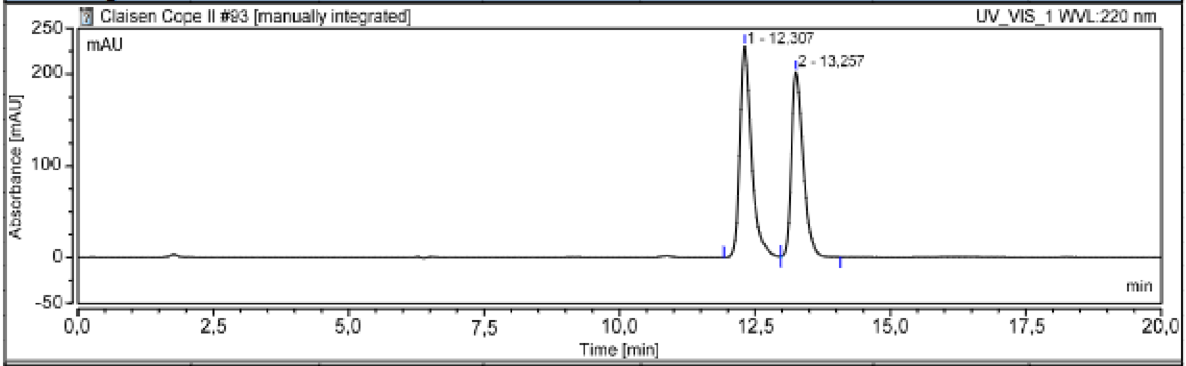

Integration Results

No.	Peak Name	Retention Time<br>min	Area<br>mAU*min	Height<br>mAU	Relative Area<br>%	Relative Height<br>%
1		12,307	57,889	230,925	53,29	53,27
2		13,257	50,735	202,550	46,71	46,73
**Total:**			**108,625**	**433,475**	**100,00**	**100,00**

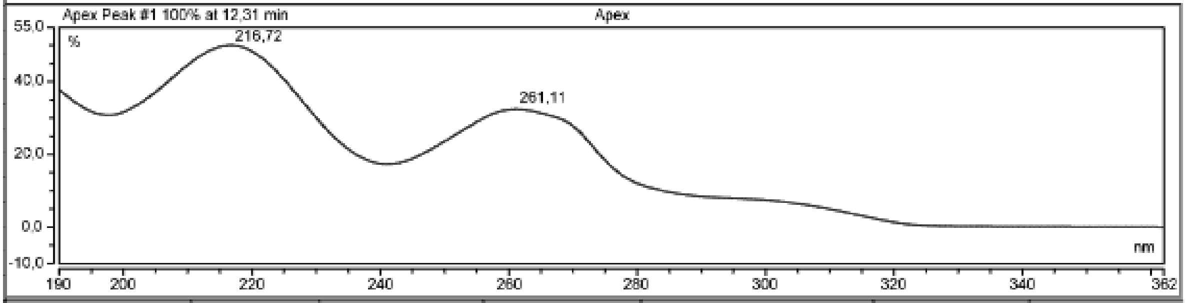

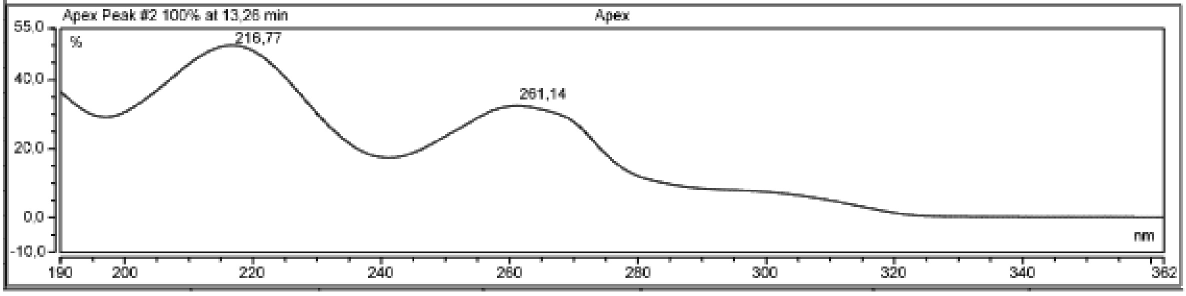

Chromatogram and Results

Instrument Method:	**Heptane_EtOH_99.7_0.3_0.5mlmin_25C_20min**	*B %:*	**0,0**
Column:	**IB**	*C %:*	**0,0**
Run Time (min):	**20,00**	*D %:*	**0,3**
Channel:	**UV_VIS_1**		
Wavelength:	**287,26**		

Chromatogram

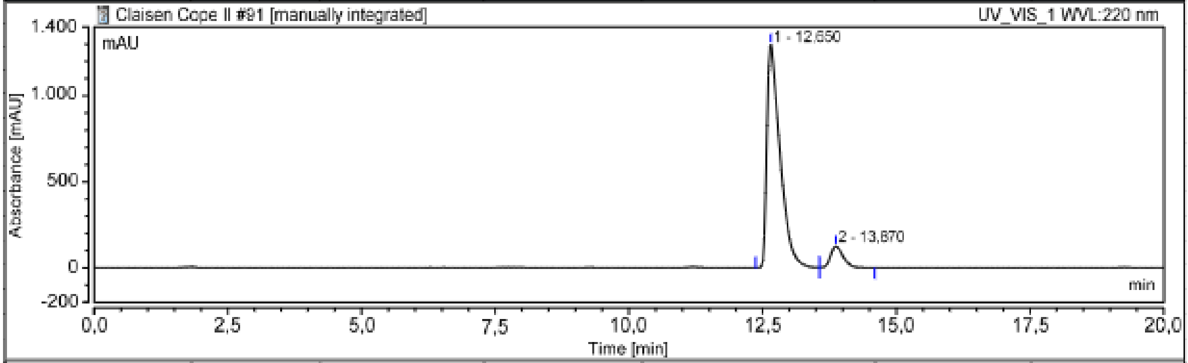

Integration Results

No.	Peak Name	Retention Time min	Area mAU*min	Height mAU	Relative Area %	Relative Height %
1		12,650	367,885	1296,496	91,54	91,20
2		13,870	34,017	125,116	8,46	8,80
**Total:**			**401,902**	**1421,612**	**100,00**	**100,00**

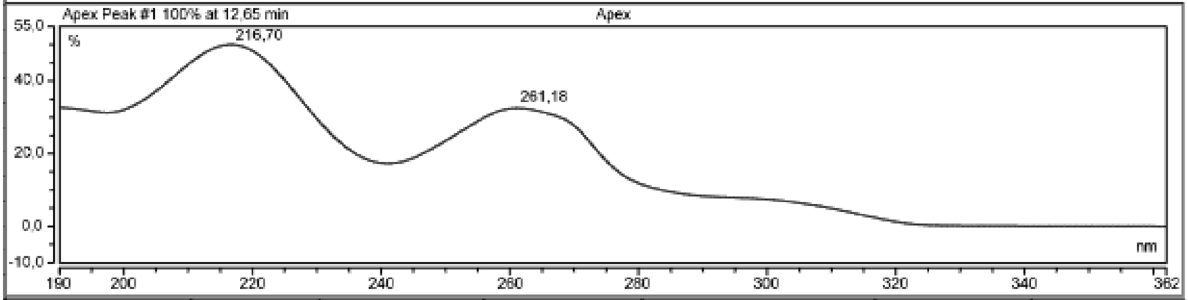

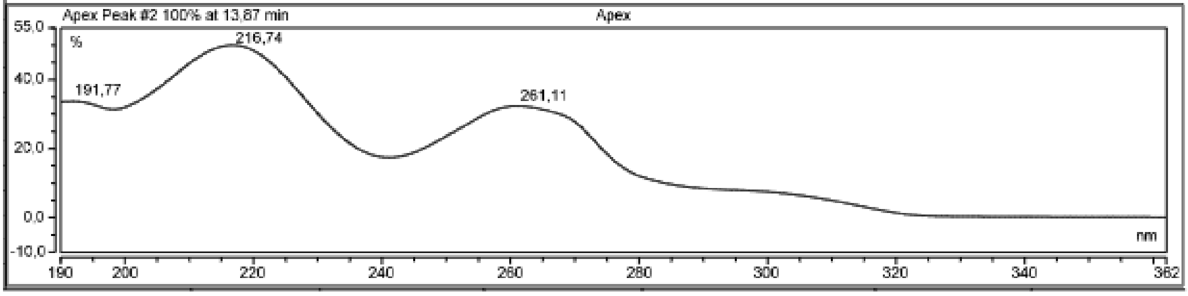

(E)-1,4-Dimethyl-2-((4-phenylbut-3-en-2-yl)oxy)benzene (4a)

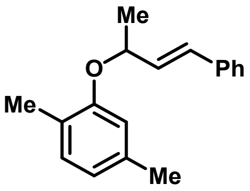

The title compound was synthesized from commercially available 2,5-dimethylphenol (156 mg, 1.28 mmol, 1 equiv.) following <u>*racemic*</u> **general procedure A** using carbonate **Carb 2**. The crude material was purified by column chromatography (petroleum ether/ethyl acetate 40:1) to provide the desired product **4a** as colorless oil in 98% yield (317 mg, 1.26 mmol).

^{1}H NMR (400 MHz, $CDCl_3$) δ 7.45 – 7.16 (m, 5H), 7.09 – 7.00 (m, 1H), 6.77 – 6.57 (m, 3H), 6.33 (ddd, *J* = 16.1, 7.9, 6.0 Hz, 1H), 5.00 – 4.90 (m, 1H), 2.30 (s, 3H), 2.25 (s, 3H), 1.58 – 1.50 (m, 3H).

^{13}C NMR (101 MHz, $CDCl_3$) δ 156.3, 136.9, 136.5, 131.4, 130.6, 130.2, 128.7, 127.7, 126.6, 124.7, 121.3, 114.8, 74.9, 21.9, 21.5, 16.2, 16.2.

^{1}H NMR (400 MHz, $CDCl_3$)

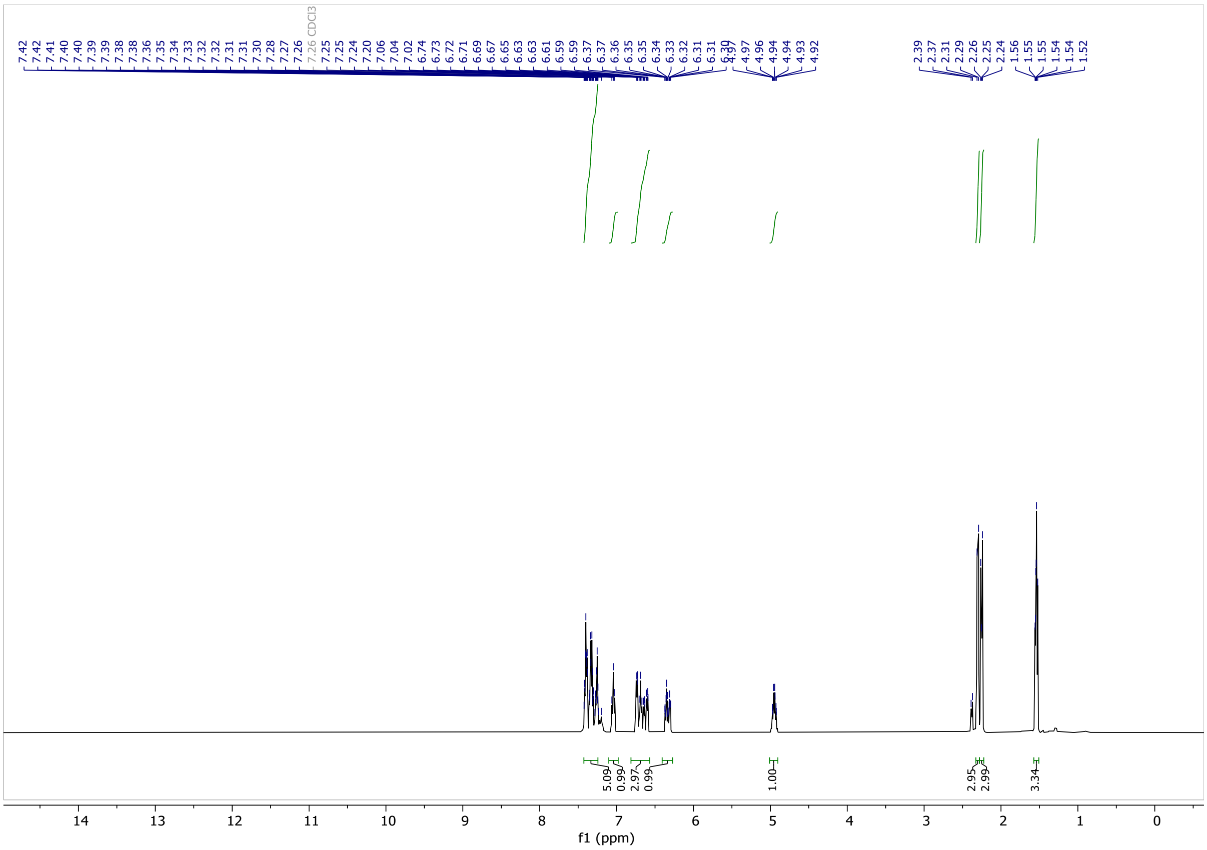

^{13}C NMR (101 MHz, $CDCl_3$)

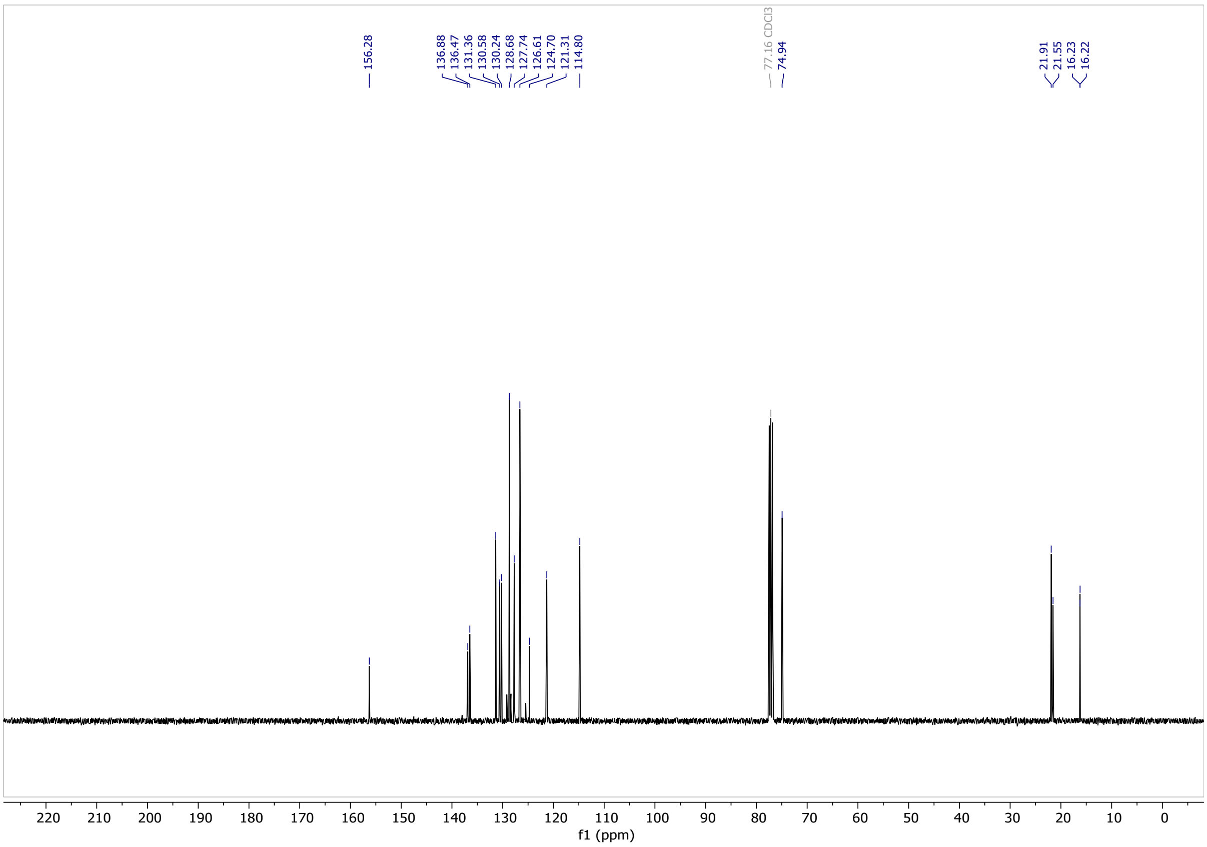

2-(Cyclohex-2-en-1-yloxy)-1,4-dimethylbenzene (4b)

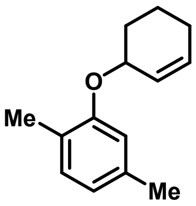

The title compound was synthesized from commercially available 2,5-dimethylphenol (150 mg, 1.23 mmol, 1 equiv.) following *racemic* **general procedure A** using carbonate **Carb 3**. The crude material was purified by column chromatography (petroleum ether/ethyl acetate 40:1) to provide the desired product **4b** as colorless oil in 96% yield (238 mg, 1.18 mmol).

^{1}H NMR (400 MHz, $CDCl_3$) δ 7.03 (d, *J* = 7.5 Hz, 1H), 6.72 (d, *J* = 1.6 Hz, 1H), 6.68 (dd, *J* = 7.4, 1.6 Hz, 1H), 6.00 – 5.85 (m, 2H), 4.80 – 4.72 (m, 1H), 2.32 (s, 3H), 2.19 (s, 3H), 2.16 – 2.02 (m, 2H), 1.99 – 1.78 (m, 3H), 1.66 (dddd, *J* = 12.9, 11.7, 7.4, 2.3 Hz, 1H).

^{13}C NMR (101 MHz, $CDCl_3$) δ 156.2, 136.5, 131.7, 130.7, 127.1, 124.9, 121.1, 114.4, 71.5, 28.9, 25.3, 21.5, 19.3, 16.2.

^{1}H NMR (400 MHz, $CDCl_3$)

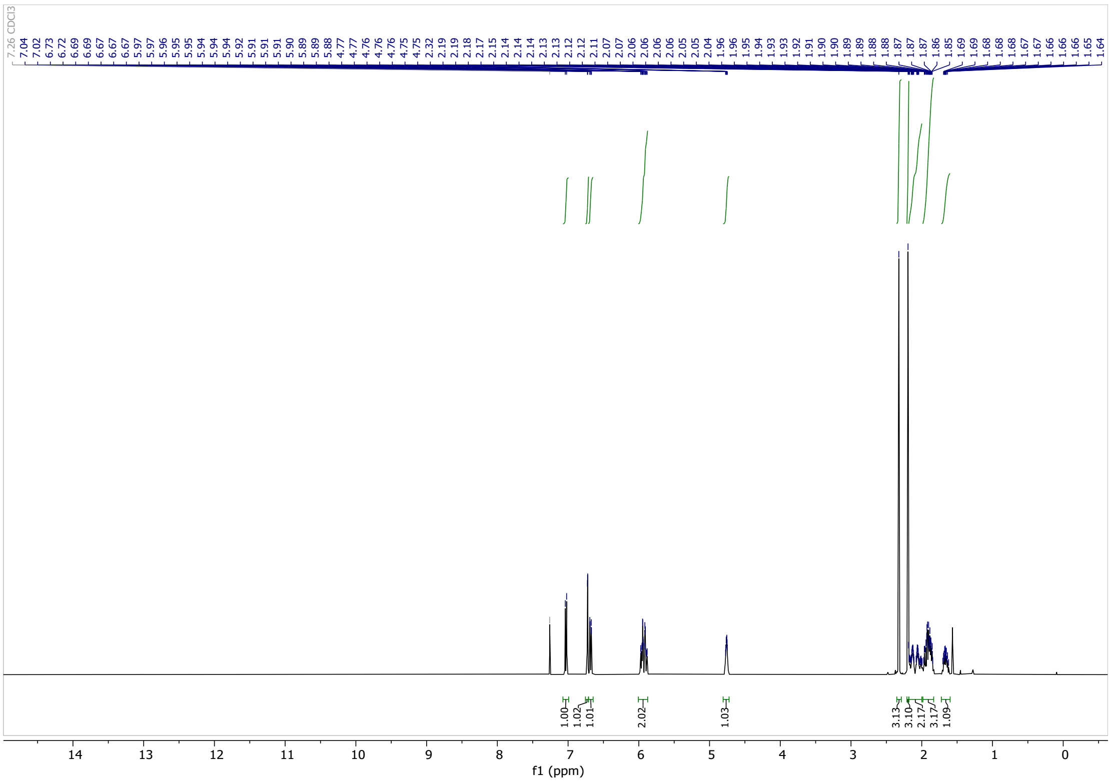

^{13}C NMR (101 MHz, $CDCl_3$)

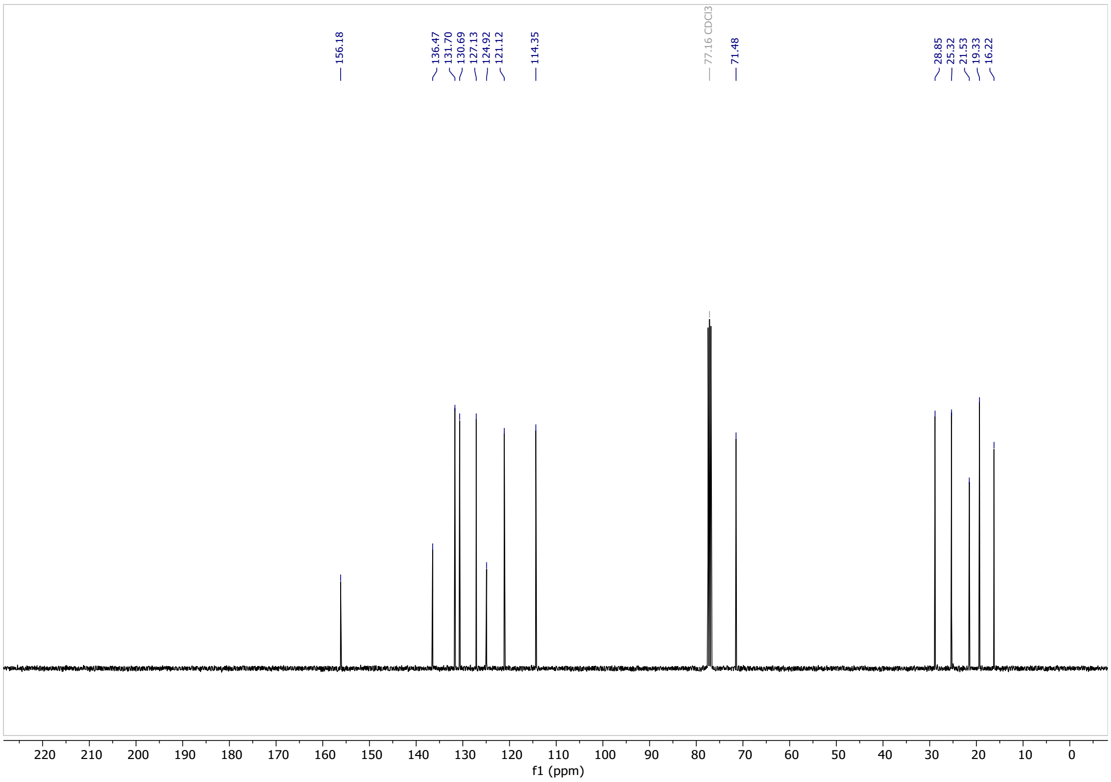

2-(Allyloxy)-1,4-dimethylbenzene (4c)

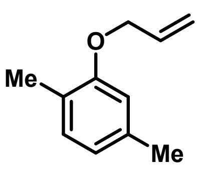

A 30 mL screw neck vial was charged with 2,5-dimethyl phenol (154 mg, 1.26 mmol, 1 equiv.), allyl bromide (183 mg, 1.51 mmol, 1.2 equiv.) and K_2CO_3 (279 mg, 2.02 mmol, 1.6 equiv.) in 6.2 mL acetone (0.2 M). The vial was placed in a heating block and stirred at 80 °C for 2 hours at which point TLC (petroleum ether/ ethyl acetate 10:1) confirmed full consumption of starting material. The reaction was quenched by addition of H_2O, and the aqueous layer was extracted with DCM three times. The combined organic layer was dried over $MgSO_4$, filtered and concentrated in vacuo. The crude material was purified by column chromatography (petroleum ether/ethyl acetate 40:1) to provide the desired product **4c** as colorless oil in 49% yield (101 mg, 0.62 mmol).

^{1}H NMR (400 MHz, $CDCl_3$) δ 7.04 (dd, *J* = 7.5, 3.2 Hz, 1H), 6.70 (dd, *J* = 7.7, 3.0 Hz, 1H), 6.68 – 6.65 (m, 1H), 6.18 – 6.03 (m, 1H), 5.51 – 5.42 (m, 1H), 5.34 – 5.25 (m, 1H), 4.59 – 4.51 (m, 2H), 2.34 (s, 3H), 2.24 (s, 3H).

^{13}C NMR (101 MHz, $CDCl_3$) δ 156.7, 136.6, 133.9, 130.6, 123.9, 121.1, 116.9, 112.5, 68.8, 21.5, 16.0.

^{1}H NMR (400 MHz, $CDCl_3$)

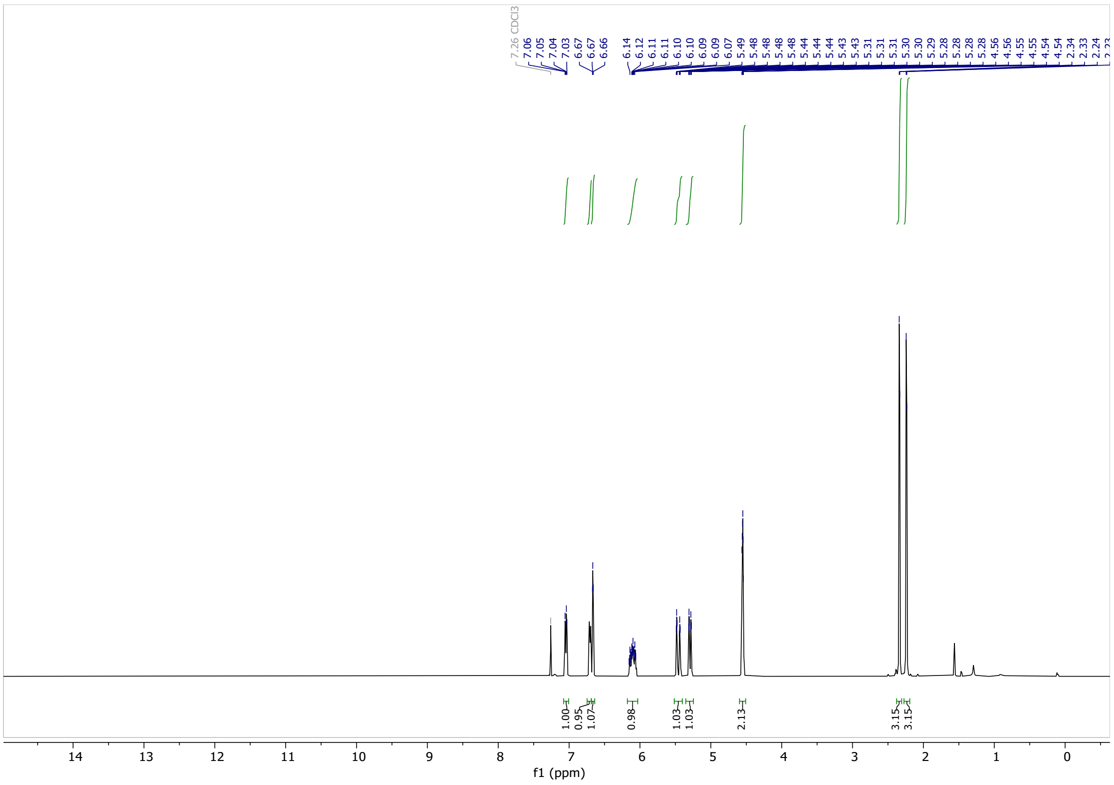

^{13}C NMR (101 MHz, $CDCl_3$)

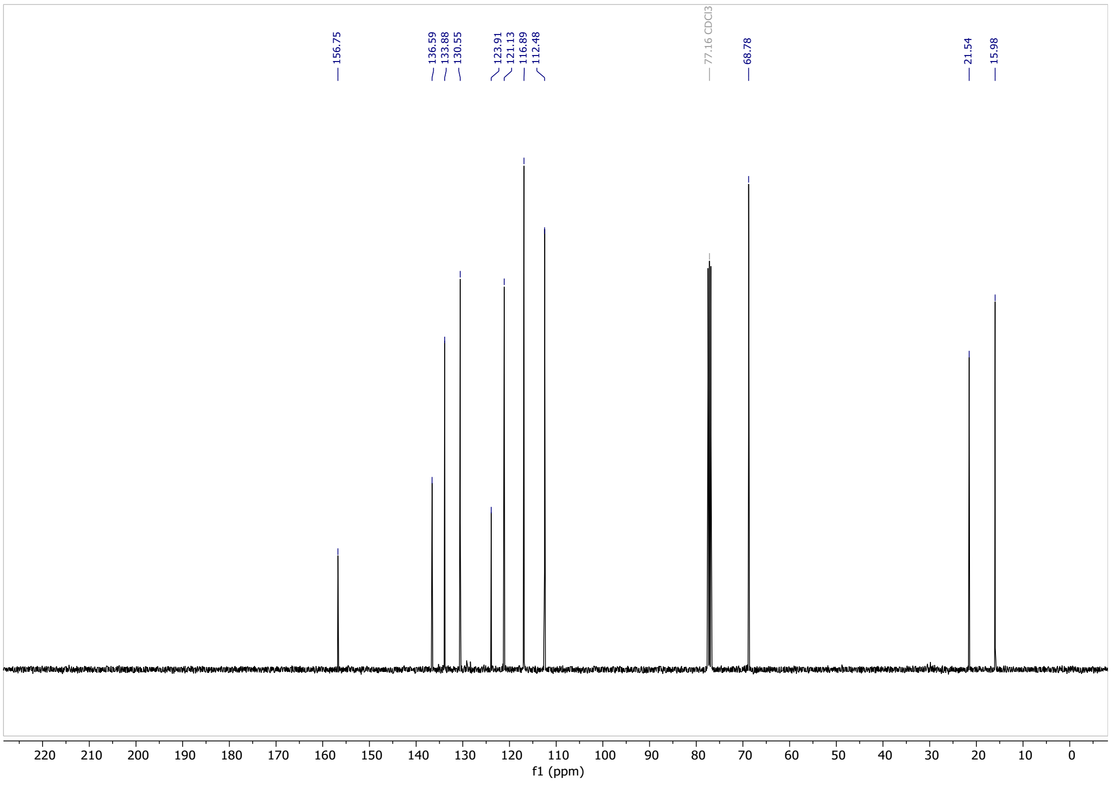

2-(But-3-yn-2-yloxy)-1,4-dimethylbenzene (E-1)

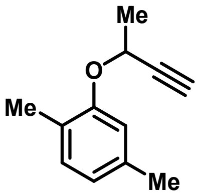

A flame-dried 250 mL Schlenk flask was charged with 2,5-dimethyl phenol (4.06 g, 33.2 mmol, 1.2 equiv.) and PPh_3 (10.9 g, 41.5 mmol, 1.5 equiv.) dissolved in 100 mL dry toluene. The colorless mixture was cooled by an ice-bath before but-3-yn-2-ol (2.25 mL, 27.7 mmol, 1 equiv.) was added. After 10 minutes, DIAD (7.54 mL, 36.0 mmol, 1.3 equiv.) was added causing a color change to brownish orange. The mixture was stirred and allowed to reach room temperature overnight. The next day, the reaction mixture was cooled by an ice-bath and 6 % H_2O_2 solution was added (100 mL) and the reaction mixture was stirred for 5 minutes. Layers were separated and the organic layer was washed with 6 % H_2O_2 solution (2x 50 mL), followed by H_2O and Brine. It was dried over $MgSO_4$ and concentrated in vacuo. The crude material was subjected to flash chromatography (80 g silica, petroleum ether/ethyl acetate 40:1) and the desired product **E-1** was obtained in sufficient purity as orange oil in 49 % unimproved yield (2.38 g, 13.7 mmol).

^{1}H NMR (400 MHz, $CDCl_3$) δ 7.05 – 7.01 (m, 1H), 6.86 (d, *J* = 1.6 Hz, 1H), 6.72 (dd, *J* = 7.6, 1.6 Hz, 1H), 4.84 (qd, *J* = 6.6, 2.0 Hz, 1H), 2.47 (d, *J* = 2.0 Hz, 1H), 2.34 (s, 3H), 2.20 (s, 3H), 1.68 (d, *J* = 6.6 Hz, 3H).

^{13}C NMR (101 MHz, $CDCl_3$) δ 155.6, 136.5, 130.6, 124.5, 122.0, 114.4, 83.6, 73.6, 63.9, 22.5, 21.5, 16.0.

HRMS (ESI): exact mass calculated for $C_{12}H_{15}O^+$ [$(M + H)^+$], 175.1117; found 175.1104.

^1H NMR (400 MHz, $CDCl_3$)

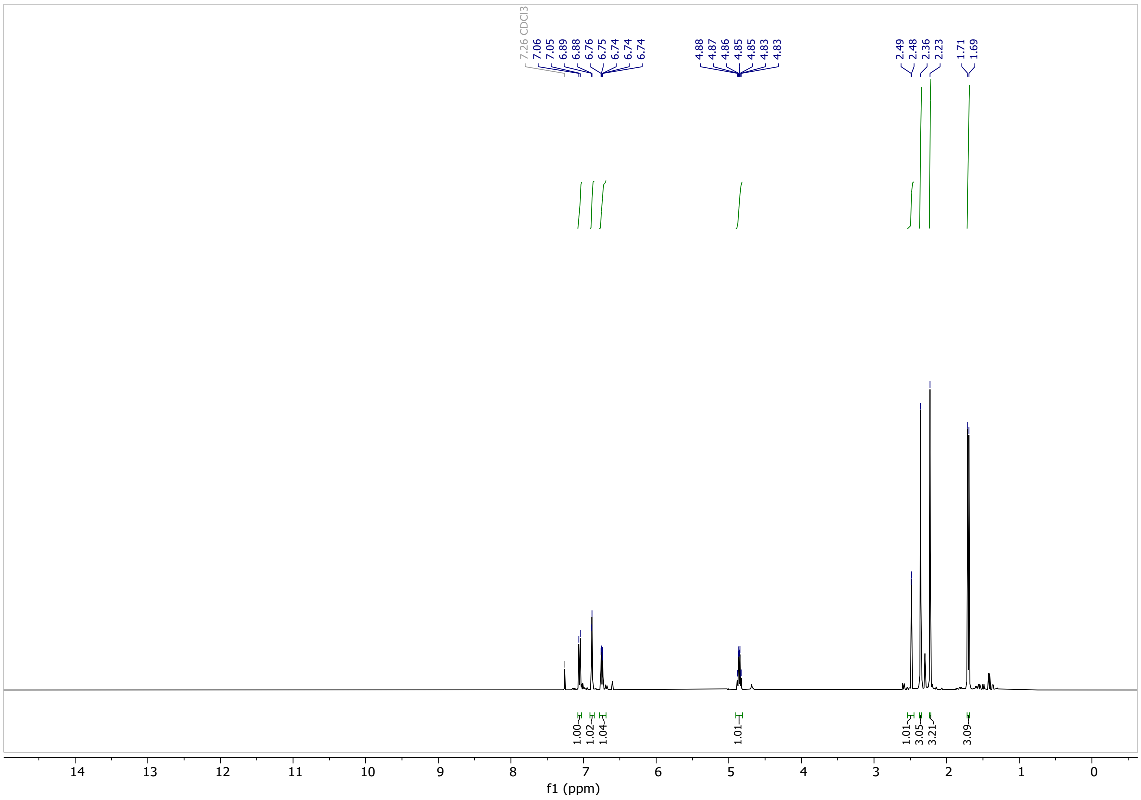

^{13}C NMR (101 MHz, $CDCl_3$)

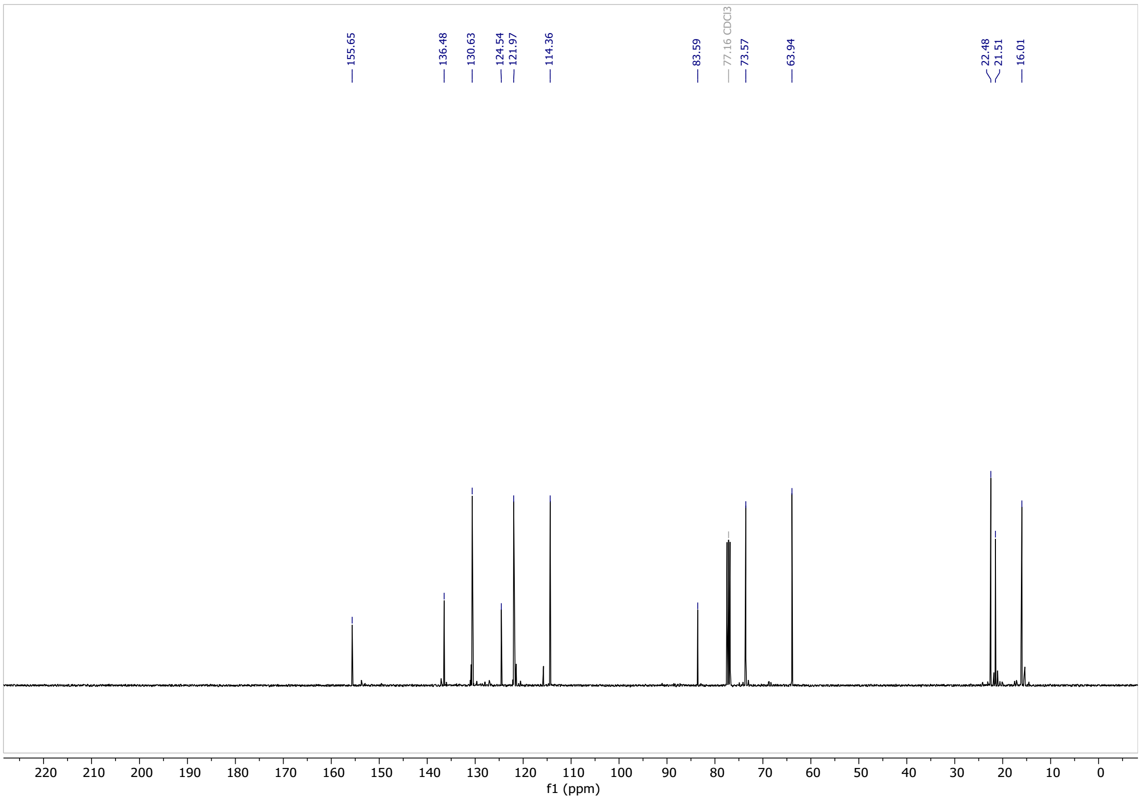

2-(But-3-en-2-yloxy)-1,4-dimethylbenzene (4d)

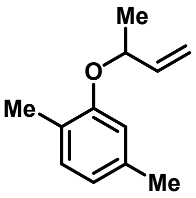

A flame-dried 50 mL Schlenk flask was charged with compound **E-1** (350 mg, 2.01 mmol, 1 equiv.) and quinoline (0.17 mL, 1.41 mmol, 0.7 equiv.) in 20 mL dry hexane. The pale-yellow solution was degassed by vacuum/Ar (10 cycles) before Lindlar's catalyst (Pd 5% on $CaCO_3$ – poisoned with lead, 428 mg, 0.20 mmol, 10 mol %) was added. The atmosphere was exchanged to H_2 by vacuum/H_2 (10 cycles). After 1 hour, TLC (petroleum ether/ ethyl acetate 10:1) confirmed full conversion of starting material The atmosphere was exchanged to Argon by vacuum/Ar (10 cycles) and the reaction mixture was filtered through a short pad of silica. The crude material was purified by column chromatography (petroleum ether/ ethyl acetate 40:1) and the desired product **4d** was obtained as pale-yellow oil in 54 % unoptimized yield (190 mg, 1.08 mmol).

^{1}H NMR (400 MHz, $CDCl_3$) δ 7.01 (d, *J* = 7.9 Hz, 1H), 6.69 – 6.62 (m, 2H), 5.94 (ddd, *J* = 17.2, 10.5, 5.6 Hz, 1H), 5.27 (dt, *J* = 17.4, 1.4 Hz, 1H), 5.15 (dt, *J* = 10.6, 1.3 Hz, 1H), 4.77 (tt, *J* = 6.4, 5.1 Hz, 1H), 2.29 (s, 3H), 2.20 (s, 3H), 1.43 (d, *J* = 6.4 Hz, 3H).

^{13}C NMR (101 MHz, $CDCl_3$) δ 156.2, 139.8, 136.4, 130.6, 124.6, 121.2, 115.2, 114.6, 74.9, 21.5, 21.5, 16.2.

^{1}H NMR (400 MHz, $CDCl_3$)

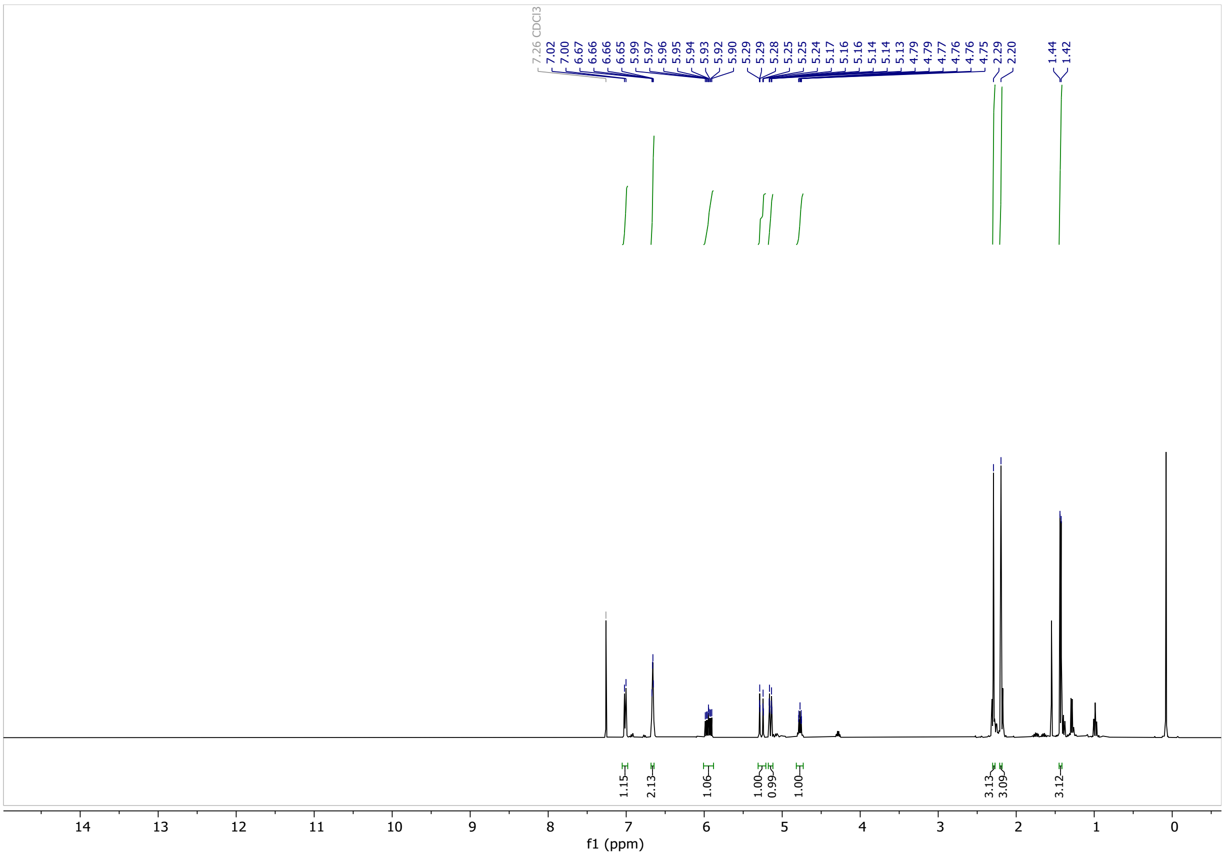

^{13}C NMR (101 MHz, $CDCl_3$)

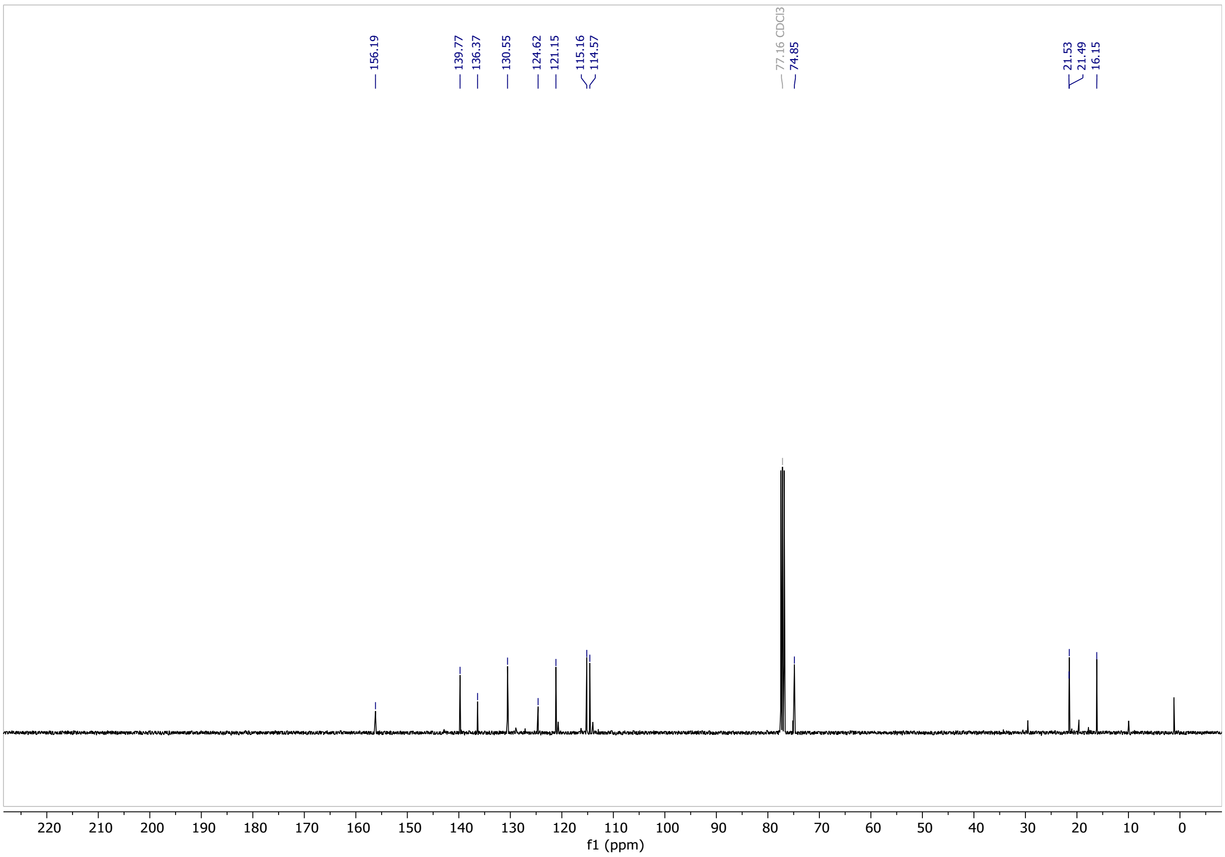

1,4-Dimethyl-2-(pent-3-yn-2-yloxy)benzene (4e)

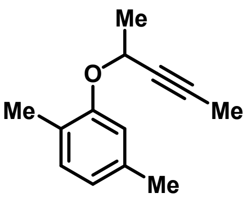

A flame-dried 50 mL Schlenk flask was charged with compound **E-1** (700 mg, 4.02 mmol, 1 equiv.) dissolved in 20 mL dry THF. The solution was cooled to -18 °C and the n-Butyllithium (2.5 M, 3.21 mL, 8.03 mmol, 2 equiv.) was added causing a color change from colorless to black. The reaction was kept at -18 °C for 1 hour before MeI (0.53 mL, 8.44 mmol, 2.1 equiv.) was added causing a color change to orange. The reaction mixture was allowed to warm to rt overnight. The next day, the reaction was quenched by addition of saturated aqueous NH_4Cl solution and extracted with Et_2O. The combined organic layer was dried over $MgSO_4$ and concentrated in vacuo. The crude material was purified by flash chromatography (petroleum ether/ ethyl acetate 40:1) and the desired product **4e** was obtained as colorless oil in 99 % yield (750 mg, 3.98 mmol).

^{1}H NMR (400 MHz, $CDCl_3$) δ 7.01 (dd, *J* = 7.5, 0.9 Hz, 1H), 6.88 – 6.84 (m, 1H), 6.70 (ddd, *J* = 7.4, 1.8, 0.9 Hz, 1H), 4.78 (qq, *J* = 6.4, 2.0 Hz, 1H), 2.33 (s, 3H), 2.19 (s, 3H), 1.83 (d, *J* = 2.0 Hz, 3H), 1.61 (d, *J* = 6.5 Hz, 3H).

^{13}C NMR (101 MHz, $CDCl_3$) δ 156.0, 136.4, 130.5, 124.5, 121.6, 114.4, 81.6, 79.2, 64.5, 22.9, 21.6, 16.1, 3.8.

^{1}H NMR (400 MHz, $CDCl_3$)

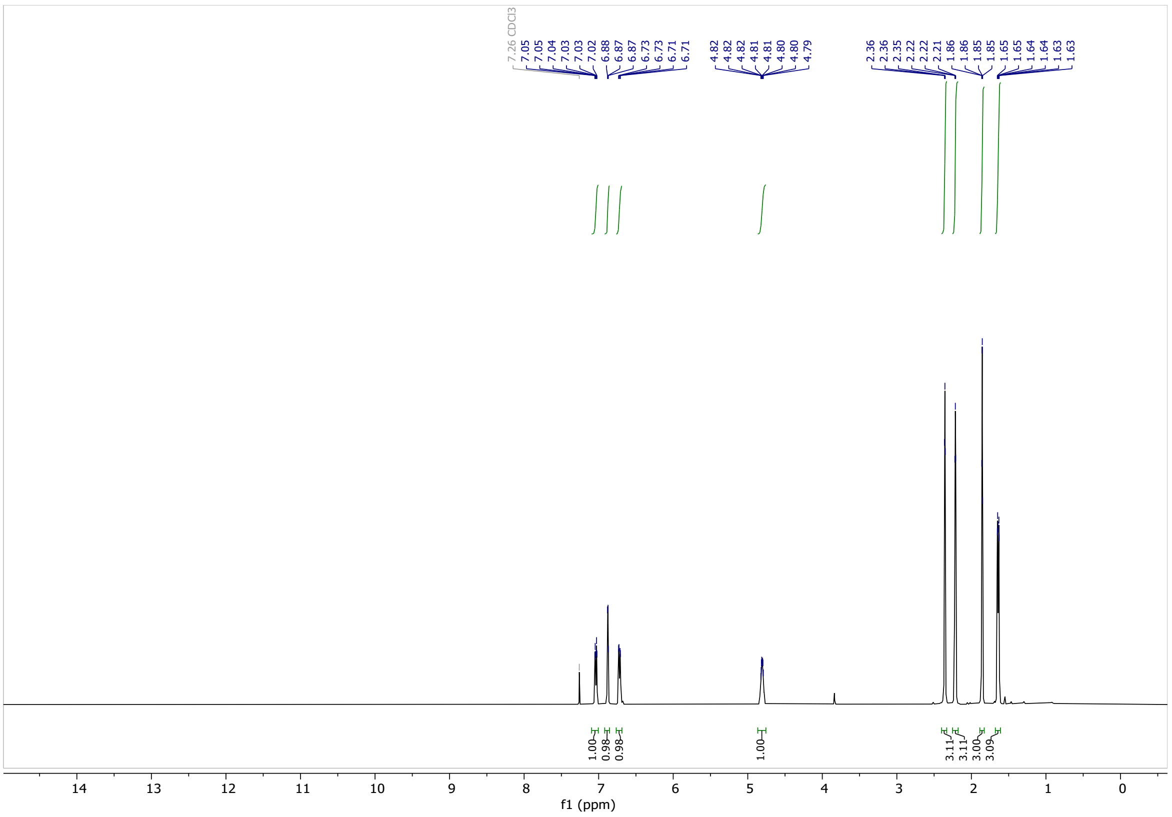

^{13}C NMR (101 MHz, $CDCl_3$)

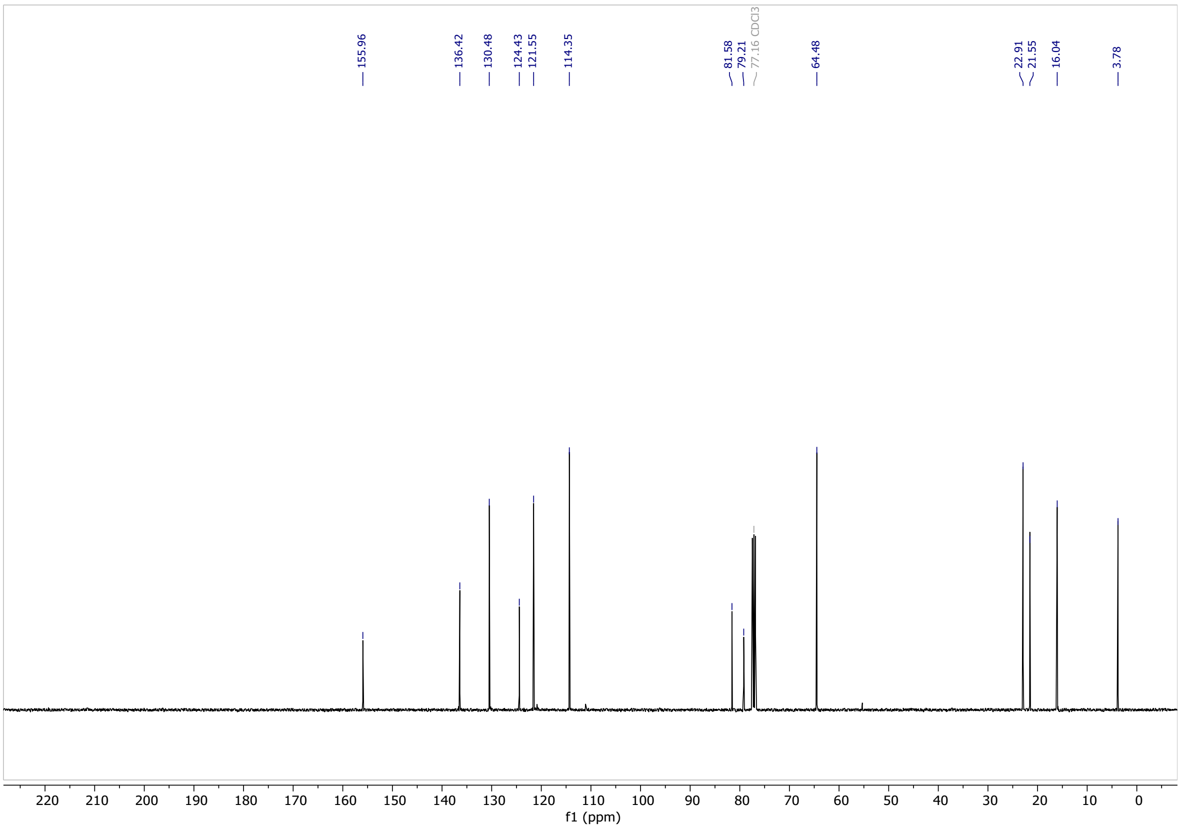

Chromatogram and Results

Instrument Method:	**Heptane_EtOH_99.9_0.1_0.5mlmin_25C_20min**	*B %:*	**0,0**
Column:	**OD**	*C %:*	**0,0**
Run Time (min):	**20,00**	*D %:*	**0,1**
Channel:	**UV_VIS_1**		
Wavelength:	**287,26**		

Chromatogram

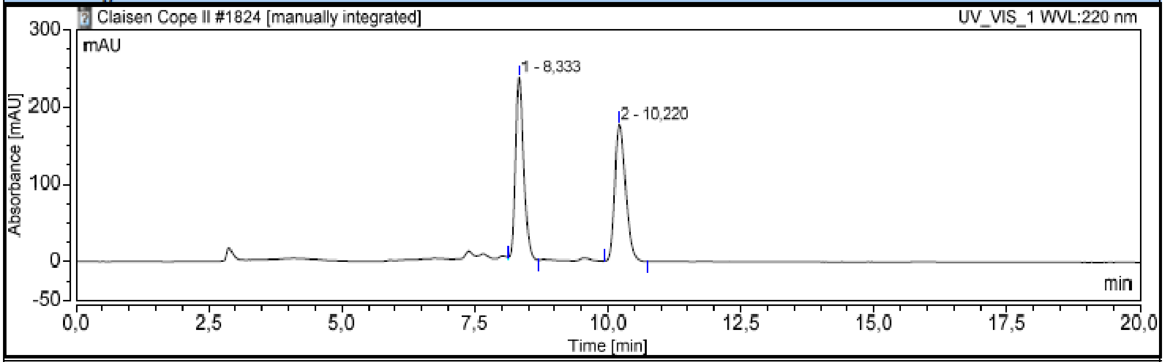

Integration Results

No.	Peak Name	Retention Time min	Area mAU*min	Height mAU	Relative Area %	Relative Height %
1		8,333	42,041	236,601	50,22	57,05
2		10,220	41,668	178,137	49,78	42,95
**Total:**			**83,709**	**414,739**	**100,00**	**100,00**

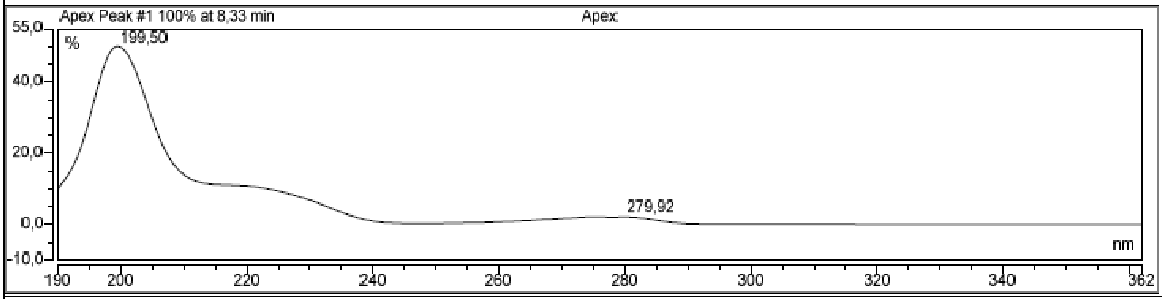

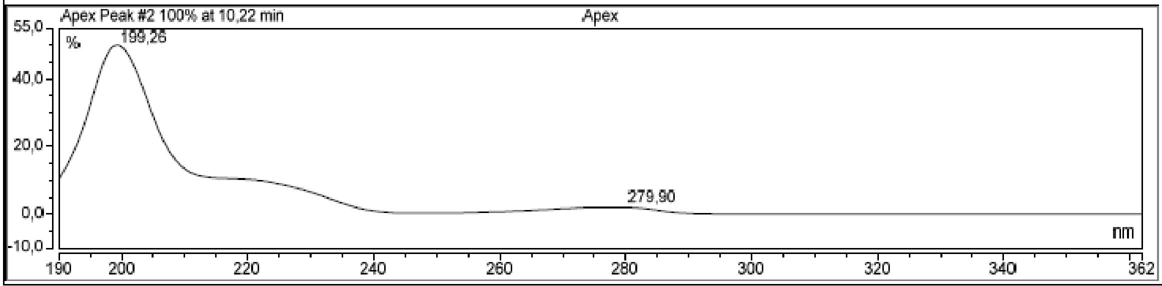

(*E*)-(Pent-3-en-2-yloxy)benzene (1e)

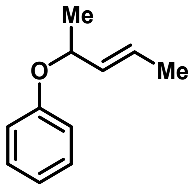

The title compound was synthesized from commercially available phenol (200 mg, 2.11 mmol) following *racemic* **general procedure A**. The crude material was purified by column chromatography (petroleum ether/ethyl acetate 40:1) to provide the desired product **1e** as colorless oil in 50% yield (171 mg, 1.05 mmol).

^{1}H NMR (400 MHz, $CDCl_3$) δ 7.28 – 7.21 (m, 2H), 6.95 – 6.84 (m, 3H), 5.77 – 5.66 (m, 1H), 5.54 (dddd, *J* = 15.5, 8.0, 2.8, 1.6 Hz, 1H), 4.76 (pd, *J* = 6.2, 1.1 Hz, 1H), 1.69 (ddt, *J* = 6.5, 1.7, 0.8 Hz, 3H), 1.40 (dd, *J* = 6.4, 0.9 Hz, 3H).

^{13}C NMR (101 MHz, $CDCl_3$) δ 158.2, 132.4, 129.4, 127.3, 120.6, 116.2, 74.4, 21.6, 17.8.

^1H NMR (400 MHz, $CDCl_3$)

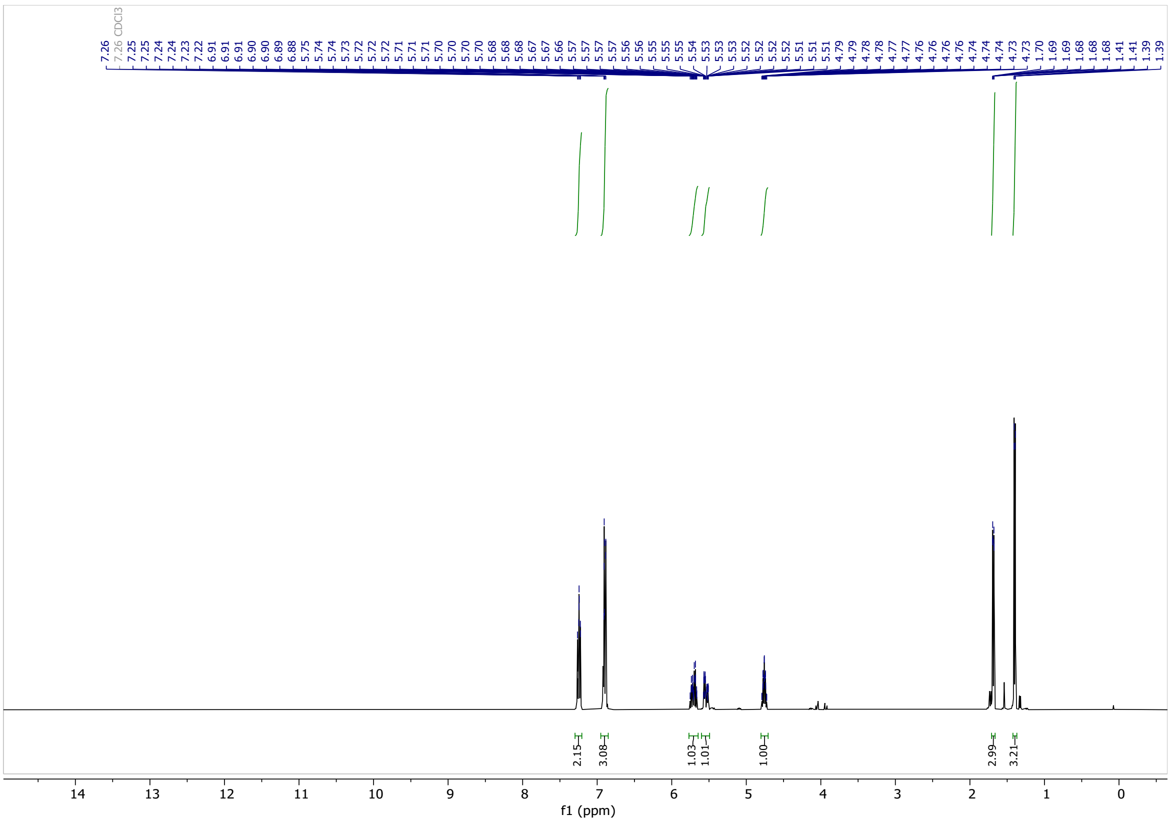

^{13}C NMR (101 MHz, $CDCl_3$)

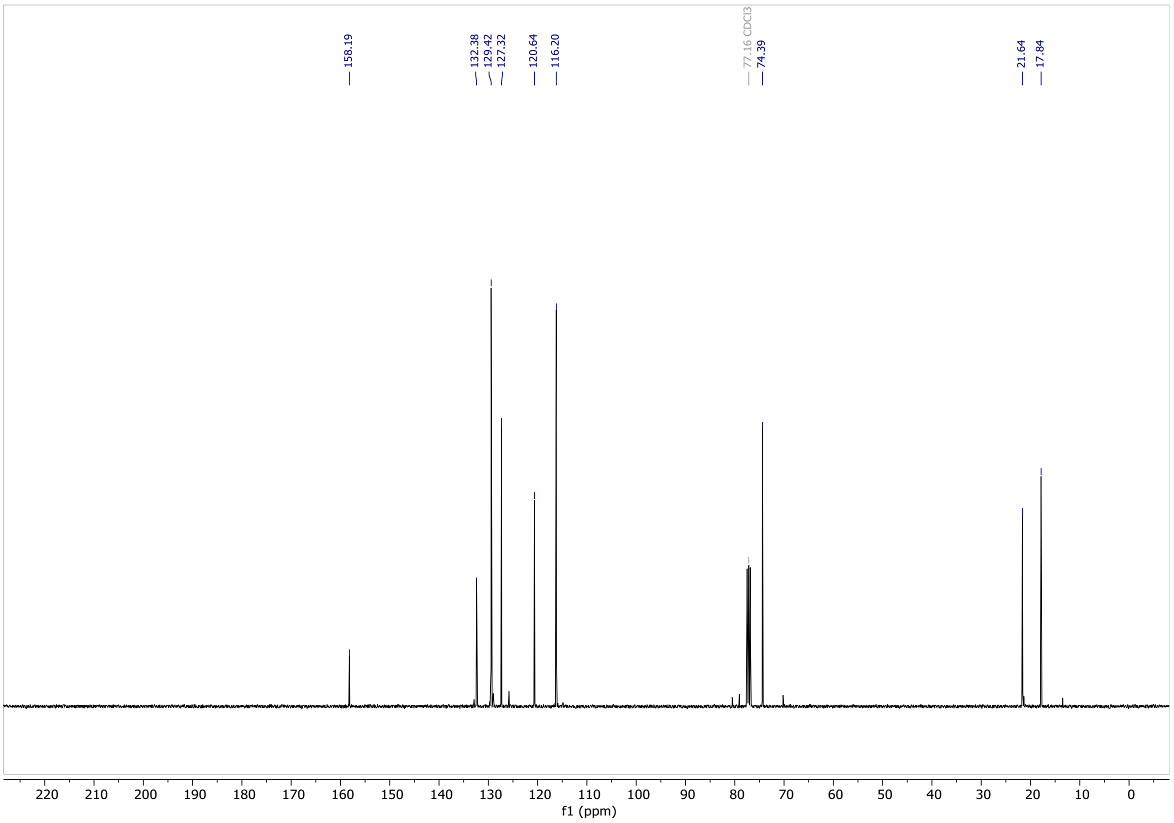

(*R,E*)-2,5-Dimethyl-4-(pent-3-en-2-yl)phenol (2f) & (*S,E*)-3,6-dimethyl-2-(pent-3-en-2-yl)phenol (3f)

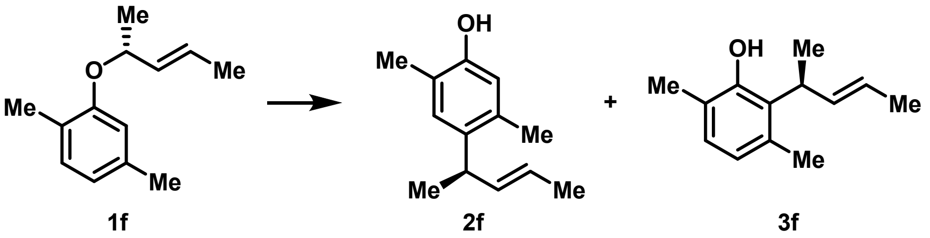

The title compounds were synthesized from **1f** (100 mg, 0.53 mmol) following **general procedure B**. The reaction was directly purified by column chromatography (petroleum ether/ethyl acetate 30:1) to provide the *para*-product **2f** as colorless oil in 65% yield (65 mg, 0.35 mmol) and the *ortho*-product **3f** as colorless oil in 35% yield (35 mg, 0.18 mmol).

(*R,E*)-2,5-Dimethyl-4-(pent-3-en-2-yl)phenol (2f)

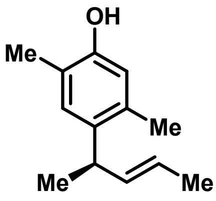

$[\alpha]^{20}$= - 38.32 (c 2.40, CH_2Cl_2)

^{1}H NMR (400 MHz, $CDCl_3$) δ 6.92 (s, 1H), 6.60 (s, 1H), 5.56 (ddq, *J* = 15.3, 6.3, 1.5 Hz, 1H), 5.49 – 5.32 (m, 1H), 4.63 (s, 1H), 3.53 (tt, *J* = 7.0, 5.7 Hz, 1H), 2.24 (d, *J* = 4.5 Hz, 6H), 1.67 (dt, *J* = 6.3, 1.4 Hz, 3H), 1.28 (d, *J* = 7.0 Hz, 3H).

^{13}C NMR (101 MHz, $CDCl_3$) δ 151.7, 136.7, 136.2, 134.4, 128.8, 123.3, 121.0, 116.9, 37.2, 21.1, 19.1, 18.1, 15.6.

HRMS (ESI): exact mass calculated for $C_{13}H_{17}O^-$ [(M - H)$^-$], 189.1285; found 189.1276.

87% *ee* (determined by chiral HPLC: Chiralcel® OJ-3 column, n-Heptane/EtOH = 99:1, 0.7 mL/min, λ = 287.3 nm, 25 °C), major enantiomer. t_r = 24.78 min, minor enantiomer. t_r = 26.14 min

^{1}H NMR (400 MHz, $CDCl_3$)

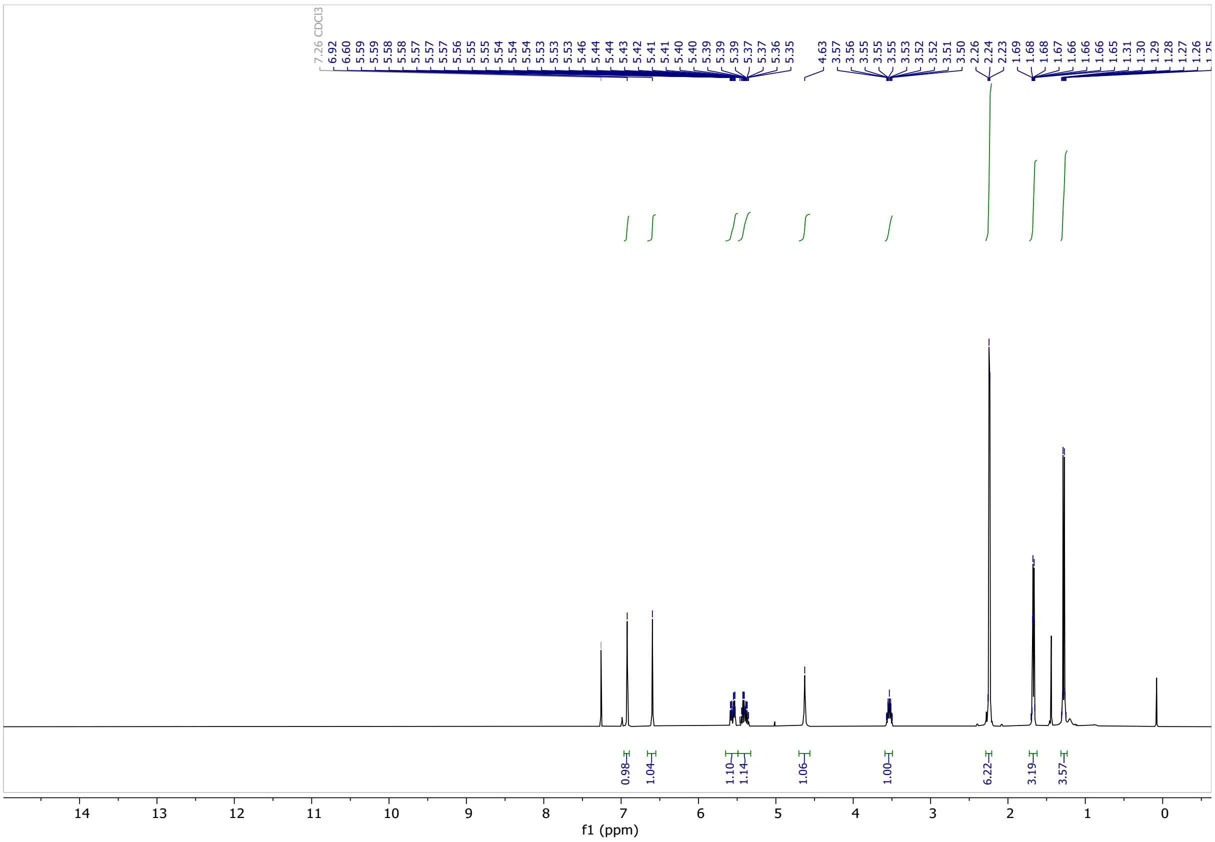

^{13}C NMR (101 MHz, $CDCl_3$)

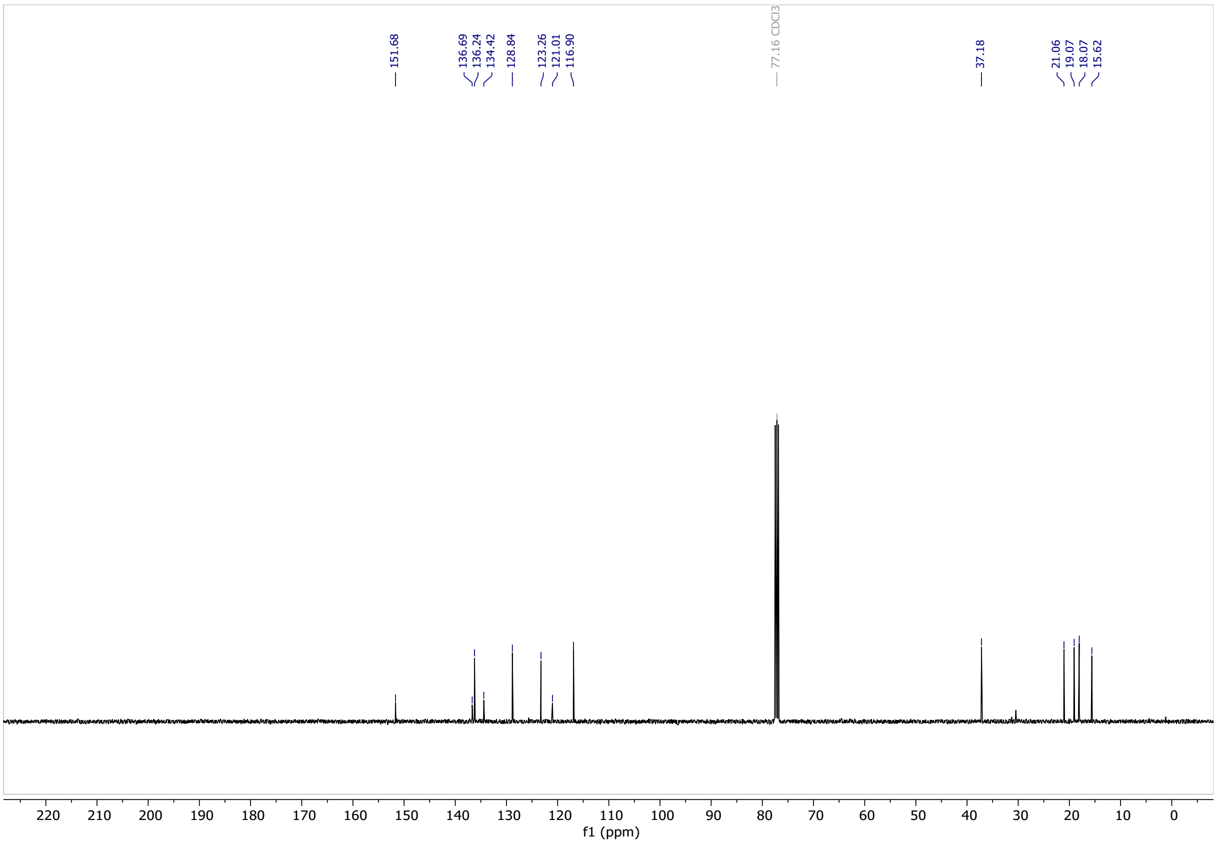

Chromatogram and Results

Instrument Method:	**Heptane_EtOH_99_1_0.7mlmin_25C_40min-MK**	*B %:*	**0,0**
Column:	**OJ3**	*C %:*	**0,0**
Run Time (min):	**40,00**	*D %:*	**1,0**
Channel:	**UV_VIS_1**		
Wavelength:	**287,26**		

Chromatogram

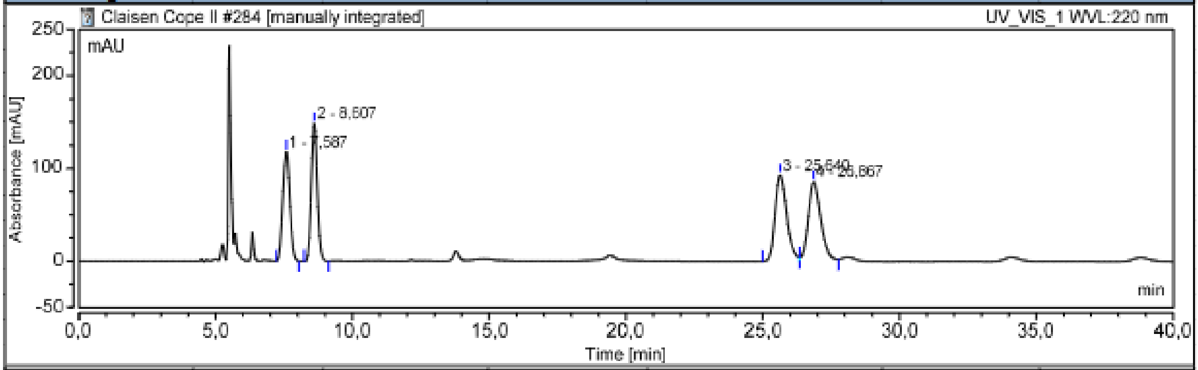

Integration Results

No.	Peak Name	Retention Time<br>min	Area<br>mAU*min	Height<br>mAU	Relative Area<br>%	Relative Height<br>%
1		7,587	35,718	118,017	22,00	26,58
2		8,607	37,187	148,370	22,91	33,42
3		25,640	45,574	93,091	28,07	20,97
4		26,867	43,861	84,508	27,02	19,03
**Total:**			**162,340**	**443,986**	**100,00**	**100,00**

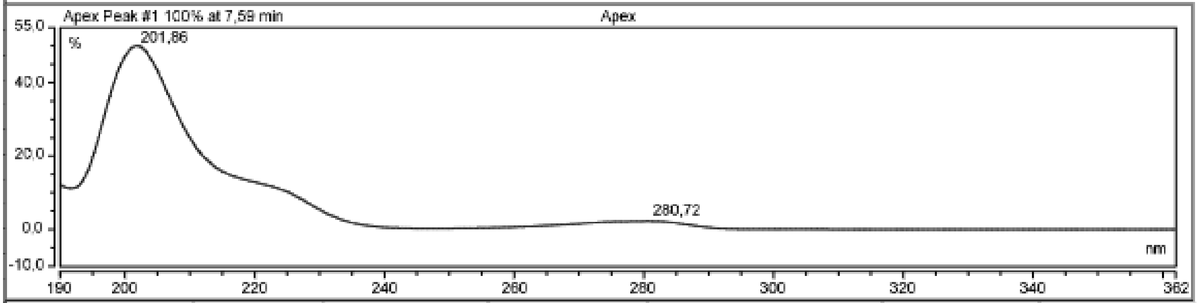

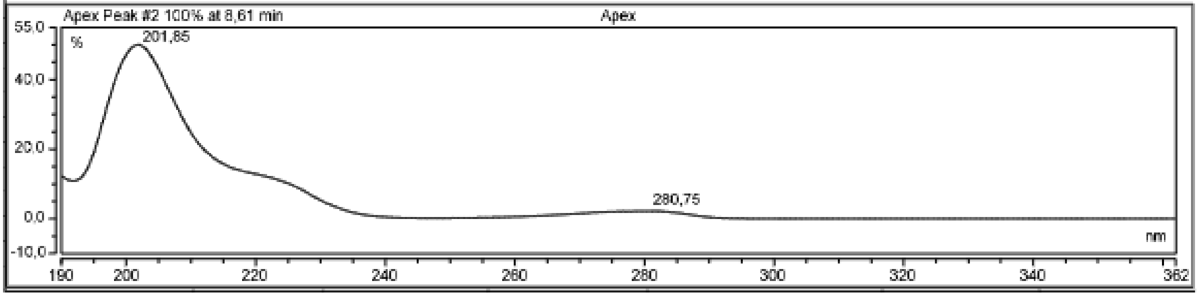

Chromatogram and Results

Instrument Method:	**Heptane_EtOH_99_1_0.7mlmin_25C_40min-MK**	*B %:*	**0,0**
Column:	**OJ3**	*C %:*	**0,0**
Run Time (min):	**40,00**	*D %:*	**1,0**
Channel:	**UV_VIS_1**		
Wavelength:	**287,26**		

Chromatogram

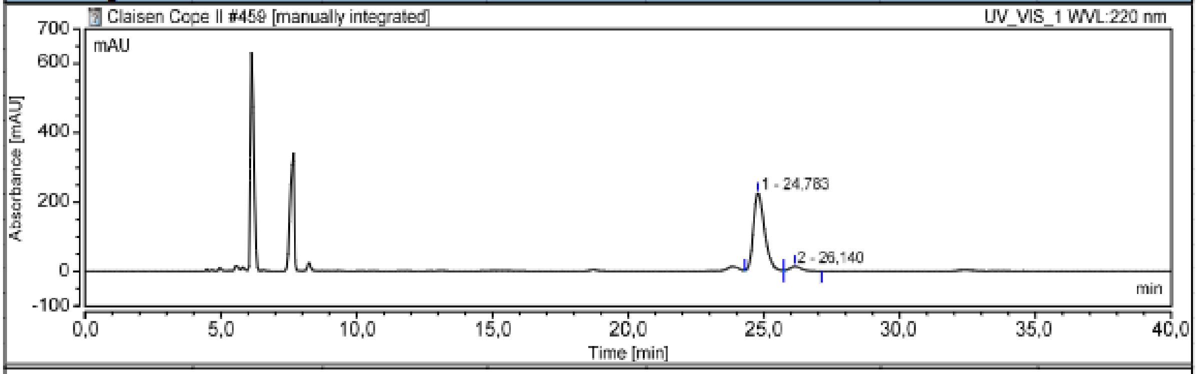

Integration Results

No.	Peak Name	Retention Time min	Area mAU*min	Height mAU	Relative Area %	Relative Height %
1		24,783	107,995	225,052	93,71	94,07
2		26,140	7,250	14,199	6,29	5,93
**Total:**			**115,246**	**239,251**	**100,00**	**100,00**

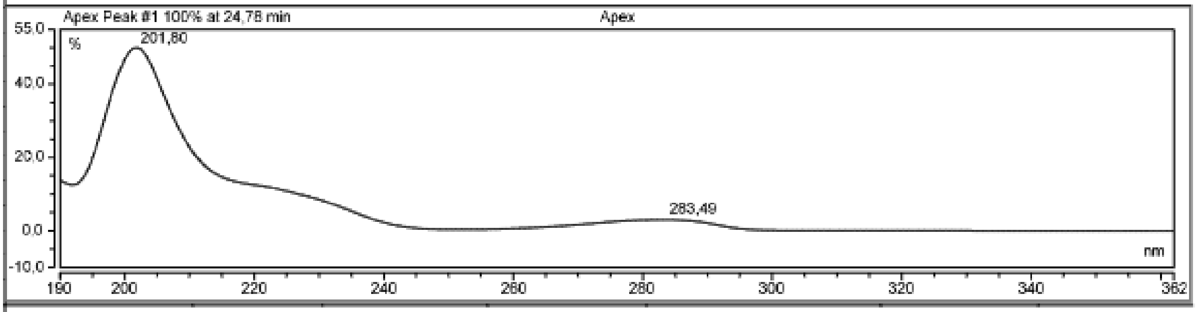

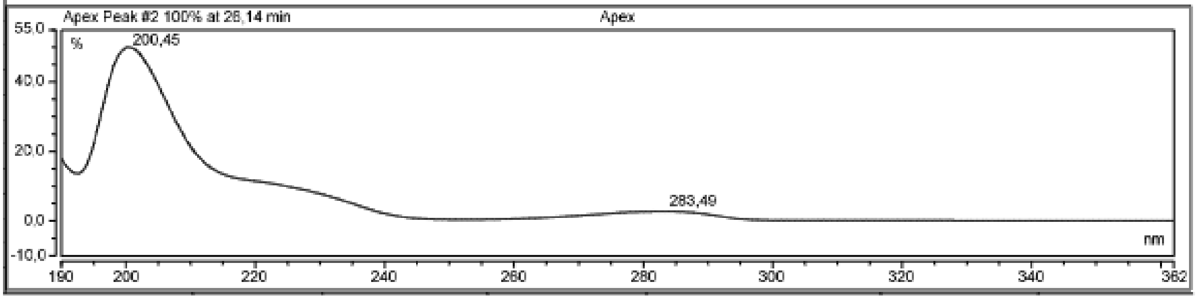

(*S,E*)-3,6-Dimethyl-2-(pent-3-en-2-yl)phenol (3f)

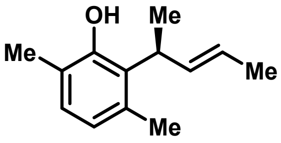

$[\alpha]^{20}$= - 15.30 (c 2.50, CH_2Cl_2).

^{1}H NMR (400 MHz, $CDCl_3$) δ 6.89 (d, *J* = 7.6 Hz, 1H), 6.65 (d, *J* = 7.5 Hz, 1H), 6.01 – 5.88 (m, 2H), 5.83 (dqd, *J* = 15.9, 6.1, 2.2 Hz, 1H), 3.88 – 3.74 (m, 1H), 2.29 (s, 3H), 2.17 (s, 3H), 1.80 (ddd, *J* = 6.1, 2.4, 1.4 Hz, 3H), 1.40 – 1.33 (m, 3H).

^{13}C NMR (101 MHz, $CDCl_3$) δ 153.6, 135.0, 134.0, 128.7, 128.0, 126.7, 123.7, 122.2, 35.0, 20.3, 18.2, 16.5, 16.0.

HRMS (ESI): exact mass calculated for $C_{13}H_{17}O^-$ [(M - H)$^-$], 189.1285; found 189.1281.

88% *ee* (determined by chiral HPLC: Chiralcel® OJ-3 column, n-Heptane/EtOH = 99:1, 0.7 mL/min, λ = 287.3 nm, 25 °C), major enantiomer. t_r = 7.45 min, minor enantiomer. t_r = 8.38 min

^{1}H NMR (400 MHz, $CDCl_3$)

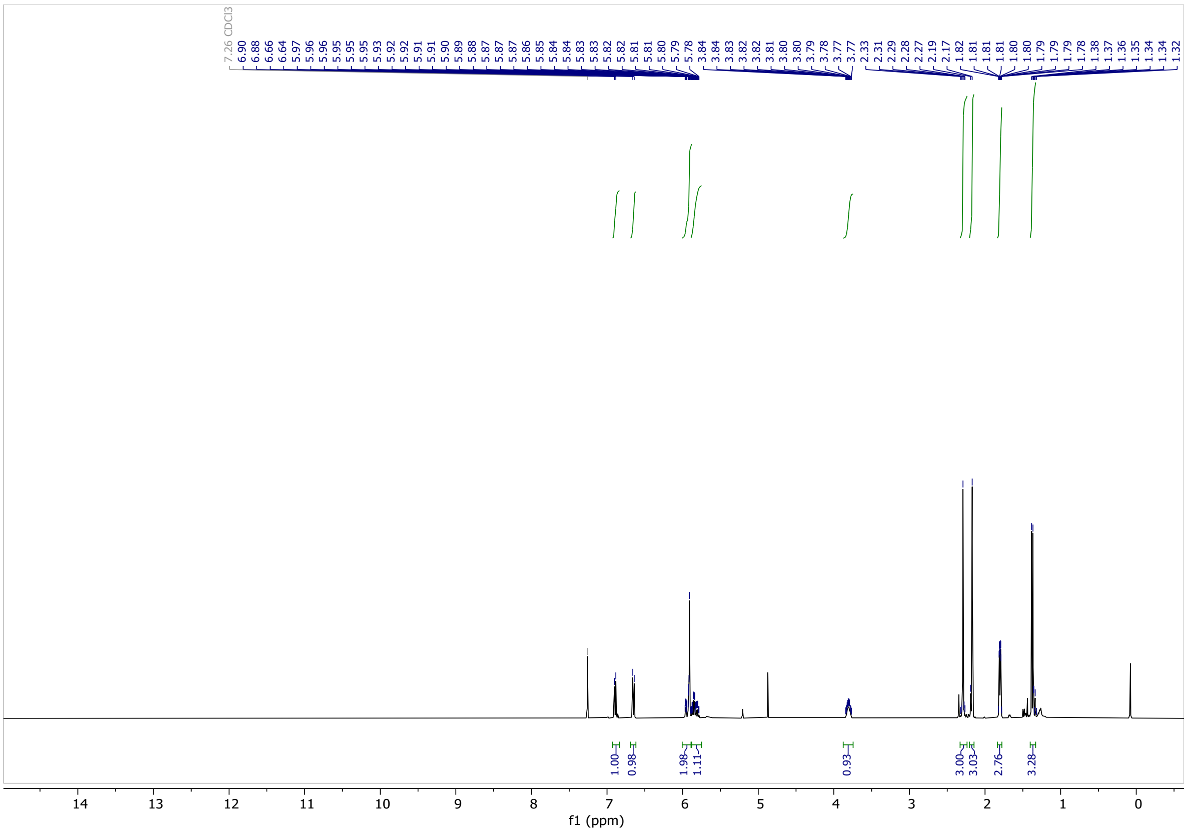

^{13}C NMR (101 MHz, $CDCl_3$)

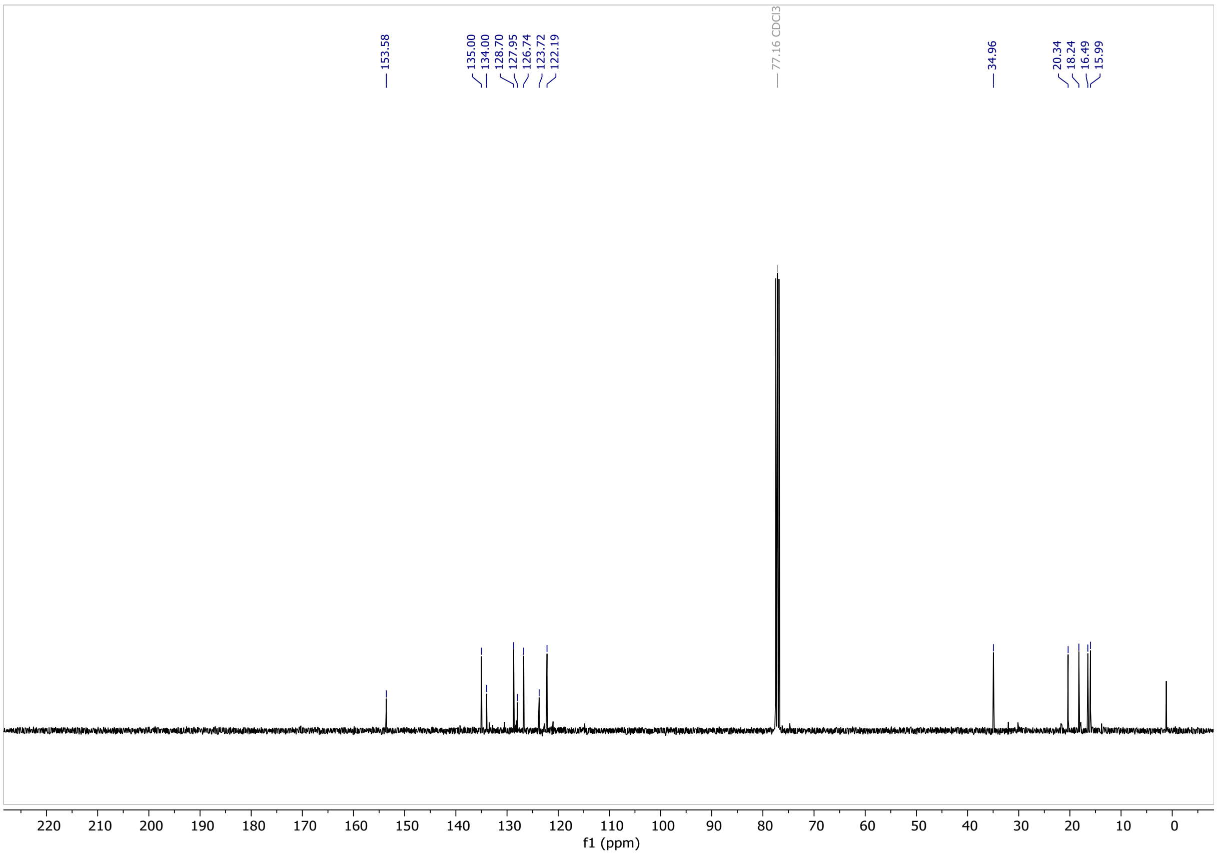

Chromatogram and Results

Instrument Method:	**Heptane_EtOH_99_1_0.7mlmin_25C_15min-MK**	*B %:*	**0,0**
Column:	**OJ3**	*C %:*	**0,0**
Run Time (min):	**15,00**	*D %:*	**1,0**
Channel:	**UV_VIS_1**		
Wavelength:	**287,26**		

Chromatogram

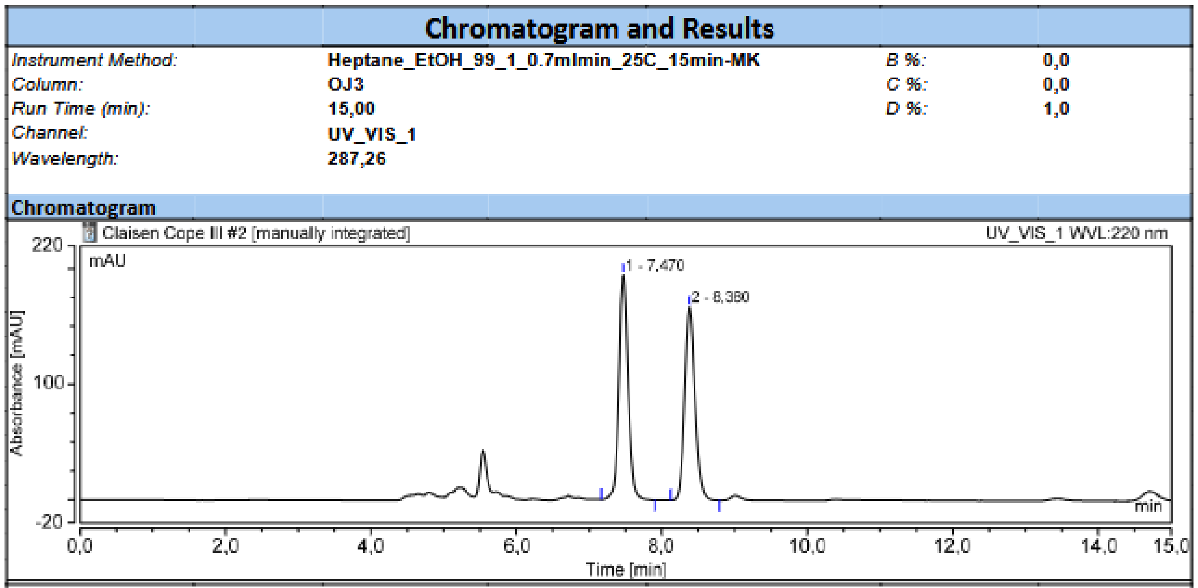

Integration Results

No.	Peak Name	Retention Time<br>min	Area<br>mAU*min	Height<br>mAU	Relative Area<br>%	Relative Height<br>%
1		7,470	28,258	194,564	50,80	53,77
2		8,380	27,373	167,272	49,20	46,23
**Total:**			**55,632**	**361,836**	**100,00**	**100,00**

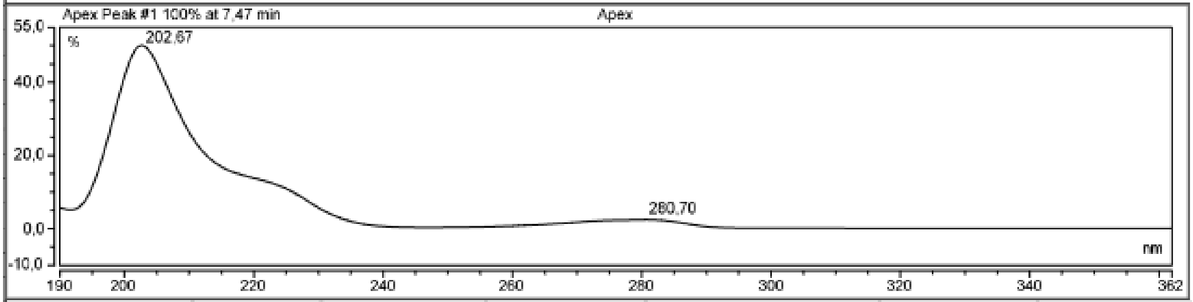

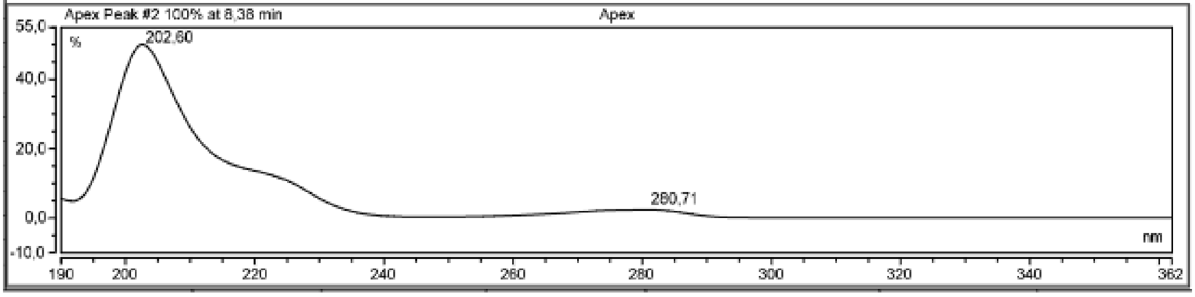

Chromatogram and Results

Instrument Method:	**Heptane_EtOH_99_1_0.7mlmin_25C_15min-MK**	*B %:*	**0,0**
Column:	**OJ3**	*C %:*	**0,0**
Run Time (min):	**15,00**	*D %:*	**1,0**
Channel:	**UV_VIS_1**		
Wavelength:	**287,26**		

Chromatogram

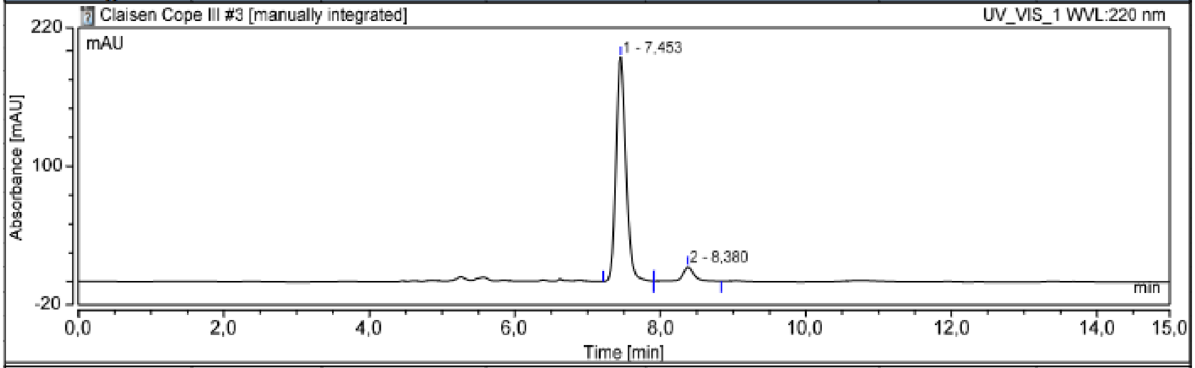

Integration Results

No.	Peak Name	Retention Time min	Area mAU*min	Height mAU	Relative Area %	Relative Height %
1		7,453	30,819	194,513	93,47	94,13
2		8,380	2,154	12,134	6,53	5,87
**Total:**			**32,973**	**206,647**	**100,00**	**100,00**

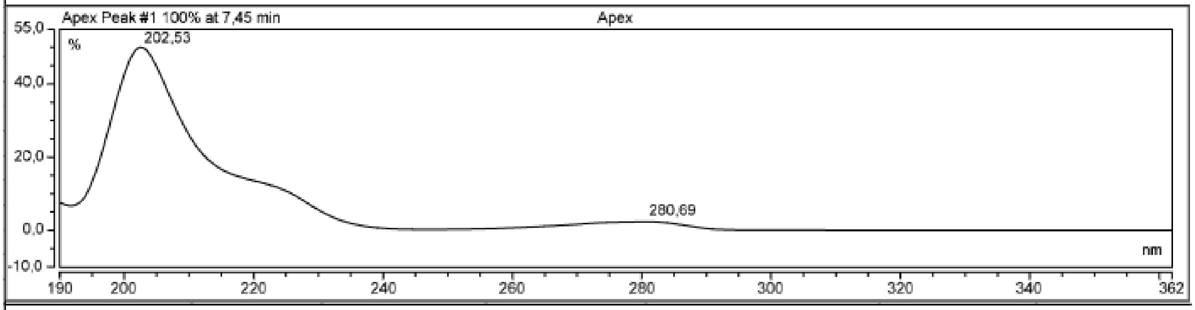

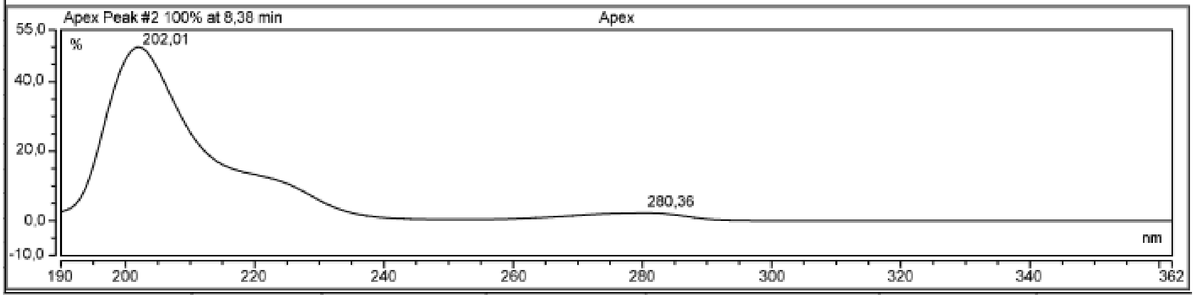

(*R,E*)-2,3-Dimethyl-4-(pent-3-en-2-yl)phenol (2g) & (*S,E*)-2,3-dimethyl-6-(pent-3-en-2-yl)phenol (3g)

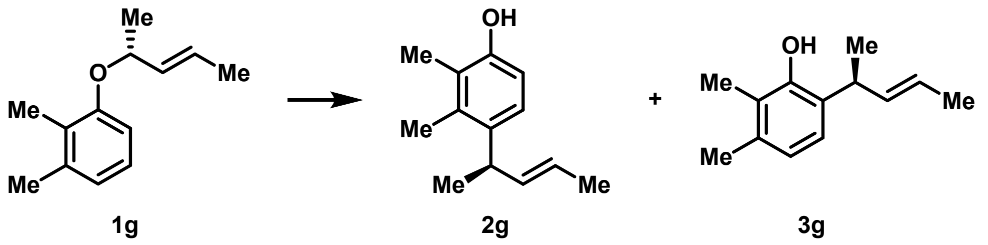

The title compounds were synthesized from **1g** (110 mg, 0.58 mmol) following **general procedure B**. The reaction was directly purified by column chromatography (petroleum ether/ethyl acetate 30:1) to provide the *para*-product **2g** as colorless oil in 22% yield (24 mg, 0.13 mmol) and the *ortho*-product **3g** as colorless oil in 78% yield (86 mg, 0.45 mmol).

(*R,E*)-2,3-Dimethyl-4-(pent-3-en-2-yl)phenol (2g)

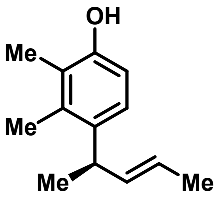

$[\alpha]^{20}$= - 418.34 (c 1.15, CH_2Cl_2).

^{1}H NMR (400 MHz, $CDCl_3$) δ 6.95 (d, *J* = 8.3 Hz, 1H), 6.69 (d, *J* = 8.3 Hz, 1H), 5.64 – 5.47 (m, 1H), 5.47 – 5.30 (m, 1H), 4.87 (s, 1H), 3.63 (p, *J* = 6.9 Hz, 1H), 2.25 (s, 6H), 1.67 (dt, *J* = 6.4, 1.5 Hz, 3H), 1.29 (d, *J* = 7.0 Hz, 3H).

^{13}C NMR (101 MHz, $CDCl_3$) δ 151.8, 136.9, 136.4, 135.9, 124.4, 123.4, 122.8, 112.6, 37.8, 21.2, 18.1, 15.3, 12.4.

HRMS (ESI): exact mass calculated for $C_{13}H_{17}O^-$ [(M - H)$^-$], 189.1285; found 189.1281.

85% *ee* (determined by chiral HPLC: Chiralcel® OJ-3 column, n-Hexane/EtOH = 98:2, 0.7 mL/min, λ = 287.3 nm, 25 °C), major enantiomer. t_r = 21.62 min, minor enantiomer. t_r = 23.02 min.

^{1}H NMR (400 MHz, $CDCl_3$)

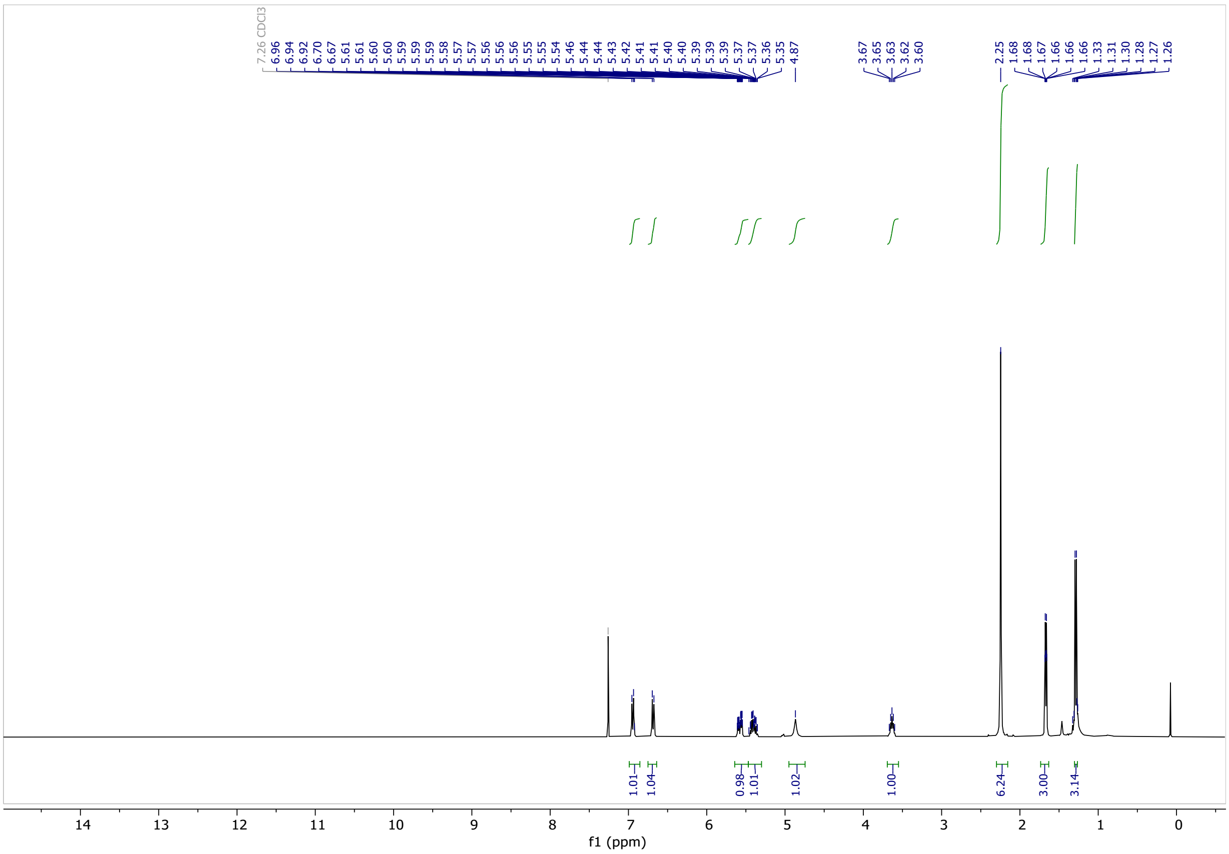

^{13}C NMR (101 MHz, $CDCl_3$)

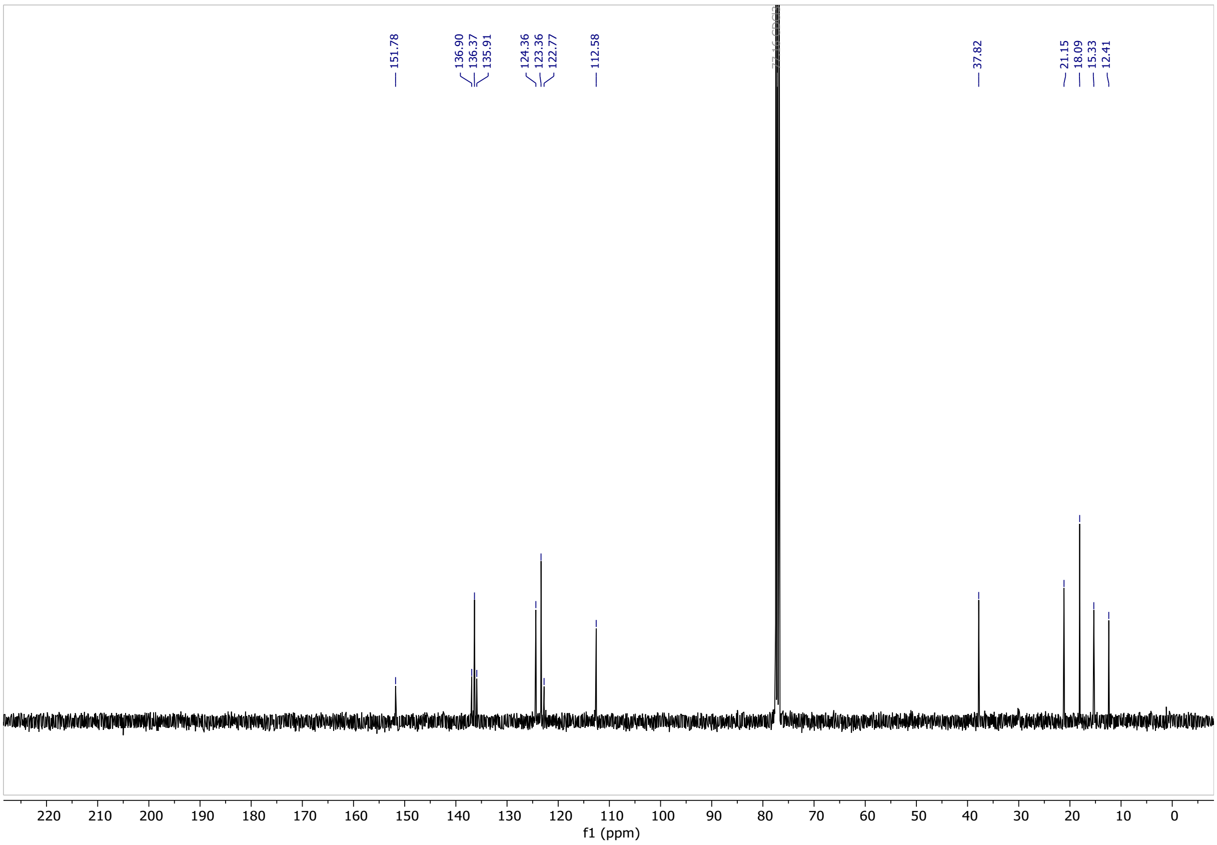
151.78
136.90
136.37
135.91
124.36
123.36
122.77
112.58
37.82
21.15
18.09
15.33
12.41
220 210 200 190 180 170 160 150 140 130 120 110 100 90 80 70 60 50 40 30 20 10 0
f1 (ppm)

Chromatogram and Results

Instrument Method:	**Hexane_EtOH_98_2_0.7mlmin_25C_45min-MK**	*B %:*	**0,0**
Column:	**OJ3**	*C %:*	**98,0**
Run Time (min):	**45,00**	*D %:*	**2,0**
Channel:	**UV_VIS_1**		
Wavelength:	**287,26**		

Chromatogram

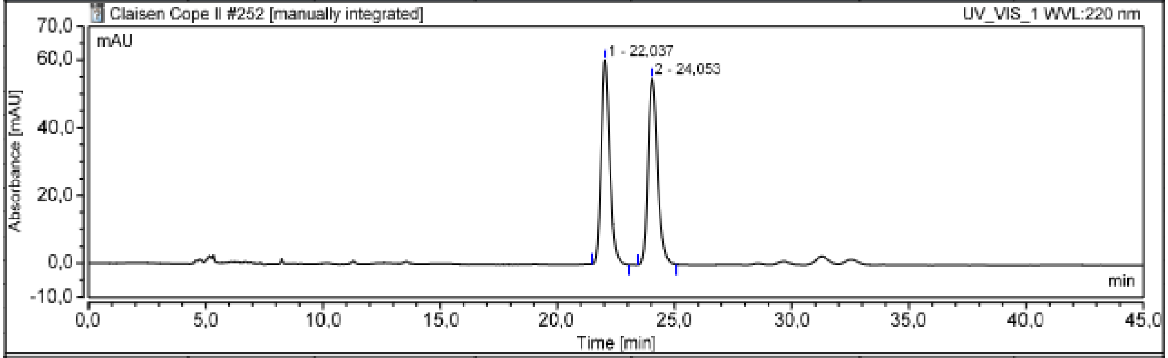

Integration Results

No.	Peak Name	Retention Time min	Area mAU*min	Height mAU	Relative Area %	Relative Height %
1		22,037	25,494	60,132	50,00	52,33
2		24,053	25,497	54,779	50,00	47,67
**Total:**			**50,991**	**114,911**	**100,00**	**100,00**

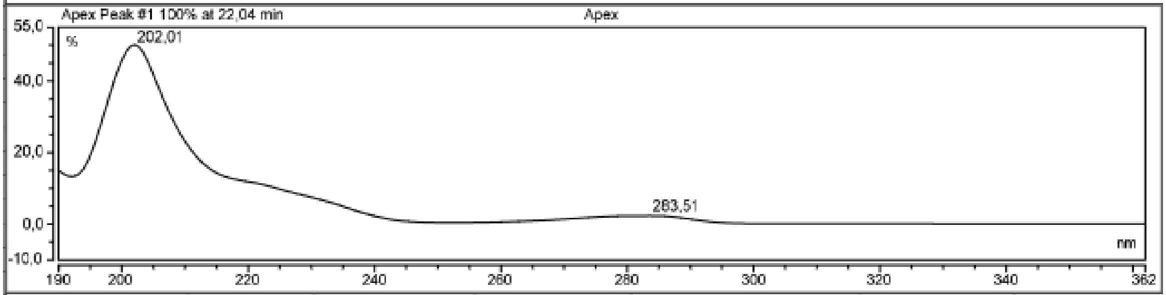

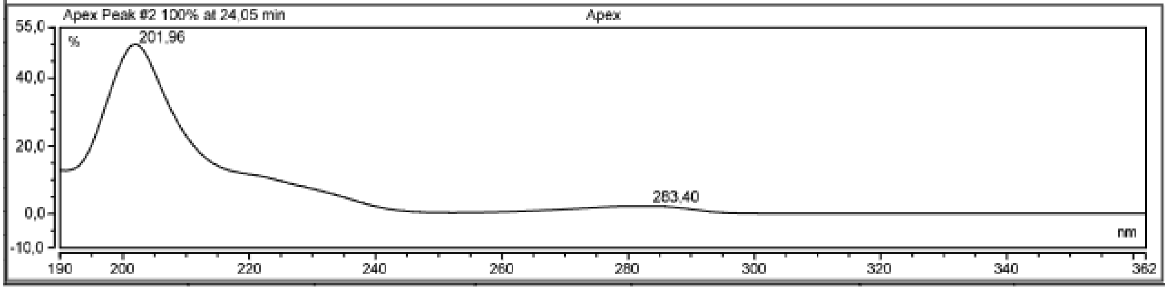

Chromatogram and Results

Instrument Method:	**Hexane_EtOH_98_2_0.7mlmin_25C_45min-MK**	*B %:*	**0,0**
Column:	**OJ3**	*C %:*	**98,0**
Run Time (min):	**45,00**	*D %:*	**2,0**
Channel:	**UV_VIS_1**		
Wavelength:	**287,26**		

Chromatogram

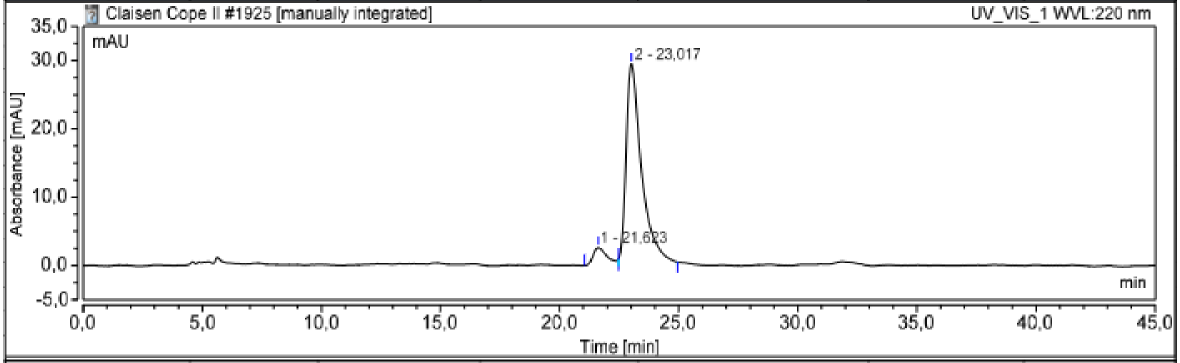

Integration Results

No.	Peak Name	Retention Time<br>min	Area<br>mAU*min	Height<br>mAU	Relative Area<br>%	Relative Height<br>%
1		21,623	1,786	2,600	7,30	8,10
2		23,017	22,690	29,474	92,70	91,90
**Total:**			**24,476**	**32,074**	**100,00**	**100,00**

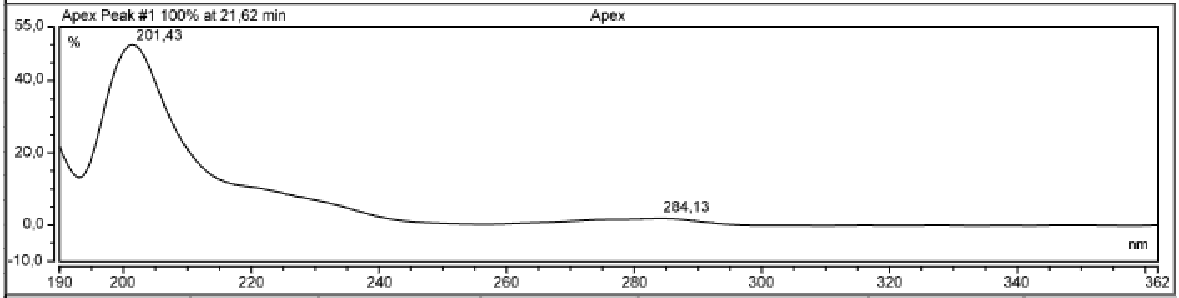

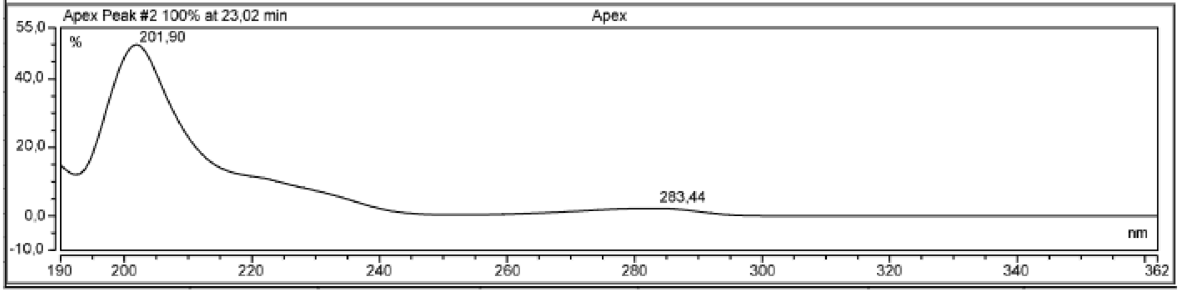

(*S,E*)-2,3-Dimethyl-6-(pent-3-en-2-yl)phenol (3g)

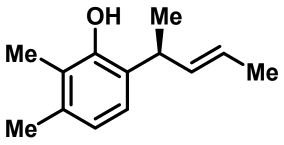

$[\alpha]^{20}$= - 18.56 (c 0.95, CH_2Cl_2).

^{1}H NMR (400 MHz, $CDCl_3$) δ 6.89 (d, *J* = 7.8 Hz, 1H), 6.74 (d, *J* = 7.8 Hz, 1H), 5.78 – 5.57 (m, 2H), 5.44 (d, *J* = 3.1 Hz, 1H), 3.60 – 3.47 (m, 1H), 2.26 (s, 4H), 2.17 (s, 3H), 1.77 – 1.69 (m, 3H), 1.38 (d, *J* = 7.0 Hz, 3H).

^{13}C NMR (101 MHz, $CDCl_3$) δ 152.3, 136.3, 135.6, 127.5, 125.9, 124.5, 122.0, 37.9, 20.1, 19.4, 18.0, 11.8.

HRMS (ESI): exact mass calculated for $C_{13}H_{17}O^-$ [(M - H)$^-$], 189.1285; found 189.1283.

86% *ee* (determined by chiral HPLC: Chiralcel® OJ-3 column, n-Heptane/EtOH = 99:1, 0.7 mL/min, λ = 287.3 nm, 25 °C), major enantiomer. t_r = 14.94 min, minor enantiomer. t_r = 19.28 min.

^{1}H NMR (400 MHz, $CDCl_3$)

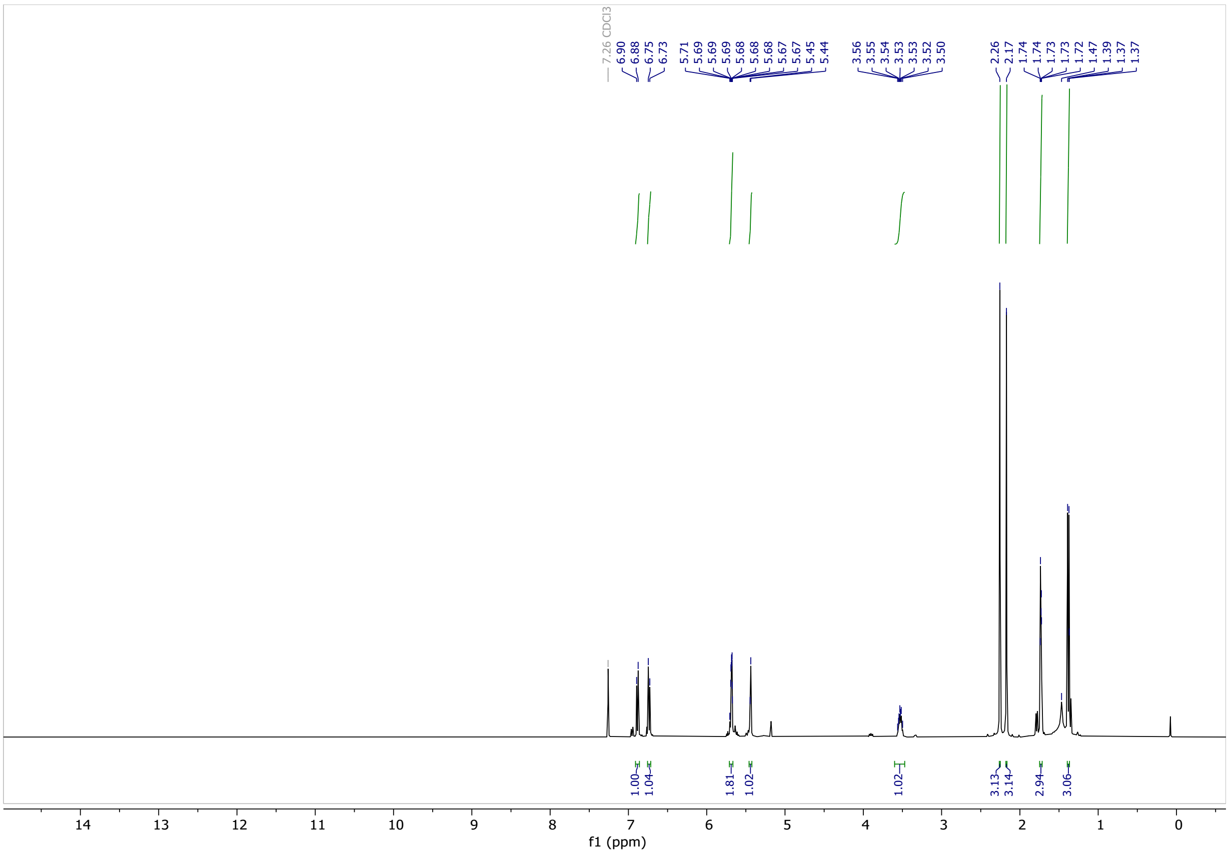

^{13}C NMR (101 MHz, $CDCl_3$)

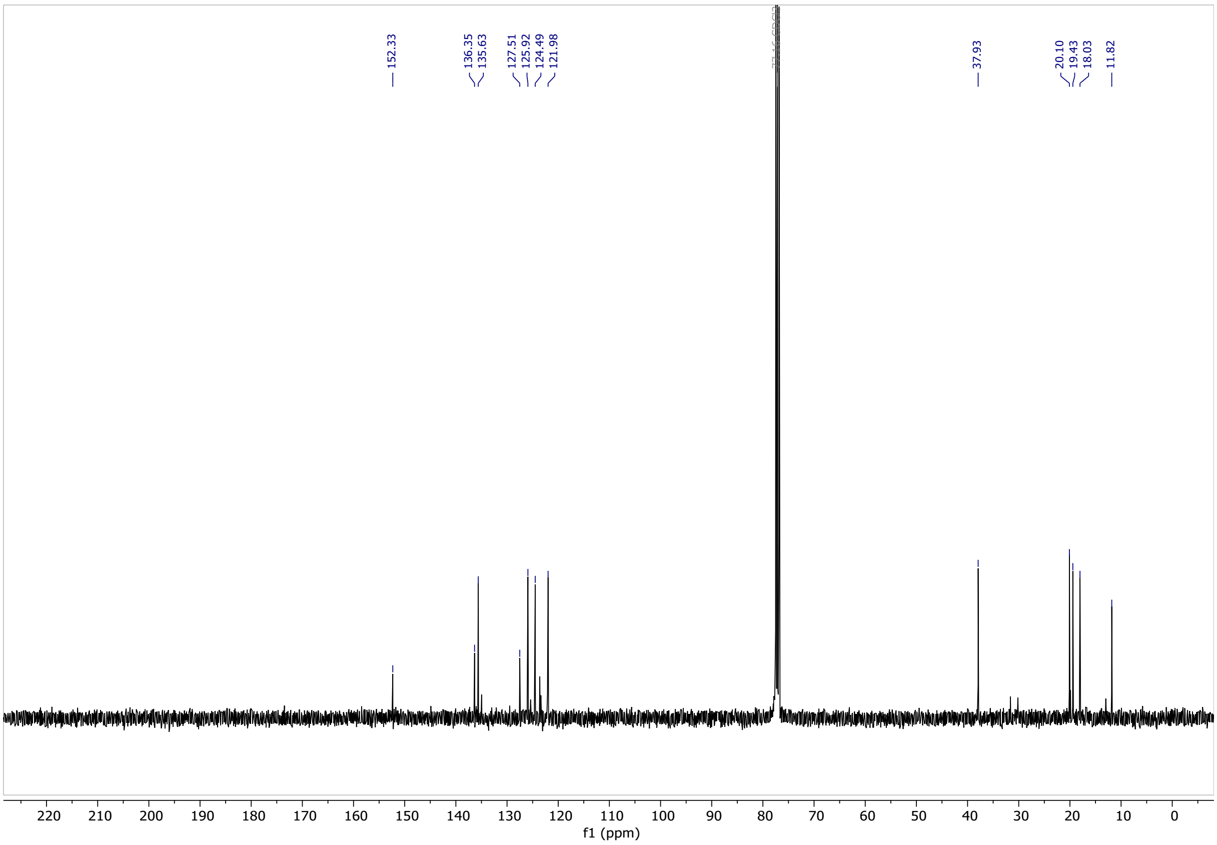

152.33
136.35
135.63
127.51
125.92
124.49
121.98
37.93
20.10
19.43
18.03
11.82
220 210 200 190 180 170 160 150 140 130 120 110 100 90 80 70 60 50 40 30 20 10 0
f1 (ppm)

Chromatogram and Results

Instrument Method:	**Heptane_EtOH_99_1_0.7mlmin_25C_40min-MK**	*B %:*	**0,0**
Column:	**OJ3**	*C %:*	**0,0**
Run Time (min):	**40,00**	*D %:*	**1,0**
Channel:	**UV_VIS_1**		
Wavelength:	**287,26**		

Chromatogram

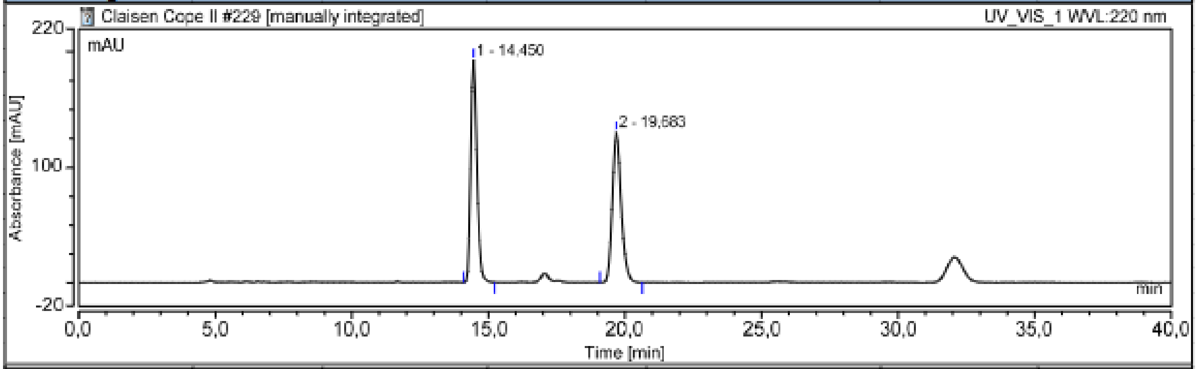

Integration Results

No.	Peak Name	Retention Time<br>min	Area<br>mAU*min	Height<br>mAU	Relative Area<br>%	Relative Height<br>%
1		14,450	49,868	192,970	50,07	59,70
2		19,683	49,731	130,278	49,93	40,30
**Total:**			**99,599**	**323,248**	**100,00**	**100,00**

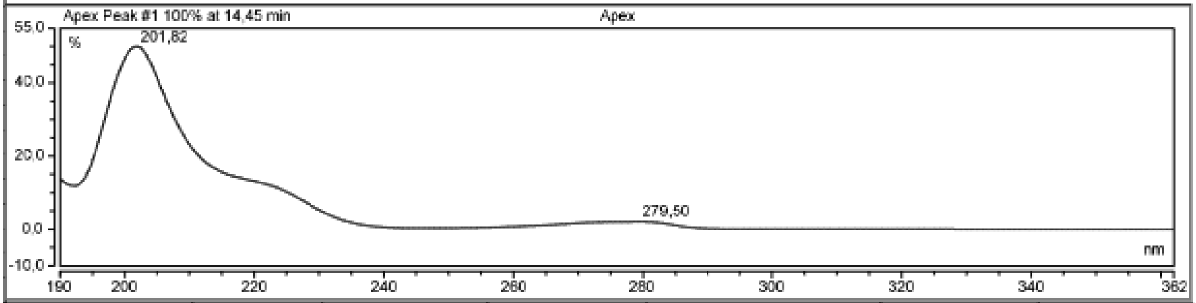

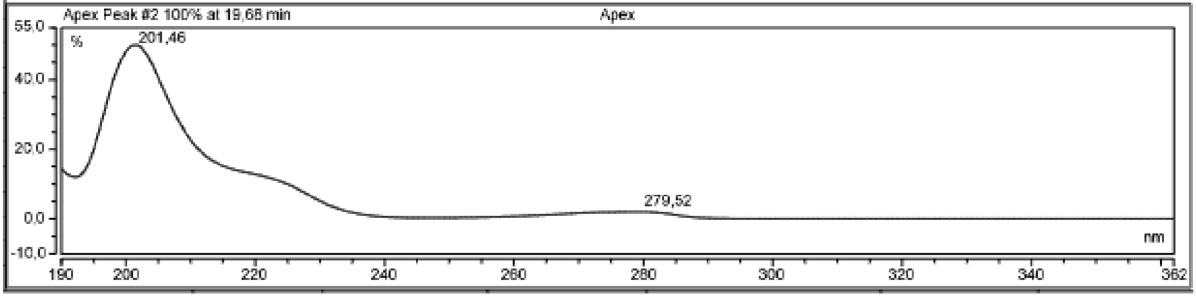

Chromatogram and Results

Instrument Method:	**Heptane_EtOH_99_1_0.7mlmin_25C_40min-MK**	*B %:*	**0,0**
Column:	**OJ3**	*C %:*	**0,0**
Run Time (min):	**40,00**	*D %:*	**1,0**
Channel:	**UV_VIS_1**		
Wavelength:	**287,26**		

Chromatogram

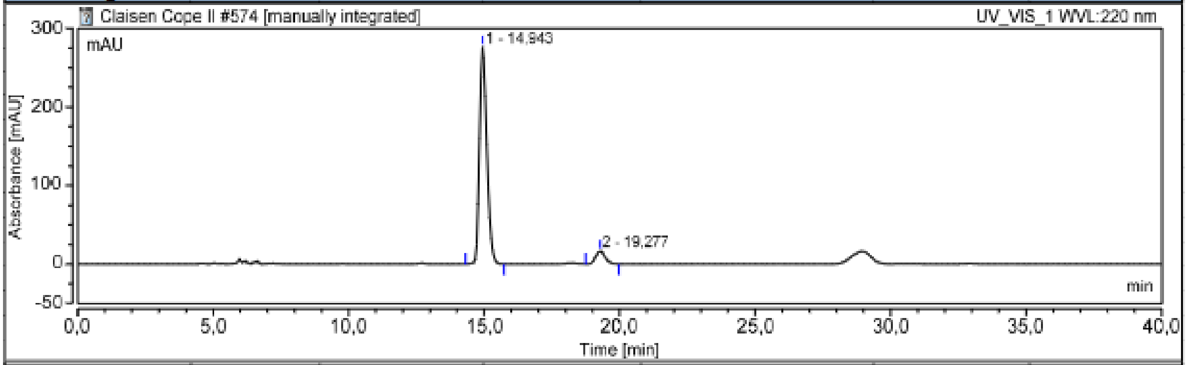

Integration Results

No.	Peak Name	Retention Time min	Area mAU*min	Height mAU	Relative Area %	Relative Height %
1		14,943	87,612	276,972	92,97	94,32
2		19,277	6,621	16,683	7,03	5,68
**Total:**			**94,233**	**293,655**	**100,00**	**100,00**

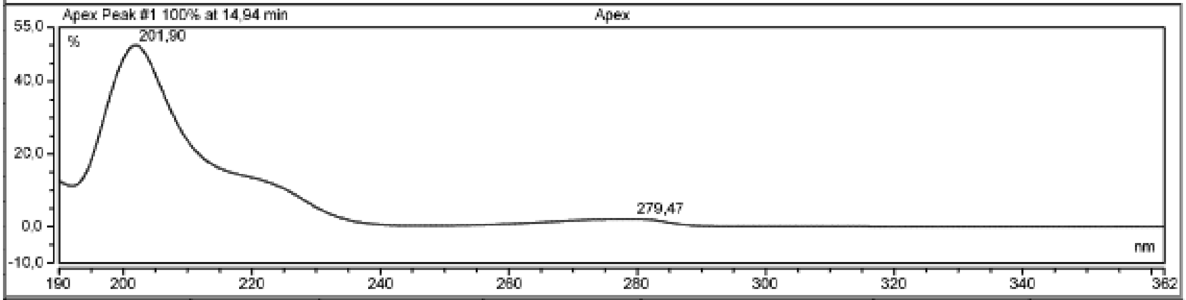

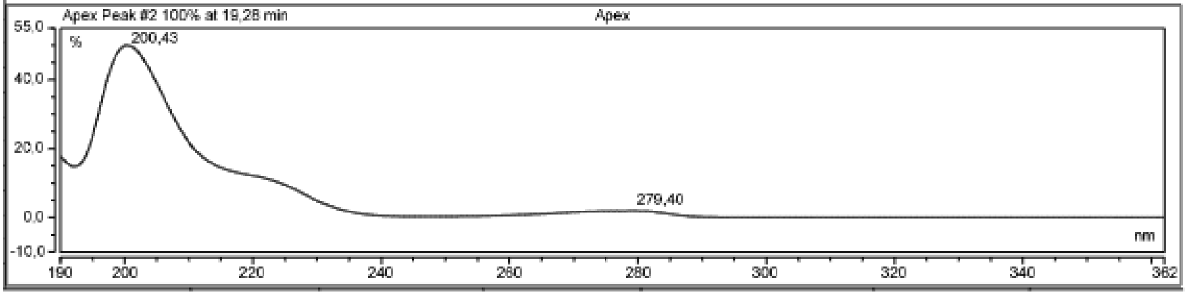

(*R,E*)-5-Isopropyl-2-methyl-4-(pent-3-en-2-yl)phenol (2h)

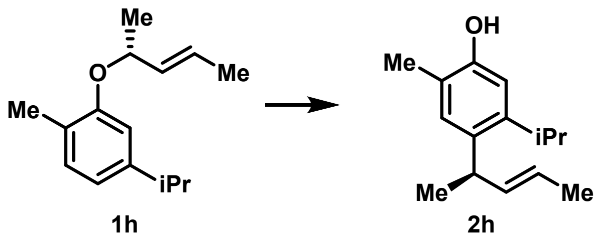

The title compound was synthesized from **1h** (100 mg, 0.46 mmol) following **general procedure B**. The reaction was directly purified by column chromatography (petroleum ether/ethyl acetate 30:1) to provide the *para*-product **2h** as colorless oil in 91% yield (91 mg, 0.42 mmol).

$[\alpha]^{20}$= + 1.53 (c 0.75, CH_2Cl_2).

^{1}H NMR (400 MHz, $CDCl_3$) δ 6.95 (d, *J* = 0.9 Hz, 1H), 6.78 (s, 1H), 5.62 (ddq, *J* = 15.3, 6.1, 1.6 Hz, 1H), 5.41 (dqd, *J* = 15.3, 6.4, 1.5 Hz, 1H), 4.99 (s, 1H), 3.68 (p, *J* = 7.0 Hz, 1H), 3.21 (hept, *J* = 6.8 Hz, 1H), 2.31 – 2.24 (m, 3H), 1.67 (dt, *J* = 6.4, 1.5 Hz, 3H), 1.30 (d, *J* = 7.0 Hz, 3H), 1.20 (t, *J* = 7.1 Hz, 6H).

^{13}C NMR (101 MHz, $CDCl_3$) δ 152.3, 145.3, 136.9, 135.6, 129.4, 123.2, 121.0, 112.0, 36.2, 28.2, 24.2, 24.2, 22.0, 18.1, 15.7.

HRMS (ESI): exact mass calculated for $C_{15}H_{21}O^-$ [(M - H)$^-$], 217.1598; found 217.1598.

84% *ee* (determined by chiral HPLC: Chiralpak® AS-H column, n-Heptane/IPA = 99.5:0.5, 0.7 mL/min, λ = 287.3 nm, 25 °C), major enantiomer. t_r = 26.30 min, minor enantiomer. t_r = 28.91 min.

^{1}H NMR (400 MHz, $CDCl_3$)

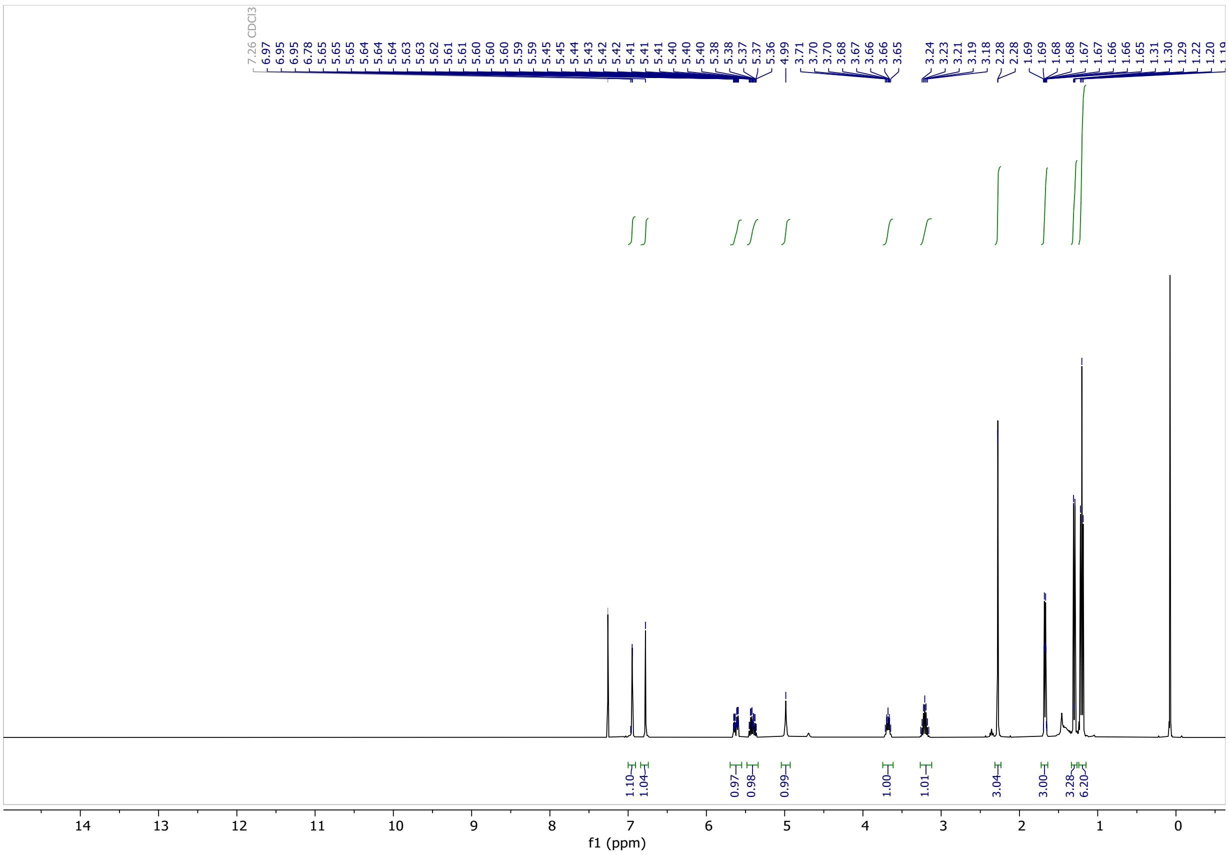

^{13}C NMR (101 MHz, $CDCl_3$)

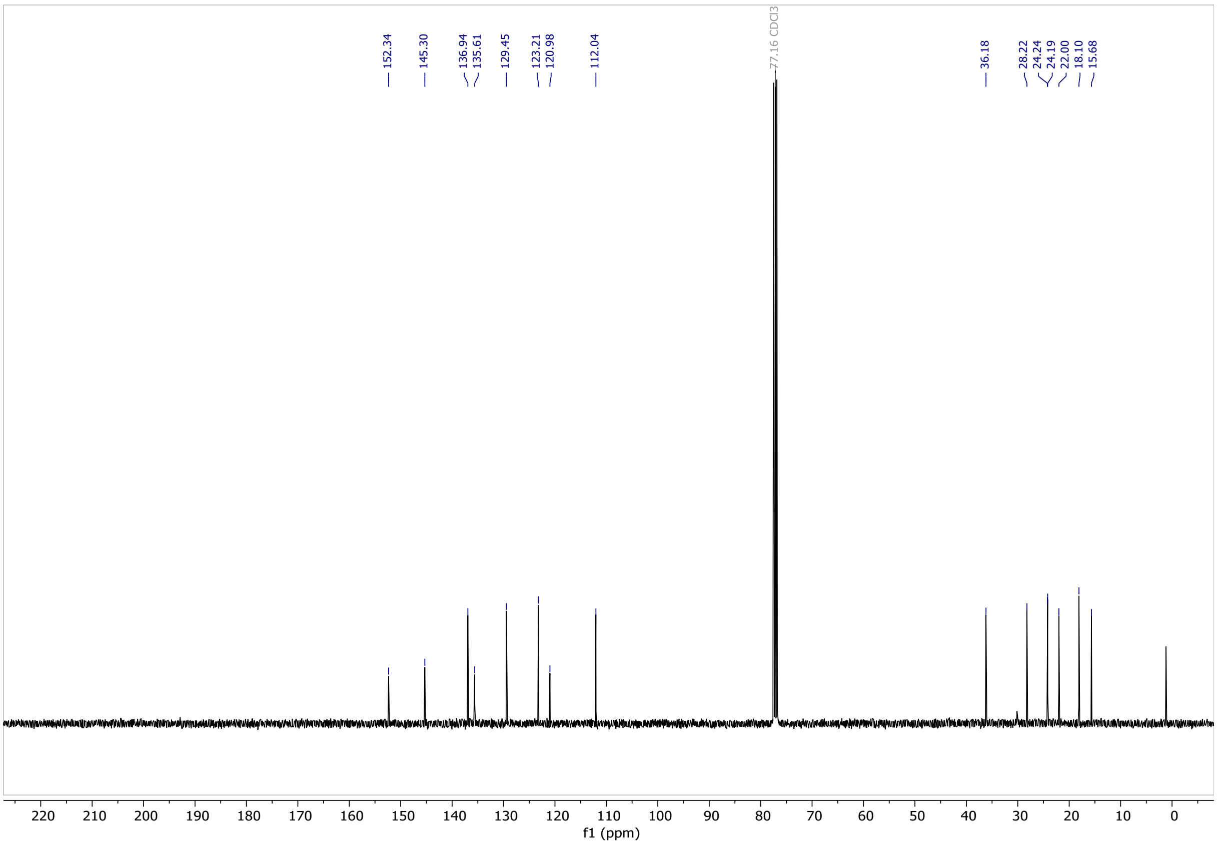
152.34
145.30
136.94
135.61
129.45
123.21
120.98
112.04
77.16 CDCl3
36.18
28.22
24.24
24.19
22.00
18.10
15.68
220 210 200 190 180 170 160 150 140 130 120 110 100 90 80 70 60 50 40 30 20 10 0
f1 (ppm)

Chromatogram and Results

Instrument Method:	**Heptane_IPA_99.5_0.5_0.7mlmin_25C_45min-MK**	*B %:*	**0,5**
Column:	**AS-H**	*C %:*	**0,0**
Run Time (min):	**45,00**	*D %:*	**0,0**
Channel:	**UV_VIS_1**		
Wavelength:	**287,26**		

Chromatogram

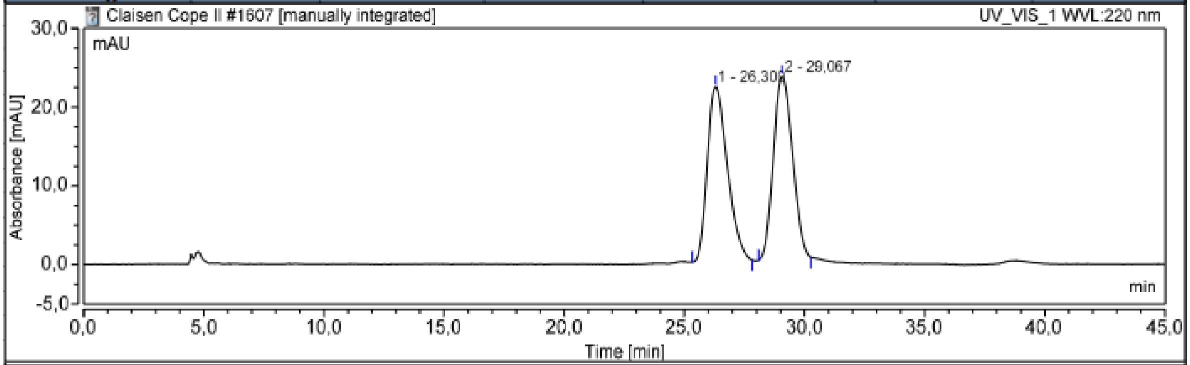

Integration Results

No.	Peak Name	Retention Time<br>min	Area<br>mAU*min	Height<br>mAU	Relative Area<br>%	Relative Height<br>%
1		26,300	21,483	22,173	50,10	48,85
2		29,067	21,394	23,213	49,90	51,15
**Total:**			**42,877**	**45,386**	**100,00**	**100,00**

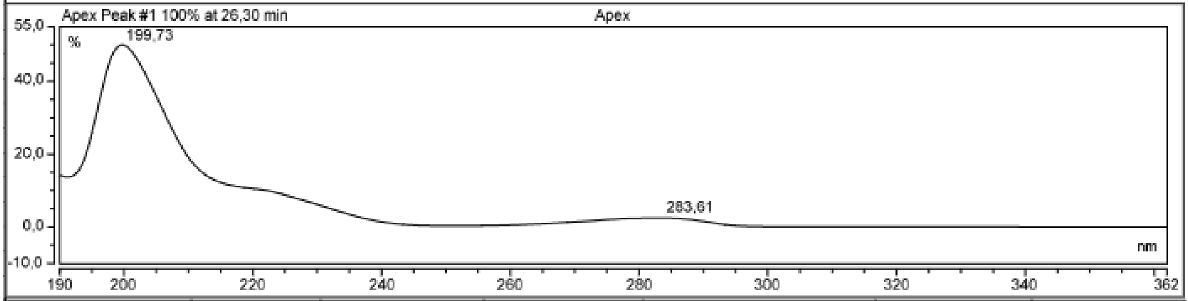

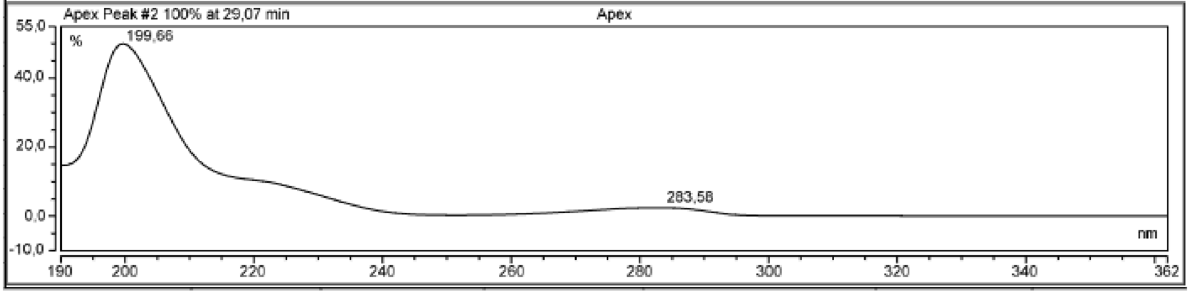

Chromatogram and Results

Instrument Method:	**Heptane_IPA_99.5_0.5_0.7mlmin_25C_45min-MK**	*B %:*	**0,5**
Column:	**AS-H**	*C %:*	**0,0**
Run Time (min):	**45,00**	*D %:*	**0,0**
Channel:	**UV_VIS_1**		
Wavelength:	**287,26**		

Chromatogram

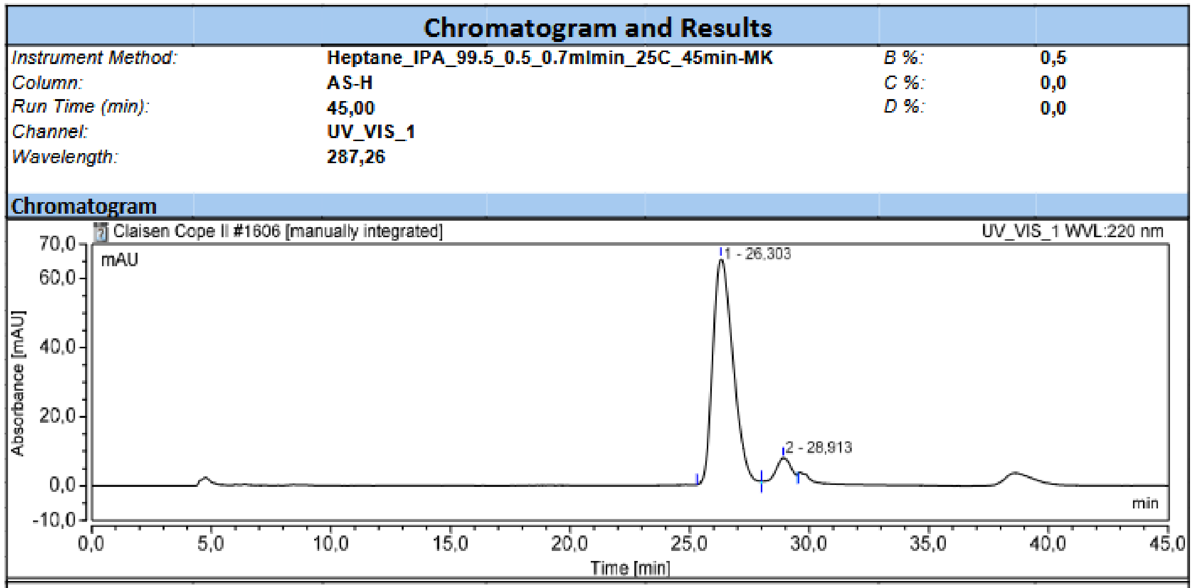

Integration Results

No.	Peak Name	Retention Time<br>min	Area<br>mAU*min	Height<br>mAU	Relative Area<br>%	Relative Height<br>%
1		26,303	64,150	65,396	91,82	89,93
2		28,913	5,716	7,321	8,18	10,07
**Total:**			**69,866**	**72,716**	**100,00**	**100,00**

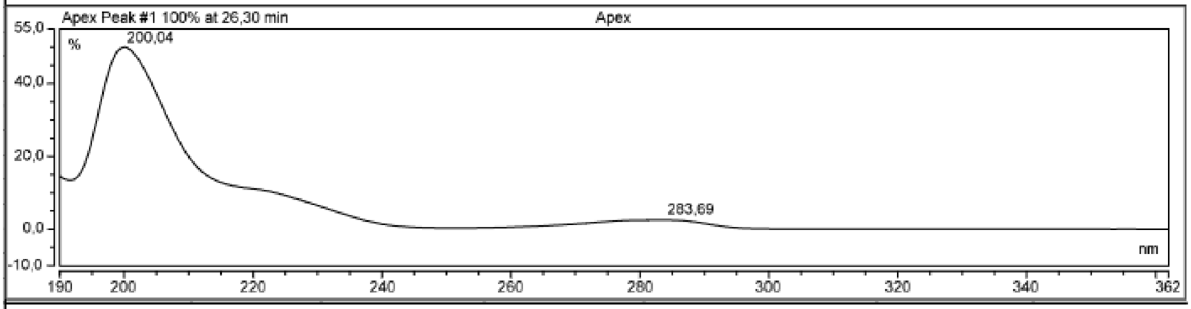

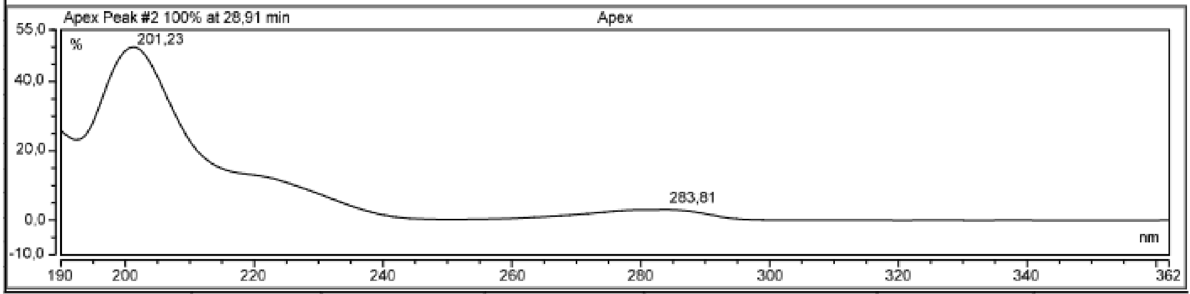

(*R,E*)-2-Isopropyl-5-methyl-4-(pent-3-en-2-yl)phenol (2i) & (*S,E*)-6-isopropyl-3-methyl-2-(pent-3-en-2-yl)phenol (3i)

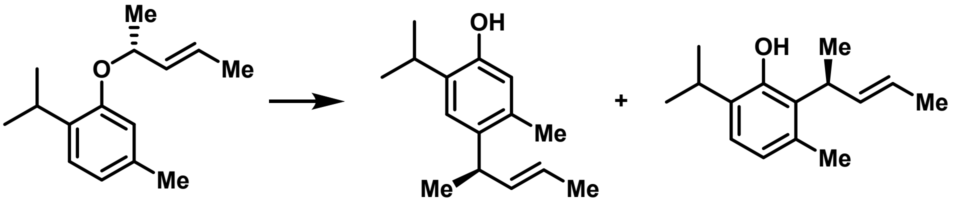

1i **2i** **3i**

The title compounds were synthesized from **1i** (100 mg, 0.46 mmol) following **general procedure B**. The reaction was directly purified by column chromatography (petroleum ether/ethyl acetate 30:1) to provide the *para*-product **2i** as colorless oil in 54% yield (54 mg, 0.25 mmol) and the *ortho*-product **3i** as colorless oil in 44% yield (44 mg, 0.20 mmol).

(*R,E*)-2-Isopropyl-5-methyl-4-(pent-3-en-2-yl)phenol (2i)

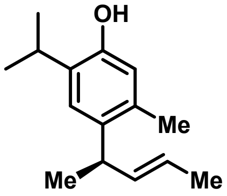

$[\alpha]^{20}$= - 12.51 (c 2.35, CH_2Cl_2).

^{1}H NMR (400 MHz, $CDCl_3$) δ 6.93 (s, 1H), 6.49 (s, 1H), 5.48 (ddq, *J* = 15.3, 6.4, 1.5 Hz, 1H), 5.33 (dqd, *J* = 15.3, 6.3, 1.3 Hz, 1H), 4.58 (s, 1H), 3.52 – 3.38 (m, 1H), 3.10 (hept, *J* = 6.9 Hz, 1H), 2.16 (s, 3H), 1.65 – 1.56 (m, 3H), 1.22 (d, *J* = 7.0 Hz, 3H), 1.19 (d, *J* = 6.9 Hz, 6H).

^{13}C NMR (101 MHz, $CDCl_3$) δ 150.9, 136.8, 136.3, 134.1, 131.9, 124.4, 123.3, 117.4, 37.6, 27.5, 22.9, 22.9, 21.1, 19.0, 18.1.

HRMS (ESI): exact mass calculated for $C_{15}H_{21}O^-$ [(M - H)$^-$], 217.1598; found 217.1592.

91% *ee* (determined by chiral HPLC: Chiralcel® OJ-3 column, n-Heptane/EtOH = 99.5:0.5, 0.7 mL/min, λ = 287.3 nm, 25 °C), major enantiomer. t_r = 23.74 min, minor enantiomer. t_r = 25.11 min

^{1}H NMR (400 MHz, $CDCl_3$)

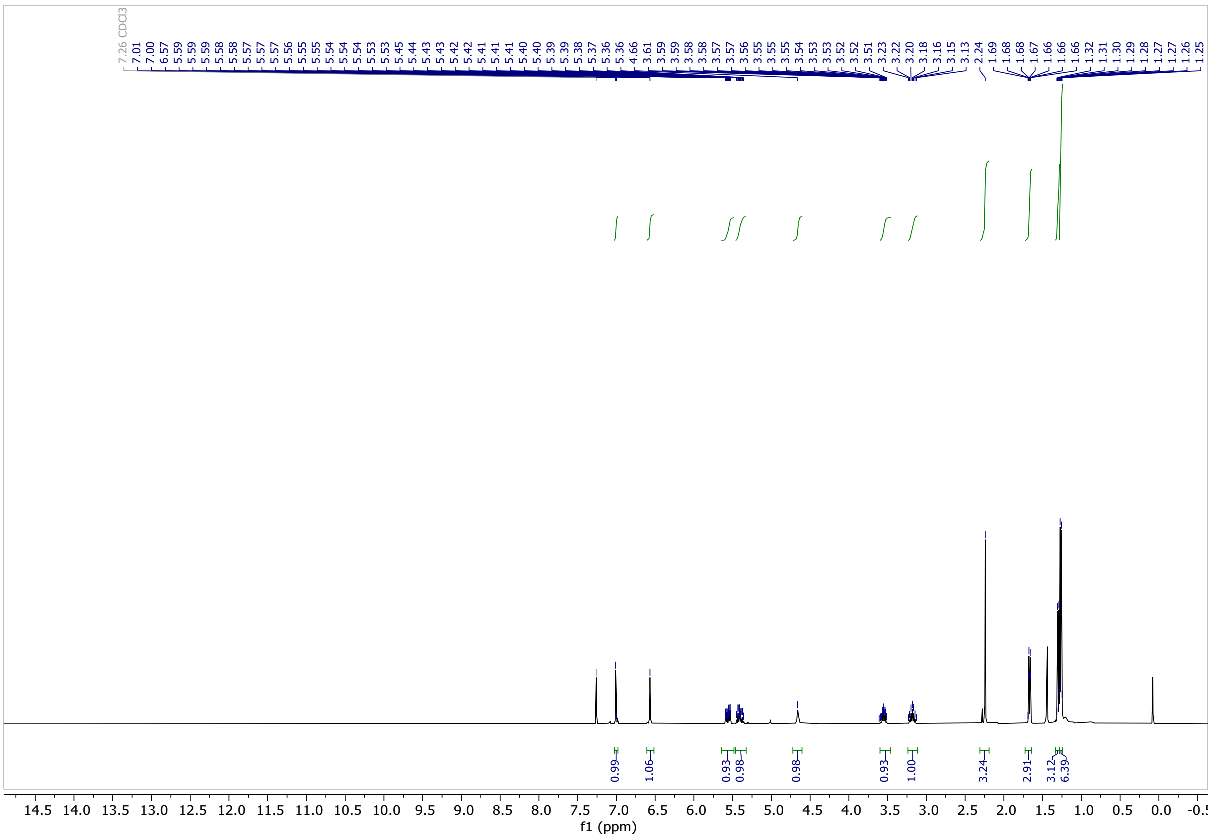

^{13}C NMR (101 MHz, $CDCl_3$)

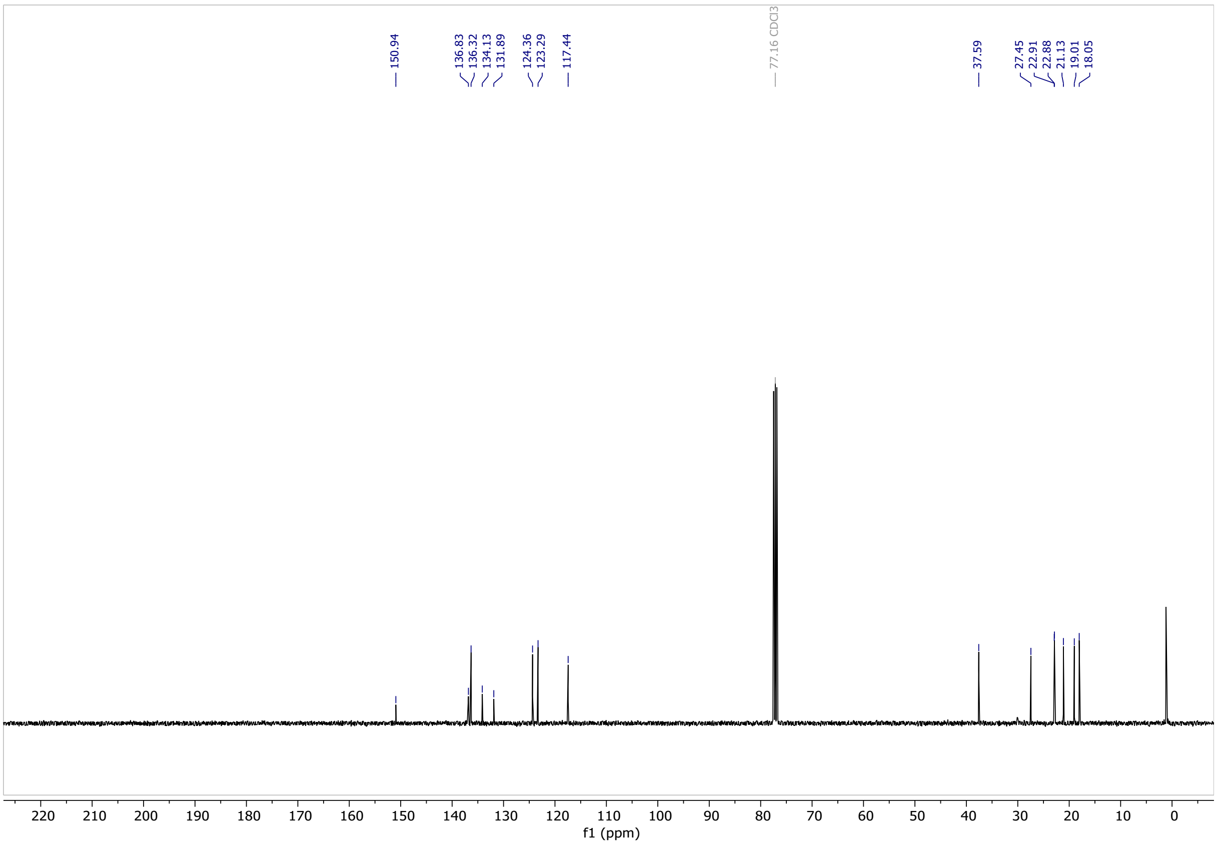

Chromatogram and Results

Instrument Method:	**Heptane_EtOH_99.5_0.5_0.7mlmin_25C_30min**	*B %:*	**0,0**
Column:	**OJ3**	*C %:*	**0,0**
Run Time (min):	**30,00**	*D %:*	**0,5**
Channel:	**UV_VIS_1**		
Wavelength:	**287,26**		

Chromatogram

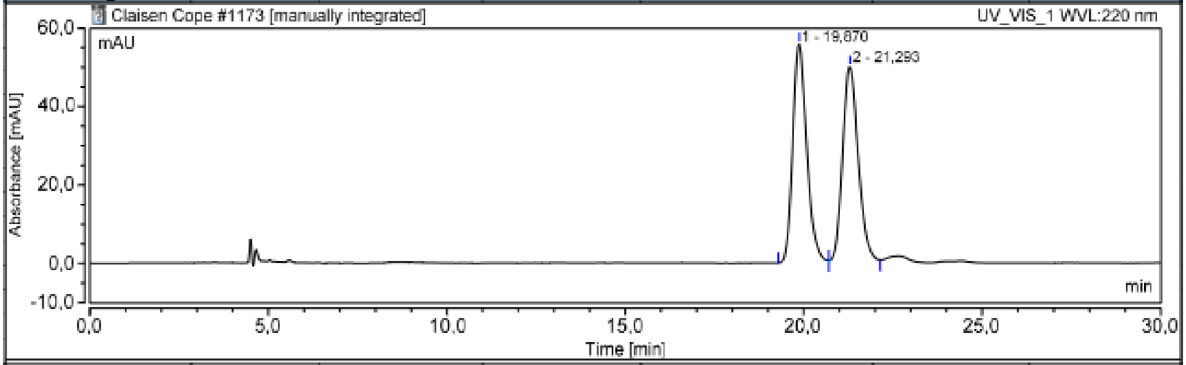

Integration Results

No.	Peak Name	Retention Time min	Area mAU*min	Height mAU	Relative Area %	Relative Height %
1		19,870	25,348	55,766	50,47	52,96
2		21,293	24,877	49,530	49,53	47,04
**Total:**			**50,225**	**105,296**	**100,00**	**100,00**

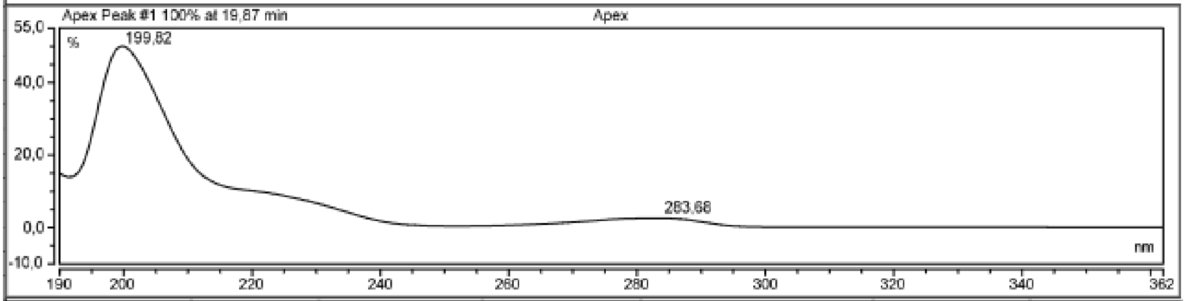

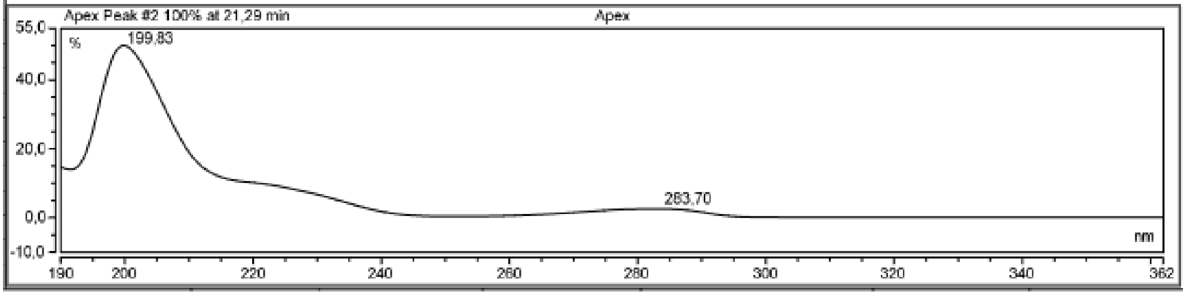

Chromatogram and Results

Instrument Method:	**Heptane_EtOH_99.5_0.5_0.7mlmin_25C_30min**	*B %:*	**0,0**
Column:	**OJ3**	*C %:*	**0,0**
Run Time (min):	**30,00**	*D %:*	**0,5**
Channel:	**UV_VIS_1**		
Wavelength:	**287,26**		

Chromatogram

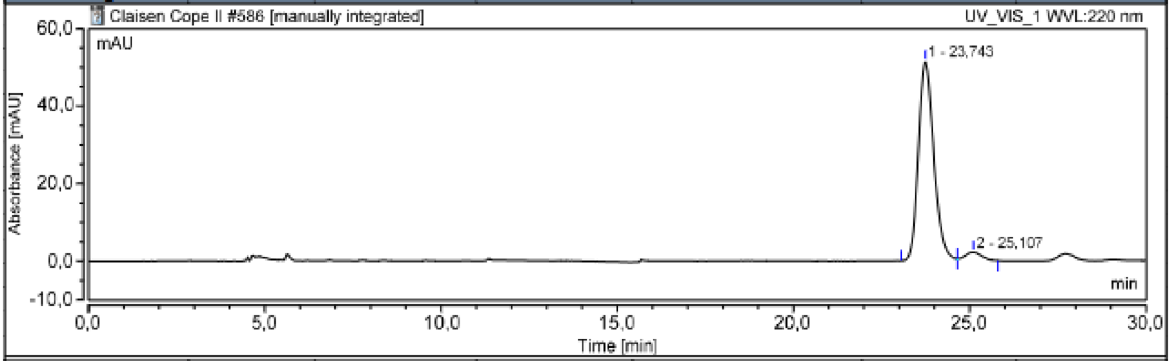

Integration Results

No.	Peak Name	Retention Time<br>min	Area<br>mAU*min	Height<br>mAU	Relative Area<br>%	Relative Height<br>%
1		23,743	26,062	51,446	95,42	95,82
2		25,107	1,251	2,246	4,58	4,18
**Total:**			**27,313**	**53,693**	**100,00**	**100,00**

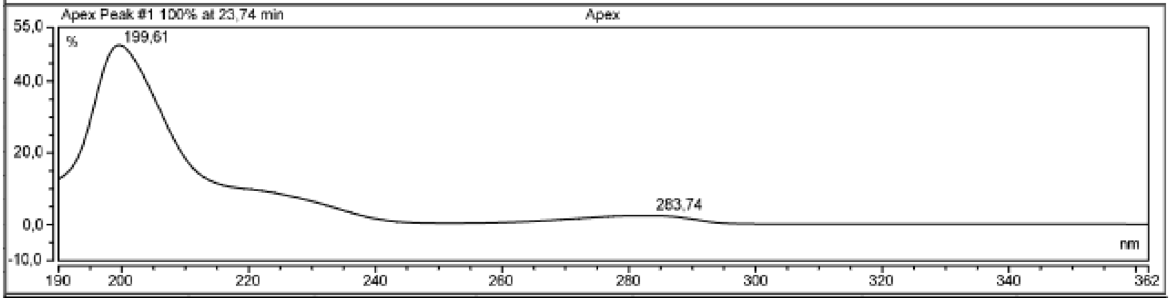

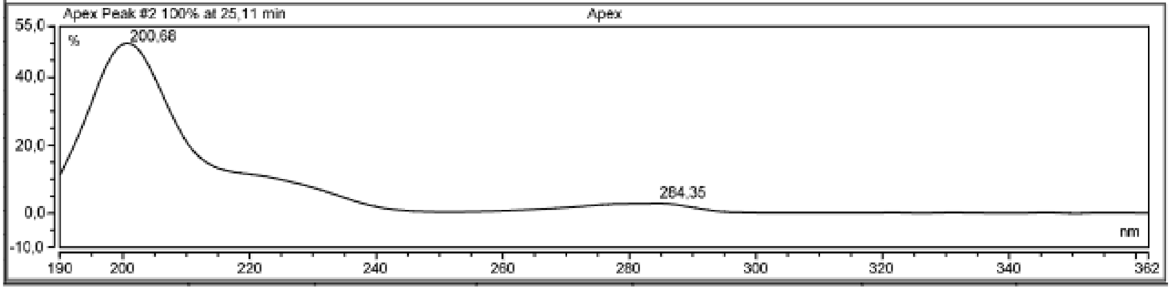

(*S,E*)-6-Isopropyl-3-methyl-2-(pent-3-en-2-yl)phenol (3i)

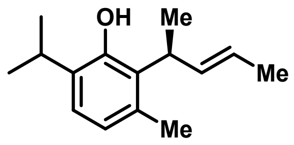

^{1}H NMR (400 MHz, $CDCl_3$) δ 6.98 (d, *J* = 7.8 Hz, 1H), 6.72 (d, *J* = 7.8 Hz, 1H), 6.01 – 5.91 (m, 2H), 5.91 – 5.79 (m, 1H), 3.82 (ttd, *J* = 7.1, 4.4, 2.4 Hz, 1H), 3.24 (hept, *J* = 7.0 Hz, 1H), 2.30 (s, 3H), 1.81 (ddt, *J* = 5.9, 2.1, 1.0 Hz, 3H), 1.39 (dd, *J* = 7.1, 0.8 Hz, 3H), 1.21 (ddd, *J* = 6.9, 3.9, 0.9 Hz, 6H).

^{13}C NMR (101 MHz, $CDCl_3$) δ 152.7, 135.0, 134.3, 133.7, 128.0, 126.9, 124.1, 122.4, 35.1, 26.6, 23.0, 22.8, 20.4, 18.3, 16.4.

HRMS (ESI): exact mass calculated for $C_{15}H_{21}O^-$ [(M - H)$^-$], 217.1598; found 217.1587.

ee not determined. No separation found on columns IB, OJ-3, OD, AS-H, IA-3.

^{1}H NMR (400 MHz, $CDCl_3$)

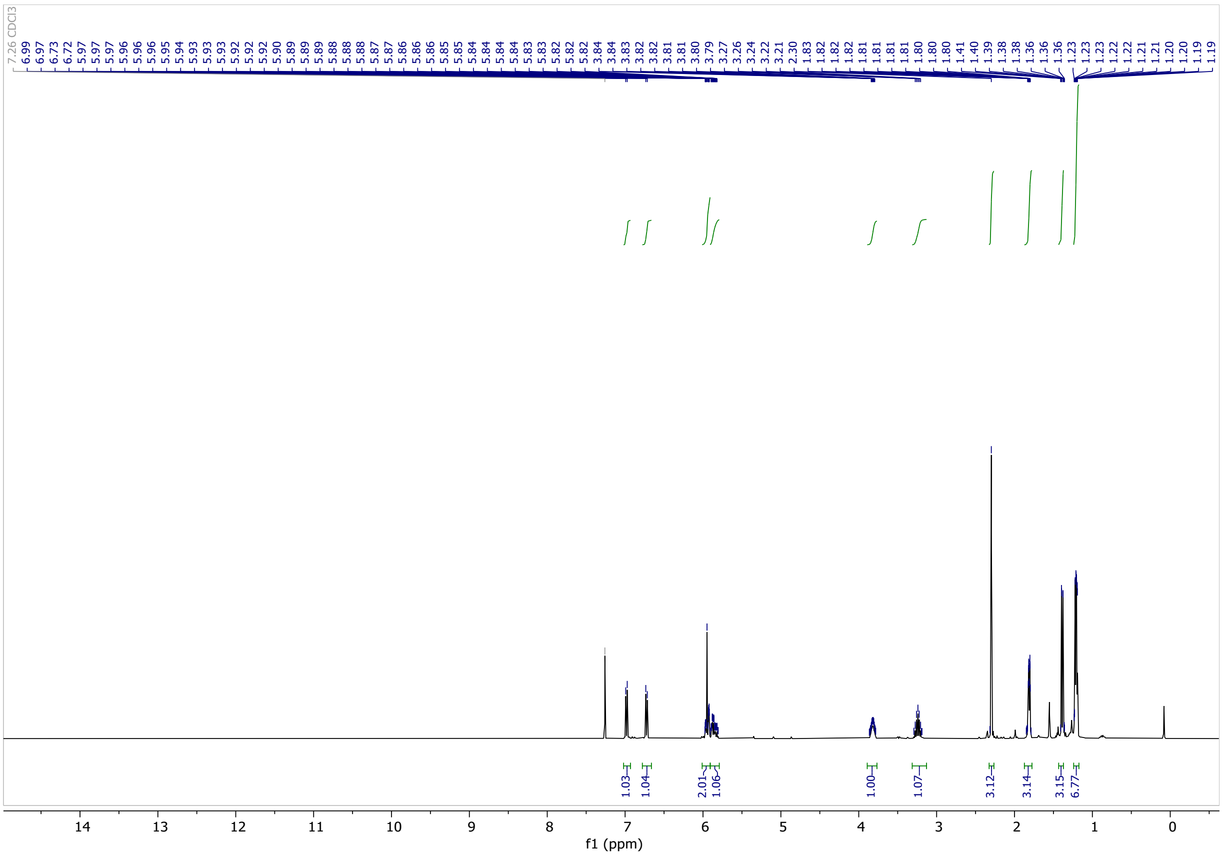

^{13}C NMR (101 MHz, $CDCl_3$)

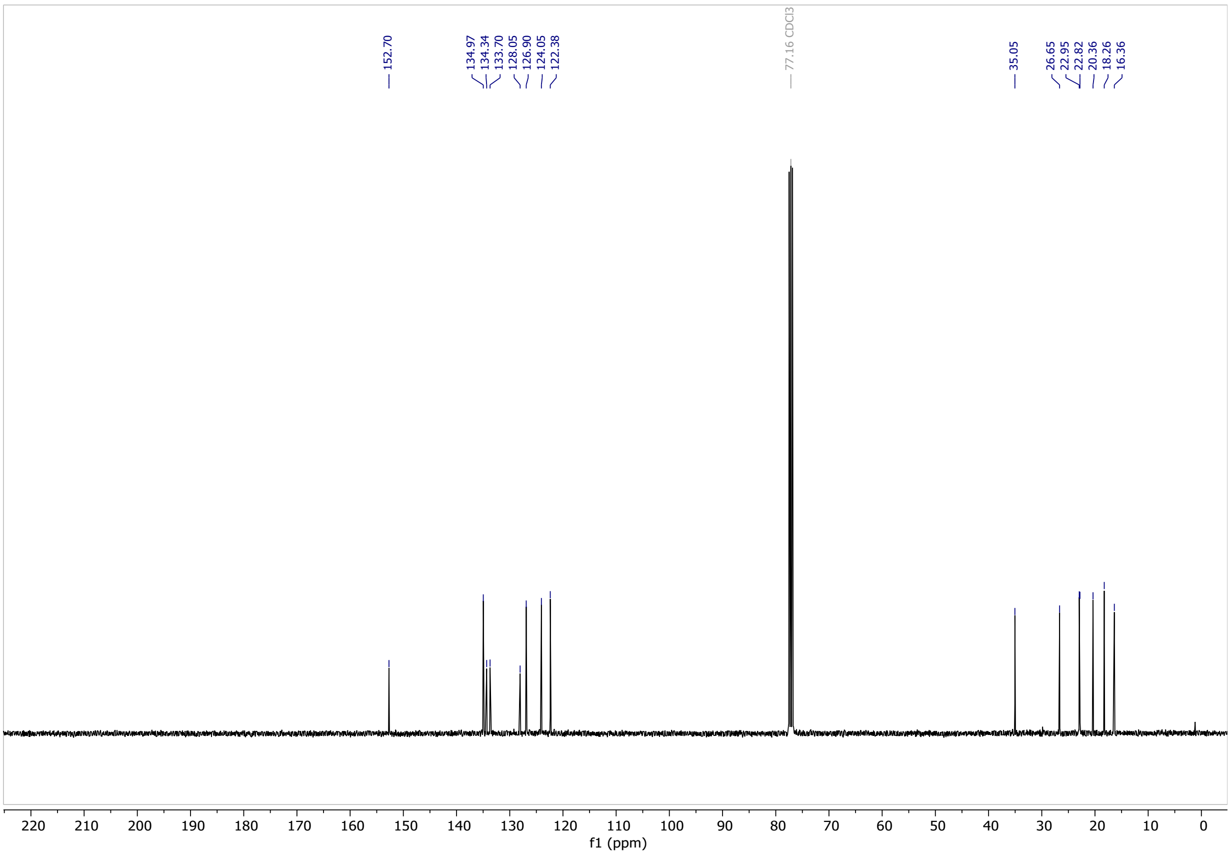

(*R,E*)-5-Methoxy-2-methyl-4-(pent-3-en-2-yl)phenol (2j) & (*S,E*)-3-Methoxy-6-methyl-2-(pent-3-en-2-yl)phenol (3j)

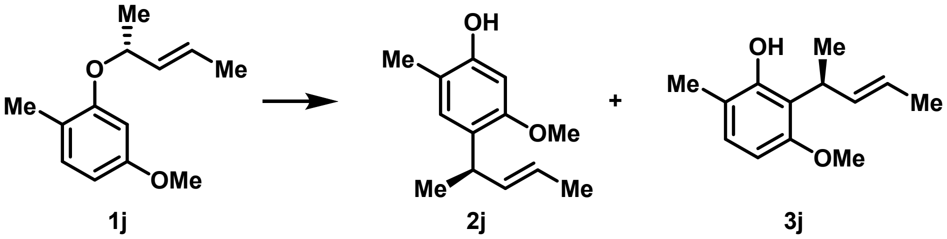

The title compounds were synthesized from **1j** (100 mg, 0.49 mmol) following **general procedure B**. The reaction was directly purified by column chromatography (petroleum ether/ethyl acetate 30:1) to provide the *para*-product **2j** as colorless oil in 42% yield (42 mg, 0.20 mmol) and the *ortho*-product **3j** as colorless oil in 47% yield (47 mg, 0.23 mmol).

(*R,E*)-5-Methoxy-2-methyl-4-(pent-3-en-2-yl)phenol (2j)

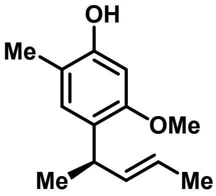

^{1}H NMR (400 MHz, $CDCl_3$) δ 6.85 (s, 1H), 6.38 (d, *J* = 2.9 Hz, 1H), 5.63 (ddq, *J* = 15.2, 6.3, 1.6 Hz, 1H), 5.43 (dqd, *J* = 15.4, 6.4, 1.4 Hz, 1H), 4.60 – 4.44 (m, 1H), 3.77 (s, 3H), 3.76 – 3.68 (m, 1H), 2.17 (s, 3H), 1.67 (dt, *J* = 6.4, 1.5 Hz, 3H), 1.24 (dd, *J* = 7.0, 1.5 Hz, 3H).

^{13}C NMR (101 MHz, $CDCl_3$) δ 155.9, 152.4, 136.1, 129.5, 127.2, 123.1, 99.2, 55.8, 34.3, 20.7, 18.1, 15.1.

HRMS (ESI): exact mass calculated for $C_{13}H_{17}O_2^-$ [(M - H)$^-$], 205.1234; found 205.1232.

ee not determined. No separation found on columns IB, OJ-3, OD, AS-H, IA-3.

^{1}H NMR (400 MHz, $CDCl_3$)

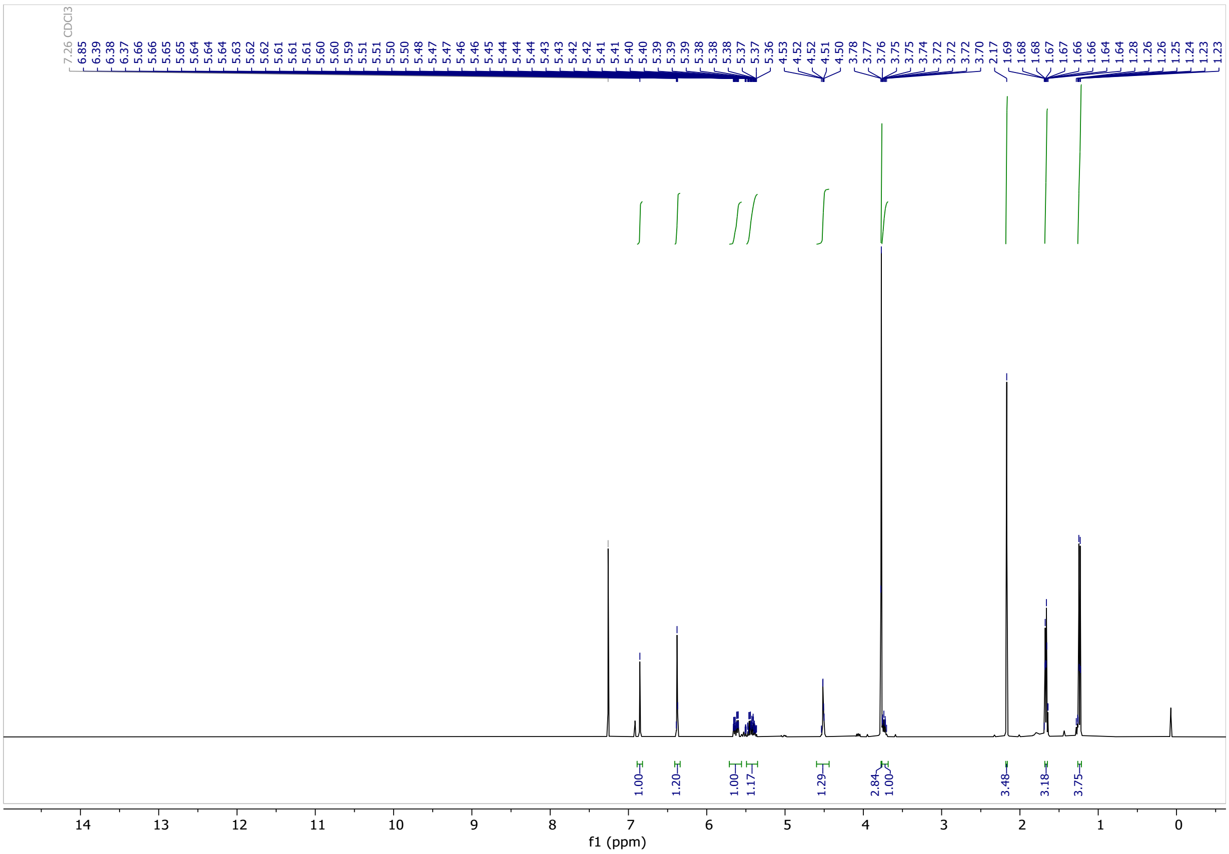

^{13}C NMR (101 MHz, $CDCl_3$)

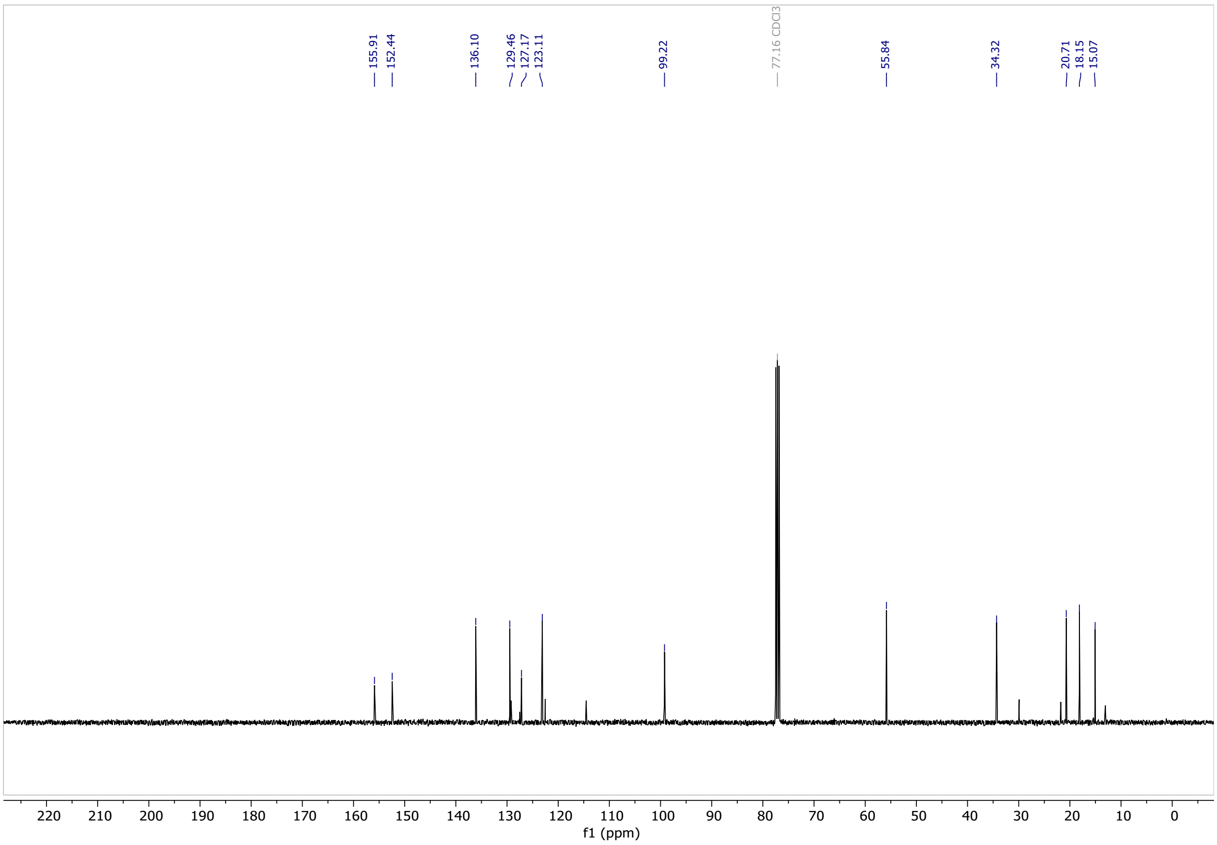

(*S,E*)-3-Methoxy-6-methyl-2-(pent-3-en-2-yl)phenol (3j)

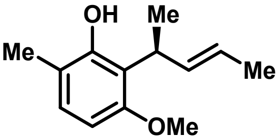

$[\alpha]^{20}$= - 14.24 (c 2.10, CH_2Cl_2).

^{1}H NMR (400 MHz, $CDCl_3$) δ 6.91 – 6.78 (m, 1H), 6.31 (d, *J* = 8.3 Hz, 1H), 5.91 (s, 1H), 5.90 – 5.66 (m, 2H), 4.16 – 4.00 (m, 1H), 3.70 (s, 3H), 2.06 (s, 3H), 1.71 (ddd, *J* = 6.1, 2.3, 1.3 Hz, 3H), 1.26 (dd, *J* = 7.1, 0.8 Hz, 3H).

^{13}C NMR (101 MHz, $CDCl_3$) δ 155.7, 154.1, 135.3, 128.5, 126.4, 118.3, 118.2, 102.7, 56.0, 30.9, 18.2, 17.0, 15.6.

84% *ee* (determined by chiral HPLC: Chiralcel® OJ-3 column, n-Heptane/EtOH = 99:1, 0.7 mL/min, λ = 287.3 nm, 25 °C), major enantiomer. t_r = 10.08 min, minor enantiomer. t_r = 7.77 min.

^{1}H NMR (400 MHz, $CDCl_3$)

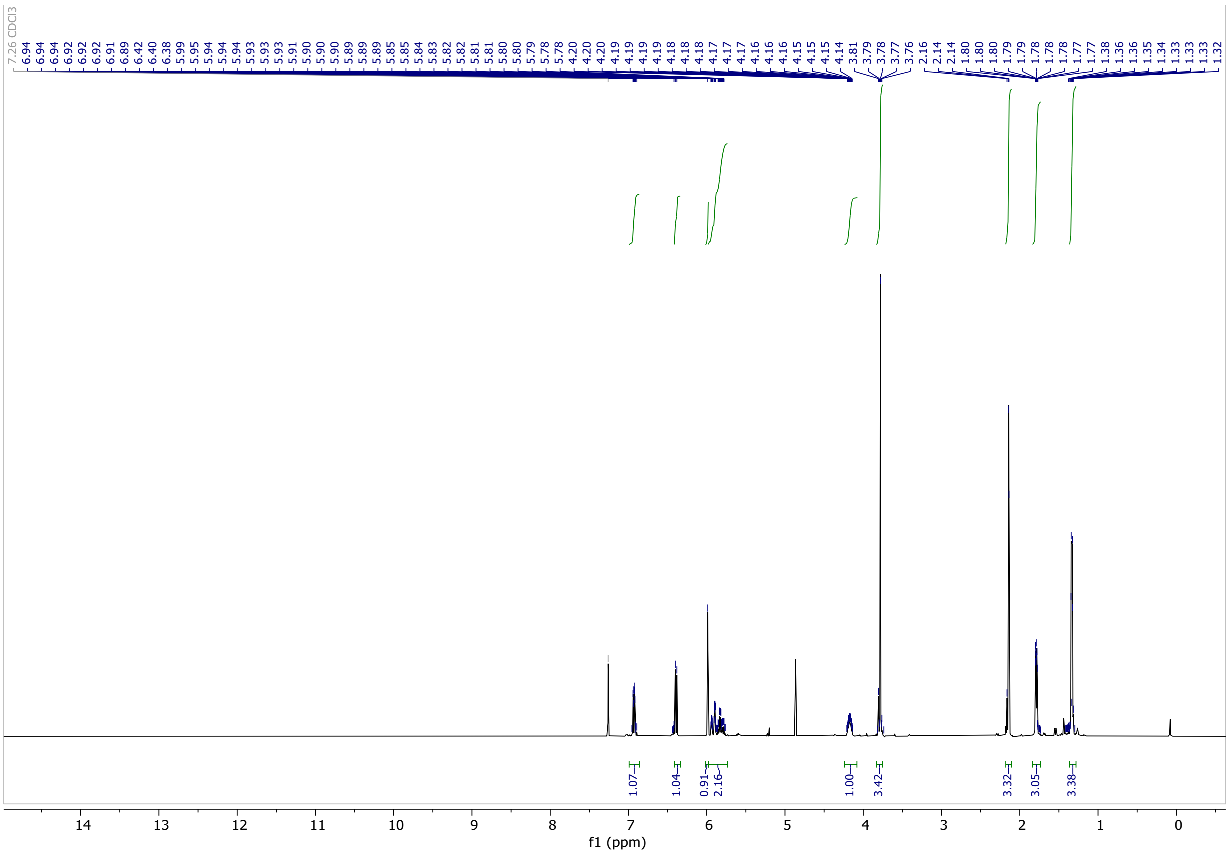

^{13}C NMR (101 MHz, $CDCl_3$)

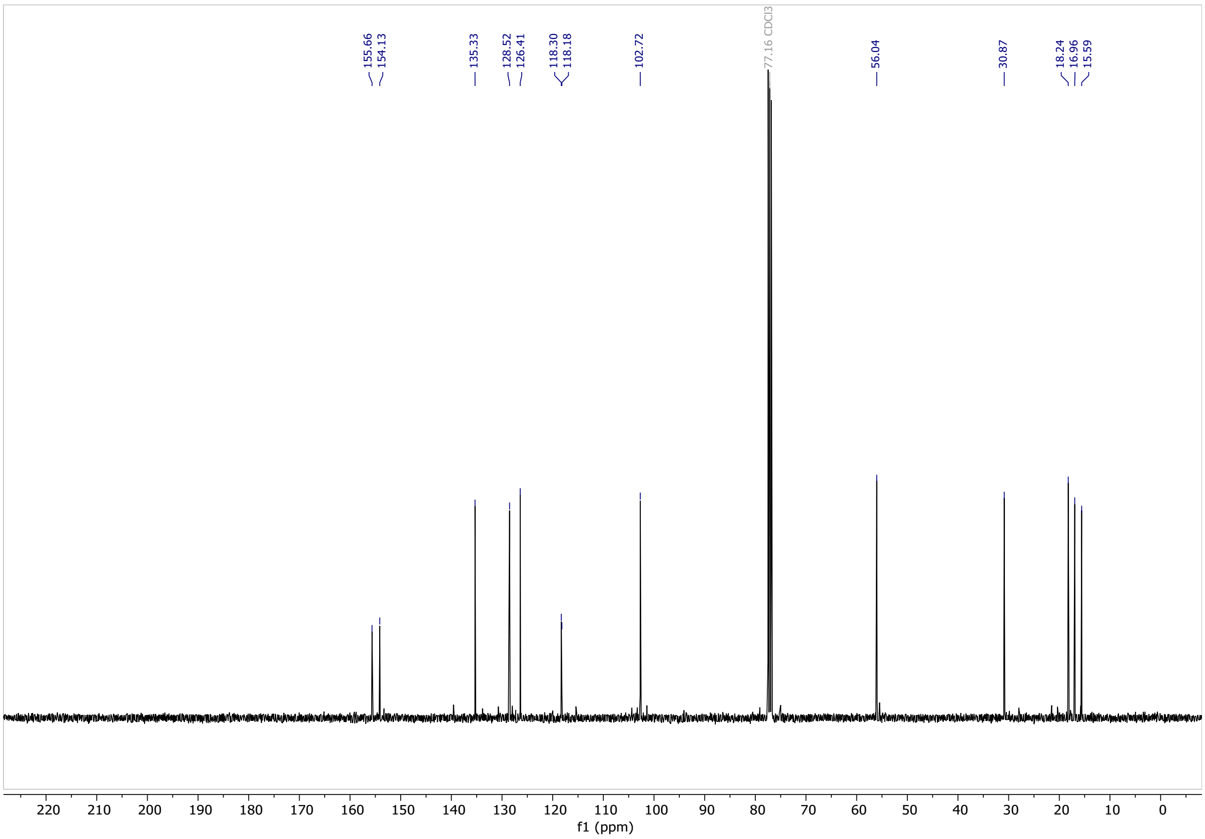

Chromatogram and Results

Instrument Method:	**Heptane_EtOH_99_1_0.7mlmin_25C_40min-MK**	*B %:*	**0,0**
Column:	**OJ3**	*C %:*	**0,0**
Run Time (min):	**40,00**	*D %:*	**1,0**
Channel:	**UV_VIS_1**		
Wavelength:	**287,26**		

Chromatogram

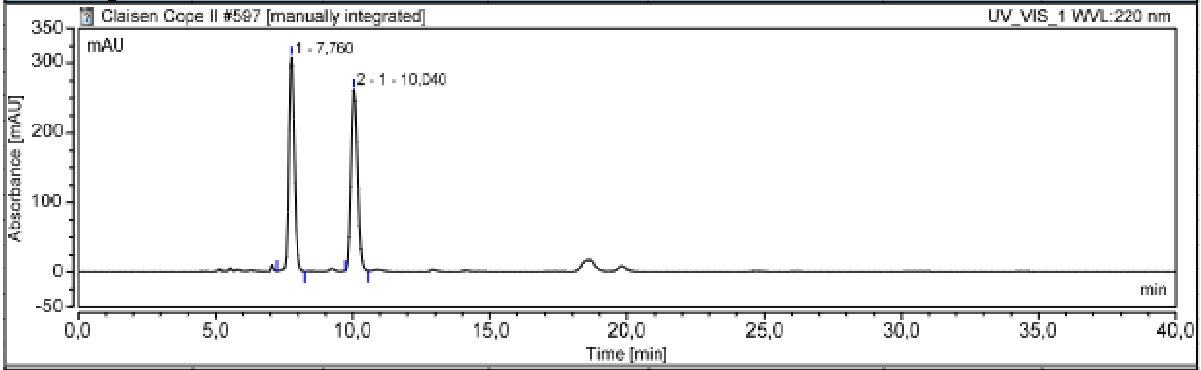

Integration Results

No.	Peak Name	Retention Time min	Area mAU*min	Height mAU	Relative Area %	Relative Height %
1		7,760	72,778	308,681	50,77	54,19
2	1	10,040	70,559	260,913	49,23	45,81
**Total:**			**143,338**	**569,594**	**100,00**	**100,00**

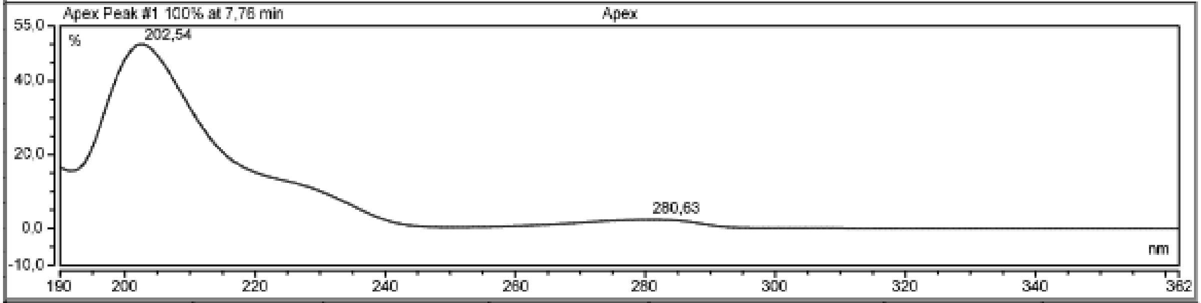

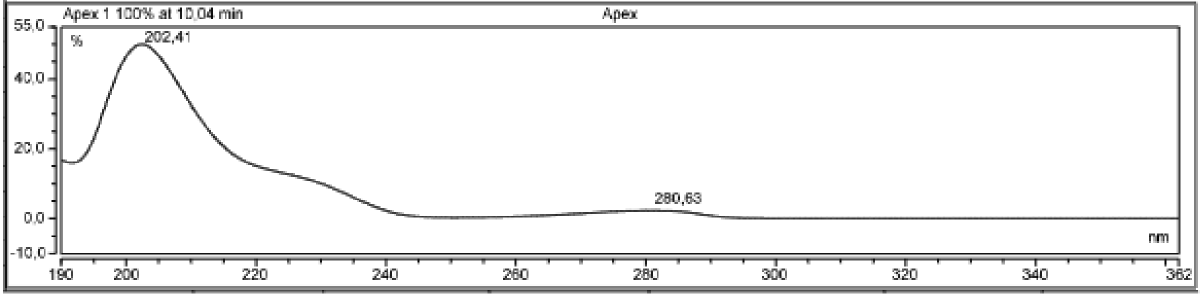

Chromatogram and Results

Instrument Method:	**Heptane_EtOH_99_1_0.7mlmin_25C_40min-MK**	*B %:*	**0,0**
Column:	**OJ3**	*C %:*	**0,0**
Run Time (min):	**40,00**	*D %:*	**1,0**
Channel:	**UV_VIS_1**		
Wavelength:	**287,26**		

Chromatogram

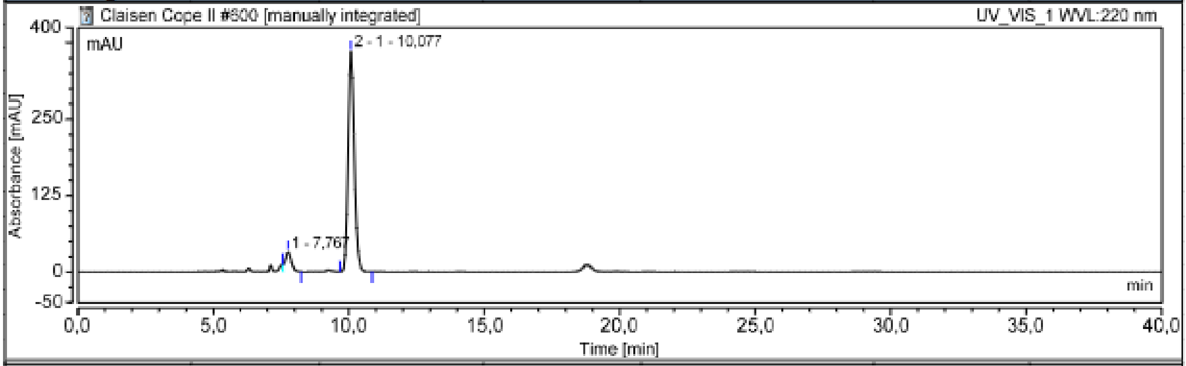

Integration Results

No.	Peak Name	Retention Time min	Area mAU*min	Height mAU	Relative Area %	Relative Height %
1		7,767	8,758	33,143	8,25	8,42
2	1	10,077	97,374	360,466	91,75	91,58
**Total:**			**106,132**	**393,609**	**100,00**	**100,00**

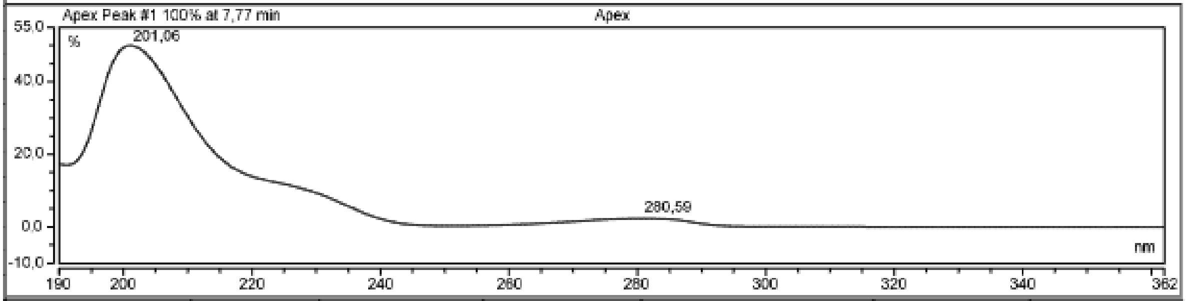

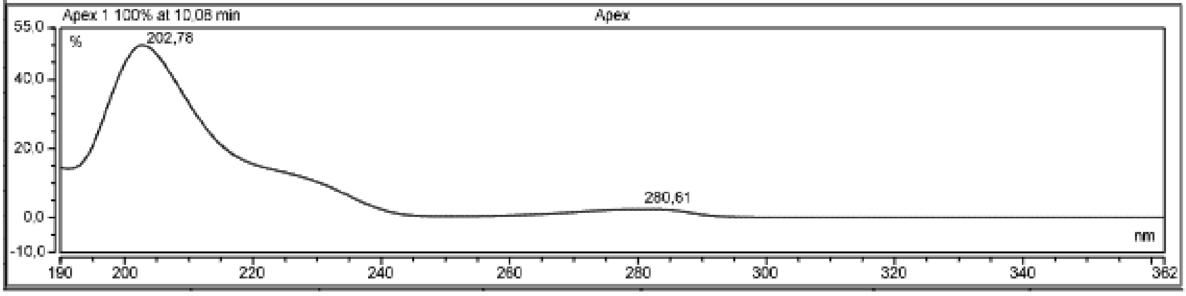

(*R,E*)-5-Fluoro-2-methyl-4-(pent-3-en-2-yl)phenol (2k) & (*S,E*)-3-Fluoro-6-methyl-2-(pent-3-en-2-yl)phenol (3k)

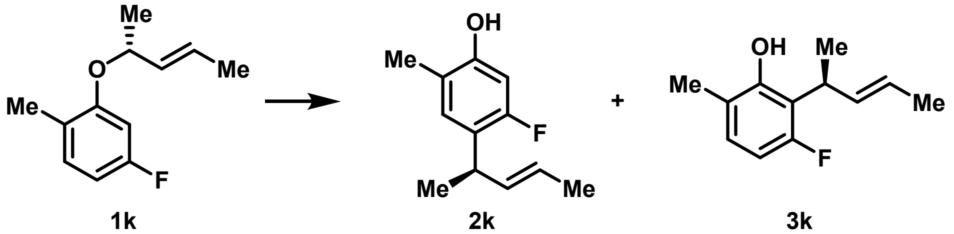

The title compounds were synthesized from **1k** (110 mg, 0.52 mmol) following **general procedure B**. The reaction was directly purified by column chromatography (petroleum ether/ethyl acetate 30:1) to provide the *para*-product **2k** as colorless oil in 50% yield (50 mg, 0.26 mmol) and the *ortho*-product **3k** as colorless oil in 44% yield (44 mg, 0.23 mmol).

(*R,E*)-5-Fluoro-2-methyl-4-(pent-3-en-2-yl)phenol (2k)

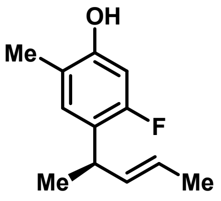

$[\alpha]^{20}$= -1.20 (c 0.30, CH_2Cl_2).

^{1}H NMR (400 MHz, $CDCl_3$) δ 6.90 (d, *J* = 8.4 Hz, 1H), 6.51 (d, *J* = 11.0 Hz, 1H), 5.67 – 5.53 (m, 1H), 5.45 (dq, *J* = 15.1, 6.3 Hz, 1H), 4.78 (s, 1H), 3.63 (p, *J* = 6.9 Hz, 1H), 2.20 (s, 3H), 1.67 (dd, *J* = 6.3, 1.6 Hz, 3H), 1.29 (d, *J* = 7.0 Hz, 3H).

^{13}C NMR (101 MHz, $CDCl_3$) δ 159.1 (d, *J* = 242.6 Hz), 152.6 (d, *J* = 11.6 Hz), 135.1, 129.9 (d, *J* = 6.5 Hz), 125.2, 124.0, 119.1, 102.8 (d, *J* = 25.9 Hz), 34.9, 20.8, 18.0, 15.3.

^{19}F NMR (377 MHz, $CDCl_3$) δ -121.2.

HRMS (ESI): exact mass calculated for $C_{12}H_{14}FO^-$ [(M - H)$^-$], 193.1034; found 193.1035.

85% *ee* (determined by chiral HPLC: Chiralcel® OJ-3 column, n-Heptane/EtOH = 99.5:0.5, 0.7 mL/min, λ = 287.3 nm, 25 °C), major enantiomer. t_r = 41.66 min, minor enantiomer. t_r = 53.58 min.

^1H NMR (400 MHz, $CDCl_3$)

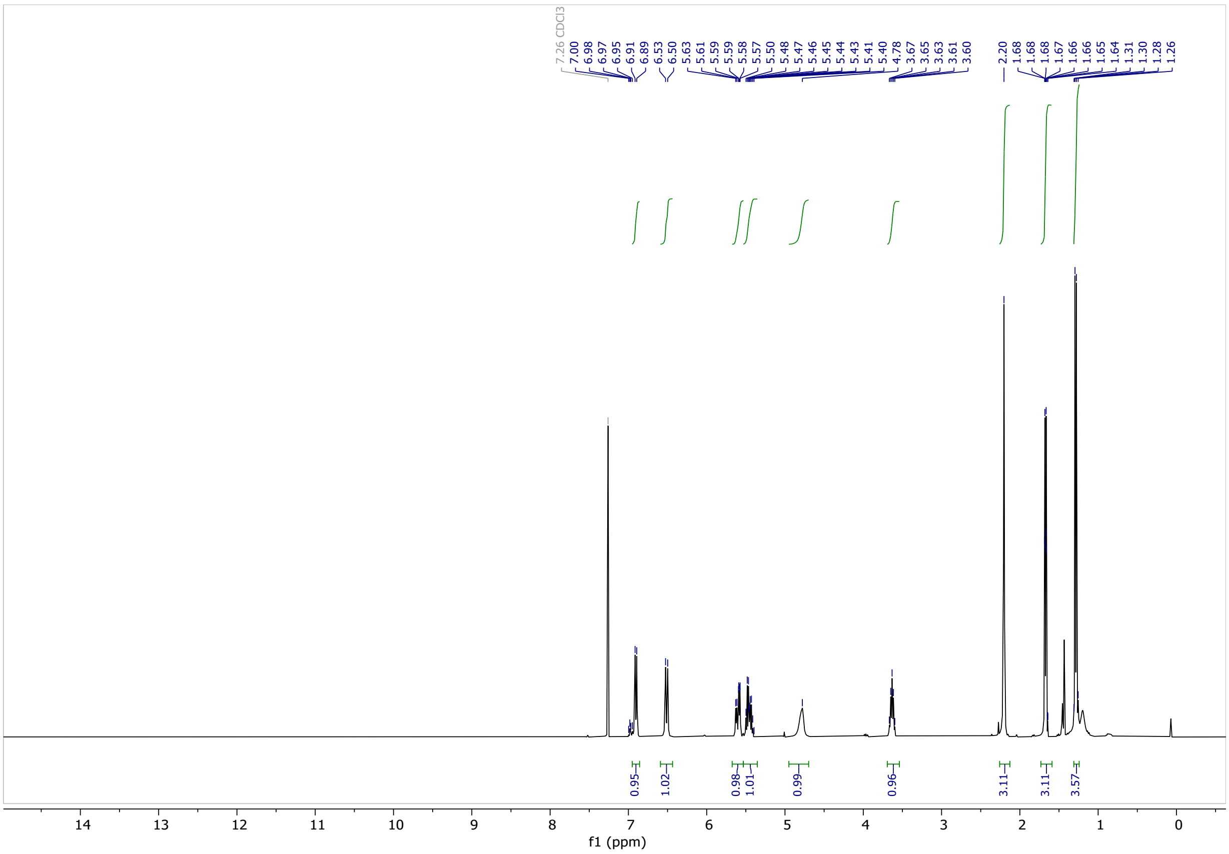

^{13}C NMR (101 MHz, $CDCl_3$)

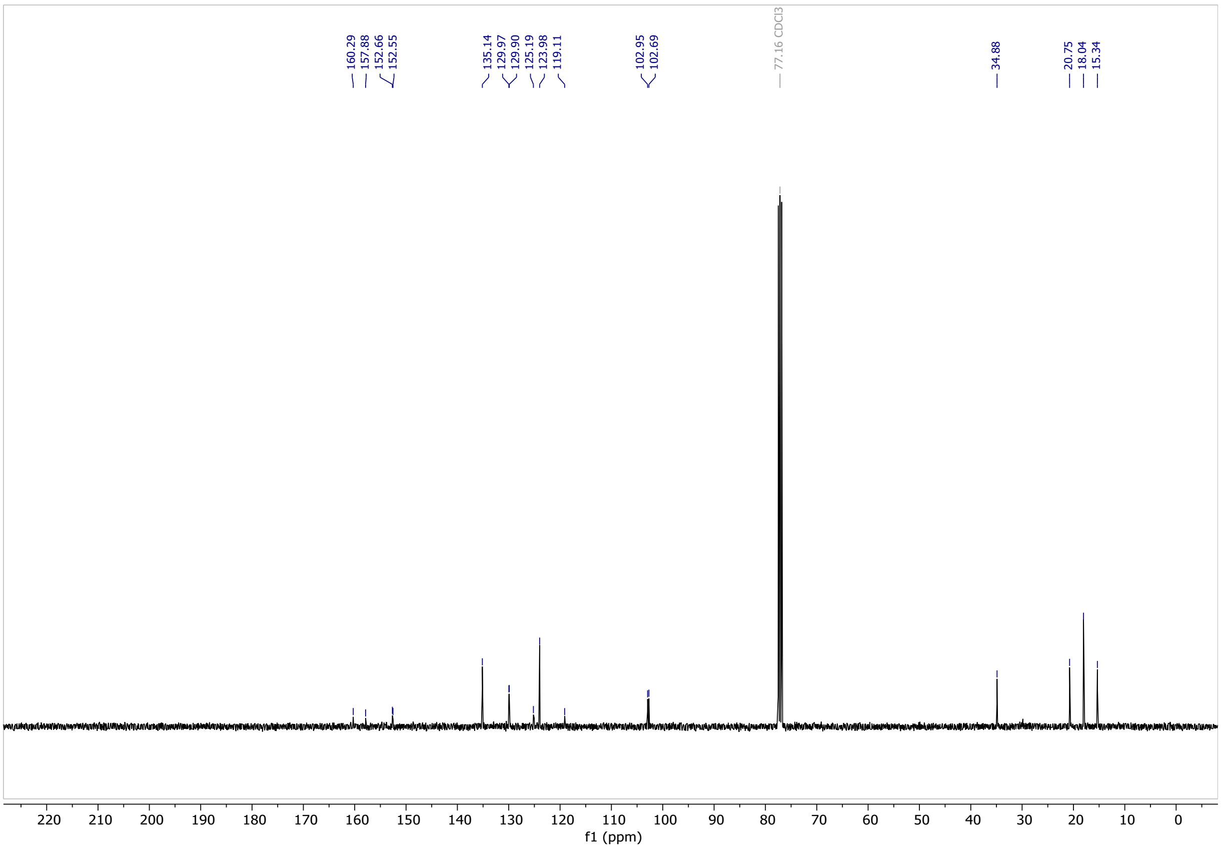

^{19}F NMR (377 MHz, $CDCl_3$)

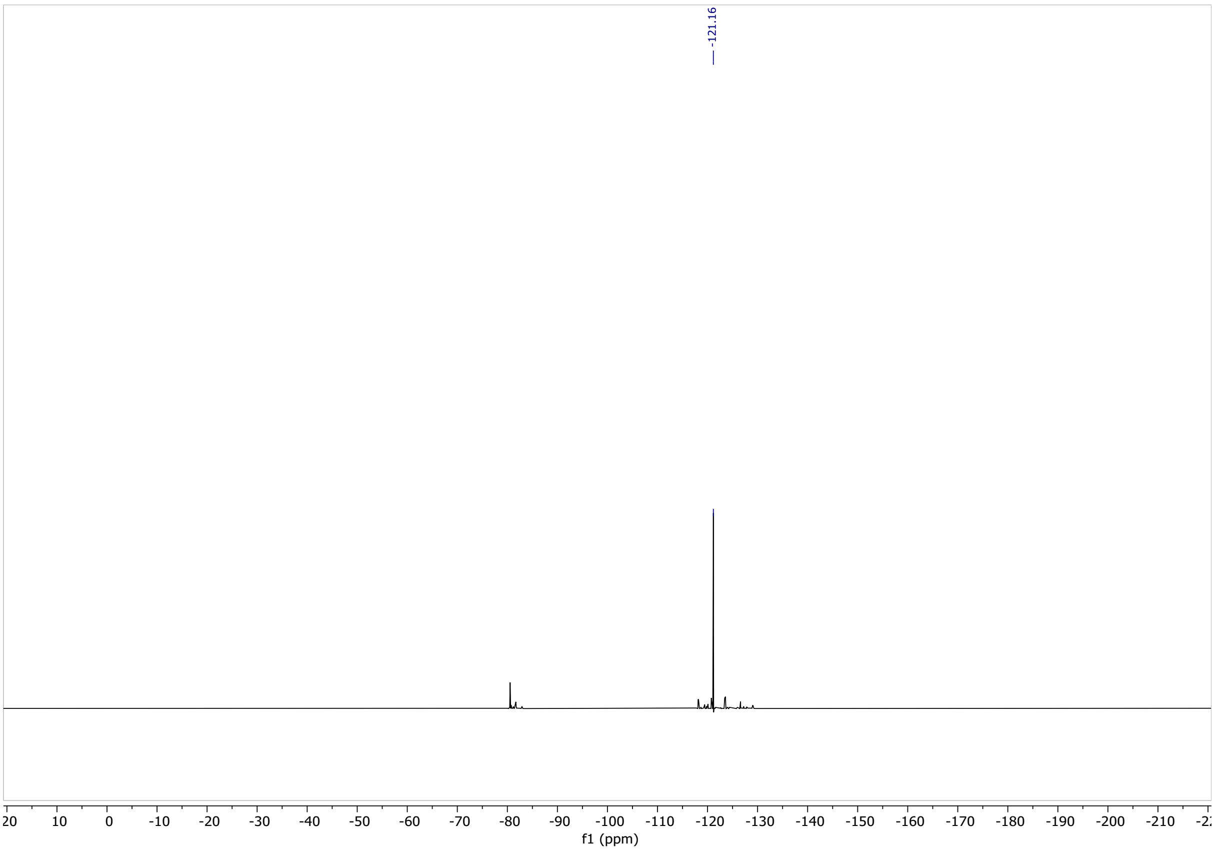

Chromatogram and Results

Instrument Method:	**Heptane_EtOH_99.5_0.5_0.7mlmin_25C_60min**	*B %:*	**0,0**
Column:	**OJ3**	*C %:*	**0,0**
Run Time (min):	**60,00**	*D %:*	**0,5**
Channel:	**UV_VIS_1**		
Wavelength:	**287,26**		

Chromatogram

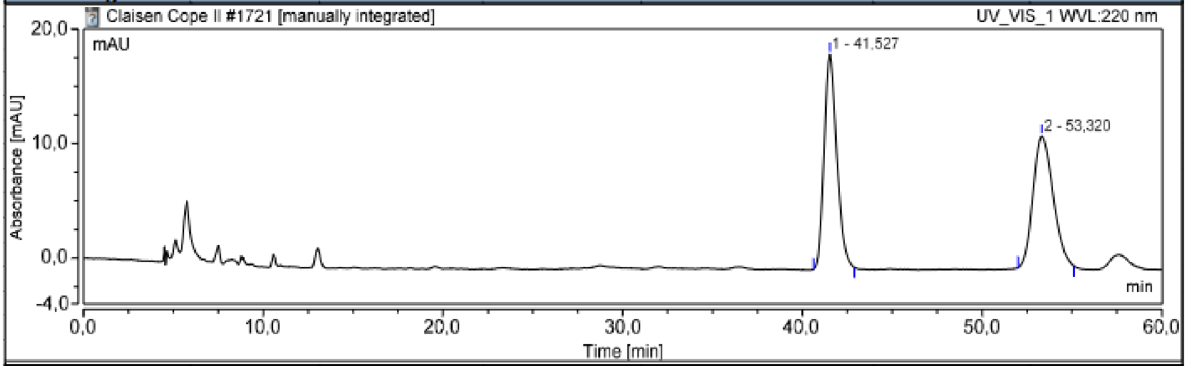

Integration Results

No.	Peak Name	Retention Time min	Area mAU*min	Height mAU	Relative Area %	Relative Height %
1		41,527	15,226	18,733	50,78	62,03
2		53,320	14,756	11,466	49,22	37,97
**Total:**			**29,981**	**30,200**	**100,00**	**100,00**

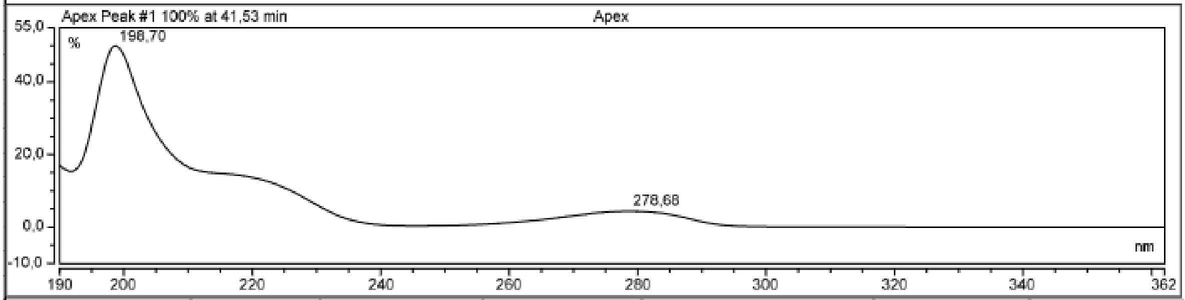

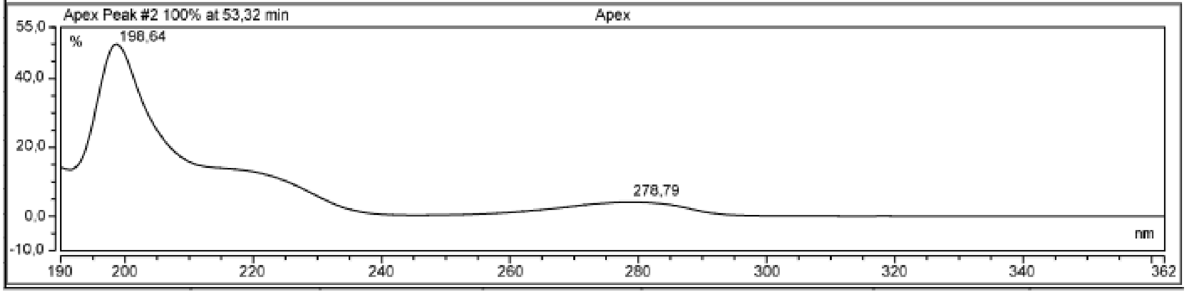

Chromatogram and Results

Instrument Method:	**Heptane_EtOH_99.5_0.5_0.7mlmin_25C_60min**	*B %:*	**0,0**
Column:	**OJ3**	*C %:*	**0,0**
Run Time (min):	**60,00**	*D %:*	**0,5**
Channel:	**UV_VIS_1**		
Wavelength:	**287,26**		

Chromatogram

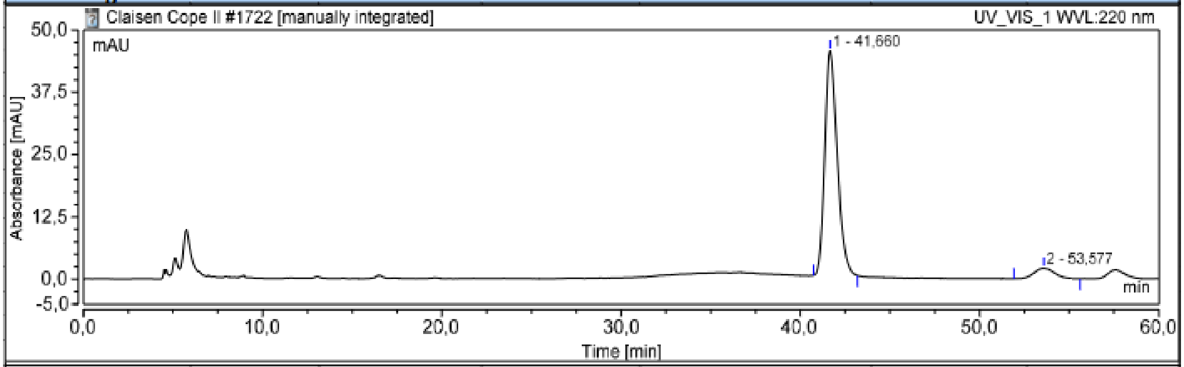

Integration Results

No.	Peak Name	Retention Time min	Area mAU*min	Height mAU	Relative Area %	Relative Height %
1		41,660	36,437	45,056	92,71	95,45
2		53,577	2,864	2,148	7,29	4,55
**Total:**			**39,302**	**47,205**	**100,00**	**100,00**

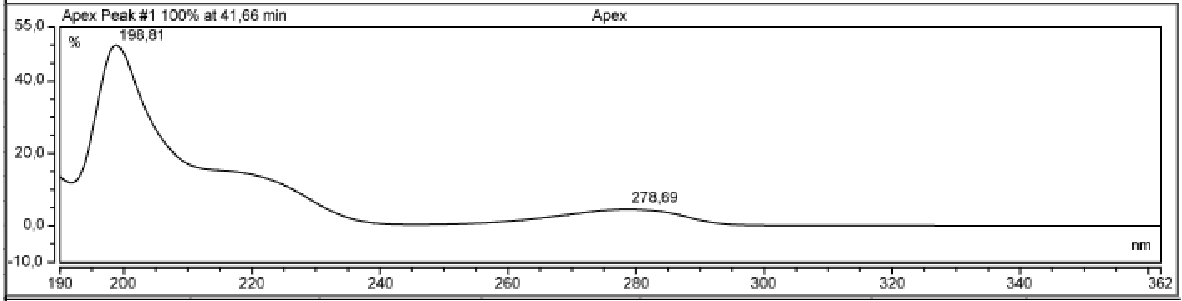

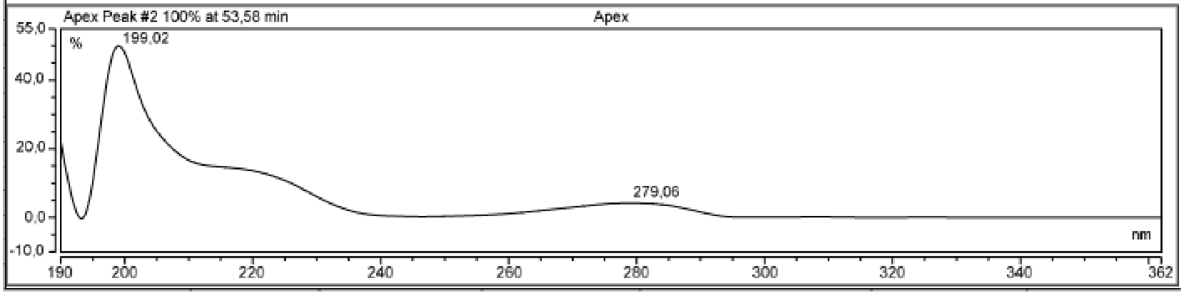

(*S,E*)-3-Fluoro-6-methyl-2-(pent-3-en-2-yl)phenol (3k)

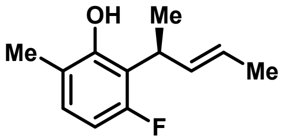

$[\alpha]^{20}$= - 20.81 (c 0.70, CH_2Cl_2).

^1H NMR (400 MHz, $CDCl_3$) δ 6.92 (t, *J* = 7.4 Hz, 1H), 6.54 (td, *J* = 9.4, 2.4 Hz, 1H), 5.95 – 5.71 (m, 3H), 4.04 – 3.92 (m, 1H), 2.16 (s, 3H), 1.86 – 1.74 (m, 3H), 1.39 (dd, *J* = 7.7, 2.4 Hz, 3H).

^{13}C NMR (101 MHz, $CDCl_3$) δ 159.2 (d, *J* = 241.1 Hz), 153.8 (d, *J* = 6.2 Hz), 134.1, 128.8 (d, *J* = 10.2 Hz), 127.1, 120.9 (d, *J* = 3.3 Hz), 117.5 (d, *J* = 17.0 Hz), 106.8 (d, *J* = 23.5 Hz), 31.3 (d, *J* = 4.6 Hz), 18.2, 17.6, 15.7.

^{19}F NMR (377 MHz, $CDCl_3$) δ -120.8.

HRMS (ESI): exact mass calculated for $C_{12}H_{14}FO^-$ [(M - H)$^-$], 193.1034; found 193.1032.

85% *ee* (determined by chiral HPLC: Chiralcel® OJ-3 column, n-Heptane/EtOH = 99:1, 0.7 mL/min, λ = 287.3 nm, 25 °C), major enantiomer. t_r = 12.56 min, minor enantiomer. t_r = 10.53 min.

^{1}H NMR (400 MHz, $CDCl_3$)

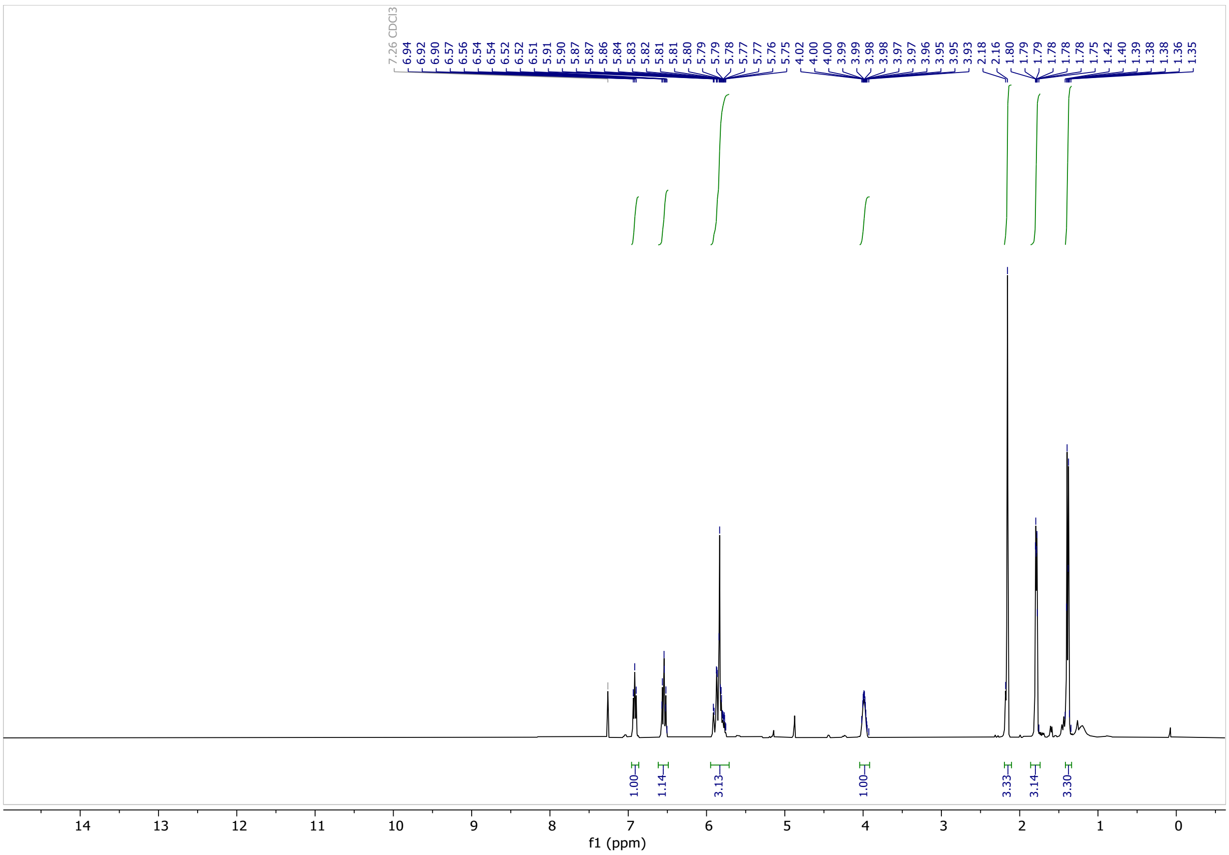

^{13}C NMR (101 MHz, $CDCl_3$)

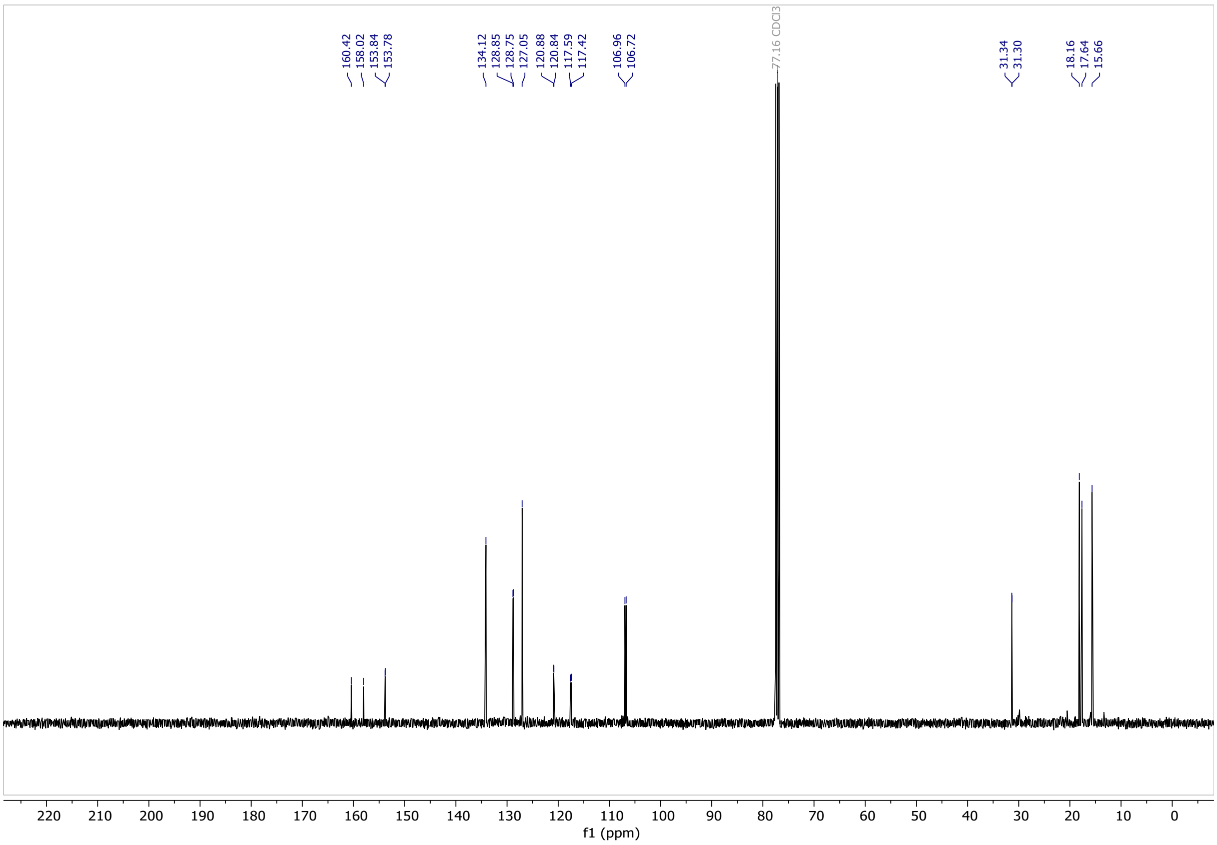

^{19}F NMR (377 MHz, $CDCl_3$)

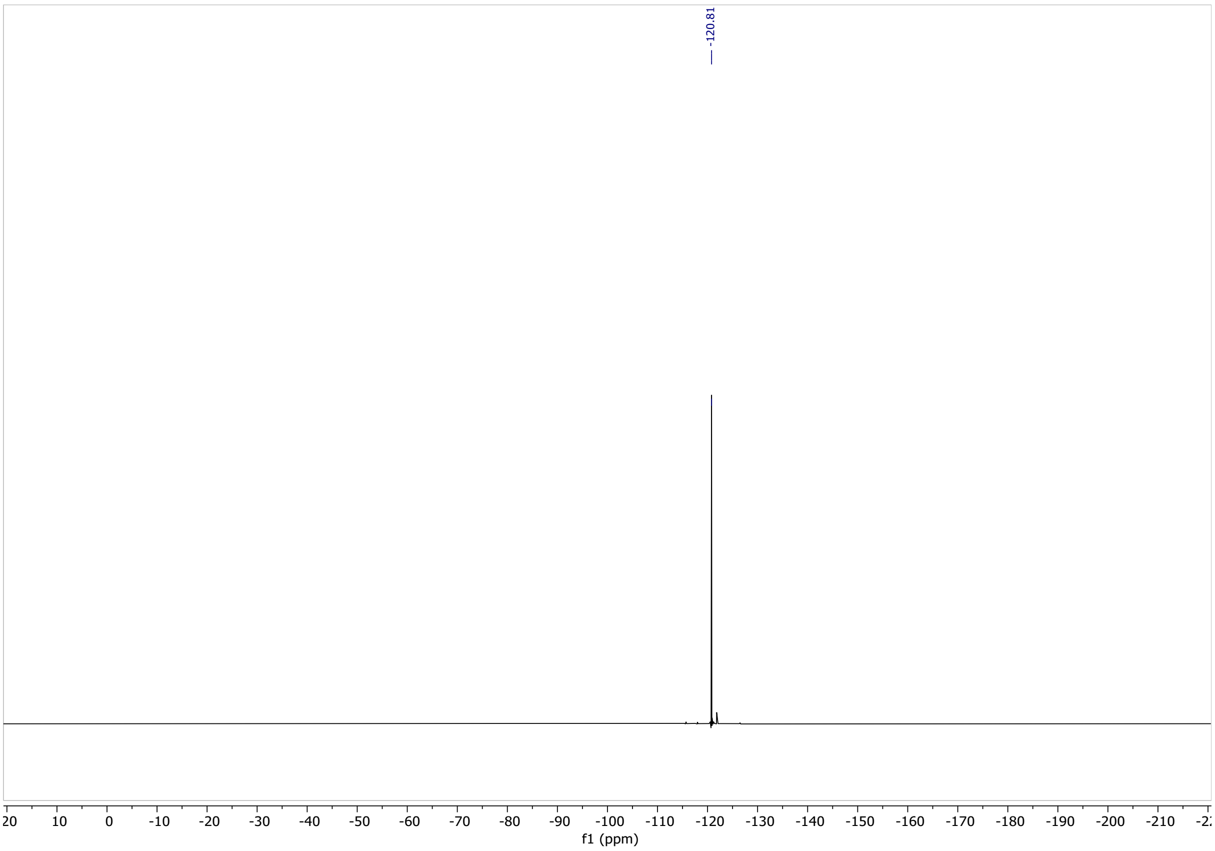

Chromatogram and Results

Instrument Method:	**Heptane_EtOH_99_1_0.7mlmin_25C_40min-MK**	*B %:*	**0,0**
Column:	**OJ3**	*C %:*	**0,0**
Run Time (min):	**40,00**	*D %:*	**1,0**
Channel:	**UV_VIS_1**		
Wavelength:	**287,26**		

Chromatogram

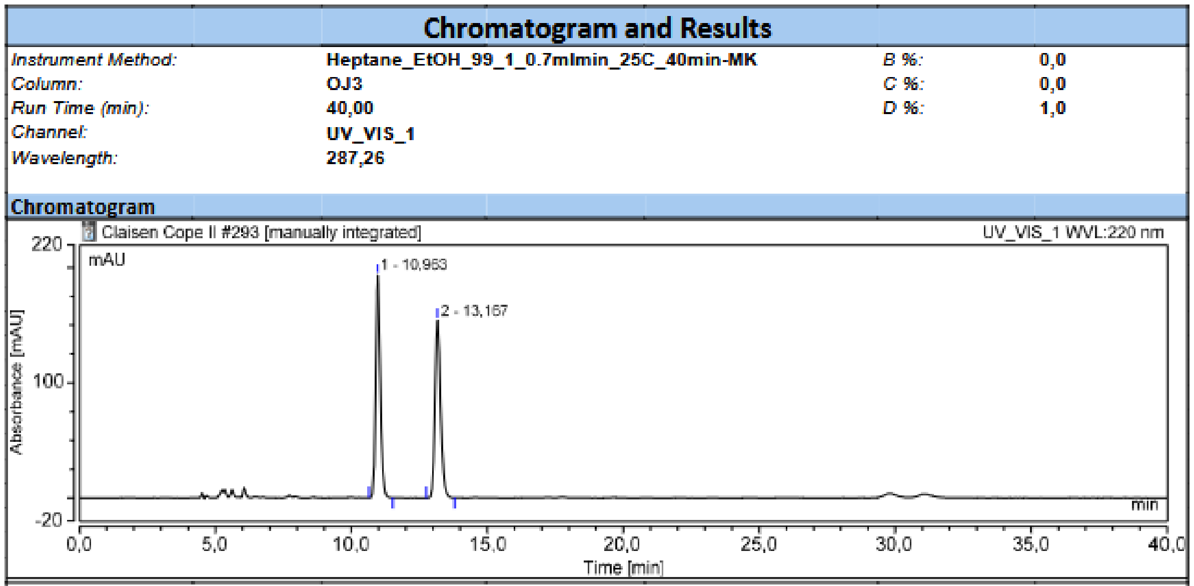

Integration Results

No.	Peak Name	Retention Time min	Area mAU*min	Height mAU	Relative Area %	Relative Height %
1		10,963	37,648	193,525	50,02	55,61
2		13,167	37,612	154,496	49,98	44,39
**Total:**			**75,260**	**348,021**	**100,00**	**100,00**

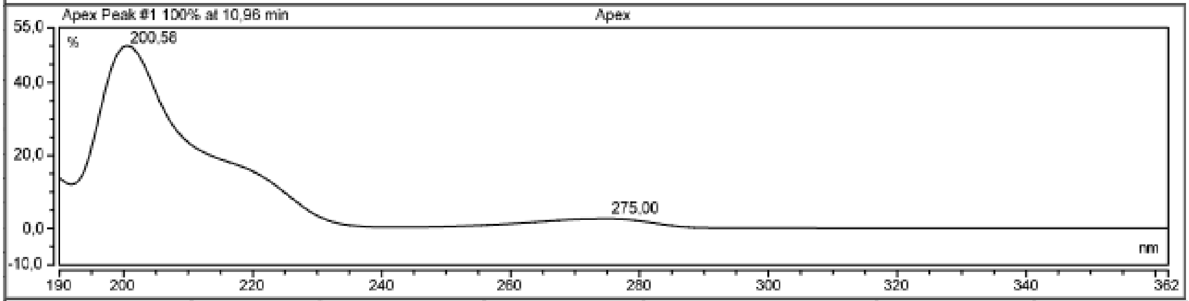

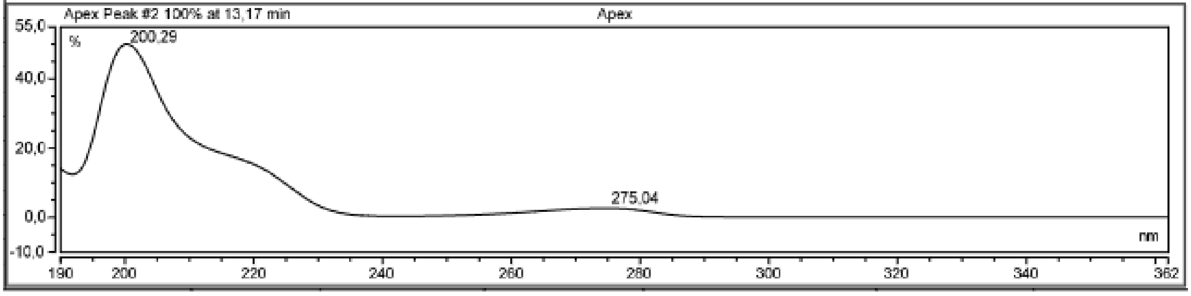

Chromatogram and Results

Instrument Method:	**Heptane_EtOH_99_1_0.7mlmin_25C_25min-MK**	*B %:*	**0,0**
Column:	**OJ3**	*C %:*	**0,0**
Run Time (min):	**25,00**	*D %:*	**1,0**
Channel:	**UV_VIS_1**		
Wavelength:	**287,26**		

Chromatogram

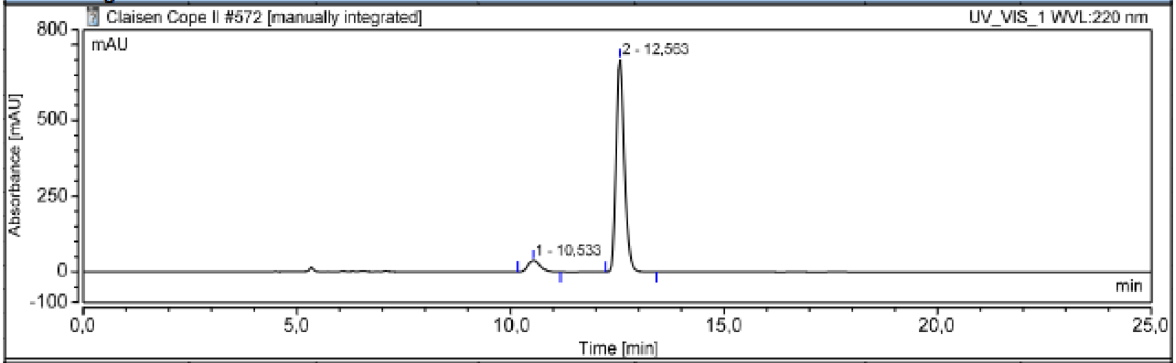

Integration Results

No.	Peak Name	Retention Time<br>min	Area<br>mAU*min	Height<br>mAU	Relative Area<br>%	Relative Height<br>%
1		10,533	12,708	37,589	7,51	5,09
2		12,563	156,548	700,572	92,49	94,91
**Total:**			**169,257**	**738,161**	**100,00**	**100,00**

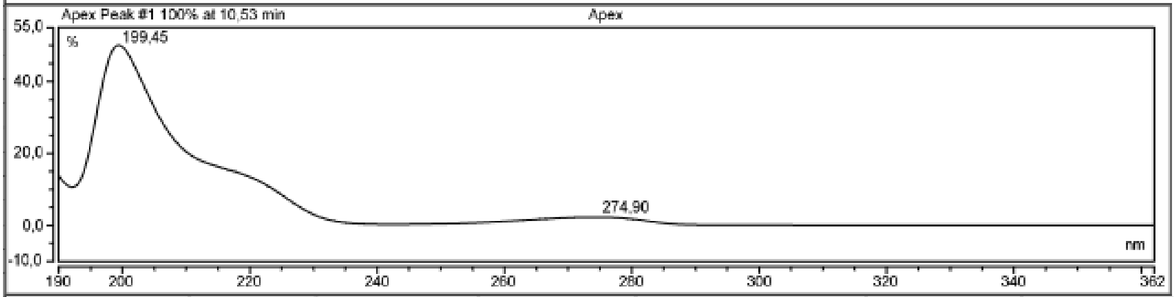

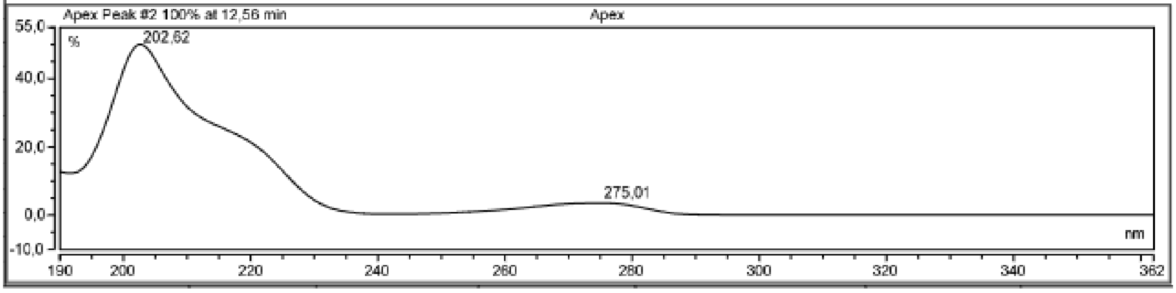

(*R,E*)-5-Chloro-2-methyl-4-(pent-3-en-2-yl)phenol (2l) & (*S,E*)-3-Chloro-6-methyl-2-(pent-3-en-2-yl)phenol (3l)

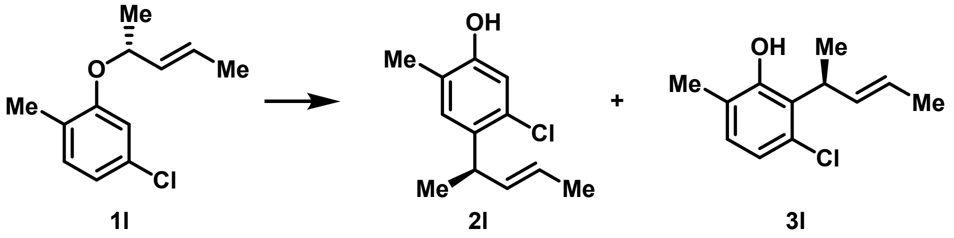

The title compounds were synthesized from **1l** (96 mg, 0.46 mmol) following **general procedure B**. The reaction was directly purified by column chromatography (petroleum ether/ethyl acetate 40:1 to 30:1) to provide the *para*-product **2l** as orange oil in 45% yield (43 mg, 0.20 mmol) and the *ortho*-product **3l** as yellow oil in 43% yield (41 mg, 0.20 mmol).

(*R,E*)-5-Chloro-2-methyl-4-(pent-3-en-2-yl)phenol (2l)

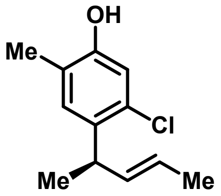

$[\alpha]^{20}$= + 7.48 (c 1.35, CH_2Cl_2).

^{1}H NMR (400 MHz, $CDCl_3$) δ 6.96 (s, 1H), 6.82 (t, *J* = 1.7 Hz, 1H), 5.66 – 5.54 (m, 1H), 5.54 – 5.36 (m, 1H), 4.79 (d, *J* = 14.3 Hz, 1H), 3.82 (p, *J* = 6.7 Hz, 1H), 2.22 (s, 3H), 1.69 (dd, *J* = 6.3, 1.7 Hz, 3H), 1.27 (d, *J* = 7.0 Hz, 3H).

^{13}C NMR (101 MHz, $CDCl_3$) δ 152.5, 135.9, 135.0, 130.8, 130.1, 124.2, 122.9, 115.9, 37.4, 20.6, 18.1, 15.7.

HRMS (ESI): exact mass calculated for $C_{12}H_{14}ClO^-$ [(M - H)$^-$], 209.0739 (100.0%), 211.0709 (32.0%); found 209.0738 (100.0%), 211.0706 (32.7%).

88% *ee* (determined by chiral HPLC: Chiralcel® OJ-3 column, n-Heptane/EtOH = 99.5:0.5, 0.5 mL/min, λ = 287.3 nm, 25 °C), major enantiomer. t_r = 51.38 min, minor enantiomer. t_r = 53.79 min.

^{1}H NMR (400 MHz, $CDCl_3$)

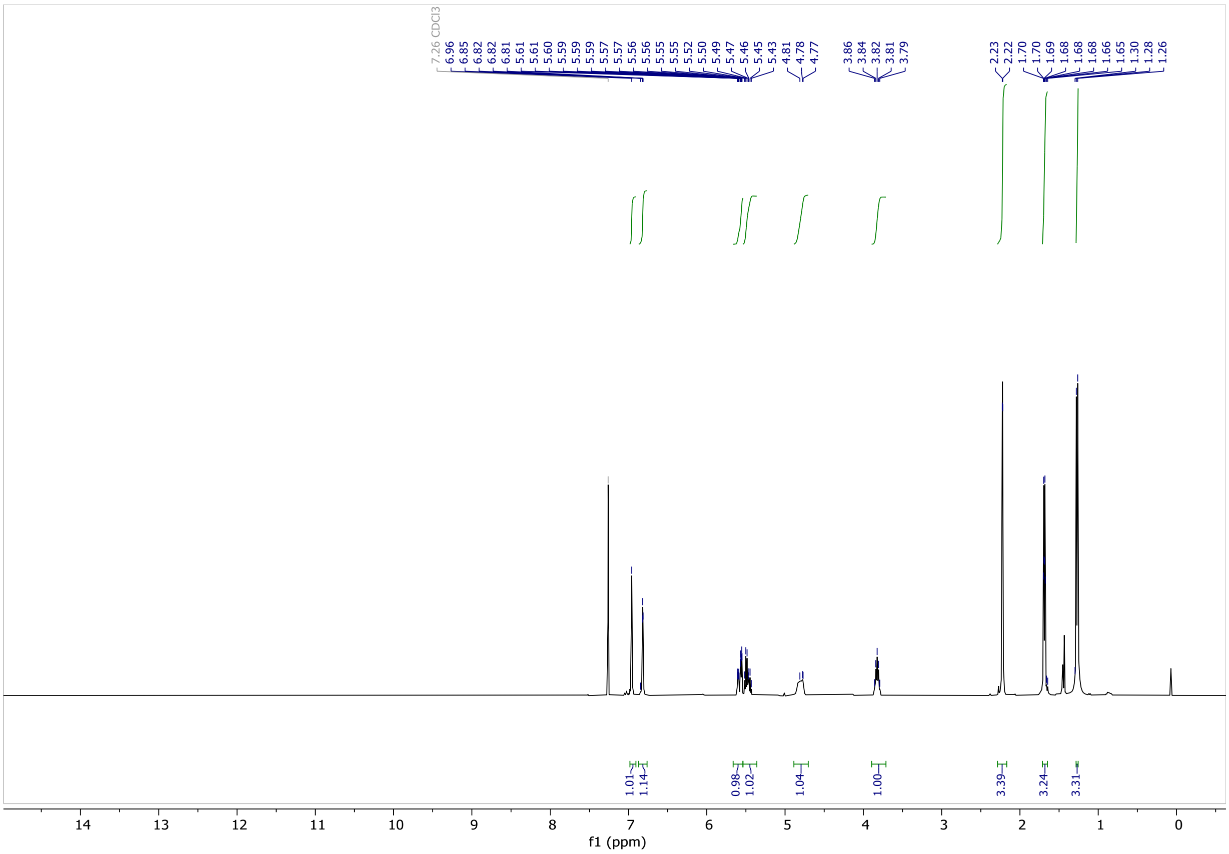

^{13}C NMR (101 MHz, $CDCl_3$)

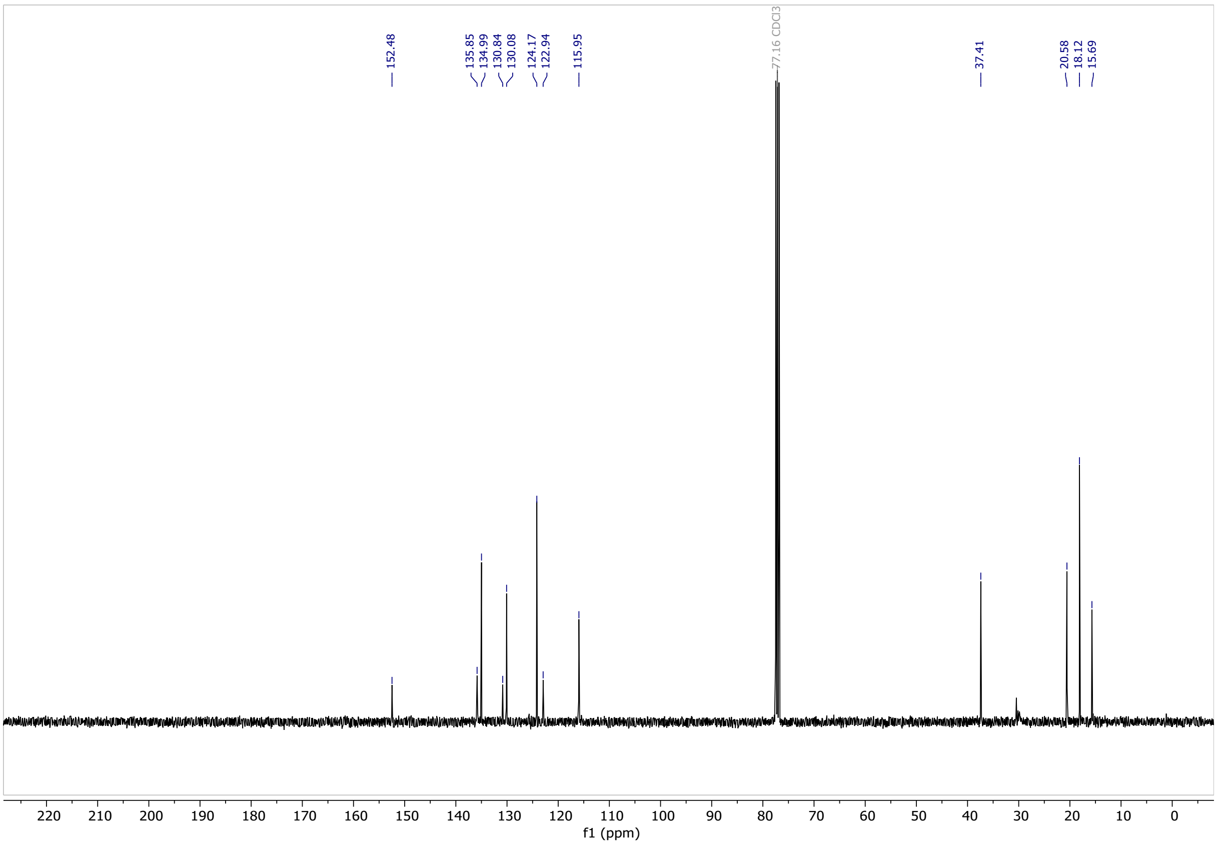

152.48
135.85
134.99
130.84
130.08
124.17
122.94
115.95
77.16 CDCl3
37.41
20.58
18.12
15.69
220 210 200 190 180 170 160 150 140 130 120 110 100 90 80 70 60 50 40 30 20 10 0
f1 (ppm)

Chromatogram and Results

Instrument Method:	**Heptane_EtOH_99.5_0.5_0.5mlmin_25C_60min**	*B %:*	**0,0**
Column:	**OJ3**	*C %:*	**0,0**
Run Time (min):	**60,00**	*D %:*	**0,5**
Channel:	**UV_VIS_1**		
Wavelength:	**287,26**		

Chromatogram

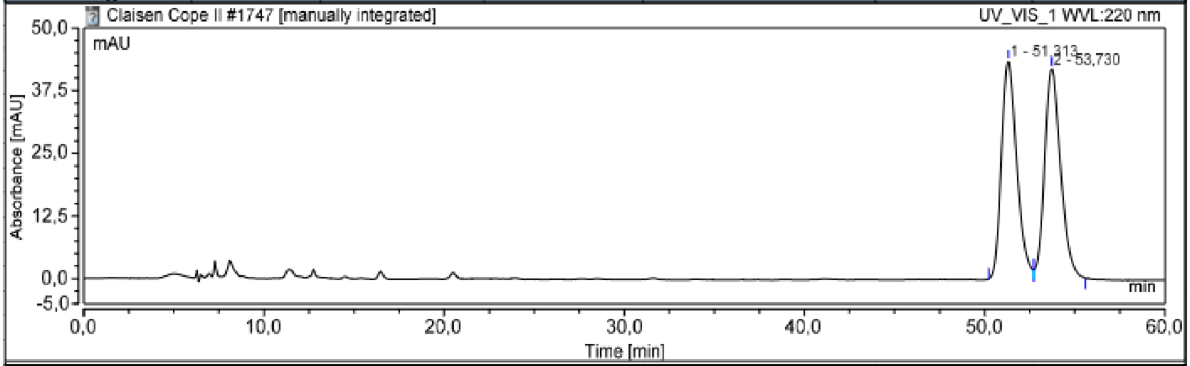

Integration Results

No.	Peak Name	Retention Time<br>min	Area<br>mAU*min	Height<br>mAU	Relative Area<br>%	Relative Height<br>%
1		51,313	42,826	43,452	49,78	50,90
2		53,730	43,204	41,919	50,22	49,10
**Total:**			**86,030**	**85,370**	**100,00**	**100,00**

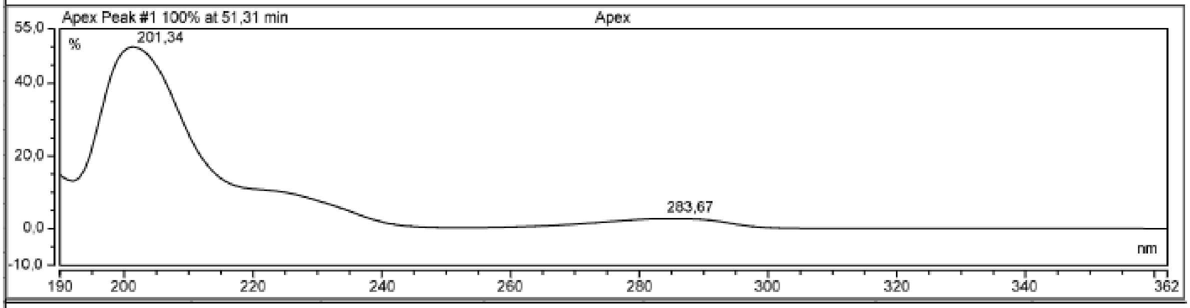

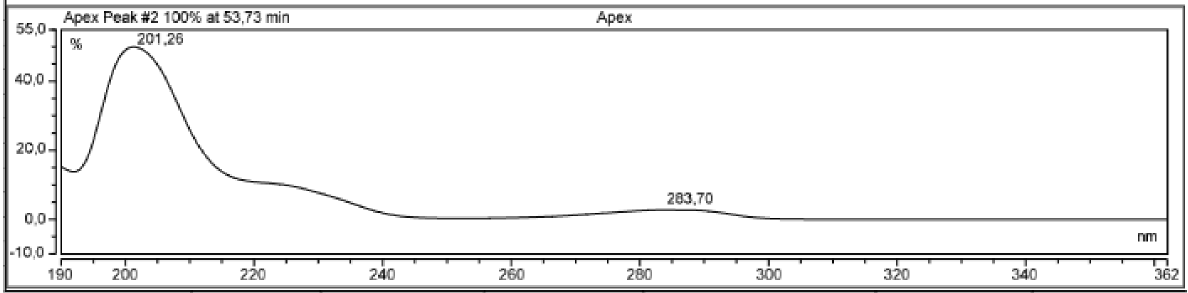

Chromatogram and Results

Instrument Method:	**Heptane_EtOH_99.5_0.5_0.5mlmin_25C_60min**	*B %:*	**0,0**
Column:	**OJ3**	*C %:*	**0,0**
Run Time (min):	**60,00**	*D %:*	**0,5**
Channel:	**UV_VIS_1**		
Wavelength:	**287,26**		

Chromatogram

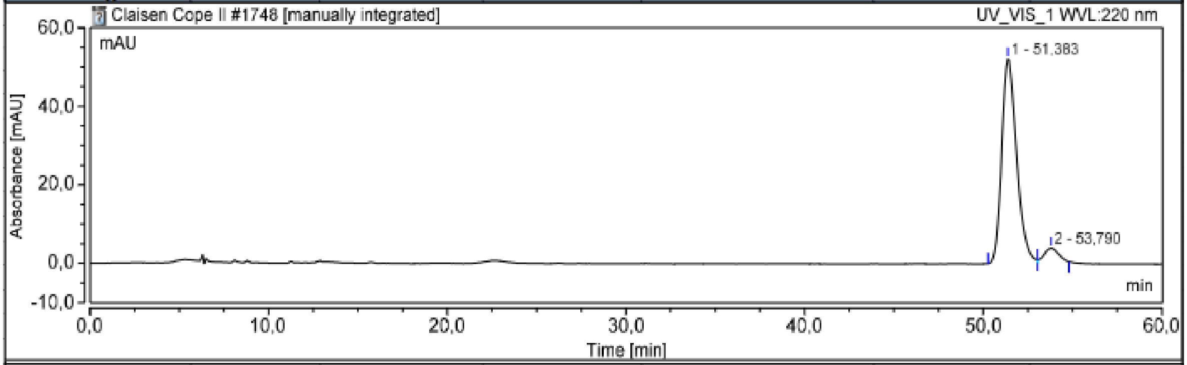

Integration Results

No.	Peak Name	Retention Time min	Area mAU*min	Height mAU	Relative Area %	Relative Height %
1		51,383	50,246	52,129	93,78	93,55
2		53,790	3,335	3,594	6,22	6,45
**Total:**			**53,580**	**55,723**	**100,00**	**100,00**

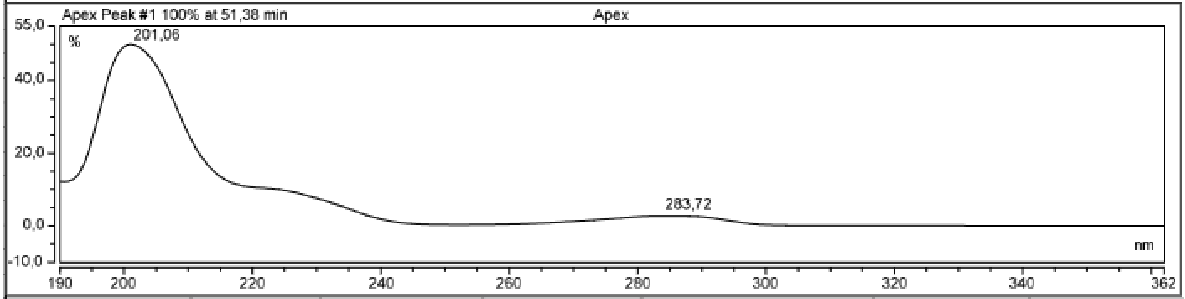

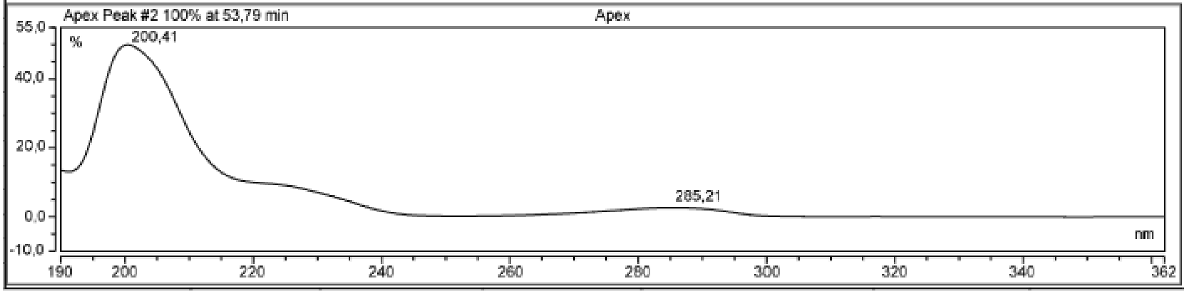

(*S,E*)-3-Chloro-6-methyl-2-(pent-3-en-2-yl)phenol (3l)

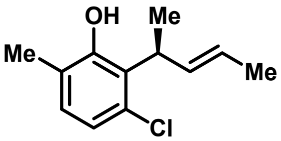

$[\alpha]^{20}$= -35.52 (c 0.75, CH_2Cl_2).

^{1}H NMR (400 MHz, $CDCl_3$) δ 6.91 (dt, *J* = 8.0, 0.7 Hz, 1H), 6.86 (d, *J* = 8.1 Hz, 1H), 6.16 (s, 1H), 5.96 – 5.79 (m, 2H), 4.29 – 4.14 (m, 1H), 2.15 (d, *J* = 0.7 Hz, 3H), 1.87 – 1.78 (m, 3H), 1.37 (d, *J* = 7.2 Hz, 3H).

^{13}C NMR (101 MHz, $CDCl_3$) δ 154.3, 134.0, 131.2, 129.4, 127.6, 127.0, 124.8, 121.1, 36.0, 18.2, 16.2, 15.9.

HRMS (ESI): exact mass calculated for $C_{12}H_{14}ClO^-$ [$(M - H)^-$], 209.0739 (100.0%), 211.0709 (32.0%); found 209.0741 (100.0%), 211.0710 (32.6%).

85% *ee* (determined by chiral HPLC: Chiralpak® AS-H column, n-Hexane/IPA = 99.9:0.1, 0.3 mL/min, λ = 287.3 nm, 25 °C), major enantiomer. t_r = 15.37 min, minor enantiomer. t_r = 16.99 min.

^{1}H NMR (400 MHz, $CDCl_3$)

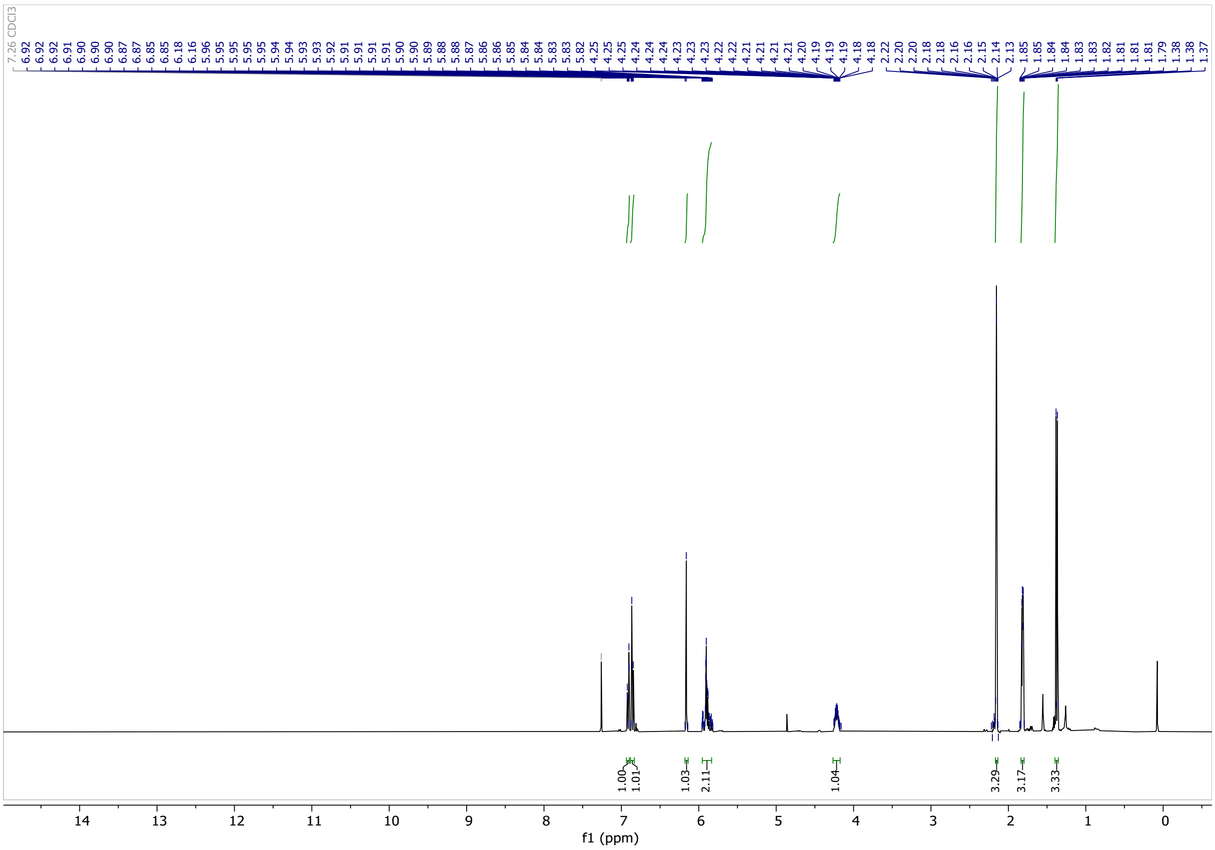

^{13}C NMR (101 MHz, $CDCl_3$)

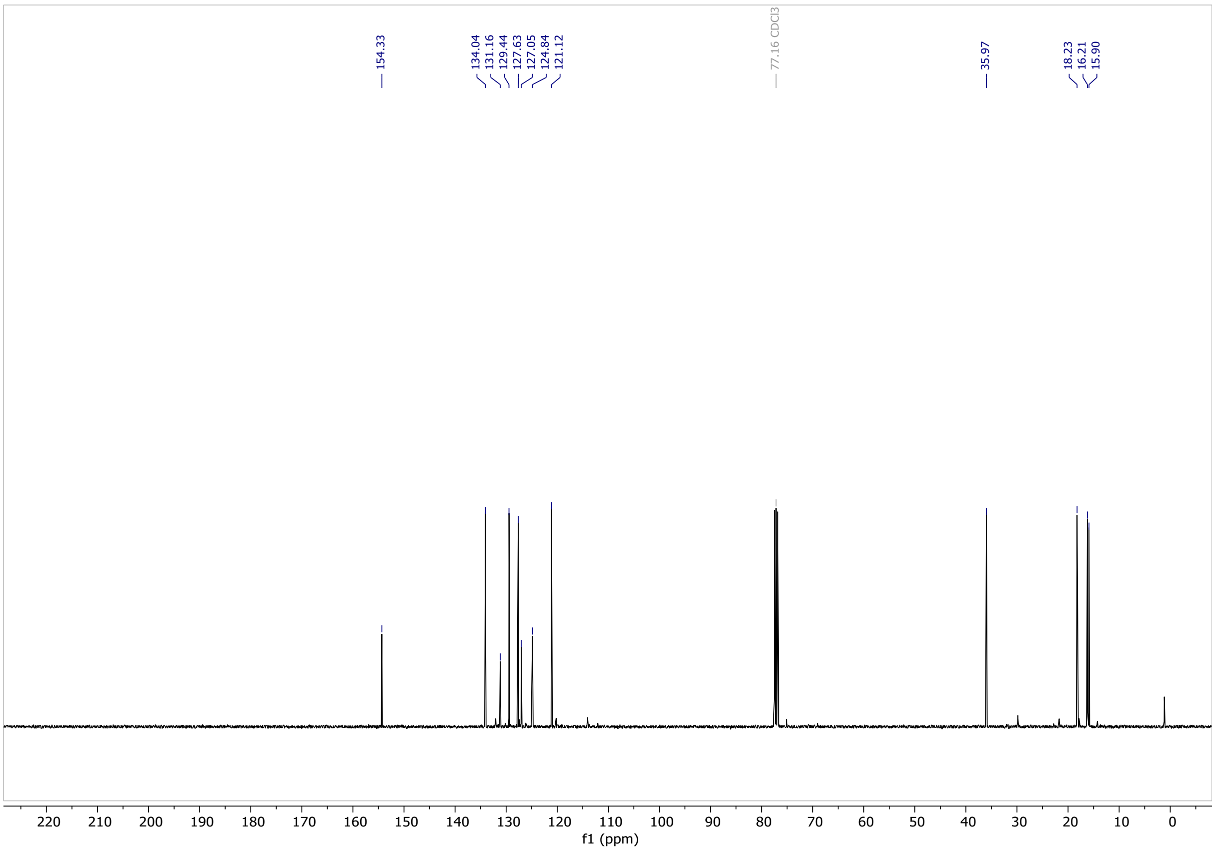

Chromatogram and Results

Instrument Method:	**Hexane_IPA_99.9_0.1_0.3mlmin_25C_20min**	*B %:*	**0,1**
Column:	**AS-H**	*C %:*	**99,9**
Run Time (min):	**20,00**	*D %:*	**0,0**
Channel:	**UV_VIS_1**		
Wavelength:	**287,26**		

Chromatogram

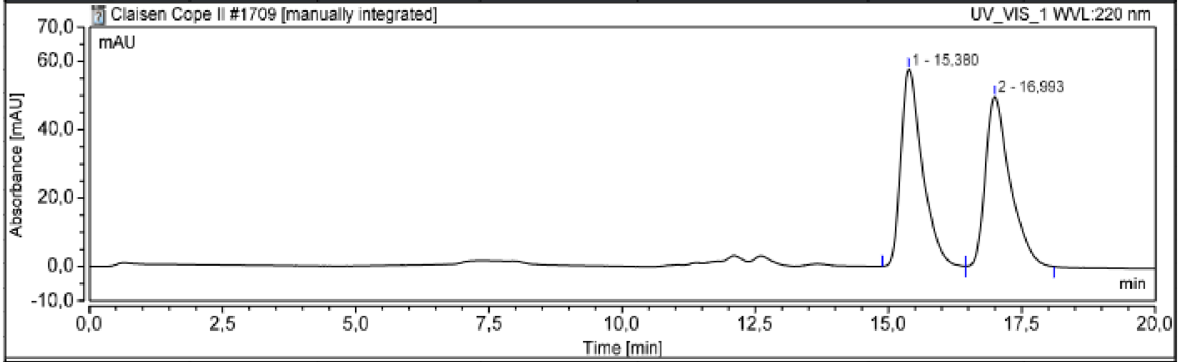

Integration Results

No.	Peak Name	Retention Time min	Area mAU*min	Height mAU	Relative Area %	Relative Height %
1		15,380	26,965	57,665	50,45	53,75
2		16,993	26,480	49,627	49,55	46,25
**Total:**			**53,445**	**107,293**	**100,00**	**100,00**

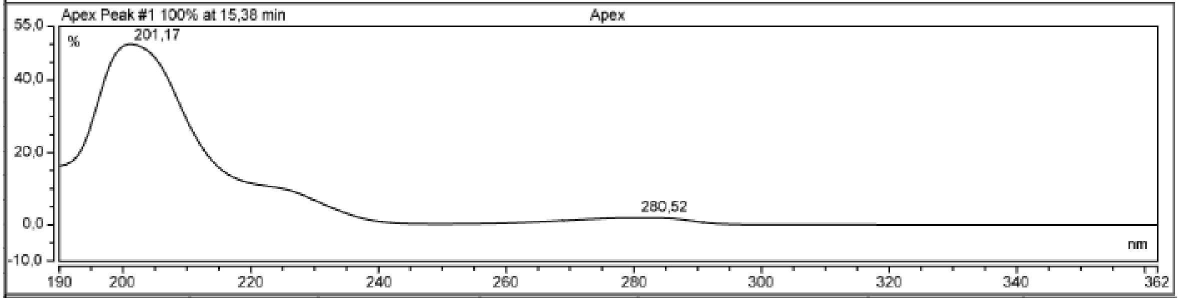

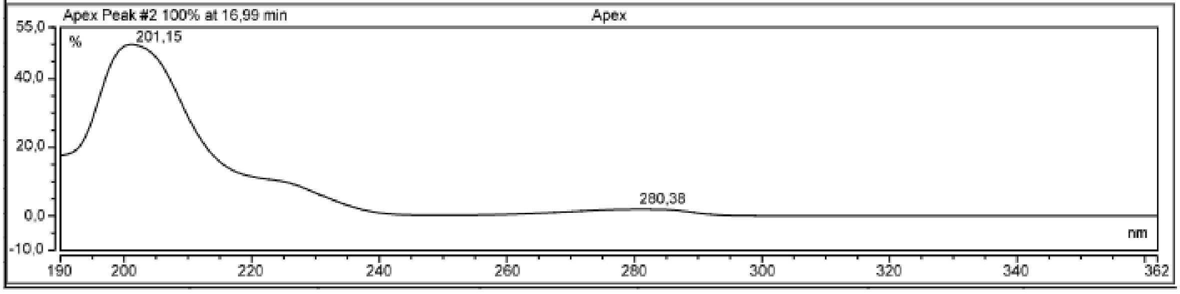

Chromatogram and Results

Instrument Method:	**Hexane_IPA_99.9_0.1_0.3mlmin_25C_20min**	*B %:*	**0,1**
Column:	**AS-H**	*C %:*	**99,9**
Run Time (min):	**20,00**	*D %:*	**0,0**
Channel:	**UV_VIS_1**		
Wavelength:	**287,26**		

Chromatogram

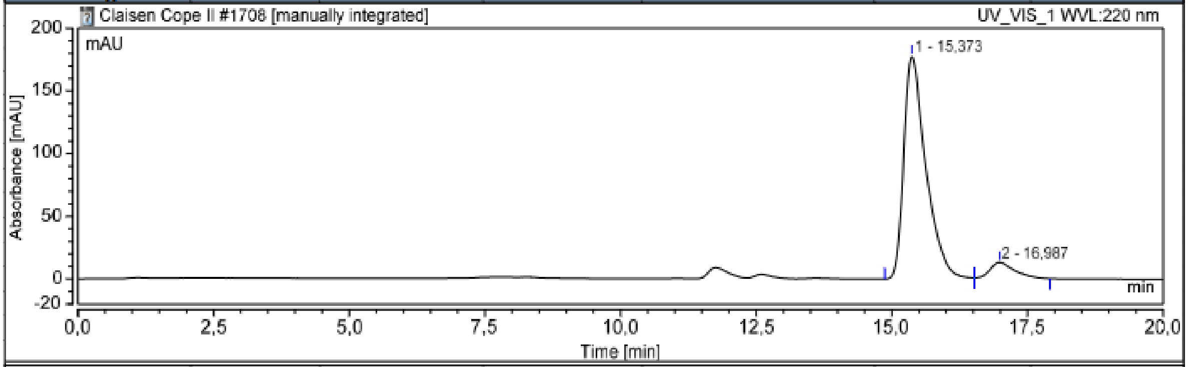

Integration Results

No.	Peak Name	Retention Time min	Area mAU*min	Height mAU	Relative Area %	Relative Height %
1		15,373	84,048	177,749	92,30	93,11
2		16,987	7,013	13,156	7,70	6,89
**Total:**			**91,062**	**190,905**	**100,00**	**100,00**

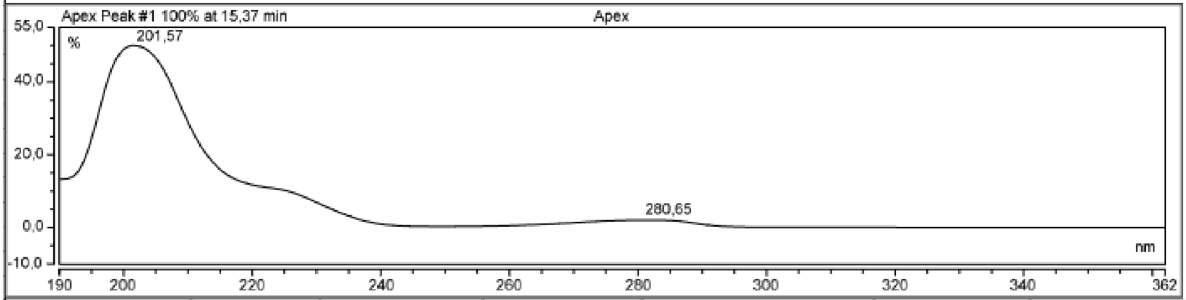

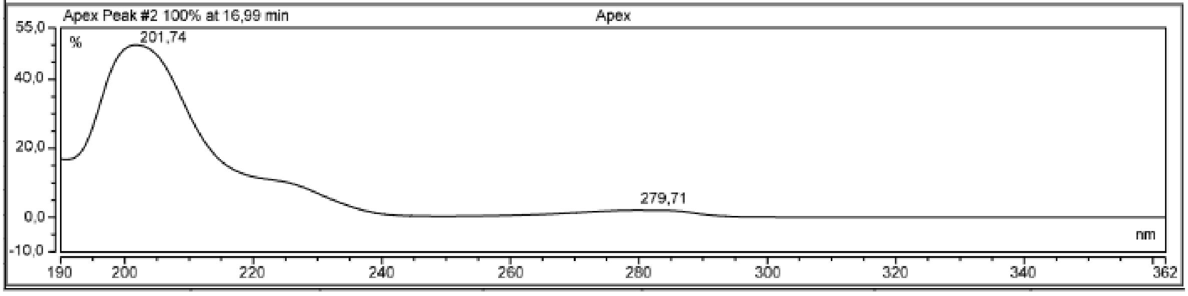

(*R,E*)-5-Bromo-2-methyl-4-(pent-3-en-2-yl)phenol (2m) & (*S,E*)-3-bromo-6-methyl-2-(pent-3-en-2-yl)phenol (3m)

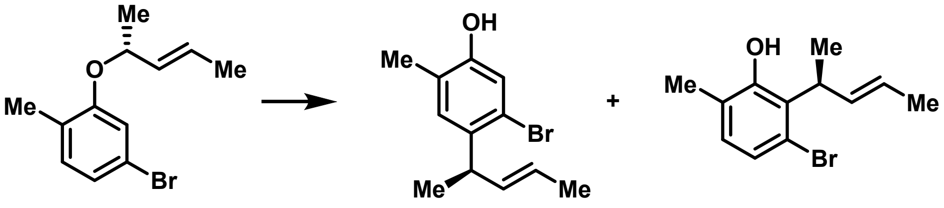

1m 2m 3m

The title compounds were synthesized from **1m** (100 mg, 0.39 mmol) following **general procedure B**. The reaction was directly purified by column chromatography (petroleum ether/ethyl acetate 30:1) to provide the *para*-product **2m** as colorless oil in 63% yield (63 mg, 0.25 mmol) and the *ortho*-product **3m** as colorless oil in 34% yield (34 mg, 0.13 mmol).

(*R,E*)-5-Bromo-2-methyl-4-(pent-3-en-2-yl)phenol (2m)

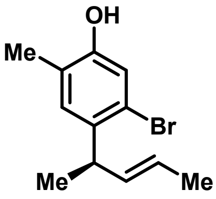

$[\alpha]^{20}$= + 15.03 (c 2.20, CH_2Cl_2).

^{1}H NMR (400 MHz, $CDCl_3$) δ 6.98 (s, 1H), 6.97 – 6.89 (m, 1H), 5.66 – 5.39 (m, 2H), 4.69 (s, 1H), 3.79 (ddt, *J* = 7.0, 5.7, 1.3 Hz, 1H), 2.19 (s, 3H), 1.69 (dt, *J* = 6.0, 1.3 Hz, 3H), 1.30 – 1.22 (m, 3H).

^{13}C NMR (101 MHz, $CDCl_3$) δ 152.6, 137.4, 135.0, 130.1, 129.9, 124.2, 121.0, 119.0, 39.9, 20.7, 18.1, 15.8.

HRMS (ESI): exact mass calculated for $C_{12}H_{14}BrO^-$ [(M - H)$^-$], 253.0234 (100.0%), 255.0213 (97.3%); found 253.0234 (100.0%), 255.0213 (96.0%).

85% *ee* (determined by chiral HPLC: Chiralcel® OJ-3 column, n-Heptane/EtOH = 99.5:0.5, 0.7 mL/min, λ = 287.3 nm, 25 °C), major enantiomer. t_r = 45.27 min, minor enantiomer. t_r = 48.13 min

^{1}H NMR (400 MHz, $CDCl_3$)

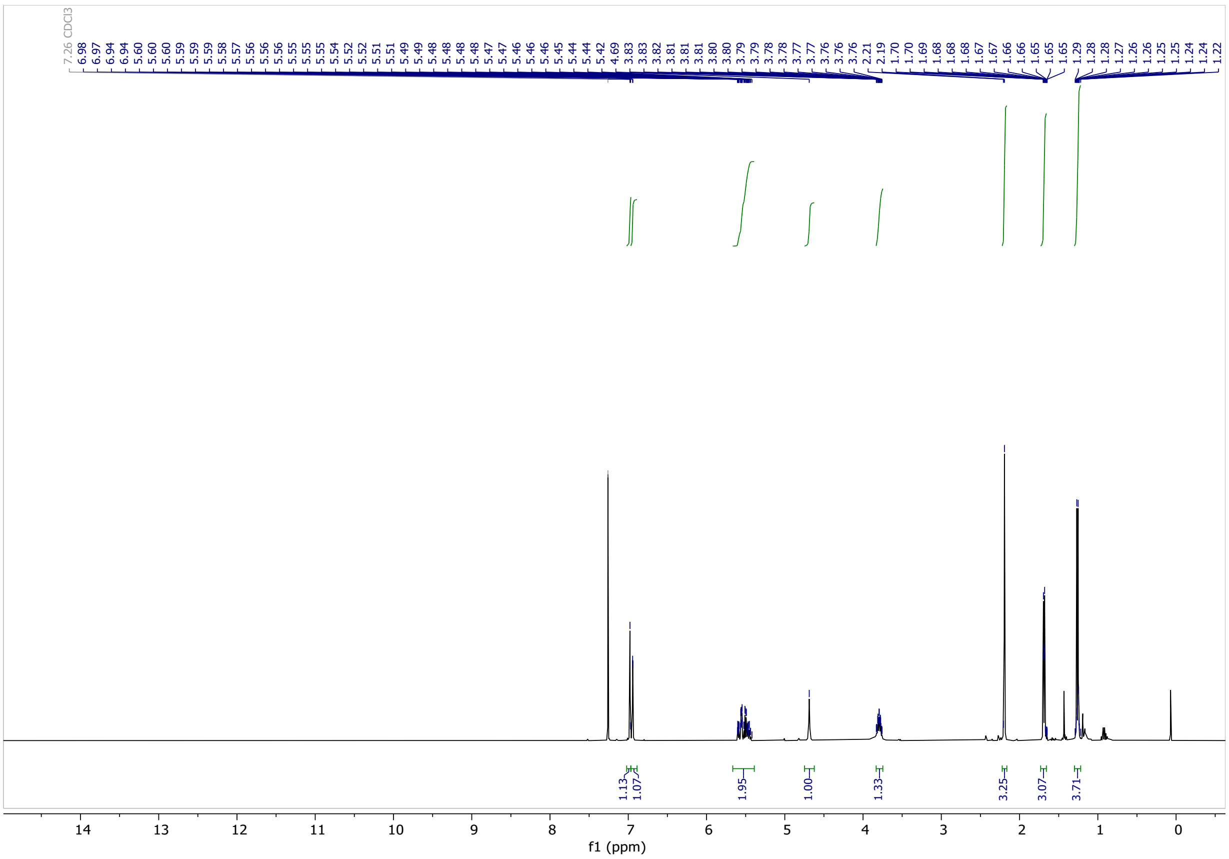

^{13}C NMR (101 MHz, $CDCl_3$)

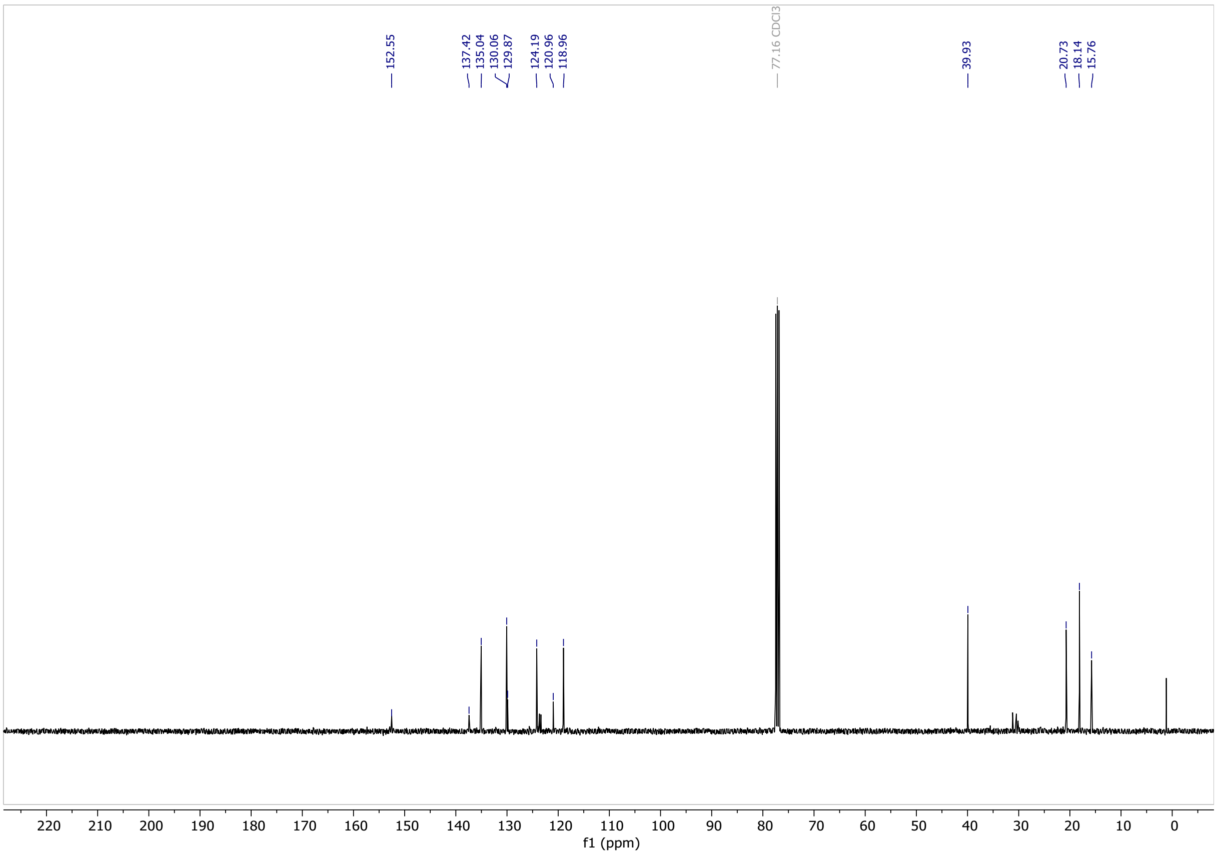

152.55
137.42
135.04
130.06
129.87
124.19
120.96
118.96
77.16 CDCl3
39.93
20.73
18.14
15.76
220 210 200 190 180 170 160 150 140 130 120 110 100 90 80 70 60 50 40 30 20 10 0
f1 (ppm)

Chromatogram and Results

Instrument Method:	**Heptane_EtOH_99.5_0.5_0.7mlmin_25C_60min**	*B %:*	**0,0**
Column:	**OJ3**	*C %:*	**0,0**
Run Time (min):	**60,00**	*D %:*	**0,5**
Channel:	**UV_VIS_1**		
Wavelength:	**287,26**		

Chromatogram

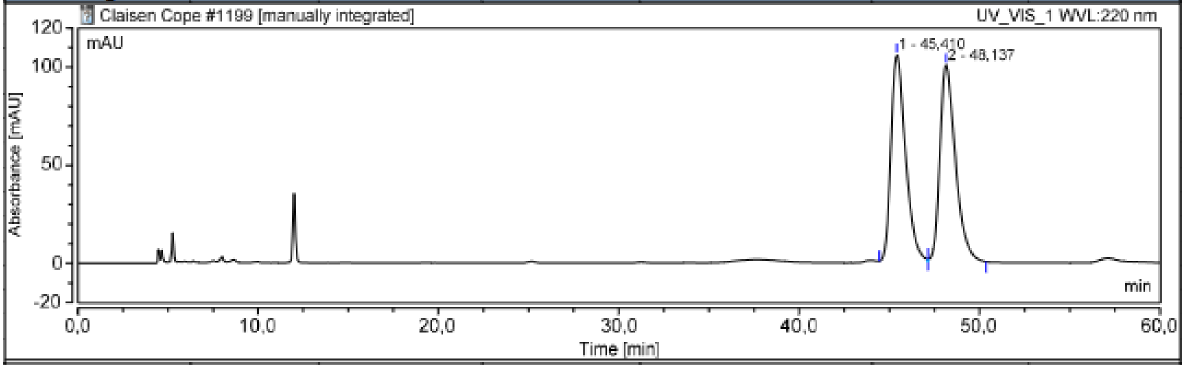

Integration Results

No.	Peak Name	Retention Time min	Area mAU*min	Height mAU	Relative Area %	Relative Height %
1		45,410	99,124	105,362	49,70	51,23
2		48,137	100,322	100,318	50,30	48,77
**Total:**			**199,446**	**205,680**	**100,00**	**100,00**

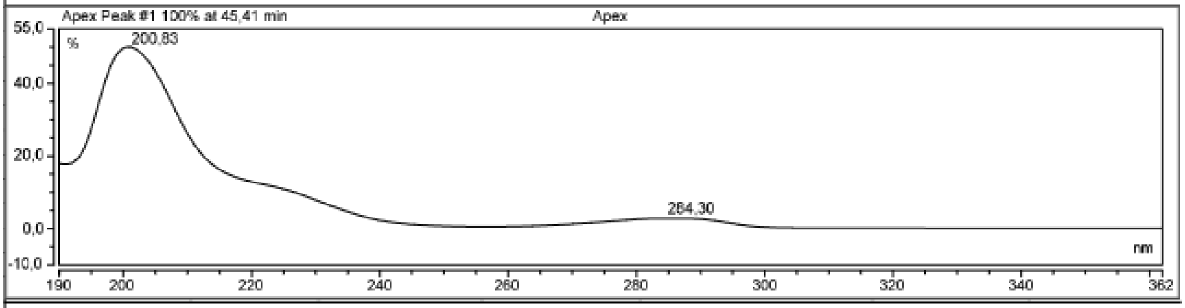

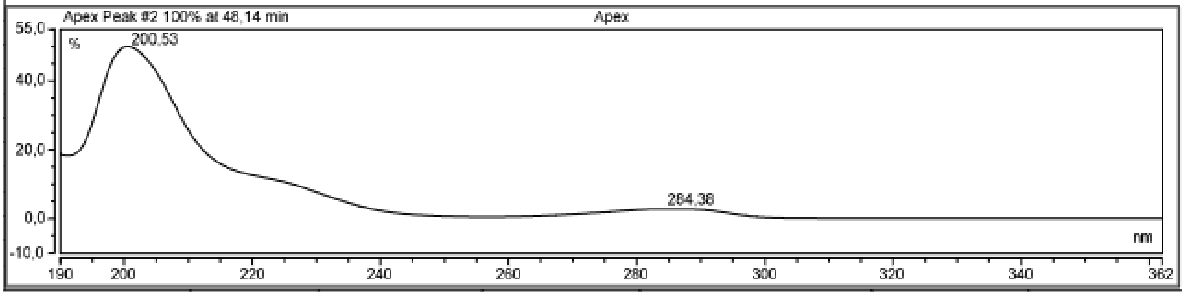

Chromatogram and Results

Instrument Method:	**Heptane_EtOH_99.5_0.5_0.7mlmin_25C_60min**	*B %:*	**0,0**
Column:	**OJ3**	*C %:*	**0,0**
Run Time (min):	**60,00**	*D %:*	**0,5**
Channel:	**UV_VIS_1**		
Wavelength:	**287,26**		

Chromatogram

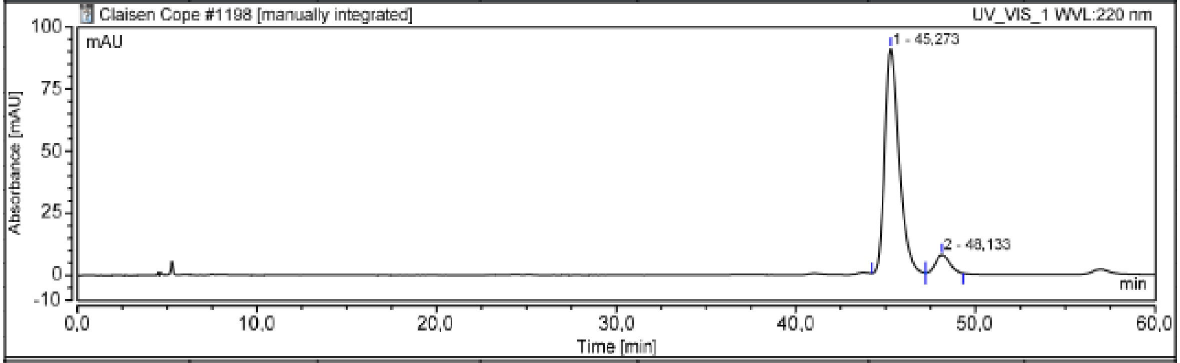

Integration Results

No.	Peak Name	Retention Time min	Area mAU*min	Height mAU	Relative Area %	Relative Height %
1		45,273	83,692	90,430	92,46	92,34
2		48,133	6,828	7,498	7,54	7,66
**Total:**			**90,520**	**97,928**	**100,00**	**100,00**

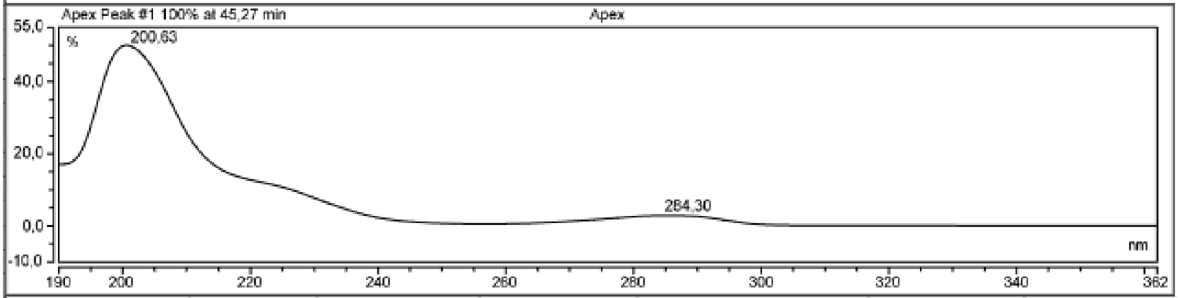

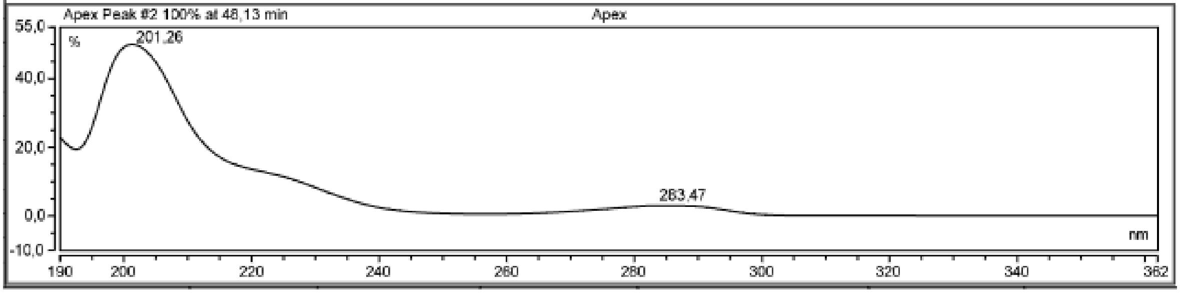

(*S,E*)-3-Bromo-6-methyl-2-(pent-3-en-2-yl)phenol (3m)

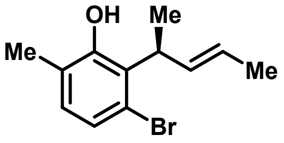

$[\alpha]^{20}$= - 59.67 (c 1.35, CH_2Cl_2).

^{1}H NMR (400 MHz, $CDCl_3$) δ 7.04 (d, *J* = 8.1 Hz, 1H), 6.85 (dp, *J* = 8.1, 0.7 Hz, 1H), 6.19 (d, *J* = 0.5 Hz, 1H), 5.95 – 5.84 (m, 2H), 4.32 – 4.13 (m, 1H), 2.14 (d, *J* = 0.8 Hz, 3H), 1.85 – 1.77 (m, 3H), 1.37 (d, *J* = 7.1 Hz, 3H).

^{13}C NMR (101 MHz, $CDCl_3$) δ 154.2, 134.0, 130.0, 128.5, 127.7, 125.7, 124.5, 121.8, 39.3, 18.3, 16.1, 15.9.

HRMS (ESI): exact mass calculated for $C_{12}H_{14}BrO^-$ [$(M - H)^-$], 253.0234 (100.0%), 255.0213 (97.3%); found 253.0238 (100.0%), 255.0217 (96.1%).

81% *ee* (determined by chiral HPLC: Chiralcel® OD column, n-Heptane/EtOH = 99.9:0.1, 0.3 mL/min, λ = 287.3 nm, 25 °C), major enantiomer. t_r = 15.96 min, minor enantiomer. t_r = 16.92 min

^{1}H NMR (400 MHz, $CDCl_3$)

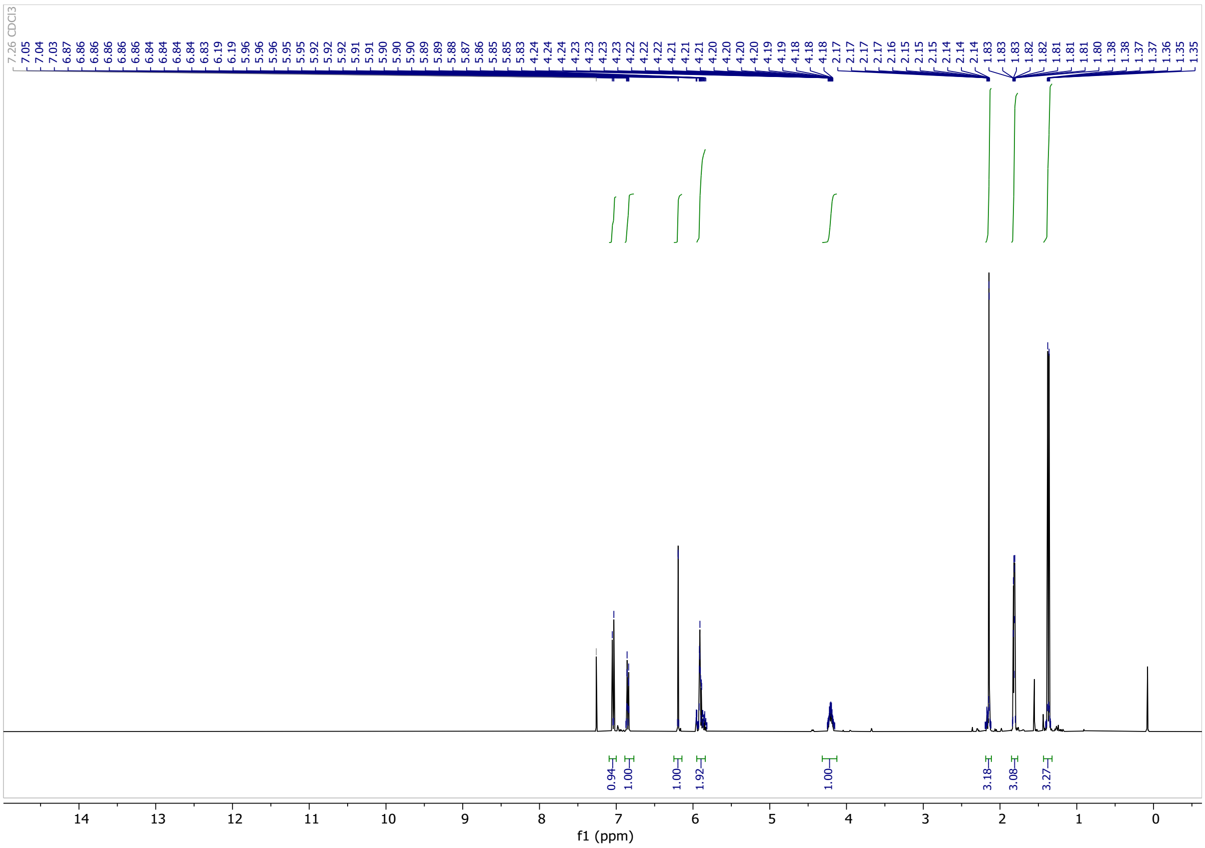

^{13}C NMR (101 MHz, $CDCl_3$)

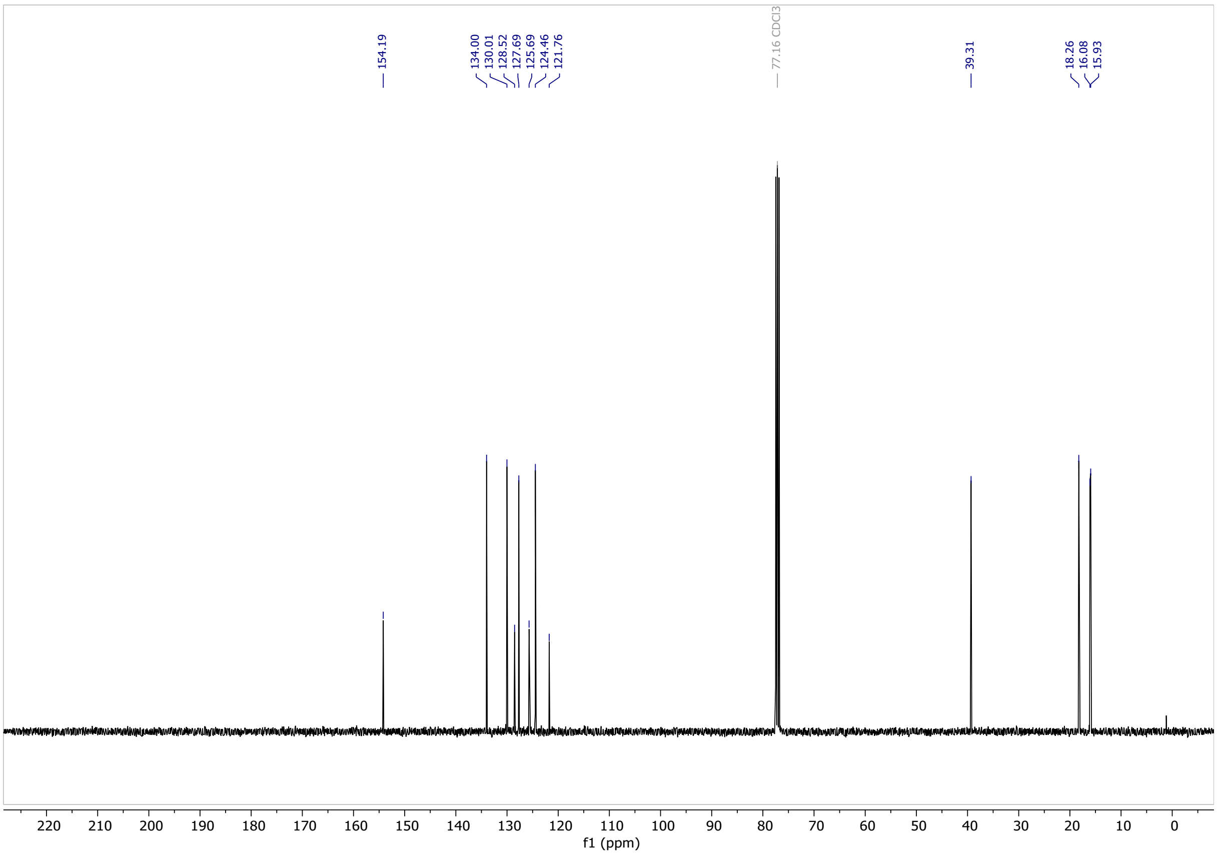
154.19
134.00
130.01
128.52
127.69
125.69
124.46
121.76
77.16 CDCl3
39.31
18.26
16.08
15.93
220 210 200 190 180 170 160 150 140 130 120 110 100 90 80 70 60 50 40 30 20 10 0
f1 (ppm)

Chromatogram and Results

Instrument Method:	**Heptane_EtOH_99.9_0.1_0.3mlmin_25C_30min**	*B %:*	**0,0**
Column:	**OD**	*C %:*	**0,0**
Run Time (min):	**30,00**	*D %:*	**0,1**
Channel:	**UV_VIS_1**		
Wavelength:	**287,26**		

Chromatogram

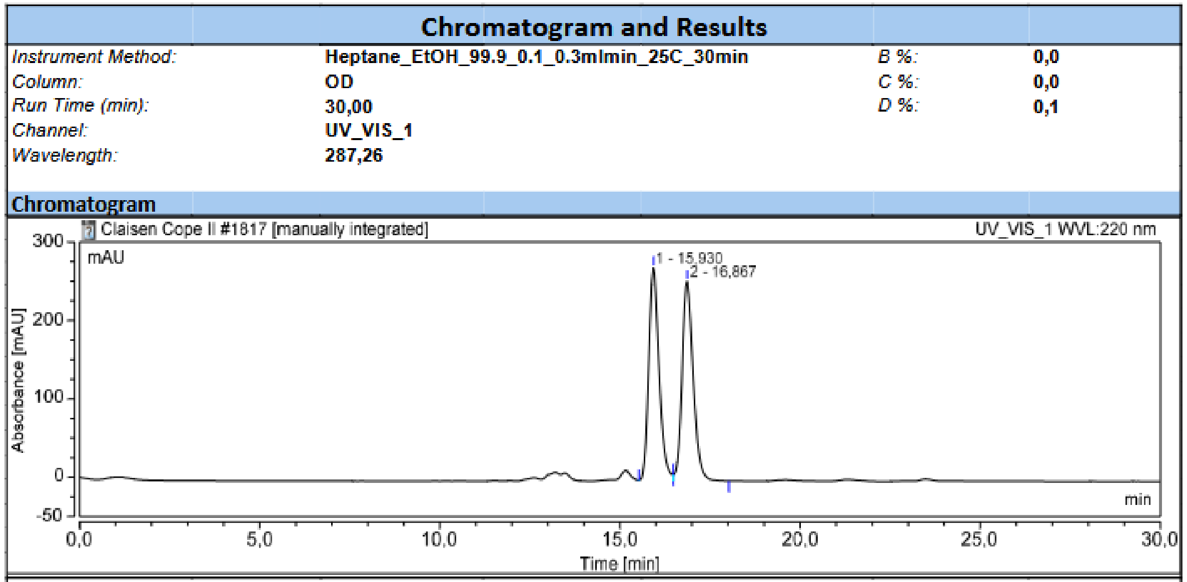

Integration Results

No.	Peak Name	Retention Time min	Area mAU*min	Height mAU	Relative Area %	Relative Height %
1		15,930	91,313	272,583	50,02	51,71
2		16,867	91,234	254,585	49,98	48,29
**Total:**			**182,547**	**527,169**	**100,00**	**100,00**

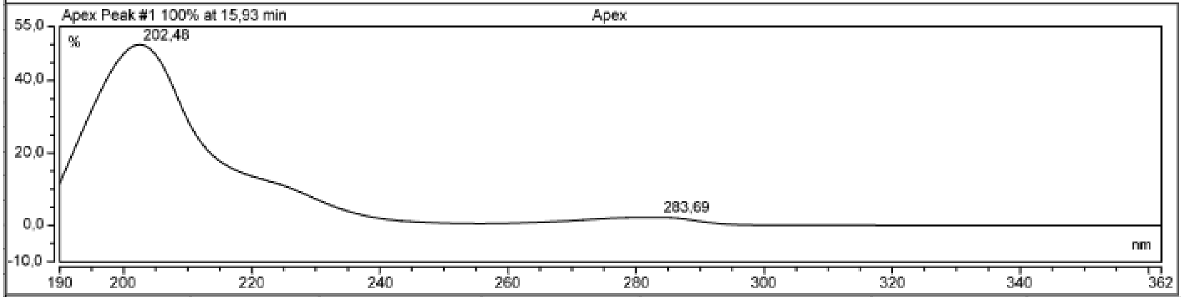

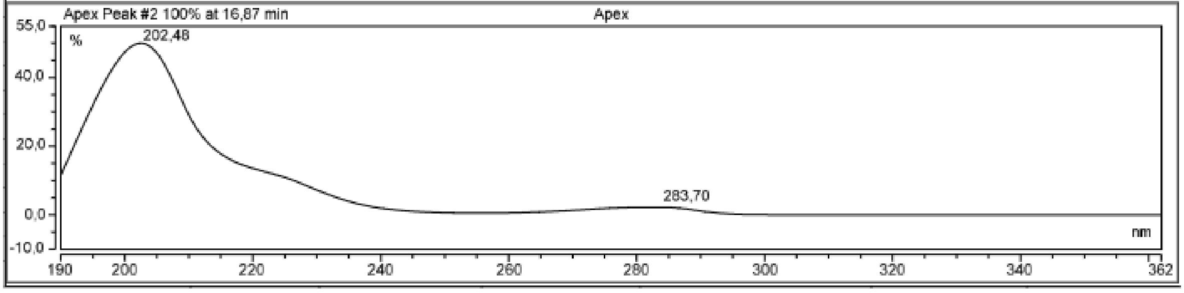

Chromatogram and Results

Instrument Method:	**Heptane_EtOH_99.9_0.1_0.3mlmin_25C_30min**	*B %:*	**0,0**
Column:	**OD**	*C %:*	**0,0**
Run Time (min):	**30,00**	*D %:*	**0,1**
Channel:	**UV_VIS_1**		
Wavelength:	**287,26**		

Chromatogram

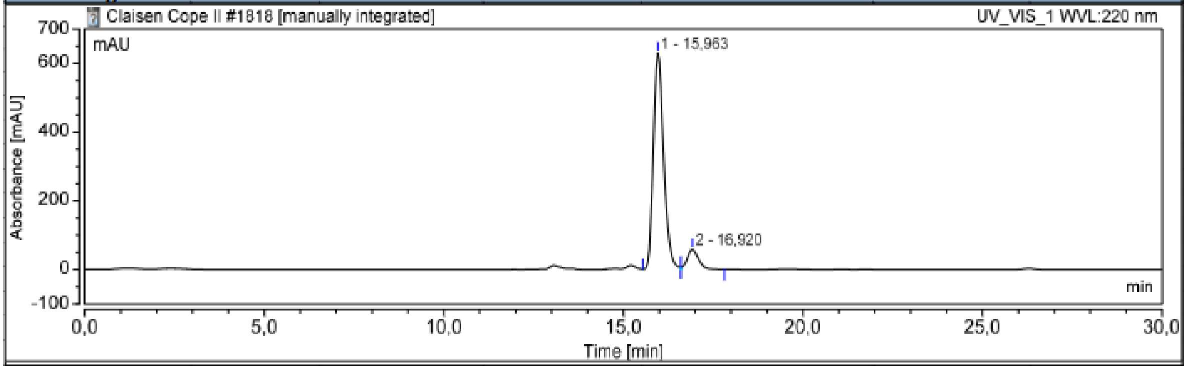

Integration Results

No.	Peak Name	Retention Time min	Area mAU*min	Height mAU	Relative Area %	Relative Height %
1		15,963	209,859	630,559	90,70	91,45
2		16,920	21,509	58,954	9,30	8,55
**Total:**			**231,368**	**689,513**	**100,00**	**100,00**

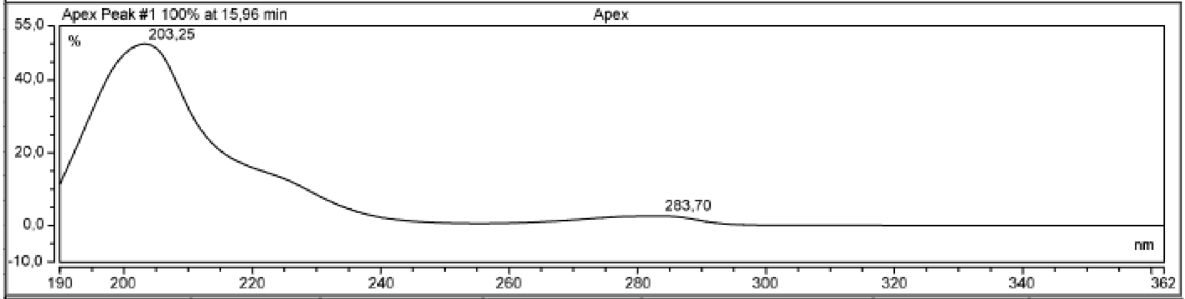

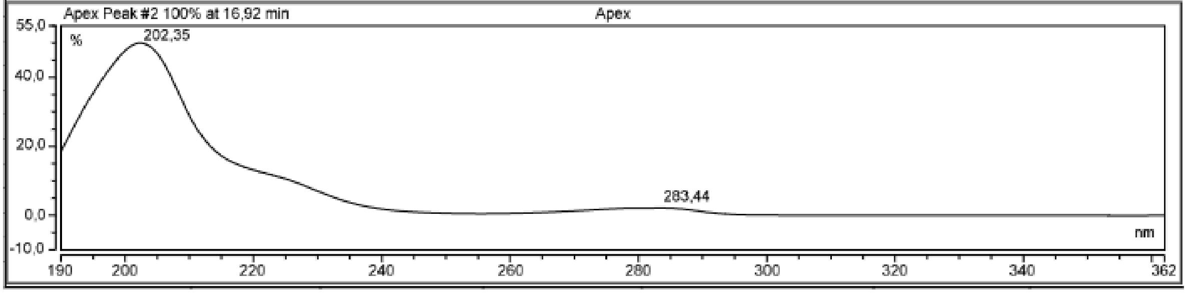

(*R,E*)-2-Methoxy-5-methyl-4-(pent-3-en-2-yl)phenol (2n) & (*S,E*)-6-methoxy-3-methyl-2-(pent-3-en-2-yl)phenol (3n)

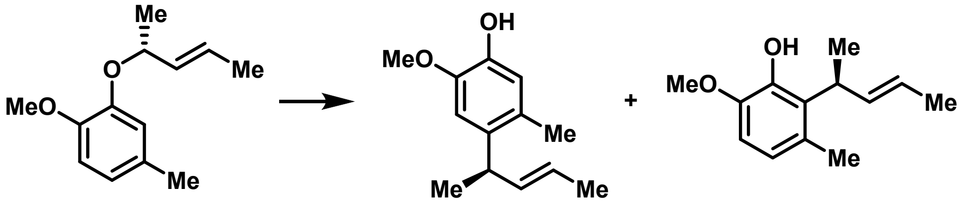

The title compounds were synthesized from **1n** (100 mg, 0.49 mmol) following **general procedure B**. The reaction was directly purified by column chromatography (petroleum ether/ethyl acetate 30:1) to provide the *para*-product **2n** as colorless oil in 31% yield (31 mg, 0.15 mmol) and the *ortho*-product **3n** as colorless oil in 68% yield (68 mg, 0.33 mmol).

(*R,E*)-2-Methoxy-5-methyl-4-(pent-3-en-2-yl)phenol (2n)

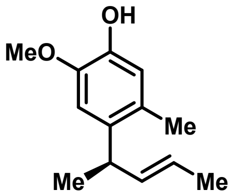

$[\alpha]^{20}$= + 19.76 (c 2.85, CH_2Cl_2).

^{1}H NMR (400 MHz, $CDCl_3$) δ 6.69 (d, *J* = 11.8 Hz, 2H), 5.56 (dd, *J* = 15.4, 6.1 Hz, 1H), 5.41 (d, *J* = 17.6 Hz, 2H), 3.86 (s, 3H), 3.56 (t, *J* = 6.8 Hz, 1H), 2.22 (s, 3H), 1.70 – 1.62 (m, 3H), 1.28 (d, *J* = 7.1 Hz, 3H).

^{13}C NMR (101 MHz, $CDCl_3$) δ 151.8, 144.8, 143.4, 135.9, 128.3, 123.4, 116.3, 109.1, 56.2, 37.5, 20.9, 18.6, 18.0.

HRMS (ESI): exact mass calculated for $C_{13}H_{17}O_2^-$ [(M - H)$^-$], 205.1234; found 205.1231.

87% *ee* (determined by chiral HPLC: Chiralcel® OJ-3 column, n-Heptane/EtOH = 99:1, 0.7 mL/min, λ = 287.3 nm, 25 °C), major enantiomer. t_r = 15.33 min, minor enantiomer. t_r = 16.72 min.

^{1}H NMR (400 MHz, $CDCl_3$)

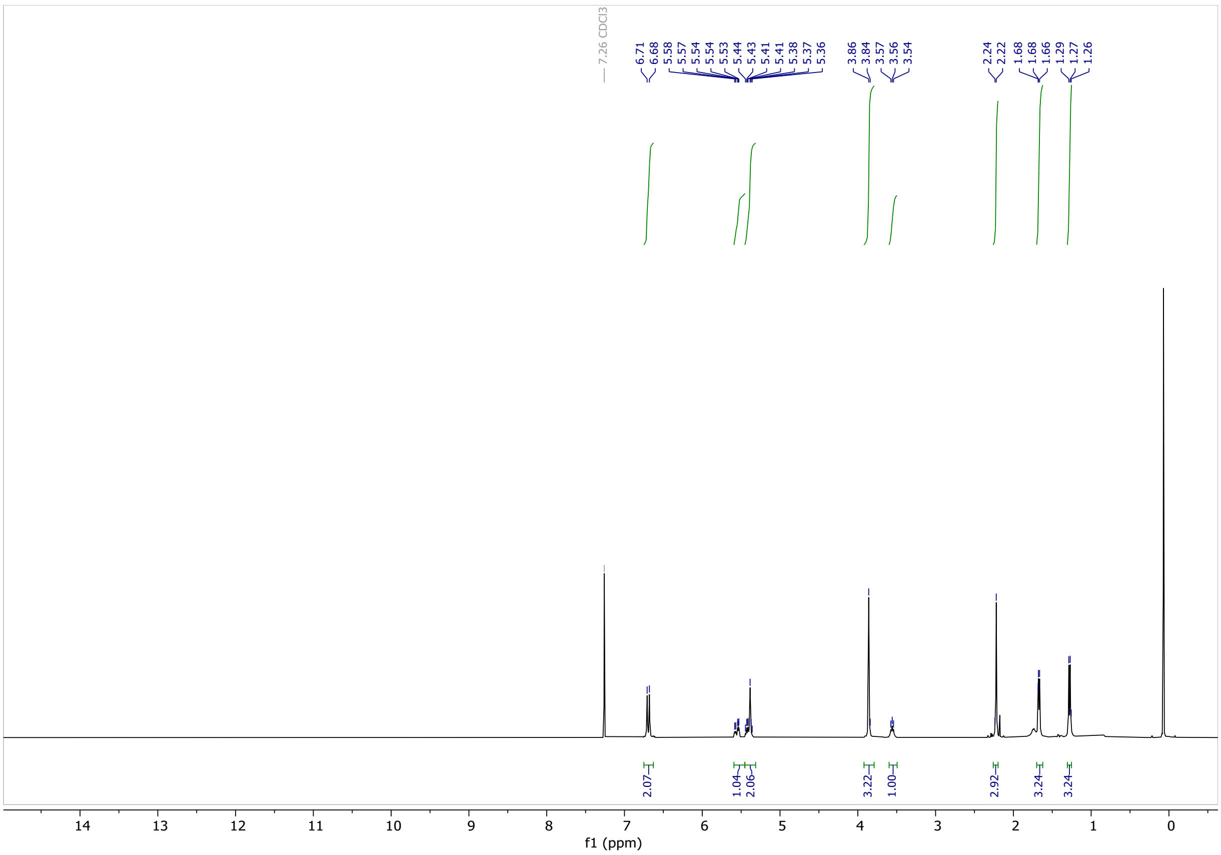

^{13}C NMR (101 MHz, $CDCl_3$)

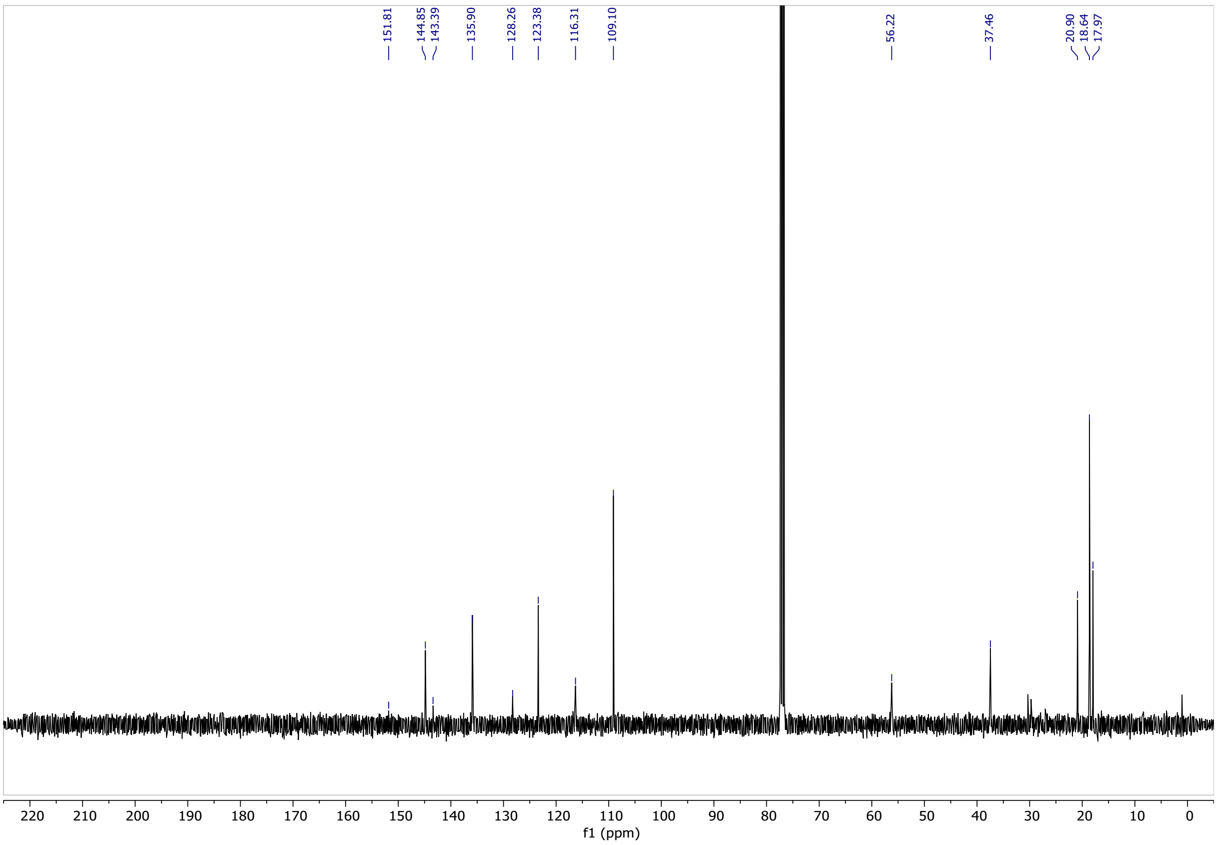
151.81
144.85
143.39
135.90
128.26
123.38
116.31
109.10
56.22
37.46
20.90
18.64
17.97
220 210 200 190 180 170 160 150 140 130 120 110 100 90 80 70 60 50 40 30 20 10 0
f1 (ppm)

Chromatogram and Results

Instrument Method:	**Heptane_EtOH_99_1_0.7mlmin_25C_40min-MK**	*B %:*	**0,0**
Column:	**OJ3**	*C %:*	**0,0**
Run Time (min):	**40,00**	*D %:*	**1,0**
Channel:	**UV_VIS_1**		
Wavelength:	**287,26**		

Chromatogram

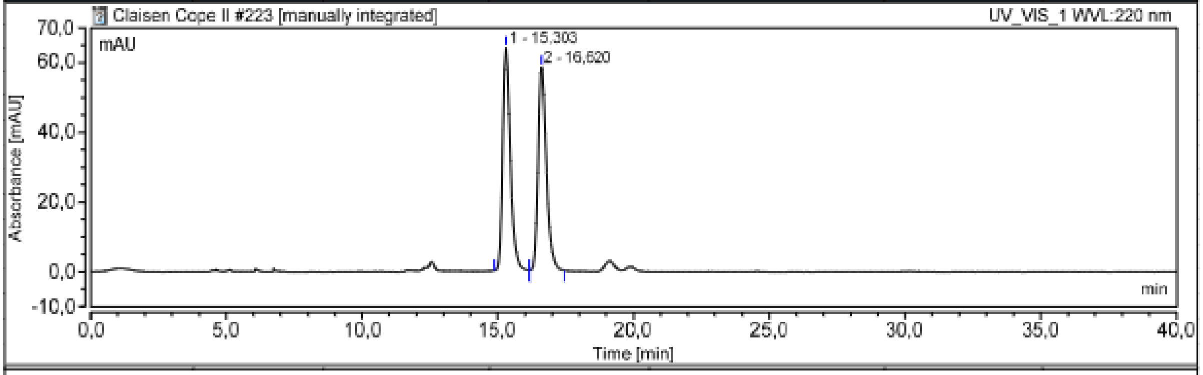

Integration Results

No.	Peak Name	Retention Time<br>min	Area<br>mAU*min	Height<br>mAU	Relative Area<br>%	Relative Height<br>%
1		15,303	20,423	63,958	50,31	52,23
2		16,620	20,172	58,499	49,69	47,77
**Total:**			**40,595**	**122,457**	**100,00**	**100,00**

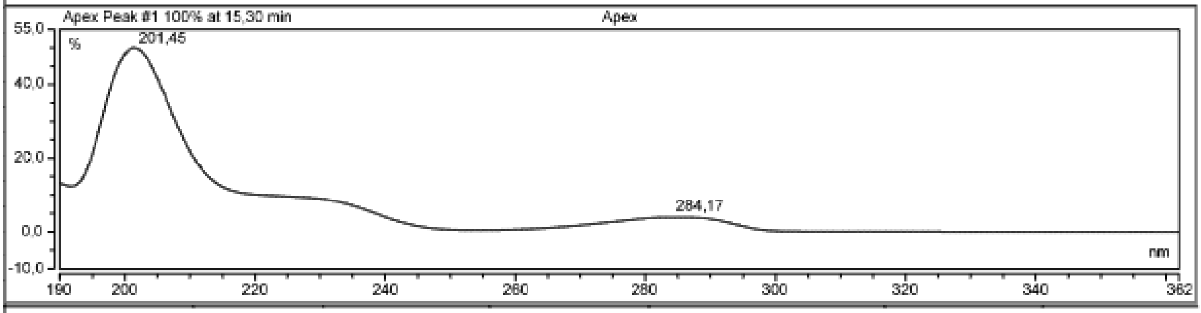

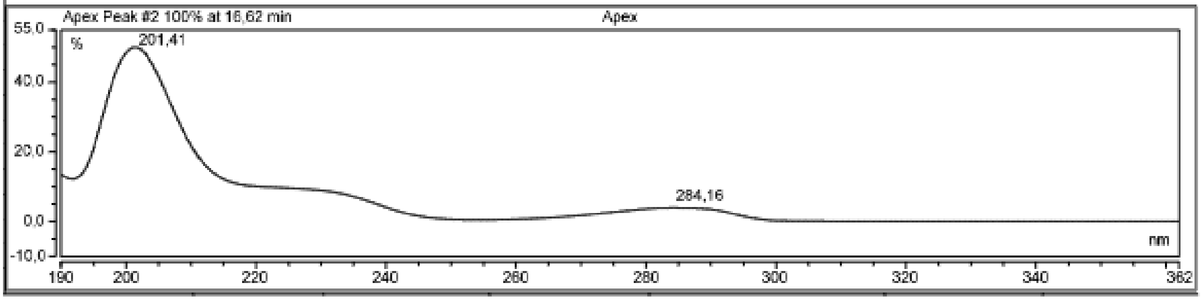

Chromatogram and Results

Instrument Method:	**Heptane_EtOH_99_1_0.7mlmin_25C_40min-MK**	*B %:*	**0,0**
Column:	**OJ3**	*C %:*	**0,0**
Run Time (min):	**40,00**	*D %:*	**1,0**
Channel:	**UV_VIS_1**		
Wavelength:	**287,26**		

Chromatogram

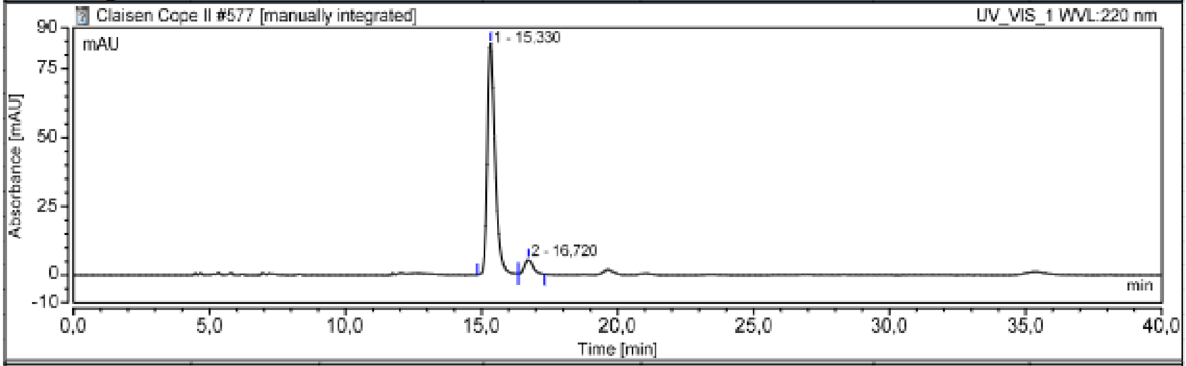

Integration Results

No.	Peak Name	Retention Time<br>min	Area<br>mAU*min	Height<br>mAU	Relative Area<br>%	Relative Height<br>%
1		15,330	27,477	84,107	93,42	94,01
2		16,720	1,936	5,359	6,58	5,99
**Total:**			**29,413**	**89,467**	**100,00**	**100,00**

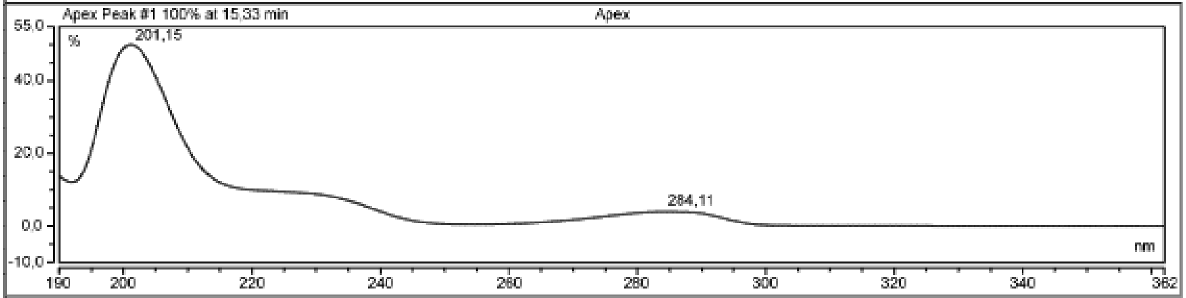

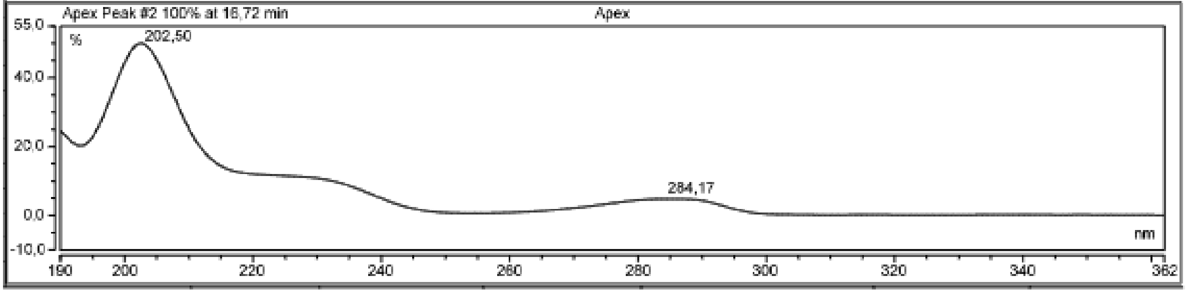

(*S,E*)-6-Methoxy-3-methyl-2-(pent-3-en-2-yl)phenol (3n)

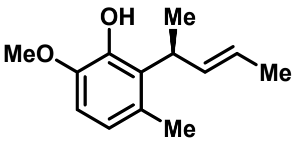

$[\alpha]^{20}$= -8.09 (c 2.00, CH_2Cl_2).

Compound **3n** was obtained as 1.5:1.0 *E/Z* mixture as measured by the ratio of the major (*E*)-isomer δ 6.62 (s, 2H, integral= 2.98), to the minor (*Z*)-isomer δ 6.61 (s, 2H, integral= 2.00); ^{1}H NMR (400 MHz, $CDCl_3$) δ 6.62 (s, 2.98H), 6.61 (s, 2.00H), 6.04 (ddq, *J* = 10.5, 8.6, 1.8 Hz, 1.08H), 5.95 (ddq, *J* = 15.3, 6.7, 1.7 Hz, 1.66H), 5.78 (s, 1.56H), 5.76 (s, 1.03H), 5.49 (dqd, *J* = 15.2, 6.4, 1.5 Hz, 1.76H), 5.44 – 5.36 (m, 1.08H), 4.10 (p, *J* = 7.5 Hz, 1.15H), 3.84 (d, *J* = 1.4 Hz, 10.54H), 2.33 (s, 3.23H), 2.27 (s, 5.24H), 1.67 (dt, *J* = 6.4, 1.4 Hz, 4.98H), 1.61 (ddd, *J* = 6.9, 1.8, 0.5 Hz, 3.30H), 1.41 (d, *J* = 7.2 Hz, 5.10H), 1.38 (d, *J* = 7.1 Hz, 3.40H).

^{13}C NMR (101 MHz, $CDCl_3$) δ 145.4, 145.2, 144.0, 143.9, 135.0, 134.8, 130.5, 129.9, 128.9, 128.6, 123.8, 122.7, 121.2, 121.1, 108.2, 108.0, 56.1, 36.6, 31.8, 20.4, 20.3, 19.5, 18.6, 18.0, 13.1.

HRMS (ESI): exact mass calculated for $C_{13}H_{17}O_2^-$ $[(M - H)^-]$, 205.1234; found 205.1232.

(*E*)-isomer: 87% *ee* (determined by chiral HPLC: Chiralcel® OJ-3 column, n-Heptane/EtOH = 99:1, 0.7 mL/min, λ = 287.3 nm, 25 °C), major enantiomer. t_r = 12.30 min, minor enantiomer. t_r = 19.07 min.

(*Z*)-isomer: 87% *ee* (determined by chiral HPLC: Chiralcel® OJ-3 column, n-Heptane/EtOH = 99:1, 0.7 mL/min, λ = 287.3 nm, 25 °C), major enantiomer. t_r = 30.41 min, minor enantiomer. t_r = 15.14 min.

^1H NMR (400 MHz, $CDCl_3$)

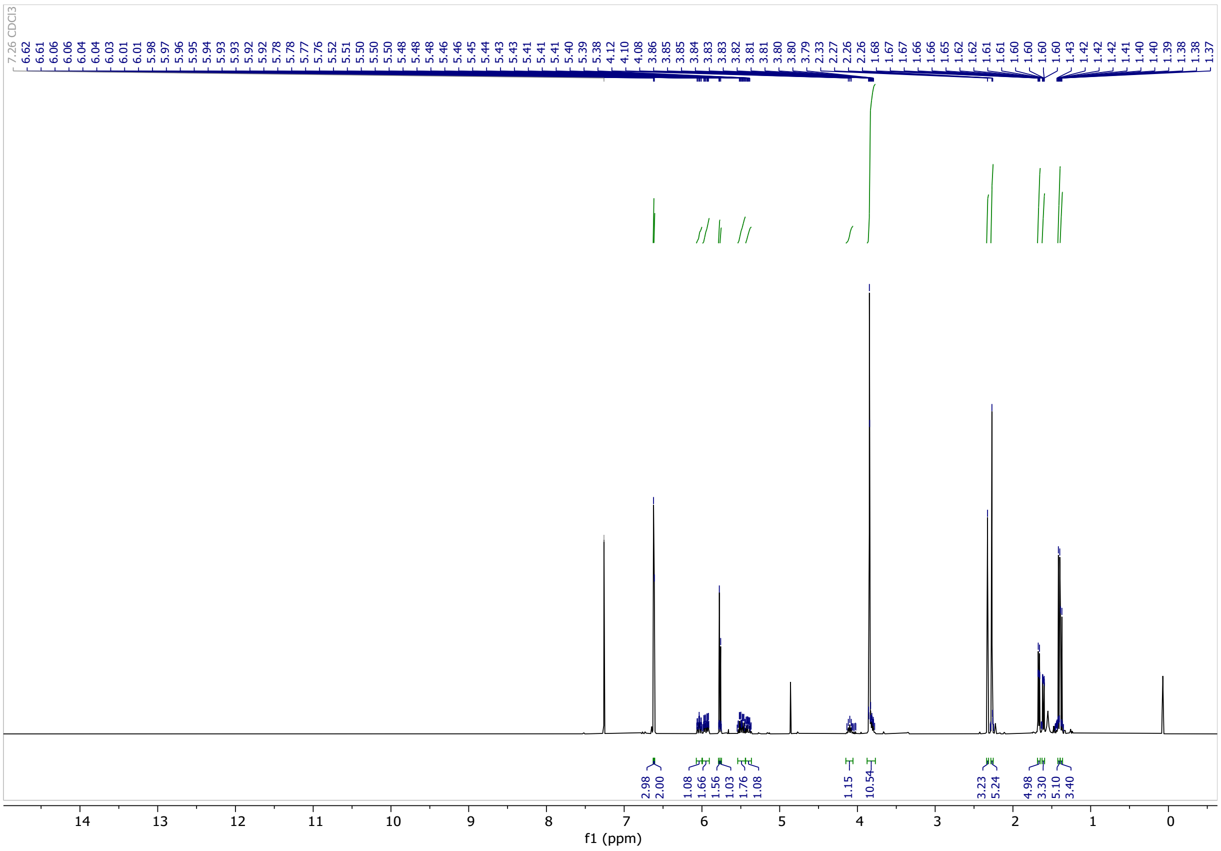

^{13}C NMR (101 MHz, $CDCl_3$)

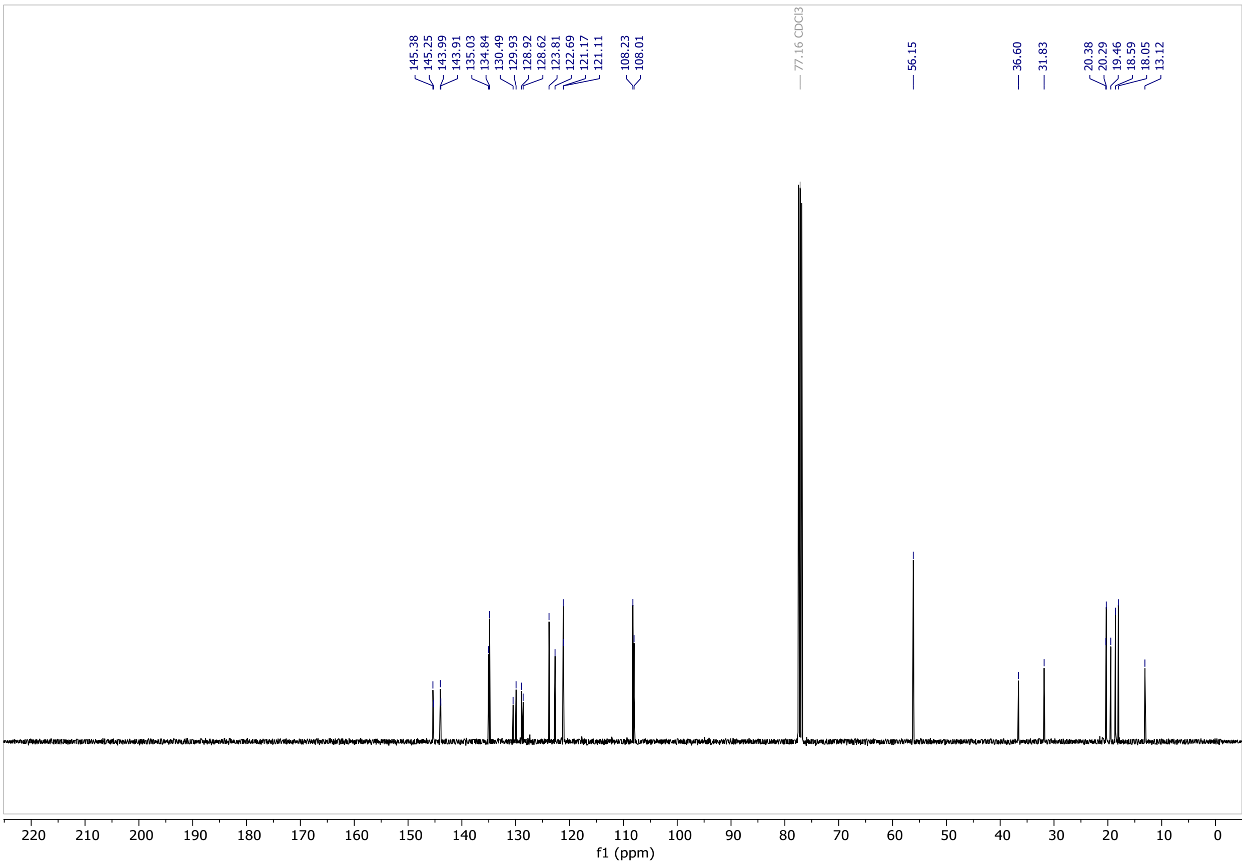
145.38
145.25
143.99
143.91
135.03
134.84
130.49
129.93
128.92
128.62
123.81
122.69
121.17
121.11
108.23
108.01
77.16 CDCl3
56.15
36.60
31.83
20.38
20.29
19.46
18.59
18.05
13.12
220 210 200 190 180 170 160 150 140 130 120 110 100 90 80 70 60 50 40 30 20 10 0
f1 (ppm)

Chromatogram and Results

Instrument Method:	**Heptane_EtOH_99_1_0.7mlmin_25C_40min-MK**	*B %:*	**0,0**
Column:	**OJ3**	*C %:*	**0,0**
Run Time (min):	**40,00**	*D %:*	**1,0**
Channel:	**UV_VIS_1**		
Wavelength:	**287,26**		

Chromatogram

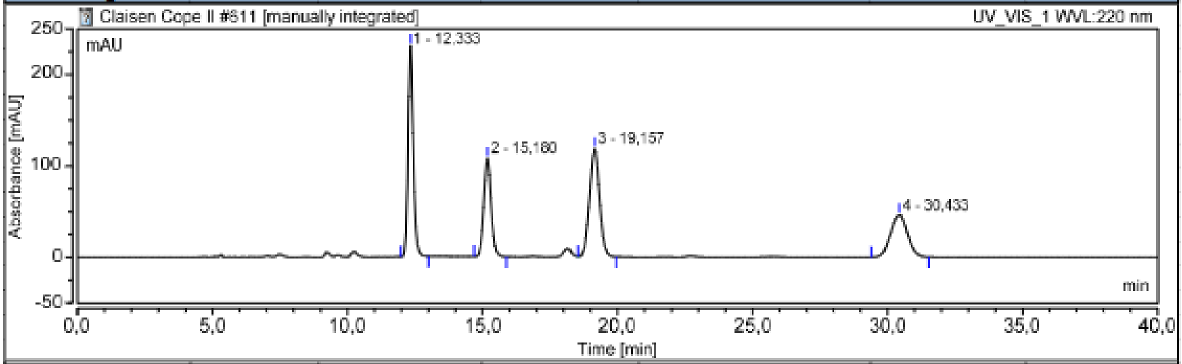

Integration Results

No.	Peak Name	Retention Time min	Area mAU*min	Height mAU	Relative Area %	Relative Height %
1		12,333	52,300	230,863	30,62	45,87
2		15,180	33,642	107,562	19,69	21,37
3		19,157	51,532	118,571	30,17	23,56
4		30,433	33,342	46,320	19,52	9,20
**Total:**			**170,817**	**503,316**	**100,00**	**100,00**

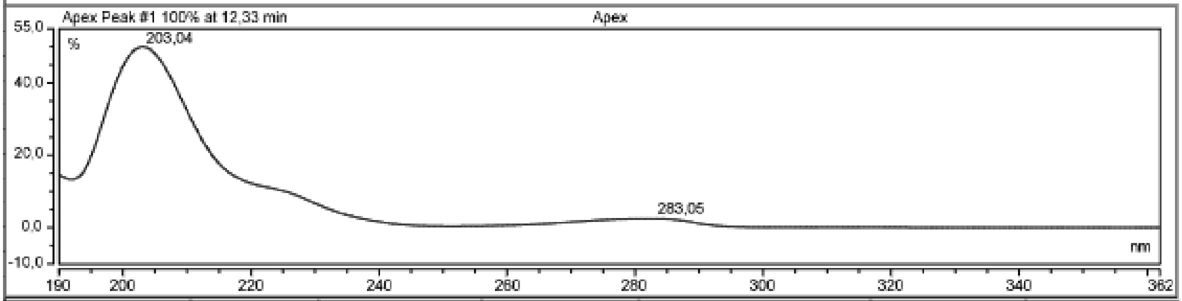

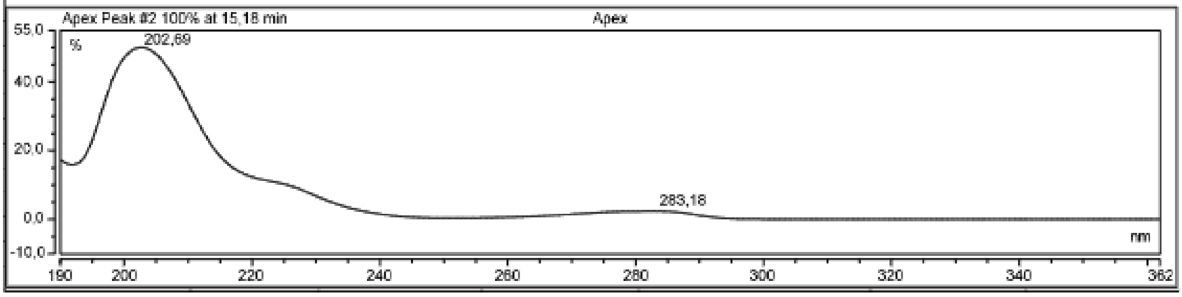

Chromatogram and Results

Instrument Method:	**Heptane_EtOH_99_1_0.7mlmin_25C_40min-MK**	*B %:*	**0,0**
Column:	**OJ3**	*C %:*	**0,0**
Run Time (min):	**40,00**	*D %:*	**1,0**
Channel:	**UV_VIS_1**		
Wavelength:	**287,26**		

Chromatogram

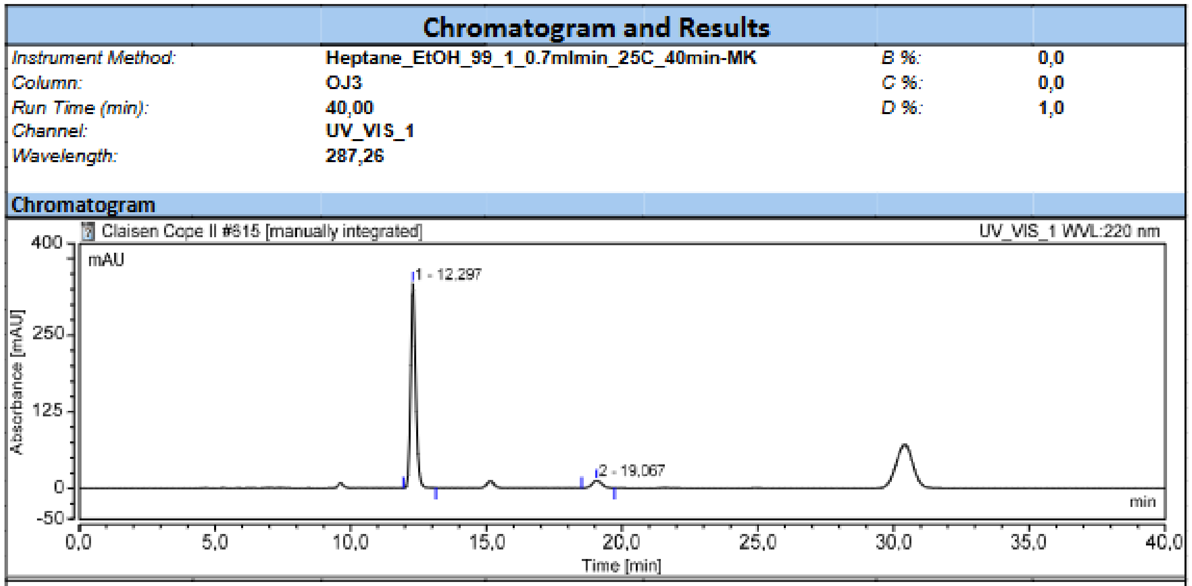

Integration Results

No.	Peak Name	Retention Time<br>min	Area<br>mAU*min	Height<br>mAU	Relative Area<br>%	Relative Height<br>%
1		12,297	74,803	334,602	93,37	96,38
2		19,067	5,314	12,585	6,63	3,62
**Total:**			**80,117**	**347,188**	**100,00**	**100,00**

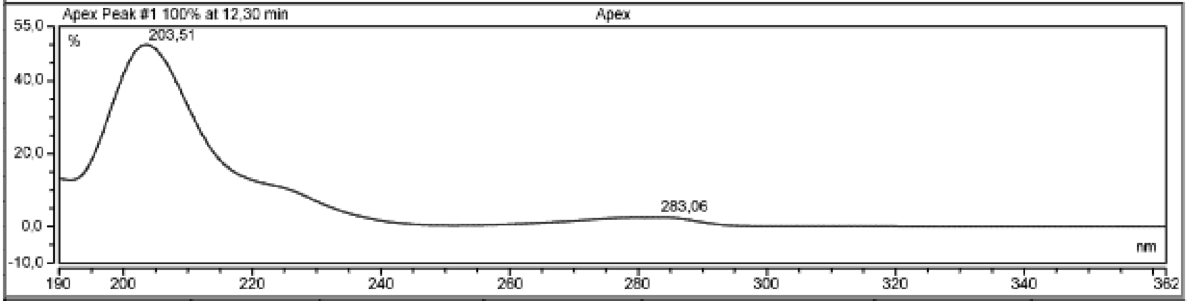

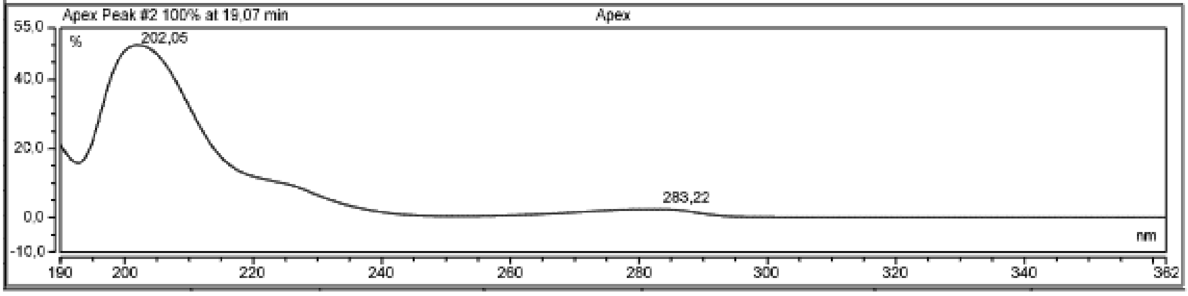

Chromatogram and Results

Instrument Method:	**Heptane_EtOH_99_1_0.7mlmin_25C_40min-MK**	*B %:*	**0,0**
Column:	**OJ3**	*C %:*	**0,0**
Run Time (min):	**40,00**	*D %:*	**1,0**
Channel:	**UV_VIS_1**		
Wavelength:	**287,26**		

Chromatogram

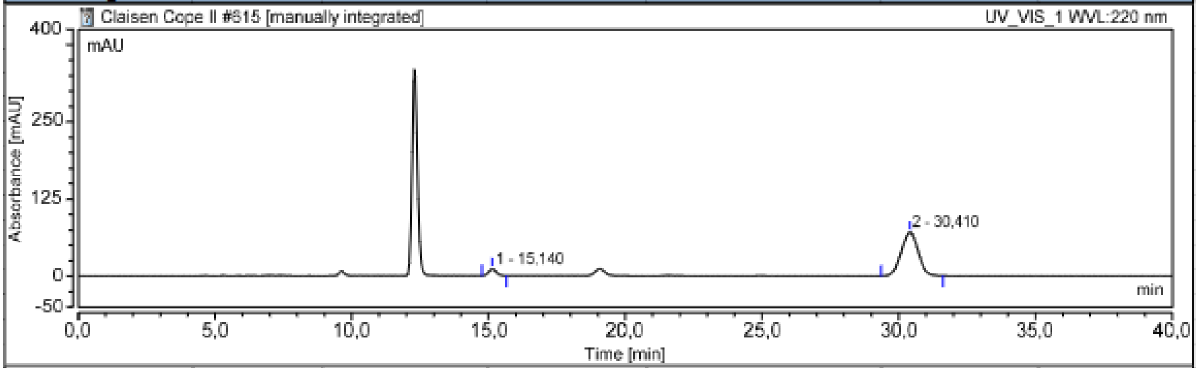

Integration Results

No.	Peak Name	Retention Time<br>min	Area<br>mAU*min	Height<br>mAU	Relative Area<br>%	Relative Height<br>%
1		15,140	3,662	11,879	6,63	14,24
2		30,410	51,568	71,567	93,37	85,76
**Total:**			**55,229**	**83,446**	**100,00**	**100,00**

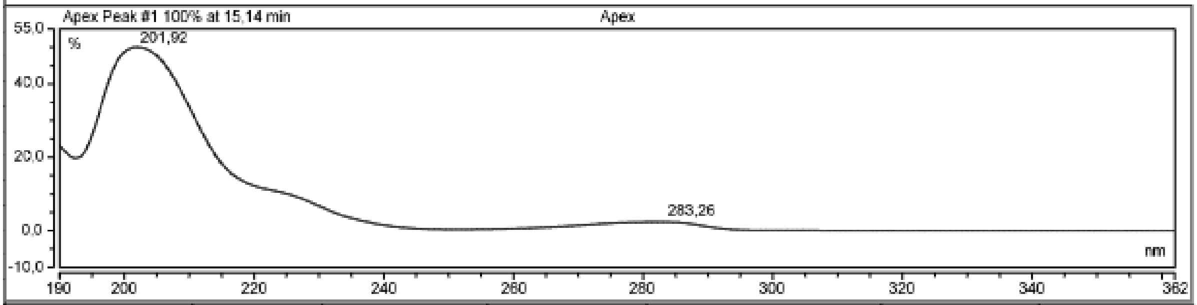

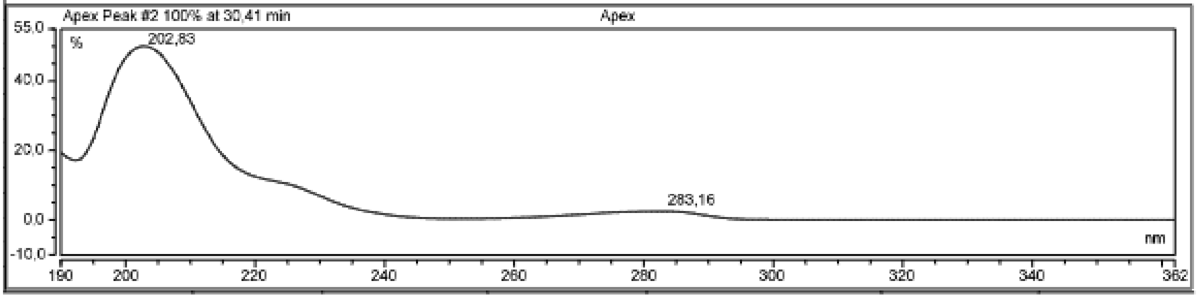

(*R,E*)-2-Fluoro-5-methyl-4-(pent-3-en-2-yl)phenol (2o) & (*S,E*)-6-fluoro-3-methyl-2-(pent-3-en-2-yl)phenol (3o)

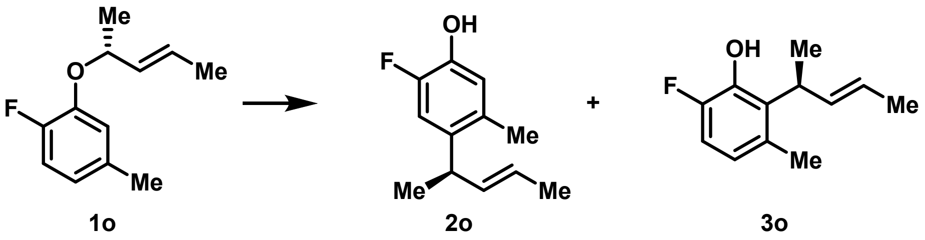

The title compounds were synthesized from **1o** (90 mg, 0.46 mmol) following **general procedure B**. The reaction was directly purified by column chromatography (petroleum ether/ethyl acetate 40:1 to 30:1) to provide the *para*-product **2o** as pale-yellow oil in 53% yield (48 mg, 0.25 mmol) and the *ortho*-product **3o** as pale-yellow oil in 46% yield (41 mg, 0.21 mmol).

(*R,E*)-2-Fluoro-5-methyl-4-(pent-3-en-2-yl)phenol (2o)

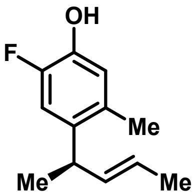

$[\alpha]^{20}$= + 0.60 (c 1.15, CH_2Cl_2).

^1H NMR (400 MHz, $CDCl_3$) δ 6.89 (d, *J* = 12.2 Hz, 1H), 6.79 (d, *J* = 9.1 Hz, 1H), 5.51 (ddd, *J* = 15.4, 6.1, 1.6 Hz, 1H), 5.46 – 5.33 (m, 1H), 5.06 (s, 1H), 3.52 (p, *J* = 6.8 Hz, 1H), 2.23 (s, 3H), 1.67 (dt, *J* = 6.2, 1.4 Hz, 3H), 1.26 (d, *J* = 7.0 Hz, 3H).

^{13}C NMR (101 MHz, $CDCl_3$) δ 149.6 (d, *J* = 234.2 Hz), 140.9 (d, *J* = 14.4 Hz), 137.3 (d, *J* = 4.7 Hz), 135.4, 132.0 (d, *J* = 3.5 Hz), 123.9, 118.8, 113.3 (d, *J* = 18.1 Hz), 37.3, 20.8, 18.8, 18.0.

^{19}F NMR (377 MHz, $CDCl_3$) δ -144.7.

88% *ee* (determined by chiral HPLC: Chiralpak® AS-H column, n-Hexane/iPrOH = 99.7:0.3, 0.5 mL/min, λ = 287.3 nm, 25 °C), major enantiomer. t_r = 35.18 min, minor enantiomer. t_r = 40.70 min.

HRMS (ESI): exact mass calculated for $C_{12}H_{14}FO^-$ [(M - H)$^-$], 193.1034; found 193.1025.

^1H NMR (400 MHz, $CDCl_3$)

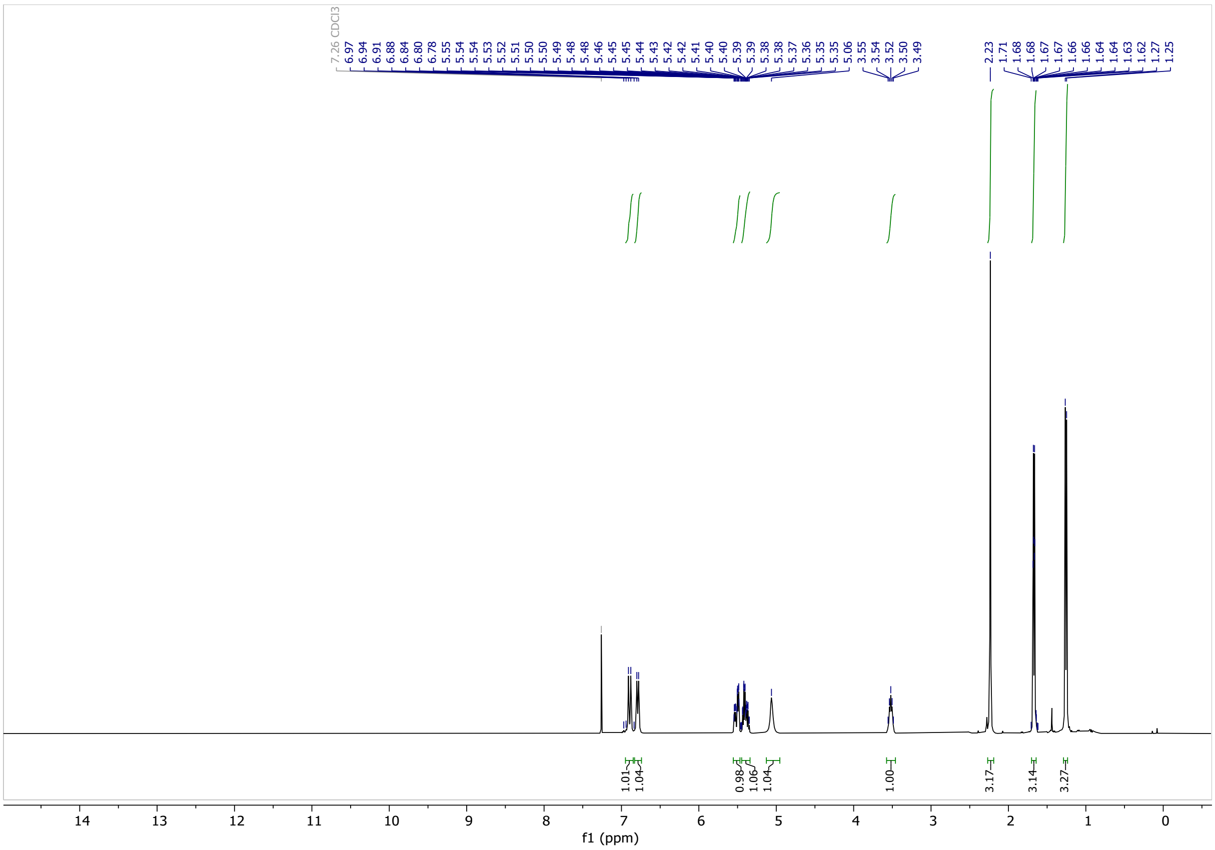

^{13}C NMR (101 MHz, $CDCl_3$)

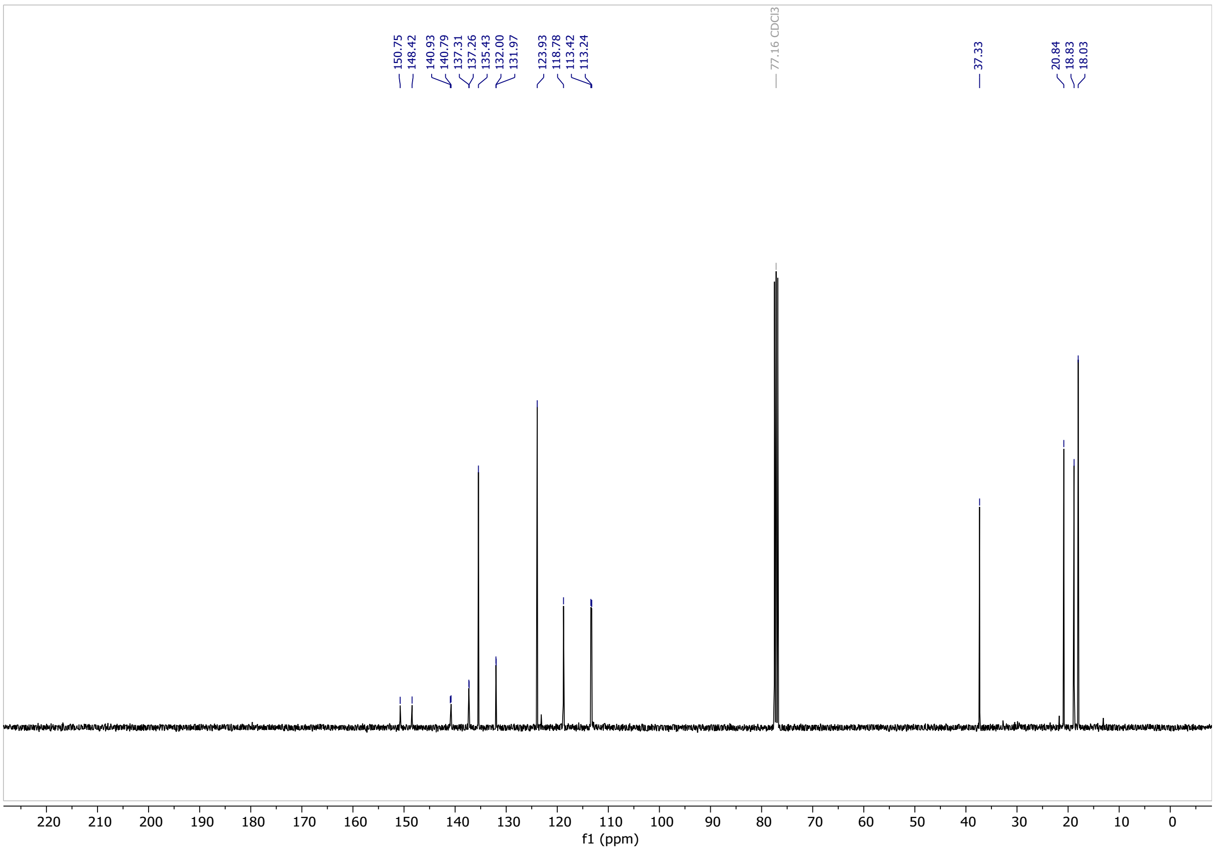

^{19}F NMR (377 MHz, $CDCl_3$)

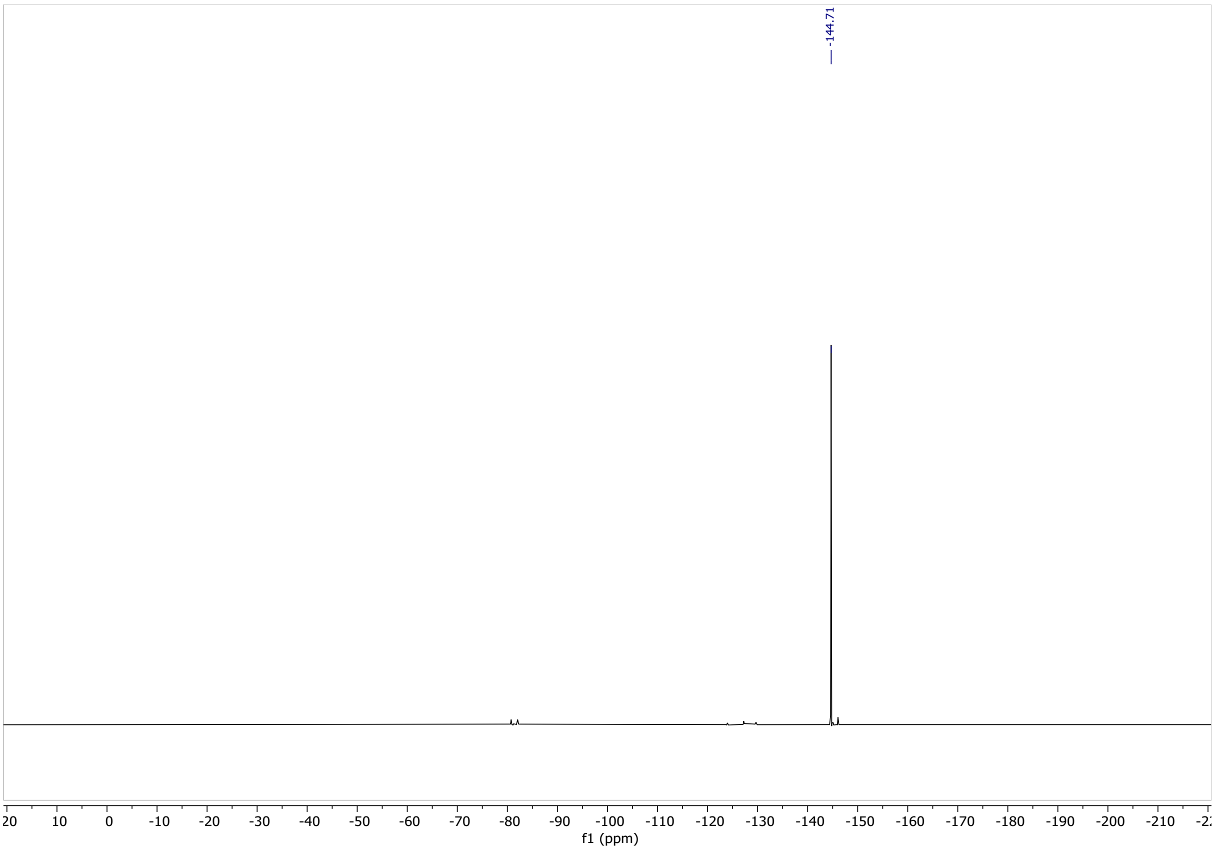

Chromatogram and Results

Instrument Method:	**Hexane_IPA_99.7_0.3_0.5mlmin_25C_60min**	*B %:*	**0,3**
Column:	**AS-H**	*C %:*	**99,7**
Run Time (min):	**60,00**	*D %:*	**0,0**
Channel:	**UV_VIS_1**		
Wavelength:	**287,26**		

Chromatogram

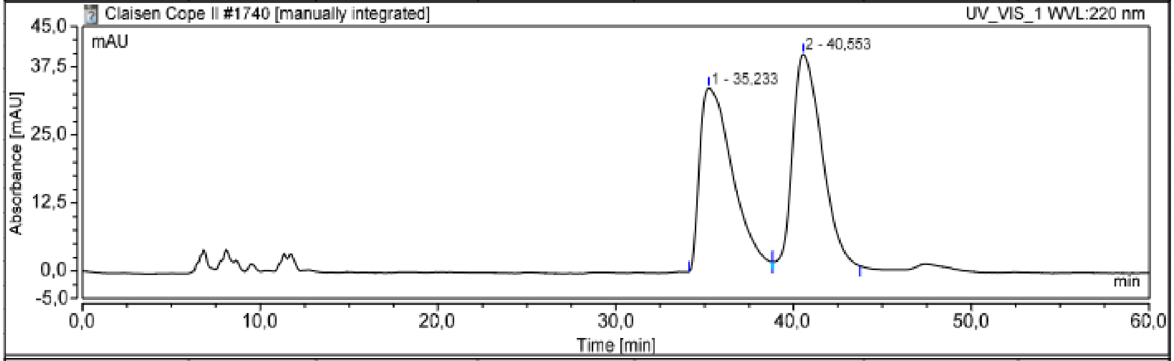

Integration Results

No.	Peak Name	Retention Time<br>min	Area<br>mAU*min	Height<br>mAU	Relative Area<br>%	Relative Height<br>%
1		35,233	71,920	33,653	48,99	46,15
2		40,553	74,888	39,265	51,01	53,85
**Total:**			**146,808**	**72,918**	**100,00**	**100,00**

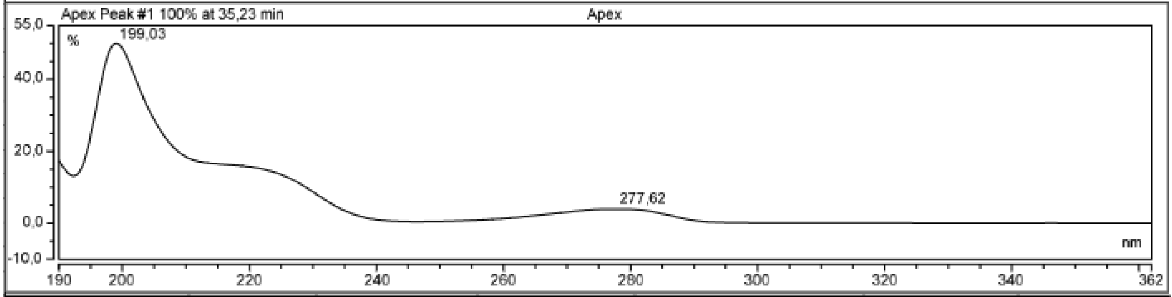

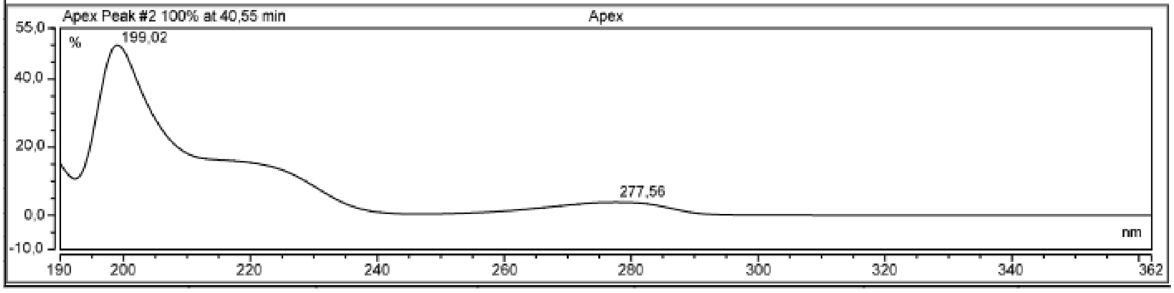

Chromatogram and Results

Instrument Method:	**Hexane_IPA_99.7_0.3_0.5mlmin_25C_60min**	*B %:*	**0,3**
Column:	**AS-H**	*C %:*	**99,7**
Run Time (min):	**60,00**	*D %:*	**0,0**
Channel:	**UV_VIS_1**		
Wavelength:	**287,26**		

Chromatogram

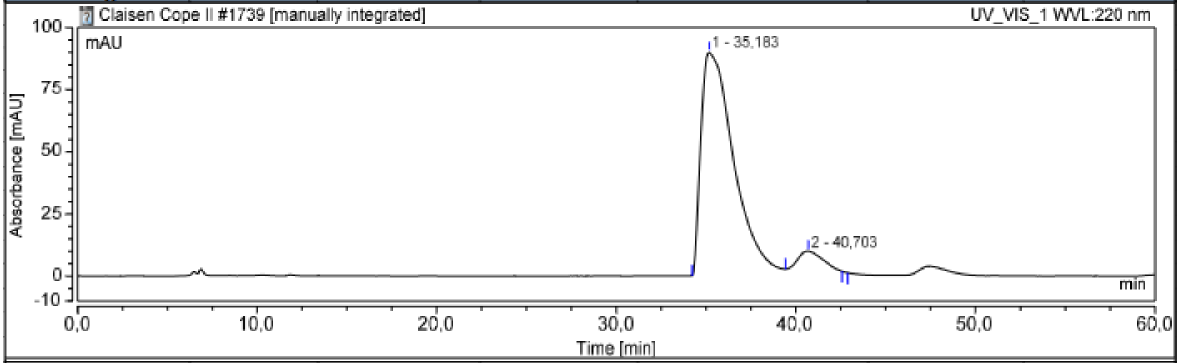

Integration Results

No.	Peak Name	Retention Time<br>min	Area<br>mAU*min	Height<br>mAU	Relative Area<br>%	Relative Height<br>%
1		35,183	193,630	89,763	94,16	92,17
2		40,703	12,008	7,620	5,84	7,83
**Total:**			**205,638**	**97,383**	**100,00**	**100,00**

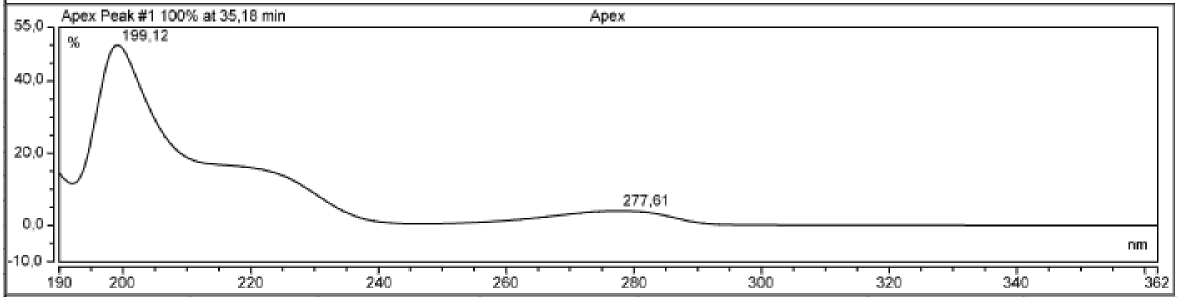

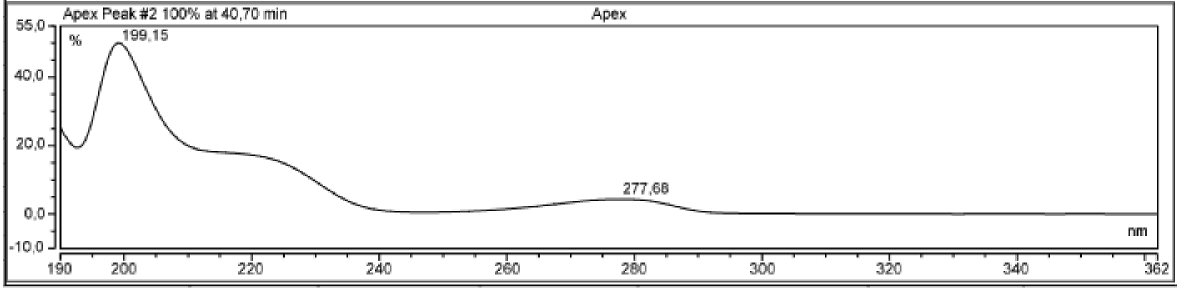

(*S,E*)-6-Fluoro-3-methyl-2-(pent-3-en-2-yl)phenol (3o)

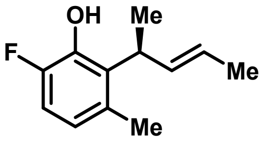

$[\alpha]^{20}$= - 21.88 (c 2.05, CH_2Cl_2).

^{1}H NMR (400 MHz, $CDCl_3$) δ 6.82 (dd, *J* = 10.2, 8.4 Hz, 1H), 6.62 (dd, *J* = 8.8, 5.0 Hz, 1H), 5.92 (ddq, *J* = 15.6, 5.1, 1.6 Hz, 1H), 5.71 – 5.54 (m, 1H), 5.47 (dd, *J* = 3.8, 0.8 Hz, 1H), 3.86 – 3.74 (m, 1H), 2.28 (s, 3H), 1.73 (dt, *J* = 6.4, 1.7 Hz, 3H), 1.39 (d, *J* = 7.2 Hz, 3H).

^{13}C NMR (101 MHz, $CDCl_3$) δ 150.5 (d, *J* = 236.5 Hz), 142.5 (d, *J* = 13.1 Hz), 134.3, 131.8 (d, *J* = 3.6 Hz), 131.6, 125.5, 121.8 (d, *J* = 6.9 Hz), 112.8 (d, *J* = 17.9 Hz), 36.3 (d, *J* = 2.5 Hz), 20.2, 18.1, 17.8.

^{19}F NMR (377 MHz, $CDCl_3$) δ -142.6.

HRMS (ESI): exact mass calculated for $C_{12}H_{14}FO^-$ [(M - H)$^-$], 193.1034; found 193.1031.

86% *ee* (determined by chiral HPLC: Chiralcel® OJ-3 column, n-Heptane/EtOH = 99:1, 0.7 mL/min, λ = 287.3 nm, 25 °C), major enantiomer. t_r = 13.36 min, minor enantiomer. t_r = 18.51 min.

^{1}H NMR (400 MHz, $CDCl_3$)

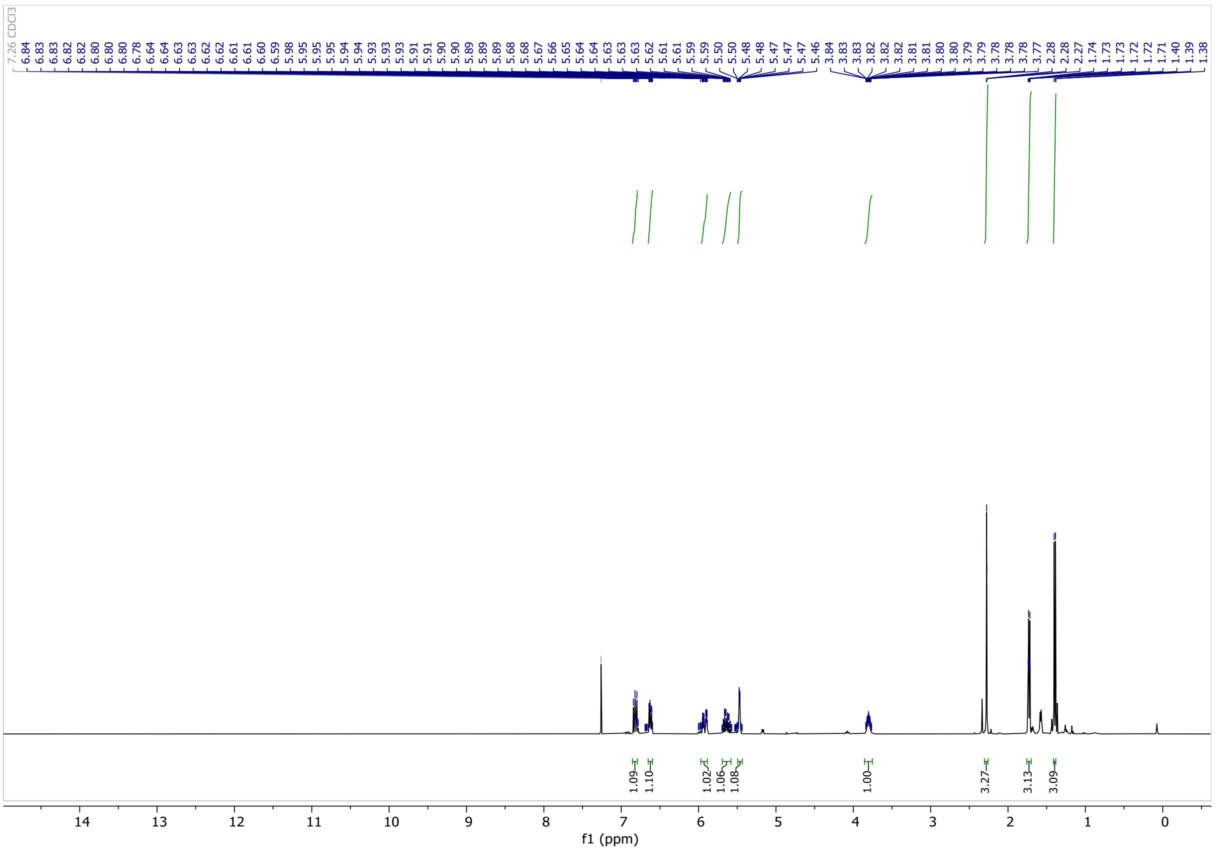

^{13}C NMR (101 MHz, $CDCl_3$)

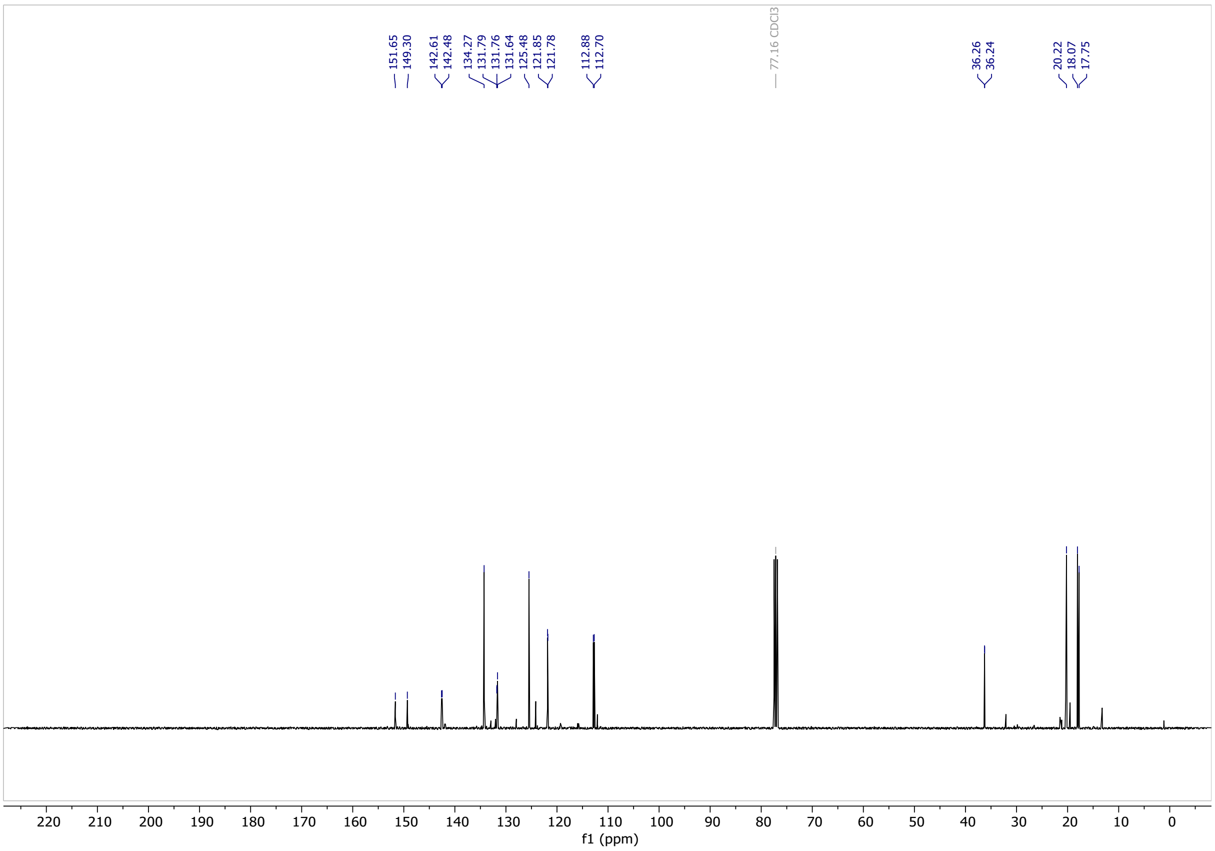

^{19}F NMR (377 MHz, $CDCl_3$)

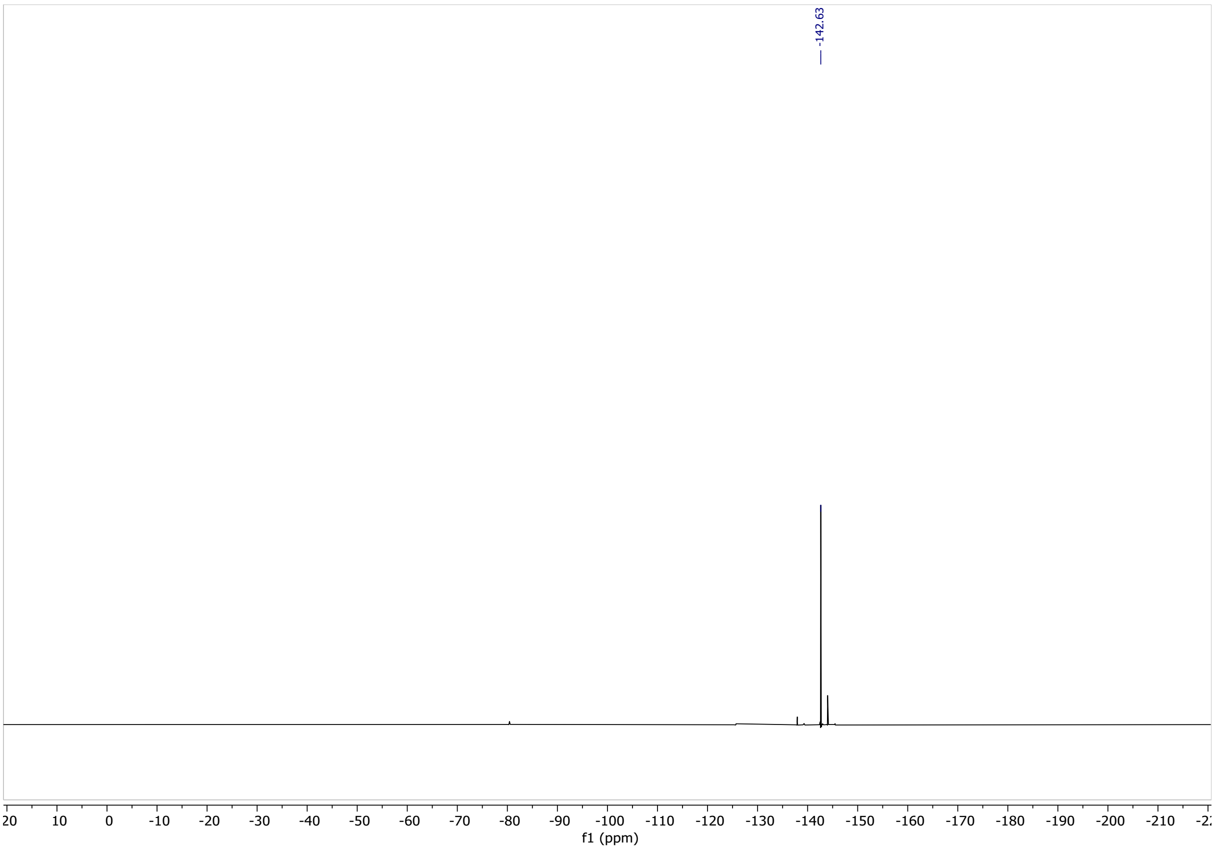

Chromatogram and Results

Instrument Method:	**Heptane_EtOH_99_1_0.7mlmin_25C_40min-MK**	*B %:*	**0,0**
Column:	**OJ3**	*C %:*	**0,0**
Run Time (min):	**40,00**	*D %:*	**1,0**
Channel:	**UV_VIS_1**		
Wavelength:	**287,26**		

Chromatogram

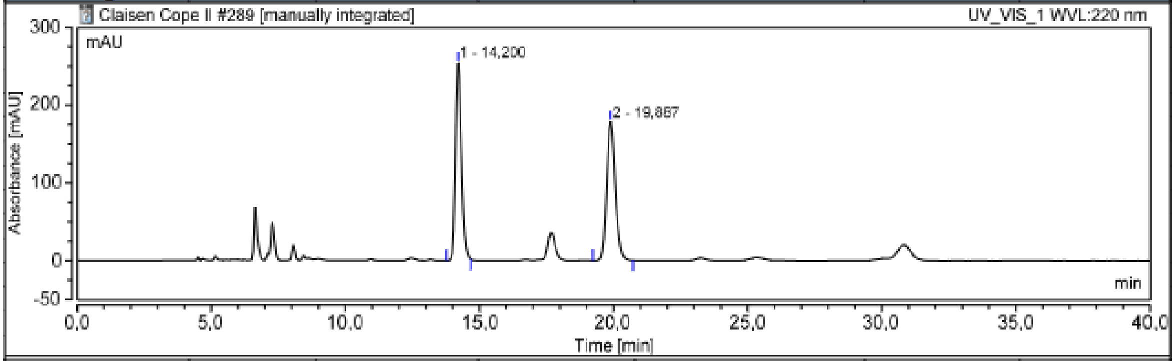

Integration Results

No.	Peak Name	Retention Time<br>min	Area<br>mAU*min	Height<br>mAU	Relative Area<br>%	Relative Height<br>%
1		14,200	66,085	253,624	49,76	58,66
2		19,887	66,730	178,752	50,24	41,34
**Total:**			**132,815**	**432,376**	**100,00**	**100,00**

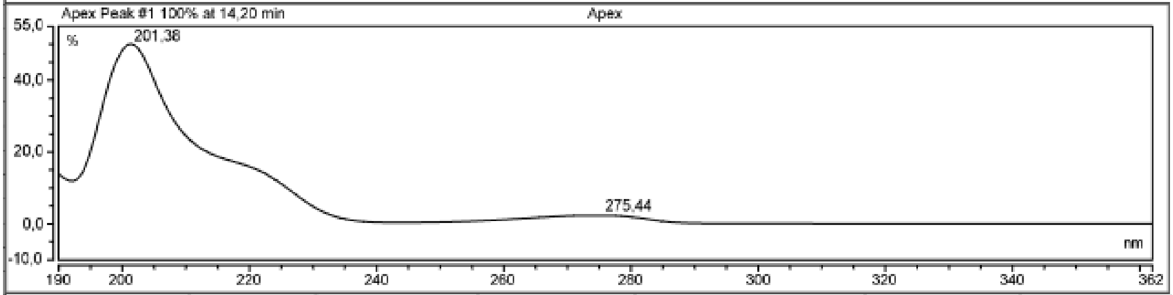

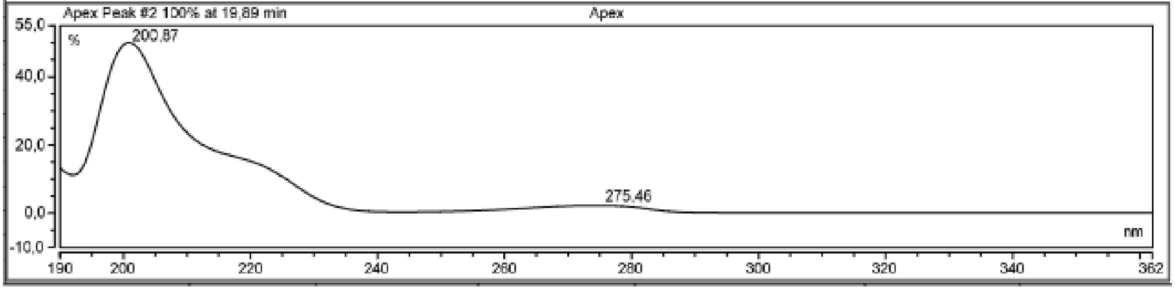

Chromatogram and Results

Instrument Method:	**Heptane_EtOH_99_1_0.7mlmin_25C_40min-MK**	*B %:*	**0,0**
Column:	**OJ3**	*C %:*	**0,0**
Run Time (min):	**40,00**	*D %:*	**1,0**
Channel:	**UV_VIS_1**		
Wavelength:	**287,26**		

Chromatogram

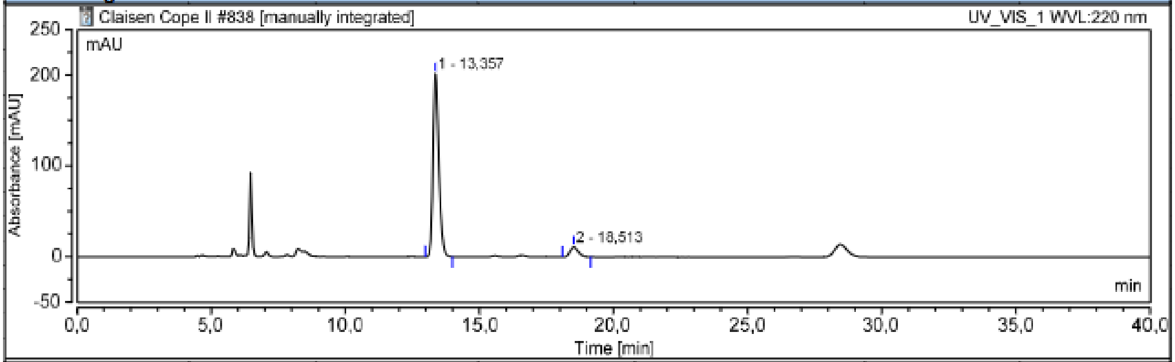

Integration Results

No.	Peak Name	Retention Time min	Area mAU*min	Height mAU	Relative Area %	Relative Height %
1		13,357	50,674	201,506	92,97	94,86
2		18,513	3,833	10,912	7,03	5,14
**Total:**			**54,507**	**212,418**	**100,00**	**100,00**

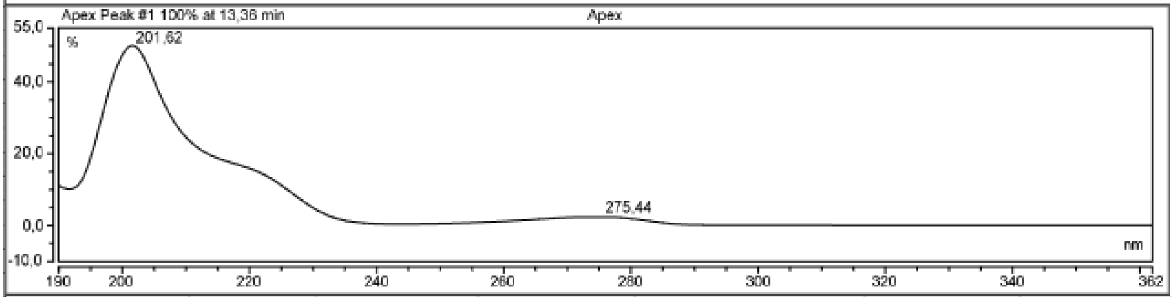

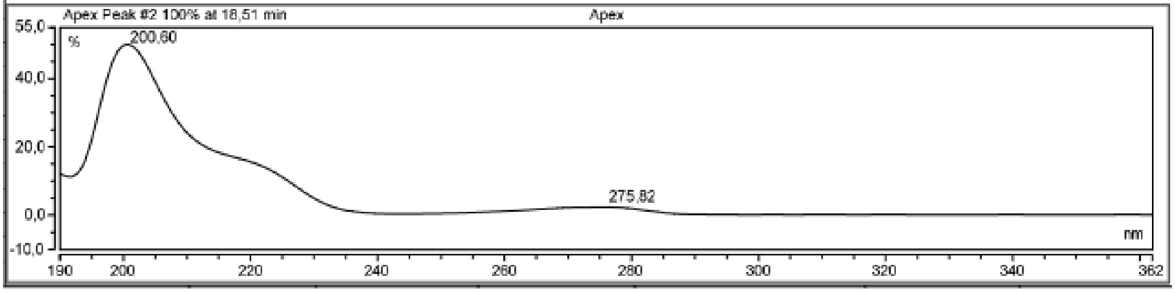

(*R,E*)-2-Chloro-5-methyl-4-(pent-3-en-2-yl)phenol (2p) & (*S,E*)-6-chloro-3-methyl-2-(pent-3-en-2-yl)phenol (3p)

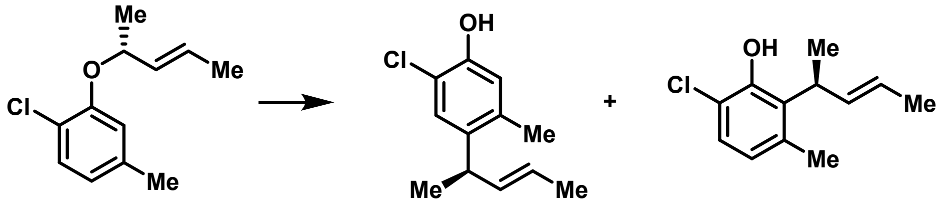

1p 2p 3p

The title compounds were synthesized from **1p** (102 mg, 0.48 mmol) following **general procedure B**. The reaction was directly purified by column chromatography (petroleum ether/ethyl acetate 30:1) to provide the *para*-product **2p** as orange oil in 40% yield (41 mg, 0.20 mmol) and the *ortho*-product **3p** as yellow oil in 47% yield (48 mg, 0.23 mmol).

(*R,E*)-2-Chloro-5-methyl-4-(pent-3-en-2-yl)phenol (2p)

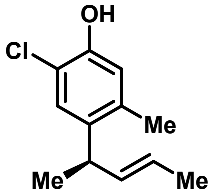

$[\alpha]^{20}$= - 24.22 (c 1.25, CH_2Cl_2).

^{1}H NMR (400 MHz, $CDCl_3$) δ 7.09 (s, 1H), 6.83 (s, 1H), 5.62 – 5.45 (m, 1H), 5.45 – 5.29 (m, 2H), 3.52 (p, *J* = 6.9 Hz, 1H), 2.25 (s, 3H), 1.67 (dt, *J* = 6.2, 1.4 Hz, 3H), 1.27 (d, *J* = 7.0 Hz, 3H).

^{13}C NMR (101 MHz, $CDCl_3$) δ 149.1, 138.0, 136.3, 135.4, 126.6, 124.0, 117.9, 117.2, 37.3, 20.9, 19.2, 18.0.

HRMS (ESI): exact mass calculated for $C_{12}H_{14}ClO^-$ [(M - H)$^-$], 209.0739 (100.0%), 211.0709 (32.0%); found 209.0740 (100.0%), 211.0707 (30.3%).

84% *ee* (determined by chiral HPLC: Chiralcel® OJ-3 column, n-Heptane/iPrOH = 99.5:0.5, 0.7 mL/min, λ = 287.3 nm, 25 °C), major enantiomer. t_r = 20.59 min, minor enantiomer. t_r = 22.48 min.

^{1}H NMR (400 MHz, $CDCl_3$)

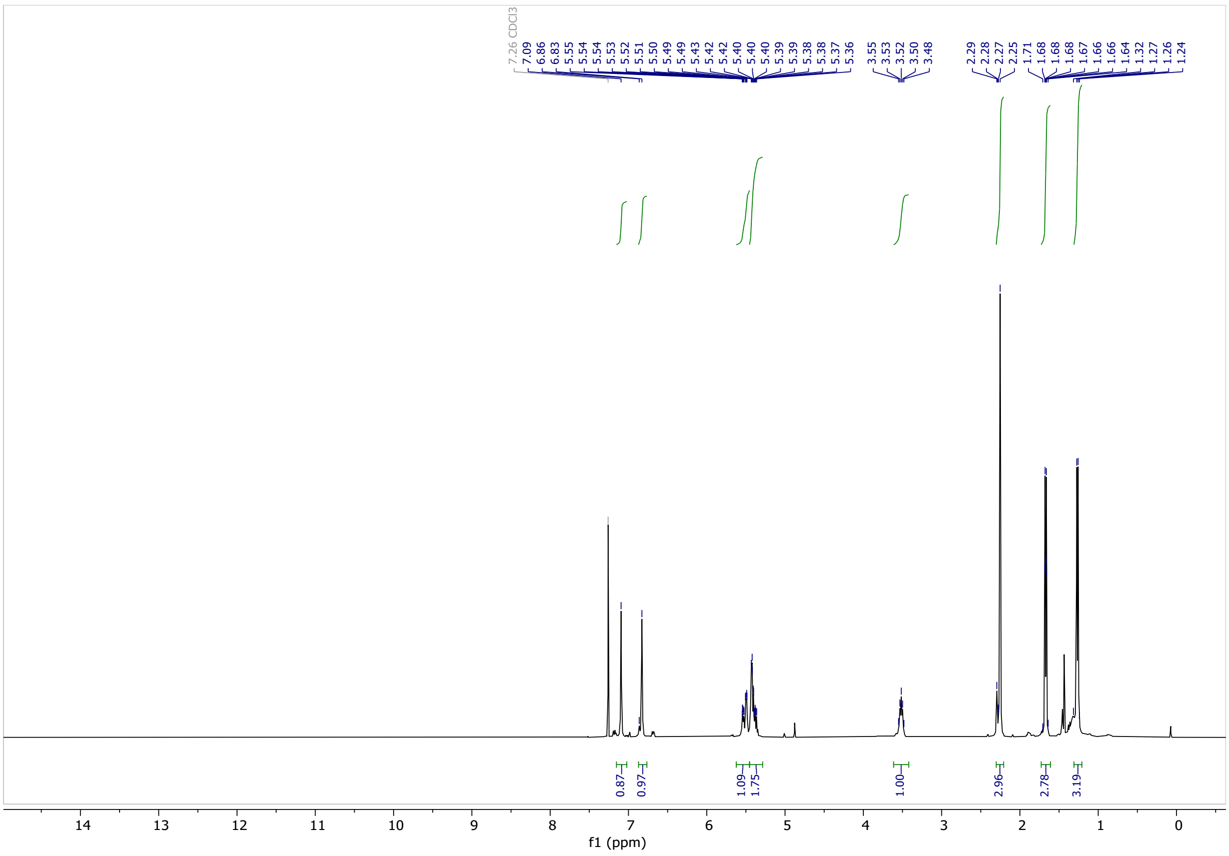

^{13}C NMR (101 MHz, $CDCl_3$)

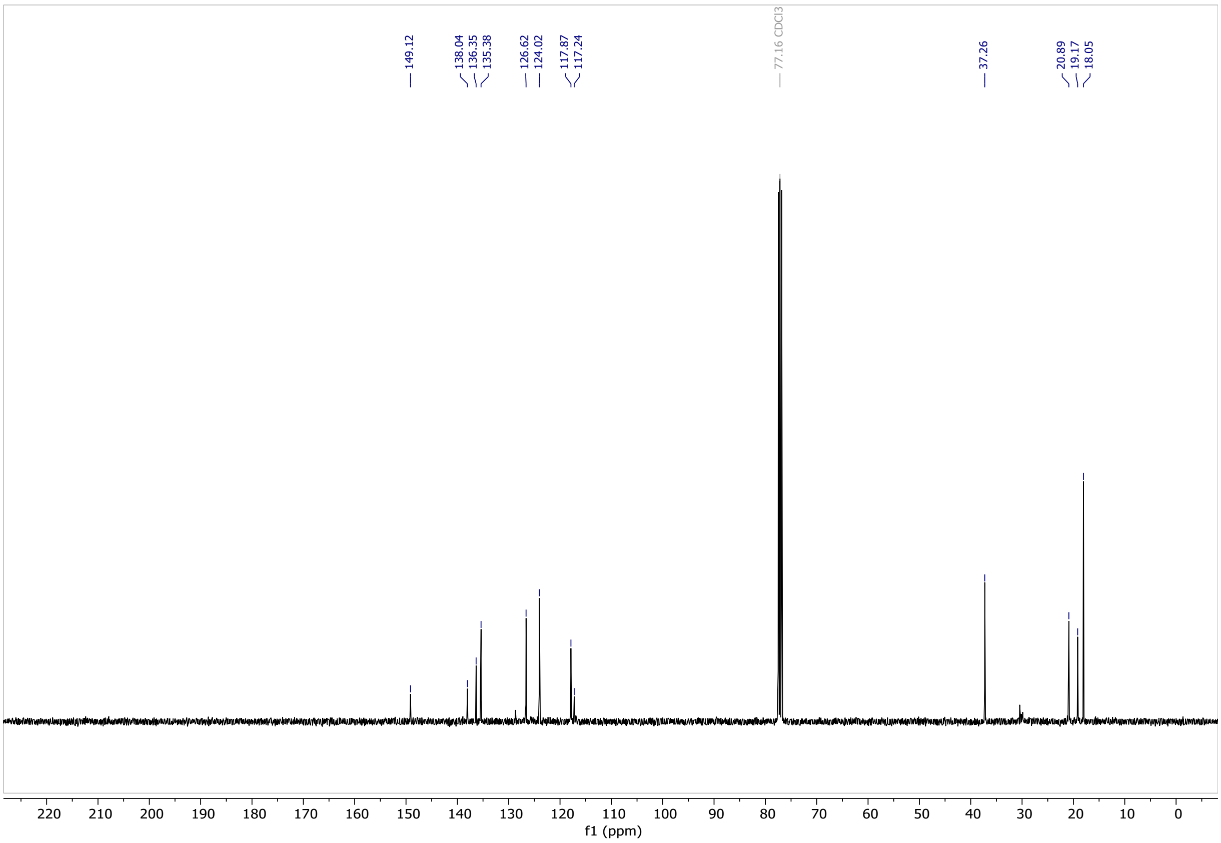
149.12
138.04
136.35
135.38
126.62
124.02
117.87
117.24
77.16 CDCl3
37.26
20.89
19.17
18.05
220 210 200 190 180 170 160 150 140 130 120 110 100 90 80 70 60 50 40 30 20 10 0
f1 (ppm)

Chromatogram and Results

Instrument Method:	**Heptane_IPA_99.5_0.5_0.7mlmin_25C_45min-MK**	*B %:*	**0,5**
Column:	**OJ3**	*C %:*	**0,0**
Run Time (min):	**45,00**	*D %:*	**0,0**
Channel:	**UV_VIS_1**		
Wavelength:	**287,26**		

Chromatogram

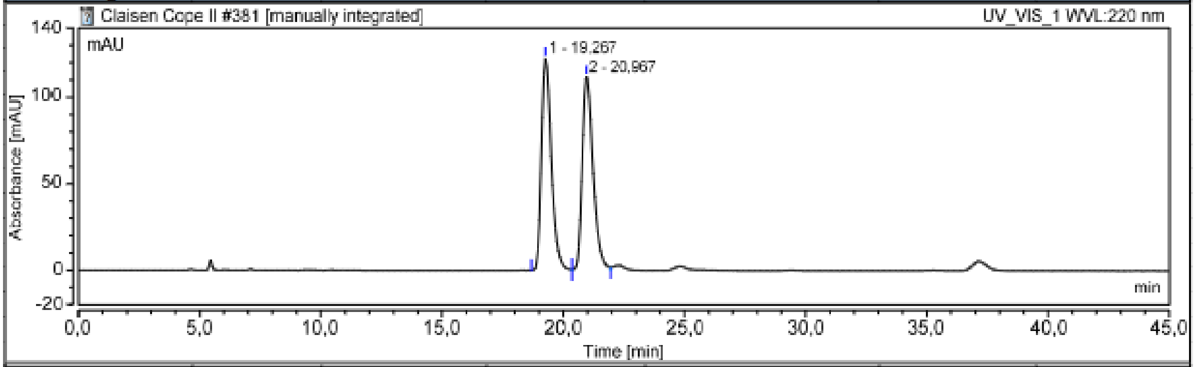

Integration Results

No.	Peak Name	Retention Time min	Area mAU*min	Height mAU	Relative Area %	Relative Height %
1		19,267	57,434	122,689	49,85	52,29
2		20,967	57,770	111,925	50,15	47,71
**Total:**			**115,204**	**234,614**	**100,00**	**100,00**

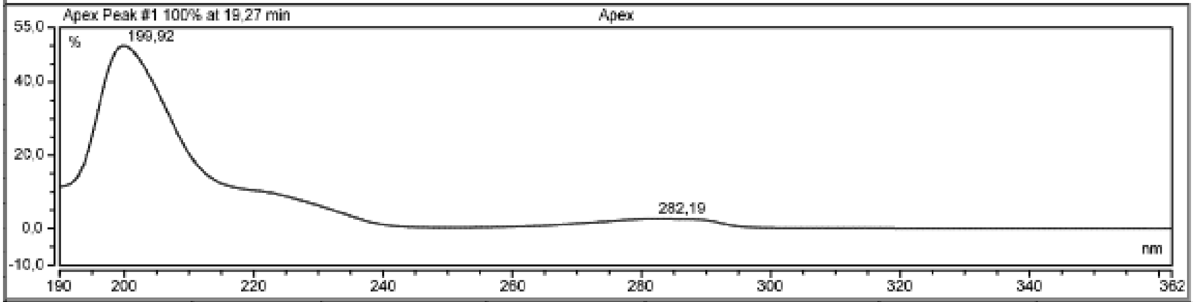

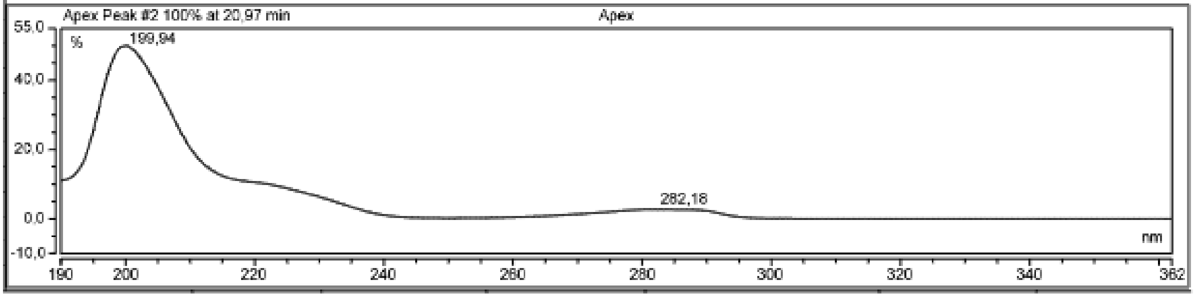

Chromatogram and Results

Instrument Method:	**Heptane_IPA_99.5_0.5_0.7mlmin_25C_45min-MK**	*B %:*	**0,5**
Column:	**OJ3**	*C %:*	**0,0**
Run Time (min):	**45,00**	*D %:*	**0,0**
Channel:	**UV_VIS_1**		
Wavelength:	**287,26**		

Chromatogram

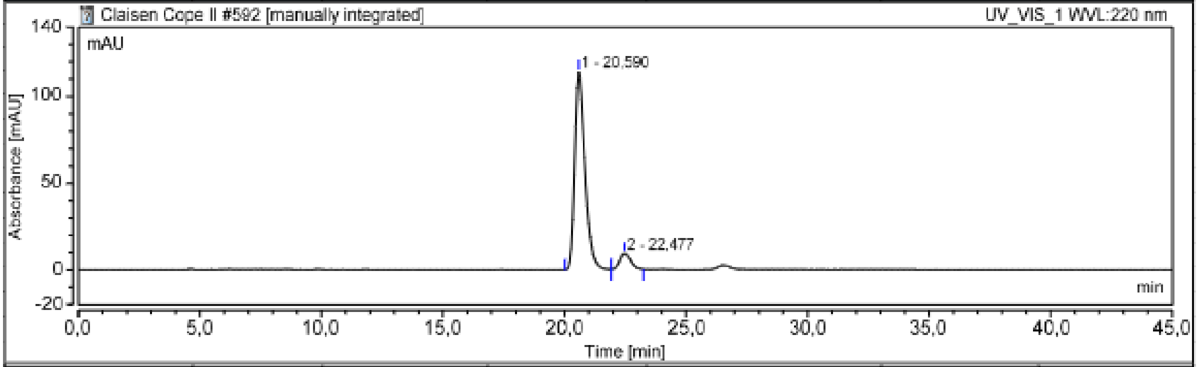

Integration Results

No.	Peak Name	Retention Time min	Area mAU*min	Height mAU	Relative Area %	Relative Height %
1		20,590	56,029	114,494	92,00	92,52
2		22,477	4,869	9,250	8,00	7,48
**Total:**			**60,899**	**123,744**	**100,00**	**100,00**

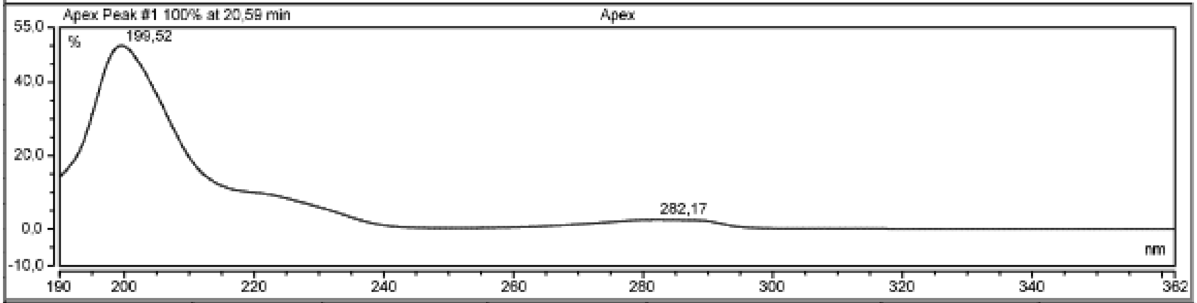

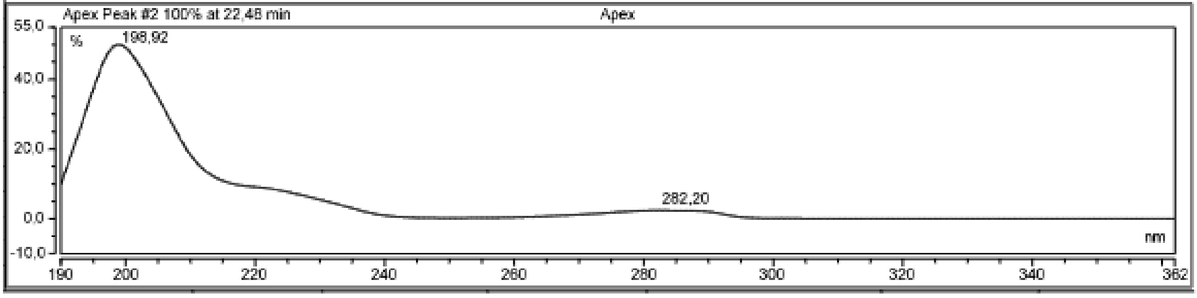

(*S,E*)-6-Chloro-3-methyl-2-(pent-3-en-2-yl)phenol (3p)

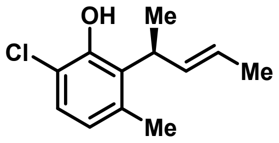

$[\alpha]^{20}$= - 2.38 (c 2.40, CH_2Cl_2).

^{1}H NMR (400 MHz, $CDCl_3$) δ 7.07 (d, *J* = 8.2 Hz, 1H), 6.66 (d, *J* = 8.3, 0.7 Hz, 1H), 5.97 – 5.88 (m, 2H), 5.68 – 5.54 (m, 1H), 3.89 – 3.78 (m, 1H), 2.30 (d, *J* = 0.7 Hz, 3H), 1.71 (dt, *J* = 6.4, 1.6 Hz, 3H), 1.39 (d, *J* = 7.2 Hz, 3H).

^{13}C NMR (101 MHz, $CDCl_3$) δ 149.9, 135.8, 134.3, 131.2, 126.5, 125.3, 123.2, 118.8, 36.5, 20.5, 18.1, 17.8.

HRMS (ESI): exact mass calculated for $C_{12}H_{14}ClO^-$ [(M - H)$^-$], 209.0739 (100.0%), 211.0709 (32.0%); found 209.0738 (100.0%), 211.0701 (28.6%).

81% *ee* (determined by chiral HPLC: Chiralcel® OJ-3 column, n-Heptane/EtOH = 99:1, 0.7 mL/min, λ = 287.3 nm, 25 °C), major enantiomer. t_r = 8.37 min, minor enantiomer. t_r = 9.75 min.

^{1}H NMR (400 MHz, $CDCl_3$)

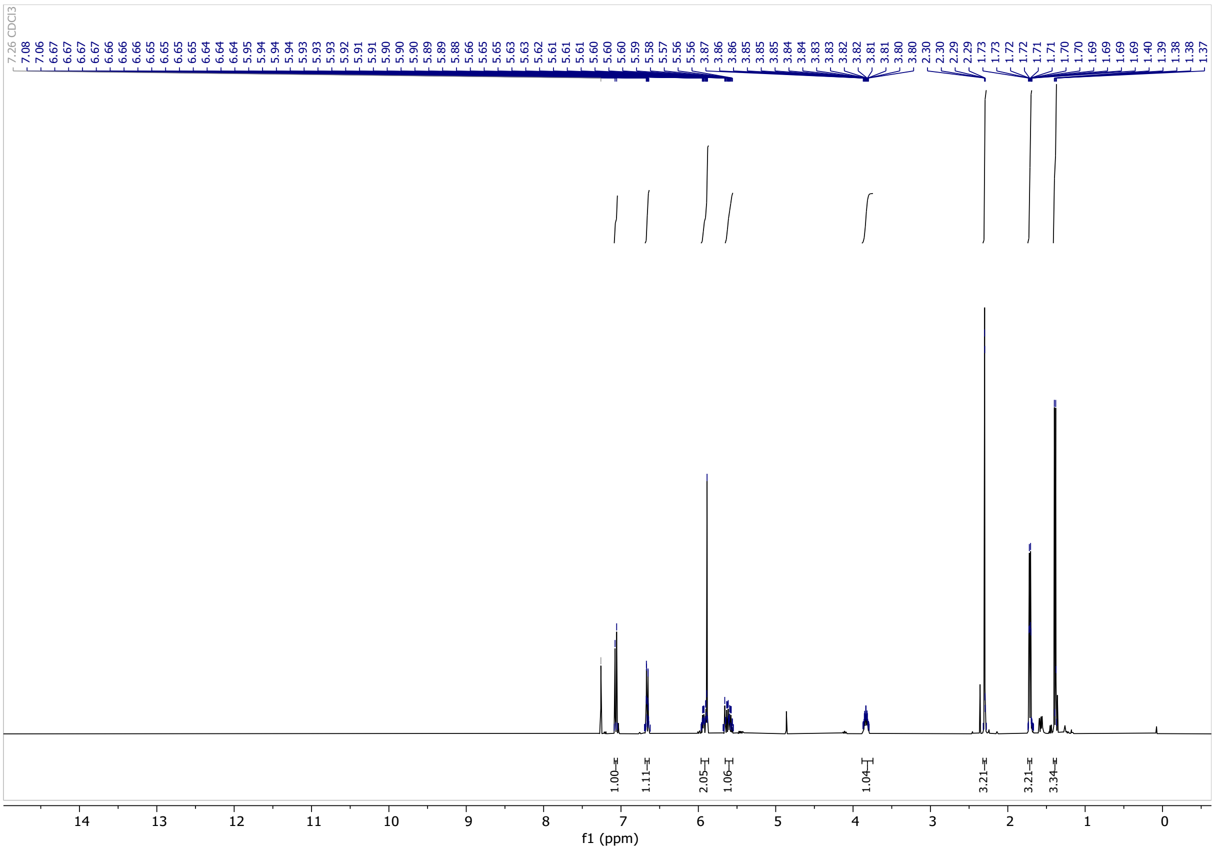

^{13}C NMR (101 MHz, $CDCl_3$)

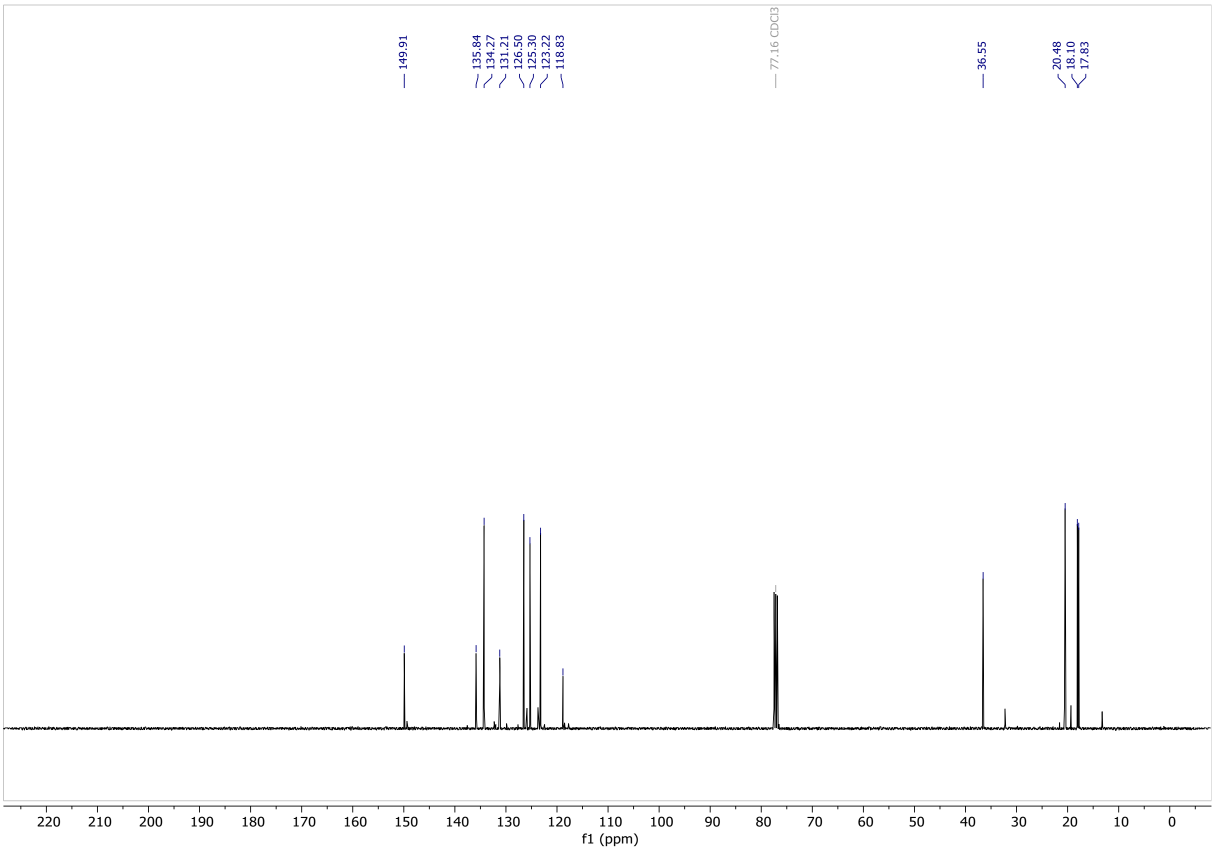

Chromatogram and Results

Instrument Method:	**Heptane_EtOH_99_1_0.7mlmin_25C_30min-MK**	*B %:*	**0,0**
Column:	**OJ-3**	*C %:*	**0,0**
Run Time (min):	**30,00**	*D %:*	**1,0**
Channel:	**UV_VIS_1**		
Wavelength:	**287,26**		

Chromatogram

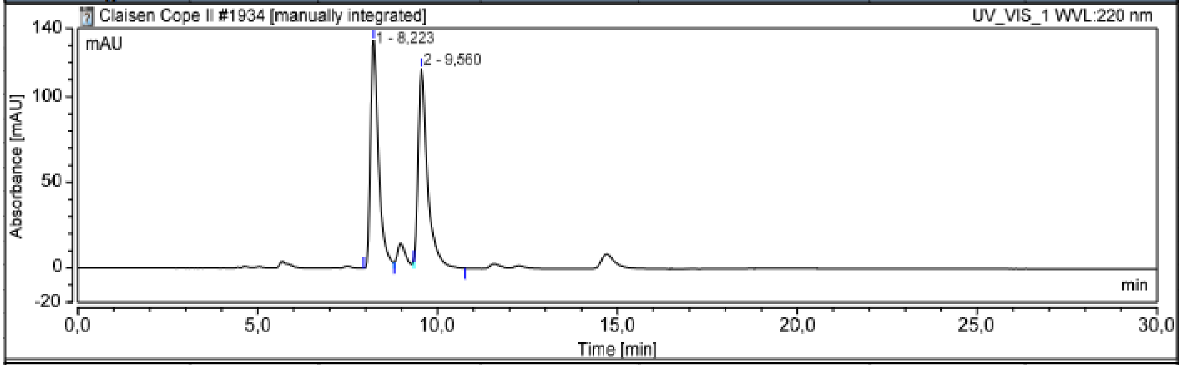

Integration Results

No.	Peak Name	Retention Time min	Area mAU*min	Height mAU	Relative Area %	Relative Height %
1		8,223	34,146	133,141	49,05	53,36
2		9,560	35,469	116,379	50,95	46,64
**Total:**			**69,616**	**249,520**	**100,00**	**100,00**

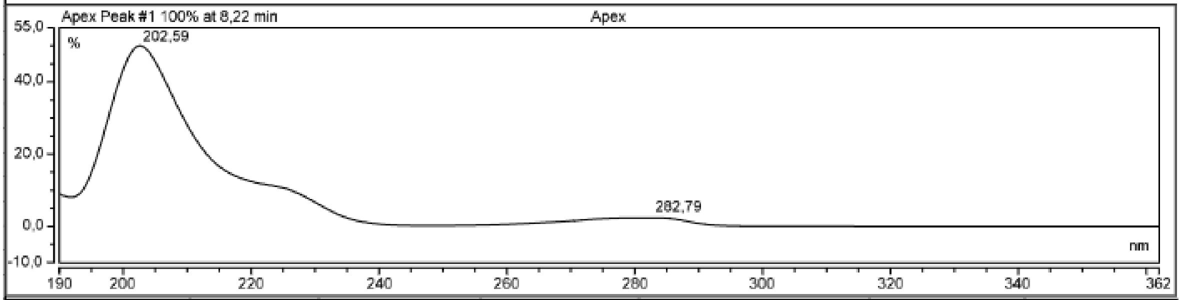

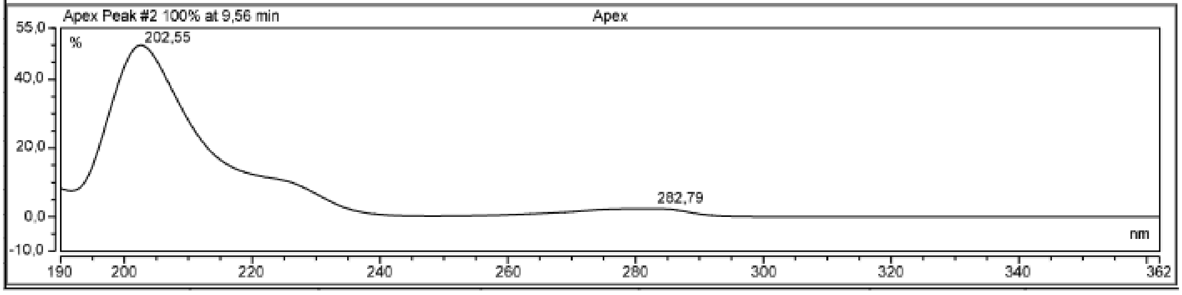

Chromatogram and Results

Instrument Method:	**Heptane_EtOH_99_1_0.7mlmin_25C_20min-MK**	*B %:*	**0,0**
Column:	**OJ-3**	*C %:*	**0,0**
Run Time (min):	**20,00**	*D %:*	**1,0**
Channel:	**UV_VIS_1**		
Wavelength:	**287,26**		

Chromatogram

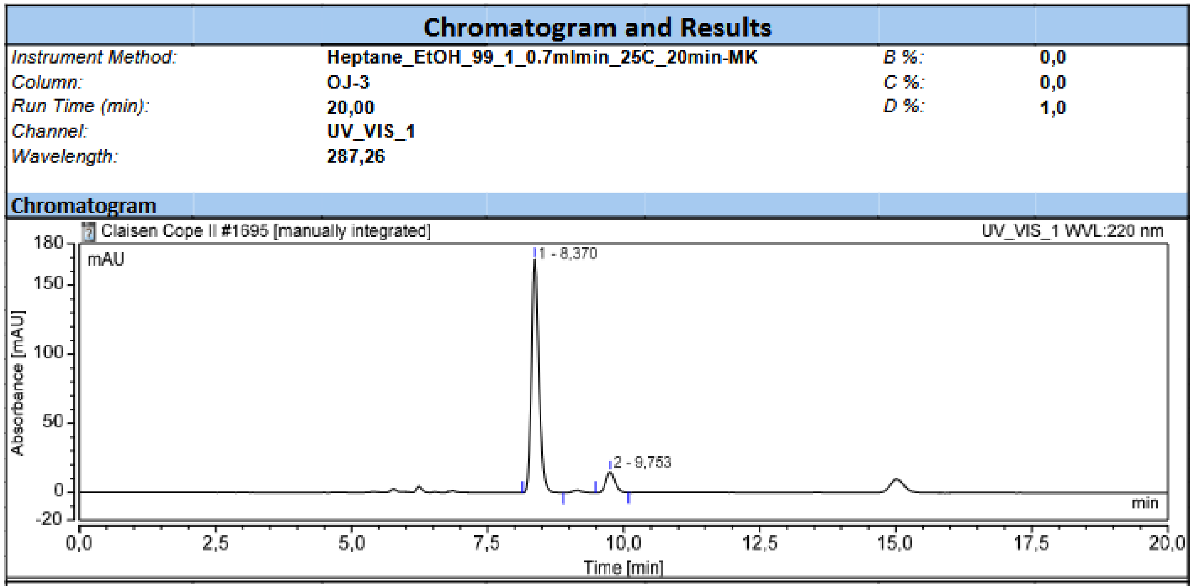

Integration Results

No.	Peak Name	Retention Time<br>min	Area<br>mAU*min	Height<br>mAU	Relative Area<br>%	Relative Height<br>%
1		8,370	26,833	169,220	90,66	91,90
2		9,753	2,763	14,922	9,34	8,10
**Total:**			**29,596**	**184,141**	**100,00**	**100,00**

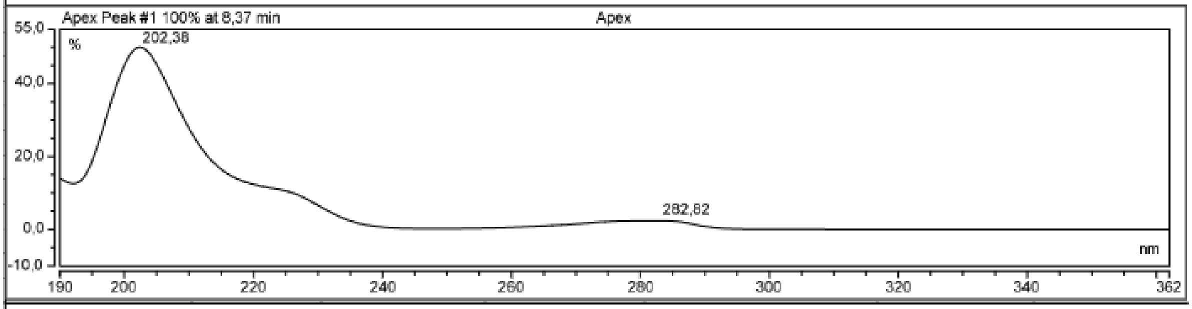

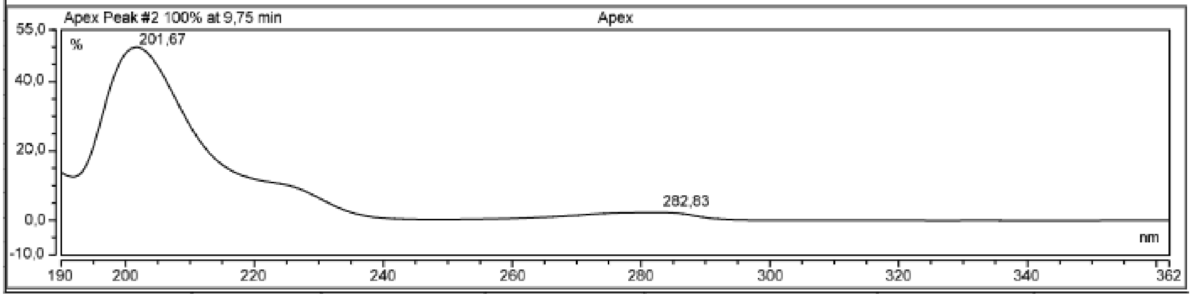

(*R,E*)-2-Bromo-5-methyl-4-(pent-3-en-2-yl)phenol (2q) & (*S,E*)-6-bromo-3-methyl-2-(pent-3-en-2-yl)phenol (3q)

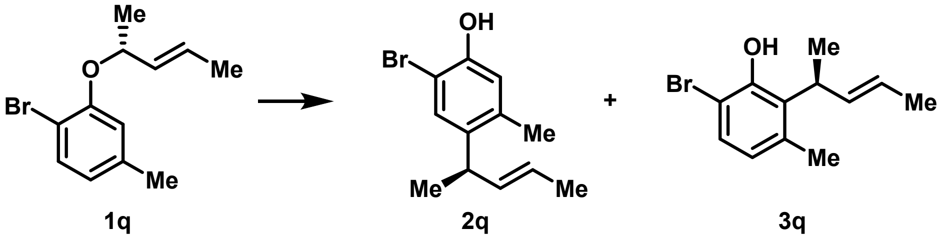

The title compounds were synthesized from **1q** (100 mg, 0.39 mmol) following **general procedure B**. The reaction was directly purified by column chromatography (petroleum ether/ethyl acetate 30:1) to provide the *para*-product **2q** as colorless oil in 28% yield (28 mg, 0.11 mmol) and the *ortho*-product **3q** as colorless oil in 60% yield (60 mg, 0.24 mmol).

(*R,E*)-2-Bromo-5-methyl-4-(pent-3-en-2-yl)phenol (2q)

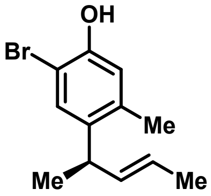

$[\alpha]^{20}$= - 12.61 (c 1.40, CH_2Cl_2).

^{1}H NMR (400 MHz, $CDCl_3$) δ 7.21 (s, 1H), 6.81 (d, *J* = 0.8 Hz, 1H), 5.51 (ddq, *J* = 15.3, 6.0, 1.4 Hz, 1H), 5.39 (dqd, *J* = 15.3, 6.2, 1.3 Hz, 1H), 5.26 (d, *J* = 1.0 Hz, 1H), 3.57 – 3.42 (m, 1H), 2.24 (d, *J* = 0.7 Hz, 3H), 1.67 (dt, *J* = 6.2, 1.4 Hz, 3H), 1.30 – 1.20 (m, 3H).

^{13}C NMR (101 MHz, $CDCl_3$) δ 150.0, 138.5, 137.2, 135.4, 129.5, 124.1, 117.7, 107.4, 37.2, 20.9, 19.3, 18.1.

HRMS (ESI): exact mass calculated for $C_{12}H_{14}BrO^-$ [(M - H)$^-$], 253.0234 (100.0%), 255.0213 (97.3%); found 253.0238 (100.0%), 255.0257 (96.5%).

84% *ee* (determined by chiral HPLC: Chiralcel® OJ-3 column, n-Heptane/EtOH = 99.5:0.5, 0.7 mL/min, λ = 287.3 nm, 25 °C), major enantiomer. t_r = 18.21 min, minor enantiomer. t_r = 19.11 min.

^{1}H NMR (400 MHz, $CDCl_3$)

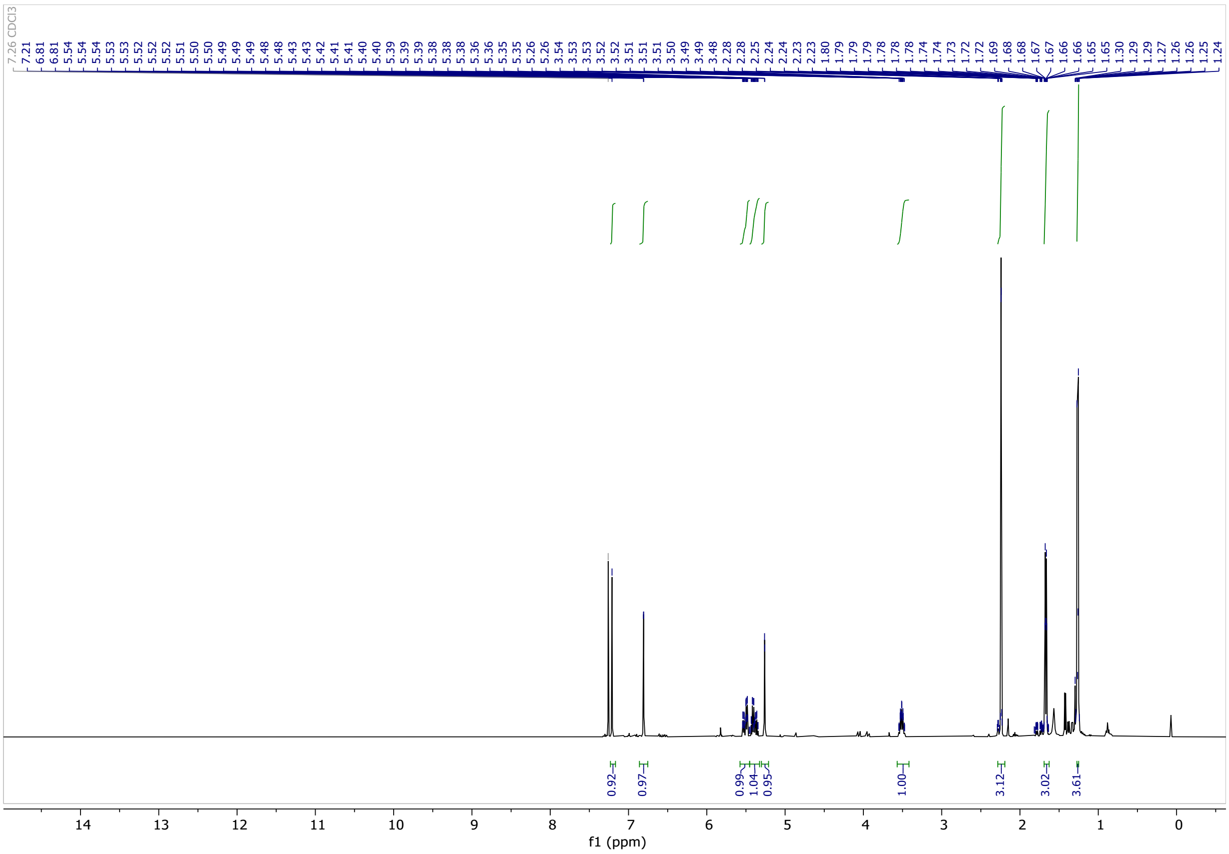

^{13}C NMR (101 MHz, $CDCl_3$)

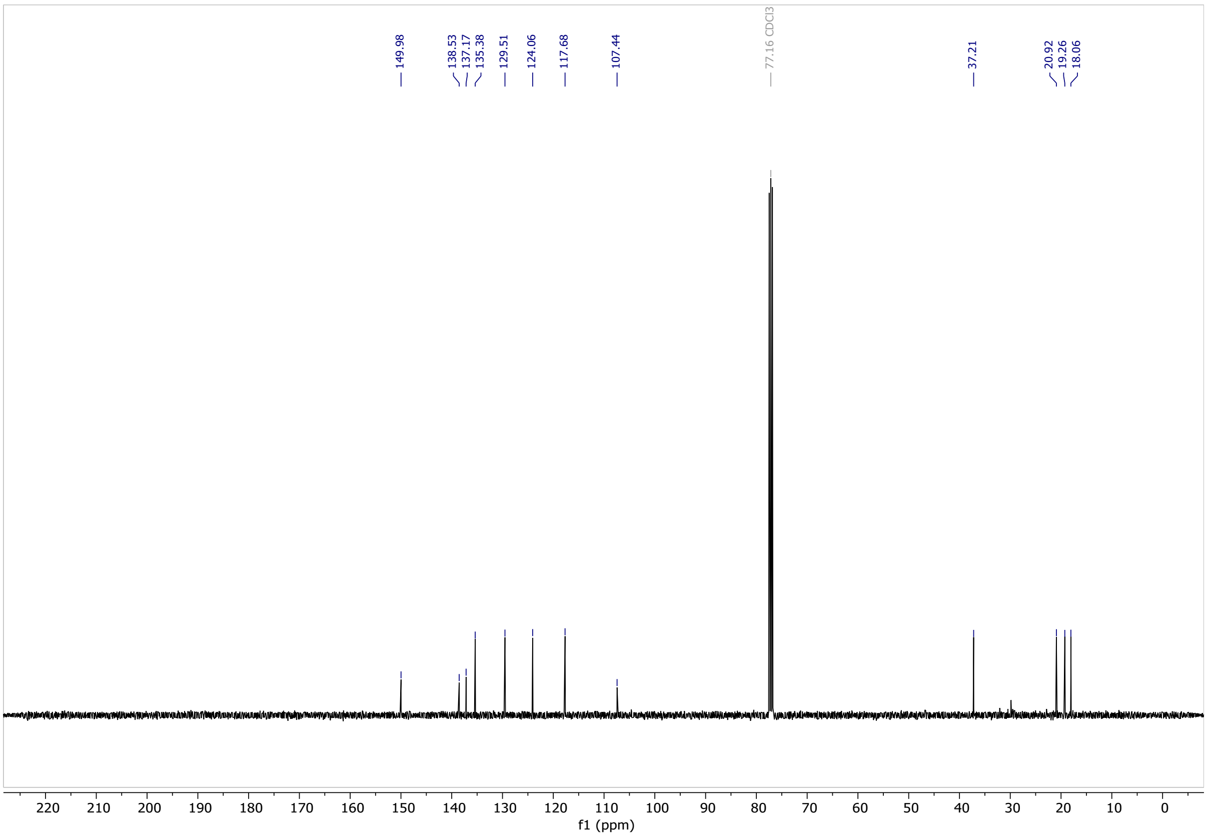

Chromatogram and Results

Instrument Method:	**Heptane_EtOH_99.5_0.5_0.7mlmin_25C_30min**	*B %:*	**0,0**
Column:	**OJ3**	*C %:*	**0,0**
Run Time (min):	**30,00**	*D %:*	**0,5**
Channel:	**UV_VIS_1**		
Wavelength:	**287,26**		

Chromatogram

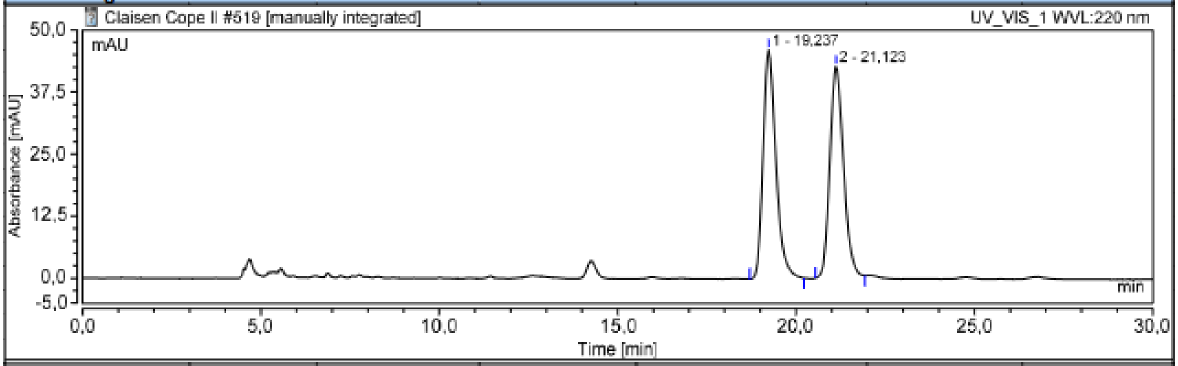

Integration Results

No.	Peak Name	Retention Time min	Area mAU*min	Height mAU	Relative Area %	Relative Height %
1		19,237	19,195	46,091	50,69	52,08
2		21,123	18,675	42,410	49,31	47,92
**Total:**			**37,870**	**88,501**	**100,00**	**100,00**

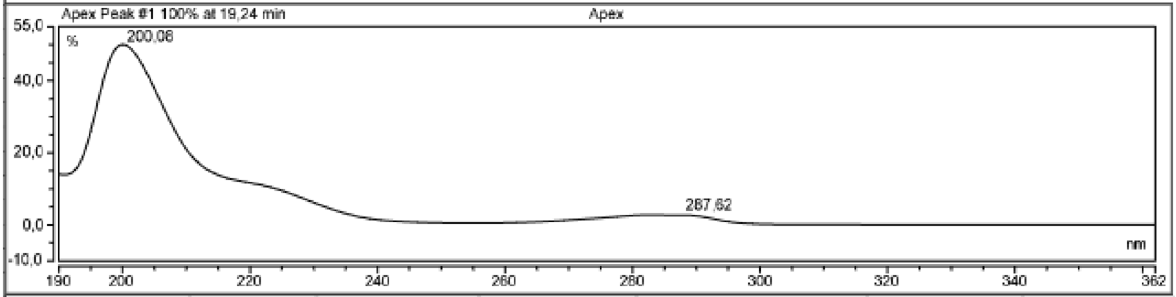

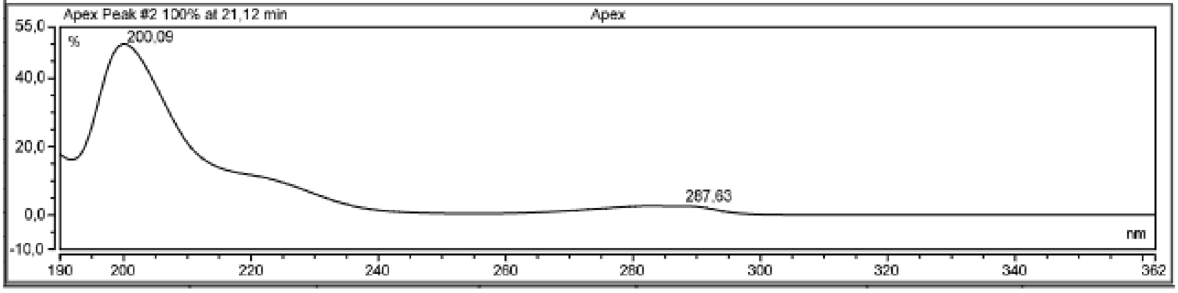

Chromatogram and Results

Instrument Method:	**Heptane_EtOH_99.5_0.5_0.7mlmin_25C_30min**	*B %:*	**0,0**
Column:	**OJ3**	*C %:*	**0,0**
Run Time (min):	**30,00**	*D %:*	**0,5**
Channel:	**UV_VIS_1**		
Wavelength:	**287,26**		

Chromatogram

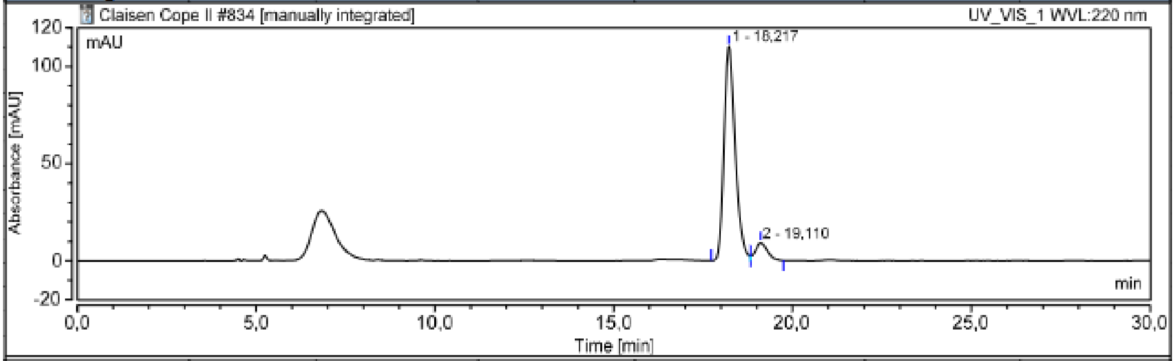

Integration Results

No.	Peak Name	Retention Time<br>min	Area<br>mAU*min	Height<br>mAU	Relative Area<br>%	Relative Height<br>%
1		18,217	40,081	110,118	91,87	92,32
2		19,110	3,546	9,160	8,13	7,68
**Total:**			**43,627**	**119,278**	**100,00**	**100,00**

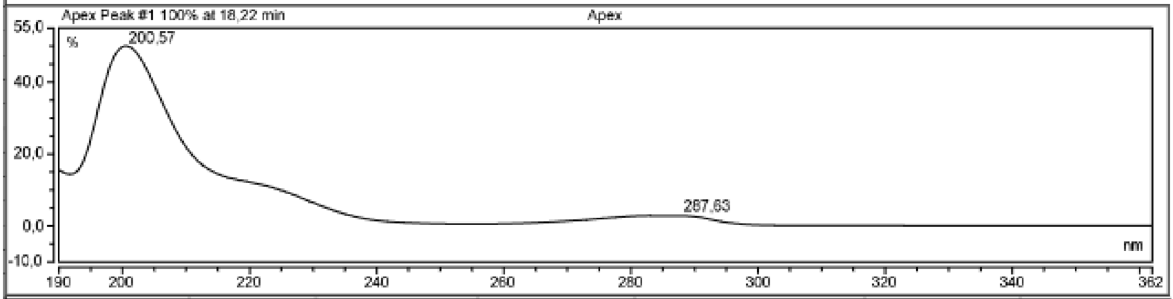

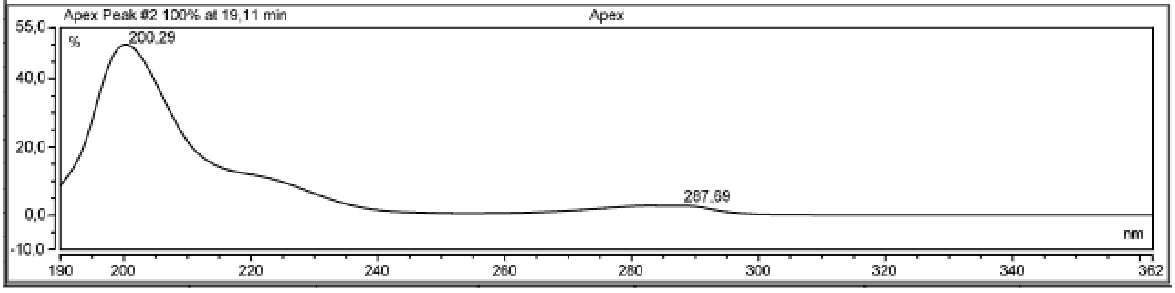

(*S,E*)-6-Bromo-3-methyl-2-(pent-3-en-2-yl)phenol (3q)

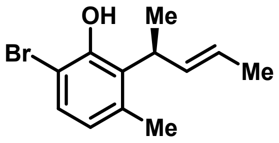

Compound **3q** was obtained as 1.3:1.0 *E/Z* mixture as measured by the ratio of the major (*E*)-isomer δ 7.22 (d, *J* = 8.2 Hz, 1H, integral= 1.30), to the minor (*Z*)-isomer δ 7.00 (d, *J* = 7.8 Hz, 1H, integral= 1.00); ^{1}H NMR (400 MHz, $CDCl_3$) δ 7.22 (d, *J* = 8.2 Hz, 1.30H), 7.00 (d, *J* = 7.8 Hz, 1.00H), 6.78 (dd, *J* = 7.8, 0.8 Hz, 1.09H), 6.63 – 6.59 (m, 1.70H), 5.95 – 5.88 (m, 2.96H), 5.75 (s, 0.97H), 5.69 – 5.58 (m, 2.77H), 5.57 – 5.43 (m, 1.45H), 3.90 – 3.74 (m, 2.72H), 2.37 (s, 3.17H), 2.29 (d, *J* = 0.6 Hz, 4.82H), 1.75 – 1.66 (m, 9.04H), 1.39 (d, *J* = 7.2 Hz, 5.53H), 1.31 (d, *J* = 7.1 Hz, 3.80H).

^{13}C NMR (101 MHz, $CDCl_3$) δ 150.8, 149.6, 136.6, 136.0, 135.1, 134.3, 131.2, 130.9, 129.7, 126.3, 125.5, 124.4, 123.9, 122.3, 113.8, 109.1, 36.6, 36.4, 23.1, 20.5, 20.2, 18.1, 18.1, 17.7.

HRMS (ESI): exact mass calculated for $C_{12}H_{14}BrO^-$ [$(M - H)^-$], 253.0234 (100.0%), 255.0213 (97.3%); found 253.0238 (100.0%), 255.0217 (96.1%).

HRMS (ESI): exact mass calculated for $C_{12}H_{14}BrO^-$ [$(M - H)^-$], 253.0234; found 253.0230.

86% *ee* (determined by chiral HPLC: Chiralcel® OJ-3 column, n-Hexane/EtOH = 99.9:0.1, 0.3 mL/min, λ = 287.3 nm, 25 °C), major enantiomer. t_r = 26.54 min, minor enantiomer. t_r = 29.43 min.

^{1}H NMR (400 MHz, $CDCl_3$)

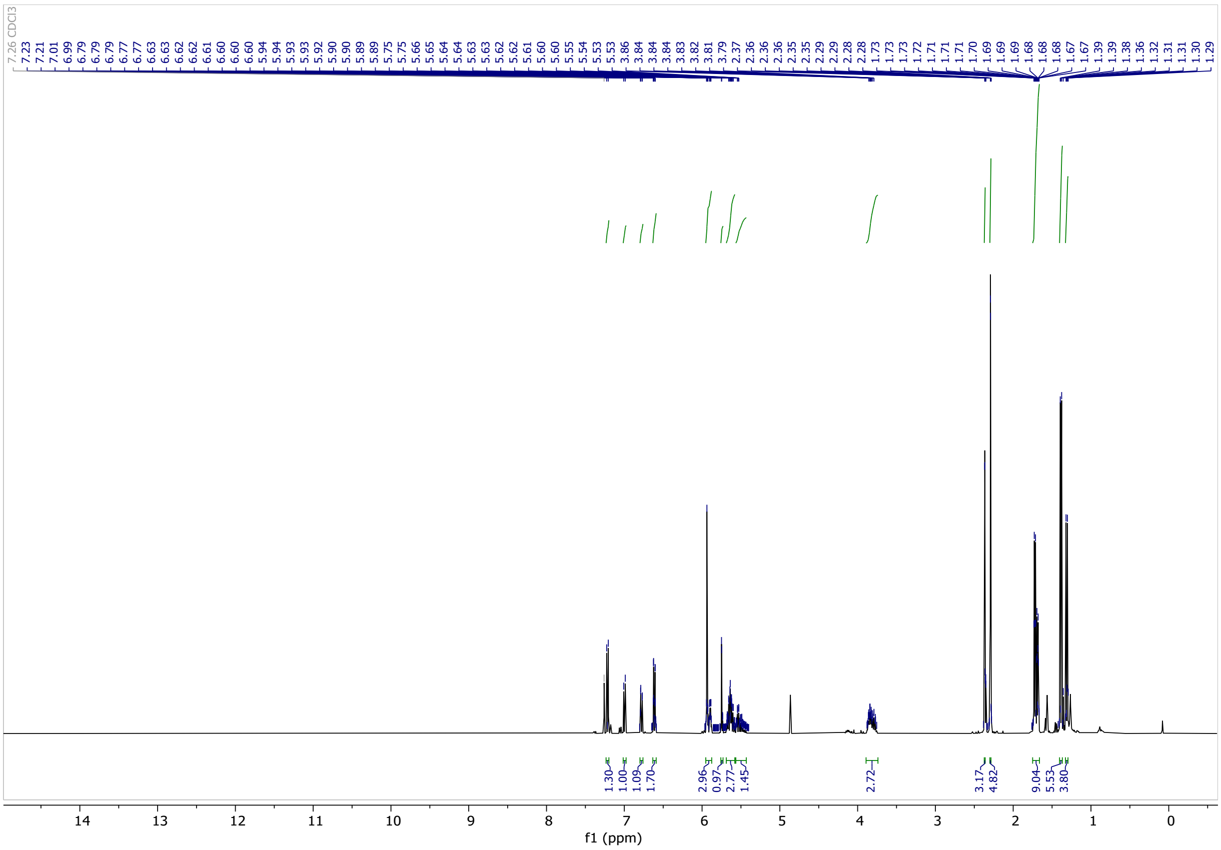

^{13}C NMR (101 MHz, $CDCl_3$)

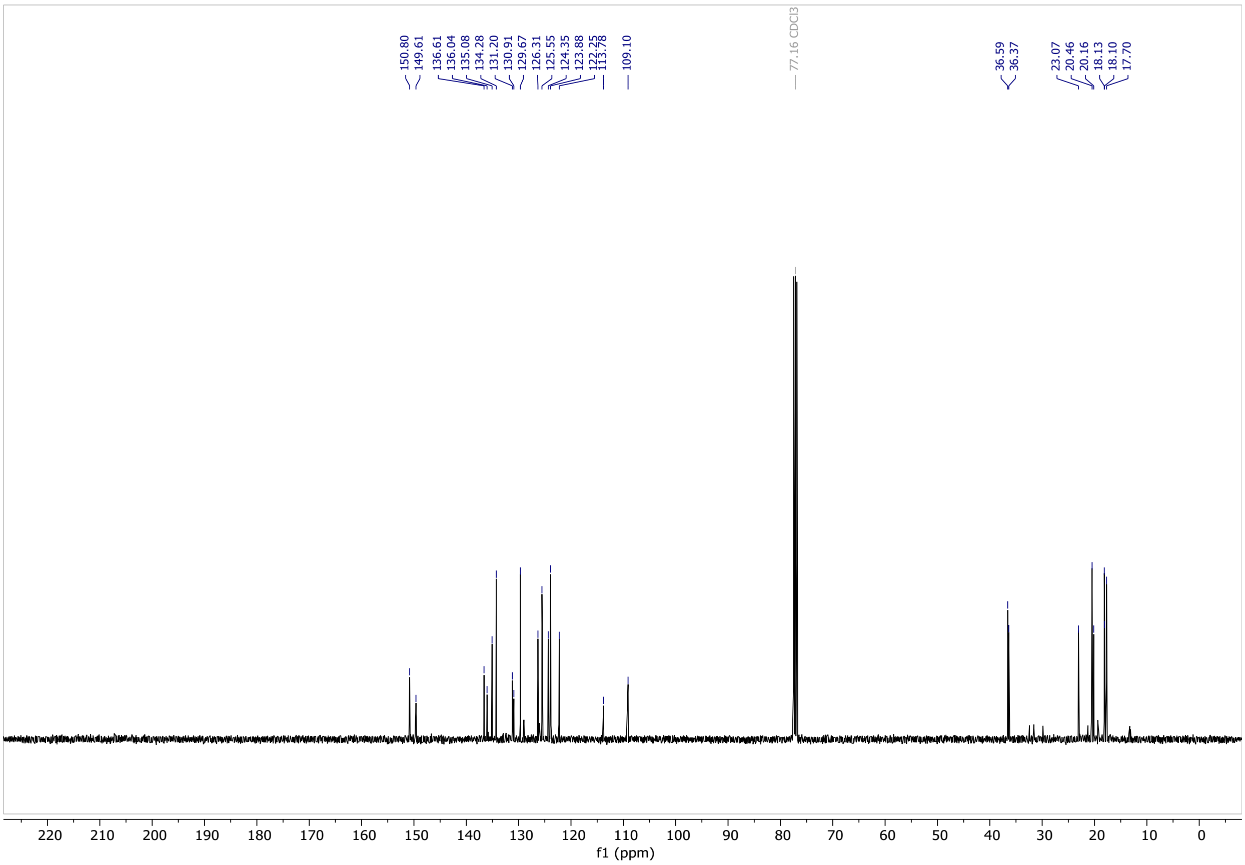
150.80
149.61
136.61
136.04
135.08
134.28
131.20
130.91
129.67
126.31
125.55
124.35
123.88
122.25
113.78
109.10
77.16 CDCl3
36.59
36.37
23.07
20.46
20.16
18.13
18.10
17.70
220 210 200 190 180 170 160 150 140 130 120 110 100 90 80 70 60 50 40 30 20 10 0
f1 (ppm)

Chromatogram and Results

Instrument Method:	**Hexane_EtOH_99.9_0.1_0.3mlmin_25C_45min-MK**	*B %:*	**0,0**
Column:	**OJ3**	*C %:*	**99,9**
Run Time (min):	**45,00**	*D %:*	**0,1**
Channel:	**UV_VIS_1**		
Wavelength:	**287,26**		

Chromatogram

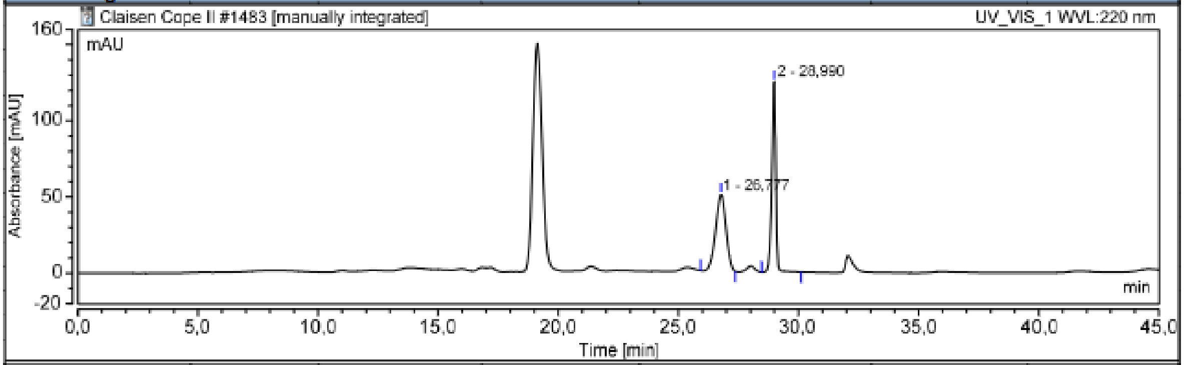

Integration Results

No.	Peak Name	Retention Time min	Area mAU*min	Height mAU	Relative Area %	Relative Height %
1		26,777	25,095	50,374	51,87	28,69
2		28,990	23,282	125,225	48,13	71,31
**Total:**			**48,377**	**175,599**	**100,00**	**100,00**

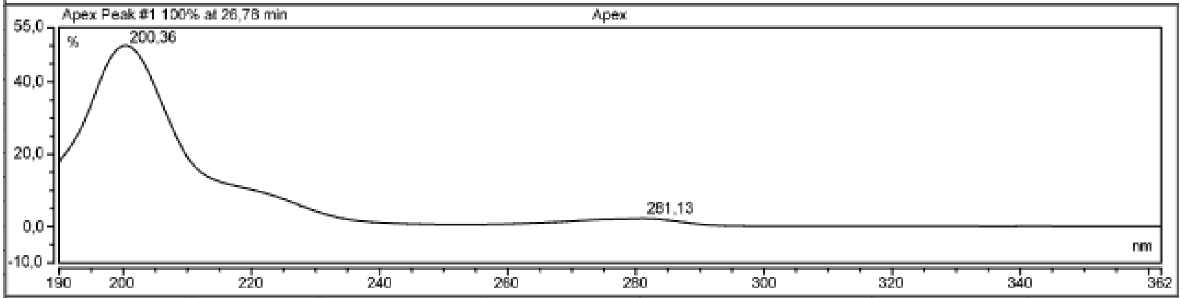

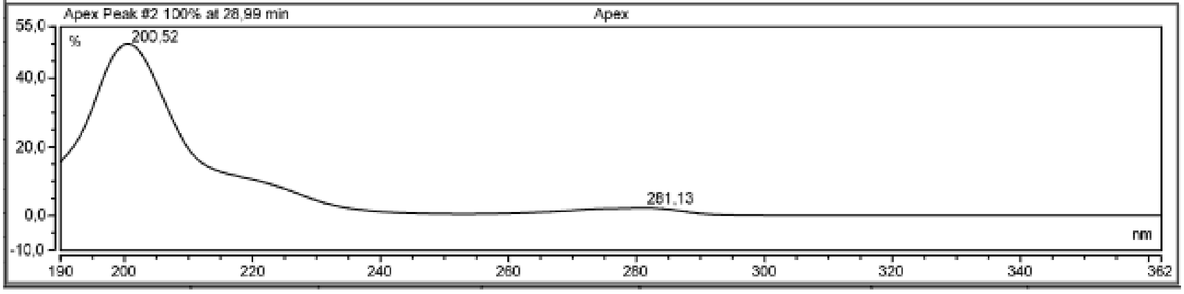

Chromatogram and Results

Instrument Method:	**Hexane_EtOH_99.9_0.1_0.3mlmin_25C_45min-MK**	*B %:*	**0,0**
Column:	**OJ3**	*C %:*	**99,9**
Run Time (min):	**45,00**	*D %:*	**0,1**
Channel:	**UV_VIS_1**		
Wavelength:	**287,26**		

Chromatogram

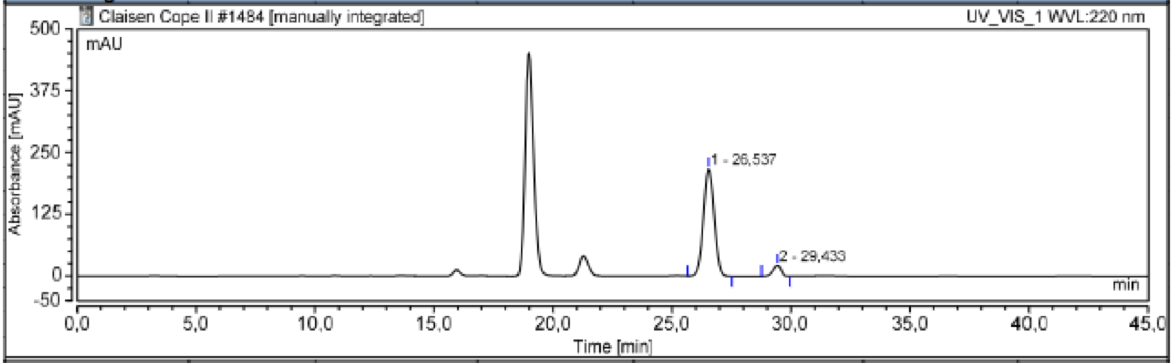

Integration Results

No.	Peak Name	Retention Time<br>min	Area<br>mAU*min	Height<br>mAU	Relative Area<br>%	Relative Height<br>%
1		26,537	114,665	217,356	92,81	90,75
2		29,433	8,884	22,154	7,19	9,25
**Total:**			**123,549**	**239,510**	**100,00**	**100,00**

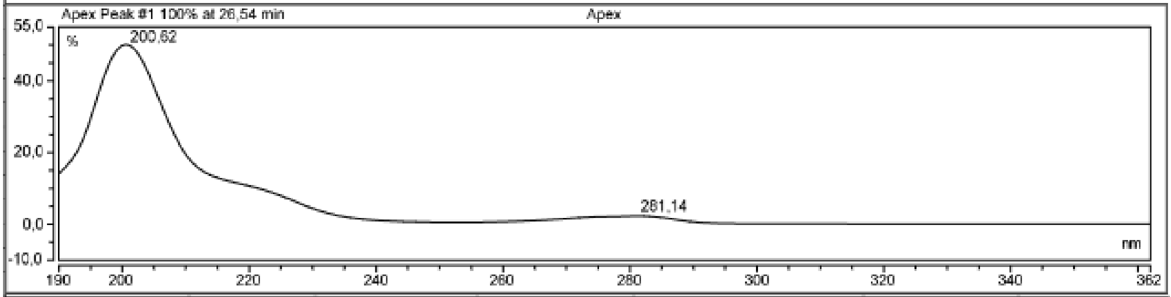

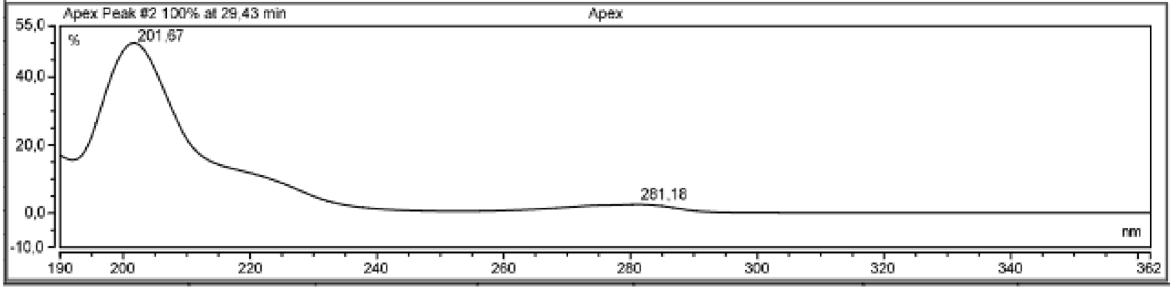

(*R,E*)-5-Bromo-2-methoxy-4-(pent-3-en-2-yl)phenol (2r) & (*S,E*)-3-bromo-6-methoxy-2-(pent-3-en-2-yl)phenol (3r)

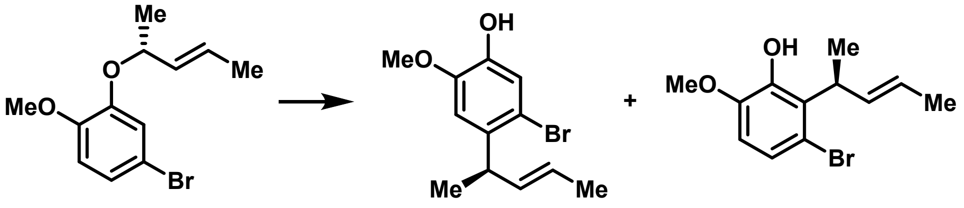

The title compounds were synthesized from **1r** (100 mg, 0.37 mmol) following **general procedure B**. The reaction was directly purified by column chromatography (petroleum ether/ethyl acetate 30:1) to provide the *para*-product **2r** as colorless oil in 26% yield (26 mg, 0.10 mmol) and the *ortho*-product **3r** as colorless oil in 73% yield (73 mg, 0.27 mmol).

(*R,E*)-5-Bromo-2-methoxy-4-(pent-3-en-2-yl)phenol (2r)

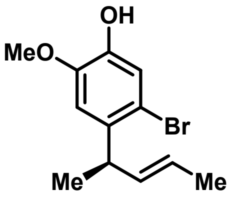

$[\alpha]^{20}$= + 2.01 (c 1.20, CH_2Cl_2).

^{1}H NMR (400 MHz, $CDCl_3$) δ 7.09 (s, 1H), 6.68 (s, 1H), 5.62 – 5.48 (m, 2H), 5.47 (s, 1H), 3.87 (s, 3H), 3.83 (tdd, *J* = 8.2, 4.0, 2.7 Hz, 1H), 1.69 (dt, *J* = 6.0, 1.3 Hz, 3H), 1.27 (d, *J* = 7.0 Hz, 3H).

^{13}C NMR (101 MHz, $CDCl_3$) δ 146.30, 144.47, 136.88, 134.87, 124.44, 118.48, 114.55, 110.02, 56.18, 40.46, 20.65, 18.15.

HRMS (ESI): exact mass calculated for $C_{12}H_{14}BrO_2^-$ [(M - H)$^-$], 269.0183; found 269.0181.

85% *ee* (determined by chiral HPLC: Chiralcel® OJ-3 column, n-Heptane/EtOH = 99:1, 0.7 mL/min, λ = 287.3 nm, 25 °C), major enantiomer. t_r = 17.71 min, minor enantiomer. t_r = 20.25 min.

^{1}H NMR (400 MHz, $CDCl_3$)

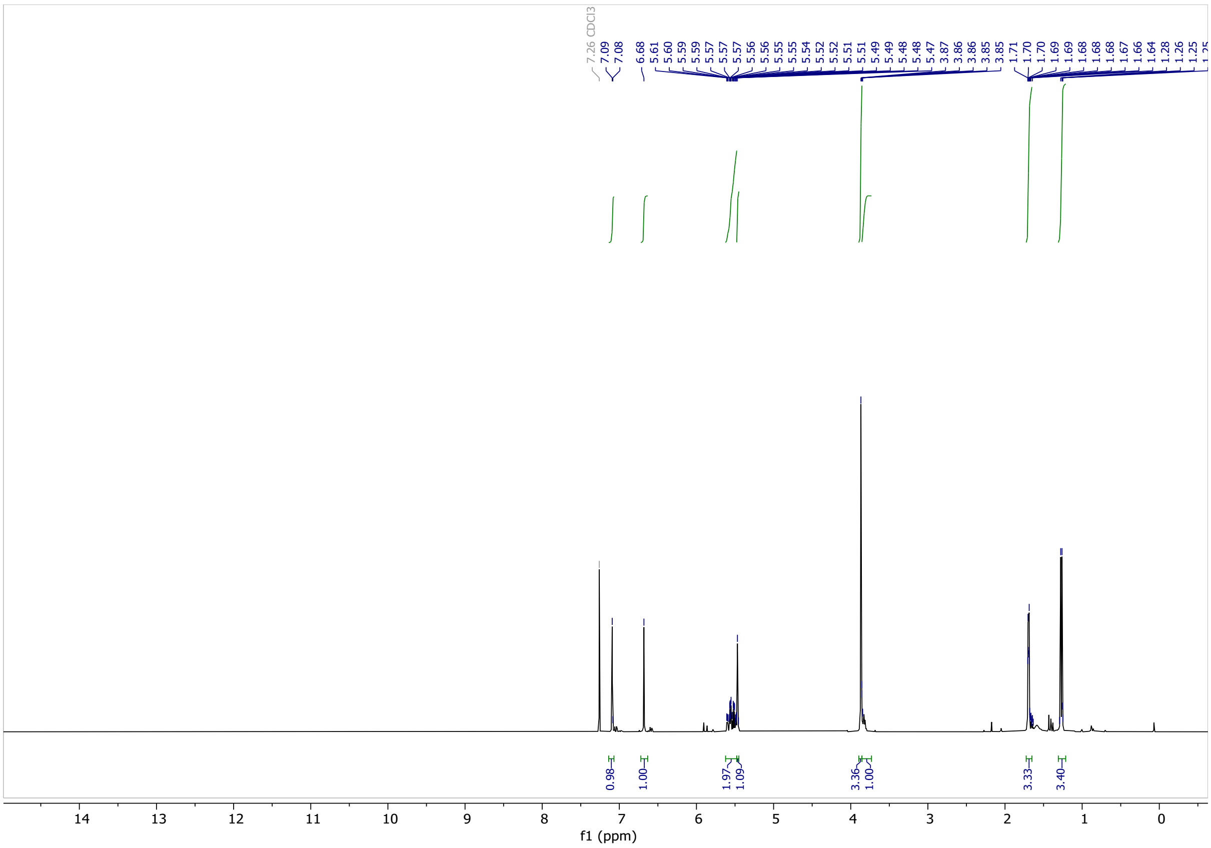

^{13}C NMR (101 MHz, $CDCl_3$)

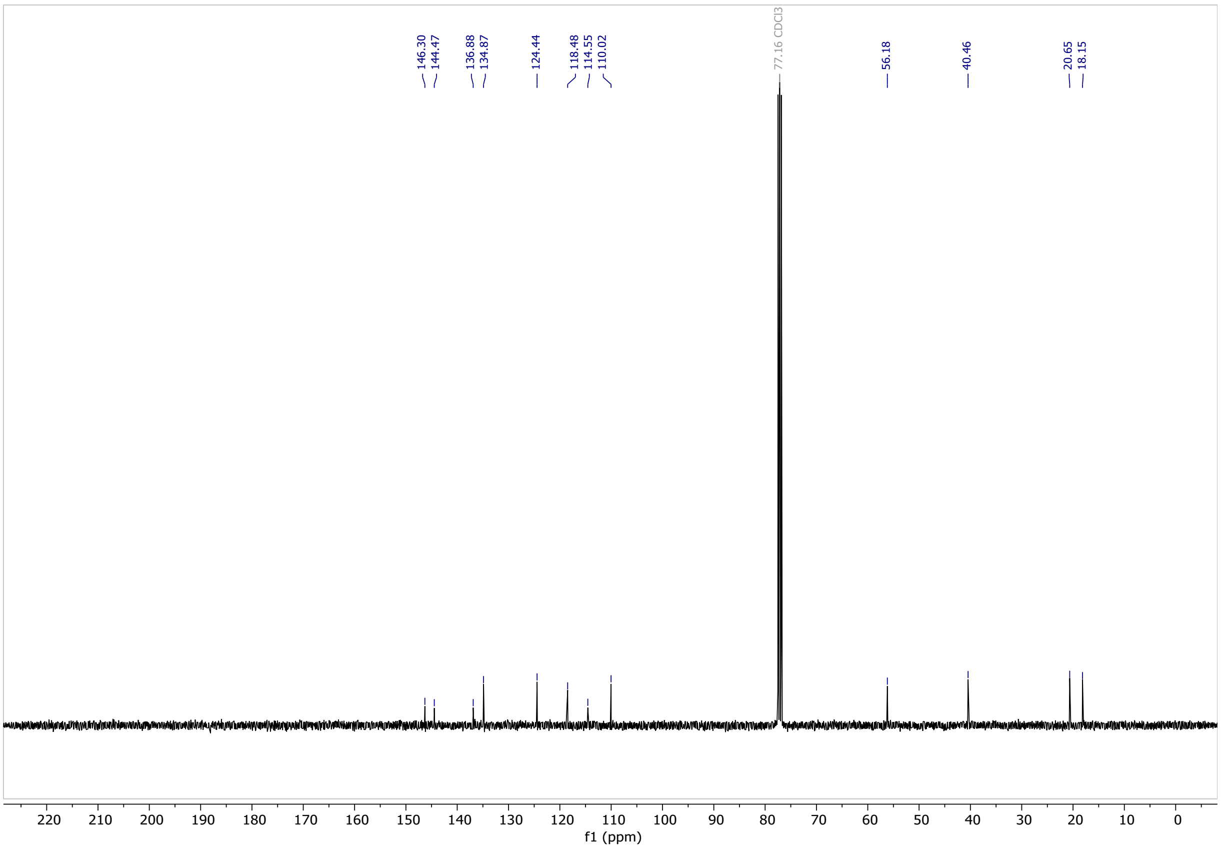
146.30
144.47
136.88
134.87
124.44
118.48
114.55
110.02
77.16 CDCl3
56.18
40.46
20.65
18.15
220 210 200 190 180 170 160 150 140 130 120 110 100 90 80 70 60 50 40 30 20 10 0
f1 (ppm)

Chromatogram and Results

Instrument Method:	**Heptane_EtOH_99_1_0.7mlmin_25C_40min-MK**	*B %:*	**0,0**
Column:	**OJ3**	*C %:*	**0,0**
Run Time (min):	**40,00**	*D %:*	**1,0**
Channel:	**UV_VIS_1**		
Wavelength:	**287,26**		

Chromatogram

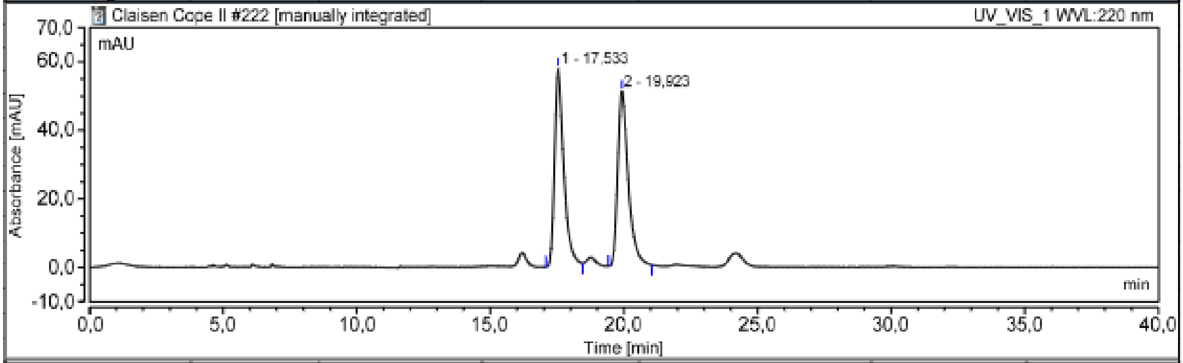

Integration Results

No.	Peak Name	Retention Time min	Area mAU*min	Height mAU	Relative Area %	Relative Height %
1		17,533	23,433	57,925	50,19	53,17
2		19,923	23,255	51,027	49,81	46,83
**Total:**			**46,688**	**108,953**	**100,00**	**100,00**

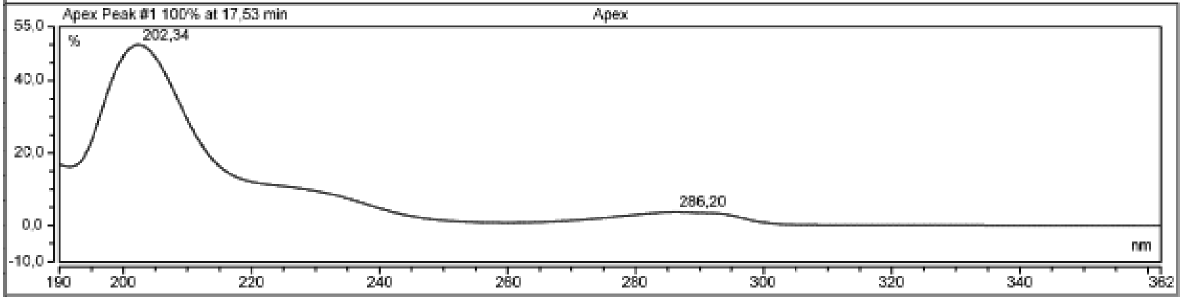

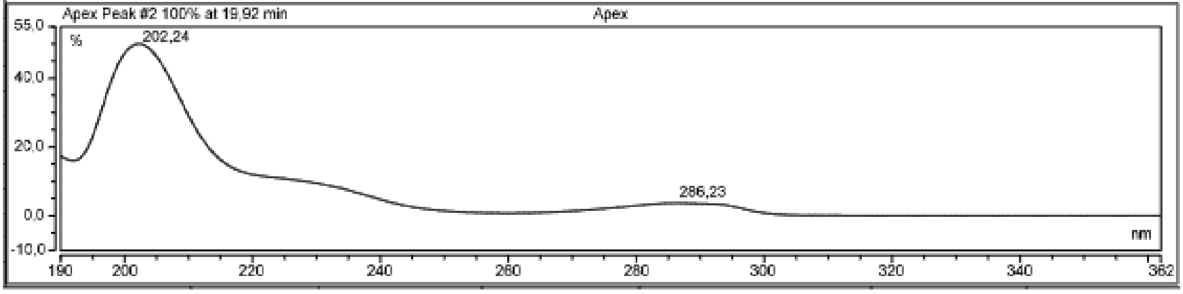

Chromatogram and Results

Instrument Method:	**Heptane_EtOH_99_1_0.7mlmin_25C_40min-MK**	*B %:*	**0,0**
Column:	**OJ3**	*C %:*	**0,0**
Run Time (min):	**40,00**	*D %:*	**1,0**
Channel:	**UV_VIS_1**		
Wavelength:	**287,26**		

Chromatogram

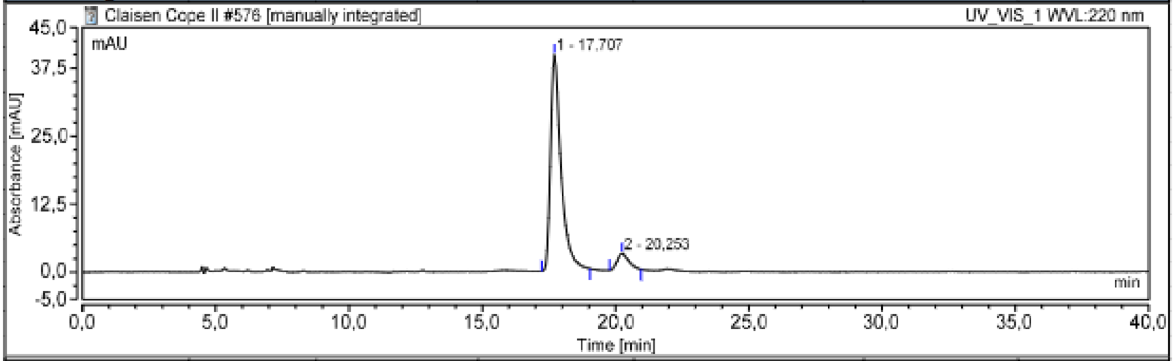

Integration Results

No.	Peak Name	Retention Time min	Area mAU*min	Height mAU	Relative Area %	Relative Height %
1		17,707	18,029	39,650	92,33	92,95
2		20,253	1,498	3,009	7,67	7,05
**Total:**			**19,527**	**42,659**	**100,00**	**100,00**

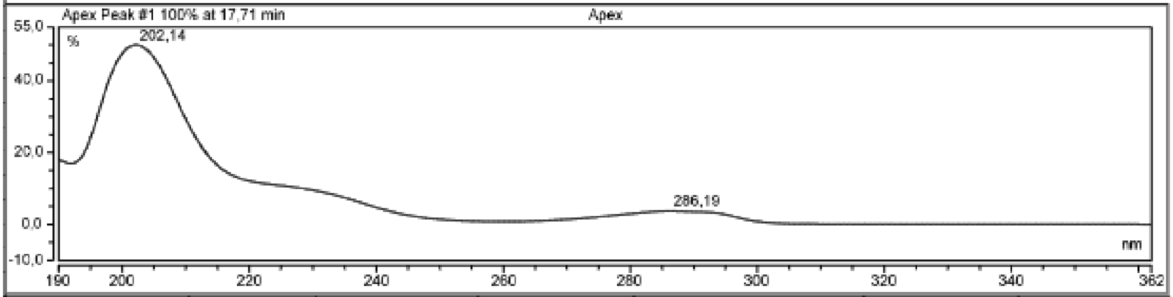

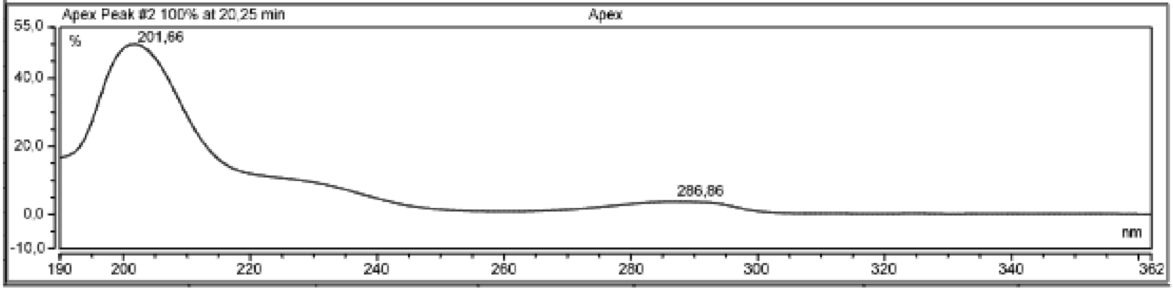

(*S,E*)-3-Bromo-6-methoxy-2-(pent-3-en-2-yl)phenol (3r)

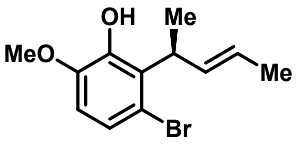

$[\alpha]^{20}$= - 10.19 (c 1.80, CH_2Cl_2).

Compound **3r** was obtained as 2.2:1.0 *E/Z* mixture as measured by the ratio of the major (*E*)-isomer δ 4.17 – 4.05 (m, 1H, integral= 2.22), to the minor (*Z*)-isomer δ 4.45 – 4.32 (m, 1H, integral= 1.00); ^{1}H NMR (400 MHz, $CDCl_3$) δ 7.08 – 7.03 (m, 2.89H), 6.62 – 6.55 (m, 3.08H), 6.06 (ddq, *J* = 10.5, 8.7, 1.8 Hz, 1.01H), 5.98 (ddq, *J* = 15.3, 7.0, 1.7 Hz, 2.17H), 5.92 (s, 2.03H), 5.88 (s, 0.99H), 5.59 (dqd, *J* = 15.4, 6.4, 1.4 Hz, 2.23H), 5.45 (dqd, *J* = 10.6, 6.8, 1.3 Hz, 1.04H), 4.45 – 4.32 (m, 1.0H), 4.17 – 4.05 (m, 2.22H), 3.85 (s, 9.53H), 1.70 – 1.65 (m, 9.43H), 1.43 (d, *J* = 7.1 Hz, 6.48H), 1.40 (d, *J* = 7.1 Hz, 3.25H).

^{13}C NMR (101 MHz, $CDCl_3$) δ 146.5, 146.3, 144.9, 144.8, 134.0, 133.7, 131.3, 130.7, 124.9, 123.7, 123.6, 123.6, 115.9, 115.6, 109.7, 109.5, 56.3, 19.0, 18.3, 18.0, 13.4.

(*E*)-isomer: 83% *ee* (determined by chiral HPLC: Chiralcel® OJ-3 column, n-Hexane/EtOH = 99:1, 0.7 mL/min, λ = 287.3 nm, 25 °C), major enantiomer. t_r = 14.39 min, minor enantiomer. t_r = 19.55 min.

HRMS (ESI): exact mass calculated for $C_{12}H_{14}BrO_2^-$ [(M - H)$^-$], 269.0183 (100.0%), 271.0162 (97.3%); found 269.0175 (100.0%), 271.0156 (94.2%).

(*Z*)-isomer: 84% *ee* (determined by chiral HPLC: Chiralcel® OJ-3 column, n-Hexane/EtOH = 99:1, 0.7 mL/min, λ = 287.3 nm, 25 °C), major enantiomer. t_r = 32.04 min, minor enantiomer. t_r = 16.17 min.

^{1}H NMR (400 MHz, $CDCl_3$)

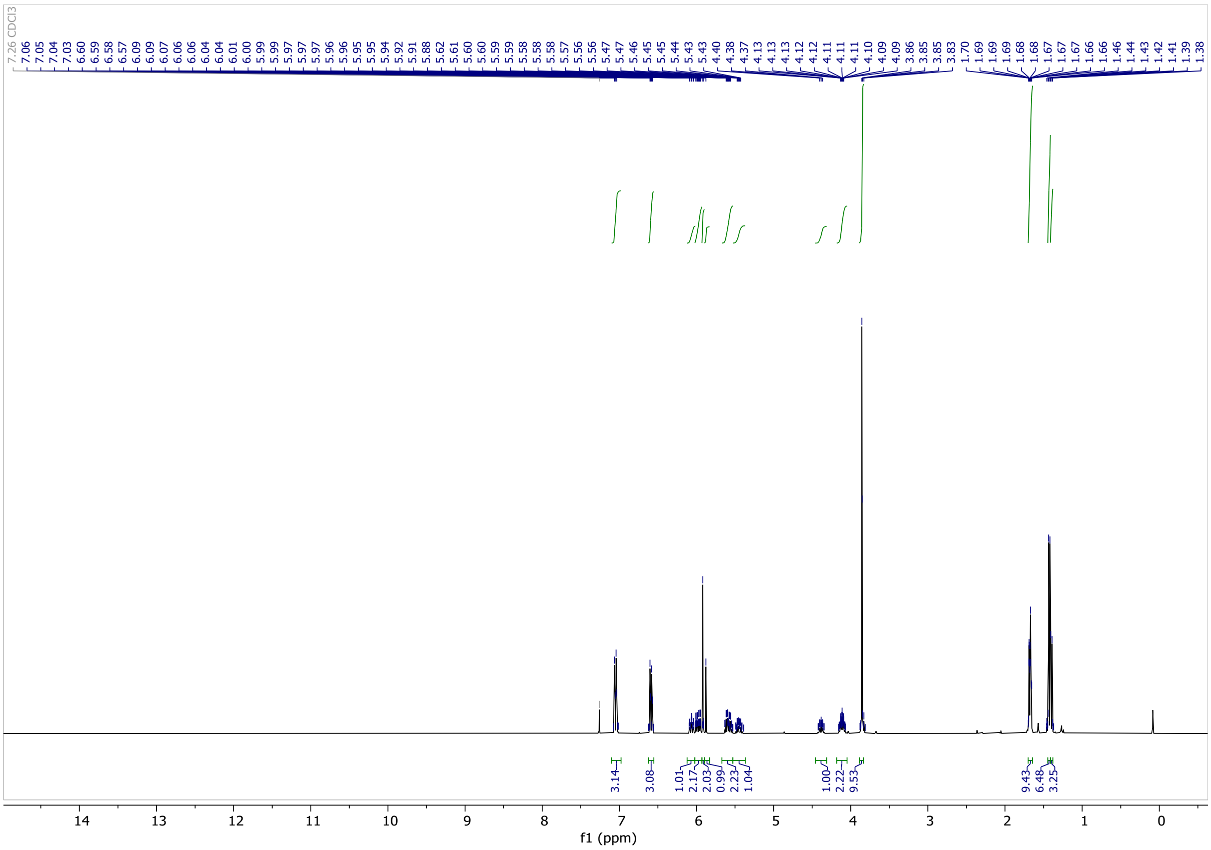

^{13}C NMR (101 MHz, $CDCl_3$)

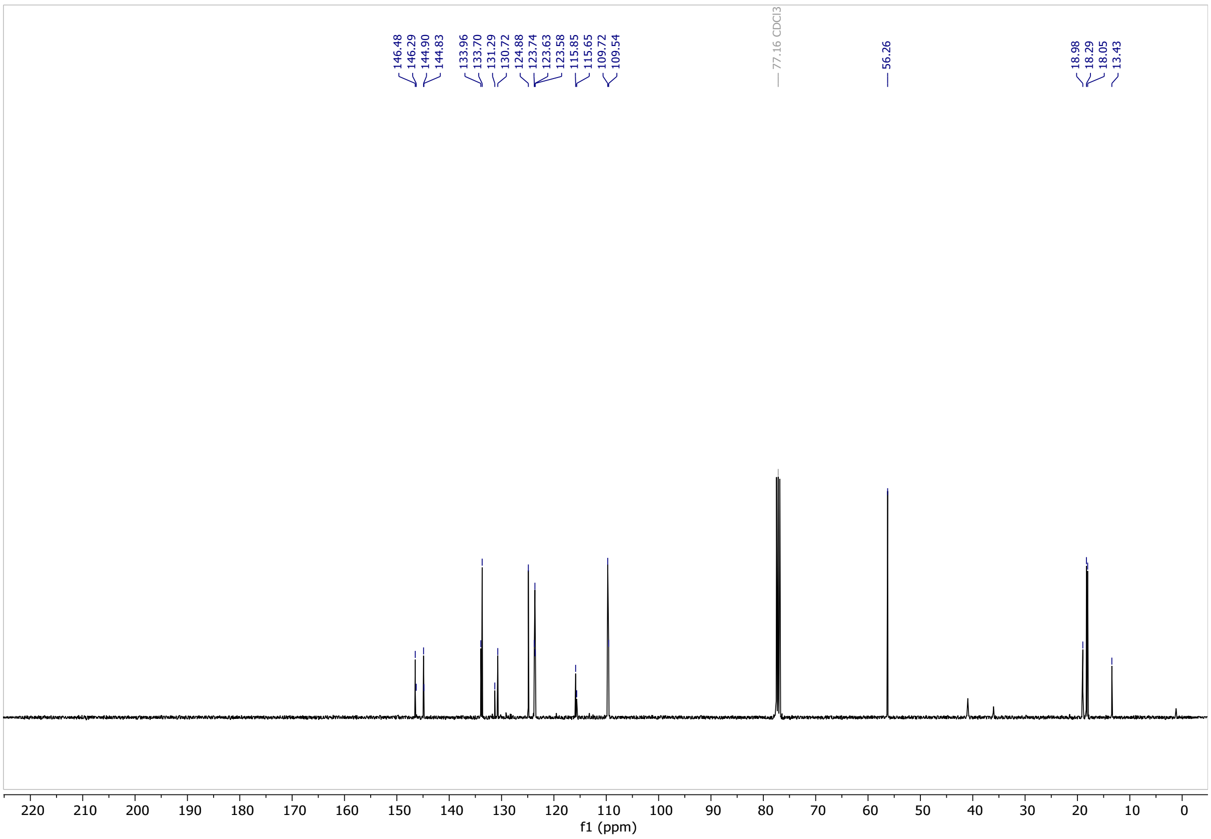

Chromatogram and Results

Instrument Method:	**Hexane_EtOH_99_1_0.7mlmin_25C_45min-MK**	*B %:*	**0,0**
Column:	**OJ3**	*C %:*	**99,0**
Run Time (min):	**45,00**	*D %:*	**1,0**
Channel:	**UV_VIS_1**		
Wavelength:	**287,26**		

Chromatogram

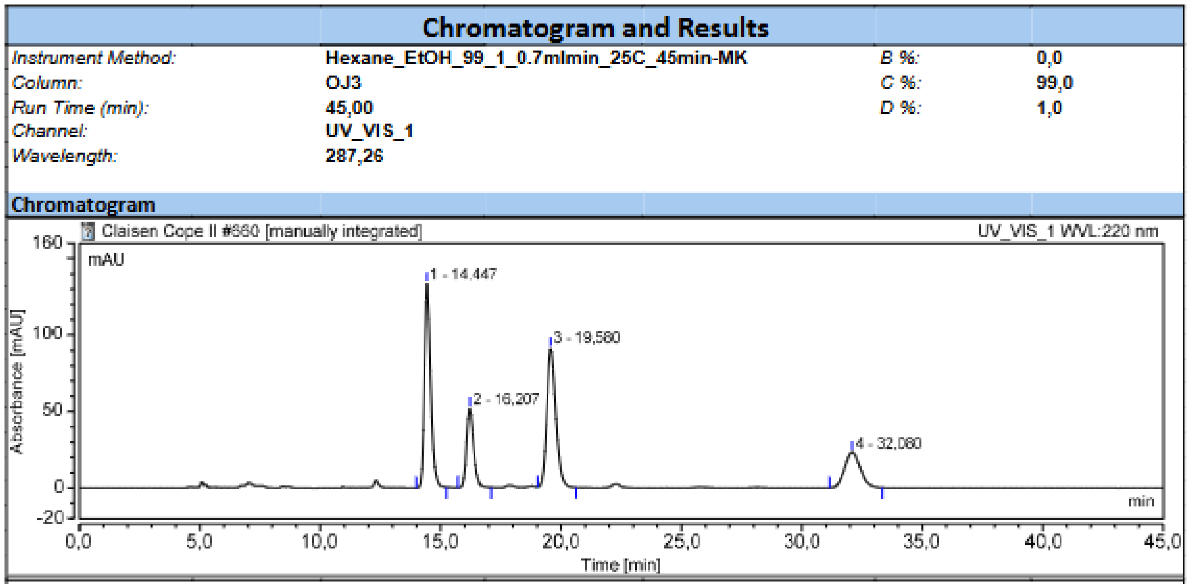

Integration Results

No.	Peak Name	Retention Time min	Area mAU*min	Height mAU	Relative Area %	Relative Height %
1		14,447	40,408	133,251	34,84	44,50
2		16,207	17,640	51,975	15,21	17,36
3		19,580	40,358	91,008	34,80	30,39
4		32,080	17,575	23,199	15,15	7,75
**Total:**			**115,982**	**299,434**	**100,00**	**100,00**

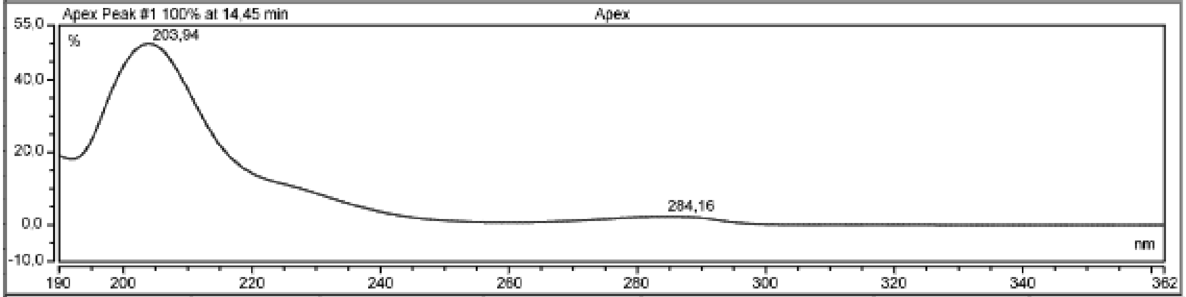

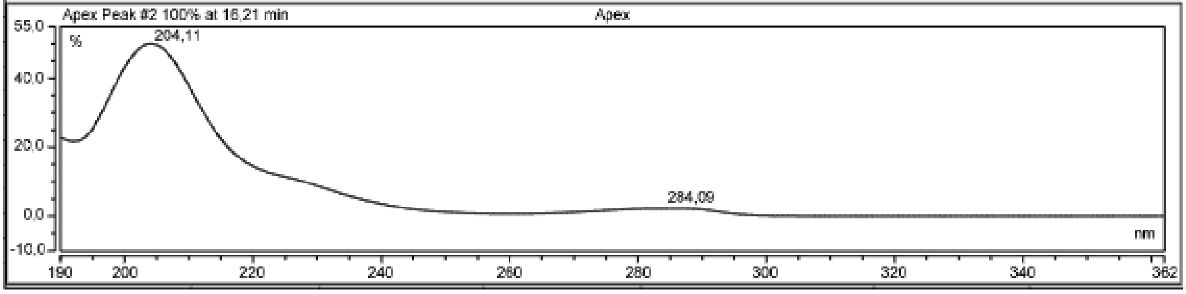

Chromatogram and Results

Instrument Method:	**Hexane_EtOH_99_1_0.7mlmin_25C_45min-MK**	*B %:*	**0,0**
Column:	**OJ3**	*C %:*	**99,0**
Run Time (min):	**45,00**	*D %:*	**1,0**
Channel:	**UV_VIS_1**		
Wavelength:	**287,26**		

Chromatogram

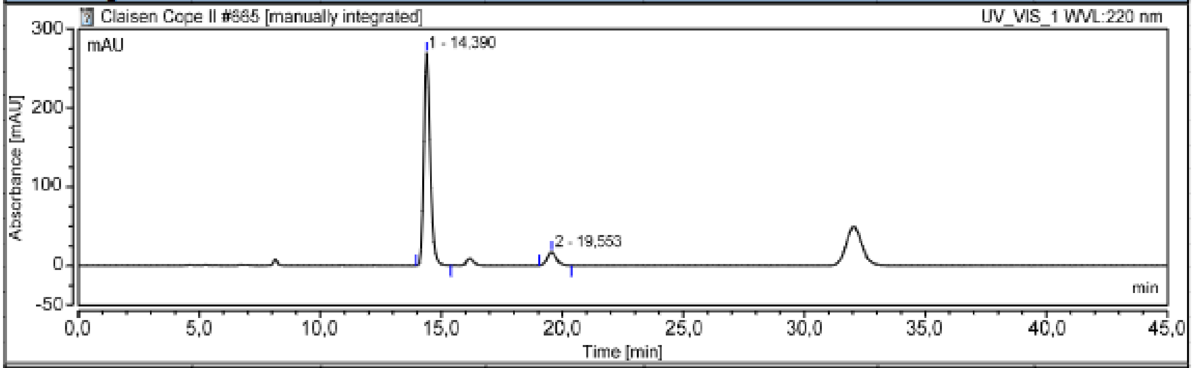

Integration Results

No.	Peak Name	Retention Time<br>min	Area<br>mAU*min	Height<br>mAU	Relative Area<br>%	Relative Height<br>%
1		14,390	82,007	269,235	91,51	93,95
2		19,553	7,606	17,352	8,49	6,05
**Total:**			**89,613**	**286,587**	**100,00**	**100,00**

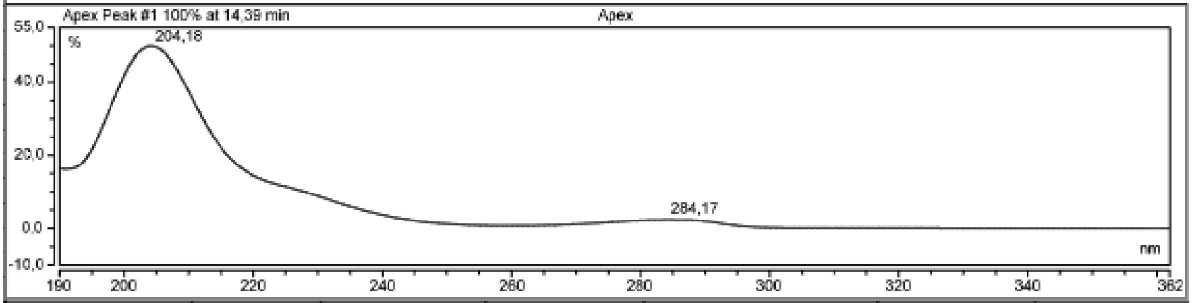

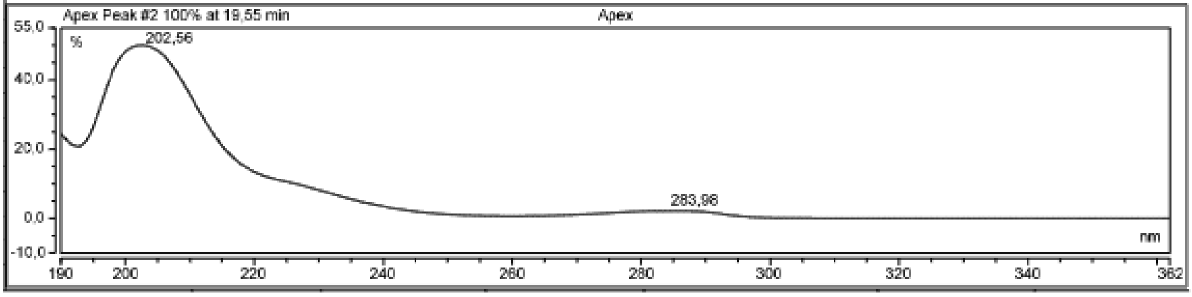

Chromatogram and Results

Instrument Method:	**Hexane_EtOH_99_1_0.7mlmin_25C_45min-MK**	*B %:*	**0,0**
Column:	**OJ3**	*C %:*	**99,0**
Run Time (min):	**45,00**	*D %:*	**1,0**
Channel:	**UV_VIS_1**		
Wavelength:	**287,26**		

Chromatogram

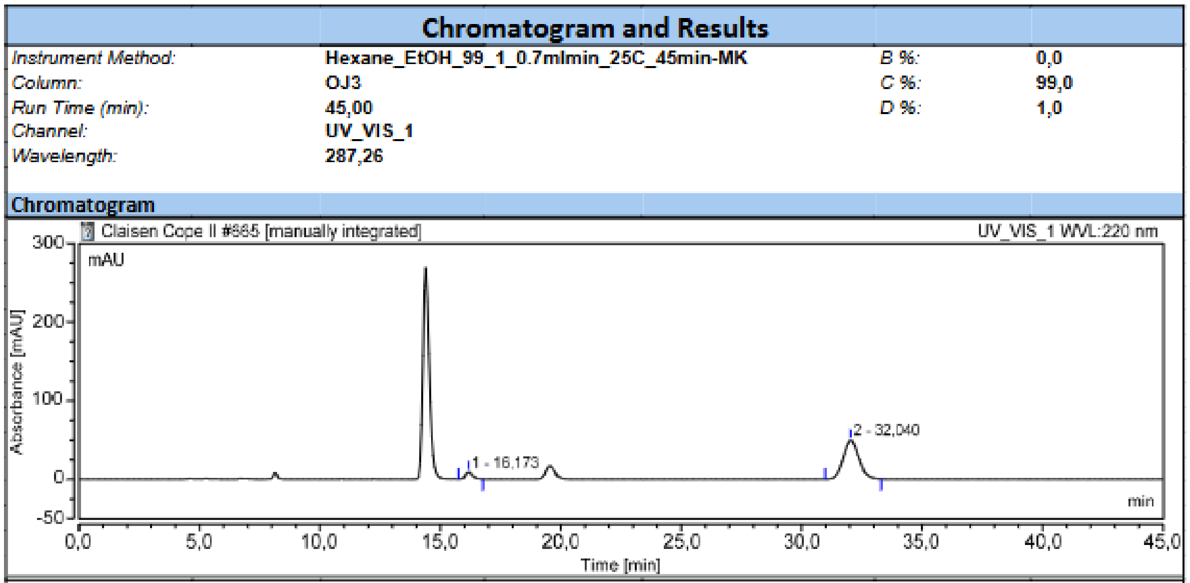

Integration Results

No.	Peak Name	Retention Time<br>min	Area<br>mAU*min	Height<br>mAU	Relative Area<br>%	Relative Height<br>%
1		16,173	3,151	9,339	7,81	15,89
2		32,040	37,201	49,451	92,19	84,11
**Total:**			**40,353**	**58,790**	**100,00**	**100,00**

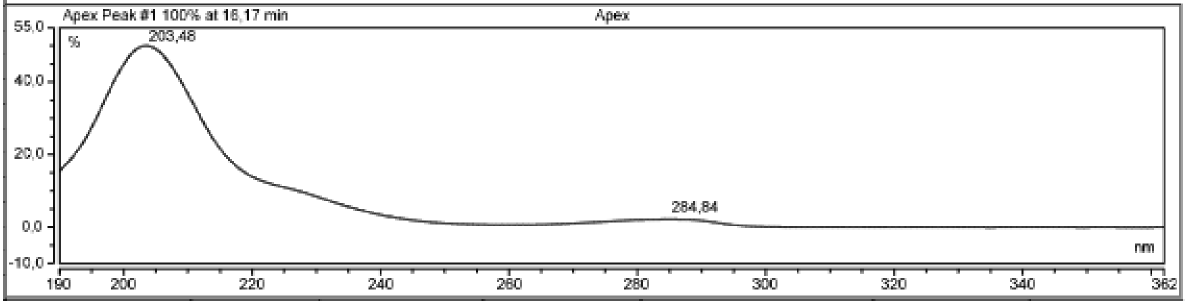

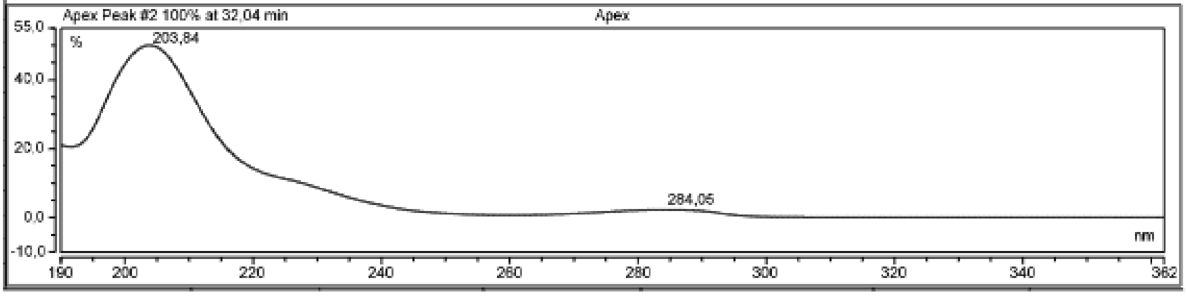

2-Ethoxy-4-((*R,E*)-pent-3-en-2-yl)-5-((*E*)-prop-1-en-1-yl)phenol (2s) & 6-ethoxy-2-((*S,E*)-pent-3-en-2-yl)-3-((*E*)-prop-1-en-1-yl)phenol (3s)

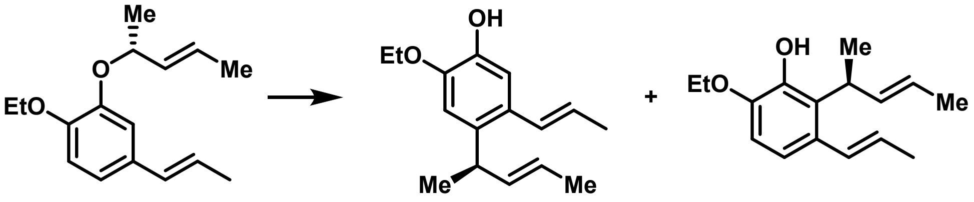

1s **2s** **3s**

The title compounds were synthesized from **1s** (50 mg, 0.20 mmol) following **general procedure B**. The reaction was directly purified by column chromatography (petroleum ether/ethyl acetate 30:1) to provide the *para*-product **2s** as colorless oil in 30% yield (15 mg, 0.06 mmol) and the *ortho*-product **3s** as colorless oil in 70% yield (35 mg, 0.14 mmol).

2-Ethoxy-4-((*R,E*)-pent-3-en-2-yl)-5-((E)-prop-1-en-1-yl)phenol (2s)

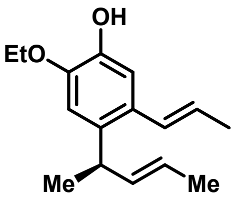

$[\alpha]^{20}$= + 24.72 (c 0.50, CH_2Cl_2).

^{1}H NMR (400 MHz, $CDCl_3$) δ 6.90 (s, 1H), 6.56 (d, *J* = 5.1 Hz, 1H), 5.88 (dq, *J* = 15.4, 6.6 Hz, 1H), 5.55 – 5.45 (m, 1H), 5.39 (s, 1H), 5.37 – 5.25 (m, 1H), 4.14 – 3.94 (m, 2H), 3.68 – 3.54 (m, 1H), 1.78 (td, *J* = 6.7, 1.7 Hz, 3H), 1.61 (dt, *J* = 6.3, 1.5 Hz, 3H), 1.36 (td, *J* = 7.0, 3.8 Hz, 3H), 1.19 (d, *J* = 7.1 Hz, 3H).

^{13}C NMR (101 MHz, $CDCl_3$) δ 145.2, 143.9, 136.1, 135.0, 129.6, 128.1, 126.2, 123.7, 112.1, 110.0, 64.6, 36.9, 21.2, 18.9, 18.1, 15.1.

HRMS (ESI): exact mass calculated for $C_{16}H_{21}O_2^-$ [(M - H)$^-$], 245.1547; found 245.1525.

79% *ee* (determined by chiral HPLC: Chiralcel® OD column, n-Hexane/EtOH = 99.5:0.5, 0.7 mL/min, λ = 287.3 nm, 25 °C), major enantiomer. t_r = 11.50 min, minor enantiomer. t_r = 9.73 min.

^1H NMR (400 MHz, $CDCl_3$)

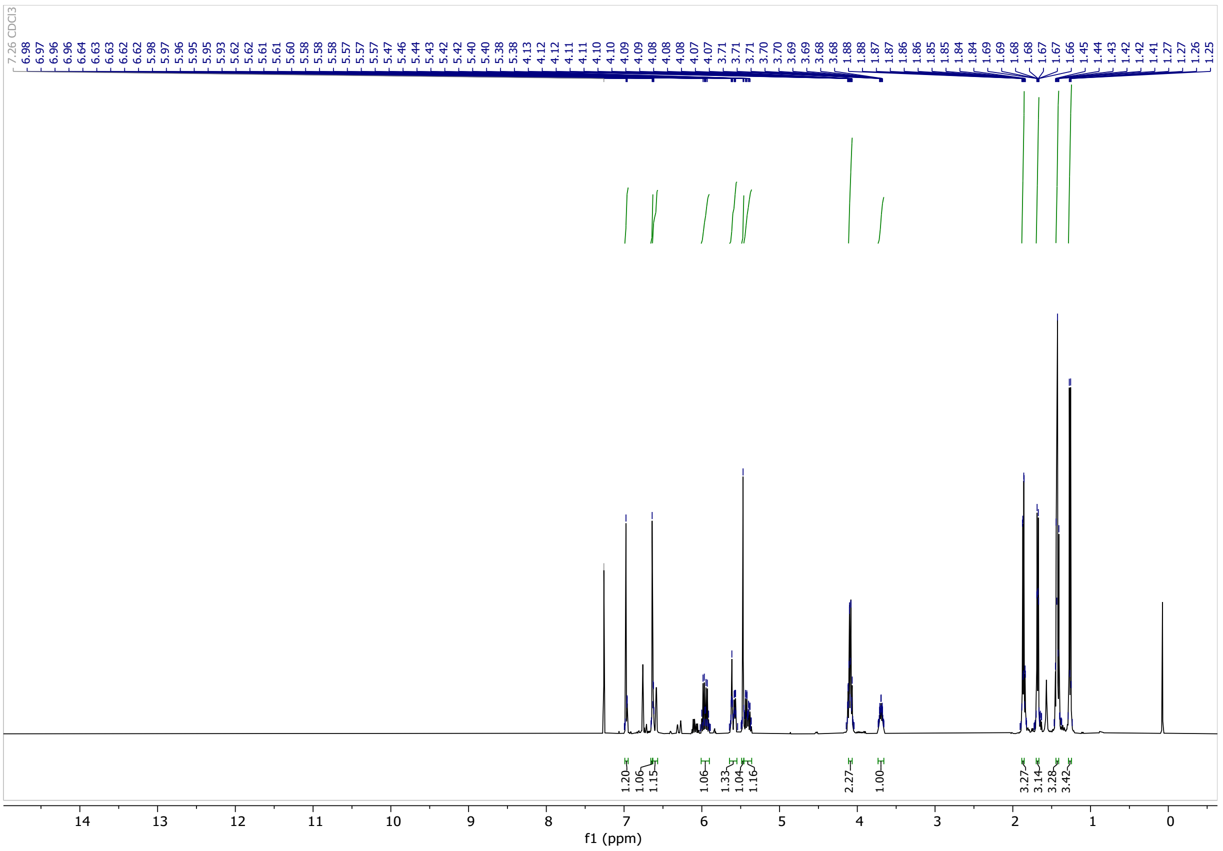

^{13}C NMR (101 MHz, $CDCl_3$)

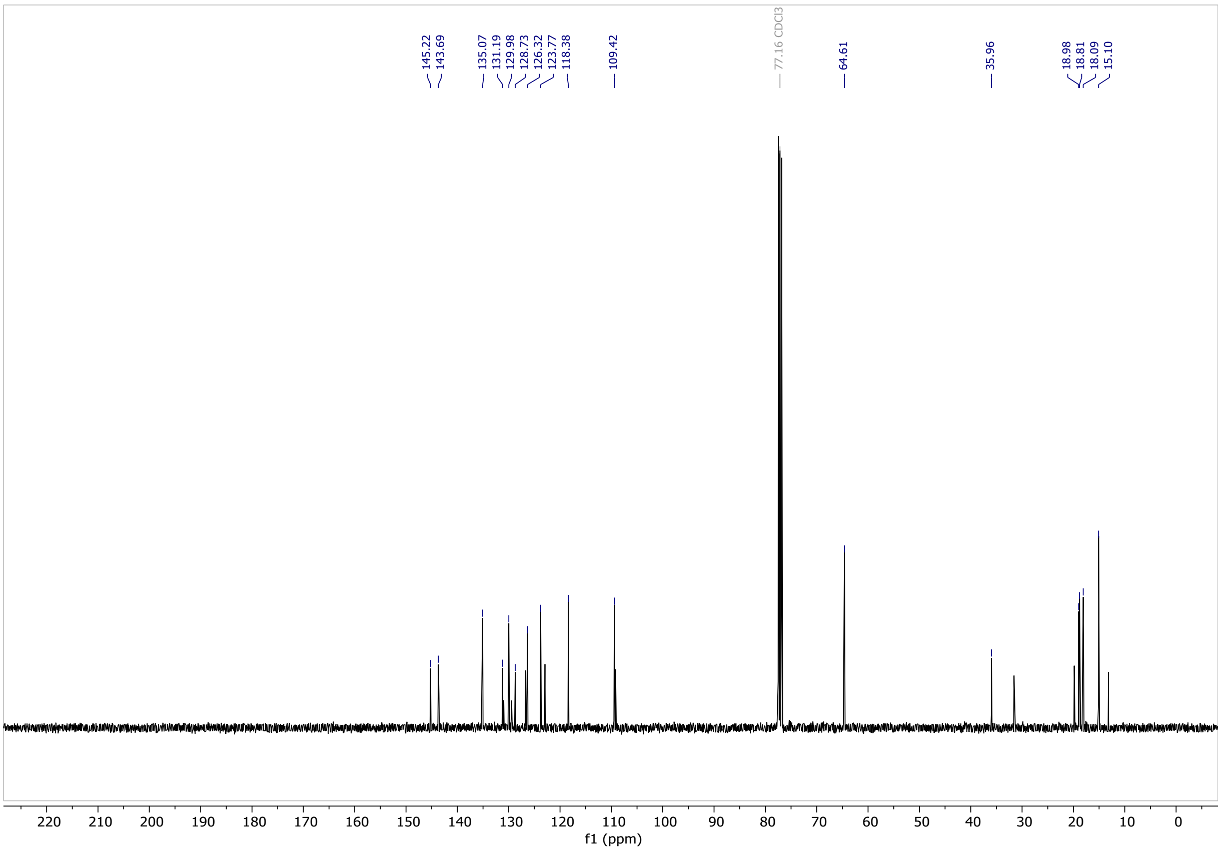

145.22
143.69
135.07
131.19
129.98
128.73
126.32
123.77
118.38
109.42
77.16 CDCl3
64.61
35.96
18.98
18.81
18.09
15.10
220 210 200 190 180 170 160 150 140 130 120 110 100 90 80 70 60 50 40 30 20 10 0
f1 (ppm)

Chromatogram and Results

Instrument Method:	**Hexane_EtOH_99.5_0.5_0.7mlmin_25C_20min-MK**	*B %:*	**0,0**
Column:	**OD**	*C %:*	**99,5**
Run Time (min):	**20,00**	*D %:*	**0,5**
Channel:	**UV_VIS_1**		
Wavelength:	**287,26**		

Chromatogram

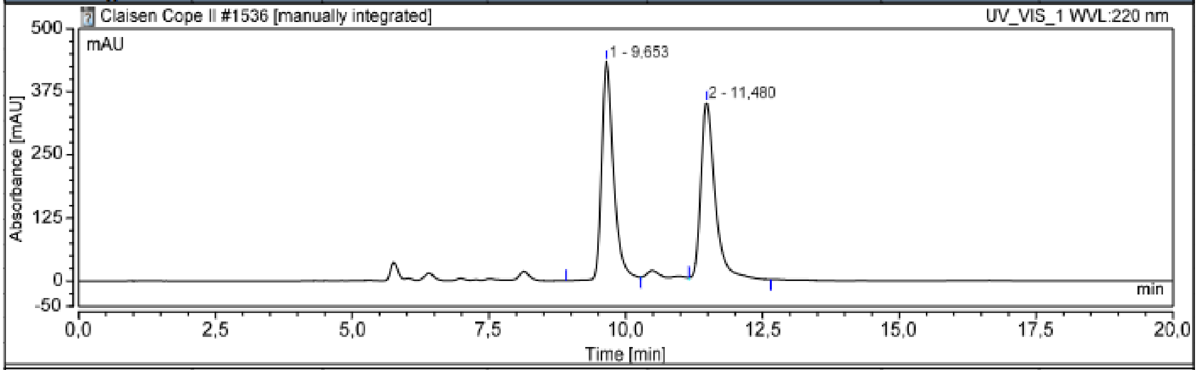

Integration Results

No.	Peak Name	Retention Time min	Area mAU*min	Height mAU	Relative Area %	Relative Height %
1		9,653	110,100	434,025	50,71	55,21
2		11,480	107,035	352,104	49,29	44,79
**Total:**			**217,135**	**786,129**	**100,00**	**100,00**

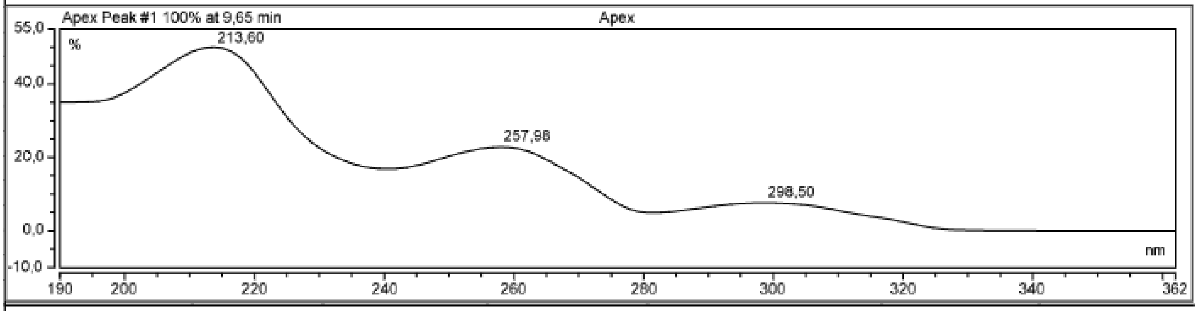

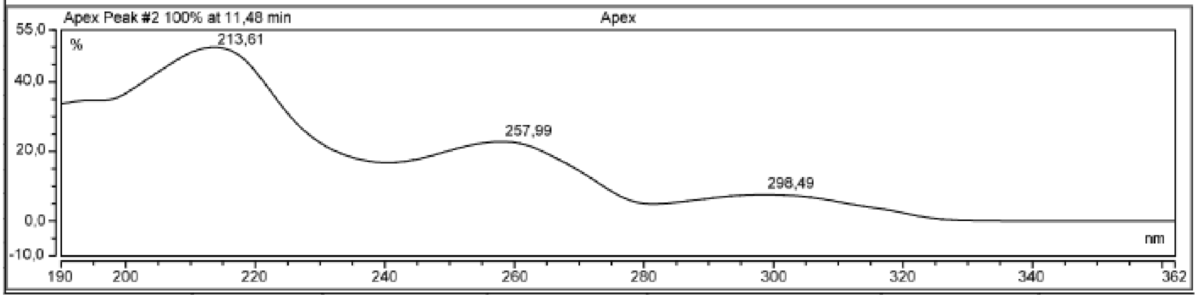

Chromatogram and Results

Instrument Method:	**Hexane_EtOH_99.5_0.5_0.7mlmin_25C_20min-MK**	*B %:*	**0,0**
Column:	**OD**	*C %:*	**99,5**
Run Time (min):	**20,00**	*D %:*	**0,5**
Channel:	**UV_VIS_1**		
Wavelength:	**287,26**		

Chromatogram

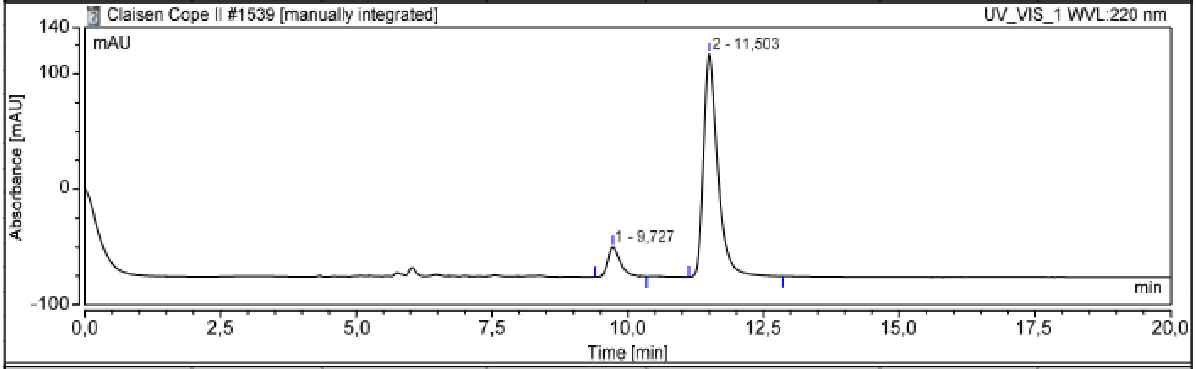

Integration Results

No.	Peak Name	Retention Time<br>min	Area<br>mAU*min	Height<br>mAU	Relative Area<br>%	Relative Height<br>%
1		9,727	7,036	25,993	10,64	11,86
2		11,503	59,116	193,225	89,36	88,14
**Total:**			**66,151**	**219,218**	**100,00**	**100,00**

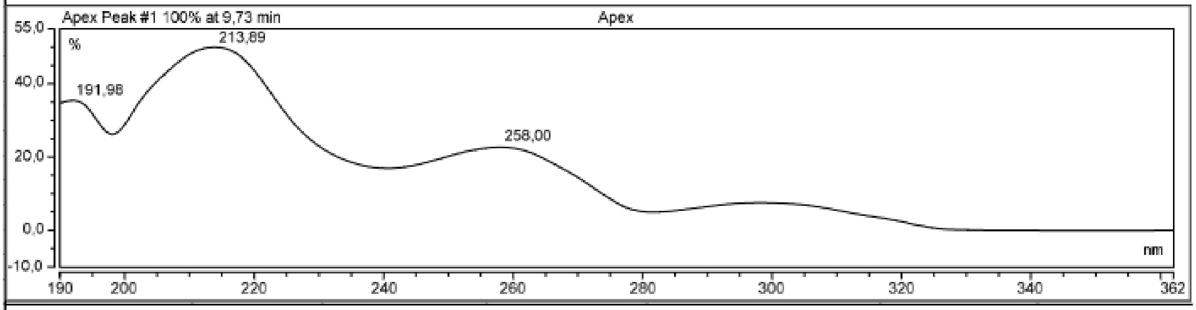

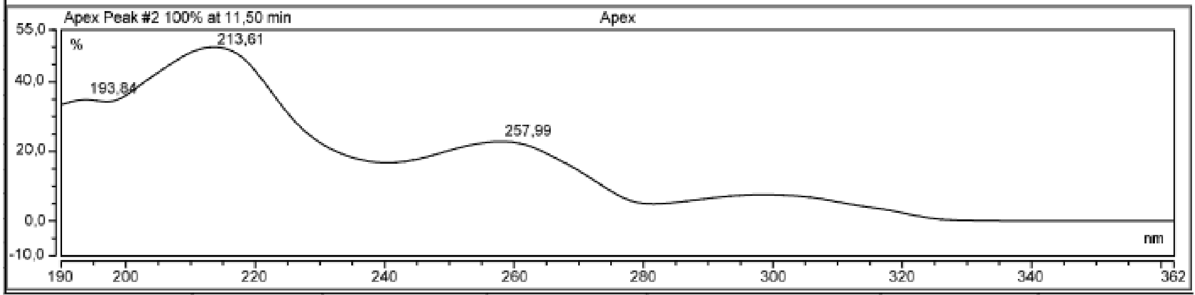

6-Ethoxy-2-((*S,E*)-pent-3-en-2-yl)-3-((*E*)-prop-1-en-1-yl)phenol (3s)

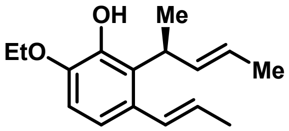

[α][20]= - 44.01 (c 1.20, CH_2Cl_2).

Compound **3s** was obtained as 1.9:1.0 *E/Z* mixture as measured by the ratio of the major (*E*)-isomer δ 3.94 (tt, *J* = 7.5, 5.8 Hz, 1H, integral= 1.92) to the minor (*Z*)-isomer δ 4.20 (p, *J* = 7.5 Hz, 1H, integral= 1.00); ^{1}H NMR (400 MHz, $CDCl_3$) δ 6.86 – 6.78 (m, 2.88H), 6.76 – 6.59 (m, 5.76H), 6.02 (ddq, *J* = 10.4, 8.5, 1.8 Hz, 1.00H), 5.97 – 5.85 (m, 3.95H), 5.83 (s, 1.95H), 5.83 (s, 1.06H), 5.53 – 5.44 (m, 1.72H), 5.40 (dqd, *J* = 10.7, 6.6, 1.5 Hz, 1.05H), 4.20 (p, *J* = 7.5 Hz, 1.0H), 4.12 – 4.04 (m, 5.96H), 3.94 (tt, *J* = 7.5, 5.8 Hz, 1.92H), 1.87 (ddd, *J* = 8.4, 6.6, 1.7 Hz, 8.66H), 1.67 (dt, *J* = 6.4, 1.6 Hz, 5.92H), 1.58 (dd, *J* = 6.8, 1.9 Hz, 4.26H), 1.48 – 1.34 (m, 17.91H).

^{13}C NMR (101 MHz, $CDCl_3$) δ 145.2, 145.1, 143.7, 143.6, 135.2, 135.1, 131.2, 131.0, 130.0, 130.0, 129.4, 128.7, 126.7, 126.3, 123.8, 122.9, 118.4, 118.4, 109.4, 109.2, 64.6, 36.0, 31.6, 19.8, 19.0, 18.8, 18.8, 18.1, 15.1, 13.2.

(*E*)-isomer: 84% *ee* (determined by chiral HPLC: Chiralcel® OJ-3 column, n-Hexane/EtOH = 99.9:0.1, 0.2 mL/min, λ = 287.3 nm, 25 °C), major enantiomer. t_r = 36.85 min, minor enantiomer. t_r = 31.60 min.

HRMS (ESI): exact mass calculated for $C_{16}H_{21}O_2^-$ [(M - H)$^-$], 245.1547; found 245.1525.

(*Z*)-isomer: 84% *ee* (determined by chiral HPLC: Chiralcel® OJ-3 column, n-Hexane/EtOH = 99.9:0.1, 0.2 mL/min, λ = 287.3 nm, 25 °C), major enantiomer. t_r = 33.94 min, minor enantiomer. t_r = 32.13 min.

^1H NMR (400 MHz, $CDCl_3$)

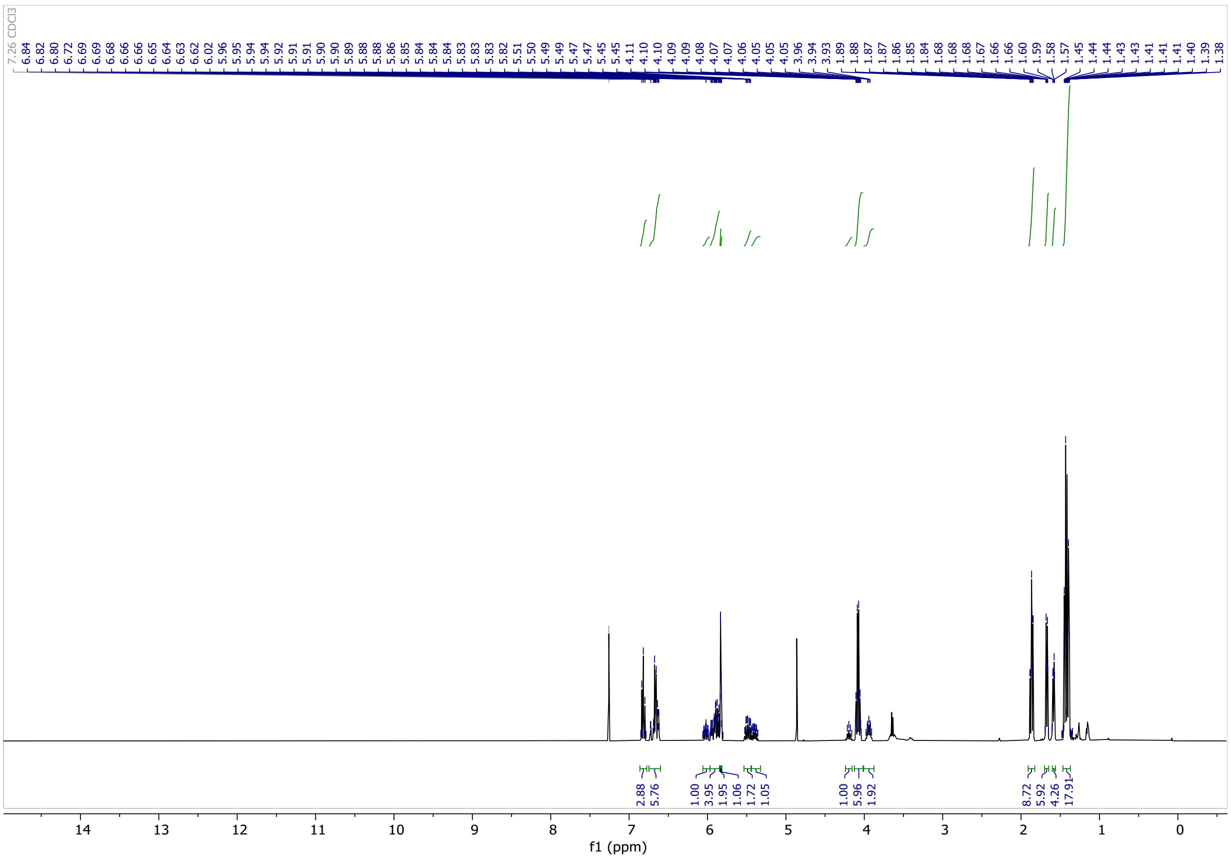

^{13}C NMR (101 MHz, $CDCl_3$)

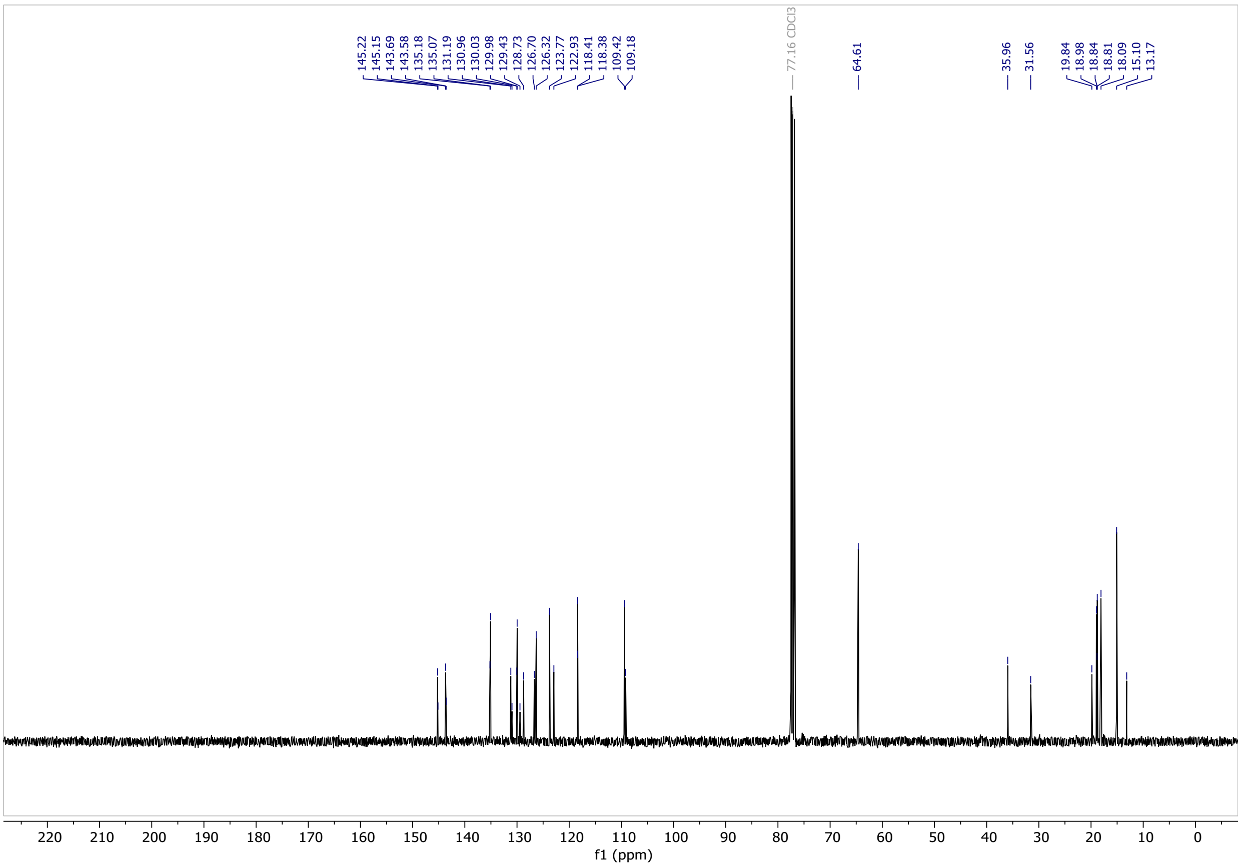

145.22
145.15
143.69
143.58
135.18
135.07
131.19
130.96
130.03
129.98
129.43
128.73
126.70
126.32
123.77
122.93
118.41
118.38
109.42
109.18
77.16 CDCl3
64.61
35.96
31.56
19.84
18.98
18.84
18.81
18.09
15.10
13.17
220 210 200 190 180 170 160 150 140 130 120 110 100 90 80 70 60 50 40 30 20 10 0
f1 (ppm)

Chromatogram and Results

Instrument Method:	**Hexane_EtOH_99.9_0.1_0.2mlmin_25C_40min-MK**	*B %:*	**0,0**
Column:	**OJ3**	*C %:*	**99,9**
Run Time (min):	**40,00**	*D %:*	**0,1**
Channel:	**UV_VIS_1**		
Wavelength:	**287,26**		

Chromatogram

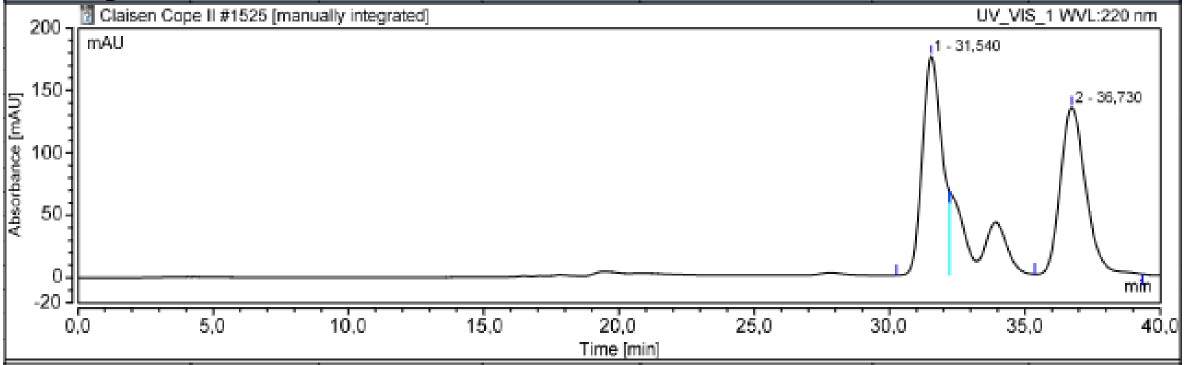

Integration Results

No.	Peak Name	Retention Time min	Area mAU*min	Height mAU	Relative Area %	Relative Height %
1		31,540	146,753	175,570	50,25	56,74
2		36,730	145,300	133,881	49,75	43,26
**Total:**			**292,053**	**309,452**	**100,00**	**100,00**

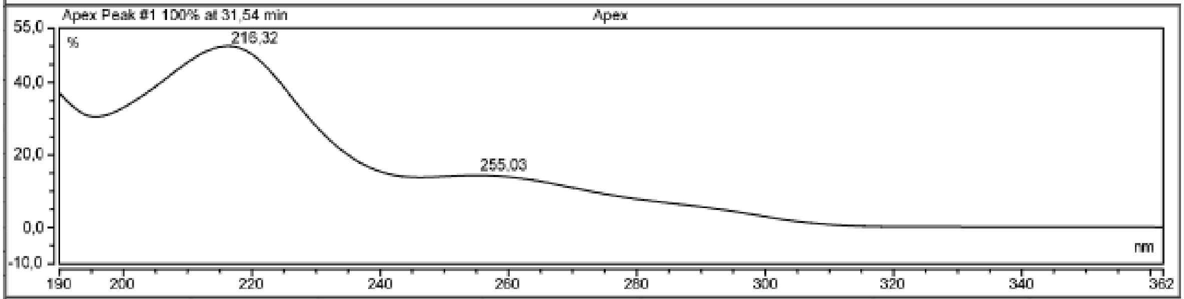

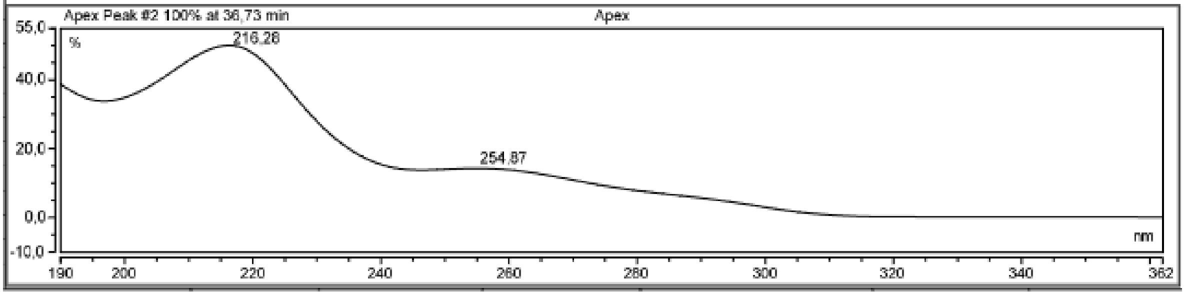

Chromatogram and Results

Instrument Method:	**Hexane_EtOH_99.9_0.1_0.2mlmin_25C_40min-MK**	*B %:*	**0,0**
Column:	**OJ3**	*C %:*	**99,9**
Run Time (min):	**40,00**	*D %:*	**0,1**
Channel:	**UV_VIS_1**		
Wavelength:	**287,26**		

Chromatogram

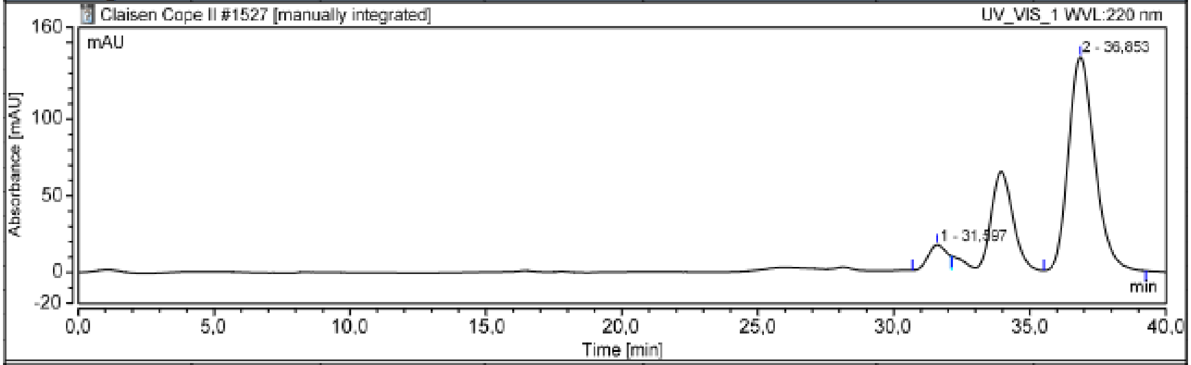

Integration Results

No.	Peak Name	Retention Time min	Area mAU*min	Height mAU	Relative Area %	Relative Height %
1		31,597	13,217	16,543	7,81	10,62
2		36,853	155,919	139,292	92,19	89,38
**Total:**			**169,136**	**155,835**	**100,00**	**100,00**

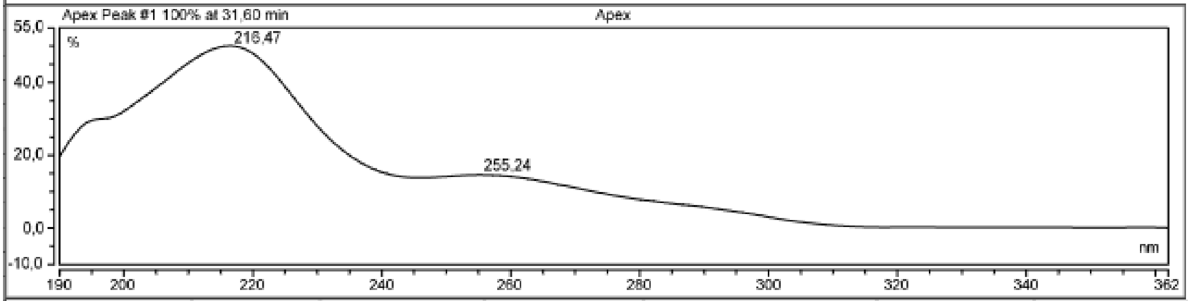

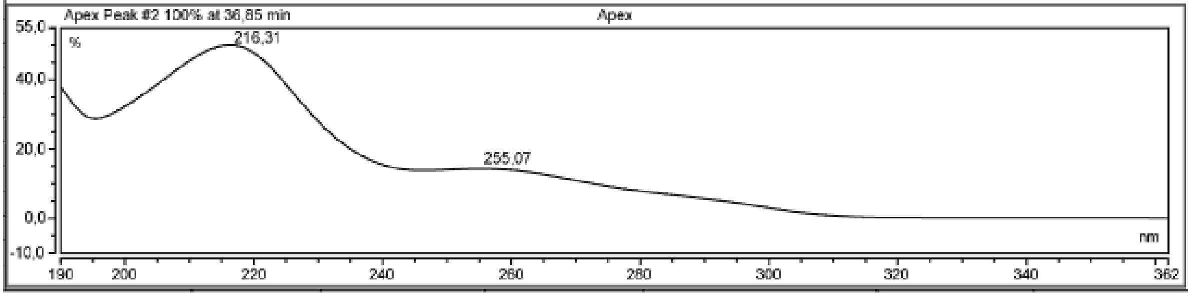

Chromatogram and Results

Instrument Method:	**Hexane_EtOH_99.9_0.1_0.2mlmin_25C_40min-MK**	*B %:*	**0,0**
Column:	**OJ3**	*C %:*	**99,9**
Run Time (min):	**40,00**	*D %:*	**0,1**
Channel:	**UV_VIS_1**		
Wavelength:	**287,26**		

Chromatogram

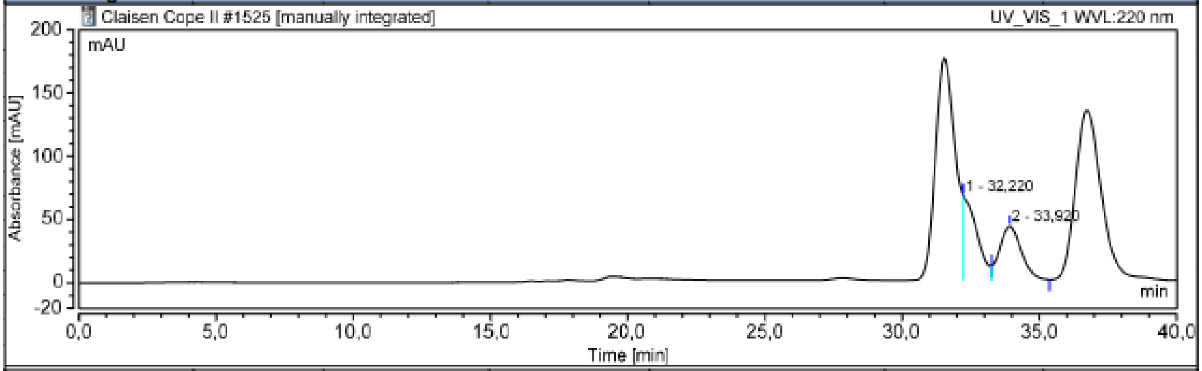

Integration Results

No.	Peak Name	Retention Time<br>min	Area<br>mAU*min	Height<br>mAU	Relative Area<br>%	Relative Height<br>%
1		32,220	38,299	66,894	49,65	61,21
2		33,920	38,839	42,388	50,35	38,79
**Total:**			**77,138**	**109,282**	**100,00**	**100,00**

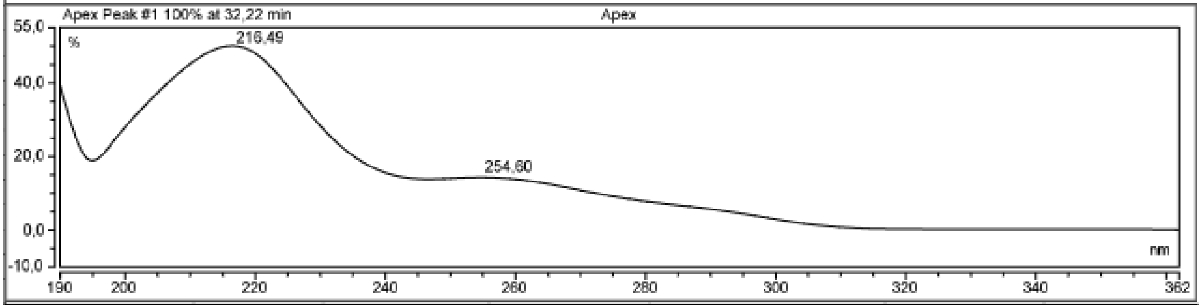

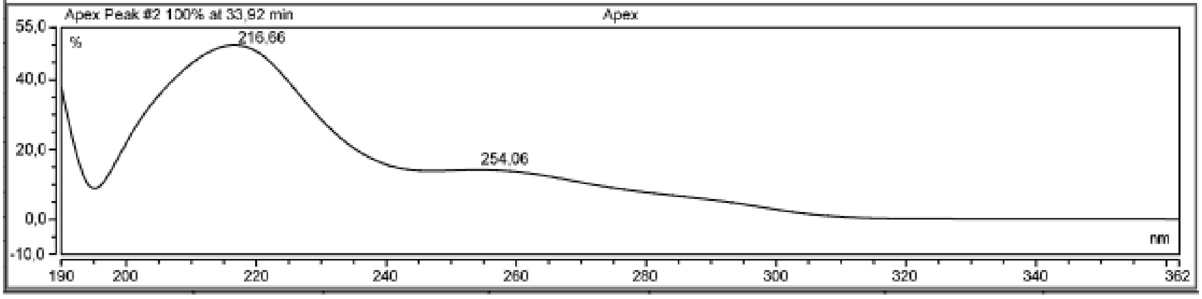

Chromatogram and Results

Instrument Method:	**Hexane_EtOH_99.9_0.1_0.2mlmin_25C_40min-MK**	*B %:*	**0,0**
Column:	**OJ3**	*C %:*	**99,9**
Run Time (min):	**40,00**	*D %:*	**0,1**
Channel:	**UV_VIS_1**		
Wavelength:	**287,26**		

Chromatogram

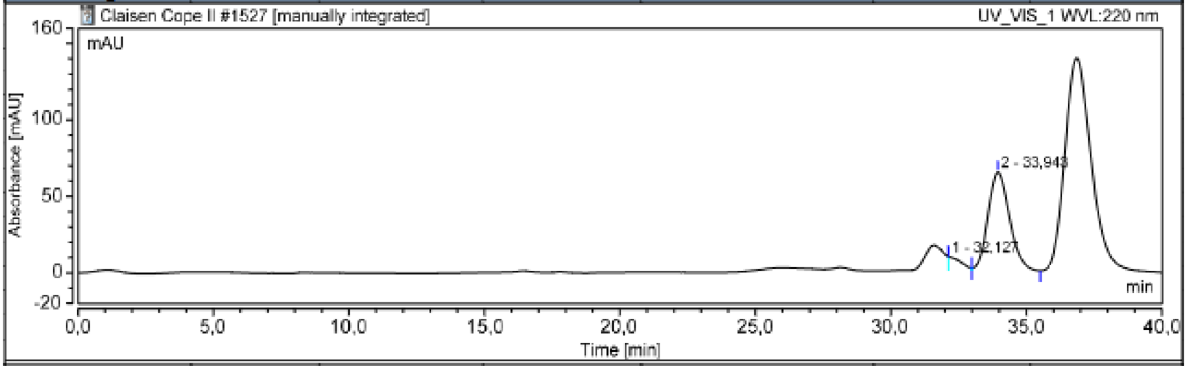

Integration Results

No.	Peak Name	Retention Time<br>min	Area<br>mAU*min	Height<br>mAU	Relative Area<br>%	Relative Height<br>%
1		32,127	4,774	9,116	7,42	12,38
2		33,943	59,534	64,527	92,58	87,62
**Total:**			**64,308**	**73,643**	**100,00**	**100,00**

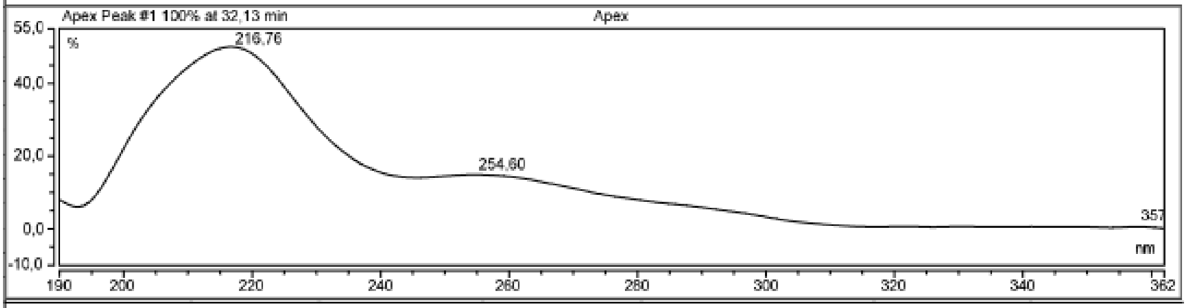

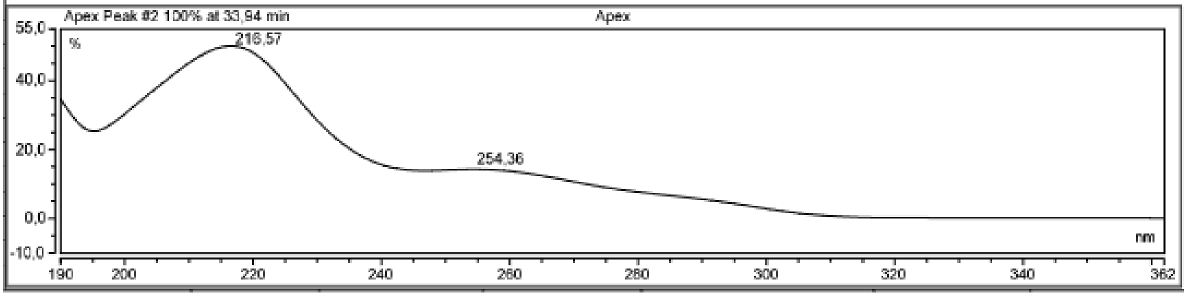

(*E*)-2,5-Dimethyl-4-(4-phenylbut-3-en-2-yl)phenol (5a)

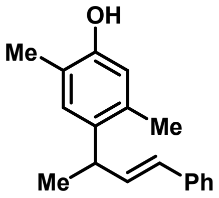

The title compound was synthesized from **4a** (100 mg, 0.40 mmol) following **general procedure B**. The reaction was directly purified by column chromatography (petroleum ether/ethyl acetate 40:1 to 5:1) to provide the product **5a** as orange oil in 92% yield (92 mg, 0.37 mmol).

^{1}H NMR (400 MHz, $CDCl_3$) δ 7.39 – 7.27 (m, 4H), 7.23 – 7.17 (m, 1H), 6.97 (s, 1H), 6.61 (s, 1H), 6.35 (d, *J* = 2.6 Hz, 2H), 4.63 (s, 1H), 3.76 (qt, *J* = 7.0, 2.4 Hz, 1H), 2.30 (s, 3H), 2.23 (s, 3H), 1.42 (d, *J* = 7.0 Hz, 3H).

^{13}C NMR (101 MHz, $CDCl_3$) δ 151.9, 137.9, 135.8, 135.4, 134.6, 129.1, 128.6, 128.2, 127.1, 126.2, 121.2, 117.0, 37.5, 20.8, 19.2, 15.6.

^{1}H NMR (400 MHz, $CDCl_3$)

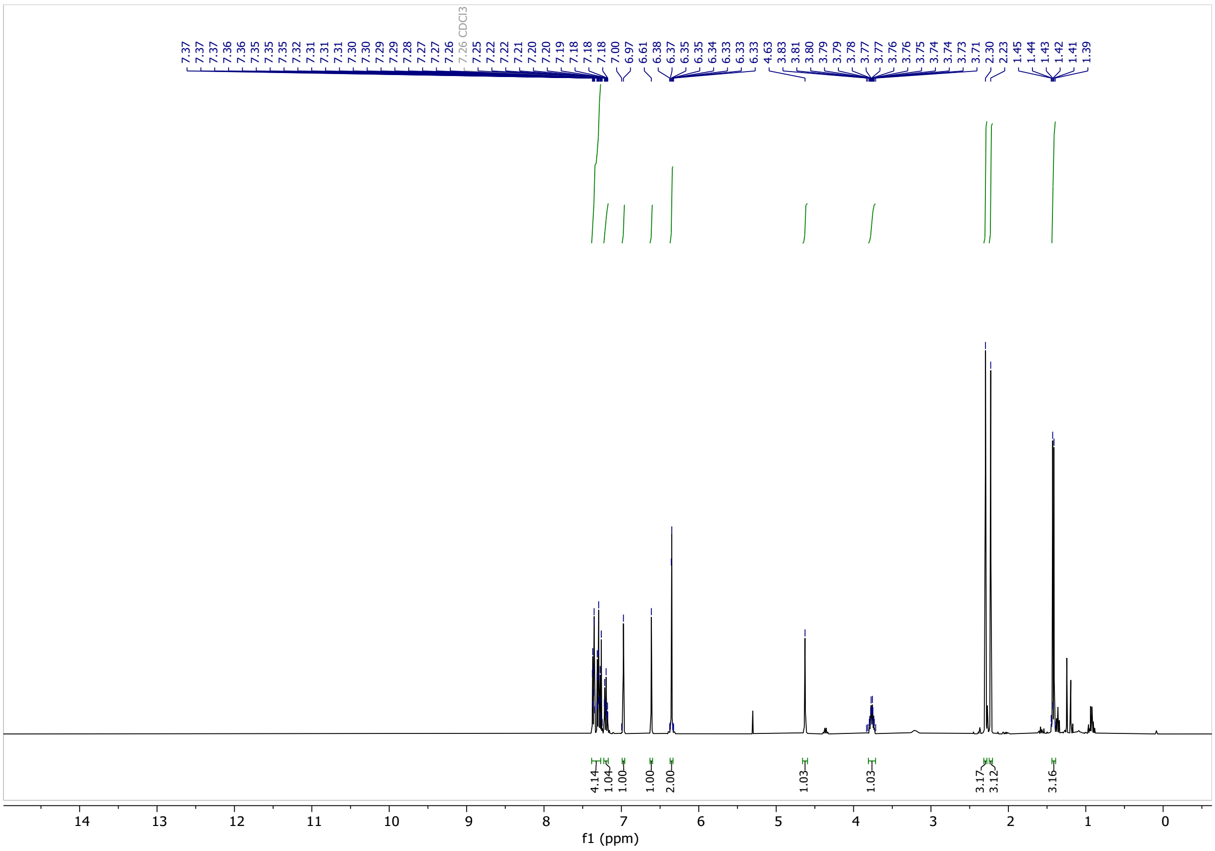

^{13}C NMR (101 MHz, $CDCl_3$)

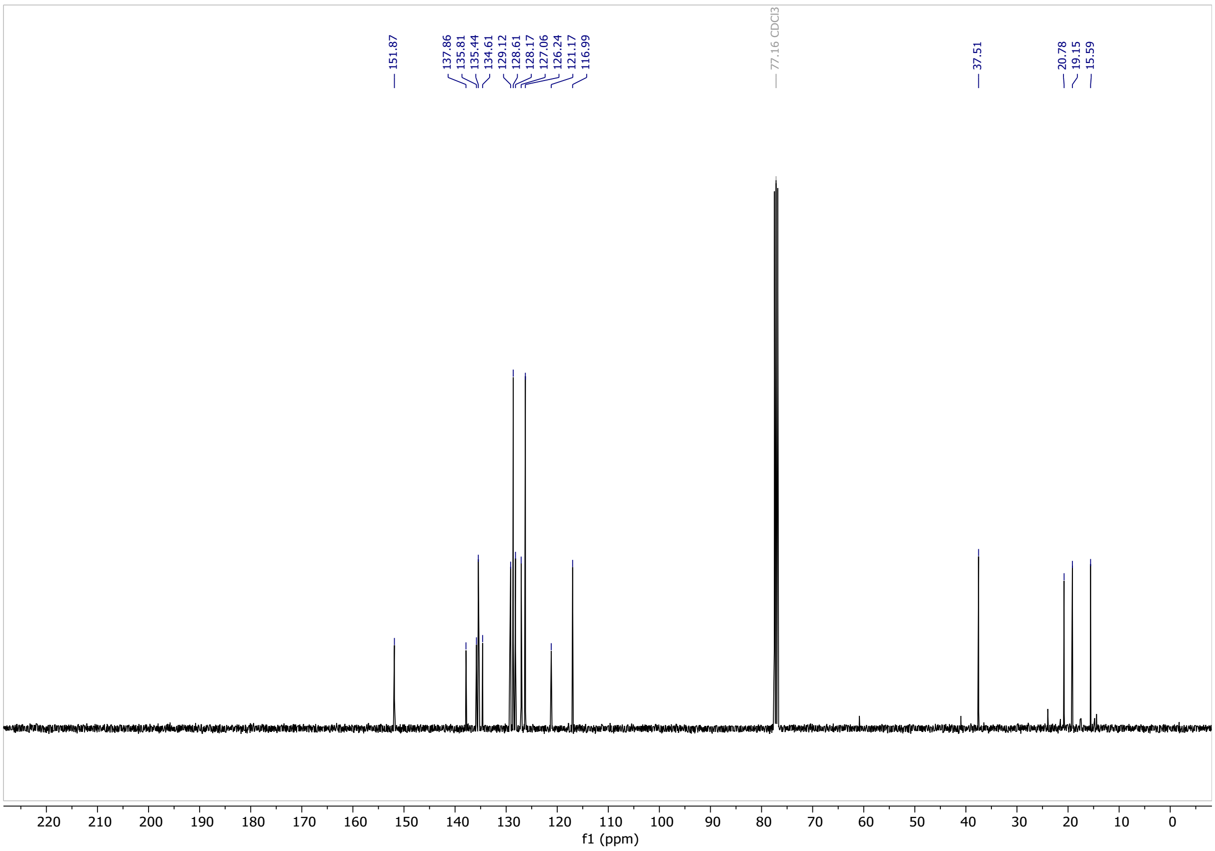

3,6-Dimethyl-1',2',3',4'-tetrahydro-[1,1'-biphenyl]-2-ol (6b)

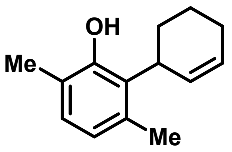

The title compound was synthesized from **4b** (96 mg, 0.48 mmol) following **general procedure B**. The reaction was directly purified by column chromatography (petroleum ether/ethyl acetate 40:1) to provide the product **6b** as colorless oil in quantitative yield (96 mg, 0.48 mmol).

^{1}H NMR (400 MHz, $CDCl_3$) δ 6.91 (d, *J* = 7.6 Hz, 1H), 6.66 (d, *J* = 7.6 Hz, 1H), 6.23 – 6.14 (m, 2H), 6.00 – 5.92 (m, 1H), 3.79 – 3.69 (m, 1H), 2.30 (s, 3H), 2.25 – 2.15 (m, 5H), 2.01 – 1.90 (m, 2H), 1.82 – 1.64 (m, 2H).

^{13}C NMR (101 MHz, $CDCl_3$) δ 153.7, 134.2, 132.8, 130.5, 128.7, 127.7, 123.8, 121.9, 36.2, 27.9, 25.0, 22.5, 20.2, 16.1.

^{1}H NMR (400 MHz, $CDCl_3$)

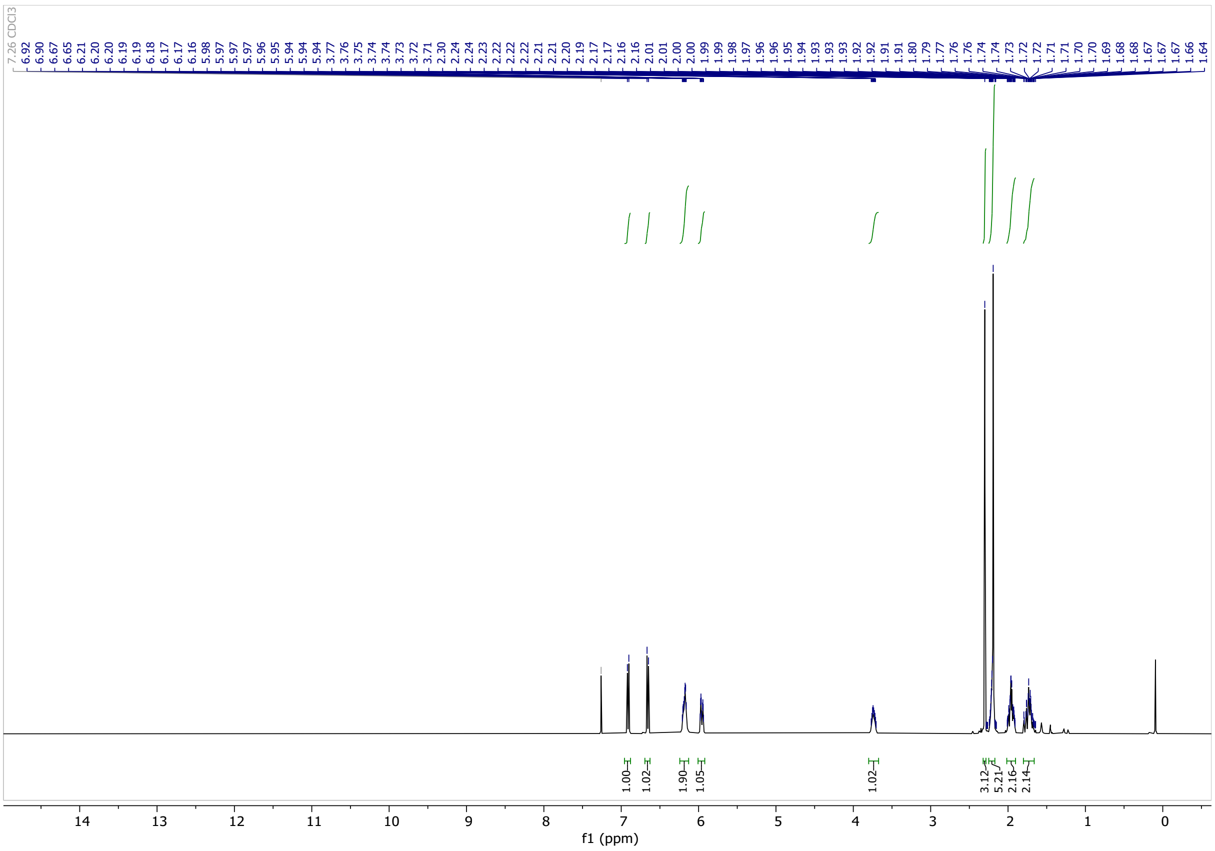

^{13}C NMR (101 MHz, $CDCl_3$)

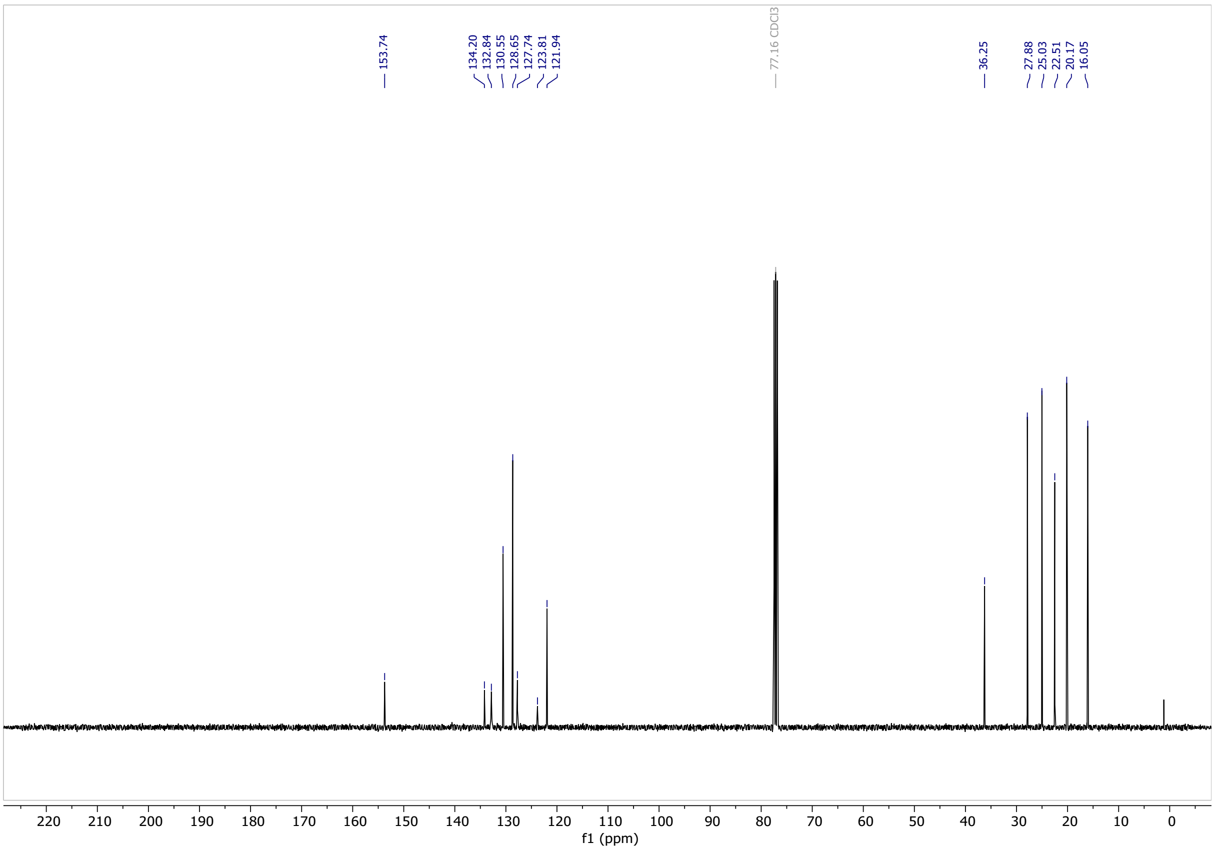

4-Allyl-2,5-dimethylphenol (5c) & 2-Allyl-3,6-dimethylphenol (6c)

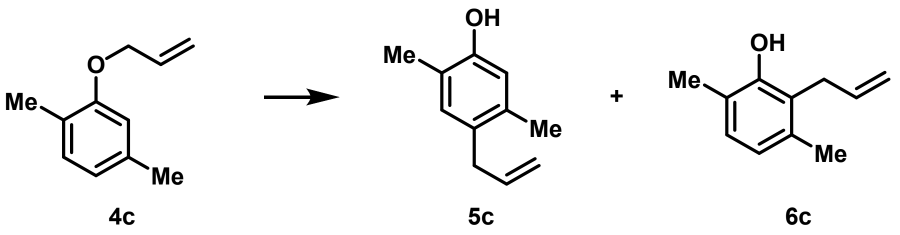

The title compounds were synthesized from **4c** (60 mg, 0.38 mmol) following modified **general procedure B at 100 °C**. The reaction was directly purified by column chromatography (petroleum ether/ethyl acetate 30:1) to provide the *para*-product **5c** as yellow oil in 33 % yield (20 mg, 0.12 mmol) and the *ortho*-product **6c** as pale-yellow oil in 52 % yield (31 mg, 0.19 mmol).

4-Allyl-2,5-dimethylphenol (5c)

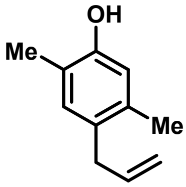

^{1}H NMR (400 MHz, $CDCl_3$) δ 6.88 (s, 1H), 6.61 (s, 1H), 5.99 – 5.85 (m, 1H), 5.06 – 4.92 (m, 2H), 3.27 (dt, J = 6.3, 1.7 Hz, 2H), 2.22 (s, 3H), 2.20 (s, 3H).

^{13}C NMR (101 MHz, $CDCl_3$) δ 152.2, 137.3, 135.3, 131.9, 130.3, 121.0, 116.9, 115.3, 37.0, 19.0, 15.4.

^1H NMR (400 MHz, $CDCl_3$)

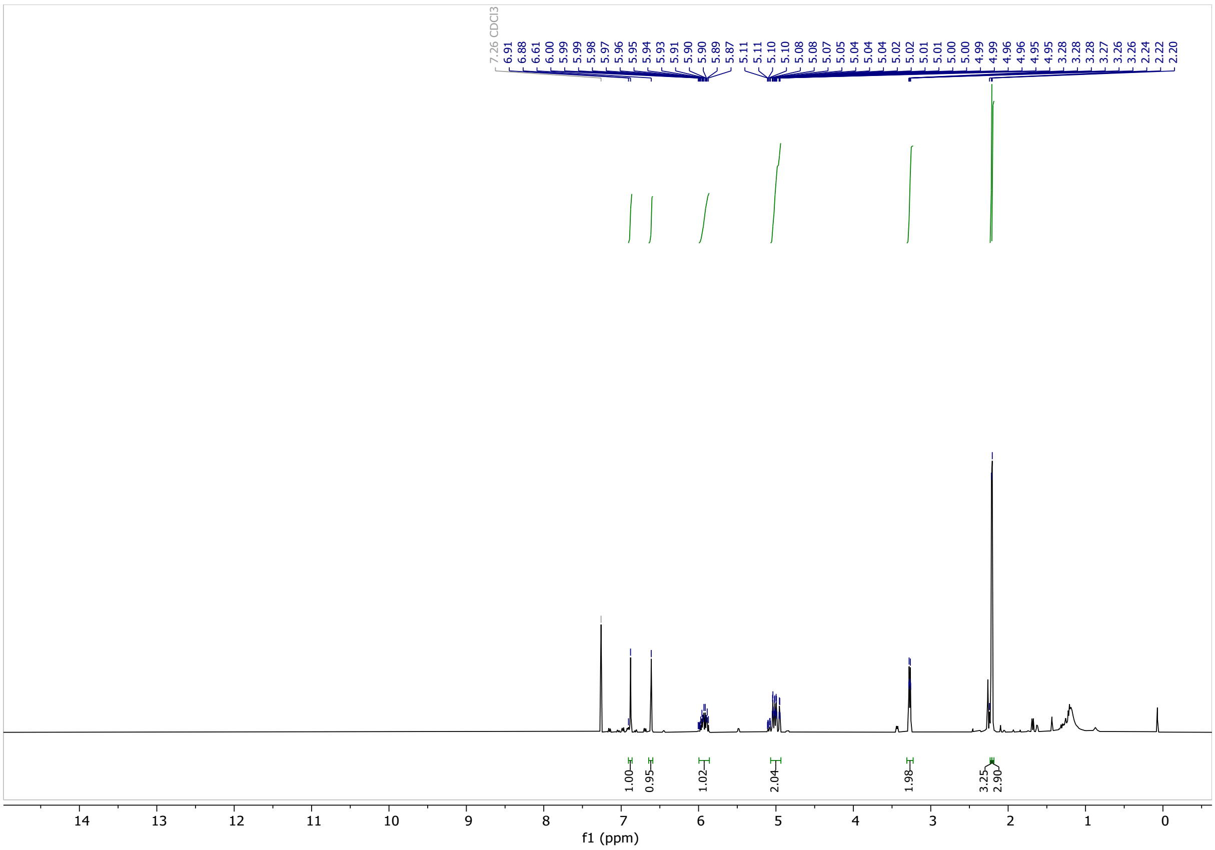

^{13}C NMR (101 MHz, $CDCl_3$)

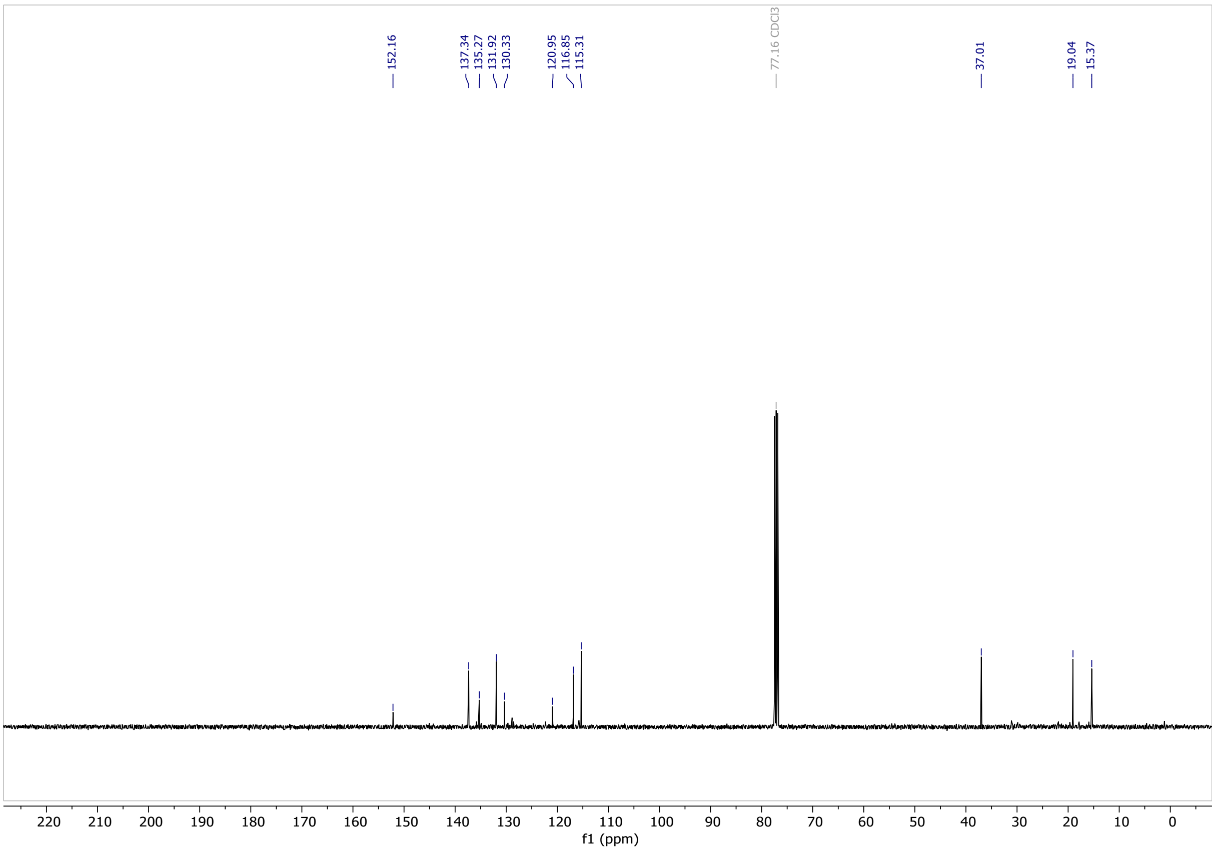

2-Allyl-3,6-dimethylphenol (6c)

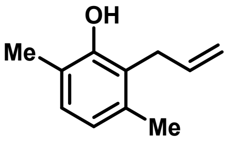

^{1}H NMR (400 MHz, $CDCl_3$) δ 6.92 (dd, *J* = 7.6, 4.9 Hz, 1H), 6.70 (t, *J* = 6.4 Hz, 1H), 5.98 (ddq, *J* = 17.3, 10.1, 5.8 Hz, 1H), 5.08 (ttd, *J* = 15.2, 3.7, 1.8 Hz, 2H), 4.87 – 4.79 (m, 1H), 3.45 (ddt, *J* = 5.9, 4.0, 1.8 Hz, 2H), 2.27 (d, *J* = 5.4 Hz, 3H), 2.23 (d, *J* = 4.7 Hz, 3H).

^{13}C NMR (101 MHz, $CDCl_3$) δ 152.5, 135.8, 135.5, 128.5, 123.3, 122.3, 121.4, 115.7, 31.1, 19.6, 15.9.

^{1}H NMR (400 MHz, $CDCl_3$)

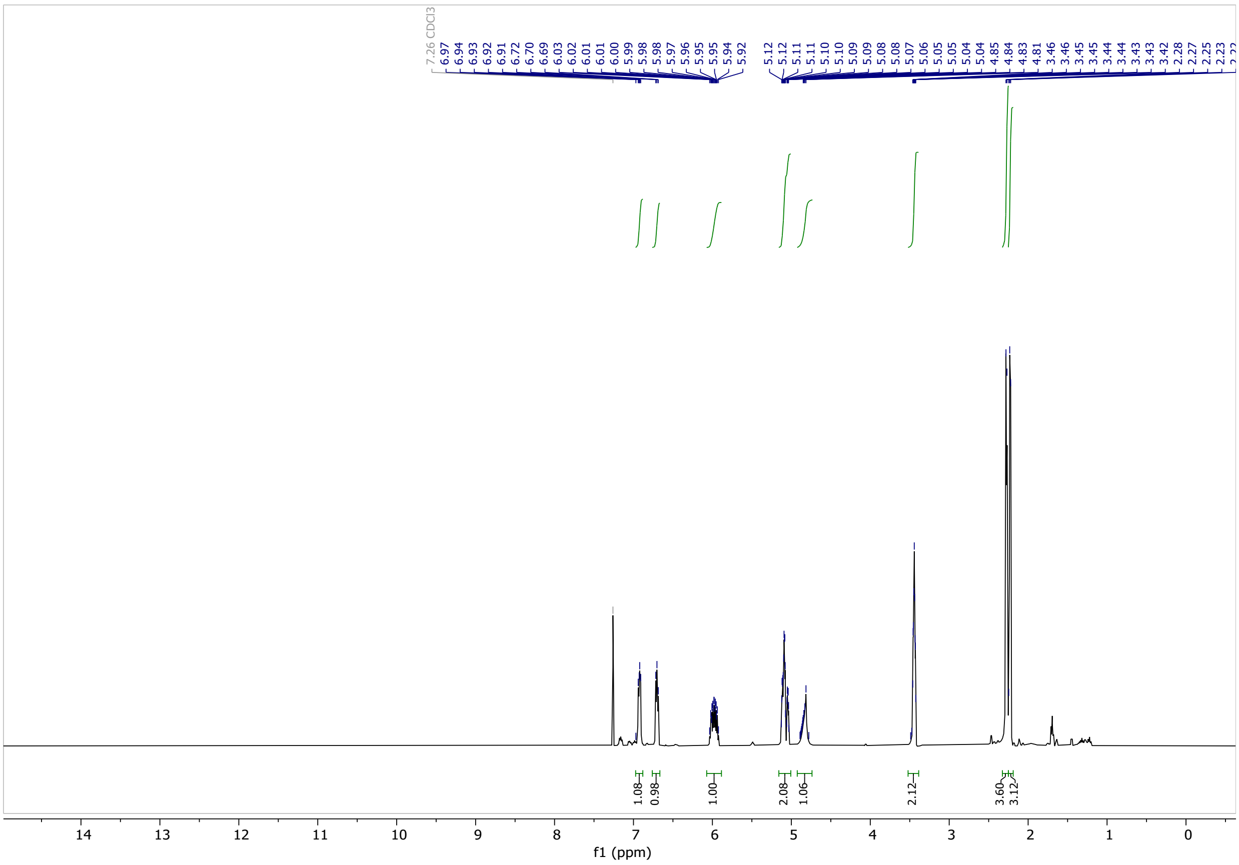

^{13}C NMR (101 MHz, $CDCl_3$)

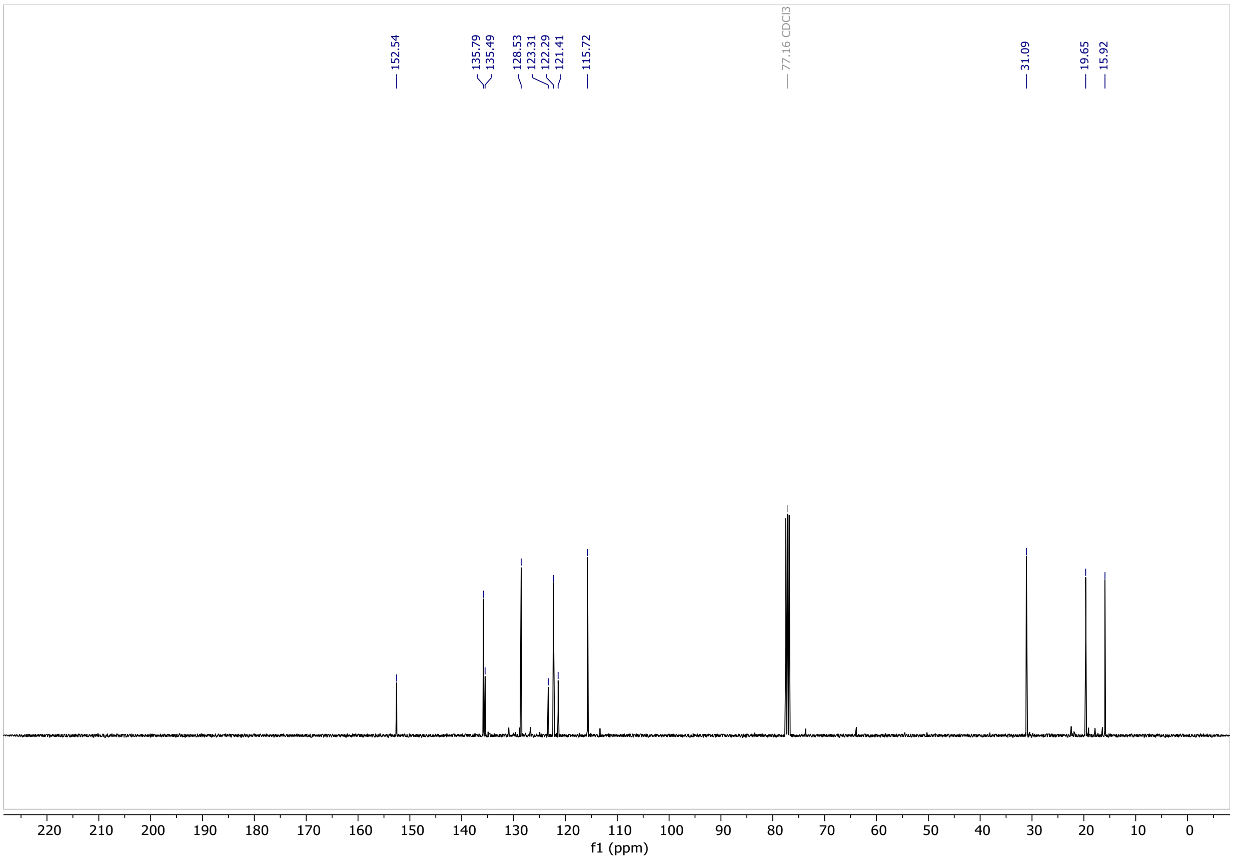

4-(But-3-en-2-yl)-2,5-dimethylphenol (5d) & (*E*)-2-(But-2-en-1-yl)-3,6-dimethylphenol (6d)

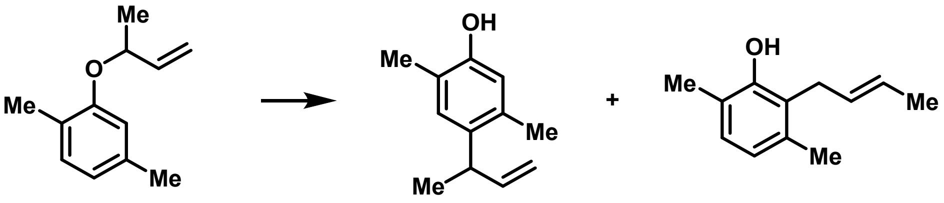

The title compounds were synthesized from **4d** (100 mg, 0.57 mmol) following **general procedure B**. The reaction was directly purified by column chromatography (petroleum ether/ethyl acetate 40:1 to 30:1 to 20:1) to provide the *para*-product **5d** as yellow oil in 54 % yield (54 mg, 0.31 mmol) and the *ortho*-product **6d** as pale green oil in 41 % yield (41 mg, 0.23 mmol).

4-(But-3-en-2-yl)-2,5-dimethylphenol (5d)

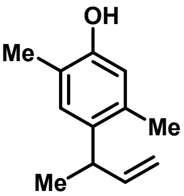

^{1}H NMR (400 MHz, $CDCl_3$) δ 6.94 (s, 1H), 6.71 (s, 1H), 6.04 – 5.91 (m, 1H), 5.27 (s, 1H), 5.05 – 4.97 (m, 2H), 3.65 – 3.55 (m, 1H), 2.31 (s, 3H), 2.26 (s, 3H), 1.32 (d, *J* = 7.0 Hz, 3H).

^{13}C NMR (101 MHz, $CDCl_3$) δ 152.9, 143.4, 136.0, 134.8, 129.2, 121.4, 117.5, 112.8, 38.1, 20.2, 19.1, 15.9.

^{1}H NMR (400 MHz, $CDCl_3$)

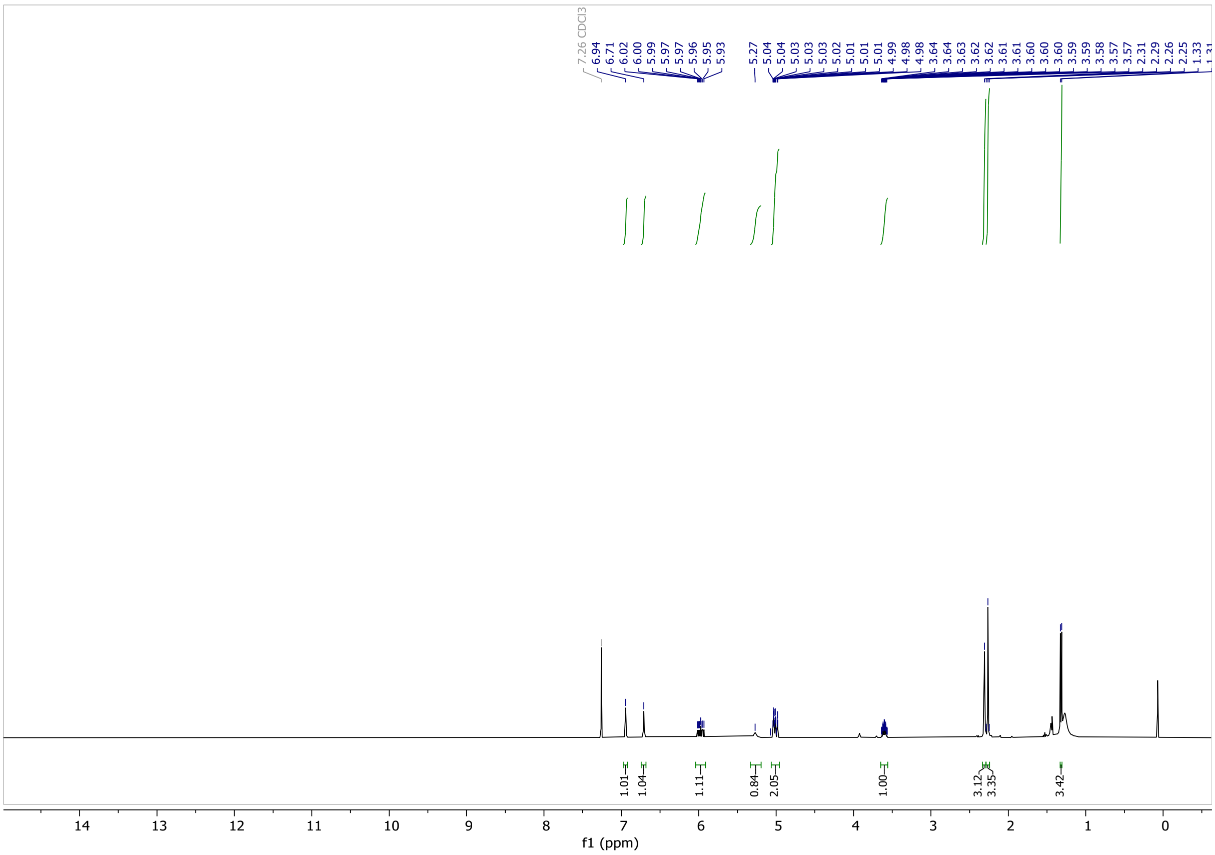

^{13}C NMR (101 MHz, $CDCl_3$)

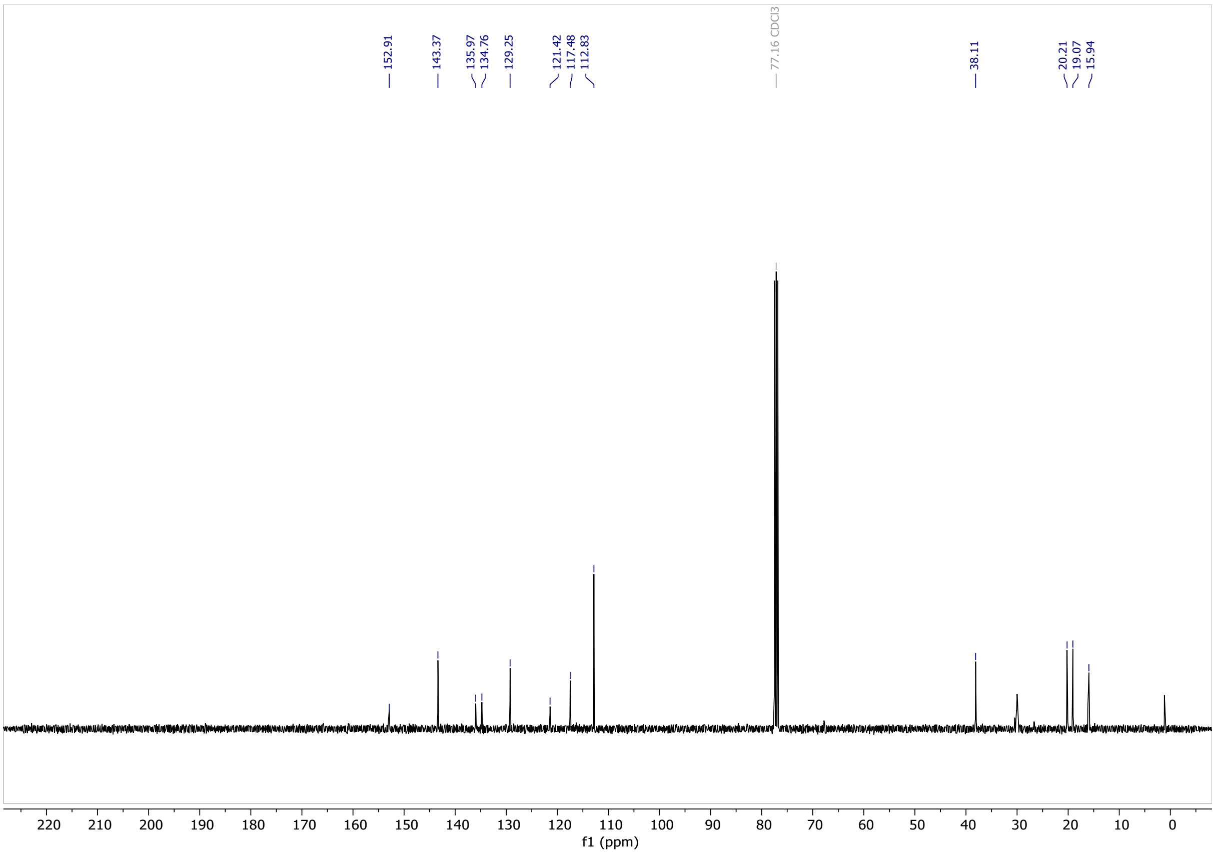

(*E*)-2-(But-2-en-1-yl)-3,6-dimethylphenol (6d)

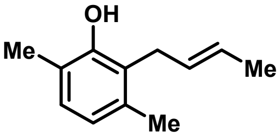

^{1}H NMR (400 MHz, $CDCl_3$) δ 6.93 – 6.88 (m, 1H), 6.68 (d, *J* = 7.6 Hz, 1H), 5.68 – 5.36 (m, 2H), 4.98 (s, 1H), 3.39 – 3.30 (m, 2H), 2.26 (s, 3H), 2.21 (s, 3H), 1.68 (dt, *J* = 4.8, 1.6 Hz, 3H).

^{13}C NMR (101 MHz, $CDCl_3$) δ 152.7, 135.1, 128.4, 128.3, 126.8, 123.9, 122.2, 121.7, 30.0, 19.7, 17.9, 15.9.

^{1}H NMR (400 MHz, $CDCl_3$)

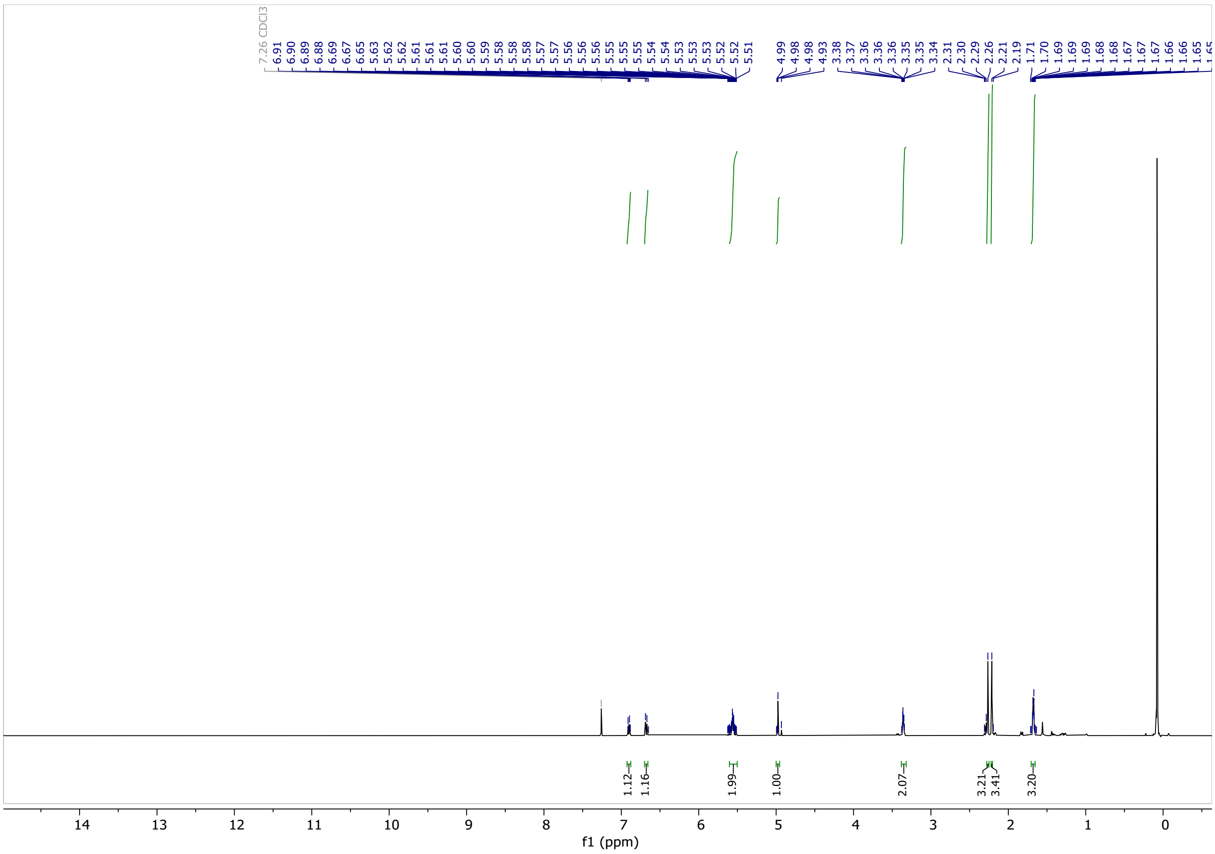

^{13}C NMR (101 MHz, $CDCl_3$)

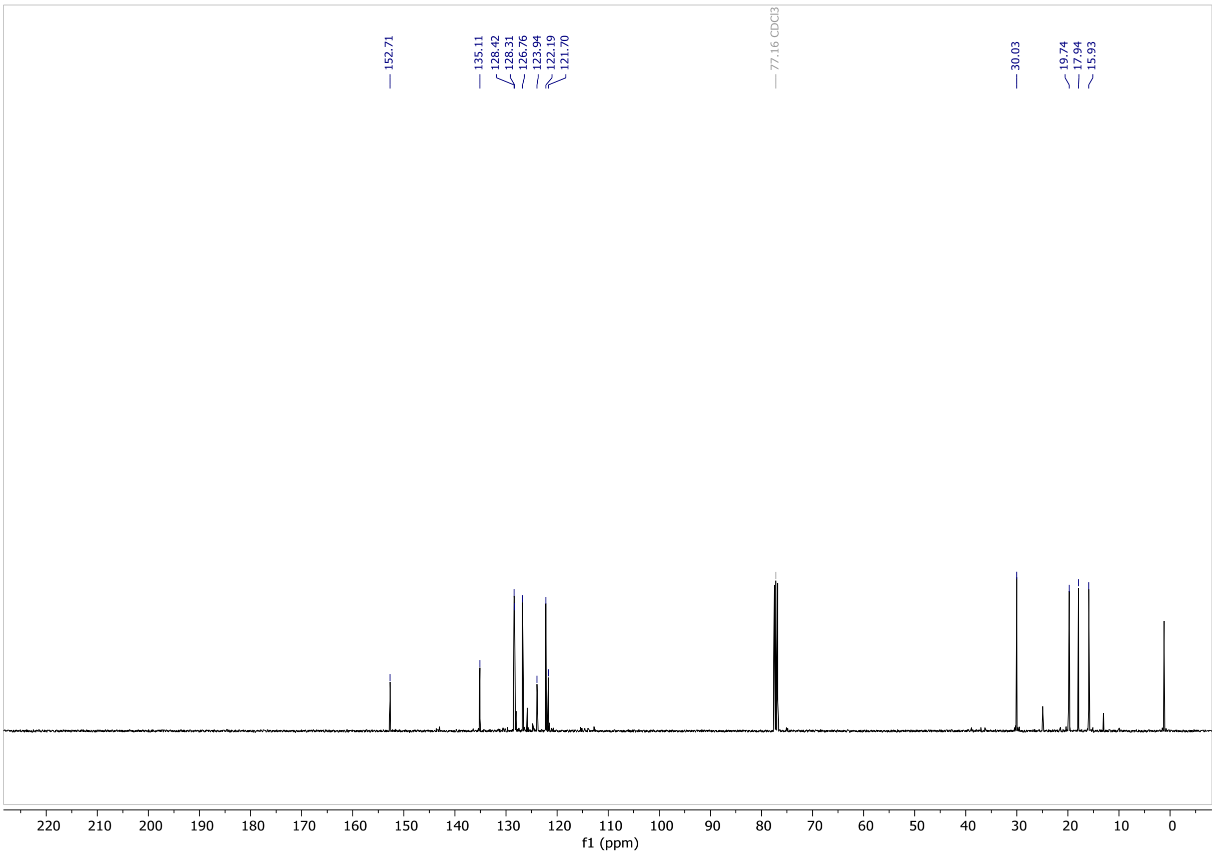

2,5-Dimethyl-4-(pent-3-yn-2-yl)phenol (5e) & 3,6-dimethyl-2-(penta-2,3-dien-2-yl)phenol (6e)

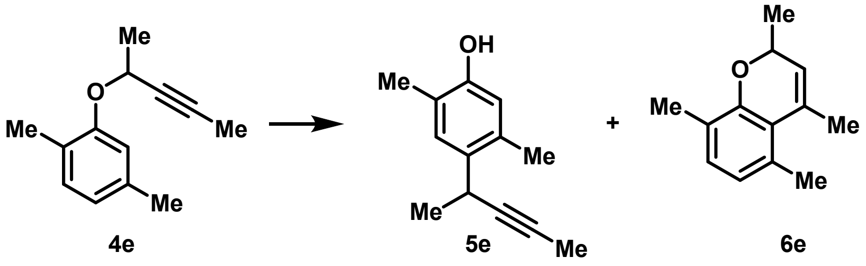

The title compounds were synthesized from **4e** (98 mg, 0.52 mmol) following **general procedure B at 110 °C**. The reaction was directly purified by column chromatography (petroleum ether/ethyl acetate 200:1 to 10:1) to provide the *para*-product **5e** as orange solids in 26% yield (25 mg, 0.13 mmol) and compound **6e** as colorless oil in 27% yield (26 mg, 0.14 mmol).

2,5-Dimethyl-4-(pent-3-yn-2-yl)phenol (5e)

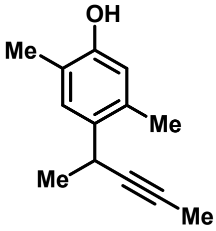

^{1}H NMR (400 MHz, $CDCl_3$) δ 7.22 (s, 1H), 6.55 (s, 1H), 4.50 (s, 1H), 3.78 (qq, *J* = 7.1, 2.4 Hz, 1H), 2.26 (s, 3H), 2.23 (s, 3H), 1.83 (d, *J* = 2.4 Hz, 3H), 1.38 (d, *J* = 7.1 Hz, 3H).

^{13}C NMR (101 MHz, $CDCl_3$) δ 152.2, 134.3, 133.7, 129.3, 121.3, 116.9, 82.9, 76.6, 27.7, 23.4, 18.8, 15.5, 3.8.

^{1}H NMR (400 MHz, $CDCl_3$)

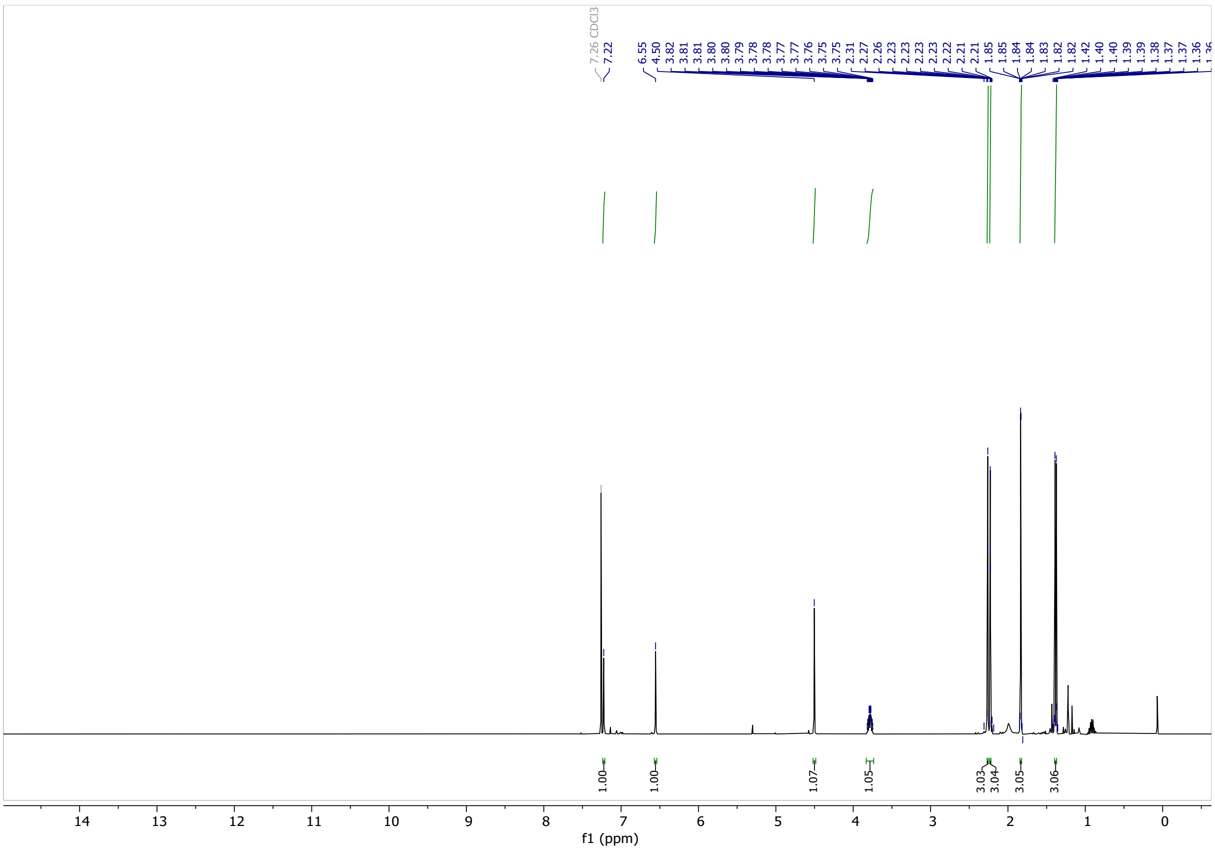

^{13}C NMR (101 MHz, $CDCl_3$)

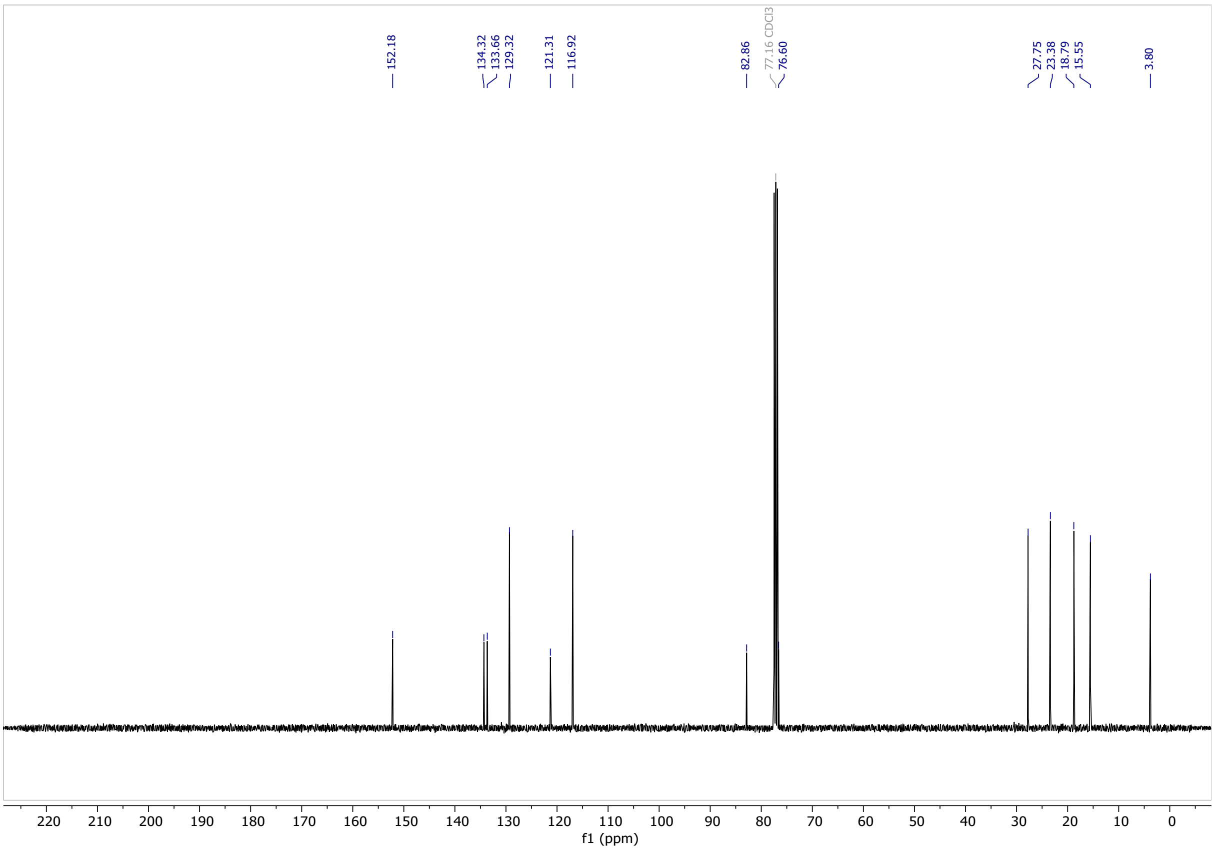

3,6-Dimethyl-2-(penta-2,3-dien-2-yl)phenol (6e)

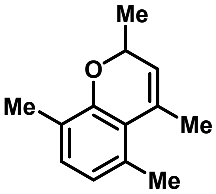

^{1}H NMR (400 MHz, $CDCl_3$) δ 6.90 (d, *J* = 7.6 Hz, 1H), 6.63 (d, *J* = 7.6 Hz, 1H), 5.53 (dq, *J* = 3.1, 1.5 Hz, 1H), 4.57 (ddddt, *J* = 8.2, 6.6, 5.0, 3.4, 1.5 Hz, 1H), 2.44 (s, 3H), 2.18 (s, 6H), 1.42 (d, *J* = 6.6 Hz, 3H).

^{13}C NMR (101 MHz, $CDCl_3$) δ 153.3, 132.4, 129.8, 126.3, 124.1, 124.0, 123.6, 70.2, 23.0, 22.7, 20.3, 16.2.

^{1}H NMR (400 MHz, $CDCl_3$)

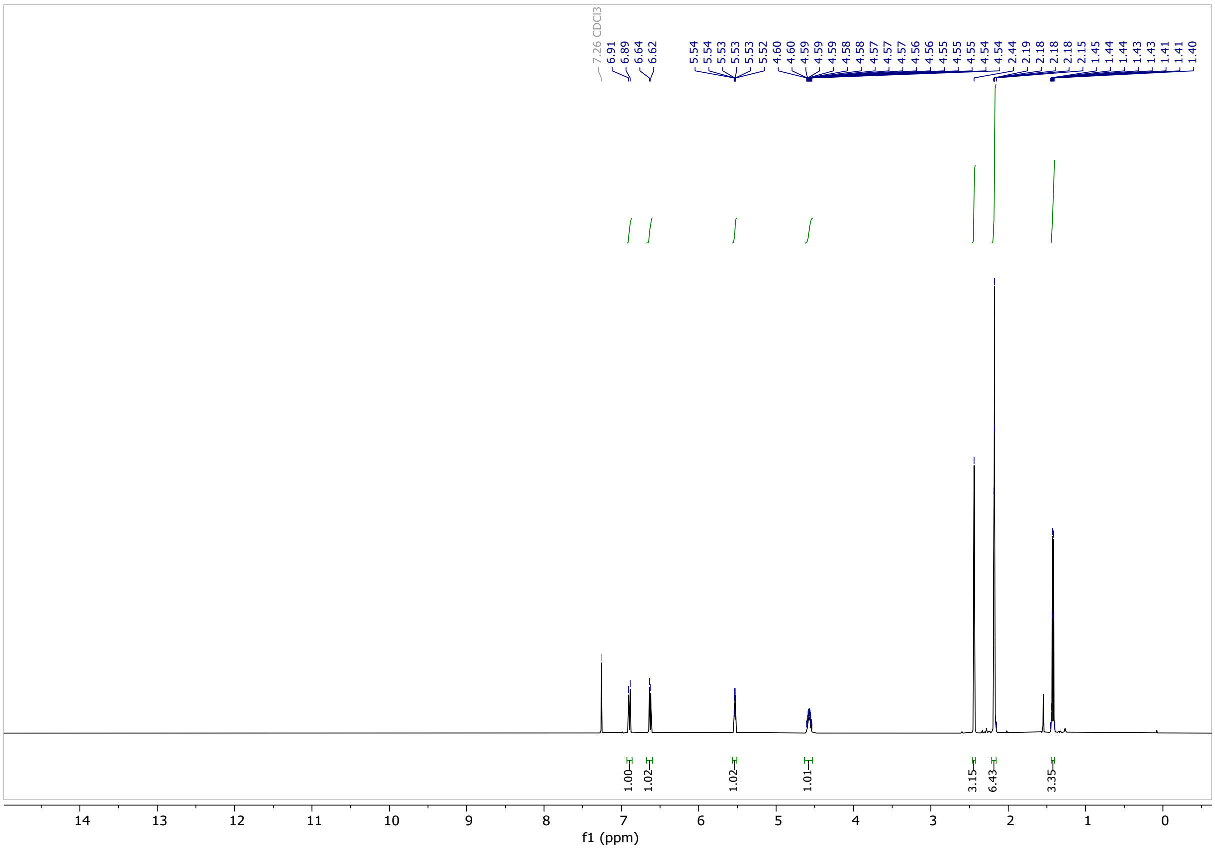

^{13}C NMR (101 MHz, $CDCl_3$)

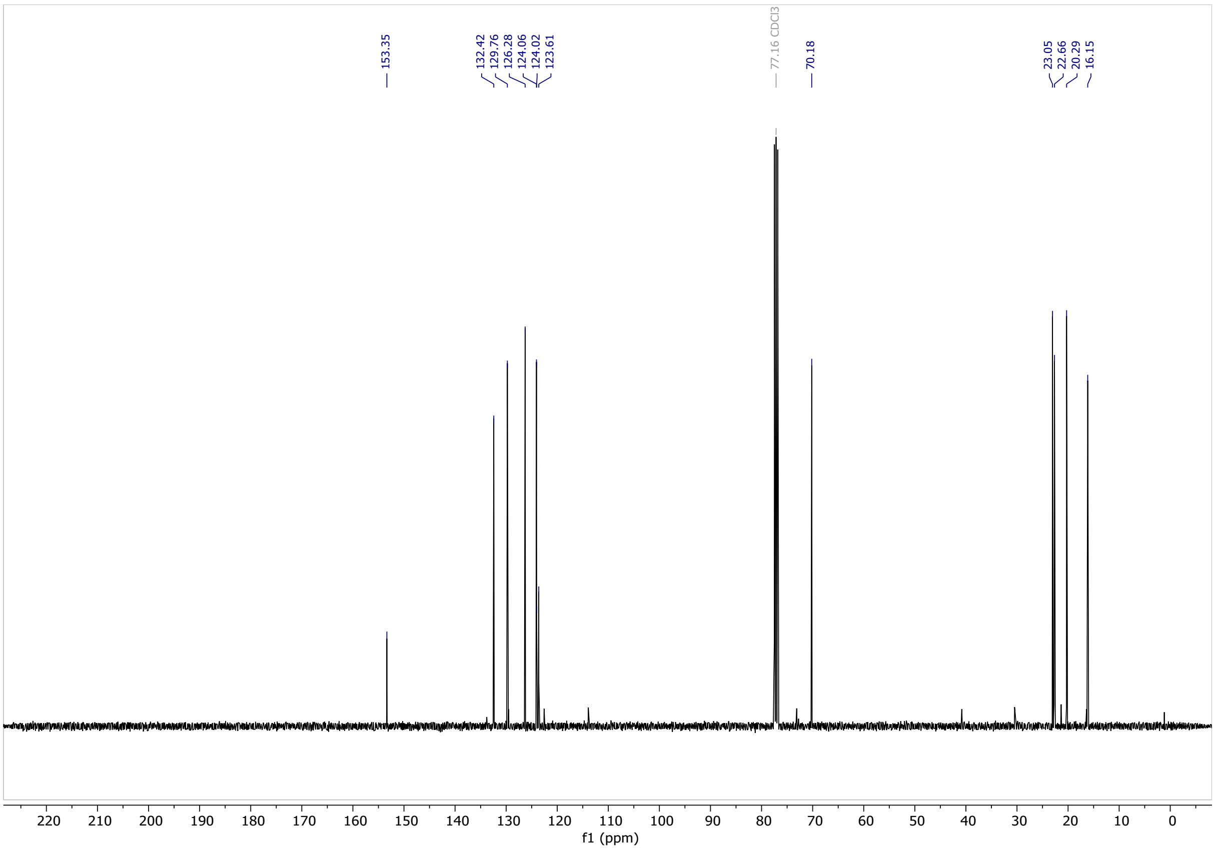

Limitations

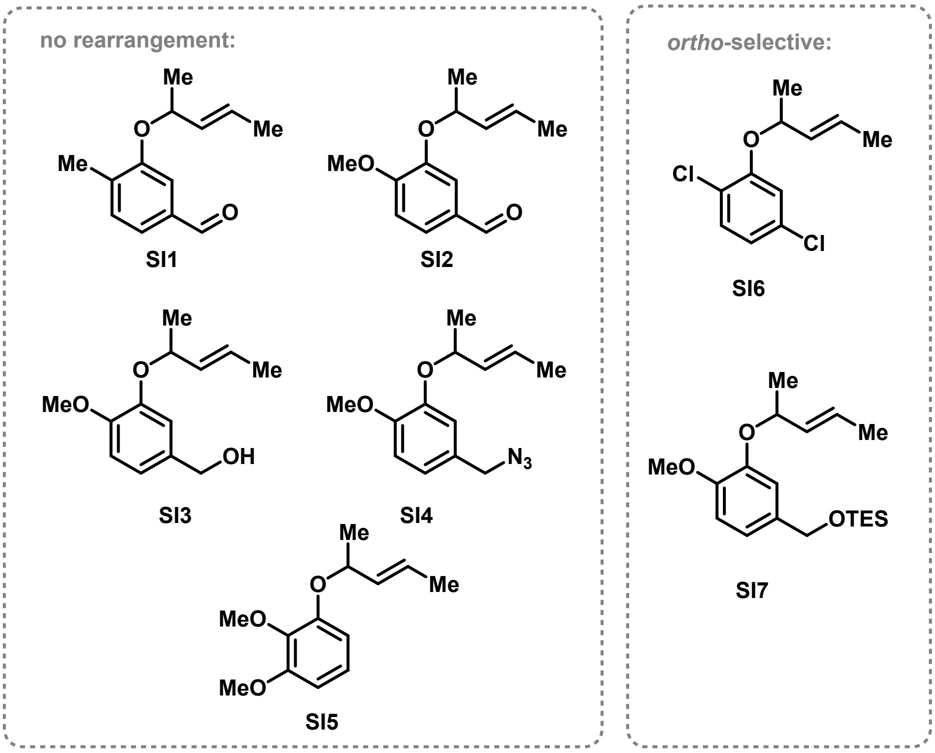

During substrate scope exploration, we faced difficulties with the rearrangement of certain compounds. Compounds **SI1-SI5** were successfully synthesized as racemates. However, the subsequent rearrangement did not provide satisfactory results, as compounds **SI1-5** failed to give conversion at 60 °C (and 100 °C). Compounds **SI6-7** afforded selectively the *ortho*-product. As a consequence, these compounds were excluded from further studies, and no asymmetric synthesis was attempted.

(*E*)-4-Methyl-3-(pent-3-en-2-yloxy)benzaldehyde (SI1)

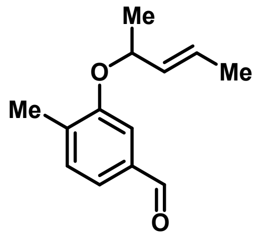

The title compound was synthesized from commercially available 3-hydroxy-4-methylbenzaldehyde (192 mg, 1.38 mmol) following *racemic* **general procedure A**. The crude material was purified by column chromatography (petroleum ether/ethyl acetate 40:1) to provide the desired product **SI1** as colorless oil in 97% yield (274 mg, 1.34 mmol).

^{1}H NMR (400 MHz, $CDCl_3$) δ 9.89 (s, 1H), 7.36 – 7.26 (m, 3H), 5.74 (dqd, *J* = 15.4, 6.4, 1.0 Hz, 1H), 5.53 (ddq, *J* = 15.5, 6.5, 1.6 Hz, 1H), 4.97 – 4.75 (m, 1H), 2.29 (s, 3H), 1.69 (ddd, *J* = 6.5, 1.7, 0.8 Hz, 3H), 1.43 (d, *J* = 6.3 Hz, 3H).

^{13}C NMR (101 MHz, $CDCl_3$) δ 192.2, 156.9, 135.9, 135.8, 131.9, 131.1, 127.7, 124.0, 111.6, 74.8, 21.7, 17.9, 17.2.

HRMS (ESI): exact mass calculated for $C_{13}H_{17}O_2^+$ [$(M + H)^+$], 205.1223; found 205.1218.

^{1}H NMR (400 MHz, $CDCl_3$)

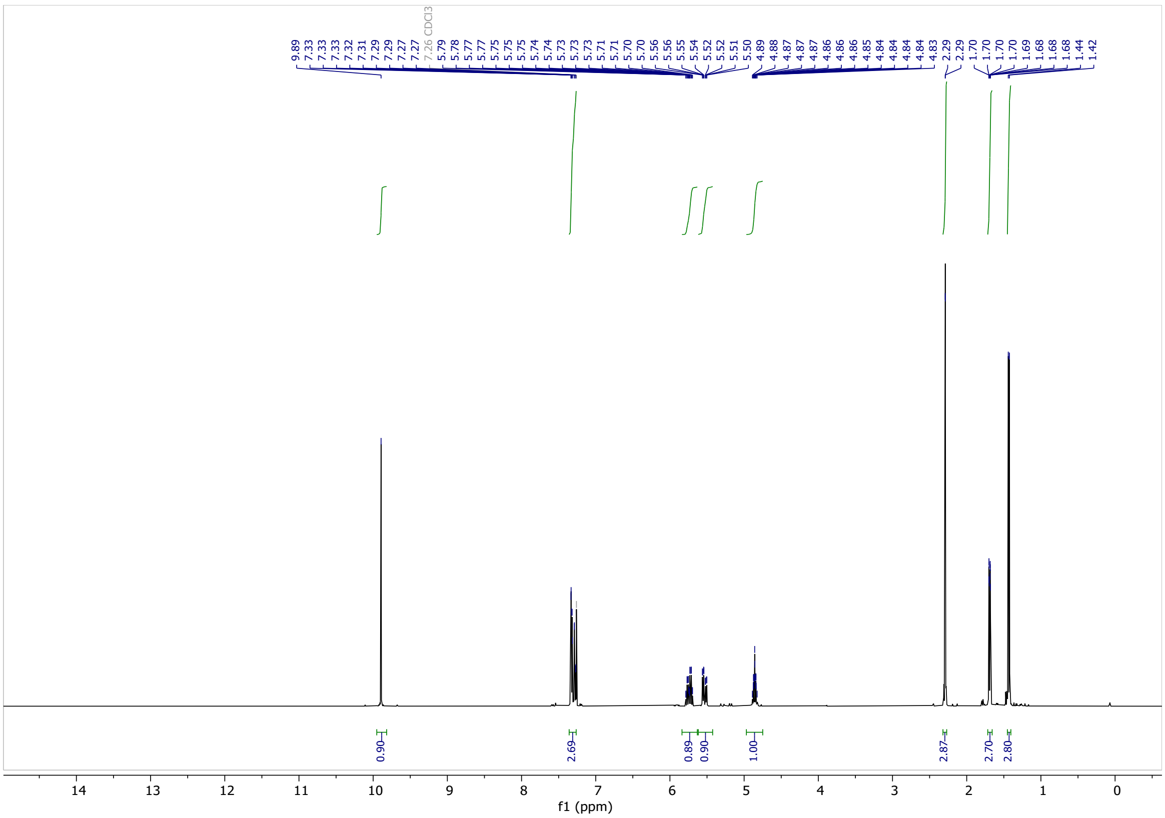

^{13}C NMR (101 MHz, $CDCl_3$)

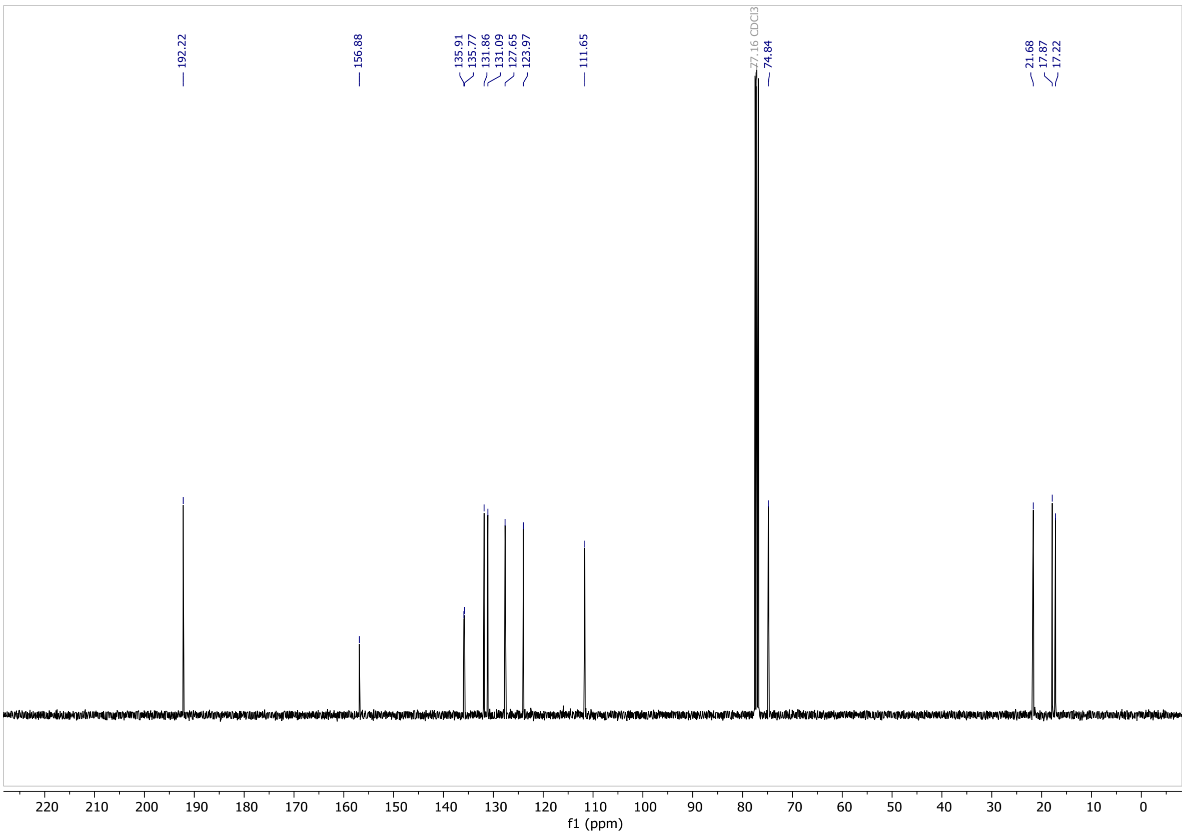

***(E)*-4-Methoxy-3-(pent-3-en-2-yloxy)benzaldehyde (SI2)**

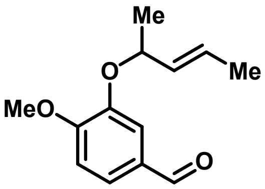

The title compound was synthesized from commercially available 3-hydroxy-4-methoxybenzaldehyde (154 mg, 0.99 mmol) following *racemic* **general procedure A**. The crude material was purified by column chromatography (petroleum ether/ethyl acetate 40:1) to provide the desired product **SI2** as colorless oil in 75% yield (164 mg, 0.75 mmol).

^{1}H NMR (400 MHz, $CDCl_3$) δ 9.81 (s, 1H), 7.48 – 7.32 (m, 2H), 6.95 (d, *J* = 8.0 Hz, 1H), 5.73 (dqd, *J* = 15.5, 6.4, 0.9 Hz, 1H), 5.56 (ddq, *J* = 15.4, 6.9, 1.6 Hz, 1H), 4.84 (p, *J* = 6.4 Hz, 1H), 3.92 (s, 3H), 1.67 (ddd, *J* = 6.4, 1.6, 0.7 Hz, 3H), 1.46 (d, *J* = 6.3 Hz, 3H).

^{13}C NMR (101 MHz, $CDCl_3$) δ 191.1, 155.7, 148.0, 131.5, 130.0, 128.3, 126.5, 113.7, 110.9, 75.9, 56.2, 21.4, 17.8.

HRMS (ESI): exact mass calculated for $C_{13}H_{17}O_3^+$ [$(M + H)^+$], 221.1172; found 221.1165.

^{1}H NMR (400 MHz, $CDCl_3$)

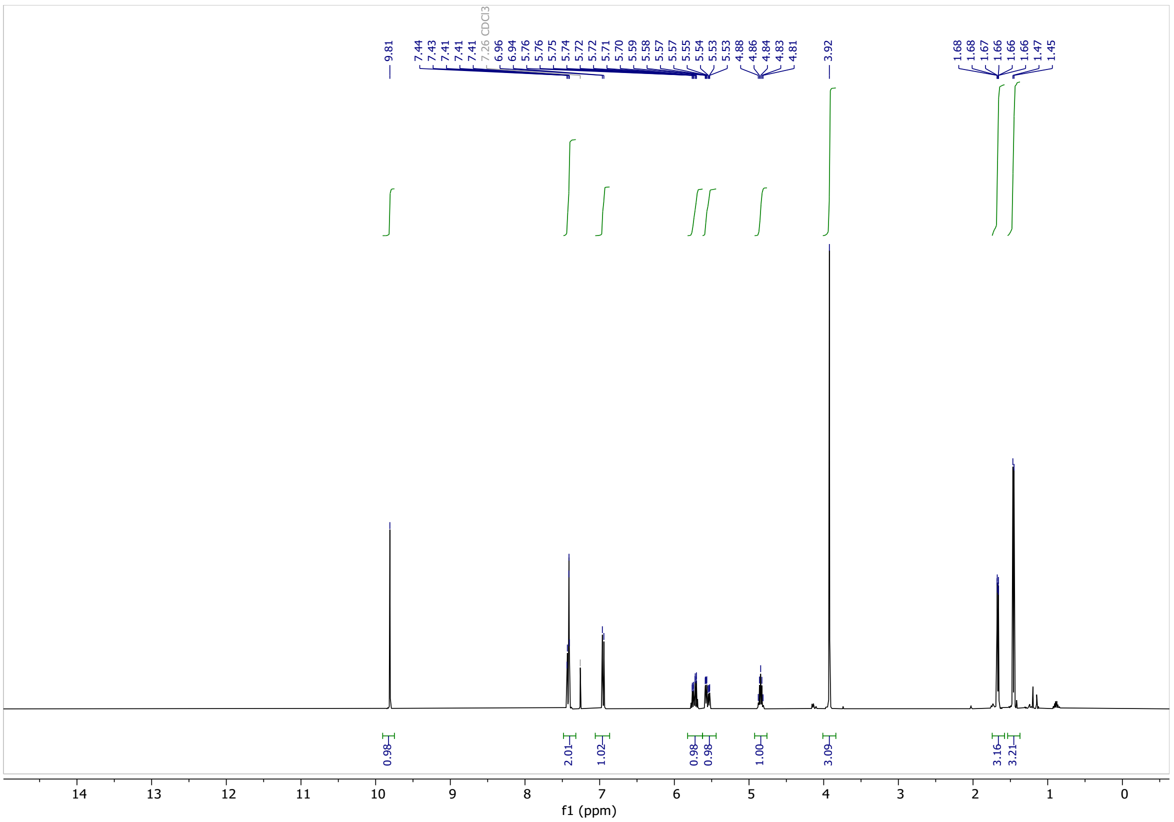

^{13}C NMR (101 MHz, $CDCl_3$)

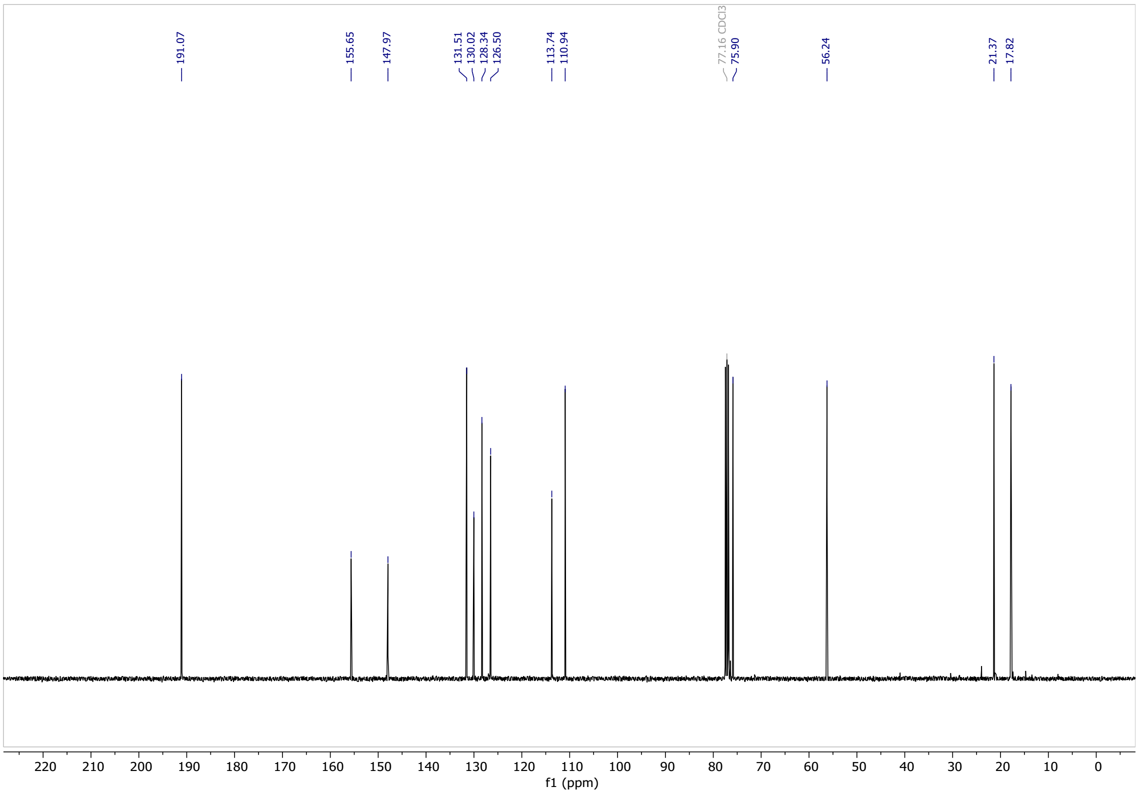

***(E)*-(4-Methoxy-3-(pent-3-en-2-yloxy)phenyl)methanol (SI3)**

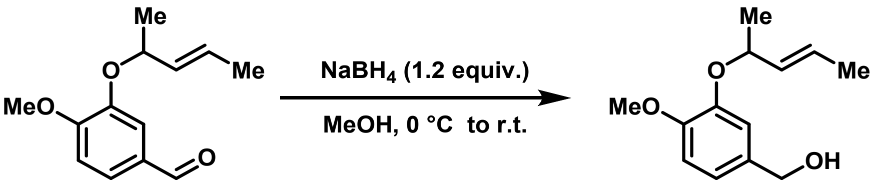

In a round bottom flask compound **SI2** (1.00 g, 4.54 mmol, 1 eq.) was dissolved in 15 mL methanol and cooled by an ice bath. Sodium borohydride (210 mg, 5.45 mmol, 1.2 eq.) was added in portions and the reaction mixture was allowed to warm to room temperature. After TLC (petroleum ether/ethyl acetate 5:1) confirmed full conversion acetone and saturated NH_4Cl was added. The aqueous phase was extracted with DCM three times, the combined organic layer was dried over $MgSO_4$, filtered and concentrated *in vacuo*. The crude material was flashed over a plug of silica (petroleum ether/ethyl acetate 10:1) to provide the desired product **SI3** as colorless oil in 97% yield (980 mg, 4.41 mmol).

^{1}H NMR (400 MHz, $CDCl_3$) δ 6.95 – 6.86 (m, 2H), 6.84 (dd, *J* = 8.2, 1.2 Hz, 1H), 5.76 – 5.63 (m, 1H), 5.63 – 5.50 (m, 1H), 4.82 – 4.69 (m, 1H), 4.58 (d, *J* = 1.9 Hz, 2H), 3.85 (d, *J* = 1.3 Hz, 3H), 1.68 (ddt, *J* = 6.3, 1.6, 0.7 Hz, 3H), 1.44 (dd, *J* = 6.3, 0.8 Hz, 3H).

^{13}C NMR (101 MHz, $CDCl_3$) δ 150.0, 147.6, 133.5, 132.3, 127.6, 120.1, 115.6, 111.9, 76.0, 65.5, 56.2, 21.5, 17.9.

^{1}H NMR (400 MHz, $CDCl_3$)

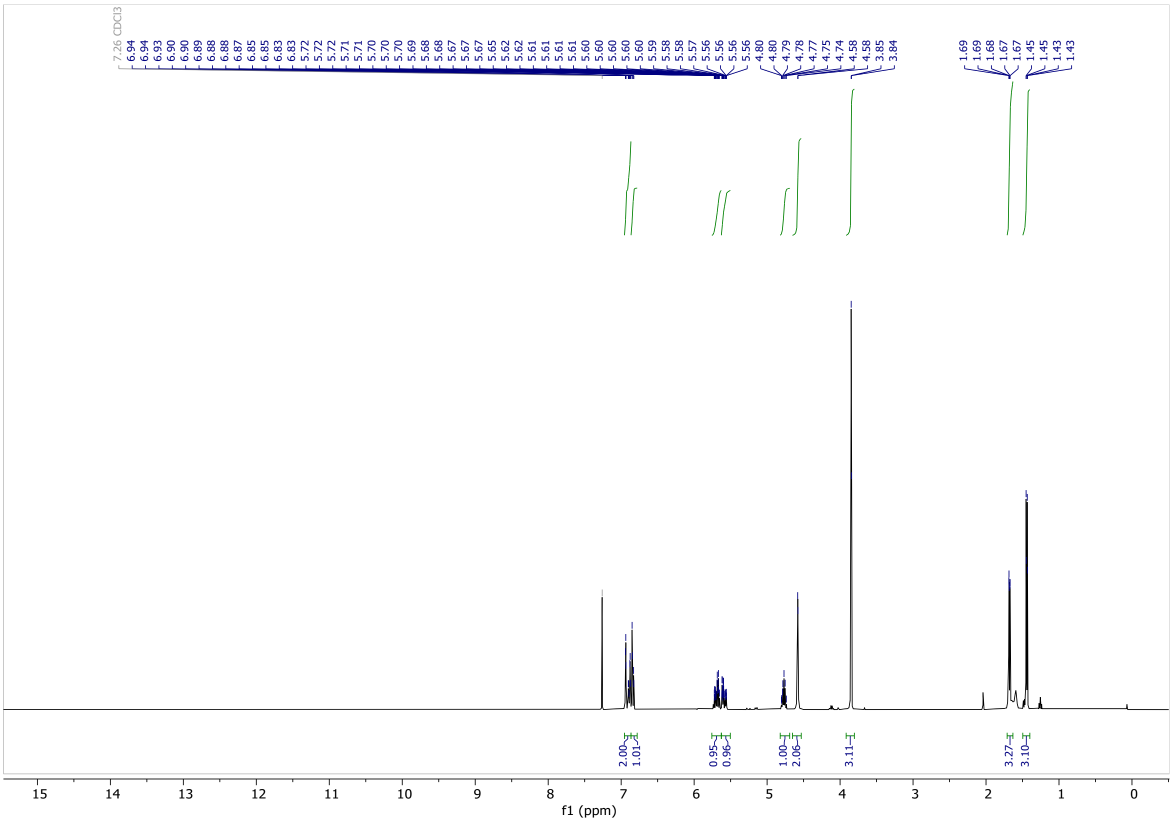

^{13}C NMR (101 MHz, $CDCl_3$)

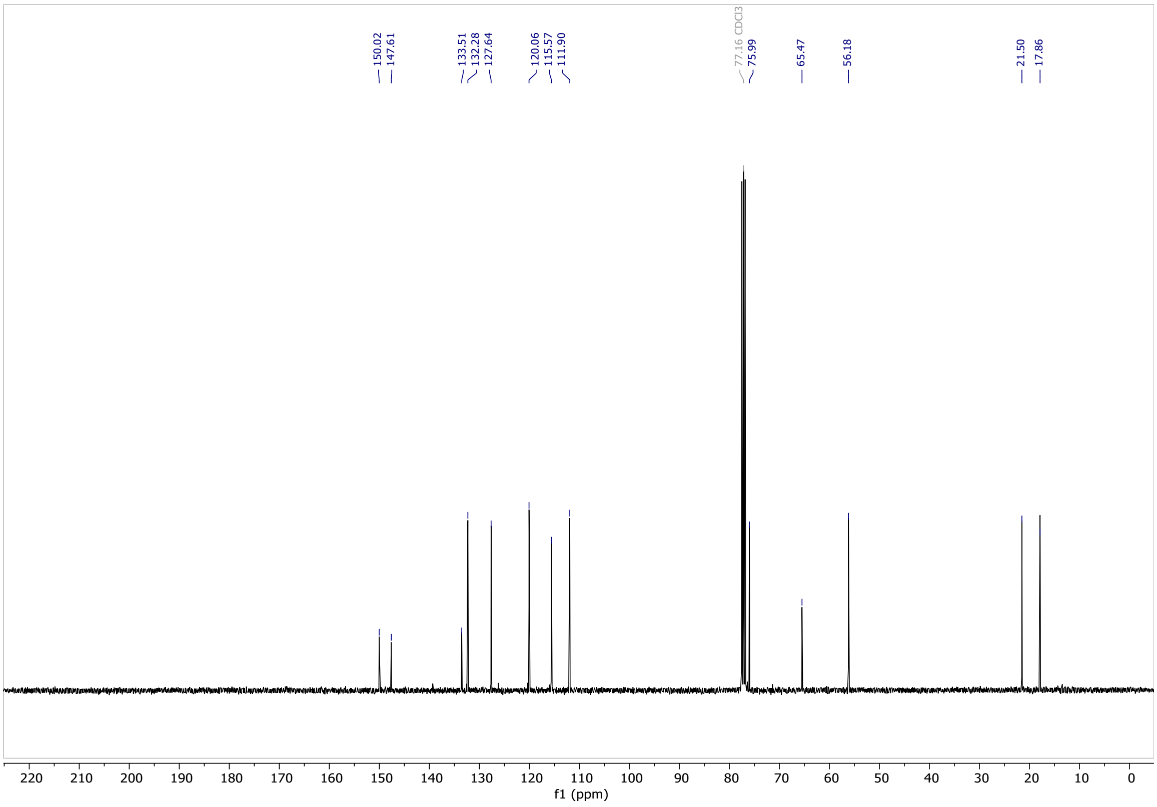

***(E)*-4-(Azidomethyl)-1-methoxy-2-(pent-3-en-2-yloxy)benzene (SI4)**

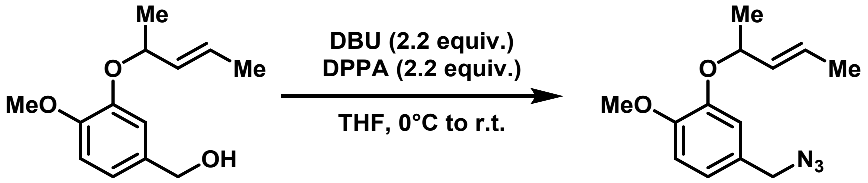

A 10 mL Schlenk flask was charged with compound **SI3** (265 mg, 1.19 mmol, 1 eq.) dissolved in 3 mL dry THF and cooled by an ice bath. DBU (399 mg, 2.62 mmol, 2.2 eq.) was added dropwise, followed by DPPA (744 mg, 2.62 mmol, 2.2 eq.). The reaction mixture turned turbid and was allowed to warm to room temperature. After TLC (petroleum ether/ethyl acetate 5:1) confirmed full conversion ethylacetate was added. The organic layer was washed with water three times, dried over $MgSO_4$, filtered and concentrated *in vacuo*. The crude material was purified via column chromatography (petroleum ether/ethyl acetate 20:1) to provide the desired product **SI4** as colorless oil in 80% yield (236 mg, 0.95 mmol).

^{1}H NMR (400 MHz, $CDCl_3$) δ 6.86 (d, *J* = 1.0 Hz, 1H), 6.85 (d, *J* = 1.1 Hz, 2H), 5.76 – 5.60 (m, 1H), 5.60 – 5.50 (m, 1H), 4.82 – 4.70 (m, 1H), 4.22 (s, 2H), 3.86 (s, 3H), 1.68 (ddd, *J* = 6.4, 1.5, 0.7 Hz, 3H), 1.46 (d, *J* = 6.3 Hz, 3H).

^{13}C NMR (101 MHz, $CDCl_3$) δ 150.43, 147.53, 132.00, 127.83, 127.51, 121.37, 116.62, 111.82, 76.06, 55.97, 54.68, 21.44, 17.70.

^{1}H NMR (400 MHz, $CDCl_3$)

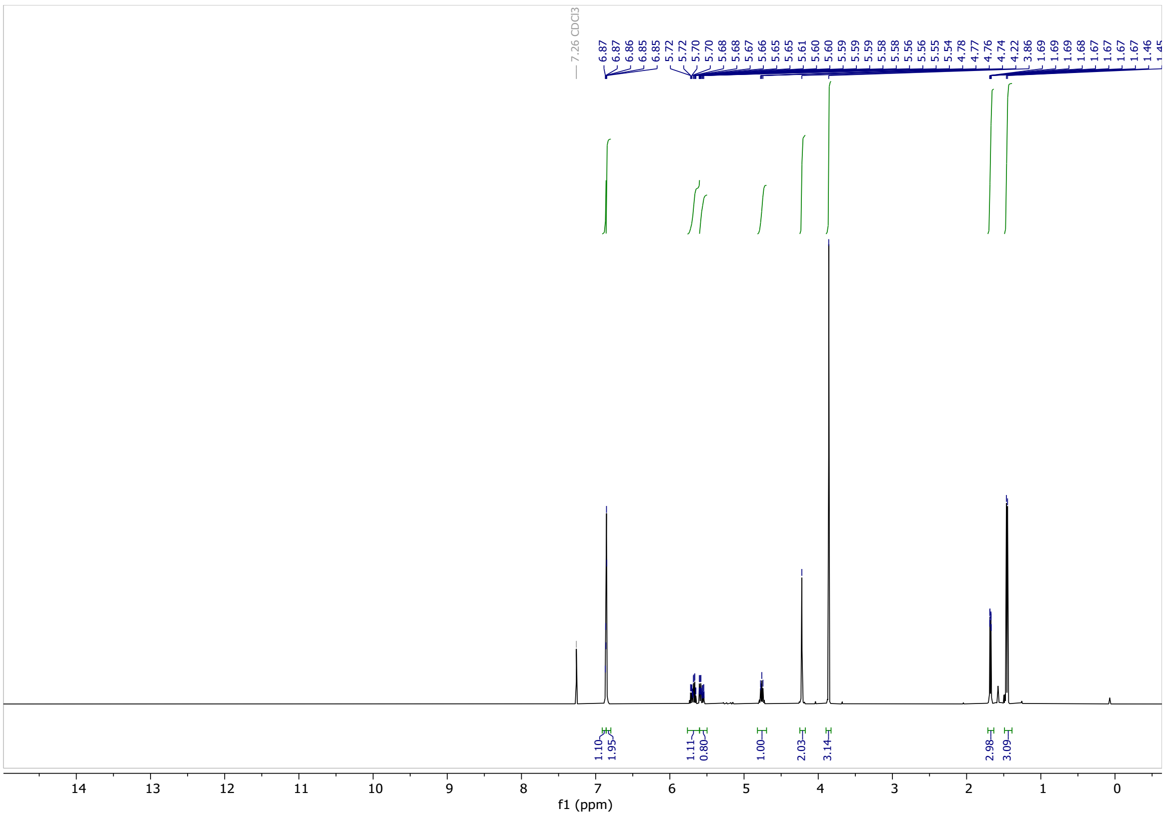

^{13}C NMR (101 MHz, $CDCl_3$)

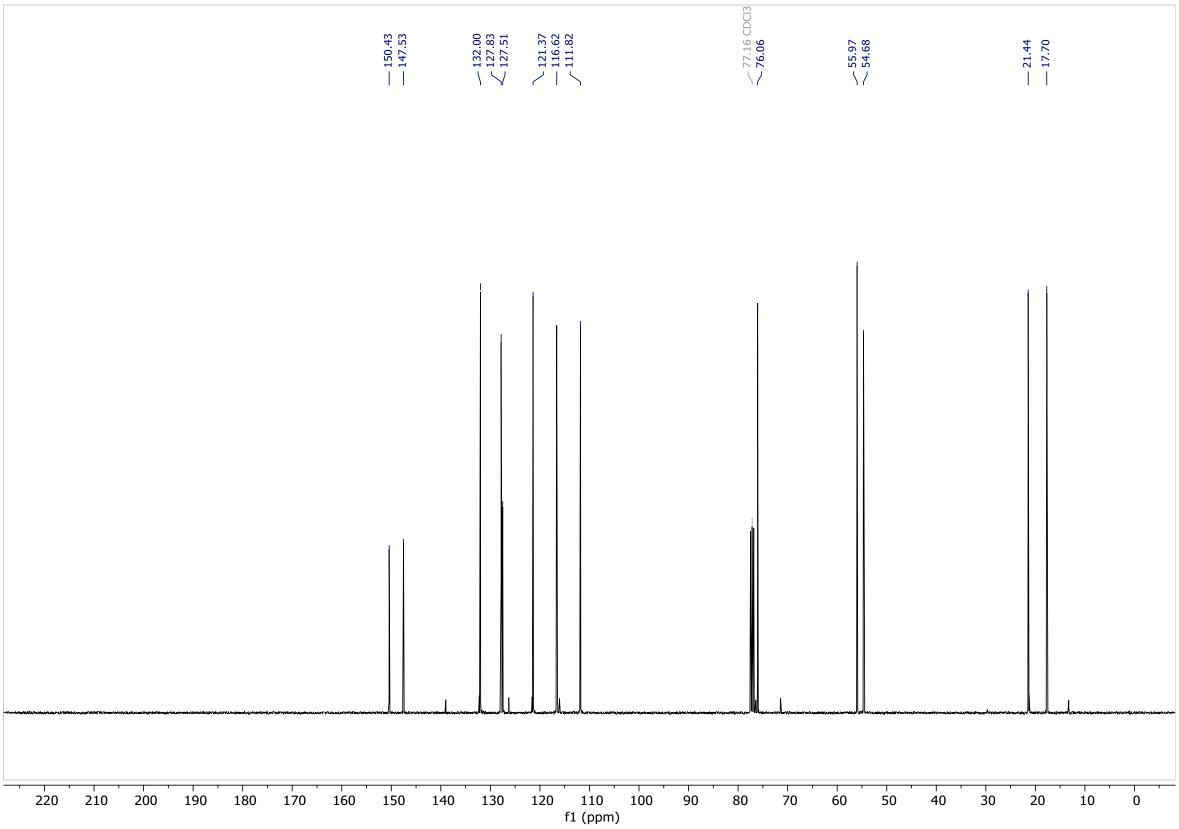

***(E)*-1,2-Dimethoxy-3-(pent-3-en-2-yloxy)benzene (SI5)**

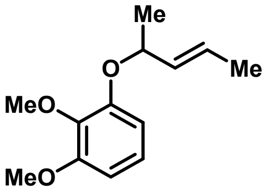

The title compound was synthesized from commercially available 2,3-dimethoxyphenol (154 mg, 0.98 mmol) following *racemic* **general procedure A**. The crude material was purified by column chromatography (petroleum ether/ethyl acetate 40:1) to provide the desired product **SI5** as colorless oil in 83% yield (181 mg, 0.81 mmol).

^{1}H NMR (400 MHz, $CDCl_3$) δ 6.91 (td, *J* = 8.3, 1.0 Hz, 1H), 6.57 (ddd, *J* = 11.7, 8.4, 1.3 Hz, 2H), 5.69 (dqd, *J* = 15.4, 6.3, 0.9 Hz, 1H), 5.57 (ddq, *J* = 15.4, 6.6, 1.4 Hz, 1H), 4.79 – 4.69 (m, 1H), 3.90 – 3.79 (m, 6H), 1.68 (ddd, *J* = 6.3, 1.5, 0.8 Hz, 3H), 1.43 (d, *J* = 6.4 Hz, 3H).

^{13}C NMR (101 MHz, $CDCl_3$) δ 153.8, 152.0, 139.7, 132.4, 127.4, 123.3, 109.9, 105.4, 76.1, 60.8, 56.2, 21.7, 17.8.

^{1}H NMR (400 MHz, $CDCl_3$)

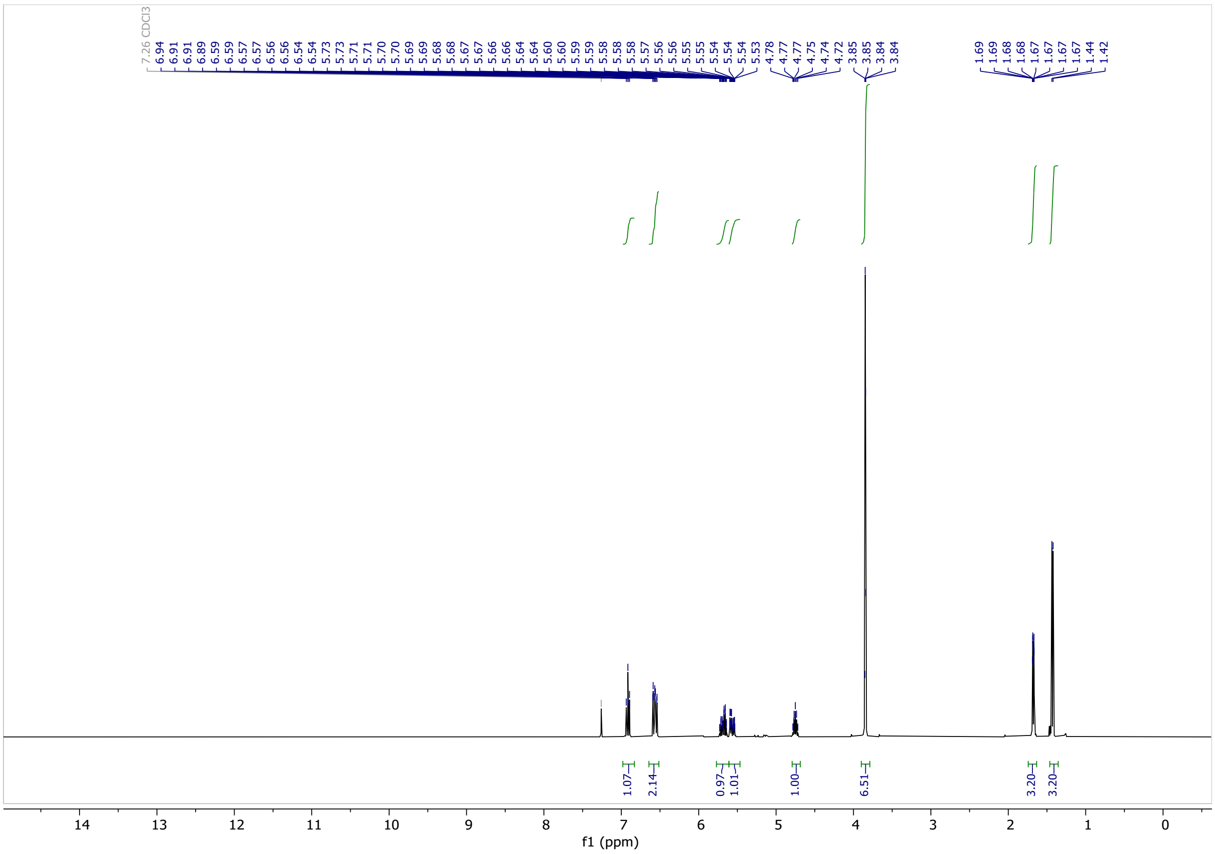

^{13}C NMR (101 MHz, $CDCl_3$)

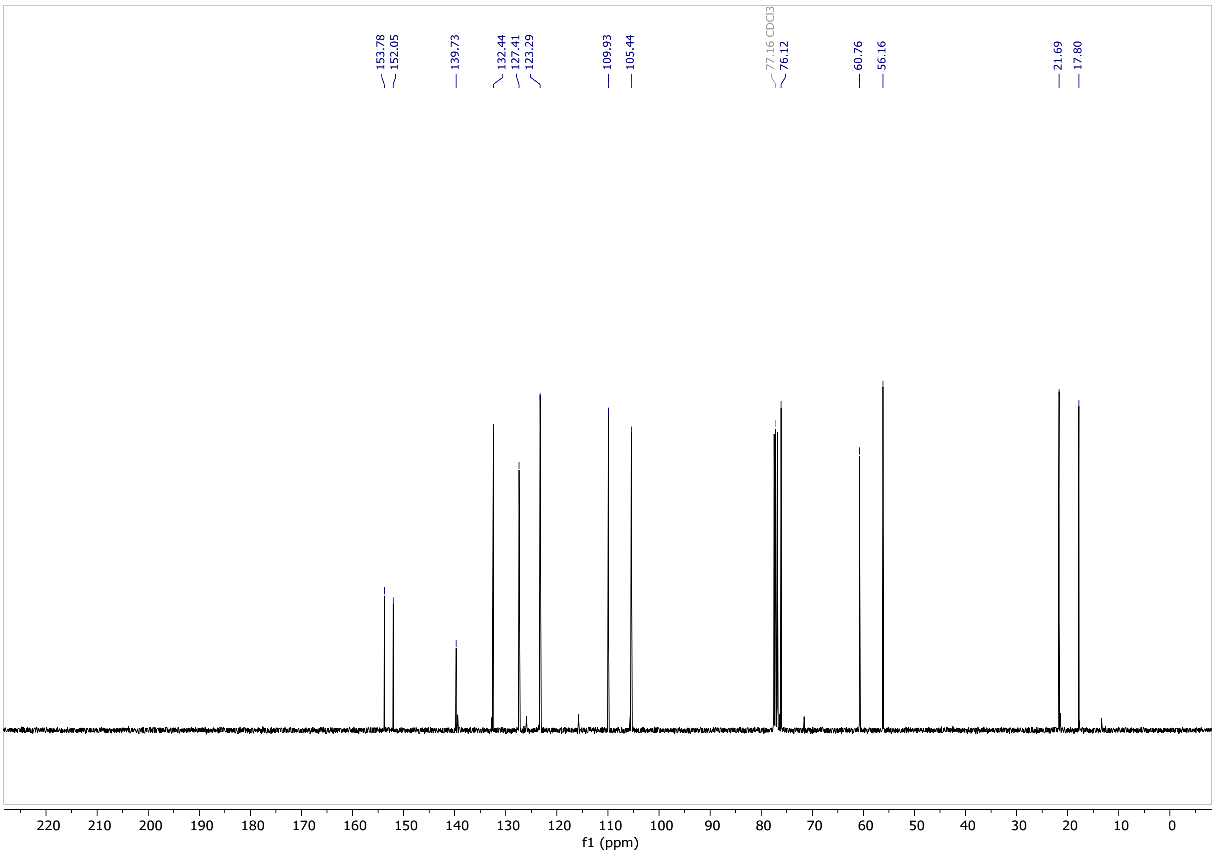

_(E)_-1,4-Dichloro-2-(pent-3-en-2-yloxy)benzene (SI6)

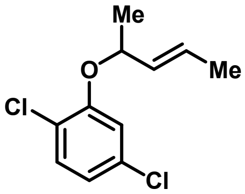

The title compound was synthesized from commercially available 2,5-dichlorophenol (160 mg, 0.96 mmol) following _racemic_ **general procedure A**. The crude material was purified by column chromatography (petroleum ether/ethyl acetate 40:1) to provide the desired product **#** as colorless oil in 84% yield (187 mg, 0.81 mmol).

^{1}H NMR (400 MHz, $CDCl_3$) δ 7.28 (s, 1H), 6.94 (d, J = 2.3 Hz, 1H), 6.87 (dd, J = 8.4, 2.3 Hz, 1H), 5.74 (dqd, J = 15.5, 6.4, 1.0 Hz, 1H), 5.55 (ddq, J = 15.4, 6.7, 1.6 Hz, 1H), 4.76 (p, J = 6.4 Hz, 1H), 1.72 (ddd, J = 6.5, 1.6, 0.8 Hz, 3H), 1.48 (d, J = 6.3 Hz, 3H).

^{13}C NMR (101 MHz, $CDCl_3$) δ 154.3, 132.8, 131.2, 130.8, 128.4, 122.6, 121.5, 116.7, 21.6, 17.9.

^{1}H NMR (400 MHz, $CDCl_3$)

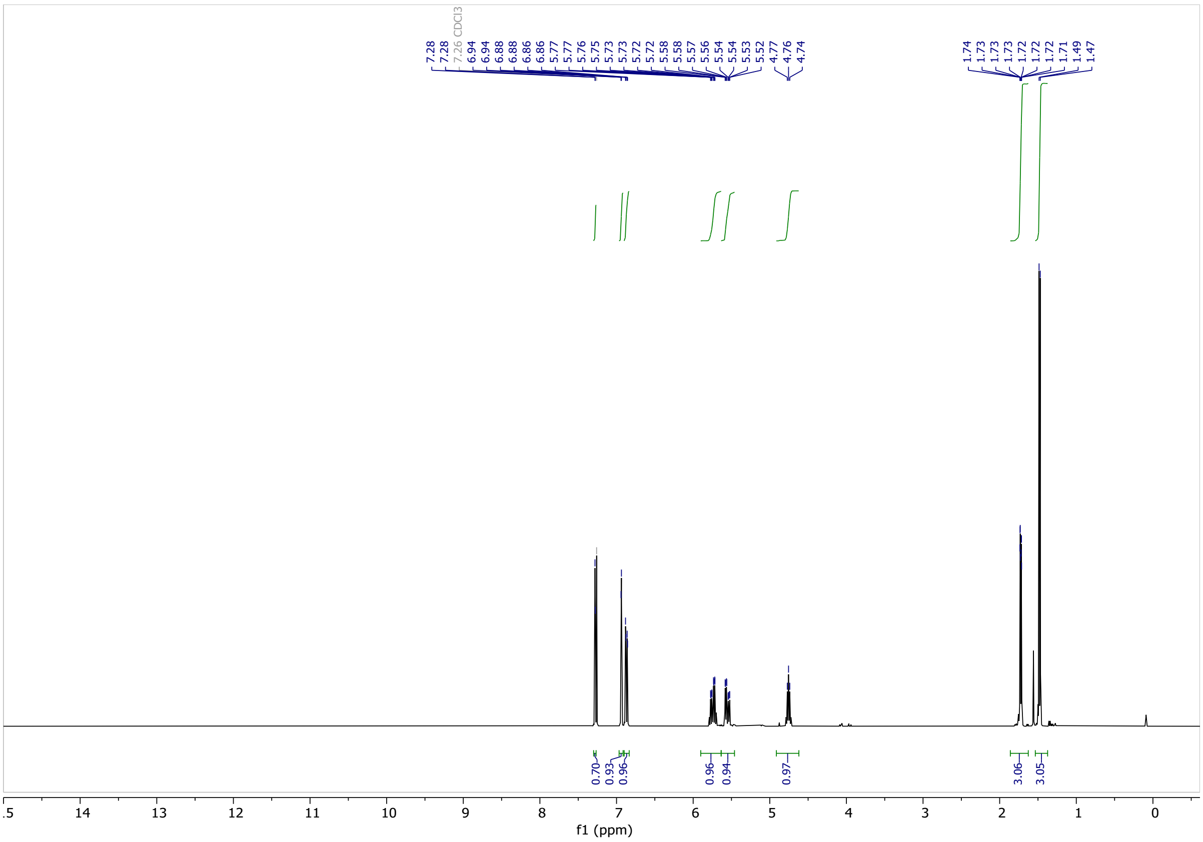

^{13}C NMR (101 MHz, $CDCl_3$)

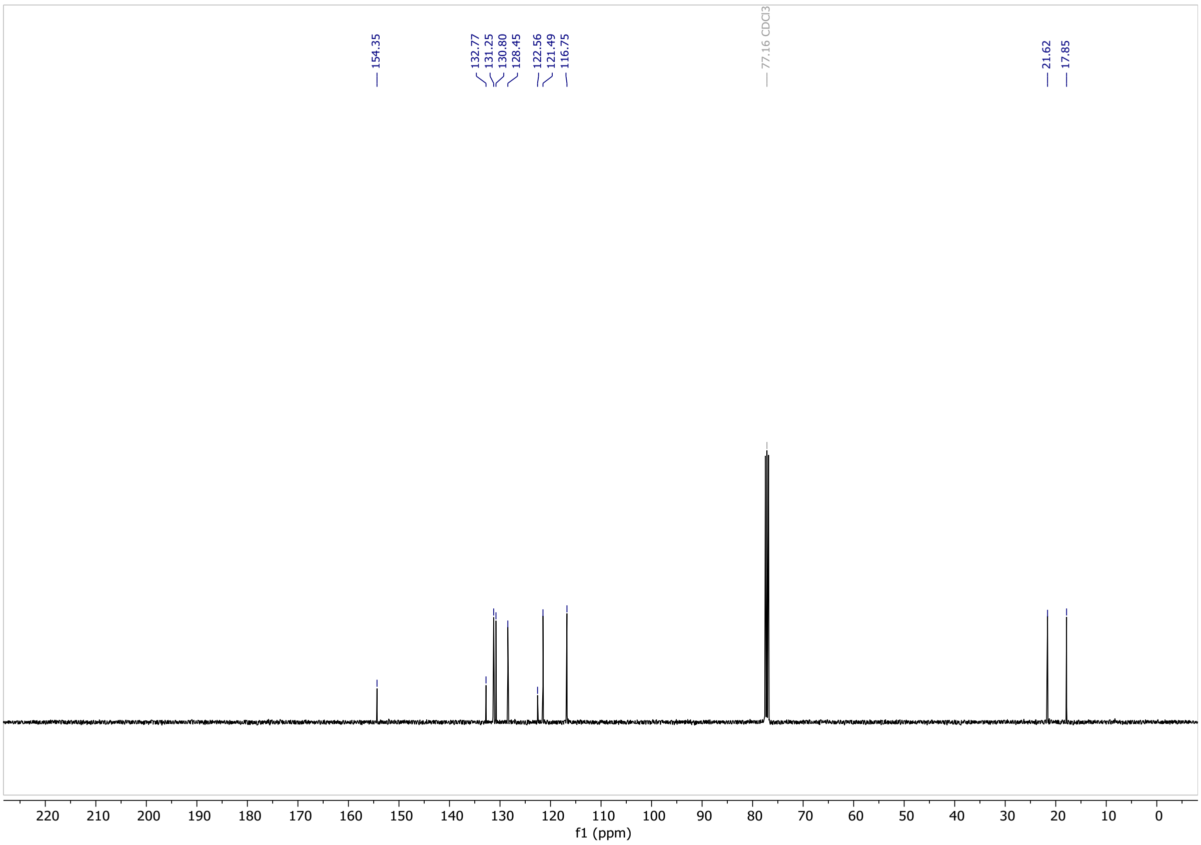

(*E*)-3,6-Dichloro-2-(pent-3-en-2-yl)phenol (SI8)

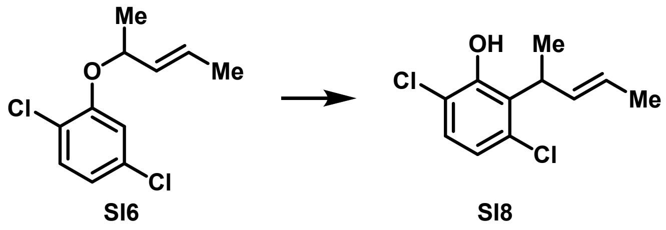

The title compound was synthesized from **SI6** (35 mg, 0.15 mmol) following **general procedure B**. The reaction was directly purified by column chromatography (petroleum ether/ethyl acetate 30:1) to provide the *ortho*-product **SI8** as colorless oil in 63% yield (22 mg, 0.10 mmol.

^{1}H NMR (400 MHz, $CDCl_3$) δ 7.11 (d, *J* = 8.6 Hz, 1H), 6.90 (d, *J* = 8.7 Hz, 1H), 6.11 (s, 1H), 5.92 (ddq, *J* = 15.6, 5.9, 1.6 Hz, 1H), 5.69 (dqd, *J* = 15.5, 6.4, 1.7 Hz, 1H), 4.20 (tddd, *J* = 8.8, 7.2, 5.7, 1.6 Hz, 1H), 1.73 (dt, *J* = 6.4, 1.6 Hz, 3H), 1.44 – 1.36 (m, 3H).

^{13}C NMR (101 MHz, $CDCl_3$) δ 150.9, 133.0, 132.6, 130.5, 127.3, 126.4, 122.1, 119.7, 37.4, 18.0, 17.3.

HRMS (ESI): exact mass calculated for $C_{11}H_{11}Cl_2O^-$ [(M - H)$^-$], 229.0192 (100.0%), 231.0163 (63.9%), 230.0226 (11.9%); found 229.0193 (100.0%), 231.0159 (64.2%), 230.0221 (11.6%).

^{1}H NMR (400 MHz, $CDCl_3$)

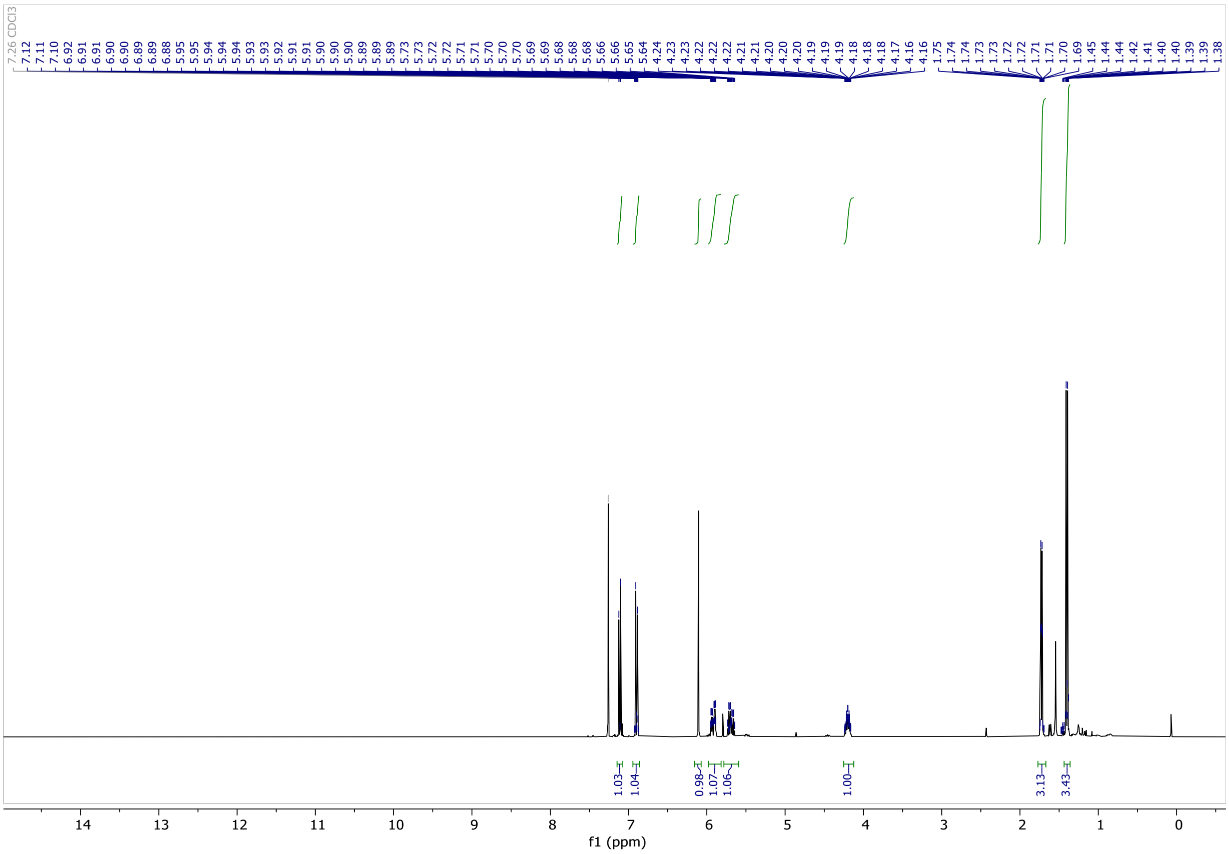

^{13}C NMR (101 MHz, $CDCl_3$)

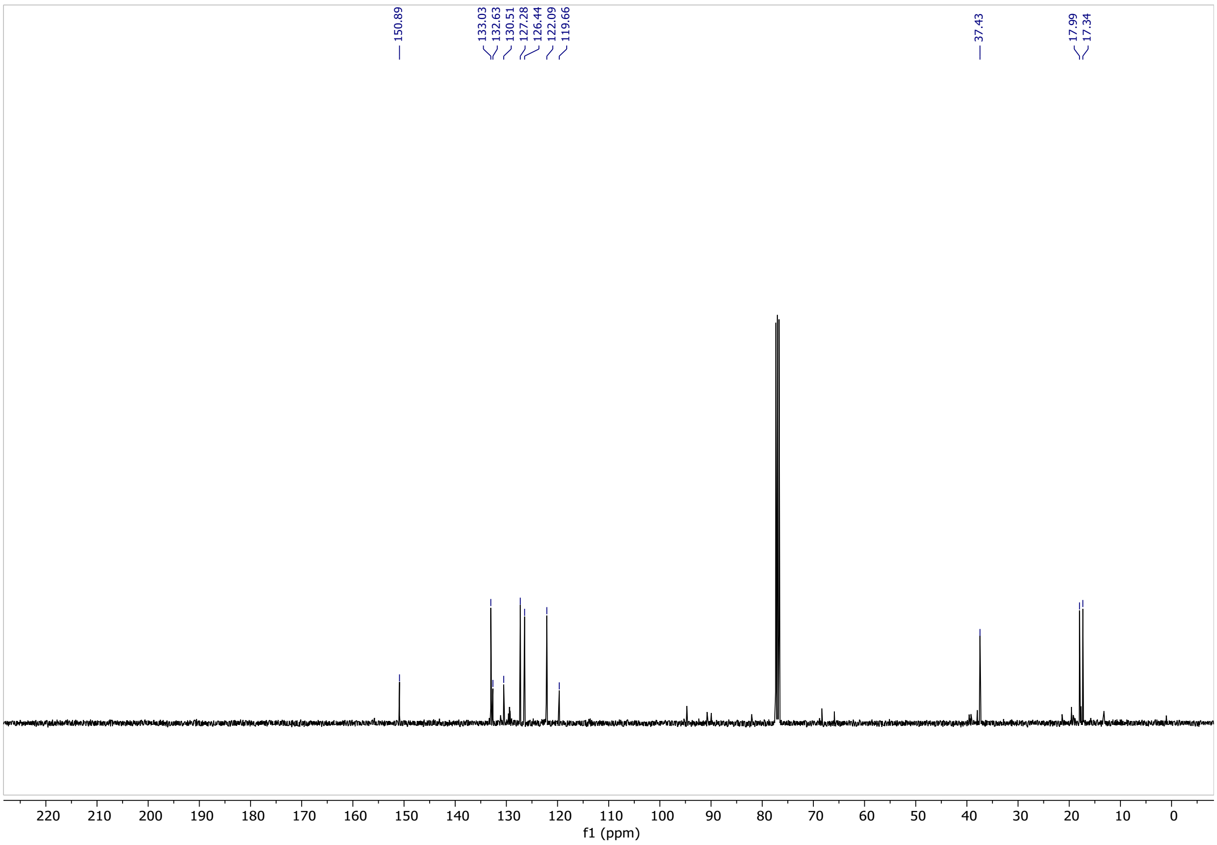

(*E*)-Triethyl((4-methoxy-3-(pent-3-en-2-yloxy)benzyl)oxy)silane (SI7)

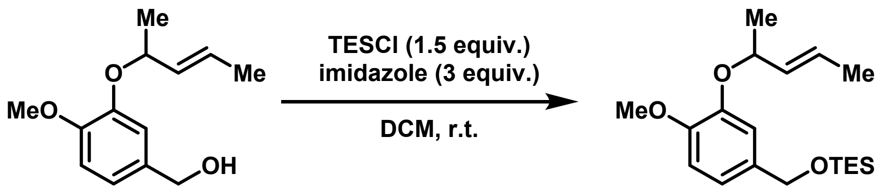

A round bottom flask was charged with compound **SI3** (207 mg, 0.93 mmol, 1 eq.) and imidazole (192 mg, 2.79 mmol, 3 eq.) dissolved in 25 mL dry DCM. TESCl (211 mg, 1.40 mmol, 1.5 eq.) was added dropwise to the colorless solution and immediate formation of precipitate was observed. After 20 minutes TLC (petroleum ether/ethyl acetate 5:1) confirmed full conversion and the reaction was quenched by addition of solid $NaHCO_3$. The mixture was filtered over silica with DCM and concentrated *in vacuo*. The desired product **SI7** was obtained in sufficient purity as colorless oil in 99% yield (310 mg, 0.92 mmol).

^{1}H NMR (400 MHz, $CDCl_3$) δ 6.93 (d, *J* = 1.7 Hz, 1H), 6.88 – 6.77 (m, 2H), 5.75 – 5.51 (m, 2H), 4.75 (p, *J* = 6.3 Hz, 1H), 4.63 (s, 2H), 3.84 (s, 3H), 1.67 (dt, *J* = 6.2, 1.0 Hz, 3H), 1.44 (d, *J* = 6.4 Hz, 3H), 0.95 (dt, *J* = 15.3, 7.9 Hz, 15H), 0.68 – 0.60 (m, 6H).

^{13}C NMR (101 MHz, $CDCl_3$) δ 149.4, 147.4, 133.9, 132.5, 127.4, 119.1, 114.9, 111.7, 75.9, 64.7, 56.2, 21.6, 17.8, 6.9, 4.7.

^{1}H NMR (400 MHz, $CDCl_3$)

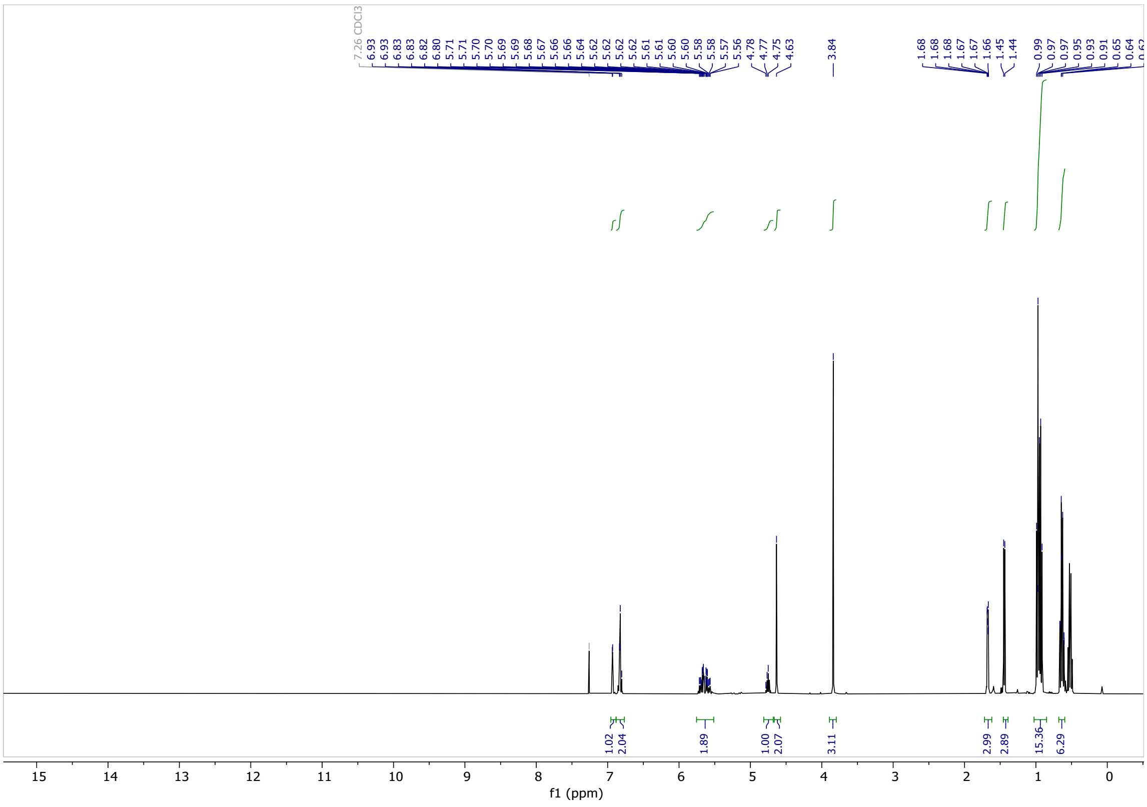

^{13}C NMR (101 MHz, $CDCl_3$)

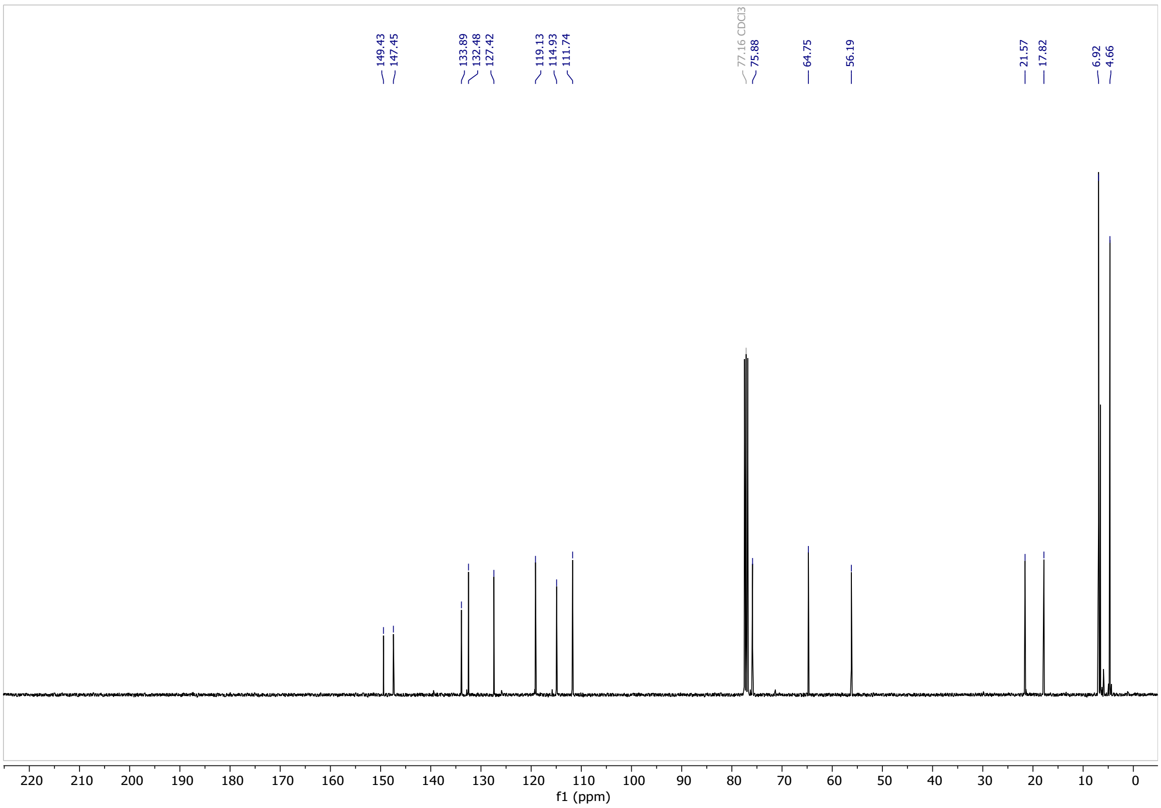

(*E*)-6-Methoxy-2-(pent-3-en-2-yl)-3-(((triethylsilyl)oxy)methyl)phenol (SI9)

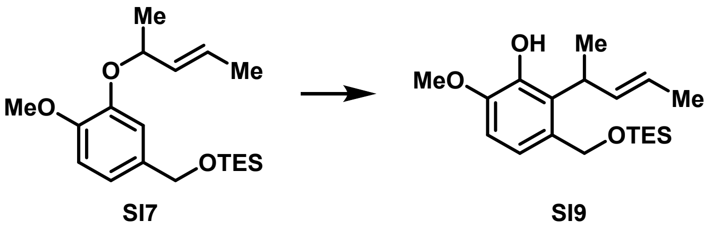

The title compound was synthesized from **SI7** (62 mg, 0.18 mmol) following **general procedure B**. The reaction was directly purified by column chromatography (petroleum ether/ethyl acetate 30:1) to provide the *ortho*-product **SI9** as colorless oil in 86% yield (53 mg, 0.16 mmol) as 1.4:1.0 *E/Z* mixture as measured by the ratio of the major (*E*)-isomer δ 1.67 (dd, *J* = 6.4, 1.6 Hz, 3H, integral= 4.22), to the minor (*Z*)-isomer δ 1.62 (dd, *J* = 6.9, 1.8 Hz, 3H, integral= 3.00); ^{1}H NMR (400 MHz, $CDCl_3$) δ 6.87 (d, *J* = 3.9 Hz, 1.06H), 6.85 (d, *J* = 3.8 Hz, 1.29H), 6.70 (d, *J* = 1.0 Hz, 1.29H), 6.67 (d, *J* = 1.0 Hz, 1.00H), 6.10 – 6.02 (m, 0.96H), 6.02 – 5.94 (m, 1.35H), 5.79 (d, *J* = 0.9 Hz, 1.28H), 5.78 (d, *J* = 1.0 Hz, 1.07H), 5.57 – 5.46 (m, 1.42H), 5.41 (ddt, *J* = 10.7, 8.2, 6.2 Hz, 0.99H), 4.80 (d, *J* = 12.2 Hz, 1.01H), 4.74 – 4.60 (m, 4.02H), 4.18 – 4.03 (m, 1.01H), 3.86 (s, 8.86H), 1.67 (dd, *J* = 6.4, 1.6 Hz, 4.22H), 1.62 (dd, *J* = 6.9, 1.8 Hz, 3.00H), 1.41 (ddd, *J* = 8.0, 7.0, 0.8 Hz, 7.45H), 1.03 – 0.89 (m, 37.19H), 0.65 (tdd, *J* = 8.6, 7.6, 5.1 Hz, 15.18H), 0.57 – 0.48 (m, 9.84H).

^{13}C NMR (101 MHz, $CDCl_3$) δ 146.6, 146.4, 144.1, 144.0, 134.9, 134.9, 131.8, 131.6, 129.9, 129.7, 124.0, 122.7, 119.5, 119.2, 108.0, 107.8, 63.8, 63.7, 56.1, 36.1, 31.4, 19.9, 18.9, 18.1, 13.1, 7.0, 6.9, 6.6, 4.7.

^{1}H NMR (400 MHz, $CDCl_3$)

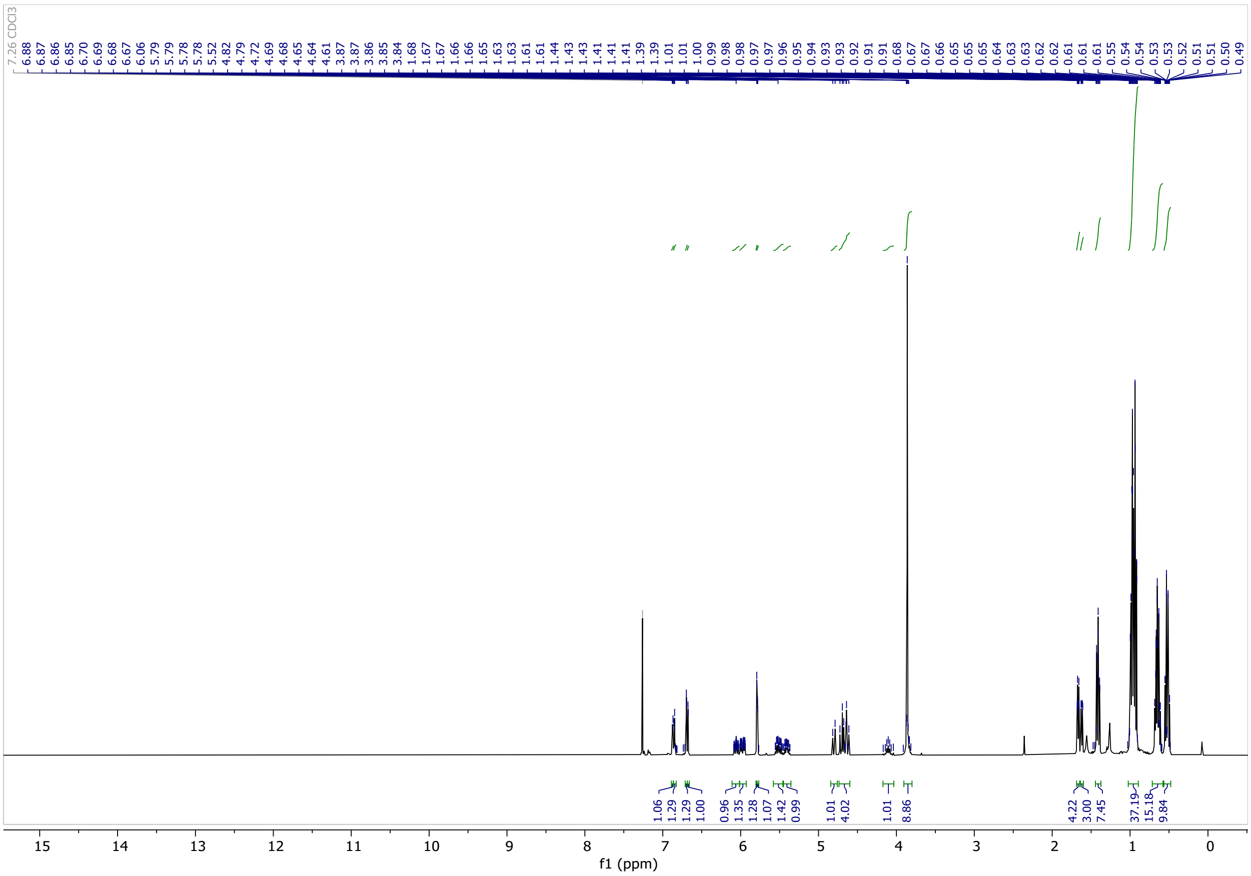

^{13}C NMR (101 MHz, $CDCl_3$)

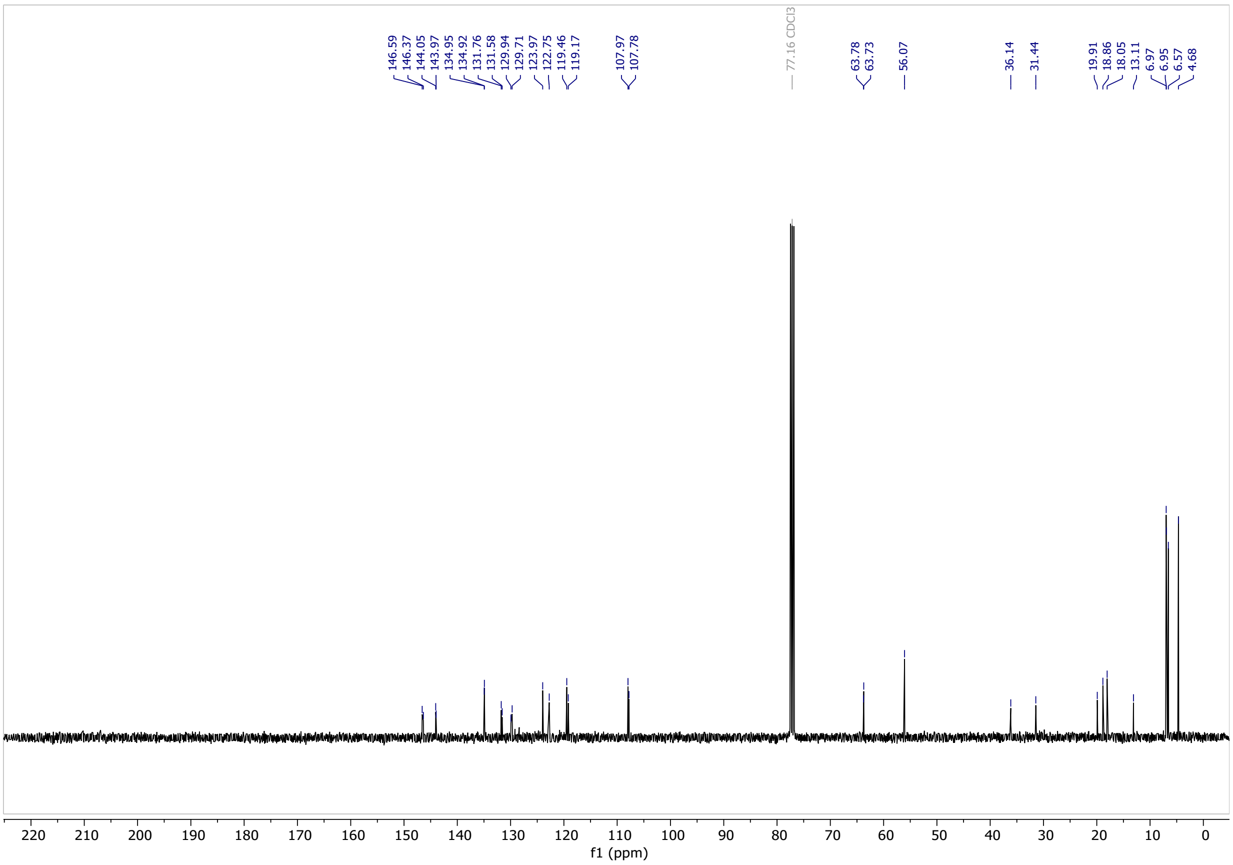

(*E*)-2-(Pent-3-en-2-yl)phenol (2e) & 2-(pentan-2-yl)phenol (SI10)

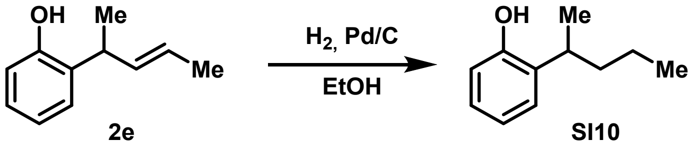

The title compound was synthesized from **1e** (100 mg, 0.62 mmol) following **general procedure B**. The reaction was directly purified by column chromatography (petroleum ether/ethyl acetate 40:1 to 20:1) to provide the product **2e** as pale orange oil as a 3.2:1 mixture of E/Z isomers. *Note:* To determine the depicted *ortho*-selectivity, the crude material was subjected to hydrogenation.

A 25 mL Schlenk flask was charged with crude **2e** in 6 mL dry EtOH and the mixture was degassed by vacuum/Ar-backfill (10 cycles). Then, Pd/C (10 % Pd on charcoal, 66 mg, 0.062 mmol, 0.1 equiv.) was added and the atmosphere was exchanged to hydrogen (ballon) by vacuum/H_2-backfill (10 cycles). After 7 hours, TLC (10:1, stained with anisaldehyde, SM in blue, product in red) confirmed full conversion. The atmosphere was changed again to Ar and the reaction mixture was filtered over a short plug of silica with ethyl acetate. Solvents were removed in vacuo, and the crude material was purified by column chromatography (10 g silica, petroleum ether/ ethyl acetate 40:1). The desired product **SI10** was obtained as colorless oil in 94 % yield over 2 steps (92 mg, 0.56 mmol).

^{1}H NMR (400 MHz, $CDCl_3$) δ 7.19 (dd, *J* = 7.7, 1.7 Hz, 1H), 7.08 (ddd, *J* = 8.1, 7.4, 1.7 Hz, 1H), 6.94 (td, *J* = 7.5, 1.2 Hz, 1H), 6.76 (dd, *J* = 8.0, 1.3 Hz, 1H), 4.76 (d, *J* = 3.9 Hz, 1H), 3.09 (h, *J* = 7.0 Hz, 1H), 1.73 – 1.50 (m, 2H), 1.40 – 1.23 (m, 5H), 0.91 (t, *J* = 7.4 Hz, 3H).

^{13}C NMR (101 MHz, $CDCl_3$) δ 153.0, 133.6, 127.3, 126.7, 121.1, 115.4, 39.5, 32.1, 21.0, 20.9, 14.3.

^{1}H NMR (400 MHz, $CDCl_3$)

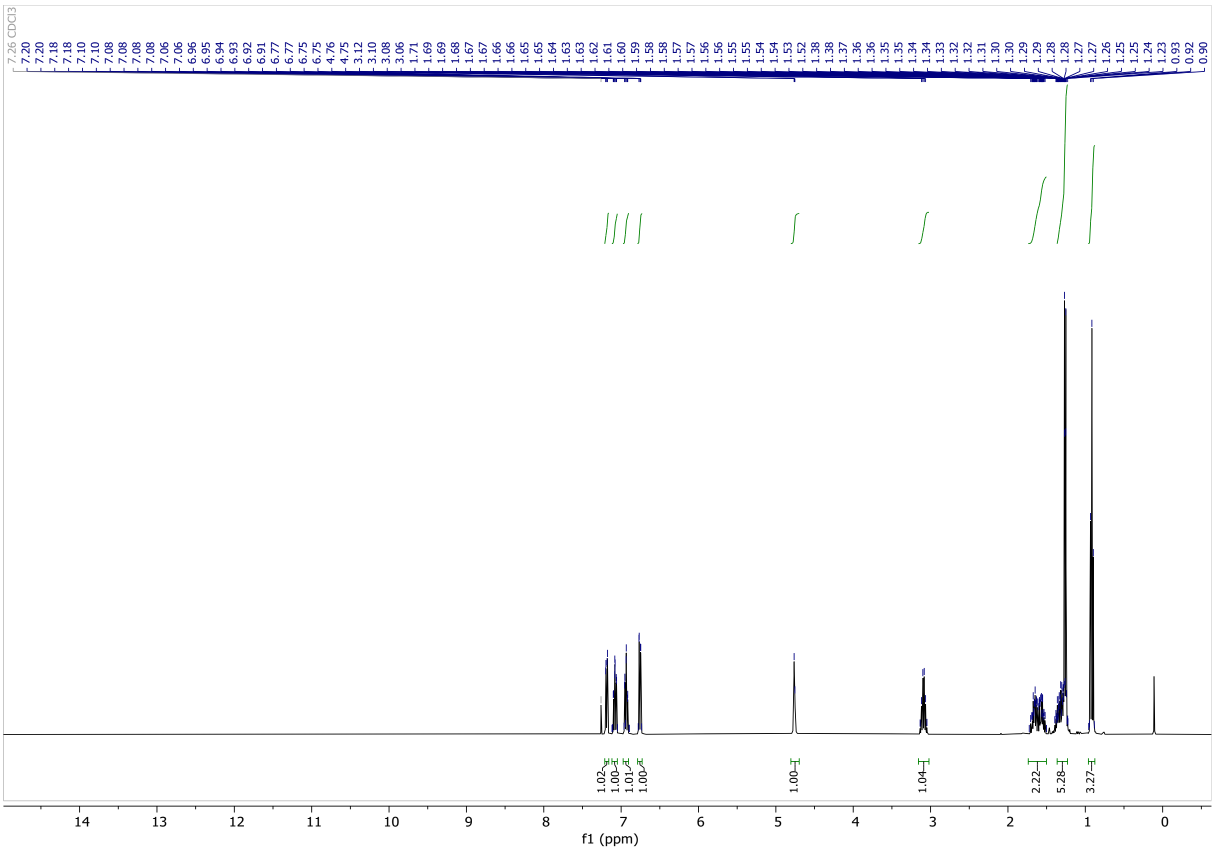

^{13}C NMR (101 MHz, $CDCl_3$)

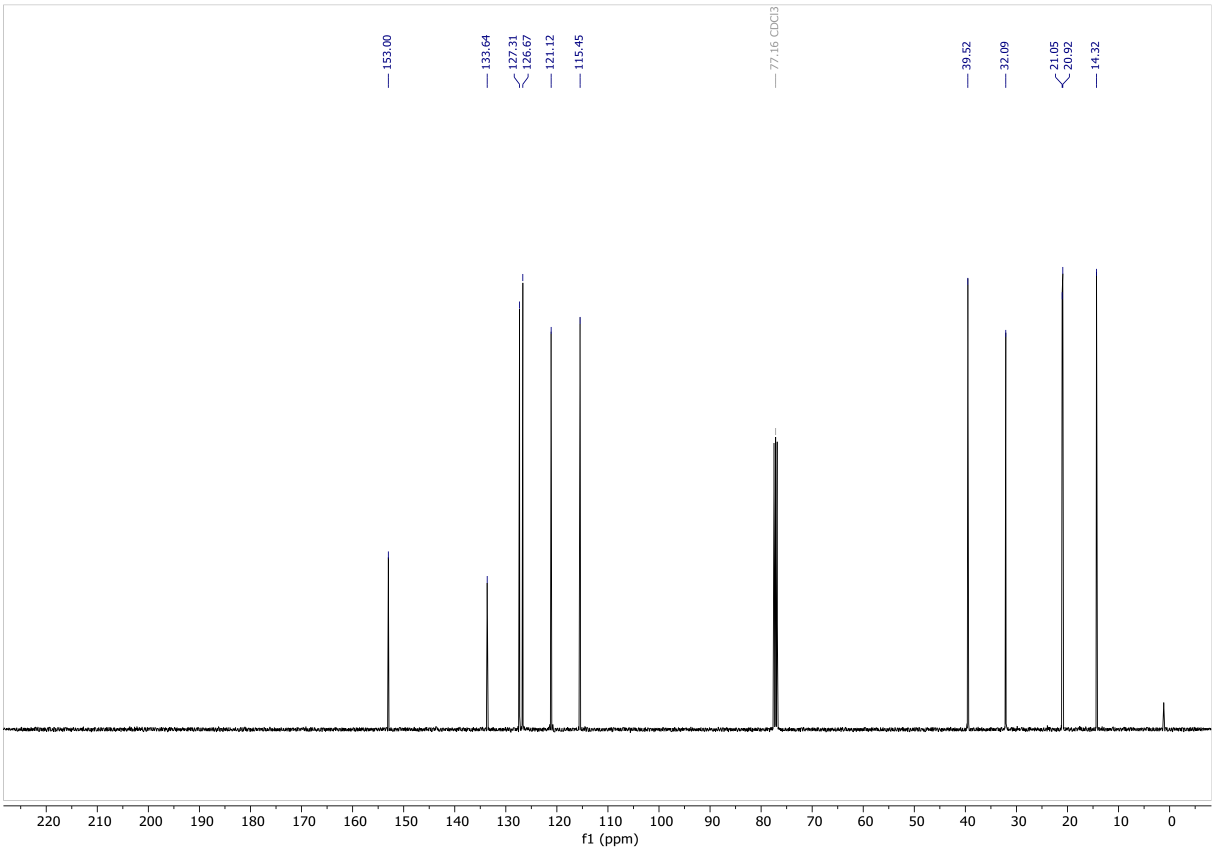

Supplement: QO-013-D6QO00040A-s001 [file QO-013-D6QO00040A-s001.pdf]
